# Demonstration of the utility of DOS-derived fragment libraries for rapid hit derivatisation in a multidirectional fashion†

**DOI:** 10.1039/d0sc01232g

**Published:** 2020-05-14

**Authors:** Sarah L. Kidd, Elaine Fowler, Till Reinhardt, Thomas Compton, Natalia Mateu, Hector Newman, Dom Bellini, Romain Talon, Joseph McLoughlin, Tobias Krojer, Anthony Aimon, Anthony Bradley, Michael Fairhead, Paul Brear, Laura Díaz-Sáez, Katherine McAuley, Hannah F. Sore, Andrew Madin, Daniel H. O'Donovan, Kilian V. M. Huber, Marko Hyvönen, Frank von Delft, Christopher G. Dowson, David R. Spring

**Affiliations:** Department of Chemistry, University of Cambridge Lensfield Road Cambridge CB2 1EW UK spring@ch.cam.ac.uk; School of Life Sciences, University of Warwick Coventry UK; Diamond Light Source Ltd., Harwell Science and Innovation Campus Didcot OX11 0QX UK; Structural Genomics Consortium (SGC), University of Oxford Oxford OX3 7DQ UK; Department of Biochemistry, University of Cambridge Tennis Court Road Cambridge CB2 1GA UK; Target Discovery Institute, Nuffield Department of Medicine, University of Oxford Oxford UK; Hit Discovery, Discovery Sciences, R&D, AstraZeneca Cambridge UK; Oncology R&D, AstraZeneca Cambridge CB4 0WG UK; Department of Biochemistry, University of Johannesburg Auckland Park 2006 South Africa

## Abstract

Organic synthesis underpins the evolution of weak fragment hits into potent lead compounds. Deficiencies within current screening collections often result in the requirement of significant synthetic investment to enable multidirectional fragment growth, limiting the efficiency of the hit evolution process. Diversity-oriented synthesis (DOS)-derived fragment libraries are constructed in an efficient and modular fashion and thus are well-suited to address this challenge. To demonstrate the effective nature of such libraries within fragment-based drug discovery, we herein describe the screening of a 40-member DOS library against three functionally distinct biological targets using X-Ray crystallography. Firstly, we demonstrate the importance for diversity in aiding hit identification with four fragment binders resulting from these efforts. Moreover, we also exemplify the ability to readily access a library of analogues from cheap commercially available materials, which ultimately enabled the exploration of a minimum of four synthetic vectors from each molecule. In total, 10–14 analogues of each hit were rapidly accessed in three to six synthetic steps. Thus, we showcase how DOS-derived fragment libraries enable efficient hit derivatisation and can be utilised to remove the synthetic limitations encountered in early stage fragment-based drug discovery.

## Abstract 2

Fragment-based screening of a shape-diverse collection yielded four hits against three proteins. Up to 14 analogues of each hit were rapidly generated, enabling four fragment growth vectors to be explored using inexpensive materials and reliable synthetic transformations.

## Graphical abstract

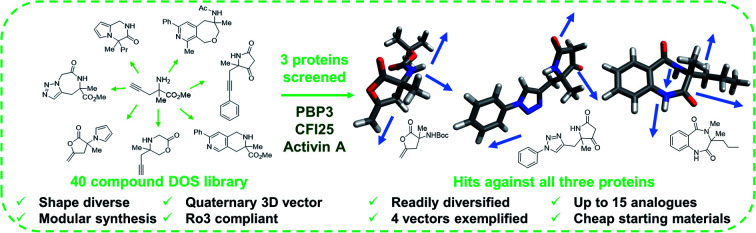

## Introduction

In the twenty years since its conception, fragment-based drug discovery (FBDD) has evolved into a mainstream approach to develop bioactive compounds. Three drugs originating from this technique have now been approved, whilst over 30 FBDD-derived clinical candidates remain under evaluation highlighting the effectiveness of this strategy.^1,2^ The fundamental challenge of developing potent molecules from the small, weakly bound initial hits that are identified by this method, however, should not be underestimated. Hits must be strategically optimised through fragment growing,^3^ linking^4^ or merging,^5^ often guided by structural information. In early development, this can be achieved using commercial compounds *via* an SAR-by catalogue approach,^6,7^ however, with less trivial fragments and as the research evolves, this rapidly becomes challenging. In this context, organic synthesis is a vital component that can contribute to the viability of a given early-stage drug discovery project.

Since the emergence of this strategy, physicochemical constraints have been used to assemble collections of molecules to screen based upon the properties of successful hits from early campaigns, now termed the Rule of Three (Ro3).^8^ Indeed, several commercial libraries adhering to these criteria are now readily available from many vendors. However, in recent years, in addition to the Ro3 compliance, synthetic accessibility and the ability to derivatise fragment molecules have been noted as important but arguably less-well represented features.^9,10^ As a result, calls from leaders within the field have focused on the necessity for the development of novel fragments featuring multidirectional exit vectors with synthetic tractability, including demonstration of available growth vectors.^10^ Thus, within the community there has been a sustained effort to design novel fragment libraries featuring 3-dimensional (3-D) elements^11–15^ (such as high fraction of sp^3^ carbons) and polar functionality,^16^ both of which enable facile fragment elaboration. Moreover, despite the debate within the literature on the relevance of 3-D fragments,^17,18^ recent examples have validated the utility of enriching screening libraries with these motifs.^19–21^

**Fig. 1:**
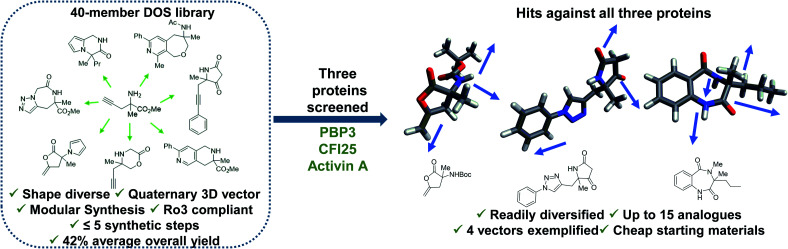
Demonstration of the utility of DOS libraries in X-ray based fragment screening and the ability to enable rapid analogue generation along multiple 3-D vectors around initial hits in a facile manner. See ref. 27 for chemistry towards the library.

Diversity-oriented synthesis (DOS) is a strategy by which libraries of structurally diverse compounds are constructed in a rapid and synthetically efficient manner through the employment of divergent synthetic manipulations.^22–25^ Whilst traditionally efforts in this field were focussed on larger molecules, in recent years the application of this methodology toward the synthesis of novel 3-D fragments has emerged.^26^ Herein, we demonstrate the relevance and utility of such libraries within FBDD. Firstly, we validate the importance for diversity in enabling identification of hits against several targets. In this case, fragment binders for three distinct proteins from different protein families were found from our recently published small but shape diverse 40-member library (Fig. 1).^27^ This included novel hits for challenging protein targets with no previously reported small molecule binders. Secondly, we highlight how molecules of this origin allow for analogues to be accessed in a synthetically efficient manner, including complex quaternary centre-containing compounds, in three to six steps from cheap (<£3 per gram) and readily available starting materials. Finally, we exemplify how the inherently modular chemistry can enable fragment elaboration from a variety of vectors, with derivatives of each hit exploring a minimum of four different positions.

## Results

With advances in foundational technologies such as third-generation synchrotrons and high-throughput technologies,^9,28–30^ X-ray crystallography methods have since become one of the most well-used techniques for hit finding within the field of FBDD.^31^ Thus, this method was selected as the primary screening technique conducted through a collaboration with the XChem platform.^32^ The DOS library was screened in a racemic fashion to provide both enantiomers and was used in a 500 mM§§Three examples were screened at 250 mM concentrations due to solubility limitations. format in d_6_-DMSO.

### Penicillin binding protein 3

The first protein screened with our DOS library was penicillin binding protein 3 (PBP3). The PBP family is responsible for the synthesis and cross-linking of peptidoglycan, the major component in the bacterial cell wall. The cell wall plays a pivotal role in controlling the shape and integrity of the cell and inhibition of the PBPs leads to cell lysis due to turgor pressure.^33,34^ The penicillin-binding domain contains a catalytic serine residue, which is vital for its function and a useful target for inhibition.^35^ Due to their essential role in cell division and elongation, PBPs are attractive targets for antibiotics with many β-lactam antibiotics developed for this purpose.^36^ However, the efficacy and wide-spread use of β-lactams has driven the alarming growth of bacterial resistance. Novel scaffolds capable of inhibiting the PBP family are greatly needed to overcome resistance mechanisms and restore activity against common infections.^37^ Due to this imminent need for new antibiotic leads, our DOS library was screened against *P. aeruginosa* PBP3 using the XChem platform.

**Fig. 2:**
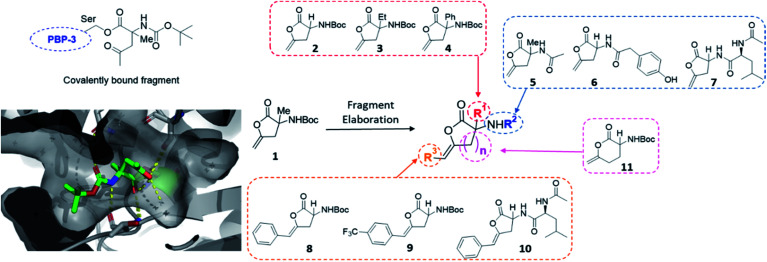
Initial hit compound **1** bound to PBP3, highlighted vectors suitable for diversification and the analogues synthesised to validate the initial hit.

This initial screen resulted in the serendipitous discovery of **1** as a covalent binder of PBP3 (PDB: 6Y6Z, Fig. 2), which strikingly was the first binder identified amongst approximately 1300 previous fragment soaks (see Table S1† for further details). The core enol lactone scaffold was found to react with Ser294, the catalytic residue found within the conserved SXXK motif of the β-lactam binding pocket. Upon incubation of the compound with the crystals, resulting electron density maps suggested a linear bound compound, which was hypothesised to result from lactone ring-opening followed by enol tautomerization to afford the linear ketone derivative. In addition to the covalent bond, hydrogen bonding interactions were observed with neighbouring residues Asn351, Ser349 and Thr487.

Considering these findings, it was proposed that the identified hit could be rapidly diversified through four primary vectors to comprehensively probe the PBP3 binding pocket. It was envisaged that the quaternary centre could be substituted at R^1^ with different alkyl (**2**, **3**) and aryl groups (**4**), amide couplings could be used to functionalise R^2^ (**5–7**), the terminal alkene could be substituted at R^3^ (**8**, **9**, **10**) and the lactone ring size could be altered (**11**). In this manner, exploring different ring sizes would allow for core scaffold modification, which is often difficult to incorporate into early fragment development. Importantly, substituents chosen for elaboration at R^2^ were designed with key β-lactam inhibitors in mind.

**Scheme 1:**
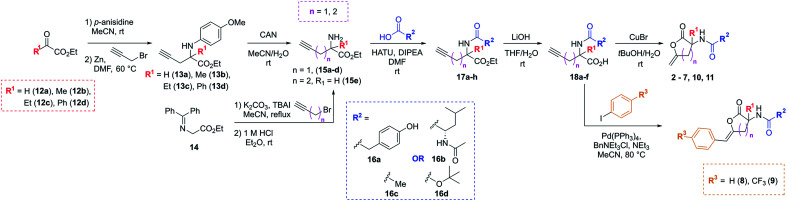
Synthetic route to analogues of the initial PBP3 hit **1** in five steps through key amine of type **15**.

The synthetic strategy used to access the proposed analogues was based upon the original chemistry used to prepare the library and utilised a common amino ester substrate in a divergent process (Scheme 1). All four vectors were accessed in five synthetic steps. Firstly, to diversify the quaternary centre, the R^1^ group in the commercially available ketoesters of type **12** could be varied. All examples shown here were purchased from Sigma Aldrich for under £3 per gram. To begin, **12a–d** were condensed with *p*-anisidine to generate the *p*-methoxyphenyl (PMP)-imine, which was subjected to a Barbier-type coupling to install the alkyne handle, giving protected amines **13a–d**. The PMP-group was subsequently removed from **13a–d** using cerium ammonium nitrate (CAN), giving amines **15a–d**. Alternatively, to access the tertiary carbon centre a simple substitution then deprotection of commercially available imine **14** allowed for easy access of amine **15e** in high yields. In this case, an alkyne featuring an extra methylene linker was employed to enable downstream formation of the larger six-membered ring derivative of **1**. From these amine intermediates, a diverse range of analogues were subsequently rapidly accessed using a simple toolkit of reliable chemistries. HATU-mediated amide couplings were exploited to connect a variety of motifs (R^2^) to the amine using substrates **16a–d**. Elaboration of this vector proved to be highly efficient since the final scaffold could be accessed in just three steps from the common amine intermediate, and hence many groups were explored with little synthetic effort. Following amide formation, the ester groups within **17a–h** were readily hydrolysed using LiOH, yielding acids **18a–f**. These final precursors could be cyclised using Cu(i)Br to form the unsaturated lactone scaffolds **2–7**, **10** and **11**. Alternatively, a procedure inspired by a reported one-pot Pd-catalysed cyclisation-coupling reaction^38,39^ was used to vary the R^3^ alkene substitution, enabling exploration of the final vector and providing access to **8–10**. This late-stage diversification provided extremely efficient access to a variety of novel analogues from cheap, commercially available aryl iodides.

Due to the inherent design features of our fragment library diversification of this fragment hit was simple, rapid and used robust chemistry. The 10 elaborated analogues were then screened using X-ray crystallography to validate the initial hit and observe the effects of vector derivatisation on the PBP3 binding preference. All compounds except for **8–11** were identified as PBP3 binders using this method. This preliminary data proved to be extremely useful in validating this hit with all analogues covalently binding to Ser294 in a similar fashion to that of **1**, whilst the specificity for the 5-membered lactone and terminal alkene could be inferred.

**Fig. 3:**
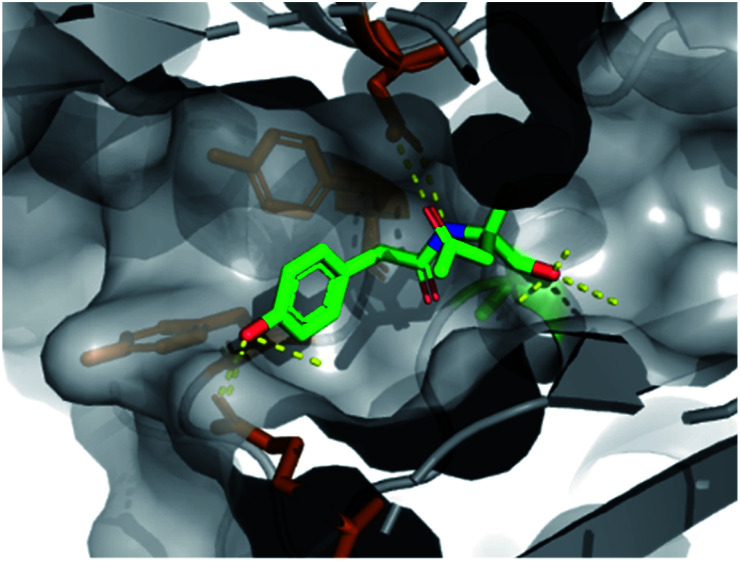
X-ray structure of **6** bound to PBP3 with conserved H-bonds highlighted.

We found that fragment elaboration from the amine vector (R^2^) was well-tolerated, including a variety of functionalities and sizes (**5–7**). Interestingly, the phenol group of **6** was found to project into a hydrophobic cleft within the pocket, appearing to make π–π interactions with proximal aromatic residues Tyr407 and Tyr409 (PDB: 6Y6U, Fig. 3, orange sticks), whilst maintaining previously observed hydrogen bonding interactions. These additional π–π interactions could prove extremely useful for further medicinal chemistry efforts in the downstream fragment evolution process.

### CFI_25_

To further demonstrate the utility of our library a second target CFI_25_ (cleavage factor 25 kDa), an essential sub-unit of the pre-mRNA cleavage factor Im, was screened. This heterotetramic complex comprises two units of CFI_25_ with two further units of either CFI_59_ or CFI_68_.^40,41^ Numerous studies have shown CFI_25_ to play a key role in determining the size of the 3′ untranslated region of mRNA, due to its involvement in the alternative polyadenylation (APA).^42^ This important mechanism is involved in gene regulation, ultimately contributing to the generation of different mRNA isoforms.^43^ Crucially, several studies have implicated CFI_25_ in oncology^44^ and neuropsychiatric disease^45^ settings, yet to date no small molecule modulators of this target are known to enable the further elucidation of its function. Thus, this served as an interesting target to explore with our novel DOS fragment library.

**Fig. 4:**
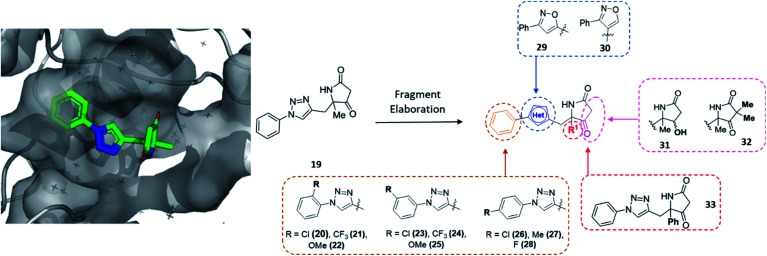
Initial hit compound **19** bound to CFI_25_, highlighted vectors suitable for diversification and the analogues synthesised to validate the initial hit.

Upon analysis of the resulting PanDDA event maps,^46^ two X-ray hits were identified (PDBs: 5R4P and 5R4Q, Fig. 4 and S1†) in a putative allosteric site away from the known mRNA substrate channel.^41,47^ Importantly, as a result of the diverse nature of the DOS library these hits related to distinctly different chemotypes, highlighting the potential of this collection to deliver hits of varied molecular architecture.

To exemplify the ability of modular DOS methodologies to enable rapid construction of varied analogues, hit **19** was further investigated. The amenability of the DOS chemistry toward multidirectional vector growth could be demonstrated *via* derivatisation to almost every functionality within **19** (Fig. 4). Specifically, in line with the structural data, it was thought that these investigations could include modification of the benzene ring through substitution (**20–28**), variation in the bridging heterocycle (**29**, **30**), derivatisation of the pyrrolidinone heterocycle *via* α-alkylation or ketone modification (**31**, **32**) and finally modification of the quaternary substituent R^1^ (**33**).

**Scheme 2:**
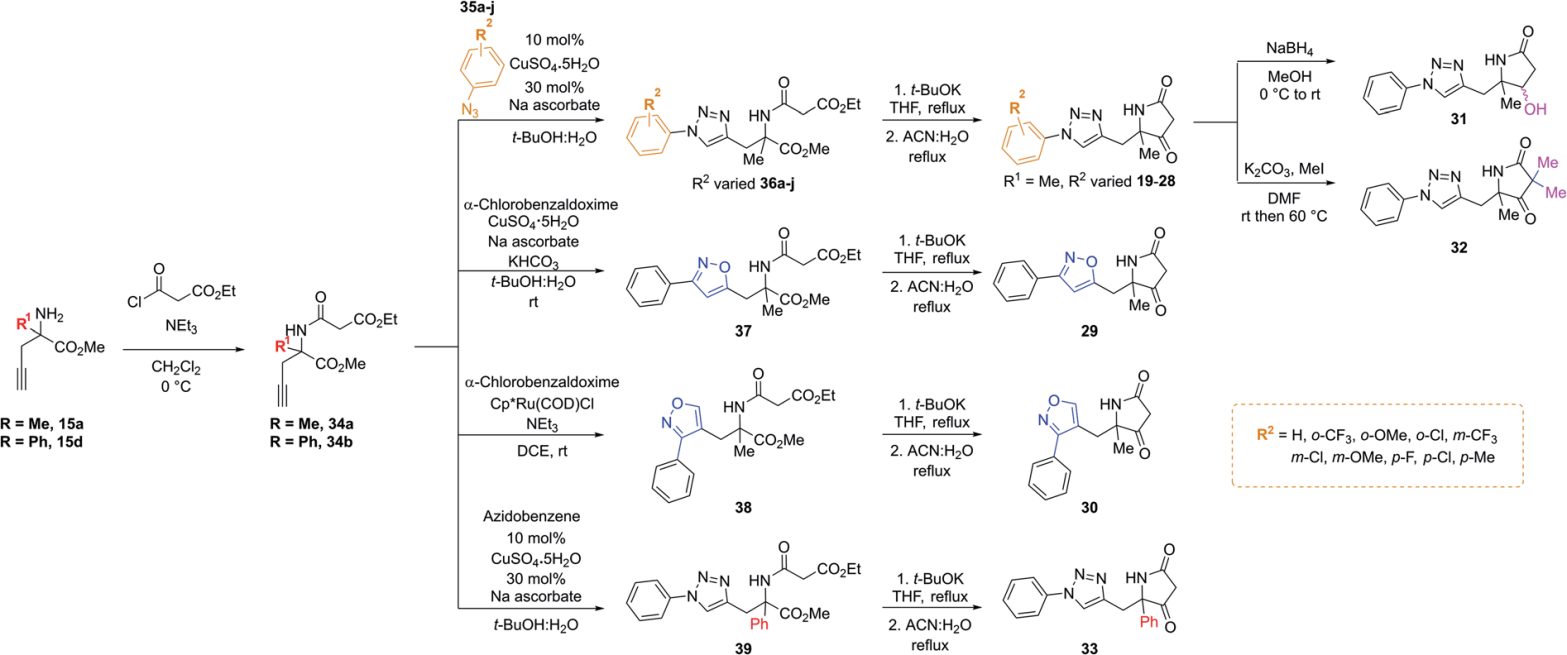
A divergent scheme was harnessed to access analogues **20–33** starting from the key quaternary amine intermediate of type **15**.

Importantly, in an analogous fashion to the explorations around **1** all derivatives were directly formed from the same quaternary amine intermediates of type **15** (Scheme 2). Firstly, to access analogues bearing substituents (R^2^) on the benzene ring amine **15a** was acylated to give amide **34a**. Next, Cu-mediated click chemistry was performed on **34a** using a variety commercially available substituted azides **35a–j**. In all cases, the resultant triazole products **36a–j** were obtained in good yields. Next, precursors **36a–j** were taken forward for cyclisation to afford the desired pyrrolidinone analogues **20–28***via* subjection to Dieckmann condensation conditions followed by thermal decarboxylation, which proceeded with good yields.

Next, using **34a** in the same click reaction but exchanging the azide component to α-chlorobenzaldoxime^48^ furnished the 1,4-substituted isoxazole intermediate **37**, which could again be cyclised by employing the same conditions to afford **29**. Alternatively, the 1,5-isoxazole variant could be obtained using the same strategy but using a Ru-based catalyst,^49^ affording **38**. Once more, Dieckmann condensation followed by decarboxylation yielded **30**. Finally, derivatives containing pyrrolidinone modifications were accessed through a late-stage modification strategy from **19** through either ketone reduction to give **31**, as a diastereomeric mixture, or α-deprotonation followed methylation to give **32**. In a similar fashion, modifying the R^1^ position could be achieved using the phenyl quaternary amine **15d**, which was subjected to the above sequence to give **34b** followed by **39**, and cyclised to give **33**.

**Fig. 5:**
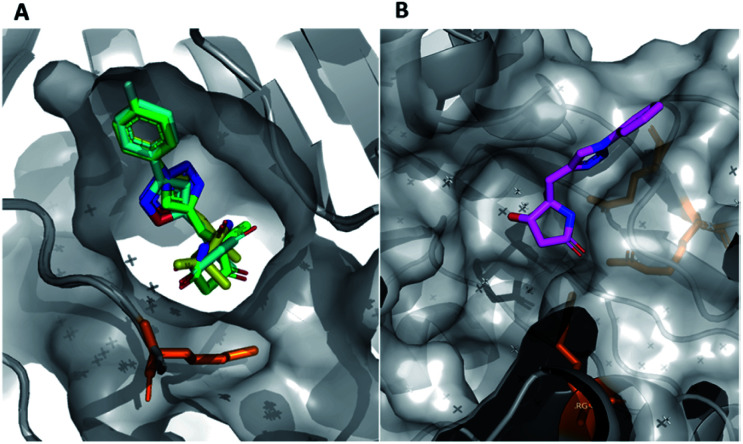
(A) Hits that bound in allosteric site, **28** = green sticks, **29** = cyan sticks, **32** = yellow sticks and (B) **31** was found to bind in the substrate channel.

In this example, the highly modular and divergent DOS strategy successfully enabled the rapid synthesis of 14 derivatives of hit **19**. These analogues were subsequently screened for binding using a further round of X-ray crystallography. This data revealed of the nine substituted aromatic analogues (**20–28**), only the *p*-fluorine analogue **28** was tolerated within the crystals (PDB: 5R4T, Fig. 5A, green sticks). Here, it was found that the aromatic portion of the molecule bound in a similar fashion to initial hit **19**, with the amide carbonyl interacting with Lys56 within the protein backbone. Similarly, the binding of **29** (PDB: 5R4U, Fig. 5A, cyan sticks) revealed that the alternative isoxazole bridging heterocycle was also tolerated, again in a similar binding pose to the original hit **19**. Importantly, selectivity for the 1,4-regioisomer could be inferred from these results since no binding of the 1,5-isomer **30** was identified. In an analogous fashion, **32** also exhibited this binding mode (PDB: 5R4R, Fig. 5A, yellow sticks). Interestingly, in this case the gem-dimethyl substituents and quaternary centre were oriented toward different channels within the protein, suggesting these positions could be utilised as two alternative 3-D growth vectors from the molecule.

Conversely, soaking of **31** revealed this compound instead bound within the distal mRNA substrate channel with a putative polar interaction between the amide carbonyl and the key Arg63 residue known to mediate binding of the UUGUAU RNA motif (PDB: 5R4S, Fig. 5B, binding protein residues in orange).^41,47^ Additional interactions toward Arg150 and Gln157 further stabilised this binding. It is worth mentioning, as with all bound derivatives, the electron density for the aromatic region proved to be much more defined, whilst that of the quaternary heterocycle was more ambiguous to assign. Thus, whilst these interactions could be hypothesised, screening of the single enantiomer or diastereomer variants of all four binders would provide vital information about the true binding preference and spatial orientation of the heterocycle. Building on previous research in the field of DOS fragments for hit evolution,^50^ in this example we have demonstrated how this can be achieved in a multidirectional fashion through leveraging the inherent modularity, the quaternary motif and sp^3^ carbons to provide insights into the most effective strategy for fragment growth.

### Activin A

**Fig. 6:**
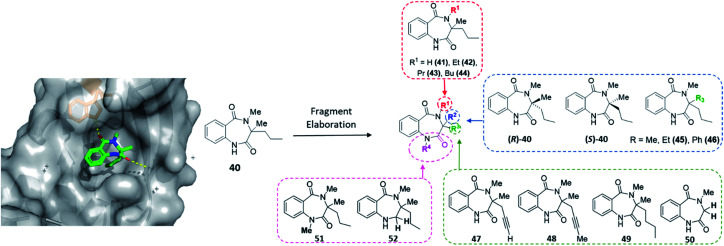
Initial hit compound **40** bound to activin A, highlighted vectors suitable for diversification and the analogues (**41–52**) synthesised to validate initial hit.

The final protein to be screened against our DOS-fragment library was activin A. Activins are members of the transforming growth factor β (TGF-β) superfamily of growth factors, which play essential roles in homeostasis and development and have been studied for many years.^51^ Research has shown activins mediate an intramolecular signalling cascade *via* binding of the extracellular domains of transmembrane serine/threonine kinases known as type I or type II receptors, ultimately conducting the phosphorylation of Smad proteins involved in target gene expression.^52–54^ Importantly, in this context, binding of the type II receptors has been shown to be crucial for type I receptor binding and therefore vital to initiate the first step of this signalling pathway. Several studies have associated the role of activin A signalling with the regulation of embryogenesis, stem cell differentiation and wound healing, among other processes. Moreover, dysregulation of activin A signalling or expression has been linked to human diseases such as inflammatory conditions, cancer and fibrodysplasia ossificans progressiva.^55–58^ Nevertheless, despite the potential of this target, to the best of our knowledge no small molecule modulators of this protein exist to enable further investigations into the associated biology. Thus, an XChem screen was conducted leading to the identification of **40** as a binding partner for activin A (PDB: 6Y6N, Fig. 6). This data suggested a key hydrogen bonding interaction between the benzylic amide carbonyl and the Trp28 residue within the site. This pocket is in the predicted binding sites for the activin A type I receptor ACVR1B/ALK4, proving an interesting avenue to pursue.

**Scheme 3:**
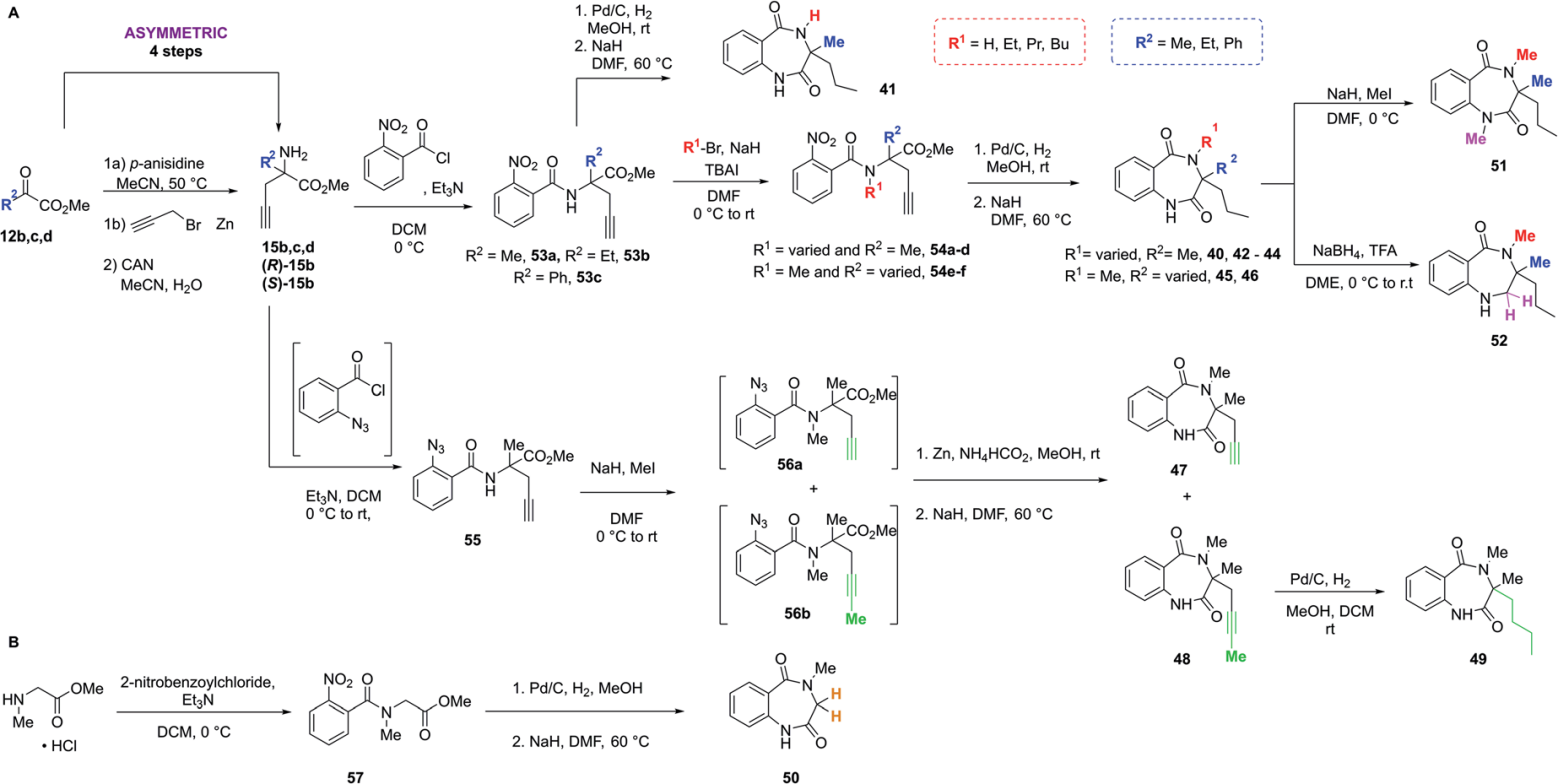
Two synthetic strategies to access analogues of **40**, (A) *via* key amine **15**, (B) from sarcosine methyl ester hydrochloride.

With the crystal structure and modular DOS route in mind, several analogues were once more explored to showcase the chemistry. It was proposed that the benzodiazepine core of the molecule provided an opportunity for several points of derivatisation, such as simple *N*-alkylation (R^1^) to form **41–44**, quaternary substituent modifications at R^2^ (**45**, **46**), including enantiopure derivatives ((*R*)/(*S*)-**40**), alkyne chain modifications (R^3^, **47–49**), removal of the quaternary substituents (**50**) and finally amide modifications (R^4^) *e.g.***51** and **52**. Once more, this was proposed to commence *via* a divergent process (Scheme 3A), utilising the same key amine intermediates **15b–d**.

Firstly, analogues bearing *N*-amide substituent variations at the R^1^ position were pursued. Starting from **15b** the acylated intermediate **53a** was accessed in good yield. To form **41** with R^1^ as hydrogen, **53a** was subjected to nitro reduction using palladium catalysis and finally hydride-mediated cyclisation of the resultant amine toward the remaining ester functionality. Alternatively, alkylation of **53a** with a variety of alkyl halides and catalytic TBAI afforded **54a–d**. These acyclic precursors could then undergo the same synthetic sequence of reduction and cyclisation to give **42–44**. Following this synthetic route, R^2^ was explored using amines **15c** and **15d**, bearing variation of the quaternary substituent. These were converted to **54e** and **54f**, followed by **45** and **46** as previously described.

Next, to showcase the ability to readily access enantiopure derivatives of both the key amine **15b** and related analogues, asymmetric routes to both (*R*)- and (*S*)-**15b** were pursued (synthetic procedure, see ESI†). Following previously established and reported chemistry from within the group,^27^ these variants were also rapidly accessed from the same commercially available ketoester starting material **12b** in just four steps. Following the previously described procedure, both enantiomers were converted to (*R*)-**40** and (*S*)-**40***via* (*R*)- or (*S*)-**53a** and -**54a**.

To modify the propyl chain (R^3^) of **40** and form **47–49**, in this instance **15b** was acylated with 2-azidobenzoyl chloride to form **55**. Surprisingly, upon subjection of this to the standard methylation procedure, both **56a** and **56b** were formed as an inseparable mixture of products. At this stage, the crude material was telescoped into the zinc-mediated reduction step, followed by cyclisation to generate separable material. Indeed, both **47** and **48** were isolated. To further explore this vector in a divergent fashion, the alkyne moiety within **48** was reduced using palladium to give **49**. Alternatively, to remove both substituents from the quaternary position (Scheme 3B), sarcosine methyl ester hydrochloride could be utilised, which was acylated to give **57** before reduction and cyclisation yielded **50**. Finally, two late-stage diversifications of **40** were used to explore R^4^. This included further alkylation of the amide to give **51** and selective reduction of the unsubstituted amide to afford **52**.

**Fig. 7:**
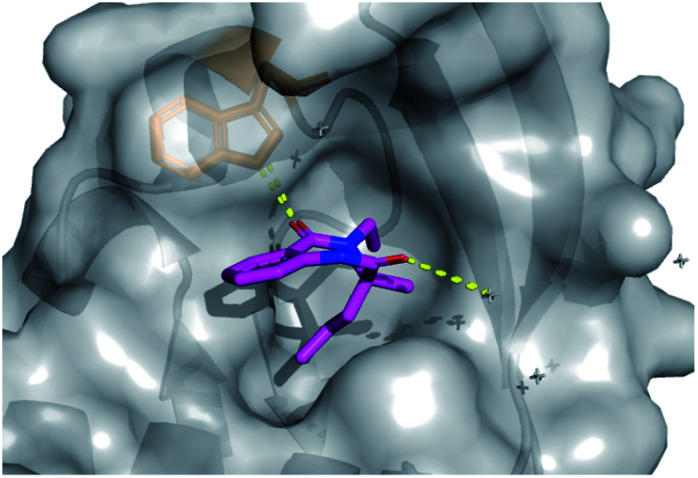
Follow-up analogue **42** bound to activin A with conserved H-bond to Trp28 highlighted.

In total four vectors of the molecule were explored using the 14 analogues described. Once more, these compounds were rapidly accessed *via* short synthetic sequences from commercial materials, highlighting the utility of the chemistry described. In a subsequent round of crystallography, analogue **42** was found to bind in the same pocket as the original hit, with the key H-bond interaction toward the Trp28 residue conserved (PDB: 6Y6O, Fig. 7). This secondary binding data was useful to validate the original hit binding and show the ethyl variant to be tolerated, suggesting the substituted amide position to be viable growth vector for future synthetic efforts.

## Discussion

Herein, we have exemplified the ability of fragment-focused DOS libraries to deliver diverse hits across numerous targets from distinct protein families, despite originating from the same amino ester building block. Screening of our DOS library containing 40 compounds gave several structurally distinct and tangible leads across all proteins considered. Importantly, PBP3, CFI_25_, and activin A are indicators for completely unrelated therapeutic areas, and as such these hits have the potential to serve as novel starting points for the development of inhibitors or chemical probes for a variety of biological purposes. The hit identified against PBP3 binds through a covalent mechanism, exemplifying the utility of our library towards the discovery of novel covalent ligands, in addition to reversible binders. Indeed, similar electrophilic fragments have been demonstrated to have enhanced utility in probe development due to their high duration of action and potency.^59,60^ We also report, to the best of our knowledge, the first small molecule binders of CFI_25_ and activin A.^61^ Furthermore, subsequent screens using the DOS library have also proven successful in delivering novel hits against additional antibiotic targets, with active discovery projects stemming from these results.

For these three proteins, four hits were identified, three of which were then diversified to rapidly generate 10–14 analogues in just three to six steps. All ketoester starting materials described are commercially available, with costs under £3 per gram. Moreover, the reaction sequences used to access the key amine intermediates of type **15** were readily and reproducibly prepared on multi-gram scales. As a result, the timescales of downstream analogue formation could be further reduced since several analogues were accessed in a divergent manner from this material. It is worth mentioning, as some examples have highlighted, that removal of the quaternary substituent could also be utilised as a strategy to decrease the number of steps required to access analogues of this library. However, in all projects described this feature was retained to exemplify the ease of utilising this position as a growth vector. Indeed, derivatisation of an sp^3^ quaternary carbon centre could prove highly challenging for most fragment hits, yet due to the simple three-step procedure previously developed, we have demonstrated how analogues of library members with this feature could be prepared with no additional route design.

The derivatives prepared explore at least four different fragment exit vectors utilising simple chemical transformations, offering significant incentives for library implementation in early FBDD programs. As discussed, one common hurdle within FBDD follow-up work remains the investigation of suitable points of hit modification to enable rapid and efficient exploration of a given binding pocket. Here, we have shown how novel libraries can be designed to alleviate this hurdle, allowing for facile initial exploratory chemistry, often where the molecules are low on the value-synthesis trajectory.^62^

In all cases, the preliminary X-ray data was used to deduce validation of each initial hit described, since at least one analogue of all three were additionally found to bind within the respective targets. In some cases, structural specificity could be inferred based upon the lack of density observed for some analogues. Thus, this initial scoping chemistry proved to be a valuable technique to also probe the binding pockets and derive potentially interesting vectors for further hit evolution during future project objectives.

In this work, we have demonstrated the advantages of using a DOS-derived library for FBDD lead validation and diversification. However, there are other factors which also limit hit progression in FBDD, including the difficulty in attaining additional biophysical characterisation required to generate structure–activity relationship data. The hits and follow-up compounds described in this work are currently under further investigation, with the overall aim to confirm binding in biophysical assays and ultimately produce potent lead compounds for each protein.

## Conclusions

Herein, we have demonstrated that DOS-derived libraries are useful tools for the generation of novel hits across a variety of different biological targets. We identified four hits for PBP3, CFI_25_, and activin A, all of which are functionally diverse proteins with great relevance for developing novel therapeutics as well as biological function elucidation. This further strengthens the precedence for incorporation of 3-D, diverse fragments within screening collections to augment existing commercial compounds.

We also evidence how the strategic design of novel libraries to incorporate modularity, whilst maintaining complexity, can result in alleviating chemistry as a limiting factor in early discovery projects. In this case, DOS methodology was exploited to facilitate rapid fragment elaboration, with up to 14 analogues of each hit readily accessed in short synthetic sequences despite the formation of challenging quaternary carbon centres. The additional advantage of these synthetic sequences is their use of cheap commercial materials, which reduces the requirement for lengthy and expensive initial explorative chemistry. The library described is currently available for screening *via* the XChem platform, where we hope it will be utilised by the scientific community to provide novel and more importantly tractable fragment hits for future development.

## Author contributions

Research was conceived by S. L. K., E. F., N. M., H. F. S. and D. R. S. H. N., D. B., R. T., J. M., A. A., A. B., P. B., L. D. S., K. M. contributed toward data acquisition, analysis, and interpretation. Synthesis and characterisation carried out by S. L. K., E. F., T. R., T. C. with support from D. H. O. D. and A. M. Protein expression was carried out by M. F., D. B., H. N., and J. M. X-ray screening was conducted by S. L. K, D. B., H. N., R. T., J. M., with support from K. V. M. H., M. H., F. D., and C. G. D. All authors provided comments and advice on the manuscript during construction.

## Conflicts of interest

There are no conflicts to declare.

## Supplementary Material

SC-011-D0SC01232G-s001
