# Supplementary material for: Demonstration of the utility of DOS-derived fragment libraries for rapid hit derivatisation in a multidirectional fashion
Source: Chem Sci. 2020 May 14;11(39):10792–801. doi: 10.1039/d0sc01232g (PMC8162264; doi:10.1039/d0sc01232g)
Supplement: SC-011-D0SC01232G-s001 [file SC-011-D0SC01232G-s001.pdf]

Electronic Supplementary Material (ESI) for Chemical Science.
This journal is © The Royal Society of Chemistry 2020

Demonstration of the utility of DOS-derived fragment libraries for rapid hit derivatisation in a multidirectional fashion

Sarah L. Kidd,‡[a] Elaine Fowler,‡[a] Till Reinhardt,[a] Thomas Compton,[a] Natalia Mateu,[a] Hector Newman,[bc] Dom Bellini,[b] Romain Talon,[cd] Joseph McLoughlin,[e] Tobias Krojer,[f] Anthony Aimon,[cd] Anthony Bradley,[c] Michael Fairhead,[d] Paul Brear,[e] Laura Díaz-Sáez,[df] Katherine McAuley,[c] Hannah F. Sore,[a] Andrew Madin,[g] Daniel H. O'Donovan,[g] Kilian V. M. Huber,[df] Marko Hyvönen,[e] Frank von Delft,[cdi] Christopher G. Dowson,[b] David R. Spring*[a]

[a.] Department of Chemistry, University of Cambridge, Lensfield Road, Cambridge, CB2 1EW, UK.

[b.] School of Life Sciences, University of Warwick, Coventry, UK

[c.] Diamond Light Source Ltd., Harwell Science and Innovation Campus, Didcot OX11 0QX, UK

[d.] Structural Genomics Consortium (SGC), University of Oxford, Oxford, OX3 7DQ, UK

[e.] Department of Biochemistry, University of Cambridge, Tennis Court Road, Cambridge, CB2 1GA, UK

[f.] Target Discovery Institute, Nuffield Department of Medicine, University of Oxford, Oxford, UK

[g.] AstraZeneca (UK) Ltd., 310 Cambridge Science Park, Cambridge, CB4 0FZ, UK

[h.] Department of Biochemistry, University of Johannesburg, Auckland Park 2006, South Africa

‡ These authors contributed equally to the work presented here.

* Correspondance: spring@ch.cam.ac.uk

SUPPLEMENTARY INFORMATION

Contents

1 Supplementary Figures 3

2 Supplementary Information: Crystallography 4

2.1 Target 1: Penicillin Binding Protein 3 4

2.2 Target 2: CFI_{25} 4

2.3 Target 3: Activin A 4

2.4 Compound Soaking, Data Collection and Analysis 5

2.5 Crystallographic data collection statistics Table_SI 1. Data collection and refinement statistics 5

3 Supplementary Information: Fragment Synthetic Schemes 11

3.1 Preparation of Common Amine Intermediates, 15 a-d 11

3.2 Hit 1: PBP3 11

3.3 Hit 2: CFI_{25} 13

3.4 Hit 3: Activin A 14

4 Supplementary Information: Synthetic Procedures 16

4.1 General Experimental Details 16

4.2 SI Hit 1: PBP3 Fragment Synthetic Procedures 18

4.3 Hit 2: CFI_{25} Fragment Synthetic Procedures 38

4.4 Hit 3: Activin A Fragment Synthetic Procedures 56

5 References 81

6 Supplementary Information: Spectra 82

1 Supplementary Figures

	Chiral Centres	Fsp3 C's
**DOS Library**	1.10	0.46
**Previous Screen**	0.92	0.44

Table S1. Comparison of key properties of the libraries screened against PBP3; Calculated using RDKit in KNIME[1] for our DOS library and 1312 covalent and non-covalent fragments from a mixture of commercial and academic libraries.

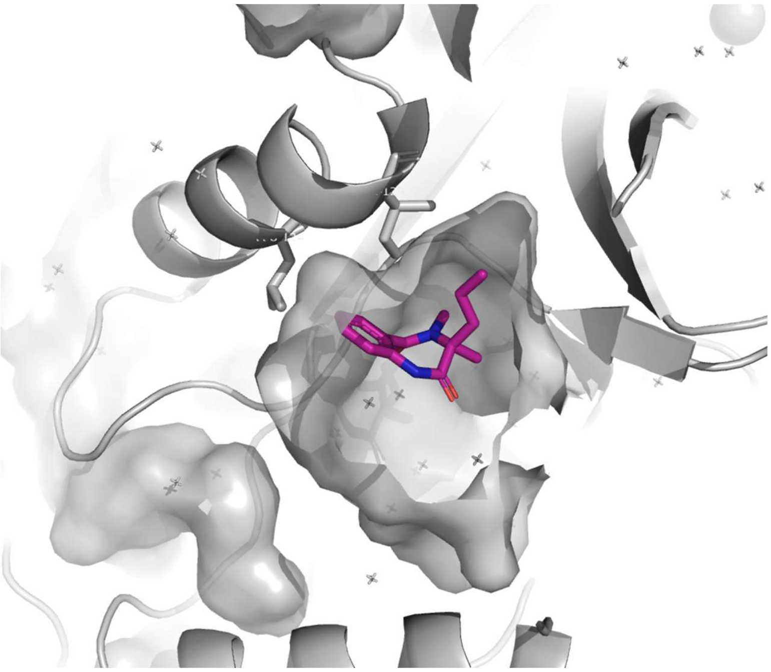

Figure S1. Additional hit **S1** bound to CFI_{25}.

2 Supplementary Information: Crystallography

2.1 Target 1: Penicillin Binding Protein 3

P. auruginosa PBP3 was expressed and purified as previously described.[2] PaPBP3 at 10 mg/mL in 10 mM Tris, pH 8.0 and 200 mM NaCl was crystallised with 25 % (w/v) polyethylene glycol 3350, 0.1 M Bis-Tris propane, 1% (w/v) protamine sulfate, pH 7.8 in a 1:1 ratio with a total volume of 2 µL by the hanging drop vapour-diffusion method. The crystals were cryo-cooled in liquid nitrogen in the same solution for data collection.

2.2 Target 2: CFI_{25}

CFI_{25} protein was expressed and purified at the Structural Genomics Consortium, Oxford, by Dr Michael Fairhead following the in-house protocol "PREPX" (Parallel rapid expression and purification of proteins for crystallography). The final protein buffer consisting of 10 mM HEPES pH 7.5, 0.5 M NaCl, 5% glycerol, 0.5 mM TCEP. Crystals were grown in SWISSCI 3 Lens crystallization sitting-drop plates at 20 °C by mixing 50–100 nL of 20 mg/ml protein solution in a 1:1 ratio with 50–100 nL reservoir solution consisting of 0.1 M sodium acetate pH 4.2-4.8, 0.1 M zinc acetate, 5–16%(w/v) PEG 3000 and placing the drops over 20 ml reservoir solution. First crystals appeared after 2 days but the high-resolution diffracting ones appeared in 5–6 days. CFI_{25} crystals diffracted to 1.5–1.8 Å resolution in space group P3221, with typical unit-cell parameters a = 59.6, c = 213.6 Å, α = 90.00, γ = 120.00 and with two CFI_{25} molecules in the asymmetric unit.

2.3 Target 3: Activin A

Activin A was cloned, expressed and purified as previously described.[3] Activin A crystals were grown from 200 nL protein (3.3 mg/mL dissolved in 20 % MeCN), 50 nL seed solution and 200 nL well solution. The well solution consisted of 1.4 M ammonium sulfate, 100 mM HEPES pH 7.4, 8 % DMSO, and the seed solution consisted of 200 nL of 1.55 M ammonium sulfate, 4 % PEG300, 100 mM HEPES pH 7.4, 8 % DMSO solution and 200 nL of mature activin A (3.6 mg/mL) dissolved in 20 % MeCN. The resulting crystals were soaked with a 40 mM compound solution.

2.4 Compound Soaking, Data Collection and Analysis

All compounds were dissolved in DMSO at a concentration of 500mM. In cases where compounds could not be dissolved at this concentration, the solution was further diluted to 250mM. Crystal soaking was performed by acoustic transfer using a Labcyte Echo 550.

X-ray diffraction data were collected on beamline I04-1 at Diamond Light Source and were processed using either:

(1) DIALS software; structures were solved by using programs from the CCP4 package. Models were iteratively refined and rebuilt by using Refmac5 and Coot programs. Ligand coordinates and restraints were generated from their SMILES strings using ELBOW software.

(2) Diamond autoprocessing pipeline, which utilizes xia2,[4] DIALS,[5] XDS,[6] POINTLESS,[7] and CCP4.[8] Electron-density maps were generated using XChemExplorer *via* DIMPLE.[9] Ligand restraints were generated with AceDRG[10] and ligand binding was detected with PanDDA,[11] with ligands built into PanDDA event maps. Iterative refinement and manual model correction was performed using REFMAC[12] and Coot,[13] respectively.

(3) Structures of activin A re-soaked with XChem **40** and follow-up molecule were determined separately using data collected at Diamond Light Source beamline I04-1. The data was processed with autoproc and scaled with aimless and structure solved using molecular replacement with 2arv as the search model. The structures were interatively refined with REFMAC and BUSTER and corrected manually using Coot. These structures have been deposited to Protein Data Bank under accession numbers 6Y6O and 6Y6N. The data collection and refinement statistics are show in table below.

2.5 Crystallographic data collection statistics Table_SI 1. Data collection and refinement statistics

2.5.1 PBP3 Data collection

**Ligand**	**1**	**6**
**PDB code**	**6Y6Z**	**6Y6U**
**Beamline**	DLS i04-1	DLS I03
**Wavelength**	0.916 Å	0.98 Å
**Resolution range (Å)§**	1.701 – 61.11 Å<br>(1.701- 1.82 Å)	1.55 – 61.10 Å<br>(1.55 – 1.69 Å)
**Space group**	P 21 21 21	P 21 21 21

**Cell (a b c) (Å)**	69.10 83.14 90.14	68.57 83.13 90.06
**Cell (α β γ) (°)**	90 90 90	90 90 90
**Unique reflections**	44456	449885
**Multiplicity[§]**	6.6 (6.2)	7.9 (7.7)
**Completeness (%)[§¶¶]**	94.9 (67.2)	95.5 (64.7)
**Mean I/sigI[§]**	11.8 (1.4)	17.5 (1.6)
**Rmeas[§§]**	0.099 (0.71)	0.055 (1.26)
**CC half [§]**	1.00 (0.65)	1.00 (0.71)
**Refinement**		
**Rwork††**	0.17	0.15
**Rfree†**	0.25	0.22
**RMS bonds[¶] (Å)**	0.010	0.010
**RMS angles[¶] (°)**	1.60	1.67
**Average B-factor ($Å^2$)**	30.75	41.95
**Ramachandran outliers**	1	0
**Rotamer outliers**	7	5
**Molecules in asymmetric unit**	1	1

[§]Parentheses indicate high resolution shell

[§§] Rmeas $=\{\sum_{hkl}\sqrt{n/(n-1)}[\sum^{n}_{j=1}|I_{hkl,j}-\langle I_{hkl}\rangle|]\}/\sum_{hkl}\sum_{j}I_{hkl,j}$

[††]Rwork =∑||Fobs| – |Fcalc||/|Fobs|×100

[†] Rfree, based on 5% of the total reflections

[¶] RMS deviation from ideality

[¶¶] Data was processed with STARANISO (Global Phasing) and completeness quoted here is the ellipsoidal completeness

2.5.2 CFI_{25} Data collection

Ligand	NUDT21A-x0266	NUDT21A-x0466
	5R4P	5R4Q
Beamline	DLS i04-1	DLS i04-1
Wavelength	0.9282 Å	0.9282 Å
Resolution range (Å)	51.74 - 1.78 Å (1.83 - 1.78 Å)	71.48 - 1.49 Å (1.53 - 1.49 Å)
Space group	P 32 2 1	P 32 2 1
Cell (a b c) Å	59.68 59.68 214.53	59.68 59.68 214.43
Cell (α β γ) (°)	90.00 90.00 120.00	90.00 90.00 120.00
Total reflections	421169 (32180)	706964 (43877)
Unique reflections	43715 (3147)	73717 (5353)
Multiplicity	9.60 (10.20)	9.60 (8.20)
Completeness (%)	100.00 (100.00)	100.00 (100.00)
Mean I/sigma(I)	12.40 (-)	17.90 (-)
R-sym	- (-)	- (-)
R-merge	0.128 (2.356)	0.053 (1.763)
R-rim	0.043 (0.769)	0.018 (0.658)
CC-half	0.999 (0.635)	0.999 (0.574)
R-factor	0.207 (0.317)	0.218 (0.351)
R-free	0.245 (0.396)	0.250 (0.353)
Number of total atoms	3470	3480
atoms for ligands	30	32
atoms for waters	255	255
Number of polymer residues	387	393
Wilson B-factor	-	-
Average B-factor	35.5	35.4
B-factor for ligands	66.1	71.3
B-factor for solvent	42.1	43.1
RMS(bonds)	0.01	0.01
RMS(bond angles)	1.509	1.84
RMS(dihedral angles)	9.958	10.415
Ramachandran favored (%)	-	-
Ramachandran outliers (%)	-	-
Clashscore	-	-

Ligand	NUDT21A-x1046	NUDT21A-x1047
	5R4R	5R4S
Beamline	DLS i04-1	DLS i04-1
Wavelength	0.9159 Å	0.9159 Å
Resolution range (Å)	70.95 - 1.50 Å (1.54 - 1.50 Å)	51.07 - 1.61 Å (1.65 - 1.61 Å)
Space group	P 32 2 1	P 32 2 1
Cell (a b c) Å	59.09 59.09 212.84	58.91 58.91 212.33
Cell (α β γ) (°)	90.00 90.00 120.00	90.00 90.00 120.00
Total reflections	669620 (42052)	552222 (40392)
Unique reflections	70428 (5105)	56721 (4136)
Multiplicity	9.50 (8.20)	9.70 (9.80)
Completeness (%)	100.00 (100.00)	100.00 (100.00)
Mean I/sigma(I)	14.60 (-)	12.40 (-)
R-sym	- (-)	- (-)
R-merge	0.066 (1.731)	0.082 (2.060)
R-rim	0.022 (0.641)	0.028 (0.690)
CC-half	0.999 (0.528)	0.998 (0.572)
R-factor	0.210 (0.344)	0.209 (0.349)
R-free	0.242 (0.374)	0.248 (0.349)
Number of total atoms	3548	3522
atoms for ligands	62	38
atoms for waters	255	246
Number of polymer residues	394	394
Wilson B-factor	-	-
Average B-factor	29.6	33.7
B-factor for ligands	53.3	66.4
B-factor for solvent	38.9	42.6
RMS(bonds)	0.011	0.01
RMS(bond angles)	1.754	1.692
RMS(dihedral angles)	7.447	7.682
Ramachandran favored (%)	-	-
Ramachandran outliers (%)	-	-
Clashscore	-	-

Ligand	NUDT21A-x1065	NUDT21A-x1068
	5R4T	5R4U
Beamline	DLS i04-1	DLS i04-1
Wavelength	0.9159 Å	0.9159 Å
Resolution range (Å)	70.99 - 1.68 Å (1.73 - 1.68 Å)	49.78 - 1.92 Å (1.97 - 1.92 Å)
Space group	P 32 2 1	P 32 2 1
Cell (a b c) Å	59.01 59.01 212.96	59.06 59.06 212.70
Cell (α β γ) (°)	90.00 90.00 120.00	90.00 90.00 120.00
Total reflections	478631 (60957)	310501 (20944)
Unique reflections	49996 (7167)	33630 (2375)
Multiplicity	9.60 (8.50)	9.20 (8.80)
Completeness (%)	100.00 (100.00)	98.80 (95.20)
Mean I/sigma(I)	13.20 (-)	10.30 (-)
R-sym	- (-)	- (-)
R-merge	0.077 (2.822)	0.189 (7.380)
R-rim	0.026 (1.026)	0.067 (2.648)
CC-half	0.999 (0.486)	0.995 (0.395)
R-factor	0.213 (0.437)	0.214 (0.409)
R-free	0.259 (0.454)	0.266 (0.411)
Number of total atoms	3545	3530
atoms for ligands	60	38
atoms for waters	247	254
Number of polymer residues	394	394
Wilson B-factor	-	-
Average B-factor	40	31.7
B-factor for ligands	67.8	60.7
B-factor for solvent	49.2	39.9
RMS(bonds)	0.008	0.008
RMS(bond angles)	1.628	1.538
RMS(dihedral angles)	7.349	7.978
Ramachandran favored (%)	-	-
Ramachandran outliers (%)	-	-
Clashscore	-	-

2.5.3 Activin A Data collection

Ligand	40	42
PDB code	6Y6N	6Y6O
Beamline	DLS i04-1	DLS i04-1
Wavelength	0.9786 Å	0.9786 Å
Resolution range	56.67 - 2.03 Å (2.14 - 2.03 Å)	74.71 - 2.04 Å (2.15 - 2.04 Å)
Space group	I 2 2 2	I 2 2 2
Cell (a b c) (Å)	64.25 96.95 120.27	64.05 96.94 117.24
Cell (α β γ) (°)	90.0 90.0 90.0	90.0 90.0 90.0
Total reflections	214143 (31874)	199275 (30679)
Unique reflections	24727 (3564)	23146 (3432)
Multiplicity	8.7 (8.9)	8.6 (8.9)
Completeness (%)	99.6 (100.0)	97.2 (100.0)
Mean I/sigma(I)	8.3 (1.0)	8.7 (0.9)
R-merge	0.182 (2.76)	0.194 (2.89)
R-pim	0.065 (0.96)	0.069 (1.01)
CC-half	0.996 (0.49)	0.997 (0.49)
Refinement:		
R-factor / Rfree	0.236 / 0.251	0.223 / 0.254
Number of total atoms	1835	1876
No of atoms for ligands	38	44
No of atoms for waters	43	50
Number of polymer residues	223	226
Average B-factor (Å^2)	55.6	54.3
B-factor for ligands (Å^2)	86.7	69.5
B-factor for solvent (Å^2)	53	45.9
RMS(bonds) (Å)	0.01	0.018
RMS(bond angles)	1.11	2.165
RMS(dihedral angles)	3.3	8.448

3 Supplementary Information: Fragment Synthetic Schemes

3.1 Preparation of Common Amine Intermediates, 15 a-d

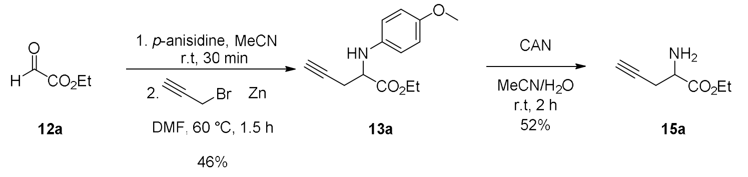

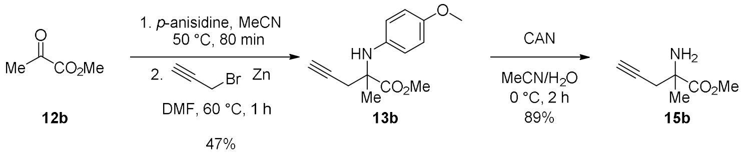

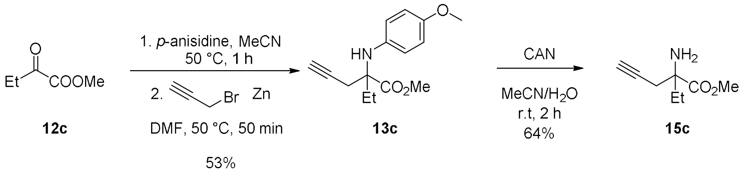

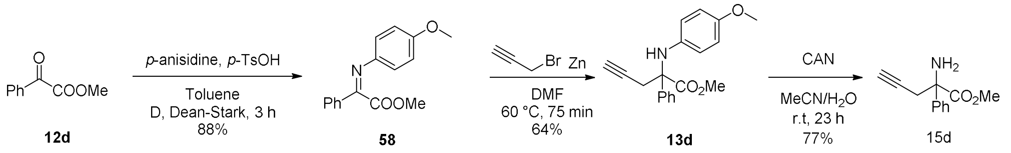

3.2 Hit 1: PBP3

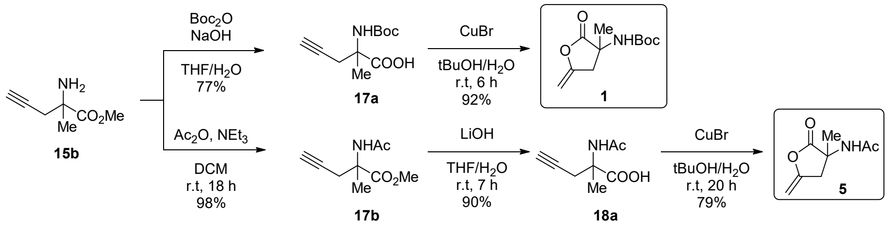

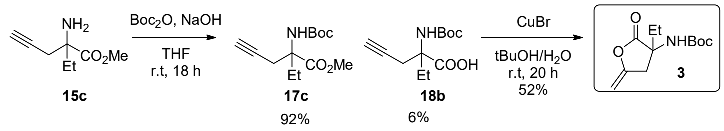

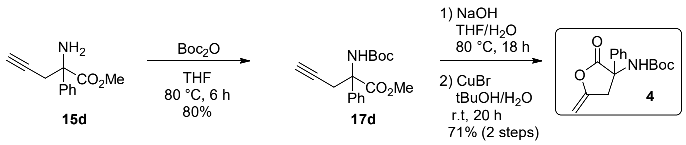

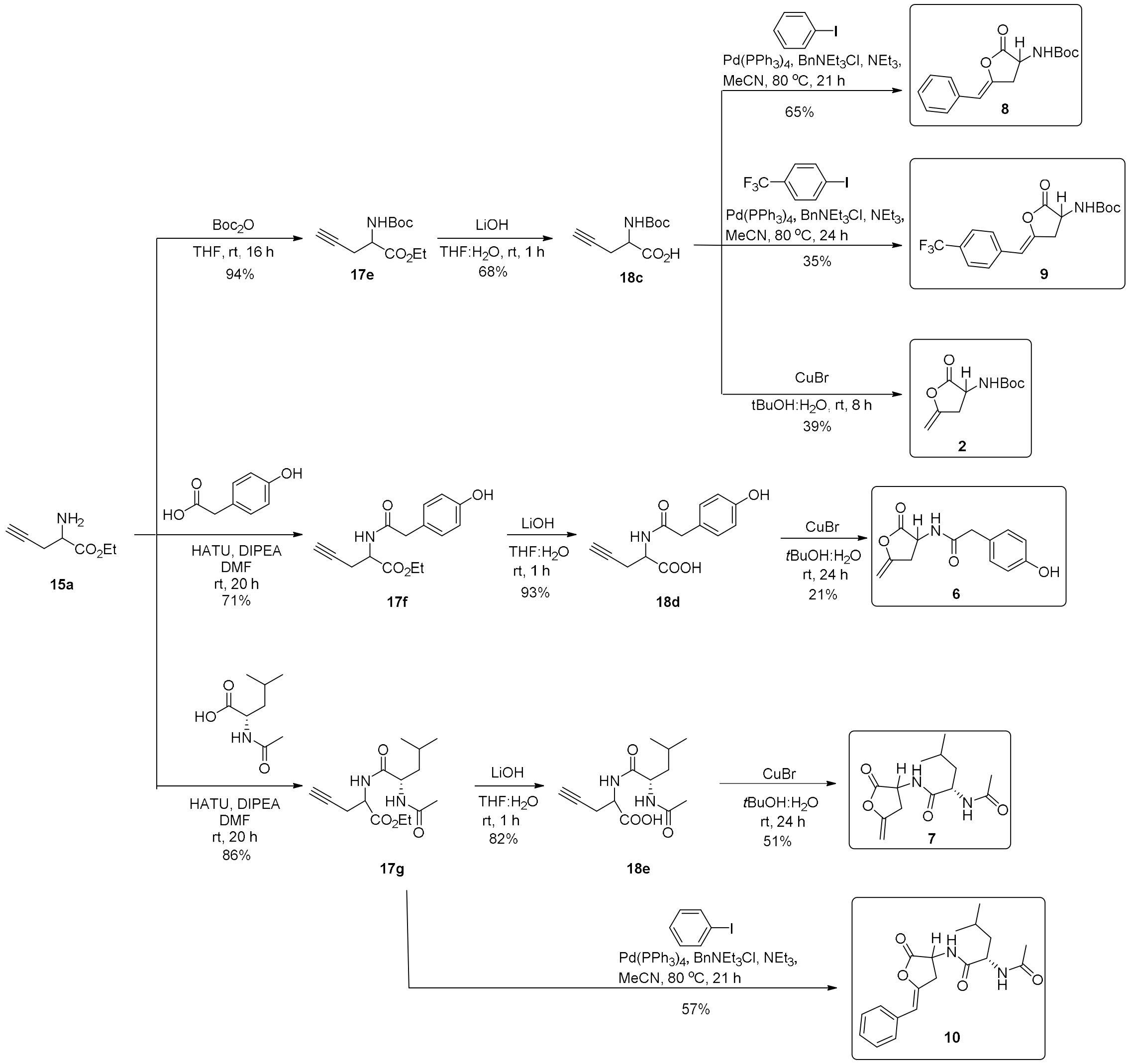
Pd(PPh3)4, BnNEt3Cl, NEt3,
MeCN, 80 ºC, 21 h
65%
NHBoc
8
F3C
Pd(PPh3)4, BnNEt3Cl, NEt3,
MeCN, 80 ºC, 24 h
35%
9
Boc2O
THF, rt, 16 h
94%
17e
LiOH
THF:H2O, rt, 1 h
68%
18c
CuBr
tBuOH:H2O, rt, 8 h
39%
2
NH2
CO2Et
15a
HATU, DIPEA
DMF
rt, 20 h
71%
17f
LiOH
THF:H2O
rt, 1 h
93%
18d
CuBr
tBuOH:H2O
rt, 24 h
21%
6
HATU, DIPEA
DMF
rt, 20 h
86%
17g
LiOH
THF:H2O
rt, 1 h
82%
18e
CuBr
tBuOH:H2O
rt, 24 h
51%
7
Pd(PPh3)4, BnNEt3Cl, NEt3,
MeCN, 80 ºC, 21 h
57%
10

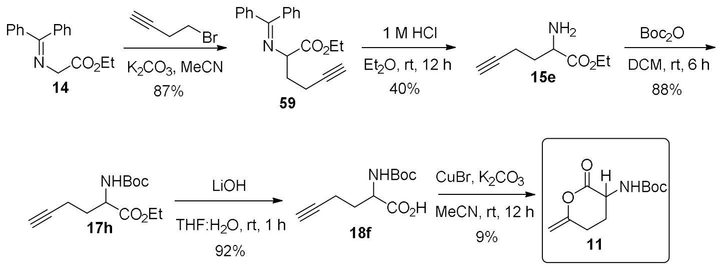
14
K2CO3, MeCN
87%
59
1 M HCl
Et2O, rt, 12 h
40%
15e
Boc2O
DCM, rt, 6 h
88%
17h
LiOH
THF:H2O, rt, 1 h
92%
18f
CuBr, K2CO3
MeCN, rt, 12 h
9%
11

3.3 Hit 2: CFI_{25}

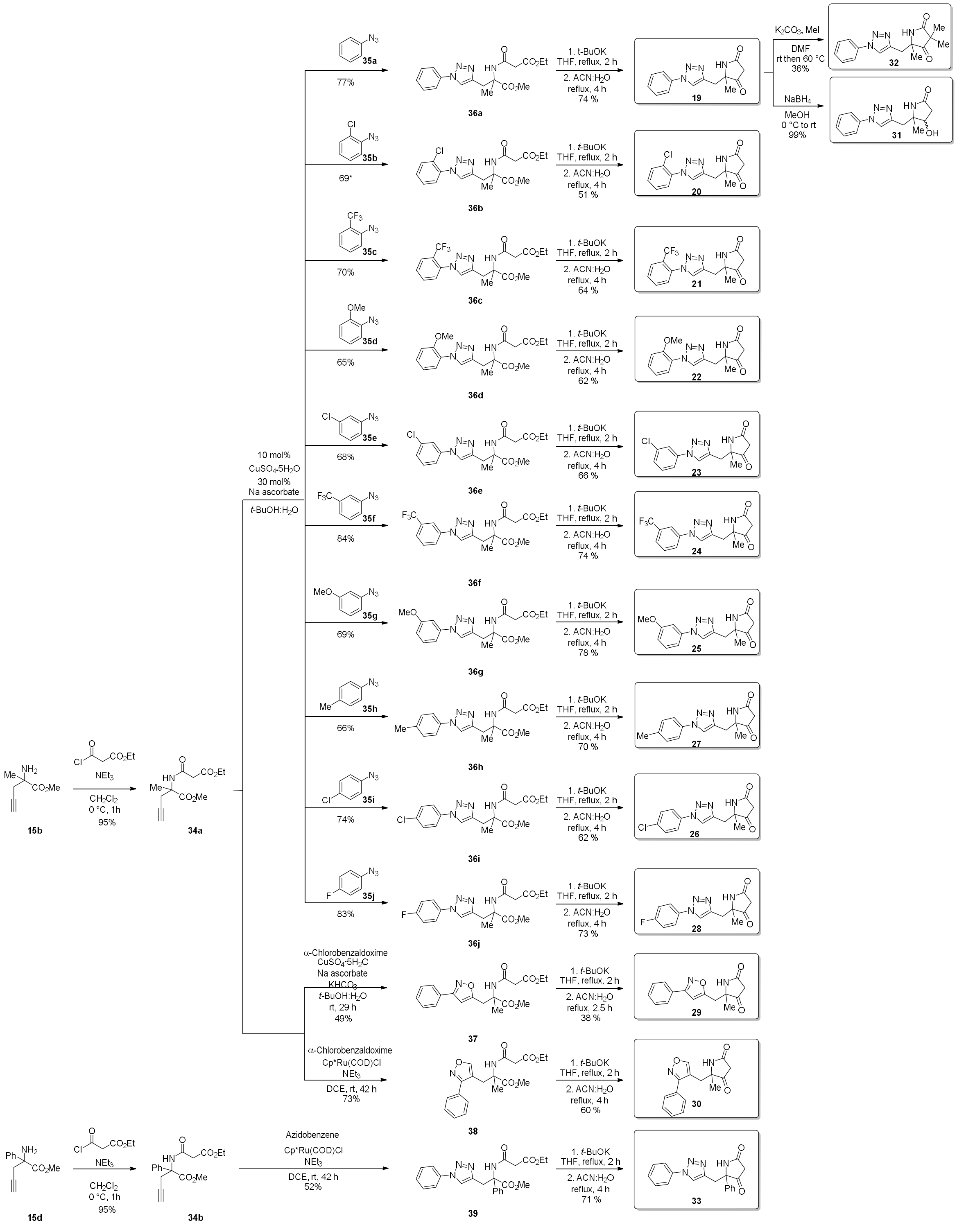

3.4 Hit 3: Activin A

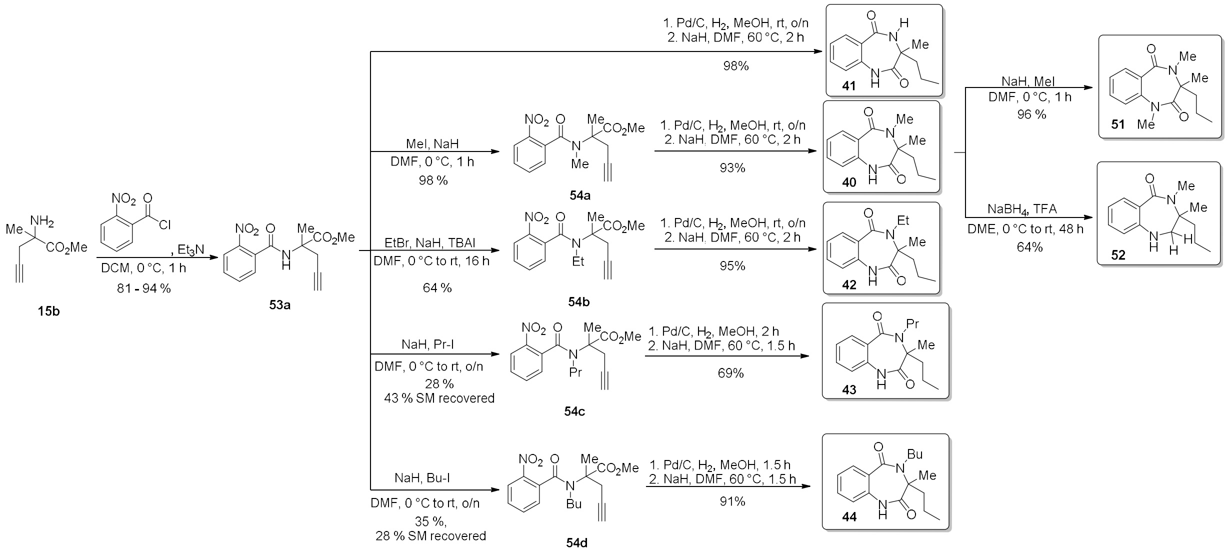

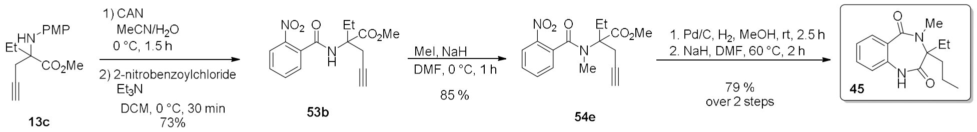

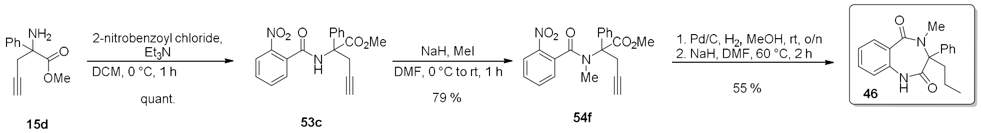

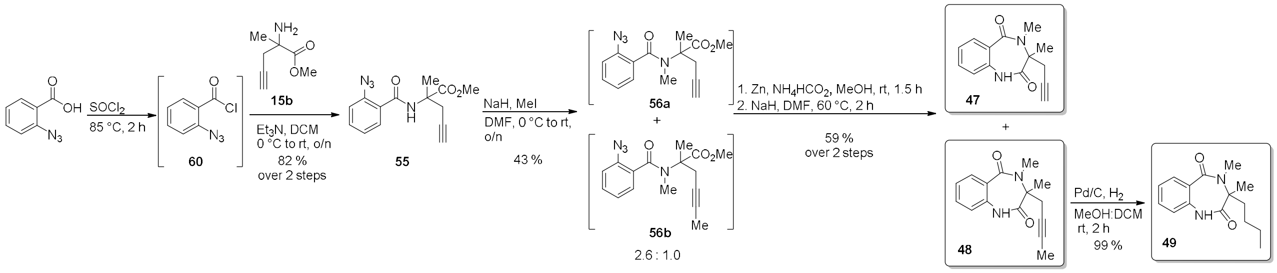

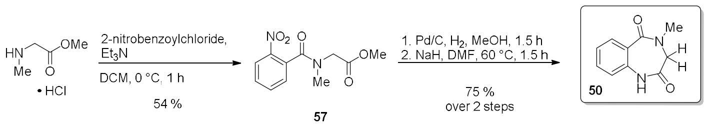

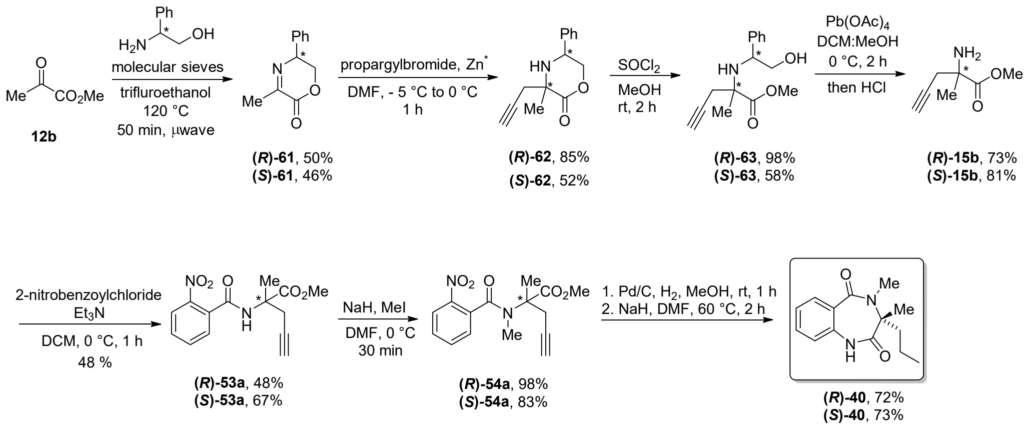

12b
molecular sieves
trifluroethanol
120 °C
50 min, μwave
(R)-61, 50%
(S)-61, 46%
propargylbromide, Zn*
DMF, - 5 °C to 0 °C
1 h
(R)-62, 85%
(S)-62, 52%
SOCl2
MeOH
rt, 2 h
(R)-63, 98%
(S)-63, 58%
Pb(OAc)4
DCM:MeOH
0 °C, 2 h
then HCl
(R)-15b, 73%
(S)-15b, 81%
2-nitrobenzoylchloride
Et3N
DCM, 0 °C, 1 h
48 %
(R)-53a, 48%
(S)-53a, 67%
NaH, MeI
DMF, 0 °C
30 min
(R)-54a, 98%
(S)-54a, 83%
1. Pd/C, H2, MeOH, rt, 1 h
2. NaH, DMF, 60 °C, 2 h
(R)-40, 72%
(S)-40, 73%

4 Supplementary Information: Synthetic Procedures

4.1 General Experimental Details

Proton nuclear magnetic resonance (^1H NMR) were recorded at ambient temperature on a Bruker DPX-400 (400 MHz), Bruker Advance 400 QNP (400 MHz) and Bruker Avance 500 Cryo Ultrashield (500 MHz). Tetramethylsilane was used as an internal standard. ^1H NMR chemical shifts (δ_H) are reported in parts per million (ppm), to the nearest 0.01 ppm and are referenced to the residual non-deuterated solvent peak ($CDCl_3$: 7.26, CD_3OD: 3.31, D_2O: 4.79). Coupling constants (J) are reported in Hertz (Hz) to the nearest 0.1 Hz. Data are reported as follows: chemical shift, integration, multiplicity (s = singlet; d = doublet; t = triplet; q = quartet; qn = quintet; sep = septet; m = multiplet; or as a combination of these), coupling constant(s) and assignment. Carbon NMR (^{13}C NMR) were recorded at ambient temperature on a Bruker DPX-400 (400 MHz), Bruker Advance 400 QNP (400 MHz,) and Bruker Avance 500 Cryo Ultrashield (500 MHz). Chemical shifts (δ_C) are quoted in ppm, to the nearest 0.1 ppm, and are referenced to the residual non-deuterated solvent peak ($CDCl_3$: 77.16, CD_3OD: 49.00).

^1H NMR and ^{13}C NMR spectra assignments were supported by DEPT-135, COSY (2D, ^1H-^1H correlations), HSQC (2D, one bond ^1H-^{13}C correlations), HMBC (2D, multi-bonds ^1H-^{13}C correlations) where appropriate. The numbering of molecules used for ^{13}C and ^1H NMR assignments does not conform to IUPAC standards. High resolution mass spectrometry (HRMS) measurements were recorded with a Micromass Q-TOF mass spectrometer or a Waters LCT Premier Time of Flight mass spectrometer using Electrospray ionisation (ESI) techniques. Mass values are reported within the ±5 ppm error limit.

Thin layer chromatography (TLC) was performed using pre-coated Merck glass backed silica gel 60 F_{254} plates and visualised by quenching of UV fluorescence (λ_{max} = 254 nm) or by staining with potassium permanganate. Retention factors (R_f) are quoted to 0.01. Flash column chromatography was carried out Merck 9385 Kieselgel 60 SiO_2 (230-400 mesh) under a positive pressure of nitrogen unless otherwise stated. Visualisation was achieved *via* ultraviolet light (254 nm) and chemical staining with $KMnO_4$ as appropriate.

Liquid chromatography mass spectroscopy (LCMS) was carried out using a Waters ACQUITY H-Class UPLC with an ESCi Multi-Mode Ionisation Waters SQ Detector 2 spectrometer using MassLynx 4.1 software; EI refers to the electrospray ionisation technique; LC system: solvent A: 2 mM NH_4OAc in H_2O/MeCN (95:5); solvent B: MeCN; solvent C: 2% formic acid; column: ACQUITY UPLC® CSH C18 (2.1 mm × 50 mm, 1.7 µm, 130 Å) at 40 °C; gradient: 5 – 95 % B with constant 5 % C over 1 minute at flow rate of 0.6 mL/minute; detector: PDA eλ Detector 220 – 800 nm, interval 1.2 nm. Melting points (m.p.) were obtained using a Büchi Melting Point B-545 melting point apparatus and are uncorrected. Infrared (IR) spectra were recorded neat on a

Perkin-Elmer Spectrum One spectrometer using an ATR sampling accessory either as solids or films in CH_2Cl_2. Selected absorptions (v_{max}) are reported in wavenumbers (cm^{-1}) with the following abbreviations: w, weak; m, medium; s, strong; br, broad.

Ethyl acetate, methanol, dichloromethane, acetonitrile and toluene were distilled from calcium hydride. Diethyl ether was distilled from a mixture of lithium aluminium hydride and calcium hydride. Tetrahydrofuran was dried using Na wire and distilled from a mixture of lithium aluminium hydride and calcium hydride with triphenylmethane as an indicator. Petroleum ether was distilled before use and refers to the fraction between 40-60 °C. All other solvents and reagents were obtained from commercial suppliers and used without further purification. Reactions were carried out under a stream of dry nitrogen using oven-dried glassware unless otherwise stated. Room temperature refers to ambient temperature. All temperatures below 0 °C were maintained using an acetone-cardice bath. Temperatures of 0 °C were maintained using an ice-water bath.

4.2 SI Hit 1: PBP3 Fragment Synthetic Procedures

Synthesis of methyl 2-((4-methoxyphenyl)amino)-2-methylpent-4-ynoate (13b)

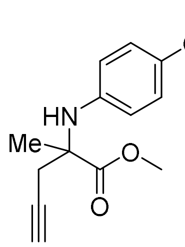

To a solution of *p*-anisidine (4.40 g, 35.7 mmol) in acetonitrile (40.0 mL) was added methyl pyruvate (4.70 mL, 52.0 mmol) and the reaction heated to 50 °C for 80 minutes. Upon completion, the reaction was concentrated *in vacuo* and the crude imine was redissolved in a mixture of petroleum ether:CH_2Cl_2 (10:1). The resulting precipitate was removed by filtration to afford an orange/brown oil. The crude imine (7.10 g, 34.3 mmol) was dissolved in DMF (172 mL) and cooled to 0 °C before propargyl bromide (4.87 mL, 45.2 mmol) was added, followed by activated Zn* powder (3.36 g, 51.7 mmol). The reaction was warmed to room temperature and then heated to 60 °C for 1 hour. Upon completion, the reaction was cooled to 0 °C and quenched with NH_4Cl saturated aqueous solution. The product was extracted with EtOAc (3 x 60 mL) and washed with brine (4 x 50 mL). The combined organic extracts were dried over Na_2SO_4, filtered and concentrated *in vacuo*. The crude product was purified by flash column chromatography (silica gel; petroleum ether/EtOAc, 4:1) to give the title product (4.16 g, 16.8 mmol, 47 %) as an orange/brown oil.

*Zn was stirred with 2 N HCl (aq) for 10-15 minutes and then washed with H_2O, EtOH, Et_2O and rigorously dried before use.

R_f = 0.43 (petroleum ether/EtOAc, 4:1); **^{1}H** NMR (400 MHz, $CDCl_3$) δ 6.77 – 6.70 (4H, m), 3.75 (3H, s), 3.74 (3H, s), 2.79 (1H, dd, *J* = 16.9, 2.7 Hz), 2.66 (1H, dd, *J* = 16.8, 2.7 Hz), 2.09 (1H, t, *J* = 2.7 Hz), 1.55 (3H, s); **^{13}C** (101 MHz, $CDCl_3$) δ 175.1, 154.7, 137.9, 121.4, 114.5, 79.7, 71.9, 61.3, 55.6, 52.7, 28.0, 24.0; **HRMS** (ESI) calcd for $[C_{14}H_{18}NO_3]^+$: 248.1287, found 248.1275; **IR** ν_{max} = 3283, 2952, 2120, 1727, 1509, 1108.

These characterisation data are in accordance with that previously reported in the literature.[14]

Synthesis of methyl 2-amino-2-methylpent-4-ynoate (15b)

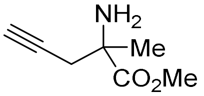

A solution of CAN (4.70 g, 8.10 mmol) in H_2O (10 mL) was added dropwise to a stirred solution of **13b** (500 mg, 2.00 mmol) in acetonitrile (10 mL) cooled to 0 °C. After stirring at 0 °C for 2 hours, the mixture was acidified to pH = 1 using 2 N HCl. The aqueous layer was washed with EtOAc (3 x 20 mL) and subsequently basified to pH = 11 using solid Na_2CO_3. The resulting emulsion was extracted with CH_2Cl_2 (3 x 50 mL), the combined organic layers washed with brine, dried over $MgSO_4$, filtered and concentrated *in vacuo* to afford the title compound (251 mg, 1.78 mmol, 89 %) as a brown oil. The product was directly subjected to the next step without further purification.

The spectroscopic data are in agreement with those previously reported in the literature.[14]

R_f = 0.21 (petroleum ether/EtOAc, 1:1); **^{1}H** NMR (400 MHz, $CDCl_3$) δ 3.72 (3H, s, 2.64 (1H, dd, *J* = 16.5, 2.6 Hz, 2.44 (1H, dd, *J* = 16.5, 2.6 Hz), 2.04 (1H, t, *J* = 2.6 Hz), 1.86 (2H, br s), 1.36 (3H, s); **^{13}C** NMR (101 MHz, $CDCl_3$) δ 176.6, 79.8, 71.5, 57.5, 52.6, 31.0, 26.0; **HRMS** (ESI) calcd for $[C_7H_{12}NO_2]^+$: 142.0863, found 142.0865; **IR** ν_{max} = 3293, 2956, 2114, 1730, 1598, 1208.

Synthesis of *tert*-butyl (3-methyl-5-methylene-2-oxotetrahydrofuran-3-yl)carbamate (1)

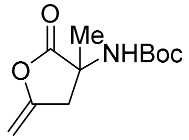

This compound was prepared as a member of our previously reported library.[14]

Synthesis of methyl 2-acetamido-2-methylpent-4-ynoate (17b)

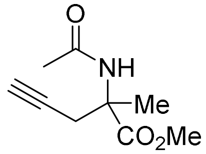

To a solution of **15b** (0.100 g, 0.71 mmol) in CH_2Cl_2 (2 mL) cooled to 0 °C, were added acetyl chloride (76 µL, 1.07 mmol) and triethylamine (198 µL, 1.42 mmol). The reaction was stirred at room temperature for 18 hours before being quenched with NH_4Cl (aq. Sat.), extracted into CH_2Cl_2 (3 x 20 mL), dried over Na_2SO_4, filtered and concentrated *in vacuo*. The crude product was purified by flash column chromatography (silica gel; petroleum ether/EtOAc, 2:3) to yield the title compound as a yellow oil (128 mg, 98%).

R_f = 0.21 (petroleum ether/EtOAc, 2:3); **^{1}H** NMR (400 MHz, $CDCl_3$) δ: 6.29 (1H, br s), 3.75 (3H, s), 3.04 – 2.90 (2H, m), 1.99 – 1.98 (4H, m), 1.57 (3H, s); **^{13}C** NMR (101 MHz, $CDCl_3$) δ: 173.4, 169.7, 79.6, 71.0, 58.5, 52.9, 26.2, 23.4, 22.8; **IR** ν_{max} = 3285, 3055, 2992, 2952, 1740, 1653, 1536, 1450, 1374, 1320, 1299, 1251, 1155, 1121.

The spectroscopic data are in agreement with those previously reported in the literature.[14]

Synthesis of 2-acetamido-2-methylpent-4-ynoic acid (18a)

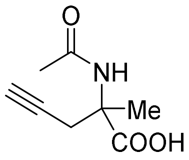

To a solution of **17b** (63 mg, 0.35 mmol) in a mixture of THF (2 mL) and water (1 mL) was added lithium hydroxide (37.3 mg, 8.88 mmol). The reaction was stirred at room temperature for 7 hours before being washed with EtOAc (10 mL), acidified to pH 2-3 with HCl (1 M), extracted into EtOAc (3 x 15 mL), dried over Na_2SO_4, filtered and concentrated *in vacuo* to yield the title compound as a white solid (53 mg, 90%).

^{1}H NMR (400 MHz, MeOD) δ: 3.13 (1H, dd, J = 16.9, 2.6 Hz), 2.69 (1H, dd, J = 16.9, 2.6 Hz), 2.33 (1H, t, J = 2.6 Hz), 1.95 (3H, s), 1.52 (3H, s); **^{13}C** NMR (101 MHz, MeOD) δ: 176.3, 172.8, 80.6, 72.1, 58.6, 26.6, 23.2, 22.4; **HRMS** (ESI-): [M-H]- calculated ($C_8H_{10}NO_3$): 168.0666; observed: 168.0665; **IR** ν_{max} = 3287, 2992, 2929, 1797, 1721, 1641, 1623, 1542, 1457, 1426, 1376, 1317, 1255, 1193, 1129, 1064.

Synthesis of *N*-(3-methyl-5-methylene-2-oxotetrahydrofuran-3-yl)acetamide (5)

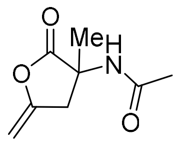

To a solution of **18a** (48 mg, 0.28 mmol) in a mixture of *t*BuOH (2 mL) and water (2 mL) was added copper (I) bromide (8 mg, 0.06 mmol). The reaction was stirred at room temperature for 20 hours before being diluted with water (5 mL), extracted into EtOAc (3x 15 mL), washed with brine (2 x 10 mL), dried over Na_2SO_4, filtered and concentrated *in vacuo.* The crude product was purified by flash column chromatography (silica gel; petroleum ether/EtOAc, 1:4) to yield the title compound as a white solid (38 mg, 79%).

R_f = 0.33 (petroleum ether/EtOAc, 1:4); **^{1}H** NMR (400 MHz, $CDCl_3$) δ: 6.04 (1H, br s), 4.80 (1H, s), 4.36 (1H, s), 3.36 (1H, d, *J* = 16.3 Hz), 2.82 (1H, d, *J* = 16.3 Hz), 2.01 (3H, s), 1.50 (3H, s); **^{13}C** NMR (101 MHz, $CDCl_3$) δ: 175.3, 169.8, 152.4, 90.0, 56.6, 38.7, 23.1, 22.8; **HRMS** (ESI+): [M+Na]+ calculated ($C_8H_{11}NO_3Na$): 192.0637; observed: 192.0631; **IR** ν_{max} = 3287, 1799, 1673, 1649, 1536, 1454, 1431, 1378, 1317, 1257, 1228, 1187, 1105, 1085.

Synthesis of methyl (*Z*)-2-((4-methoxyphenyl)imino)-2-phenylacetate (58)

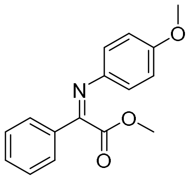

A solution of methyl benzyl formate (250 mg, 1.52 mmol), *p*-anisidine (197 mg, 1.60 mmol) and *p*-TsOH (14.4 mg, 0.0760 mmol) in toluene (3 mL) was heated to reflux with a dean-stark for 190 minutes. The reaction was cooled to room temperature and concentrated *in vacuo*. The crude product was purified by flash column chromatography (silica gel; petroleum ether/EtOAc, 3:1) to give the title compound (361 mg, 1.34 mmol, 88%) as a yellow solid.

R_f = 0.55 (petroleum ether/EtOAc, 3:1); **m.p.** 87.3 – 88.8 °C; **^{1}H** NMR (400 MHz, $CDCl_3$) δ 7.87 – 7.84 (2H, m), 7.53 – 7.43 (3H, m), 6.99 – 6.95 (2H, m), 6.90 – 6.87 (2H, m), 3.81 (3H, s), 3.70 (3H, s); **^{13}C** NMR (101 MHz, $CDCl_3$) δ 166.2, 159.3, 157.5, 143.3, 134.3, 131.7, 128.8, 127.9, 121.3, 114.3, 55.6, 52.1; **HRMS** (ESI+) calcd for $[C_{16}H_{16}NO_3]^+$: 270.1133, found 270.1130; **IR** ν_{max} = 2950, 1733, 1618, 1576, 1498, 1226.

These characterisation data are in accordance with that previously reported in the literature.[15]

Synthesis of methyl 2-((4-methoxyphenyl)amino)-2-phenylpent-4-ynoate (13d)

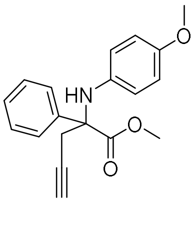

To an ice-cooled solution of **58** (620 mg, 2.30 mmol) and propargyl bromide (0.484 mL, 3.57 mmol) in DMF (13.6 mL) was added Zn* (301 mg, 4.60 mmol) and the reaction warmed to room temperature over 1 hour and then heated to 60 °C for 75 minutes. The reaction was quenched with NH_4Cl saturated aqueous solution and extracted with EtOAc (3 x 30 mL). The

combined organic extracts were washed with brine, dried over Na_2SO_4, filtered and concentrated *in vacuo*. The crude product was purified by flash column chromatography (silica gel; petroleum ether/EtOAc, 85:15) to give the title compound (457 mg, 1.47 mmol, 64%) as an orange oil.

R*f* = 0.48 (petroleum ether/EtOAc, 85:15); **^{1}H** NMR (400 MHz, $CDCl_3$) δ 7.58 – 7.55 (2H, m), 7.40 – 7.31 (3H, m), 6.65 – 6.61 (2H, m), 6.42 – 6.38 (2H, m), 4.97 (1H, br s), 3.72 (3H, s), 3.69 (3H, s), 3.39 (1H, dd, *J* = 16.4, 2.6 Hz), 3.21 (1H, dd, *J* = 16.4, 2.6 Hz), 2.02 (1H, t, *J* = 2.6 Hz); **^{13}C** NMR (101 MHz, $CDCl_3$) δ 173.1, 153.3, 139.8, 137.9, 128.9, 128.2, 127.0, 118.8, 114.5, 79.4, 71.9, 66.9, 55.6, 53.4, 25.0; **HRMS** (ESI+) calcd for $[C_{19}H_{20}NO_3]^+$: 310.1443, found 310.1430; **IR** ν_{max} = 3404, 2952, 2168, 1732, 1511, 1239.

The spectroscopic data are in agreement with those previously reported in the literature.[14]

Synthesis of methyl 2-amino-2-phenylpent-4-ynoate (15d)

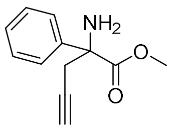

To an ice-cooled solution of **13d** (217 mg, 0.702 mmol) in acetonitrile (6 mL) was added a solution of CAN (1.53, 2.80 mmol) in H_2O (6.5 mL) dropwise. The mixture was allowed to warm to room temperature and then stirred for 23 hours. 2N HCl (aq) (6 mL) was added and the reaction stirred for 30 minutes. Upon completion, the reaction was extracted with EtOAc (3 x 20 m L) and the aqueous layer basified with Na_2CO_3 to pH 10. The resulting cream suspension was extracted with CH_2Cl_2 (3 x 30 m L) and the combined organic extracts were dried over Na_2SO_4, filtered and concentrated *in vacuo* to give the title compound (110 mg, 0.542 mmol, 77%) as a brown oil without further purification.

R*f* = 0.47 (petroleum ether/EtOAc, 3:2); **^{1}H** NMR (400 MHz, $CDCl_3$) δ 7.53 – 7.50 (2H, m), 7.38 – 7.33 (2H, m) 7.32 – 7.28 (1H, m), 3.75 (3H, s), 3.14 (1H, dd, *J* = 16.5, 3.2 Hz), 2.77 (1H, dd, *J* = 16.5, 2.5 Hz), 2.18 (2H, br s), 2.06 (1H, t, *J* = 2.6 Hz); **^{13}C** NMR (101 MHz, $CDCl_3$) δ 174.9, 141.5, 128.7, 128.1, 125.4, 80.0, 71.6, 63.5, 53.0, 31.1; **HRMS** (ESI+) calcd for $[C_{12}H_{14}NO_2]^+$: 204.1021, found 204.1025; **IR** ν_{max} = 3288, 2953, 1730, 1600.

The spectroscopic data are in agreement with those previously reported in the literature.[14]

Synthesis of methyl 2-((*tert*-butoxycarbonyl)amino)-2-phenylpent-4-ynoate (17d)

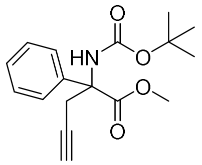

A solution of **15d** (91.0 mg, 0.448 mmol) and Boc_2O (146 mg, 0.672 mmol) in THF (4 mL) was heated to 75 °C for 18 hours in a sealed tube. Further Boc_2O (48.0 mg, 0.219 mmol) was added and the reaction stirred at 80 °C for a further 6 hours then cooled to room temperature and stirred for 18 hours. The reaction was concentrated *in vacuo* and purified by flash column chromatography (silica gel; petroleum ether/EtOAc, 85:15) to give the title compound (108 mg, 0.356 mmol, 80%) as a pale-yellow solid.

$\mathbf{R}_{f}$ = 0.32 (petroleum ether/EtOAc, 9:1); **m.p.** 100.3 – 101.9 °C; **^{1}H** NMR (400 MHz, $CDCl_3$) δ 7.44 (2H, d, *J* = 7.5 Hz), 7.38 – 7.34 (2H, m), 7.32 – 7.38 (1H, m), 6.03 (1H, br s), 3.71 (3H, s), 3.62 – 3.43 (2H, m), 1.99 (1H, m), 1.41 (9H, br s); **^{13}C** NMR (101 MHz, $CDCl_3$) δ 171.9, 154.1, 138.5, 128.8, 128.3, 126.0, 85.1, 79.6, 71.3, 64.5, 53.5, 28.4, 25.3; **HRMS** (ESI+) calcd for $[C_{17}H_{21}NO_4Na]^+$: 326.1368 , found 326.1365; **IR** ν_{max} = 3321, 2976, 2012, 1736, 1712, 1487, 1434.

Synthesis of *tert*-Butyl (5-methylene-2-oxo-3-phenyltetrahydrofuran-3-yl)carbamate (4)

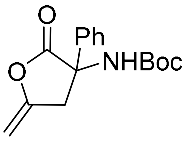

To a solution of **17d** (159 mg, 0.524 mmol) in a mixture of tetrahydrofuran (4 mL) and water (2 mL) was added 10% NaOH (2 mL). The reaction mixture was heated to 80 °C for 18 hours before being cooled and the THF removed *in vacuo*. The solution was then washed with EtOAc (3x 10 mL), acidifed to pH 3 with 1 M HCl, and the product extracted into EtOAc (3x 15 mL), dried over Na_2SO_4, filtered, and concentrated *in vacuo*. The crude residue was then dissolved in a mixture of *t*BuOH (2 mL) and water (2 mL), and copper (I) bromide (6 mg, 0.041 mmol) was added. The reaction was stirred at room temperature for 20 hours before being diluted with water (5 mL), extracted into EtOAc (3 x 15 mL), washed with brine (2 x 10 mL), dried over Na_2SO_4, filtered and concentrated *in vacuo.* The crude product was purified by flash column chromatography (silica gel; petroleum ether/EtOAc, 3:1) to yield the title compound as a white solid (42 mg, 71%).

R_f = 0.40 (petroleum ether/EtOAc, 3:1); **^{1}H** NMR (400 MHz, $CDCl_3$) δ: 7.50 – 7.47 (2H, m), 7.43 – 7.35 (3H, m), 5.37 (1H, br s), 4.82 – 4.80 (1H, m), 4.41 (1H, s), 3.77 (1H, d, *J* = 15.3 Hz), 3.42 (1H, d, *J* = 15.3 Hz), 1.44 (9H, s); **^{13}C** NMR (101 MHz, $CDCl_3$) δ: 173.4, 154.4, 152.3, 137.4, 129.3, 129.2, 125.4, 89.9, 81.1, 63.1, 39.2, 28.2; **HRMS** (ESI+): [M+Na]+ calculated ($C_{16}H_{19}NO_4Na$): 312.1206; observed: 312.1204; **IR** ν_{max} = 3362, 2977, 2924, 1809, 1701, 1677, 1485, 1450, 1367, 1290, 1251, 1224, 1159, 1128, 1026.

Synthesis of methyl 2-ethyl-2-((4-methoxyphenyl)amino)pent-4-ynoate (13c)

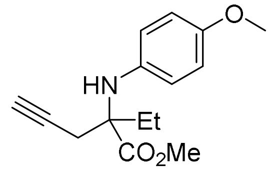

To a solution of *p*-anisidine (4.40 g, 35.7 mmol, 1.0 eq.) in acetonitrile (40 mL) was added ethylpyruvate (4.7 mL, 52.0 mmol, 1.5 eq.) and the mixture was stirred at 50 °C for 1 hour. The solvent was removed *in vacuo* and the crude product dissolved in petroleum ether/CH_2Cl_2 (9:1). The resulting precipitate was removed by filtration and the filtrate concentrated *in vacuo*. This titrulation/filtration procedure was applied four times. Subsequently, the resulting crude oil was dissolved in DMF (100 mL) and cooled to 0 °C. Propargyl bromide (80 % (w/w) in toluene, 4.6 mL, 42.8 mmol, 1.2 eq. of crude product) and activated zinc powder (3.50 g, 53.6 mmol, 1.5 eq. of crude product) were added and the reaction stirred at 0 °C for 30 minutes. The reaction was warmed to room temperature and heated to 50 °C for additional 50 minutes. Subsequently, the mixture was cooled to 0 °C and quenched with sat. aq. solution of NH_4Cl (20 mL). After extraction with EtOAc (3 x 50 mL), the combined organic layers were washed with brine, dried over $MgSO_4$, filtered and concentrated *in vacuo*. The crude product was purified by flash column chromatography (silica gel, petroleum ether/EtOAc 9:1) to afford the title compound (4.93 g, 18.9 mmol, 53 %) as a yellow oil.

R_f = 0.42 (petroleum ether/EtOAc, 4:1); **^{1}H** NMR (400 MHz, $CDCl_3$) δ: 6.77 – 6.73 (2H, m), 6.72 – 6.68 (2H, m), 3.76 (3H, s), 3.74 (3H, s), 2.82 – 2.68 (2H, m), 2.02 (1H, t, *J* = 2.7 Hz), 2.00 – 1.92 (2H, m), 0.91 (3H, t, *J* = 7.4 Hz); **^{13}C** NMR (101 MHz, $CDCl_3$) δ: 174.4, 154.2, 137.8, 120.5, 114.5, 79.8, 71.2, 65.1, 55.5, 52.5, 30.0, 24.0, 8.4; **HRMS** (ESI+): [M+H]+ calculated ($C_{15}H_{20}NO_3$): 262.1438; observed: 262.1429; **IR** ν_{max} = 3289, 2952, 1731, 1510, 1464, 1437, 1241, 1178, 1127, 1035.

Synthesis of methyl 2-amino-2-ethylpent-4-ynoate (15c)

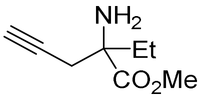

To a solution of **13c** (1.39 g, 5.3 mmol) in acetonitrile (33 mL), cooled to 0 °C was dropwise added a solution of cerium (IV) ammonium nitrate (5.83 g, 10.6 mmol) in water (33 mL). The reaction was stirred at room temperature for 2 hours before being acidified to pH 1 with HCl (3 M). The mixture was then washed with EtOAc (3x 50 mL), and the aqueous was treated with Na_2CO_3 (sat. aq.) until basic then extracted with CH_2Cl_2 (3 x 100 mL). The combined organics were dried over Na_2SO_4, filtered and concentrated *in vacuo* to give the title compound as a yellow oil (1.057 g, 64%).

^{1}**H** NMR (400 MHz, $CDCl_3$) δ: 3.73 (3H, s), 2.66 (1H, dd, *J* = 16.5, 2.7 Hz), 2.43 (1H, dd, *J* = 16.5, 2.7 Hz), 2.03 (1H, t, *J* = 2.7 Hz), 1.88 (2H, br s), 1.82 – 1.73 (1H, m), 1.67 – 1.58 (1H, m), 0.85 (3H, t, *J* = 7.5 Hz); ^{13}**C** NMR (101 MHz, $CDCl_3$) δ: 176.0, 79.7, 71.3, 61.1, 52.4, 32.3, 29.5, 8.4; **HRMS** (ESI+): [M+H]+ calculated ($C_8H_{14}NO_2$): 156.1019; observed: 156.1017; **IR** ν_{max} = 3287, 2970, 2880, 1731, 1599, 1458, 1435, 1236, 1204, 1160, 1122, 1003.

Synthesis of methyl 2-((tert-butoxycarbonyl)amino)-2-ethylpent-4-ynoate (17c) and 2-((tert-butoxycarbonyl)amino)-2-ethylpent-4-ynoic acid (18b)

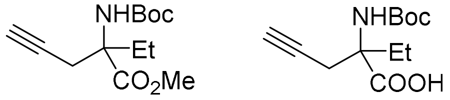

17c **18b**

To a solution of **15c** (100 mg, 0.64 mmol) in a mixture of tetrahydrofuran (6 mL) and water (3 mL) were added di-tert-butyl dicarbonate (0.44 mL, 1.93 mmol) and 10% NaOH (1 mL). The reaction at room temperature for 18 h before the THF was removed *in vacuo*. The solution was then washed with EtOAc (3x 10 mL), acidified to pH 3 with 1 M HCl, and the product extracted into EtOAc (3x 15 mL), dried over Na_2SO_4, filtered, and concentrated *in vacuo* to give **18b** as a yellow oil (10 mg, 6%). The first organic wash was then dried over Na_2SO_4, filtered, and concentrated *in vacuo* to give **17c** as a yellow oil (150 mg, 92%).

Data for 18b:

$\mathbf{R_f}$ = 0.65 (petroleum ether/EtOAc, 4:1); ^{1}**H** NMR (400 MHz, $CDCl_3$) δ: 5.45 (1H, br s), 3.77 (3H, s), 3.13 (1H, d, *J* = 16.7 Hz), 2.82 (1H, dd, *J* = 16.7, 2.4 Hz), 2.15 (1H, br s), 1.96 (1H, t, *J* = 2.4 Hz), 1.86 – 1.77 (1H, m), 1.44 (9H, s); ^{13}**C** NMR (101 MHz, $CDCl_3$) δ: 172.8, 154.1,

79.6, 70.5, 62.9, 53.4, 52.7, 28.4, 28.3, 25.1, 8.3; **HRMS** (ESI+): [M+Na]+ calculated ($C_{13}H_{20}NO_4Na$): 278.1363; observed: 278.1362; **IR** ν_{max} = 3294, 2976, 2876, 1741, 1710, 1493, 1448, 1433, 1392, 1366, 1351, 1331, 1284, 1247, 1223, 1163, 1132, 1070, 1010.

Data for 17c:

R*f* = 0.10 (8:2 EtOAc/PE); **^{1}H** NMR (400 MHz, $CDCl_3$) δ: 10.9 (1H, br s), 5.41 (1H, br s), 3.11 (1H, d, *J* = 15.2 Hz), 2.88 (1H, d, *J* = 15.2 Hz), 2.24 – 2.10 (1H, m), 1.98 (1H, t, *J* = 2.4 Hz), 1.94 – 1.83 (1H, m), 1.44 (9H, s), 0.88 (3H, t, *J* = 7.2 Hz); **^{13}C** NMR (101 MHz, $CDCl_3$) δ: 177.4, 154.4, 80.1, 79.3, 70.8, 62.6, 28.3, 24.9, 20.8, 8.2; **HRMS** (ESI-): [M-H]- calculated ($C_{12}H_{18}NO_4$): 240.1241; observed: 240.1240; **IR** ν_{max} = 3299, 2978, 2936, 1711, 1655, 1499, 1456, 1396, 1369, 1254, 1164, 1073.

Synthesis of t*ert*-butyl (3-ethyl-5-methylene-2-oxotetrahydrofuran-3-yl)carbamate (3)

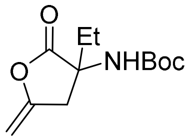

To a solution of **18b** (25 mg, 0.104 mmol) in a mixture of *t*BuOH (1 mL) and water (1 mL) was added copper (I) bromide (3 mg, 0.021 mmol). The reaction was stirred at room temperature for 20 h before being diluted with water (5 mL), extracted into EtOAc (3x 15 mL), washed with brine (2x 10 mL), dried over Na_2SO_4, filtered and concentrated *in vacuo.* The crude product was purified by flash column chromatography (silica gel; petroleum ether/EtOAc, 1:1 to 0:1) to yield the title compound as a white solid (13 mg, 52%).

R*f* = 0.35 (9:1 EtOAc/PE); **^{1}H** NMR (400 MHz, $CDCl_3$) δ: 5.30 (1H, s), 4.78 – 4.76 (1H, m), 4.34 – 4.33 (1H, m), 3.36 (1H, br d, *J* = 14.8 Hz), 2.87 (1H, d, *J* = 16.3 Hz), 1.89 – 1.72 (2H, m), 1.44 (9H, s), 1.00 (3H, t, *J* = 7.7 Hz); **^{13}C** NMR (101 MHz, $CDCl_3$) δ: 175.1, 154.5, 152.6, 89.4, 60.2, 53.4, 36.8, 29.8, 28.2, 7.6; **HRMS** (ESI+): [M+Na]+ calculated ($C_{12}H_{19}NO_4Na$): 264.1206; observed: 264.1206; **IR** ν_{max} = 3362, 2977, 2930, 1806, 1700, 1670, 1508, 1458, 1392, 1366, 1280, 1251, 1161, 1094.

Synthesis of ethyl 2-((4-methoxyphenyl)amino)pent-4-ynoate (13a)

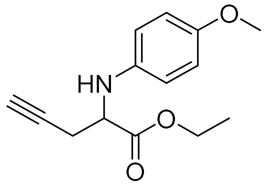

To a solution of ethyl glyoxalate (50% wt. % in toluene, 5.0 mL, 24.5 mmol) in toluene (50 mL) were added *p*-anisidine (2.01 g, 16.3 mmol) and $MgSO_4$ (4 g) and the reaction was stirred at r.t for 30 minutes. The mixture was filtered, and the filtrate concentrated *in vacuo*, resulting in a brown oil, which was triturated with 9:1 petroleum ether/DCM before concentrating *in vacuo*. The resulting yellow oil was dissolved in DMF (80 mL) and cooled to -10 ˚C. Propargyl bromide (80% wt. % in toluene, 3.5 mL, 24.5 mmol) and activated zinc powder (1.6 g, 65.4 mmol) were added and the reaction mixture was warmed to room temperature and then heated to 60 ˚C. After 1.5 hours, the mixture was cooled to 0 °C, quenched with a saturated aqueous solution of NH_4Cl and extracted with ethyl acetate (3 x 20 mL). The combined organic layers were washed with brine (3 x 10 mL), dried with $MgSO_4$, filtered, and concentrated *in vacuo*. The residue was purified by flash column chromatography (silica gel; petroleum ether/EtOAc, 9:1) to afford the title compound as a yellow oil (1.86 g, 46%).

R_f = 0.55 (petroleum ether/EtOAc, 4:1). **^{1}H** NMR (400 MHz, $CDCl_3$): δ_H 6.77 (2H, d, *J* = 9.0 Hz), 6.63 (2H, d, *J* = 9.0 Hz), 4.14-4.33 (3H, m), 3.73 (3H, s), 2.74 (2H, dd, *J* = 5.3, 2.7 Hz), 2.07 (1H, t, *J* = 2.7 Hz), 1.26 (3H, t, *J* = 7.2 Hz); **^{13}C** NMR (101 MHz, $CDCl_3$): δ_C 172.1, 153.1, 140.1, 115.7, 114.9, 77.2, 71.6, 68.7, 61.5, 55.7, 22.9, 14.2; **HRMS** (ESI+): $[M+H]^+$ calculated ($C_{14}H_{18}NO_3$): 248.1281; observed: 248.1282; **IR** ν_{max} = 3364, 3285, 2982, 2833, 2120, 1731, 1511, 1464, 1443, 1236, 1181, 1138, 1033.

These characterisation data are in accordance with that previously reported in the literature.[16]

Synthesis of ethyl 2-aminopent-4-ynoate (15a)

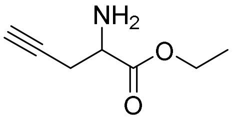

To a solution of **13a** (1.18 g, 5.1 mmol) in acetonitrile (30 mL) at 0 ˚C was dropwise added a solution of CAN (5.54 g, 10.1 mmol) in water (30 mL) over 15 minutes. Following 2 hours of stirring at room temperature, 2 M HCl was added until pH 1 was achieved, the aqueous phase

was washed with ethyl acetate (3x10 mL) and basified by the addition of Na_2CO_3. The resulting suspension was extracted with DCM (3x10 mL) and the combined organic extracts were dried over Na_2SO_4, filtered and concentrated *in vacuo* to afford the title compound as a pale yellow oil (0.337 g, 52%) with no need for further purification.

R_f = 0.26 (petroleum ether/EtOAc, 1:4). **^{1}H** NMR (400 MHz, $CDCl_3$): δ_H 4.14 (2H, m), 3.56 (1H, t, *J* = 5.7 Hz), 2.58 (2H, m), 2.01 (1H, t, *J* = 2.3 Hz), 1.77 (2H, s br), 1.23 (3H, t, *J* = 6.9 Hz); **^{13}C** NMR (100 MHz, $CDCl_3$): δ_C 173.7, 79.5, 71.2, 61.3, 53.1, 24.8, 14.2; **HRMS** (ESI+): $[M+H]^+$ calculated ($C_7H_{12}NO_2$): 142.0863; observed: 142.0859; **IR** ν_{max} = 3366, 3289, 2981, 2120, 1730, 1426, 1374, 1194, 1026.

These characterisation data are in accordance with that previously reported in the literature.[17]

Synthesis of ethyl 2-((tert-butoxycarbonyl)amino)pent-4-ynoate (17e)

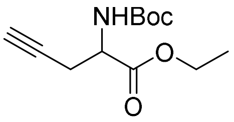

To a solution of **15a** (353 mg, 2.5 mmol) in DCM (6 mL) was added Boc_2O (546 mg, 2.5 mmol) and the reaction was stirred for 12 hours at room temperature. The reaction mixture was concentrated under reduced pressure and the crude product purified by flash column chromatography (silica gel; petroleum ether/EtOAc, 9:1) to yield the title compound as a colourless oil (569 mg, 2.36 mmol, 94%).

R_f = 0.63 (petroleum ether/EtOAc, 4:1). **^{1}H** NMR (400 MHz, $CDCl_3$): δ_H 4.42 (1H, m), 4.21 (2H, m), 2.71 (2H, m), 2.00 (1H, s), 1.42 (9H, s), 1.26 (3H, t, *J* = 7.1 Hz); **^{13}C** NMR (100 MHz, $CDCl_3$): δ_C 170.6, 155.1, 80.1, 78.6, 71.5, 61.8, 51.9, 28.3, 22.9, 14.2; **HRMS** (ESI+): $[M+H]^+$ calculated ($C_{12}H_{20}NO_4$): 242.1387; observed: 242.1394; **IR** ν_{max} = 3292, 2980, 2941, 2124, 1741, 1712, 1500, 1368, 1343, 1249, 1159, 1117, 1062, 1026.

These characterisation data are in accordance with that previously reported in the literature.[17]

Synthesis of 2-((*tert*-butoxycarbonyl)amino)pent-4-ynoic acid (18c)

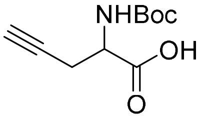

To a solution of **17e** (375 mg, 1.55 mmol) in THF (16 mL) and water (8 mL) was added LiOH monohydrate (169 mg, 4.02 mmol). After 1 hour stirring, the solution was partially concentrated under reduced pressure, acidified to pH 1 with 3 M HCl at 0 ˚C and extracted with ethyl acetate (3x 10 mL). The combined organic layers were dried over Na_2SO_4, filtered and concentrated *in vacuo* to afford the title compound as a colourless oil (228 mg, 1.05 mmol, 68%) with no need for further purification.

R*f* = 0.10 (petroleum ether/EtOAc, 4:1). **^{1}H** NMR (400 MHz, $CDCl_3$): δ_H 4.50 (1H, m), 2.77 (2H, m), 2.07 (1H, m), 1.45 (9H, s); **^{13}C** NMR (100 MHz, $CDCl_3$): δ_C 174.9, 155.4, 80.7, 78.3, 71.9, 60.5, 28.3, 22.5; **HRMS** (ESI+): $[M+H]^+$ calculated ($C_{10}H_{16}NO_4$): 214.1074; observed: 214.1083; **IR** ν_{max} = 3297, 2980, 2924, 2129, 1712, 1709, 1505, 1393, 1368, 1157, 1060, 1026.

These characterisation data are in accordance with that previously reported in the literature.[17]

Synthesis of t*ert*-butyl (*Z*)-(5-benzylidene-2-oxotetrahydrofuran-3-yl)carbamate (8)

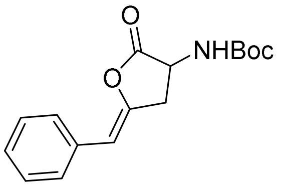

A solution of **18c** (10.8 mg, 0.0497 mmol), iodobenzene (5.6 µL, 0.0497 mmol) and $BnNEt_3Cl$ (11.3 mg, 0.0497 mmol) in acetonitrile (2 mL) and Et_3N (0.4 mL) was degassed with a stream of nitrogen for 30 minutes followed by 3 freeze-pump-thaw cycles. The reaction was frozen and $Pd(PPh_3)_4$ (5.7 mg, 0.0049 mmol) was added, the reaction was thawed at room temperature and then heated to reflux for 21 hours. The reaction mixture was concentrated under reduced pressure before being resuspended in DCM (10 mL), washed with water (3x 10 mL), dried over $MgSO_4$, filtered and solvent removed *in vacuo*. The crude product was purified by flash column chromatography (silica gel; petroleum ether/EtOAc, gradient 9:1 to 4:1) to yield the title compound as a thick, yellow oil (9.5 mg, 0.032 mmol, 65%).

R*f* = 0.41 (petroleum ether/EtOAc, 4:1). **^{1}H** NMR (400 MHz, $CDCl_3$): δ_H 7.49 (2H, m), 7.32 (2H, m), 7.21 (1H, m), 5.13 (1H, s br), 4.43 (1H, m), 3.64 (1H, m), 3.32 (1H, m), 1.45 (9H, s); **^{13}C** NMR (100 MHz, $CDCl_3$): δ_C 173.6, 156.9, 147.9, 134.1, 129.1, 128.1, 127.2, 108.8, 81.2, 66.0 (diethyl ether), 61.4, 30.0, 28.3, 15.5 (diethyl ether); **HRMS** (ESI+): $[M+H]^+$ calculated ($C_{16}H_{20}NO_4$): 290.1387; observed: 290.1382; **IR** ν_{max} = 3291, 2979, 2918, 1808, 1712, 1499, 1473, 1368, 1344, 1249, 1159, 1117, 1062, 1027.

Synthesis of t*ert*-butyl (*Z*)-(2-oxo-5-(4-(trifluoromethyl)benzylidene)tetrahydrofuran-3-yl)carbamate (9)

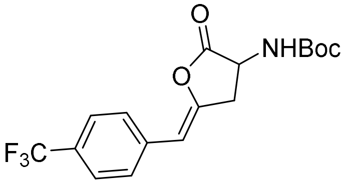

A solution of **18c** (27.2 mg, 0.125 mmol), 4-iodobenzotrifluoride (15.6 µL, 0.125 mmol) and $BnNEt_3Cl$ (28.6 mg, 0.125 mmol) in acetonitrile (2 mL) and Et_3N (0.4 mL) was degassed with a stream of nitrogen for 30 minutes followed by 3 freeze-pump-thaw cycles. The reaction was frozen and $Pd(PPh_3)_4$ (14 mg, 0.0125 mmol) was added, the reaction was thawed at room temperature and then heated to reflux for 24 hours. The reaction mixture was concentrated under reduced pressure before being resuspended in DCM (10 mL), washed with water (3x10 mL), dried over $MgSO_4$, filtered and solvent removed *in vacuo*. The crude product was purified by flash column chromatography (silica gel; petroleum ether/EtOAc, gradient 9:1 to 4:1) to yield the title compound as a yellow oil (15.8 mg, 0.0438 mmol, 35%).

R_f = 0.31 (petroleum ether/EtOAc, 4:1). **^{1}H** NMR (400 MHz, $CDCl_3$): δ_H 7.56 (2H, d, *J* = 7.6 Hz), 7.34 (2H, d, *J* = 7.6 Hz), 5.24 (1H, m), 5.01 (1H, m), 3.07 (2H, d, *J* = 7.4 Hz), 1.47 (9H, s); **^{13}C** NMR (100 MHz, $CDCl_3$): δ_C 174.3, 157.9, 145.2, 137.2, 130.2, 128.3, 127.2, 125.7, 119.3, 83.6, 66.0 (ether), 62.9, 30.0, 28.3, 15.5 (diethyl ether); **HRMS** (ESI+): $[M+H]^+$ calculated ($C_{17}H_{19}F_3NO_4$): 358.1261; observed: 358.1252; **IR** ν_{max} = 3327, 3006, 2973, 1810, 1713, 1508, 1488, 1467, 1102, 1066.

Synthesis of *tert*-Butyl (5-methylene-2-oxotetrahydrofuran-3-yl)carbamate (2)

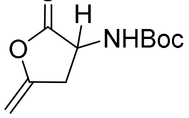

To a solution of **18c** (155 mg, 0.73 mmol) in a mixture of *t*BuOH (3 mL) and water (3 mL) was added copper (I) bromide (21 mg, 0.145 mmol). The reaction was stirred at room temperature for 20 h before being diluted with water (5 mL), extracted into EtOAc (3 x 15 mL), washed with brine (2x 10 mL), dried over Na_2SO_4, filtered and concentrated *in vacuo.* The crude product was purified by flash column chromatography (silica gel; petroleum ether/EtOAc, 9:1) to yield the title compound as a colourless oil (61 mg, 39%).

R$_f$ = 0.49 (petroleum ether/EtOAc, 8:2); **^{1}H** NMR (400 MHz, $CDCl_3$) δ: 5.09 (1H, br s), 4.80 (1H, t, *J* = 2.6 Hz), 4.50 – 4.40 (2H, m), 3.30 – 3.24 (1H, m), 2.89 – 2.83 (1H, m), 1.45 (9H, s); **^{13}C** NMR (101 MHz, $CDCl_3$) δ: 173.0, 155.2, 152.2, 90.5, 81.0, 50.5, 33.6, 28.2; **IR** ν_{max} = 3357, 2972, 2929, 1814, 1685, 1503, 1453, 1368, 1290, 1250, 1140, 1032.

Synthesis of ethyl 2-(2-(4-hydroxyphenyl)acetamido)pent-4-ynoate (17f)

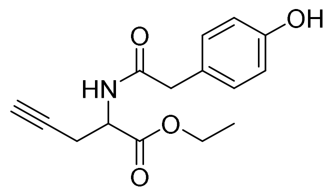

To a stirred solution of 4-hydroxyphenyl acetic acid (165 mg, 1.09 mmol), HATU (516 mg, 1.36 mmol) and DIPEA (0.24 mL, 1. 36 mmol) in DMF (2 mL) at 0 ˚C was added a solution of **15a** (160 mg, 1.13 mmol) in DMF (1 mL). Following 20 hours stirring at room temperature, the product was extracted from water (20 mL) with ethyl acetate (3 x 10 mL) and the combined organic extracts were washed with brine (3 x 10 mL), dried over $MgSO_4$, filtered and concentrated *in vacuo*. The crude product was purified by flash column chromatography (silica gel; petroleum ether/EtOAc, 1:1) to yield the title compound as a yellow oil (212 mg, 0.77 mmol, 71%).

R$_f$ = 0.30 (petroleum ether/EtOAc, 1:1); **^{1}H** NMR (400 MHz, $CDCl_3$): δ_H 7.09 (2H, d, *J* = 7.9 Hz), 6.75 (2H, d, *J* = 7.9 Hz), 6.40 (2H, m), 4.69 (1H, m), 4.19 (2H, m), 3.54 (2H, s), 2.73 (2H, m), 1.95 (1H, t, *J* = 2.6 Hz), 1.26 (3H, t, *J* = 7.3 Hz, H15 and trace ethyl acetate); **^{13}C** NMR (100 MHz, $CDCl_3$): δ_C 171.8, 170.3, 155.6, 130.7, 125.6, 115.9, 78.1, 71.7, 62.1, 50.6, 42.6, 22.3, 14.1; **HRMS** (ESI+): $[M+H]^+$ calculated ($C_{15}H_{18}NO_4$): 276.1230; observed: 276.1232; **IR** ν_{max} = 3506, 3298, 2920, 2147, 1735, 1653, 1515, 1447, 1375, 1218, 1015.

Synthesis of 2-(2-(4-hydroxyphenyl)acetamido)pent-4-ynoic acid (18d)

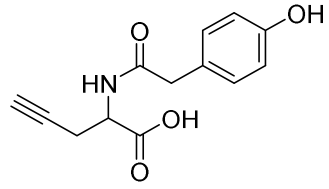

To a solution of **17f** (71 mg, 0.26 mmol) in THF (3 mL) and water (1.5 mL) was added LiOH monohydrate (27 mg, 0.65 mmol). After 1 hour stirring, the organics were removed *in vacuo*

and the solution was acidified to pH 1 with 3 M HCl at 0 ˚C and extracted with ethyl acetate (3 x 10 mL). The combined organic layers were dried over Na_2SO_4, filtered and concentrated *in vacuo* to afford the title compound as a thick, pale yellow oil (60 mg, 0.24 mmol, 93%) with no need for further purification.

^{1}H NMR (400 MHz, DMSO-d$_6$): δ_H 12.76 (1H, s br), 9.21 (1H, s br), 8.33 (1H, s br), 7.05 (2H, d, *J* = 8.8 Hz), 6.66 (2H, d, *J* = 8.8 Hz), 4.34 (1H, d, *J* = 7.3, 13.4 Hz), 3.37 (2H, s), 2.88 (1H, t, *J* = 2.6 Hz), 2.58 (2H, m); **^{13}C** NMR (100 MHz, DMSO-d$_6$): δ_C 206.6 (acetone), 171.9, 170.8, 155.9, 130.1, 126.3, 115.0, 80.4, 73.2, 51.0, 48.7 (MeOH), 41.1, 30.8 (acetone), 21.3; **HRMS** (ESI+): [M+H]$^+$ calculated ($C_{13}H_{14}NO_4$): 248.0917; observed: 248.0916; **IR** ν_{max} = 3587, 3308, 2890, 2176, 1722, 1653, 1512, 1510, 1446.

Synthesis of 2-(4-hydroxyphenyl)-*N*-(5-methylene-2-oxotetrahydrofuran-3-yl)acetamide (6)

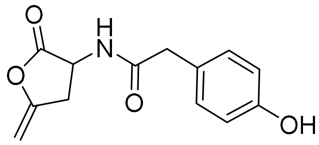

To a solution of **18d** (89 mg, 0.36 mmol) in water (3 mL) and ^{t}BuOH (3 mL) was added CuBr (13 mg, 0.09 mmol) and the reaction stirred for 24 hours at room temperature. The reaction was diluted with water (20 mL) and extracted with ethyl acetate (3x10 mL). The combined organic layers were washed with brine (2 x 10 mL), dried with Na_2SO_4, filtered and concentrated under reduced pressure. The crude product was purified by flash column chromatography (silica gel; petroleum ether/EtOAc, 1:1) to yield the title compound as a thick, colourless oil (18.7 mg, 0.076 mmol, 21%).

R_f = 0.28 (PE 40-60/ethyl acetate, 2:3).**^{1}H** NMR (400 MHz, acetone-d$_6$): δ_H 8.29 (1H, s br), 7.12 (2H, d, *J* = 8.7 Hz), 6.77 (2H, d, *J* = 8.7 Hz), 4.65 (1H, m), 4.61 (1H, dd, *J* = 2.3, 3.7 Hz), 4.34 (1H, dd, *J* = 2.3, 3.7 Hz), 3.45 (2H, s), 3.15 (1H, m), 2.93 (1H, m); **^{13}C** NMR (101 MHz, acetone-d$_6$): δ_C 172.7, 171.0, 156.3, 154.0, 130.4, 126.1, 115.3, 87.4, 49.0, 41.4, 31.6; **HRMS** (ESI+): [M+H]$^+$ calculated ($C_{13}H_{14}NO_4$): 248.0917; observed: 248.0918; **IR** ν_{max} = 3386, 1760, 1654, 1653, 1510, 1446, 1048, 1023.

Synthesis of ethyl 2-((*S*)-2-acetamido-4-methylpentanamido)pent-4-ynoate (17g)

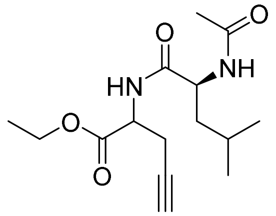

To a solution of *N*-acetyl-*L*-leucine (419 mg, 2.42 mmol), HATU (1.16 g, 3.04 mmol) and DIPEA (0.54 mL, 3.04 mmol) in DMF (4.4 mL) at 0 ˚C, was added a solution of **15a** (358 mg, 2.54 mmol) in DMF (2.2 mL). Following 20 hours stirring at room temperature, the product was extracted from water (40 mL) with ethyl acetate (3x 20 mL) and the combined organic extracts were washed with brine (3 x 20 mL), dried over $MgSO_4$, filtered and concentrated *in vacuo*. The crude product was purified by flash column chromatography (silica gel; petroleum ether/EtOAc, 1:4) to yield the title compound as a mixture of diastereoisomers (*d.r.* ~5:6, determined by ^{1}H NMR using 6.98 ppm doublets) as a yellow oil (649 mg, 2.19 mmol, 86%).

R_f = 0.28 (petroleum ether/EtOAc, 1:4); Reported as a mixture of diastereoisomers; **^{1}H** NMR (400 MHz, $CDCl_3$): δ_H 6.97 (1H, 2x d, J = 7.8 Hz), 6.23 (1H, t, J = 9.1 Hz), 4.66 (1H, m), 4.55 (1H, m), 4.24 (2H, m), 2.76 (2H, m), 2.03 (4H, m), 1.67 (2H, m), 1.52 (1H, m), 1.29 (3H, m), 0.93 (6H, app t, J = 7.2 Hz); **^{13}C** NMR (101 MHz, $CDCl_3$): δ_C 172.1 (2C), 170.2, 170.1, 170.0 (2C), 78.2 (2C), 71.7, 71.6, 62.0 (2C), 51.5, 50.7 (2C), 41.4, 41.3, 24.8, 24.7, 23.1 (2C), 22.8 (2C), 22.3, 22.2 (2C), 22.1, 14.1; **HRMS** (ESI+): $[M+H]^+$ calculated ($C_{15}H_{25}N_2O_4$): 297.1809; observed: 297.1819; **IR** ν_{max} = 3285, 2960, 2120, 1742, 1651, 1544, 1535, 1372, 1280, 1214, 1022.

Synthesis of 2-((*S*)-2-acetamido-4-methylpentanamido)pent-4-ynoic acid (18e)

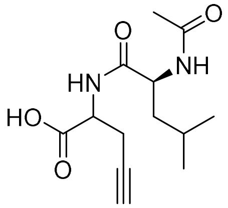

To a solution of **17g** (35 mg, 0.12 mmol) in THF (1.5 mL) and water (0.75 mL) was added LiOH monohydrate (14 mg, 0.33 mmol). After 1 hour stirring, the organics were removed *in vacuo* and the solution was acidified to pH 1 with 3 M HCl at 0 ˚C and extracted with ethyl acetate (3 x 10 mL). The combined organic layers were dried over Na_2SO_4, filtered and concentrated *in vacuo* to afford the title compound as a mixture of diastereoisomers (*d.r.*

~1:1.15, determined by ^{1}H NMR using 8.24 ppm doublets) as a thick, pale yellow oil (26 mg, 0.097 mmol, 82%) with no need for further purification.

Reported as a mixture of diastereoisomers; **^{1}H** NMR (400 MHz, DMSO-d$_6$): δ_H 8.24 (1H, 2x d, *J* = 7.8 and 7.4 Hz), 8.00 (1H, m), 4.35 (2H, m), 2.87 (1H, d, *J* = 11.5 Hz), 2.59 (2H, m), 1.83 (3H, 2x s), 1.59 (1H, m), 1.43 (2H, m), 0.85 (6H, m); **^{13}C** NMR (101 MHz, DMSO-d$_6$): δ_C 172.4, 172.3, 171.7, 169.2, 169.1, 80.4, 80.3, 73.2, 73.1, 59.9, 50.8, 50.7, 41.3, 41.0, 24.3, 24.2, 23.2, 23.1, 22.6, 21.7 (2C), 21.3, 21.0; **HRMS** (ESI+): $[M+H]^+$ calculated ($C_{13}H_{21}N_2O_4$): 269.1496; observed: 269.1499; **IR** ν_{max} = 3285, 3120, 2961, 2123, 1713, 1650.

Synthesis of (2*S*)-2-acetamido-4-methyl-*N*-(5-methylene-2-oxotetrahydrofuran-3-yl)pentanamide (7)

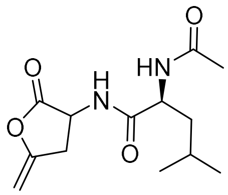

To a solution of **18e** (18 mg, 0.067 mmol) in water (0.6 mL) and ^{t}BuOH (0.6 mL) was added CuBr (3 mg, 0.021 mmol) and the reaction stirred for 24 hours at room temperature. The reaction was diluted with water (20 mL) and extracted with ethyl acetate (3x10 mL). The combined organic layers were washed with brine (2x10 mL), dried with Na_2SO_4, filtered and concentrated under reduced pressure. The crude product was purified by flash column chromatography (silica gel; petroleum ether/EtOAc, 1:4) to yield the title compound as a mixture of diastereoisomers (*d.r.* ~2:1, determined by ^{1}H NMR using ~2.00 ppm CH_3 singlet) as a thick, colourless oil (9.1 mg, 0.034 mmol, 51%).

R*f* = 0.26 (petroleum ether/EtOAc, 1:4); Reported for major diastereoisomer; **^{1}H** NMR (400 MHz, $CDCl_3$): δ_H 7.49 (1H, s br), 6.23 (1H, s br), 4.79 (1H, s), 4.52 (2H, m), 4.38 (1H, s), 3.04 (2H, m), 2.00 (3H, s), 1.66 (2H, m), 1.52 (1H, m), 0.92 (6H, q, *J* = 6.1 Hz); **^{13}C** NMR (100 MHz, $CDCl_3$): δ_C 172.7, 172.6, 170.1, 152.3, 90.2, 51.2, 49.4, 40.4, 32.4, 24.7, 23.1, 22.8, 22.1; **HRMS** (ESI+): $[M+H]^+$ calculated ($C_{13}H_{21}N_2O_4$): 269.1496; observed: 269.1492; **IR** ν_{max} = 3278, 2982, 2922, 1813, 1655, 1649, 1547, 1147.

(2*S*)-2-acetamido-*N*-(5-((*Z*)-benzylidene)-2-oxotetrahydrofuran-3-yl)-4-methylpentanamide (10)

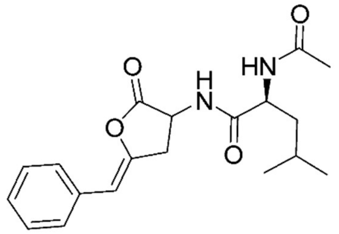

A solution of **17g** (12.4 mg, 0.0456 mmol), iodobenzene (5.1 µL, 0.0456 mmol) and $BnNEt_3Cl$ (10.4 mg, 0.0456 mmol) in acetonitrile (1.0 mL) and Et_3N (0.2 mL) was degassed with a stream of nitrogen for 30 minutes followed by three freeze-pump-thaw cycles. The reaction was frozen and $Pd(PPh_3)_4$ (5.3 mg, 0.0046 mmol) was added, the reaction was thawed at room temperature and then heated to reflux for 18 hours. The reaction mixture was concentrated under reduced pressure before being resuspended in DCM (10 mL), washed with water (3x10 mL), dried over $MgSO_4$, filtered and solvent removed *in vacuo*. The crude product was purified by flash column chromatography (silica gel; petroleum ether/EtOAc, gradient 1:1 to 1:4) to yield the title compound as a mixture of diastereoisomers (*d.r.* ~1:1, determined by ^{1}H NMR using 1.93 ppm Acetyl-CH_3 singlet) as a thick, yellow oil (9.1 mg, 0.026 mmol, 57%).

R_f = 0.42 (petroleum ether/EtOAc, 1:4); Reported as a mixture of diastereoisomers; **^{1}H** NMR (400 MHz, acetone-d_6): δ_H 8.16 (1H, s br), 7.63 (2H, m), 7.36 (3H, m), 7.21 (1H, t, *J* = 7.1 Hz), 6.26 (1H, s), 4.72 (1H, m), 4.48 (1H, m), 3.50 (1H, m, H21), 3.23 (1H, m), 1.93 (3H, 2x s, Ac-CH_3), 1.67 (2H, m), 1.52 (1H, m), 0.91 (6H, m); **^{13}C** NMR (100 MHz, acetone-d_6): δ_C 173.9, 173.8, 173.0 (2C), 170.7, 170.6, 150.9, 136.0, 133.1, 133.0, 129.8 (2C), 129.7, 129.0, 127.6, 107.3 (2C), 52.3, 52.2, 49.9 (2C), 42.1, 42.0, 33.2, 33.0, 25.7 (2C), 23.8, 23.7, 23.2 (2C), 22.4, 22.3; **HRMS** (ESI+): $[M+H]^+$ calculated ($C_{19}H_{25}N_2O_4$): 345.1809; observed: 345.1794; **IR** ν_{max} = 3298, 3066, 2960, 2922, 1809, 1678, 1652, 1535, 1448, 1438, 1370, 1279, 1175, 1118, 1075.

Synthesis of ethyl 2-((diphenylmethylene)amino)hex-5-ynoate (59)

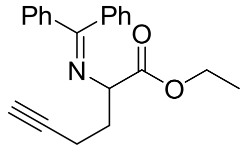

To a solution of **14** (3 g, 11.2 mmol) in acetonitrile (50 mL) was added K_2CO_3 (7.5 g, 54.5 mmol) and the reaction stirred for 1 hour. TBAI (1 g, 2.7 mmol) and 4-bromobut-1-yne (3.3 mL, 33.6 mmol) were added and the reaction mixture heated to reflux for 72 hours. During the

final 48 hours, further 4-bromobut-1-yne (6.6 mL, 67.2 mmol) and K_2CO_3 (7.5 g, 54.5 mmol) in total were added in four portions. The reaction mixture was cooled to room temperature, filtered and concentrated *in vacuo*, before being resuspended in diethyl ether (50 mL), washed with water (20 mL) and brine (20 mL), dried over $MgSO_4$, filtered and concentrated under reduced pressure to afford the title compound as a yellow oil (3.12 g, 9.78 mmol, 87%) with no need for further purification.

R*f* = 0.76 (petroleum ether/EtOAc, 4:1); **^{1}H** NMR (400 MHz, $CDCl_3$): δ_H 7.42 (10H, m), 4.19 (3H, m), 2.19 (2H, m), 1.79 (1H, s), 1.73 (2H, m), 1,25 (3H, q, *J* = 7.1 Hz); **^{13}C** NMR (100 MHz, $CDCl_3$): δ_C 171.8, 170.7, 139.3, 136.4, 130.5, 128.1, 83.4, 68.9, 63.9, 60.9, 32.5, 15.2, 14.2; **HRMS** (ESI+): $[M+H]^+$ calculated ($C_{21}H_{22}NO_2$): 320.1645; observed: 320.1646; **IR** ν_{max} = 3297, 2976, 2130, 1736, 1625, 1490, 1446, 1369, 1290, 1182, 1029.

Synthesis of ethyl 2-aminohex-5-ynoate (15e)

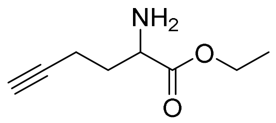

To a solution of **59** (3.11 g, 9.75 mmol) in diethyl ether (21 mL) was added 1 M HCl (14 mL) and the reaction was stirred for 15 hours. The separated aqueous layer was washed with diethyl ether (3 x 10 mL), cooled to 0 ˚C, basified to pH 10 with Na_2CO_3 and extracted with diethyl ether (3 x 10 mL). The combined organic layers were dried over Na_2SO_4, filtered and concentrated under reduced pressure to afford the title compound as a pale yellow oil (610 mg, 3.94 mmol, 40%) with no need for further purification.

R*f* = 0.30 (petroleum ether/EtOAc, 1:4); **^{1}H** NMR (400 MHz, $CDCl_3$): δ_H 4.13 (2H, q, *J* = 7.1 Hz), 3.51 (1H, m), 2.31 (2H, m), 1.93 (2H, m), 1.68 (1H, app. qn, *J* = 7.5 Hz), 1.22 (3H, t, *J* = 7.1 Hz); **^{13}C** NMR (100 MHz, $CDCl_3$): δ_C 175.5, 83.2, 69.1, 61.0, 53.3, 33.2, 15.1, 14.2; **HRMS** (ESI+): $[M+H]^+$ calculated ($C_8H_{14}NO_2$): 156.1019; observed: 156.1015; **IR** ν_{max} = 3297, 2979, 2936, 2115, 1727, 1446, 1378, 1300, 1185, 1023.

Synthesis of ethyl 2-((tert-butoxycarbonyl)amino)hex-5-ynoate (17h)

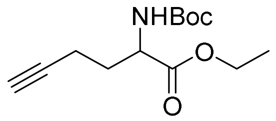

To a solution of **15e** (196 mg, 1.26 mmol) in DCM (4 mL) was added Boc_2O (275 mg, 1.26 mmol) and the reaction stirred for 12 hours at room temperature. The reaction mixture was concentrated under reduced pressure and the crude product purified by flash column chromatography (silica gel; petroleum ether/EtOAc, 9:1) to yield the title compound as a colourless oil (284 mg, 1.11 mmol, 88%).

R*f* = 0.58 (petroleum ether/EtOAc, 4:1); **^{1}H** NMR (400 MHz, $CDCl_3$): δ_H 4.34 (1H, s br), 4.18 (2H, q, *J* = 7.6 Hz), 2.26 (2H, app. t, *J* = 7.1 Hz), 2.06 (1H, m), 1.97 (1H, m), 1.85 (1H, m), 1.43 (9H, s), 1.27 (3H, t, *J* = 7.6 Hz); **^{13}C** NMR (100 MHz, $CDCl_3$): δ_C 172.2, 155.3, 82.9, 80.0, 69.2, 61.5, 52.8, 31.6, 28.3, 14.9, 14.1; **HRMS** (ESI+): $[M+H]^+$ calculated ($C_{13}H_{22}NO_4$): 256.1543; observed: 256.1533; **IR** ν_{max} = 3297, 2929, 2856, 2091, 1740, 1712, 1584, 1507, 1366, 1251, 1172, 1091, 1049, 990, 937, 833, 811, 775, 663.

Synthesis of 2-((tert-butoxycarbonyl)amino)hex-5-ynoic acid (18f)

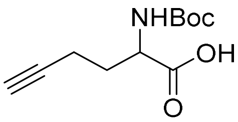

To a solution of **15e** (246 mg, 0.96 mmol) in THF (10 mL) and water (5 mL) was added LiOH monohydrate (100 mg, 2.38 mmol). After 1 hour stirring, the organics were removed *in vacuo* and the solution was acidified to pH 1 with 3 M HCl at 0 ˚C and extracted with ethyl acetate (3 x 10 mL). The combined organic layers were dried over Na_2SO_4, filtered and concentrated *in vacuo* to afford the title compound as a colourless oil (204 mg, 0.88 mmol, 92%) with no need for further purification.

R$_f$ = 0.11 (petroleum ether/EtOAc, 4:1); **^{1}H** NMR (400 MHz, $CDCl_3$): δ_H 8.48 (1H, s br), 4.38 (1H, m), 2.31 (2H, m), 2.13 (1H, m), 1.98 (1H, s), 1.91 (1H, m), 1.43 (9H, s); **^{13}C** NMR (100 MHz, $CDCl_3$): δ_C 176.7, 155.6, 82.6, 80.5, 69.6, 52.7, 31.1, 28.3, 15.0; **HRMS** (ESI+): $[M+H]^+$ calculated ($C_{11}H_{18}NO_4$): 228.1230; observed: 228.1234;); **IR** ν_{max} = 3304, 2936, 2921, 2146, 1712, 1701, 1516, 1369, 1158, 1052.

Synthesis of *tert*-butyl (6-methylene-2-oxotetrahydro-2*H*-pyran-3-yl)carbamate (11)

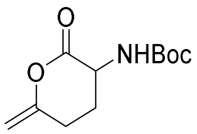

A solution of **18f** (24.4 mg, 0.106 mmol) in acetonitrile (2 mL) was degassed with a stream of nitrogen for 30 minutes followed by three freeze-pump-thaw cycles. The reaction was frozen and CuBr (4.5 mg, 0.032 mmol) and K_2CO_3 (1.5 mg, 0.011 mmol) were added, the reaction was thawed at room temperature and then stirred for 24 hours. The reaction mixture was concentrated under reduced pressure, resuspended in ethyl acetate (20 mL), washed with water (10 mL) and brine (10 mL), dried with Na_2SO_4, filtered and solvent removed *in vacuo*. The crude product was purified by flash column chromatography (silica gel; petroleum ether/EtOAc, 4:1) to afford the title compound as a white, amorphous solid (1.95 mg, 0.0084 mmol, 9%).

R_f = 0.31 (petroleum ether/EtOAc, 4:1); **^{1}H** NMR (400 MHz, $CDCl_3$): δ_H 4.73 (1H, s), 4.39 (1H, s), 4.29 (1H, s br), 2.67 (2H, m), 2.44 (1H, s br), 2.15 (1H, m), 1.43 (9H, s); **^{13}C** NMR (100 MHz, $CDCl_3$): δ_C 175.7, 154.0, 153.1, 96.2, 80.7, 61.9, 48.2, 30.2, 28.3; **HRMS** (ESI+): $[M+H]^+$ calculated ($C_{11}H_{18}NO_4$): 228.1230; observed: 228.1232; **IR** ν_{max} = 3311, 2913, 1748, 1701, 1633, 1402, 1369, 1158, 1052.

4.3 Hit 2: CFI_{25} Fragment Synthetic Procedures

Synthesis of methyl 2-(3-ethoxy-3-oxopropanamido)-2-methylpent-4-ynoate (34a)

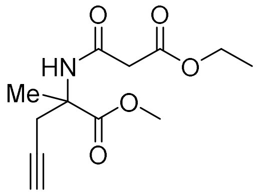

To an ice-cooled solution of **15b** (260 mg, 1.84 mmol) in CH_2Cl_2 (18 mL) was added triethylamine (0.385 mL, 2.76 mmol) followed by ethyl malonylchloride (0.354 mL, 2.76 mmol) portion-wise. The reaction was warmed to room temperature and stirred for 3 hours and then NH_4Cl saturated aqueous solution was added (20 mL). The organic layer was separated and the aqueous further extracted with CH_2Cl_2 (2 x 15 mL). The combined organic extracts were dried over Na_2SO_4 and concentrated *in vacuo*. The crude sample was purified by flash column chromatography (silica gel; Heptane/EtOAc, gradient 1:0 to 2:3) to give the title compound (494 mg, 1.23 mmol, 95%) as a colourless oil.

R_f = 0.23 (petroleum ether/EtOAc, 3:2); **^{1}H** NMR(400 MHz, $CDCl_3$) δ 7.81 (1H, br s), 4.20 (2H, q, *J* = 7.1 Hz), 3.75 (3H, s), 3.30 (2H, s), 2.96 (2H, m), 1.99 (1H, t, *J* = 2.7 Hz), 1.60 (3H, s), 1.28 (3H, t, *J* = 7.1 Hz);**^{13}C** NMR(101 MHz, $CDCl_3$) δ 173.2, 169.3, 164.6, 79.3, 71.3, 61.8,

58.8, 53.1, 41.5, 26.5, 22.8, 14.2; **HRMS** (ESI) calcd for $[C_{12}H_{18}NO_5]^+$: 256.1185, found 256.1189; **IR** ν_{max} = 3272, 2984, 1735, 1656, 1124.

Synthesis of methyl 2-(3-ethoxy-3-oxopropanamido)-2-phenylpent-4-ynoate (34b)

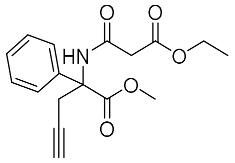

To an ice-cooled solution of **15c** (100 mg, 0.490 mmol) in CH_2Cl_2 (5 mL) was added triethylamine (0.103 mL, 0.740 mmol), followed by ethyl 3-chloro-3-oxopropanoate (0.094 mL, 0.740 mmol) dropwise. The reaction was stirred for 3 hours 45 minutes and then diluted with NH_4Cl saturated aqueous solution. The organic layer was separated, and the aqueous further extracted with CH_2Cl_2 (2 x 10 mL). The organic extracts were combined, dried over Na_2SO_4 and concentrated *in vacuo*. The sample was purified by flash column chromatography (silica gel; heptane/EtOAc, gradient 10 – 60%) to give the title compound (146 mg, 0.460 mmol, 94 %) as a yellow oil.

R_f = 0.36 (Petroleum ether/EtOAc, 3:2); **^{1}H** (400 MHz, $CDCl_3$) δ 8.54 (1H, br s), 7.47 – 7.44 (2H, m), 7.40 – 7.36 (2H, m), 7.34 – 7.30 (1H, m), 4.25 (2H, q, *J* = 7.2 Hz), 3.72 (3H, s), 3.66 (1H, dd, *J* = 16.7, 2.6 Hz), 3.50 (1H, dd, *J* = 16.7, 2.6 Hz), 3.43 – 3.33 (2H, m), 1.94 (1H, t, *J* = 2.6 Hz), 1.32 (3H, t, *J* = 7.1 Hz); **^{13}C** NMR (101 MHz, $CDCl_3$) δ 171.6, 169.3, 164.2, 137.6, 128.9, 128.5, 125.9, 79.5, 71.4, 64.8, 61.9, 53.6, 41.8, 24.7, 14.2**;** **HRMS** (ESI) calcd for $[C_{17}H_{20}NO_5]^+$: 318.1341, found 318.1345; **IR** ν_{max} = 3287, 2983, 1735, 1677, 1519, 1498, 1436.

General procedure 1

To a solution of alkyne (1 eq.) in *t*-BuOH (1.5 mL) and H_2O (1.5 mL) was added copper(II) sulfate pentahydrate (0.1 eq) and sodium ascorbate (0.3 eq.). The corresponding azide (0.9 eq.) was then added and the reaction stirred for 16 hours. Upon completion, H_2O was added and the mixture extracted with EtOAc (3 x). The combined organic extracts were dried (Na_2SO_4), concentrated *in vacuo* and purified by flash column chromatography to give the product.

General procedure 2

To a solution of triazole (1 eq.) in THF (0.1 M) was added potassium *tert*-butoxide (1.5 eq.) the reaction refluxed for 1-4 hours. Upon completion, 1 N HCl (aq.) was added and extracted with EtOAc (3 x). The combined organic extracts were dried over Na_2SO_4 and concentrated *in vacuo*. The crude intermediate was dissolved in acetonitrile and H_2O (10:1, 0.1 M) and refluxed until no starting material remained (2-4 hours). The solution was concentrated *in vacuo* and purified by flash chromatography to give the product.

Synthesis of methyl 2-(3-ethoxy-3-oxopropanamido)-2-methyl-3-(1-phenyl-1*H*-1,2,3-triazol-4-yl)propanoate (36a)

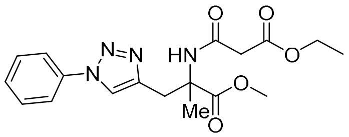

Following general procedure 1: **34a** (400 mg, 1.57 mmol), copper(II) sulfate pentahydrate (39.1 mg, 0.156 mmol), sodium ascorbate (93.0 mg, 0.469 mmol), azidobenzene (0.5M in TBME, 2.82 mL, 1.41 mmol), *t*-BuOH (8 mL) and water (8 mL) were used. The crude product was purified by flash column chromatography (silica gel; heptane/EtOAc, gradient 1:3 to 0:1) to give the title compound (450 mg, 1.20 mmol, 77%) as a white solid.

R*f* = 0.39 (petroleum ether/EtOAc, 7:13); **^{1}H** NMR (400 MHz, $CDCl_3$) δ 7.91 (1H, s), 7.76 – 7.74 (2H, m), 7.52 – 7.49 (2H, m), 7.44 – 7.39 (2H, m), 4.14 (2H, q, *J* = 7.2 Hz), 3.82 (3H, s), 3.70 (1H, d, *J* = 14.6 Hz), 3.48 (1H, d, *J* = 14.6 Hz), 3.28 (2H, s), 1.69 (3H, s), 1.22 (3H, t, *J* = 7.2 Hz); **^{13}C** NMR (101 MHz, $CDCl_3$) δ 174.0, 168.8, 164.7, 143.4, 137.2, 129.8, 128.7, 121.2, 120.4, 61.8, 60.3, 53.2, 42.7, 32.1, 23.2, 14.1; **HRMS** (ESI) calcd for $[C_{18}H_{23}N_4O_5]^+$: 375.1668, found 375.1670; **IR** ν_{max} = 3256, 3157, 3083, 1732, 1645, 1564, 1505, 1426.

Synthesis of methyl 3-(1-(2-chlorophenyl)-1*H*-1,2,3-triazol-4-yl)-2-(3-ethoxy-3-oxopropanamido)-2-methylpropanoate (36b)

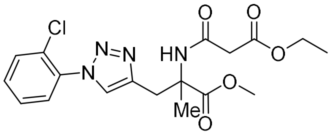

Following general procedure 1: **34a** (65.0 mg, 0.255 mmol), copper(II) sulfate pentahydrate (6.36 mg, 0.0255 mmol), sodium ascorbate (15.1 mg, 0.0762 mmol), 1-azido-2-chlorobenzene

(0.5 M in TBME, 0.458 mL, 0.230 mmol), *t*-BuOH (1.5 mL) and water (1.5 mL) were used. After 16 hours, further copper(II) sulfate pentahydrate (6.36 mg, 0.0255 mmol) was added and the reaction stirred for a further 16 hours. The crude product was purified by flash column chromatography (silica gel; heptane/EtOAc, gradient 1:4 to 0:1) to give the title compound (70.0 mg, 0.172 mmol, 69%) as a colourless oil.

R_f = 0.35 (petroleum ether/EtOAc, 7:13); **^{1}H** NMR (400 MHz, $CDCl_3$) δ 7.80 (1H, s), 7.65 (1H, br s), 7.63 – 7.60 (1H, m), 7.58 – 7.54 (1H, m), 7.47 – 7.41 (2H, m), 4.15 (2H, q, *J* = 7.2 Hz), 3.82 (3H, s), 3.68 (1H, d, *J* = 7.6 Hz), 3.51 (1H, d, *J* = 7.6 Hz), 3.28 (2H, s), 1.69 (3H, s), 1.24 (3H, t, *J* = 7.1 Hz); **^{13}C** NMR (101 MHz, $CDCl_3$) δ 173.9, 168.8, 164.7, 142.6, 135.1, 130.9, 130.8, 128.7, 128.0, 127.9, 125.0, 61.7, 60.2, 53.2, 42.3, 32.1, 23.3, 14.2; **HRMS** (ESI) calcd for $[C_{18}H_{22}N_4O_5{}^{35}Cl]^+$: 409.1279, found 409.1268; **IR** ν_{max} = 3303, 2986, 1735, 1676, 1670, 1535, 1496, 1456.

Synthesis of methyl 2-(3-ethoxy-3-oxopropanamido)-2-methyl-3-(1-(2-(trifluoromethyl)phenyl)-1*H*-1,2,3-triazol-4-yl)propanoate (36c)

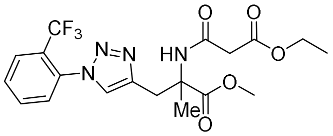

Following general procedure 1: **34a** (65.0 mg, 0.255 mmol), copper(II) sulfate pentahydrate (6.36 mg, 0.0255 mmol), sodium ascorbate (15.1 mg, 0.0762 mmol), 1-azido-2-(trifluoromethyl) benzene (0.5M in TBME, 0.458 mL, 0.230 mmol), *t*-BuOH (1.5 mL) and water (1.5 mL) were used. The crude product was purified by flash column chromatography (silica gel; heptane/EtOAc, gradient 2:3 to 0:1) to give the title compound (78.0 mg, 0.176 mmol, 70%) as a colourless oil.

R_f = 0.32 (heptane/EtOAc, 7:3); **^{1}H** NMR (400 MHz, $CDCl_3$) δ 7.85 – 7.83 (1H, m), 7.75 – 7.71 (1H, m), 7.68 – 7.66 (3H, m), 7.56 (1H, d, *J* = 8.0 Hz), 4.16 (2H, q, *J* = 7.1 Hz), 3.81 (3H, s), 3.68 (1H, d, *J* = 14.7 Hz), 3.52 (1H, d, *J* = 14.6 Hz), 3.27 (2H, s), 1.69 (3H, s), 1.24 (3H, t, *J* = 7.2 Hz); **^{13}C** NMR (101 MHz, $CDCl_3$) δ 173.9, 168.8, 164.7, 142.8, 135.1 (q, *J* = 2.4 Hz), 133.2,130.4, 129.1, 127.4 (q, *J* = 3.2 Hz), 125.6 (q, *J* = 38 Hz), 125.4, 125.0 (q, *J* = 220 Hz), 61.7, 60.2, 53.1, 42.1, 32.0, 23.3, 14.1; **^{19}F** (376 MHz, $CDCl_3$) δ -59.24; **HRMS** (ESI) calcd for $[C_{19}H_{22}N_4O_5F_3]^+$: 443.1537, found 443.1523; **IR** ν_{max} = 3315, 2988, 1736, 1677, 1608, 1509, 1316.

Synthesis of methyl 2-(3-ethoxy-3-oxopropanamido)-3-(1-(2-methoxyphenyl)-1*H*-1,2,3-triazol-4-yl)-2-methylpropanoate (36d)

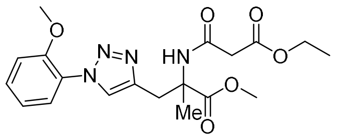

Following general procedure 1: **34a** (65.0 mg, 0.255 mmol), copper(II) sulfate pentahydrate (6.36 mg, 0.0255 mmol), sodium ascorbate (15.1 mg, 0.0762 mmol), 1-azido-2-methoxybenzene (0.5M in TBME, 0.458 mL, 0.230 mmol), *t*-BuOH (1.5 mL) and water (1.5 mL) were used. The crude product was purified by flash column chromatography (silica gel; heptane/EtOAc, gradient 1:1 to 0:1) to give the title compound (66.0 mg, 0.163 mmol, 65%) as a white solid.

R*f* = 0.27 (petroleum ether/EtOAc, 7:13); **^{1}H** NMR (400 MHz, $CDCl_3$) δ 7.92 (1H, s), 7.78 (1H, dd, *J* = 7.9, 1.7 Hz), 7.62 (1H, br s), 7.43 – 7.38 (1H, m), 7.12 – 7.06 (2H, m), 4.14 (2H, q, *J* = 7.2 Hz), 3.88 (3H, s), 3.81 (3H, s), 3.61 (1H, d, *J* = 14.6 Hz), 3.47 (1H, d, *J* = 14.6 Hz), 3.28 (2H, s), 1.69 (3H, s), 1.22 (3H, t, *J* = 7.2 Hz); **^{13}C** NMR (101 MHz, $CDCl_3$) δ 174.0, 168.7, 164.6, 151.1, 142.1, 130.0, 126.5, 125.5, 125.0, 121.4, 112.4, 61.7, 60.2, 56.1, 53.1, 42.5, 32.4, 23.1, 14.1; **HRMS** (ESI) calcd for $[C_{19}H_{25}N_4O_6]^+$: 405.1774, found 405.1787; **IR** ν_{max} = 3251, 3056, 1752, 1731, 1670, 1557, 1504, 1453, 1119.

Synthesis of methyl 3-(1-(3-chlorophenyl)-1*H*-1,2,3-triazol-4-yl)-2-(3-ethoxy-3-oxopropanamido)-2-methylpropanoate (36e)

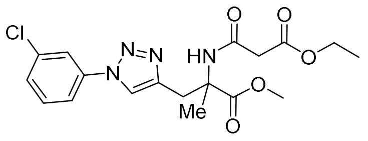

Following general procedure 1: **34a** (65.0 mg, 0.255 mmol), copper(II) sulfate pentahydrate (6.36 mg, 0.0255 mmol), sodium ascorbate (15.1 mg, 0.0762 mmol), 1-azido-3-chlorobenzene (0.5M in TBME, 0.458 mL, 0.230 mmol), *t*-BuOH (1.5 mL) and water (1.5 mL) were used. The crude product was purified by flash column chromatography (silica gel; heptane/EtOAc, gradient 1:4 to 0:1) to give the title compound (69.0 mg, 0.168 mmol, 68%) as a white solid.

R*f* = 0.34 (Heptane/EtOAc, 2:3); **^{1}H** NMR (400 MHz, $CDCl_3$) δ 8.01 (1H, s), 7.84 (1H, t, *J* = 2.0 Hz), 7.72 – 7.69 (1H, m), 7.45 (1H, t, *J* = 8.0 Hz), 7.40 – 7.38 (1H, m), 7.33 (1H, br s), 4.20 – 4.16 (2H, m), 3.83 (3H, s), 3.75 (1H, d, *J* = 14.6 Hz), 3.49 (1H, d, *J* = 14.6 Hz), 3.30 (2H, s), 1.70 (3H, s), 1.26 (3H, t, *J* = 4.8 Hz); **^{13}C** NMR (101 MHz, $CDCl_3$) δ 173.8, 168.7, 164.5, 143.5,

137.9, 135.5, 130.7, 128.5, 121.2, 120.5, 118.2, 61.7, 60.3, 53.1, 42.7, 31.8, 23.1, 14.0; **HRMS** (ESI) calcd for $[C_{18}H_{22}N_4O_5{}^{35}Cl]^+$: 409.1279, found 409.1277; **IR** ν_{max} = 3255, 3079, 2986, 1744, 1729, 1642, 1597, 1567, 1197.

Synthesis of methyl 2-(3-ethoxy-3-oxopropanamido)-2-methyl-3-(1-(3-(trifluoromethyl)phenyl)-1*H*-1,2,3-triazol-4-yl)propanoate (36f)

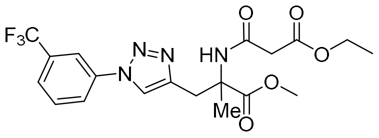

Following general procedure 1: **34a** (65.0 mg, 0.255 mmol), copper(II) sulfate pentahydrate (6.36 mg, 0.0255 mmol), sodium ascorbate (15.1 mg, 0.0762 mmol), 1-azido-3-(trifluoromethyl) benzene (0.5M in TBME, 0.458 mL, 0.230 mmol), *t*-BuOH (1.5 mL) and water (1.5 mL) were used. The crude product was purified by flash column chromatography (silica gel; heptane/EtOAc, gradient 1:4 to 0:1) to give the title compound (93.0 mg, 0.210 mmol, 84%) as a white solid.

$\mathbf{R}_f$ = 0.47 (petroleum ether/EtOAc, 7:13); **^{1}H** NMR (400MHz, $CDCl_3$) δ 8.15 (1H, s), 8.11 – 8.10 (1H, m), 8.06 – 8.04 (1H, m), 7.69 – 7.63 (2H, m), 7.24 (1H, br s), 4.20 – 4.14 (2H, m), 3.84 (3H, s), 3.80 (1H, d, *J* = 14.6 Hz), 3.51 (1H, d, *J* = 14.6 Hz), 3.30 (2H, s), 1.71 (3H, s), 1.25 (3H, t, *J* = 7.1 Hz); **^{13}C** NMR (101MHz, $CDCl_3$) 173.8, 168.8, 164.6, 143.7, 137.5, 132.3 (q, *J* = 33.2 Hz), 130.4, 125.0 (q, *J* = 3.7 Hz), 123.4 (q, *J* = 272.9 Hz), 123.3, 121.4, 117.0 (q, *J* = 4.0 Hz), 61.7, 60.4, 53.2, 42.8, 31.7, 23.2, 14.0; **^{19}F** (376 MHz, $CDCl_3$) δ -62.9; **HRMS** (ESI) calcd for $[C_{19}H_{22}N_4O_5F_3]^+$: 443.1542, found 443.1558; **IR** ν_{max} = 3277, 1736, 1675, 1661, 1535, 1460, 1126.

Synthesis of methyl 2-(3-methoxy-3-oxopropanamido)-3-(1-(3-methoxyphenyl)-1H-1,2,3-triazol-4-yl)-2-methylpropanoate (36g)

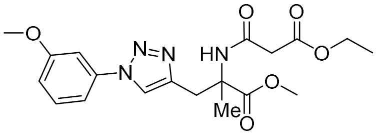

Following general procedure 1: **34a** (65.0 mg, 0.255 mmol), copper(II) sulfate pentahydrate (6.36 mg, 0.0255 mmol), sodium ascorbate (15.1 mg, 0.0762 mmol), 1-azido-3-methoxybenzene (0.5M in TBME, 0.458 mL, 0.230 mmol), *t*-BuOH (1.5 mL) and water (1.5

mL) were used. The crude product was purified by flash column chromatography (silica gel; heptane/EtOAc, gradient 1:4 to 0:1) to give the title compound (67.0 mg, 0.172 mmol, 69%) as a colourless oil.

R*f* = 0.37 (petroleum ether/EtOAc, 7:13); **^{1}H** NMR (400 MHz, $CDCl_3$) δ 7.91 (1H, s), 7.41 – 7.37 (3H, m), 7.30 – 7.28 (1H, m), 6.96 – 6.93 (1H, m), 4.17 – 4.12 (2H, m), 3.88 (3H, s), 3.83 (3H, s), 3.71 (1H, d, *J* = 14.6 Hz), 3.48 (1H, d, *J* = 14.6 Hz), 3.28 (2H, s), 1.69 (3H, s), 1.23 (3H, t, *J* = 7.2 Hz); **^{13}C** NMR (101 MHz, $CDCl_3$) δ 173.9, 169.2, 164.5, 160.7, 143.3, 138.2, 130.6, 121.3, 114.7, 112.2, 106.0, 61.8, 60.4, 55.8, 53.2, 42.5, 32.1, 23.2, 14.1; **HRMS** (ESI) calcd for $[C_{19}H_{24}N_4O_6Na]^+$: 427.1588, found 427.1586; **IR** ν_{max} = 3264, 3083, 2952, 1733, 1660, 1648, 1610, 1595, 1498.

Synthesis of methyl 2-(3-ethoxy-3-oxopropanamido)-2-methyl-3-(1-(*p*-tolyl)-1*H*-1,2,3-triazol-4-yl)propanoate (36h)

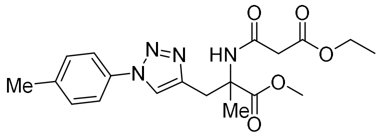

Following general procedure 1: **34a** (65.0 mg, 0.255 mmol), copper(II) sulfate pentahydrate (6.36 mg, 0.0255 mmol), sodium ascorbate (15.1 mg, 0.0762 mmol), 1-azido-2-(trifluoromethyl) benzene (0.5M in TBME, 0.458 mL, 0.230 mmol), *t*-BuOH (1.5 mL) and water (1.5 mL) were used. The crude product was purified by flash column chromatography (silica gel; heptane/EtOAc, gradient 1:4 to 0:1) to give the title compound (64.0 mg, 0.165 mmol, 66%) as a white solid.

R*f* = 0.32 (Heptane/EtOAc, 3:2); **^{1}H** NMR (400 MHz, $CDCl_3$) δ 7.86 (1H, s), 7.61 (2H, dt, *J* = 8.9, 2.0 Hz), 7.45 (1H, br s), 7.29 (2H, d, *J* = 8.1 Hz), 4.14 (2H, q, *J* = 8.1 Hz), 3.82 (3H, s), 3.67 (1H, d, *J* = 14.6 Hz), 3.47 (1H, d, *J* = 14.6 Hz), 3.28 (2H, s), 2.41 (3H, s), 1.69 (3H, s), 1.23 (3H, t, *J* = 7.2 Hz); **^{13}C** NMR (101 MHz, $CDCl_3$) δ 174.0, 168.7, 164.7, 143.3, 138.7, 134.9, 130.3, 121.2, 120.3, 61.8, 60.3, 53.2, 42.7, 32.1, 23.2, 21.2, 14.1; **HRMS** (ESI) calcd for $[C_{19}H_{25}N_4O_5]^+$: 389.1825, found 389.1831; **IR** ν_{max} = 3251, 3078, 2979, 1745, 1732, 1643, 1568, 1520, 1197.

Synthesis of methyl 3-(1-(4-chlorophenyl)-1*H*-1,2,3-triazol-4-yl)-2-(3-ethoxy-3-oxopropanamido)-2-methylpropanoate (36i)

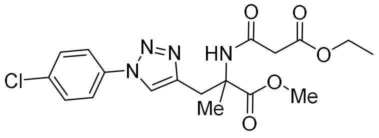

Following general procedure 1: **34a** (65.0 mg, 0.255 mmol), copper(II) sulfate pentahydrate (6.36 mg, 0.0255 mmol), sodium ascorbate (15.1 mg, 0.0762 mmol), 1-azido-4-chlorobenzene (0.5M in TBME, 0.458 mL, 0.230 mmol), *t*-BuOH (1.5 mL) and water (1.5 mL) were used. The crude product was purified by flash column chromatography (silica gel; heptane/EtOAc, gradient 1:4 to 0:1) to give the title compound (76.0 mg, 0.186 mmol, 74%) as a white solid.

R*f* = 0.41 (Heptane/EtOAc, 2:3); **^{1}H** NMR (400 MHz, $CDCl_3$) δ 7.94 (1H, s), 7.72 (2H, dt, *J* = 9.7, 2.5 Hz), 7.48 (2H, dt, *J* = 9.7, 2.5 Hz), 7.35 (1H, br s), 4.14 (2H, q, *J* = 7.2 Hz), 3.83 (3H, s), 3.74 (1H, d, *J* = 14.6 Hz), 3.48 (1H, d, *J* = 14.7 Hz), 3.28 (2H, s), 1.70 (3H, s), 1.24 (3H, t, *J* = 7.1 Hz); **^{13}C** NMR (101 MHz, $CDCl_3$) δ 173.9, 168.8, 164.7, 143.7, 135.7, 134.4, 130.0, 121.5, 121.3, 61.8, 60.4, 53.3, 42.9, 32.0, 23.3, 14.2; **HRMS** (ESI) calcd for $[C_{18}H_{22}N_4O_5{}^{35}Cl]^+$: 409.1279, found 409.1287; **IR** ν_{max} = 3258, 3078, 2981, 1744, 1729, 1641, 1563, 1502, 1198.

Synthesis of methyl 2-(3-ethoxy-3-oxopropanamido)-3-(1-(4-fluorophenyl)-1*H*-1,2,3-triazol-4-yl)-2-methylpropanoate (36j)

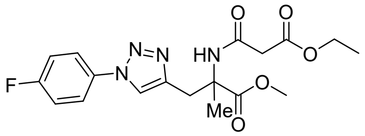

Following general procedure 1: **34a** (65.0 mg, 0.255 mmol), copper(II) sulfate pentahydrate (6.36 mg, 0.0255 mmol), sodium ascorbate (15.1 mg, 0.0762 mmol), 1-azido-4-fluorobenzene (0.5M in TBME, 0.458 mL, 0.230 mmol), *t*-BuOH (1.5 mL) and water (1.5 mL) were used. The crude product was purified by flash column chromatography (silica gel; heptane/EtOAc, gradient 1:4 to 0:1) to give the title compound (81.0 mg, 0.206 mmol, 83%) as a white solid.

R*f* = 0.37 (petroleum ether/EtOAc, 2:3); **^{1}H** NMR (400 MHz, $CDCl_3$) δ 7.90 (1H, s), 7.77 – 7.72 (2H, m), 7.37 (1H, br s), 7.23 – 7.17 (2H, m), 4.17 – 4.12 (2H, m), 3.83 (3H, s), 3.72 (1H, d, *J* =14.7 Hz), 3.48 (1H, d, *J* = 14.8 Hz), 3.28 (2H, s), 1.70 (3H, s), 1.24 (3H, t, *J* = 7.2 Hz); **^{13}C** NMR (101 MHz, $CDCl_3$) δ 174.0, 168.8, 164.6, 162.3 (d, *J* = 234.4 Hz), 143.6, 133.5, 122.3 (d, *J* = 8.7 Hz), 121.5, 116.8 (d, *J* = 23.0 Hz), 61.8, 60.4, 53.2, 42.8, 32.0, 23.3, 14.2; **^{19}F** (376

MHz, $CDCl_3$) δ -112.7; **HRMS** (ESI) calcd for $[C_{18}H_{22}N_4O_5F]^+$: 393.1574, found 393.1563; **IR** ν_{max} = 3356, 2928, 1736, 1665, 1517.

Synthesis of 5-methyl-5-((1-phenyl-1*H*-1,2,3-triazol-4-yl)methyl)pyrrolidine-2,4-dione (19)

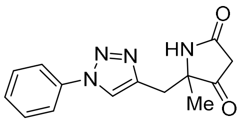

Following general procedure 2: **36a** (450 mg, 1.20 mmol) potassium *tert*-butoxide (202 mg, 1.80 mmol) and THF (12 mL) were refluxed for 3 hours. After work up, the crude intermediate was dissolved in acetonitrile (10 mL) and water (1 mL) and refluxed for 3 hours. The crude product was purified by flash column chromatography (silica gel; heptane/EtOAc, gradient 1:4 to 0:1) to give the title compound (240 mg, 0.888 mmol, 74%) as a white solid.

R$_f$ = 0.13 (EtOAc); **^{1}H** NMR (400 MHz, $CDCl_3$) δ 7.83 (1H, s), 7.68 (2H, d, *J* = 7.8 Hz), 7.52 – 7.48 (2H, m), 7.45 – 7.41 (1H, m), 7.34 (1H, br s), 3.21 (1H, d, *J* = 15.0 Hz), 3.13 (1H, d, *J* = 15.0 Hz), 3.07 (1H, d, *J* = 22.2 Hz), 2.92 (1H, d, *J* = 22.2 Hz), 1.46 (3H, s); **^{13}C** NMR (101 MHz, $CDCl_3$) δ; 209.6, 169.7, 142.5, 136.9, 129.9, 129.0, 121.0, 120.5, 67.7, 40.3, 33.8, 23.9; **HRMS** (ESI) calcd for $[C_{14}H_{14}N_4O_2]^+$: 271.1190, found 271.1191; **IR** ν_{max} = 3137, 2899, 1759, 1690, 1600, 1505.

This compound was prepared as a member of our previously reported library.[12]

Synthesis of 3,3,5-trimethyl-5-((1-phenyl-1*H*-1,2,3-triazol-4-yl)methyl)pyrrolidine-2,4-dione (32)

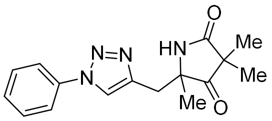

To a solution of **19** (25.0 mg, 0.0924 mmol) in DMF (1 mL) was added potassium carbonate (26.0 mg, 0.188 mmol) followed by iodomethane (0.0120 mL, 0.184 mmol). After stirring at room temp room temperature for 18 hours, iodomethane (0.0120 mL, 0.184 mmol) was added and the reaction heated to 60 °C for 7 hours. The reaction was quenched with NH_4Cl saturated aqueous solution and extracted with EtOAc (3 x 10 mL). The combined organic extracts were washed with brine, dried over Na_2SO_4 and concentrated *in vacuo.* The crude product was

purified by flash column chromatography (silica gel; heptane/EtOAc, gradient 4:1 to 0:1) to give the title compound (10.0 mg, 0.0335 mmol, 36%) as a yellow solid.

R*f* = 0.33 (EtOAc); **^{1}H** NMR (400 MHz $CDCl_3$) δ 7.80 (1H, s), 7.70 – 7.68 (2H, m), 7.54 – 7.50 (2H, m), 7.46 – 7.42 (1H, m), 6.96 (1H, br s), 3.19 – 3.10 (2H, m), 1.46 (3H, s), 1.30 (3H, s), 1.06 (3H, s); **^{13}C** NMR (101 MHz $CDCl_3$) δ 216.6, 176.7, 142.6, 136.9, 130.0, 129.1, 121.2, 120.6, 65.7, 46.3, 34.2, 25.0, 22.8, 20.4; **HRMS** (ESI) calcd for $[C_{16}H_{19}N_4O_2]^+$: 299.1508, found 299.1512; **IR** ν_{max} = 3149, 2971, 2932, 1693, 1655, 1596, 1502.

Synthesis of 4-hydroxy-5-methyl-5-((1-phenyl-1*H*-1,2,3-triazol-4-yl)methyl)pyrrolidin-2-one (31)

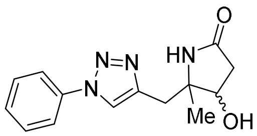

To an ice-cooled solution of **19** (40.0 mg, 0.148 mmol) in MeOH (1.5 mL) was added sodium borohydride (108 mg, 0.285 mmol) and the reaction stirred for 3 days at room temperature Then, NH_4Cl saturated aqueous solution was added and the reaction extracted with EtOAc (3 x 10 mL). The combined organic extracts were dried over Na_2SO_4 and concentrated *in vacuo* to give the title compound (*dr ca.* 77:23 by ^{1}H NMR, 40.0 mg, 0.147 mmol, 99%) as a white solid.

R*f* = 0.45, 0.39 ($MeOH/CH_2Cl_2$, 1:9); Reported for major diastereoisomer; **^{1}H** NMR (400 MHz, $CDCl_3$) δ 7.87 (1H, s), 7.74 – 7.72 (2H, m), 7.56 – 7.52 (2H, m), 7.46 (1H, t, *J* = 7.4 Hz), 6.69 (1H, br s), 5.11 (1H, m), 4.59 (1H, t, *J* = 8.1 Hz), 4.30 (1H, br s), 4.19 – 4.16 (1H, m), 3.25 (1H, d, *J* = 14.4 Hz), 3.09 (3H,m), 2.76 (1H, dd, *J* = 17.6, 7.0 Hz), 2.66 (1H, dd, *J* = 16.9, 7.9 Hz), 2.51 (1H, dd, *J* = 16.8, 8.5 Hz), 2.28 (1H, dd, *J* = 17.6, 3.2 Hz), 1.22 (3H, s); **^{13}C** NMR (101 MHz, $CDCl_3$) δ 175.5, 144.2, 136.9, 130.0, 129.2, 121.3, 120.6, 74.0, 63.7, 39.3, 32.3, 25.7; **HRMS** (ESI) calcd for $[C_{14}H_{17}N_4O_2]^+$: 273.1352, found 273.1344; **IR** ν_{max} = 3161, 2975, 2917, 1667, 1597, 1502.

Synthesis of 5-((1-(2-chlorophenyl)-1*H*-1,2,3-triazol-4-yl)methyl)-5-methylpyrrolidine-2,4-dione (20)

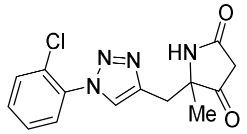

Following general procedure 2: **36b** (50.0 mg, 0.122 mmol), potassium *tert*-butoxide (27.0 mg, 0.241 mmol) and THF (1.4 mL) were refluxed for 3 hours. After work up, the crude intermediate was dissolved in acetonitrile (3 mL) and H_2O (0.3 mL) and refluxed for 4 hours. The crude product was purified by flash column chromatography (silica gel; heptane/EtOAc, gradient 1:4 to 0:1) to give the title compound (19.0 mg, 0.0624 mmol, 51%) as a white solid.

R*f* = 0.22 (EtOAc/MeOH, 24:1); **^{1}H** NMR (400 MHz, $CDCl_3$) δ 7.81 (1H, s), 7.60 – 7.56 (2H, m), 7.48 – 7.42 (2H, m), 7.11 (1H, br s), 3.25 (1H, d, *J* = 15.0 Hz), 3.14 (1H, d, *J* = 15.0 Hz), 3.06 (1H, d, *J* = 22.2 Hz), 2.91 (1H, d, *J* = 22.2 Hz), 1.46 (3H, s); **^{13}C** NMR (101 MHz, $CDCl_3$) δ 209.5, 169.4, 141.6, 134.8, 131.1, 130.9, 128.7, 128.2, 127.8, 124.9, 67.6, 40.2, 31.1, 24.0; **HRMS** calcd for $[C_{14}H_{13}N_4O_2{}^{35}ClNa]^+$: 327.0619, found 327.0618; **IR** ν_{max} = 3250, 2931, 1635, 1620, 1493.

Synthesis of 5-methyl-5-((1-(2-(trifluoromethyl)phenyl)-1*H*-1,2,3-triazol-4-yl)methyl)pyrrolidine-2,4-dione (21)

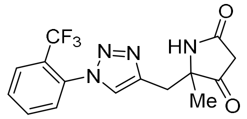

Following general procedure 2: **36c** (70.0 mg, 0.158 mmol), potassium *tert*-butoxide (26.6 mg, 0.237 mmol) and THF (2 mL) were refluxed for 3 hours. After work up, the crude intermediate was dissolved in acetonitrile (2 mL) and H_2O (0.2 mL) and refluxed for 3.5 hours. The crude product was purified by flash column chromatography (silica gel; gradient heptane/EtOAc, 1:1 to 0:1) to give the title compound (34.0 mg, 0.100 mmol, 64%) as a white solid.

R*f* = 0.20 (EtOAc); **^{1}H** NMR (400 MHz, $CDCl_3$) δ 7.87 (1H, d, *J* = 7.8 Hz), 7.78 – 7.74 (1H, m), 7.70 (1H, t, *J* = 7.7 Hz), 7.66 (1H, s), 7.55 (1H, d, *J* = 7.7 Hz), 6.72 (1H, br s), 3.23 (1H, d, *J* = 15.0 Hz), 3.14 – 3.07 (2H, m), 2.95 (1H, d, *J* = 22.2 Hz), 1.43 (3H, s); **^{13}C** NMR (101 MHz, d_6-DMSO) δ 211.2, 169. 2, 141.5, 134.3 (q, *J* = 1.7 Hz), 133.9, 131.1, 130.0 (q, *J* = 191.1 Hz), 129.2, 127.4 (q, *J* = 4.8 Hz), 126.2, 124.8 (q, J = 31.2 Hz), 66.6, 40.3, 33.6, 23.8; **^{19}F** (376

MHz, d_6-DMSO) δ 58.1; **HRMS** (ESI) calcd for $[C_{15}H_{14}N_4O_2F_3]^+$: 339.1069, found 339.1069; **IR** ν_{max} = 3241, 1635, 1625, 1503, 1467, 1405.

Synthesis of 5-((1-(2-methoxyphenyl)-1*H*-1,2,3-triazol-4-yl)methyl)-5-methylpyrrolidine-2,4-dione (22)

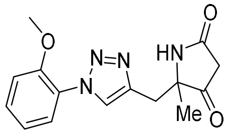

Following general procedure 2: **36d** (52.0 mg, 0.129 mmol), potassium *tert*-butoxide (21.0 mg, 0.187 mmol) and THF (1.5 mL) were refluxed for 2 hours. After work up, the crude intermediate was dissolved in acetonitrile (1 mL) and H_2O (0.1 mL) and refluxed for 3 hours. The crude product was purified by flash column chromatography (silica gel; heptane/EtOAc, gradient 1:4 to 0:1) to give the title compound (24.0 mg, 0.0799 mmol, 62%) as a white solid.

R_f = 0.11 (EtOAc); **^{1}H** NMR (400 MHz, $CDCl_3$) δ 7.92 (1H, s), 7.74 (1H, dd, *J* = 7.9, 1.5 Hz), 7.45 – 7.40 (1H, m), 7.12 – 7.06 (3H, m), 3.88 (3H, s), 3.22 (1H, d, *J* = 15.0 Hz), 3.12 (1H, d, *J* = 15.0 Hz), 3.04 (1H, d, *J* = 22.2 Hz), 2.89 (1H, d, *J* = 22.2 Hz), 1.46 (3H, s); **^{13}C** NMR (101 MHz, $CDCl_3$) δ 209.8, 169.5, 151.2, 141.1, 130.4, 126.2, 125.5, 125.0, 121.4, 112.4, 67.9, 56.1, 40.3, 33.9, 24.0; **HRMS** $[C_{15}H_{17}N_4O_3]^+$: 301.1301, found 301.1302; **IR** ν_{max} = 3246, 1634, 1602, 1508, 1467.

Synthesis of 5-((1-(3-chlorophenyl)-1*H*-1,2,3-triazol-4-yl)methyl)-5-methylpyrrolidine-2,4-dione (23)

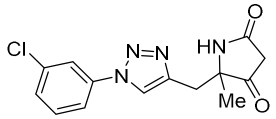

Following general procedure 2: **36e** (53.0 mg, 0.130 mmol), potassium *tert*-butoxide (21.0 mg, 0.187 mmol) and THF (1.4 mL) were refluxed for 2 hours. After work up, the crude intermediate was dissolved in acetonitrile (1 mL) and H_2O (0.1 mL) and refluxed for 3 hours. The crude product was purified by flash column chromatography (silica gel; EtOAc) to give the title compound (26.0 mg, 0.0853 mmol, 66%) as an off-white solid.

R_f = 0.15 (EtOAc); **^{1}H** NMR (400 MHz, $CDCl_3$) δ 7.86 (1H, s), 7.70 (1H, t, *J* = 1.8 Hz), 7.60 (1H, dt, *J* = 7.6, 1.7 Hz), 7.53 (1H, br s), 7.45 – 7.37 (2H, m), 3.21 (1H, d, *J* = 15.0 Hz), 3.13

(1H, d, J = 15.0 Hz), 3.08 (1H, d J = 22.2 Hz), 2.91 (1H, d, J = 22.2 Hz), 1.46 (3H, s); **^{13}C** NMR (101 MHz, $CDCl_3$) δ 209.5, 170.0, 142.8, 137.7, 135.7, 131.0, 129.0, 121.0, 120.7, 118.4, 67.8, 40.3, 33.9, 23.8; **HRMS** (ESI) calcd for $[C_{14}H_{14}N_4O_2{}^{35}Cl]^+$: 305.0805, found 305.0814; **IR** ν_{max} = 3136, 3096, 2924, 1762, 1693, 1594, 1491, 1464.

Synthesis of 5-methyl-5-((1-(3-(trifluoromethyl)phenyl)-1*H*-1,2,3-triazol-4-yl)methyl)pyrrolidine-2,4-dione (24)

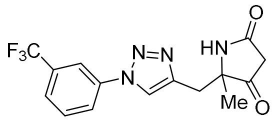

Following general procedure 2: **36f** (83.0 mg, 0.188 mmol), potassium *tert*-butoxide (32.0 mg, 0.285 mmol) and THF (2 mL) were refluxed for 2.5 hours. After work up, the crude intermediate was dissolved in acetonitrile (1 mL) and H_2O (0.1 mL) and refluxed for 3 hours. The crude product was purified by flash column chromatography (silica gel; heptane/EtOAc, gradient 1:4 to 0:1) to give the title compound (47.0 mg, 0.219 mmol, 74%) as an off-white solid.

R_f = 0.26 (EtOAc); **^{1}H** NMR (400 MHz, $CDCl_3$) δ 7.97 (1H, s), 7.95 – 7.91 (2H, m), 7.82 (1H, br s), 7.68 – 7.61 (2H, m), 3.24 (1H, d, J = 14.9 Hz), 3.16 (1H, d, J = 14.9 Hz), 3.06, (1H, d, J = 22.2 Hz), 2.88 (1H, d, J = 22.1 Hz), 1.47 (3H, s); **^{13}C** NMR (101 MHz, $CDCl_3$) δ 209.5, 170.2, 143.0, 137.2, 132.5 (q, J = 33.4 Hz), 130.7, 125.6 (q, J = 3.6 Hz), 123.5 (q, J = 273.0 Hz), 123.5, 121.0, 117.3 (q, J = 3.9 Hz), 67.8, 40.3, 33.9, 23.9; **^{19}F** (376 MHz, $CDCl_3$) δ -62.9; **HRMS** (ESI) calcd for $[C_{15}H_{14}N_4O_2F_3]^+$: 339.1069, found 339.1064; **IR** ν_{max} = 3139, 3104, 2941, 1766, 1697, 1498, 1480.

Synthesis of 5-((1-(3-methoxyphenyl)-1*H*-1,2,3-triazol-4-yl)methyl)-5-methylpyrrolidine-2,4-dione (25)

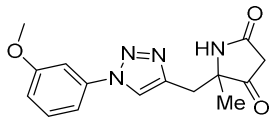

Following general procedure 2: **36g** (55.0 mg, 0.136 mmol), potassium *tert*-butoxide (23.0 mg, 0.205 mmol) and THF (1.5 mL) were refluxed for 2 hours. After work up, the crude intermediate was dissolved in acetonitrile (1 mL) and H_2O (0.1 mL) and refluxed for 3 hours. The crude product was purified by flash column chromatography (silica gel; heptane/EtOAc, gradient 1:4 to 0:1) to give the title compound (32.0 mg, 0.107 mmol, 78%) as a white film.

R_f = 0.17 (EtOAc); **^{1}H** NMR (400 MHz, $CDCl_3$) δ 7.83 (1H, s), 7.62 (1H, br s), 7.36 (1H, t, *J* = 8.2 Hz), 7.27 – 7.26 (1H, m), 7.19 (1H, dd, *J* = 7.9, 1.4 Hz), 6.93 (1H, dd, *J* = 8.3, 2.2 Hz), 3.86 (3H, s), 3.20 (1H, d, *J* = 14.9 Hz), 3.12 (1H, d, *J* = 14.9 Hz), 3.05 (1H, d, *J* = 22.2 Hz), 2.90 (1H, d, *J* = 22.2 Hz), 1.46 (3H, s); **^{13}C** NMR (101 MHz, $CDCl_3$) δ 209.7, 170.0, 160.7, 142.4, 137.9, 130.6, 121.1, 114.7, 112.3, 106.3, 67.8, 55.8, 40.3, 33.9, 23.9; **HRMS** (ESI) calcd for $[C_{15}H_{17}N_4O_3]^+$: 301.1301, found 301.1297; **IR** ν_{max} = 3128, 2976, 1769, 1698, 1608, 1594, 1503.

Synthesis of 5-((1-(4-chlorophenyl)-1*H*-1,2,3-triazol-4-yl)methyl)-5-methylpyrrolidine-2,4-dione (26)

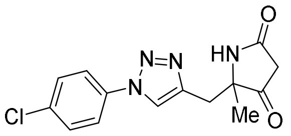

Following general procedure 2: **36i** (69.0 mg, 0.169 mmol), potassium *tert*-butoxide (28.0 mg, 0.249 mmol) and THF (1.7 mL) were refluxed for 2 hours. After work up, the crude intermediate was dissolved in acetonitrile (1 mL) and H_2O (0.1 mL) and refluxed for 2 hours. The crude product was purified by flash column chromatography (silica gel; EtOAc) to give the title compound (32.0 mg, 0.105 mmol, 62%) as an off-white solid.

R_f = 0.18 (EtOAc); **^{1}H** NMR (400 MHz, $CDCl_3$) δ 7.83 (1H, s), 7.62 (2H, d, *J* = 8.8 Hz), 7.53 (1H, br s), 7.46 (2H, d, *J* = 8.8 Hz), 3.21 (1H, d, *J* = 15.0 Hz), 3.13 (1H, d, *J* = 15.0 Hz), 3.06 (1H, d, *J* = 22.2 Hz), 2.89 (1H, d, *J* = 22.2 Hz), 1.46 (3H, s); **^{13}C** NMR (101 MHz, $CDCl_3$) δ 209.6, 169.9, 142.8, 135.3, 134.8, 130.1, 121.6, 120.9, 67.8, 40.3, 33.9, 23.9; **HRMS** (ESI) calcd for $[C_{14}H_{14}N_4O_2{}^{35}Cl]^+$: 305.0805, found 305.0811; **IR** ν_{max} = 3151, 3080, 2921, 2852, 1766, 1690, 1501, 1425.

Synthesis of 5-methyl-5-((1-(*p*-tolyl)-1*H*-1,2,3-triazol-4-yl)methyl)pyrrolidine-2,4-dione (27)

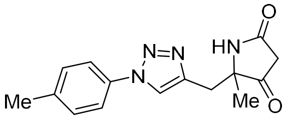

Following general procedure 2: **36h** (46.7 mg, 0.125 mmol), potassium *tert*-butoxide (20.0 mg, 0.178 mmol) and THF (1.5 mL) were refluxed for 2 hours. After work up, the crude intermediate was dissolved in acetonitrile (1 mL) and H_2O (0.1 mL) and refluxed for 3 hours. The crude

product was purified by flash column chromatography (silica gel; EtOAc) to give the title compound 25.0 mg, 0.0879 mmol, 70%) as an off-white solid.

R$_f$ = 0.17 (EtOAc); **^{1}H** NMR (400 MHz, $CDCl_3$) δ 7.79 (1H, s), 7.55 (2H, d, *J* = 8.4 Hz), 7.38 (1H, br s), 7.28 (2H, d, *J* = 8.3 Hz), 3.20 (1H, d, *J* = 15.0 Hz), 3.11 (1H, d, *J* = 15.0 Hz), 3.06 (1H, d, J = 22.2 Hz), 2.91 (1H, d, J = 22.2 Hz), 2.41 (3H, s), 1.45 (3H, s); **^{13}C** NMR (101 MHz, $CDCl_3$) δ 209.7, 169.8, 142.4, 139.2, 134.6, 130.4, 121.0, 120.4, 67.8, 40.3, 33.8, 23.9, 21.2; **HRMS** (ESI) calcd for $[C_{15}H_{17}N_4O_2]^+$: 285.1352, found 285.1359; **IR** ν_{max} = 3138, 2975, 2921, 1760, 1693, 1519.

Synthesis of 5-((1-(4-fluorophenyl)-1*H*-1,2,3-triazol-4-yl)methyl)-5-methylpyrrolidine-2,4-dione (28)

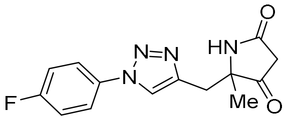

Following general procedure 2: **36j** (65.0 mg, 0.165 mmol), potassium *tert*-butoxide (28.0 mg, 0.250 mmol) and THF (1.7 mL) were refluxed for 2 hours. After work up, the crude intermediate was dissolved in acetonitrile (1 mL) and H_2O (0.1 mL) and refluxed for 3 hours. The crude product was purified by flash column chromatography (silica gel; EtOAc) to give the title compound (35.0 mg, 0.121 mmol, 73%) as an off-white solid.

R$_f$ = 0.14 (EtOAc); **^{1}H** NMR (400 MHz, $CDCl_3$) δ 7.81 (1H, s), 7.67 – 7.64 (2H, m), 7.59 (1H, br s), 7.20 – 7.16 (2H, m), 3.22 (1H, d, *J* = 15.0 Hz), 3.13 (1H, d, *J* = 15.0 Hz), 3.05 (1H, d, *J* = 22.2 Hz), 2.87 (1H, d, *J* = 22.2 Hz), 1.46 (3H, s); **^{13}C** NMR (101 MHz, $CDCl_3$) 209.6, 170.0, 162.6 (d, *J* = 249.4 Hz), 142.7, 133.2, 122. 5 (d, *J* = 8.6 Hz), 121.2, 116.9 (d, *J* = 23.0 Hz), 67.8, 40.4, 33.9, 23.9; **^{19}F** (376 MHz, $CDCl_3$) δ -111.8; **HRMS** (ESI) calcd for $[C_{14}H_{14}N_4O_2F]^+$: 289.1101, found 289.1107; **IR** ν_{max} = 3094, 2902, 1762, 1690, 1517.

Synthesis of methyl 2-(3-ethoxy-3-oxopropanamido)-2-methyl-3-(3-phenylisoxazol-5-yl)propanoate (37)

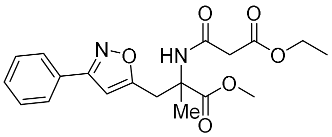

To a solution of **34a** (65.0 mg, 0.255 mmol) in *t*-BuOH (1.5 mL) and water (1.5 mL) was added *N*-hydroxybenzimidoyl chloride (36.0 mg, 0.231 mmol), copper (II) sulfate pentahydrate (13.7

mg, 0.0548 mmol), sodium ascorbate (15.3 mg, 0.0772 mmol) followed by potassium hydrogen carbonate (102 mg, 1.02 mmol). The reaction was stirred for 39 hours and then diluted with water and extracted with EtOAc (3 x 10 mL). The combined organic extracts were dried over Na_2SO_4 and concentrated *in vacuo*. The crude product was purified by flash column chromatography (silica gel; petroleum ether/EtOAc, gradient 2:3 to 3:2) to give the title compound (46.4 mg, 0.124 mmol, 49%) as a colourless oil.

R*f* = 0.18 (petroleum ether/EtOAc, 3:2); **^{1}H** NMR (400 MHz, $CDCl_3$) δ 7.78 (2H, m), 7.69 (1H, br s), 7.46 – 7.43 (3H, m), 6.38 (1H, s), 4.13 (2H, q, *J* = 7.2 Hz), 3.84 (3H, s), 3.81 (1H, d, *J* = 15.0 Hz), 3.58 (1H, d, *J* = 15.0 Hz), 3.30 (2H, s), 1.68 (3H, s), 1.22 (3H, t, *J* = 7.2 Hz); **^{13}C** NMR (101 MHz, $CDCl_3$) δ 173.4, 169.1, 168.9, 164.8, 162.4, 130.1, 129.1, 129.0, 126.9, 101.9, 61.9, 59.4, 53.4, 42.1, 32.8, 23.6, 14.1; **HRMS** (ESI) calcd for $[C_{19}H_{23}N_2O_6]^+$: 375.1551, found 375.1548. **IR** ν_{max} = 3303, 2978,1737, 1670, 1657, 1535, 1444.

Synthesis of 5-methyl-5-((3-phenylisoxazol-5-yl)methyl)pyrrolidine-2,4-dione (29)

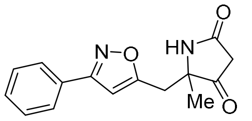

Following general procedure 2: **37** (60.0 mg, 0.160 mmol), potassium *tert*-butoxide (27.0 mg, 0.241 mmol) and THF (2 mL) were refluxed for 2 hours. After work up, the crude intermediate was dissolved in acetonitrile (2 mL) and H_2O (0.2 mL) and refluxed for 2.5 hours. The crude product was purified by flash column chromatography (silica gel; heptane/EtOAc, gradient 1:4 to 0:1) to give the title compound (26.0 mg, 0.0961 mmol, 38%) as a white solid.

R*f* = 0.16 (petroleum ether/EtOAc, 7:13); **^{1}H** NMR (400 MHz, $CDCl_3$) δ 7.77 – 7.75 (2H, m), 7.45 – 7.44 (3H, m), 6.89 (1H, br s), 6.42 (1H, s), 3.23 (1H, d, *J* = 15.1 Hz), 3.16 (1H, d, *J* = 15.1 Hz), 3.08 (1H, d, *J* = 22.3 Hz), 2.98 (1H, d, *J* = 22.3 Hz), 1.45 (1H, s); **^{13}C** NMR (101 MHz, $CDCl_3$) δ 208.2, 169.7, 167.5, 162.7, 130.4, 129.1, 128.6, 126.9, 102.3, 67.0, 39.8, 35.4, 24.0; **HRMS** (ESI) calcd for $[C_{15}H_{15}N_2O_3]^+$: 271.1083, found 271.1088; **IR** ν_{max} = 3088, 1770, 1698, 1690, 1607, 1470, 1443.

Synthesis of methyl 2-(3-ethoxy-3-oxopropanamido)-2-methyl-3-(3-phenylisoxazol-4-yl)propanoate (38)

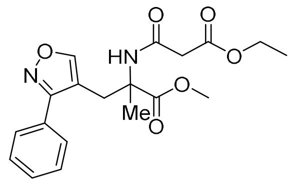

A solution of **34a** (65.0 mg, 0.255 mmol) in 1,2-dichloroethane (2.5 mL) was degassed for 15 minutes before *N*-hydroxybenzimidoyl chloride (36.0 mg, 0.231 mmol), triethylamine (39.0 µL, 0.281 mmol) and Cp*Ru(COD)Cl (9.70 mg, 0.0255 mmol) were added. The reaction was stirred at r.t for 42 hours. The reaction was concentrated *in vacuo* and purified by flash column chromatography (silica gel; heptane/EtOAc, gradient 1:9 to 7:13) to give the title compound (70.0 mg, 0.187 mmol, 73%) as a pale-yellow solid.

$\mathbf{R}_f$ = 0.18 (petroleum ether/EtOAc, 3:2); **^{1}H** NMR (400 MHz, $CDCl_3$) δ 8.33 (1H, s), 7.66 (1H, br s), 7.57 – 7.54 (2H, m), 7.49 – 7.46 (3H, m), 4.20 (2H, q, *J* = 7.2 Hz), 3.65 (1H, d, *J* = 15.2 Hz), 3.44 (3H, s), 3.28 (1H, d, *J* = 15.1 Hz), 3.05 (2H, s), 1.57 (3H, s), 1.30 (3H, t, *J* = 7.1 Hz); **^{13}C** NMR (101 MHz, $CDCl_3$) δ 173.7, 169.2, 164.6, 162.1, 158.0, 129.6, 129.2, 129.0, 128.8, 112.8, 61.9, 60.4, 52.9, 41.7, 28.1, 23.3, 14.2; **HRMS** (ESI) calcd for $[C_{19}H_{23}N_2O_6]^+$: 375.1556, found 375.1555; **IR** ν_{max} = 3308, 2985, 1733, 1672, 1655, 1531, 1446, 1118.

Synthesis of 5-methyl-5-((3-phenylisoxazol-4-yl)methyl)pyrrolidine-2,4-dione (30)

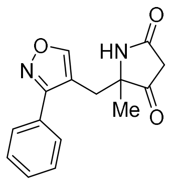

Following general procedure 2: **38** (52.0 mg, 0.139 mmol), potassium *tert*-butoxide (16.0 mg, 0.143 mmol) and THF (1.5 mL) were refluxed for 2 hours. After work up, the crude intermediate was dissolved in acetonitrile (1.5 mL) and H_2O (0.1 mL) and refluxed for 4 hours. The crude product was purified by flash column chromatography (silica gel; heptane/EtOAc, gradient 1:4 to 0:1) to give the title compound (21.0 mg, 0.0777 mmol, 56%) as a white solid.

$\mathbf{R}_f$ = 0.27 (EtOAc); **^{1}H** (400 MHz, $CDCl_3$) δ 8.31 (1H, s), 7.51 (5H, br s), 6.16 (1H, br s), 3.06 (1H, d, *J* = 15.0 Hz), 2.91 (1H, d, *J* = 22.2 Hz), 2.84 (1H, d, *J* = 15.0 Hz), 2.60 (1H, d, *J* = 22.2 Hz), 1.30 (3H, s); **^{13}C** (101 MHz, $CDCl_3$) δ; 209.0, 169.4, 162.5, 157.6, 130.1, 129.3, 129.3,

128.6, 112.0, 68.4, 40.3, 30.5, 24.1,**HRMS** (ESI) calcd for $[C_{15}H_{15}N_2O_3]^+$: 271.1083, found 271.1092; **IR** ν_{max} = 3259, 2932, 1645, 1551, 1444.

Synthesis of methyl 2-(3-ethoxy-3-oxopropanamido)-2-phenyl-3-(1-phenyl-1*H*-1,2,3-triazol-4-yl)propanoate (39)

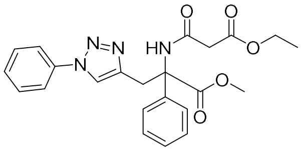

Following general procedure 1: **34b** (72.0 mg, 0.227 mmol), copper(II) sulfate pentahydrate (5.66 mg, 0.0227 mmol), sodium ascorbate (13.5 mg, 0.0681 mmol), azidobenzene (0.5M in TBME, 0.408 mL, 0.207 mmol), *t*-BuOH (1.5 mL) and water (1.5 mL) were used. The crude product was purified by flash column chromatography (silica gel; heptane/EtOAc, gradient 1:4 to 0:1) to give the title compound (52.0 mg, 0.119 mmol, 52 %) as a white solid.

$\mathbf{R_f}$ = 0.61 (Petroleum ether/EtOAc, 7:13); ^{1}**H** (400 MHz, $CDCl_3$) δ 8.07 (1H, br s), 7.84 (1H, s), 7.75 – 7.72 (2H, m), 7.53 – 7.48 (4H, m), 7.44 – 7.37 (3H, m), 7.34 – 7.30 (1H, m), 4.30 (1H, d, *J* = 14.3 Hz), 4.17 – 4.10 (3H, m), 3.80 (3H, s), 3.36 – 3.27 (2H, m), 1.23 (3H, t, *J* = 7.2 Hz); ^{13}**C** (101 MHz, $CDCl_3$) δ 172.4, 168.7, 164.1, 143.3, 138.7, 137.2, 129.8, 128.9, 128.6, 128.3, 126.0, 121.4, 120.3, 65.7, 61.9, 53.8, 42.9, 30.0, 14.1; **HRMS** (ESI) calcd for $[C_{23}H_{25}N_4O_5]^+$: 437.1825, found 437.1834; **IR** ν_{max} = 3246, 2961, 1739, 1651, 1598, 1521, 1504.

Synthesis of 5-phenyl-5-((1-phenyl-1*H*-1,2,3-triazol-4-yl)methyl)pyrrolidine-2,4-dione (33)

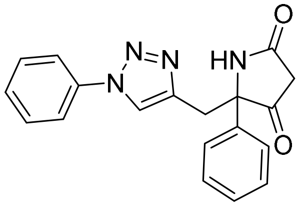

Following general procedure 2: **39** (43.0 mg, 0.0985 mmol), potassium *tert*-butoxide (17.0 mg, 0.151 mmol) and THF (1 mL) were refluxed for 2.5 hours. After work up, the crude intermediate was dissolved in acetonitrile (1 mL) and H_2O (0.1 mL) and refluxed for 3 hours. The crude product was purified by flash column chromatography (silica gel; heptane/EtOAc, gradient 1:4 to 0:1) to give the title compound (23.0 mg, 0.0692 mmol, 71%) as white film.

R_f = 0.49 (EtOAc); **^{1}H** NMR (400 MHz, $CDCl_3$) δ 7.76 (1H, br s), 7.57 – 7.51 (4H, m), 7.50 (1H, s), 7.47 – 7.44 (2H, m), 7.41 – 7.35 (3H, m), 7.33 – 7.29 (1H, m), 3.66 – 3.58 (2H, m), 3.11 (1H, d, *J* = 21.8 Hz), 2.98 (1H, d, *J* = 21.8 Hz); **^{13}C** NMR (101 MHz, $CDCl_3$) δ 205.8, 169.8, 142.5, 137.4, 136.8, 129.9, 129.1, 129.0, 128.7, 125.5, 120.8, 120.5, 73.1, 40.0, 34.2; **HRMS** (ESI) calcd for $[C_{19}H_{17}N_4O_2]^+$: 333.1346, found 333.1340; **IR** ν_{max} = 3256, 1773, 1698, 1667, 1500, 1412.

4.4 Hit 3: Activin A Fragment Synthetic Procedures

General Procedure 3:

2-Nitrobenzoyl chloride (1.2 eq.) and Et_3N (2.0 eq.) were added dropwise to a solution of the respective amine (1.0 eq.) in CH_2Cl_2 (0.5 M) cooled to 0 °C. After stirring for 30 minutes at 0 °C, the mixture was diluted CH_2Cl_2 and sat. aq. solution of NH_4Cl was added. The aqueous layer was extracted with CH_2Cl_2, the combined organic layers were washed with brine, dried over $MgSO_4$, filtered and concentrated *in vacuo*. The crude products were purified by flash column chromatography.

General Procedure 4:

To a solution of the respective amide (1.0 eq.) in DMF (0.1 M) was added NaH (60 % (w/w) in mineral oil, 1.5 eq.) at 0 °C. After stirring for 10 minutes at 0 °C, MeI (2.0 eq.) was added and the mixture stirred at 0 °C for additional 30 minutes. Subsequently, sat. aq. solution of NH_4Cl was added, the mixture extracted with EtOAc, the combined organic layers washed with brine, dried over $MgSO_4$, filtered and concentrated *in vacuo*. The crude product was purified by flash column chromatography.

General procedure 5:

Pd/C (10 % (w/w), 10 mol%) was added to a degassed solution of the respective amide (1.0 eq.) in MeOH (0.05 M) and the mixture was stirred under H_2 atmosphere (1 atm) for 2 hours. The mixture was filtered through celite and the filtrate concentrated *in vacuo*. The crude hydrogenation product was dissolved in DMF (0.1 M) and NaH (60 % (w/w) in mineral oil, 2.0 eq.) was added at 0 °C. After stirring the reaction at 0 °C for 10 min, the mixture was heated to 60 °C and stirred for 2 hours. Subsequently, the reaction was cooled to room temperature and quenched with sat. aq. solution of NH_4Cl. After extraction with EtOAc, the combined

organic layers were washed with brine, dried over $MgSO_4$, filtered and concentrated *in vacuo*. The crude product was purified by flash column chromatography.

General procedure 6:

Activated zinc powder (1.5 eq.) and ammonium formate (3.0 eq.) were added to a stirred solution of the respective amide (1.0 eq.) in methanol (0.05 M). The reaction was stirred at room temperature for 1 hour and subsequently filtered through a pad of Celite and eluted with CH_2Cl_2. The solvent was removed *in vacuo* and the crude dissolved in DMF (0.1 M). Sodium hydride (60 % (w/w) in mineral oil, 2.0 eq.) was added and the mixture was stirred at 60 °C for 2 hours. Subsequently, the mixture was diluted with H_2O and the aqueous layer was extracted with EtOAc. The combined organic layers were washed with brine, dried over $MgSO_4$, filtered and concentrated *in vacuo*. The crude product was purified by flash column chromatography.

Synthesis of methyl 2-methyl-2-(2-nitrobenzamido)pent-4-ynoate (53a)

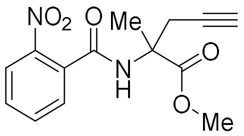

2-methyl-2-(2-nitrobenzamido)pent-4-ynoate was synthesized according to general procedure 3 from amine **15b** (140 mg, 0.99 mmol), purified by flash column chromatography (silica gel; petroleum ether/EtOAc, gradient 3:2 to 1:1) and isolated as a white solid (269 mg, 0.93 mmol, 94 %).

R*f* = 0.18 (petroleum ether/EtOAc, 3:2); **m.p.** = 122 – 123 °C; **^{1}H** NMR (400 MHz, $CDCl_3$) *δ* 8.08 (1H, dd, *J* = 8.4, 1.2 Hz), 7.68 (1H, td, *J* = 7.2, 6.5, 1.3 Hz), 7.61 – 7.57 (2H, m), 6.74 (1H, s), 3.85 (3H, s), 3.35 (1H, dd, *J* = 16.9, 2.6 Hz), 2.98 (1H, dd, *J* = 16.9, 2.6 Hz), 2.06 (1H, t, *J* = 2.6 Hz), 1.79 (3H, s); **^{13}C** NMR (101 MHz, $CDCl_3$) *δ* 173.3, 165.7, 146.5, 133.8, 132.9, 130.7, 129.0, 124.7, 79.5, 71.4, 60.1, 53.5, 26.6, 22.4; **HRMS** (ESI) calcd for $C_{14}H_{15}N_2O_5$ [$(M+H)^+$]: 291.0975, found 291.0969; **IR** ν_{max} = 1741, 1647, 1532, 1455, 1347, 1269, 1125.

Synthesis of 3-methyl-4-phenyl-3-propyl-3,4-dihydro-1*H*-benzo[*e*][1,4]diazepine-2,5-dione (41)

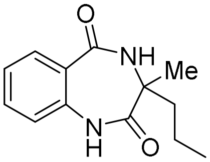

3-methyl-4-phenyl-3-propyl-3,4-dihydro-1*H*-benzo[*e*][1,4]diazepine-2,5-dione was synthesized according to general procedure 5 from amide **53a** (40 mg, 0.138 mmol), purified by flash column chromatography (silica gel; petroleum ether/EtOAc, gradient 3:2 to 1:1) and isolated as a white solid (32 mg, 0.138 mmol, quant.).

R*f* = 0.17 (petroleum ether/EtOAc, 1:1); **m.p.** = 191 – 192 °C; **^{1}H** NMR (400 MHz, DMSO-d_6) δ 10.38 (1H, s), 8.24 (1H, s), 7.77 (1H, dd, *J* = 7.8, 1.6 Hz), 7.50 (1H, ddd, *J* = 8.8, 7.3, 1.7 Hz), 7.17 (1H, td, *J* = 7.6, 1.2 Hz), 7.11 (1H, d, *J* = 8.0 Hz), 1.45 – 1.37 (2H, m), 1.27 (3H, s), 1.22 – 1.13 (2H, m), 0.66 (3H, t, *J* = 7.3 Hz); **^{13}C** NMR (101 MHz, DMSO-d_6) δ 173.2, 167.0, 136.6, 132.6, 130.2, 125.3, 123.4, 119.8, 57.3, 37.3, 22.7, 16.5, 14.0; **HRMS** (ESI) calcd for $C_{13}H_{17}O_2N_2$ [(M+H)$^+$]: 233.1285, found 233.1276; **IR** ν_{max} = 3177, 3058, 2932, 2873, 1675, 1646, 1609, 1586, 1489, 1445, 1406, 1379, 1368, 1357, 1310, 1290, 1263, 1236, 1194, 1164, 1152, 1082, 1054.

Synthesis of methyl 2-(*N*-ethyl-2-nitrobenzamido)-2-methylpent-4-ynoate (54a)

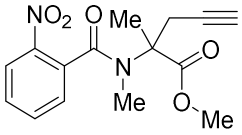

Methyl 2-(*N*-ethyl-2-nitrobenzamido)-2-methylpent-4-ynoate was synthesized according to general procedure 4 from amide **53a** (250 mg, 0.86 mmol), purified by flash column chromatography (silica gel; petroleum ether/EtOAc, 1:1) and isolated as a white solid (257 mg, 0.84 mmol, 98 %).

R*f* = 0.29 (petroleum ether/EtOAc, 1:1); **m.p.** = 114 – 115 °C; **^{1}H** NMR (400 MHz, $CDCl_3$) δ 8.18 (1H, dd, *J* = 8.3, 1.3 Hz), 7.71 (1H, td, *J* = 7.5, 1.2 Hz), 7.56 (1H, ddd, *J* = 8.8, 7.5, 1.5 Hz), 7.42 (1H, d, *J* = 7.4 Hz), 3.77 (3H, s), 3.49 (1H, dd, *J* = 17.3, 2.7 Hz), 3.01 (3H, s), 2.83 (1H, br s), 2.07 (1H, t, *J* = 2.7 Hz), 1.72 (3H, s); **^{13}C** NMR (101 MHz, $CDCl_3$) δ 173.3, 168.2, 145.1, 134.6, 133.4, 130.0, 128.1, 124.8, 80.4, 71.0, 62.0, 52.7, 34.5, 25.2, 21.6; **HRMS** (ESI) calcd for $C_{15}H_{17}N_2O_5$ [(M+H)$^+$]: 305.1137, found 305.1135; **IR** ν_{max} = 3238, 2955, 1736, 1620, 1575, 1536, 1491, 1444, 1401, 1376, 1344, 1314, 1256, 1234, 1197, 1144, 1108, 1052, 1036.

Synthesis of 3,4-dimethyl-3-propyl-3,4-dihydro-1*H*-benzo[*e*][1,4]diazepine-2,5-dione (40)

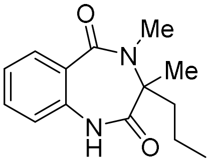

3,4-dimethyl-3-propyl-3,4-dihydro-1*H*-benzo[*e*][1,4]diazepine-2,5-dione was synthesized according to general procedure 5 from amide **54a** (30 mg, 0.10 mmol), purified by flash column chromatography (silica gel; petroleum ether/EtOAc, gradient 1:1 to 2:3) and isolated as a white solid (23 mg, 0.093 mmol, 93 %).

R*f* = 0.18 (petroleum ether/EtOAc, 1:1); **m.p.** = 149 – 150 °C; **^{1}H** NMR (400 MHz, $CDCl_3$) *δ* 8.51 (1H, s), 7.96 (1H, dd, *J* = 7.9, 1.6 Hz), 7.44 (1H, td, *J* = 7.7, 1.6 Hz), 7.21 (1H, ddd, *J* = 8.2, 7.4, 1.2 Hz), 6.92 (1H, dd, *J* = 8.1, 1.1 Hz), 3.24 (3H, s), 1.68 (3H, s), 1.63 – 1.57 (1H, m), 1.49 – 1.41 (1H, m), 1.26 – 1.16 (2H, m), 0.64 (3H, t, *J* = 7.3 Hz); **^{13}C** NMR (101 MHz, $CDCl_3$) *δ* 173.4, 168.3, 135.0, 132.5, 131.4, 127.8, 124.7, 119.2, 62.9, 38.4, 32.6, 23.1, 17.4, 14.1; **HRMS** (ESI) calcd for $C_{14}H_{19}O_2N_2$ [$(M+H)^+$]: 247.1441, found 247.1437; **IR** ν_{max} = 1671, 1607, 1486, 1447, 1416, 1376, 1291, 1263, 1222, 1196, 1162, 1121, 1079, 1036.

This compound was prepared as part of our previous library.[12]

Synthesis of 1,3,4-trimethyl-3-propyl-3,4-dihydro-1*H*-benzo[*e*][1,4]diazepine-2,5-dione (51)

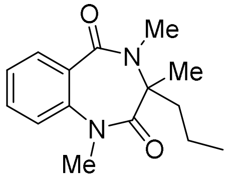

Sodium hydride (60 % (w/w) in mineral oil, 5.0 mg, 0.12 mmol, 1.5 eq.) was added to a solution of dihydrobenzodiazepinedione **40** (20 mg, 0.081 mmol, 1.0 eq.) in DMF (0.5 mL) cooled at 0 °C. After stirring for 10 minutes, methyl iodide (10 µL, 0.16 mmol, 2.0 eq.) was added and the mixture was stirred at the same temperature for 1 hour. Subsequently, the mixture was diluted with sat. aq. solution of NH_4Cl (5 mL) and the aqueous layer was extracted with EtOAc (3 x 5 mL). The organic layer was separated, dried over $MgSO_4$ and concentrated *in vacuo*. The crude product was purified by flash column chromatography (silica gel; petroleum ether/EtOAc, gradient 3:2) to afford the title compound (20 mg, 0.078 mmol, 96 %) as a colourless oil.

R_f = 0.40 (petroleum ether/EtOAc, 1:1); **^{1}H** NMR (400 MHz, $CDCl_3$) δ 7.81 (1H, dd, J = 7.8, 1.7 Hz), 7.49 (1H, ddd, J = 8.2, 7.4, 1.7 Hz), 7.27 – 7.23 (1H, m), 7.12 (1H, dd, J = 8.2, 1.1 Hz), 3.39 (3H, s), 3.22 (3H, s), 1.69 (3H, s), 1.32 – 1.18 (2H, m), 1.12 – 0.99 (2H, m), 0.52 (3H, t, J = 7.2 Hz); **^{13}C** NMR (101 MHz, $CDCl_3$) δ 172.0, 168.4, 140.0, 132.0, 130.6, 130.4, 125.4, 120.4, 63.7, 39.3, 37.1, 31.8, 24.4, 17.8, 14.0; **HRMS** (ESI) calcd for $C_{15}H_{21}N_2O_2$ [$(M+H)^+$]: 261.1603, found 261.1593; **IR** ν_{max} = 2960, 2931, 2874, 1663, 1626, 1459, 1416, 1376, 1316, 1280, 1260, 1229, 1153, 1128, 1001, 1036.

Synthesis of 3,4-dimethyl-3-propyl-1,2,3,4-tetrahydro-5*H*-benzo[*e*][1,4]diazepin-5-one (52)

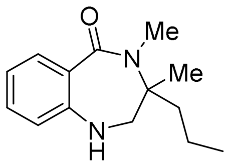

Sodium borohydride (9.1 mg, 0.24 mmol, 3.0 eq.) was added to a solution of **40** (20 mg, 0.081 mmol, 1.0 eq.) in dimethoxyethane (1 mL) at 0 °C. Trifluoroacetic acid (18 µL, 0.24 mmol, 3.0 eq.) was added dropwise and the mixture stirred for 10 minutes. Subsequently, the reaction was warmed to room temperature and stirred for 24 hours. Further sodium borohydride (18.2 mg, 0.48 mmol, 6.0 eq.) and trifluoroacetic acid (36 µL, 0.48 mmol, 6.0 eq.) were added and after stirring for a total of 48 hours, sat. aq. solution of NH_4Cl (5 mL) was added and the mixture extracted with EtOAc (3 x 10 mL). The organic layer was separated, dried over $MgSO_4$ and concentrated *in vacuo*. The crude product was purified by flash column chromatography (silica gel; petroleum ether/EtOAc, 1:1) to afford the title compound (12 mg, 0.052 mmol, 64 %) as a colourless oil and unreacted **40** (4.0 mg, 0.016 mmol, 20 %) as white solid.

R_f = 0.27 (EtOAc:Hexane, 1:1); **^{1}H** NMR (400 MHz, $CDCl_3$) δ (ppm) = 8.35 (1H, dd, J = 8.3, 1.6 Hz), 7.18 (1H, ddd, J = 8.0, 7.0, 1.6 Hz), 6.80 (1H, ddd, J = 8.2, 7.0, 1.2 Hz), 6.61 (1H, dd, J = 8.1, 1.2 Hz), 4.47 (1H, br s), 3.38 – 3.22 (2H, m), 3.04 (3H, s), 1.76 (1H, ddd, J = 14.1, 11.9, 4.8 Hz), 1.56 (1H, ddd, J = 14.0, 11.8, 5.2 Hz), 1.37 (3H, s), 1.34 – 1.26 (2H, m), 0.90 (3H, t, J = 7.3 Hz); **^{13}C** NMR (101 MHz, $CDCl_3$) δ 167.9, 149.1, 135.0, 131.6, 118.6, 118.3, 116.2, 63.2, 56.3, 40.7, 33.7, 24.9, 17.3, 14.7; **HRMS** (ESI) calcd for $C_{14}H_{21}N_2O$ [$(M+H)^+$]: 233.1654, found 233.1650; **IR** ν_{max} = 3314, 2957, 2936, 2872, 1611, 1590, 1555, 1491, 1419, 1350, 1308, 1273, 1226, 1158, 1138, 1115, 1082, 1027.

Synthesis of methyl 2-(*N*-ethyl-2-nitrobenzamido)-2-methylpent-4-ynoate (54b)

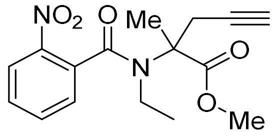

Sodium hydride (60 % (w/w) in mineral oil, 6.0 mg, 0.15 mmol, 1.5 eq.) and TBAI (18 mg, 0.050 mmol, 0.50 eq.) was added to a solution of **53a** (30 mg, 0.10 mmol, 1.0 eq.) in DMF (1 mL) cooled at 0 °C. After stirring for 10 minutes at 0 °C, bromoethane (22 µL, 0.30 mmol, 3.0 eq.) was added and the mixture was warmed to room temperature and stirred for 16 hours. Subsequently, the mixture was diluted with sat. aq. solution of NH_4Cl (5 mL) and the aqueous layer was extracted with EtOAc (3 x 10 mL). The organic layer was separated, dried over $MgSO_4$ and concentrated *in vacuo*. The crude product was purified by flash column chromatography (silica gel; petroleum ether/EtOAc, 3:2) to afford the title compound (20 mg, 0.063 mmol, 63 %) as a white solid.

R*f* = 0.27 (petroleum ether/EtOAc, 3:2); **m.p.** = 92 – 95 °C; **^{1}H** NMR (400 MHz, $CDCl_3$) δ 8.21 (1H, dd, *J* = 8.3, 1.2 Hz), 7.71 (1H, td, *J* = 7.5, 1.3 Hz), 7.57 (1H, td, *J* = 8.7, 7.5, 1.5 Hz), 7.45 (1H, dd, *J* = 7.6, 1.5 Hz), 3.79 (3H, s), 3.48 (2H, br d, *J* = 17.5 Hz), 3.29 (1H, br s), 2.90 (1H, br s), 2.08 (1H, t, *J* = 2.7 Hz), 1.76 (3H, s), 1.11 (3H, t, *J* = 7.2 Hz); **^{13}C** NMR (101 MHz, $CDCl_3$) δ 173.5, 168.6, 145.0, 134.2, 133.3, 130.0, 128.8, 124.9, 80.7, 71.2, 62.9, 52.8, 41.5, 25.4, 21.5, 16.5; **HRMS** (ESI) calcd for $C_{16}H_{18}N_2O_5Na$ [$(M+Na)^+$]: 341.1113, found 341.1113; **IR** ν_{max} = 1739, 1635, 1575, 1529, 1485, 1414, 1383, 1346, 1287, 1260, 1225, 1143, 1110, 1060.

Synthesis of 4-ethyl-3-methyl-3-propyl-3,4-dihydro-1*H*-benzo[*e*][1,4]diazepine-2,5-dione (42)

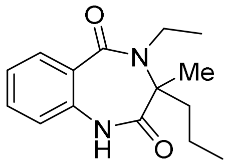

4-ethyl-3-methyl-3-propyl-3,4-dihydro-1*H*-benzo[*e*][1,4]diazepine-2,5-dione was synthesized according to general procedure 5 from amide **54b** (42 mg, 0.13 mmol), purified by flash column chromatography (silica gel; petroleum ether/EtOAc, gradient 3:2 to 1:1) and isolated as a white solid (32 mg, 0.12 mmol, 95 %).

R*f* = 0.37 (petroleum ether/EtOAc, 1:1); **m.p.** = 98 – 100 °C; **^{1}H** NMR (400 MHz, $CDCl_3$) δ 8.58 (1H, s), 7.91 (1H, dd, *J* = 7.9, 1.5 Hz), 7.42 (1H, td, *J* = 7.7, 1.6 Hz), 7.20 (1H, td, *J* = 7.7, 1.1 Hz), 6.91 (1H, dd, *J* = 8.0, 1.0 Hz), 3.91 (1H, dq, *J* = 14.2, 7.0 Hz), 3.66 (1H, dq, *J* = 14.1, 7.0

Hz), 1.70 (3H, s), 1.59 – 1.43 (2H, m), 1.25 – 1.15 (2H, m), 1.21 (3H, t, *J* = 7.0 Hz), 0.62 (3H, t, *J* = 7.3 Hz); **^{13}C** NMR (101 MHz, $CDCl_3$) *δ* 173.8, 167.8, 134.9, 132.3, 131.1, 128.5, 124.7, 119.0, 62.9, 40.3, 38.7, 22.7, 17.8, 15.2, 14.1; **HRMS** (ESI) calcd for $C_{15}H_{21}O_2N_2$ [$(M+H)^+$]: 261.1598, found 261.1592; **IR** ν_{max} = 3198, 3068, 2980, 2934, 1666, 1616, 1487, 1436, 1393, 1372, 1354, 1309, 1261, 1234, 1206, 1189, 1168, 1122, 1082, 1056.

Synthesis of methyl 2-methyl-2-(2-nitro-*N*-propylbenzamido)pent-4-ynoate (54c)

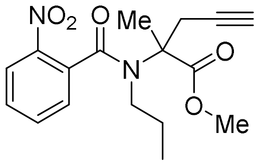

Sodium hydride (60 % (w/w) in mineral oil, 10 mg, 0.34 mmol, 1.5 eq.) was added to a solution of amide **53a** (50 mg, 0.17 mmol, 1.0 eq.) in DMF (1.0 mL) cooled at 0 °C. After stirring for 10 minutes at 0 °C, 1-iodopropane (33 µL, 0.34 mmol, 2.0 eq.) was added and the mixture was warmed to room temperature and stirred for 22 hours. Subsequently, the mixture was diluted with sat. aq. solution of NH_4Cl (5 mL) and the aqueous layer was extracted with EtOAc (3 x 10 mL). The organic layer was separated, dried over $MgSO_4$ and concentrated *in vacuo*. The crude product was purified by flash column chromatography (silica gel; petroleum ether/EtOAc, 3:2) to afford the title compound (16 mg, 0.048 mmol, 28%) as a colourless oil and unreacted **53a** (21 mg, 0.072 mmol, 42%) as a white solid.

R*f* = 0.21 (petroleum ether/EtOAc, 3:2); **^{1}H** NMR (400 MHz, $CDCl_3$) *δ* 8.20 (1H, dd, *J* = 8.3, 1.2 Hz), 7.71 (1H, td, *J* = 7.5, 1.2 Hz), 7.57 (1H, td, *J* = 8.0, 7.5, 1.5 Hz), 7.44 (1H, d, *J* = 7.6 Hz), 3.80 (3H, s), 3.52 – 3.24 (2H, m), 3.14 – 2.66 (2H, m), 2.08 (1H, t, *J* = 2.7 Hz), 1.76 (3H, s), 1.67 – 1.54 (2H, m), 0.61 (3H, t, *J* = 7.4 Hz); **^{13}C** NMR (101 MHz, $CDCl_3$) *δ* 173.4, 168.6, 145.0, 134.1, 133.2, 130.0, 128.7, 124.9, 80.6, 71.2, 62.8, 52.7, 48.9, 25.5, 24.5, 21.5, 11.4; **HRMS** (ESI) calcd for $C_{17}H_{20}N_2O_5Na$ [$(M+Na)^+$]: 355.1264, found 355.1274; **IR** ν_{max} = 3286, 2970, 1740, 1638, 1575, 1529, 1486, 1412, 1346, 1298, 1257, 1226, 1143, 1112, 1066.

Synthesis of 3-methyl-3,4-dipropyl-3,4-dihydro-1*H*-benzo[*e*][1,4]diazepine-2,5-dione (43)

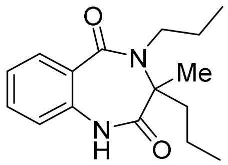

3-methyl-3,4-dipropyl-3,4-dihydro-1*H*-benzo[*e*][1,4]diazepine-2,5-dione was synthesized according to general procedure 5 from amide **54c** (30 mg, 0.09 mmol), purified by flash column chromatography (silica gel; petroleum ether/EtOAc, 3:2) and isolated as a colourless oil (17 mg, 0.06 mmol, 69%).

R*f* = 0.44 (petroleum ether/EtOAc, 1:1); **^{1}H** NMR (500 MHz, $CDCl_3$) δ 8.17 (1H, s), 7.92 (1H, dd, *J* = 7.9, 1.6 Hz), 7.42 (1H, td, *J* = 8.0, 1.6 Hz), 7.21 (1H, td, *J* = 7.7, 1.1 Hz), 6.88 (1H, dd, *J* = 8.1, 1.0 Hz), 3.94 – 3.85 (1H, m), 3.46 – 3.44 (1H, m), 1.69 (3H, s), 1.65 – 1.49 (4H, m), 1.25 – 1.14 (2H, m), 0.90 (3H, t, *J* = 7.4 Hz), 0.62 (3H, t, *J* = 7.3 Hz); **^{13}C** NMR (126 MHz, $CDCl_3$) δ 173.7, 167.9, 134.8, 132.3, 131.2, 128.5, 124.8, 119.0, 62.9, 47.1, 38.7, 23.1, 22.9, 17.7, 14.1, 11.5; **HRMS** (ESI) calcd for $C_{16}H_{23}N_2O_2$ [(M+H)$^+$]: 275.1760, found 275.1765; **IR** ν_{max} = 3225, 2962, 2933, 2874, 1672, 1607, 1487, 1437, 1393, 1365, 1317, 1260, 1228, 1192, 1161, 1124, 1087, 1043.

Synthesis of methyl 2-(*N*-butyl-2-nitrobenzamido)-2-methylpent-4-ynoate (54d)

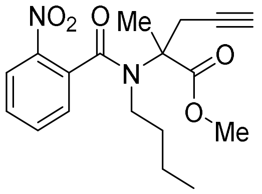

Sodium hydride (60 % (w/w) in mineral oil, 10 mg, 0.34 mmol, 1.5 eq.) was added to a solution of amide **53a** (50 mg, 0.17 mmol, 1.0 eq.) in DMF (1.0 mL) cooled at 0 °C. After stirring for 10 minutes at 0 °C, 1-iodobutane (39 µL, 0.34 mmol, 2.0 eq.) was added and the mixture was warmed to room temperature and stirred for 22 hours. Subsequently, the mixture was diluted with sat. aq. solution of NH_4Cl (5 mL) and the aqueous layer was extracted with EtOAc (3 x 10 mL). The organic layer was separated, dried over $MgSO_4$ and concentrated *in vacuo*. The crude product was purified by flash column chromatography (silica gel; petroleum ether/EtOAc, 3:2) to afford the title compound (22 mg, 0.064 mmol, 38%) as a colourless oil and unreacted starting material **53a** (14 mg, 0.048 mmol, 28% starting material recovered) as a white solid.

R*f* = 0.24 (petroleum ether/EtOAc, 3:2); **m.p.** = 81 – 84 °C; **^{1}H** NMR (400 MHz, $CDCl_3$) δ 8.22 (1H, dd, *J* = 8.2, 1.1 Hz), 7.71 (1H, td, *J* = 7.7, 7.6, 1.1 Hz), 7.57 (1H, td, *J* = 7.9, 1.4 Hz), 7.45 (1H, d, *J* = 7.5 Hz), 3.79 (3H, s), 3.50 – 2.84 (4H, m), 2.08 (1H, t, *J* = 2.7 Hz), 1.75 (3H, s), 1.62 – 1.49 (2H, m), 1.09 – 0.96 (2H, m), 0.67 (3H, t, *J* = 7.3 Hz); **^{13}C** NMR (101 MHz, $CDCl_3$) δ 173.5, 168.6, 145.1, 134.2, 133.2, 130.0, 128.8, 124.9, 80.6, 71.2, 62.8, 52.8, 46.9, 33.1, 26.6, 21.5, 20.2, 13.5; **HRMS** (ESI) calcd for $C_{18}H_{23}N_2O_5$ [(M+H)$^+$]: 347.1607, found 347.1617;

IR ν_{max} = 3265, 2960, 2873, 1750, 1632, 1576, 1530, 1486, 1455, 1419, 1373, 1345, 1302, 1249, 1220, 1144, 1112, 1068, 1038, 1000.

Synthesis of 4-butyl-3-methyl-3-propyl-3,4-dihydro-1*H*-benzo[*e*][1,4]diazepine-2,5-dione (44)

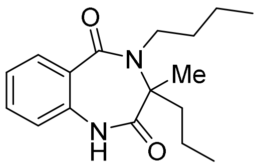

4-butyl-3-methyl-3-propyl-3,4-dihydro-1*H*-benzo[*e*][1,4]diazepine-2,5-dione was synthesized according to general procedure 5 from amide **54d** (20 mg, 0.06 mmol), purified by flash column chromatography (silica gel; petroleum ether/EtOAc, 3:2) and isolated as a colourless oil (15 mg, 0.05 mmol, 91%).

$\mathbf{R_f}$ = 0.40 (petroleum ether/EtOAc, 3:2); **^{1}H** NMR (400 MHz, $CDCl_3$) δ 8.41 (1H, br s), 7.92 (1H, dd, J = 7.9, 1.5 Hz), 7.42 (1H, td, J = 7.7, 1.6 Hz), 7.20 (1H, td, J = 7.7, 1.2 Hz), 6.89 (1H, dd, J = 8.9, 2.8 Hz), 4.01 – 3.83 (1H, m), 3.57 – 3.41 (1H, m), 1.69 (3H, s), 1.65 – 1.47 (4H, m), 1.32 (2H, h, J = 7.4 Hz), 1.22 – 1.15 (2H, m), 0.92 (3H, t, J = 7.3 Hz), 0.62 (3H, t, J = 7.3 Hz); **^{13}C** NMR (101 MHz, $CDCl_3$) δ 173.8, 167.8, 134.9, 132.3, 131.2, 128.5, 124.8, 119.0, 62.9, 45.4, 38.7, 32.0, 22.8, 20.4, 17.7, 14.1, 13.9; **HRMS** (ESI) calcd for $C_{17}H_{24}N_2O_2Na$ [$(M+Na)^+$]: 311.1735, found 311.1750; **IR** ν_{max} = 2960, 2934, 2873, 1673, 1611, 1488, 1437, 1394, 1368, 1312, 1262, 1227, 1191, 1125, 1043.

Synthesis of methyl 2-ethyl-2-(2-nitrobenzamido)pent-4-ynoate (53b)

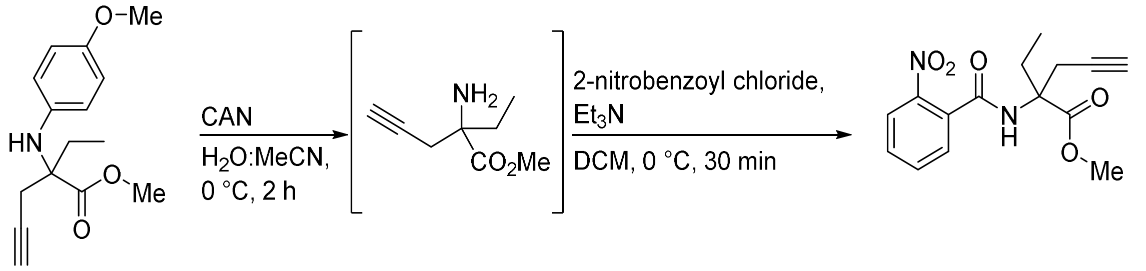

13c **15c** **53b**

Step 1: A solution of CAN (2.34 g, 4.00 mmol, 2.0 eq.) in H_2O (10 mL) was added dropwise to a stirred solution of **13c** (522 mg, 2.00 mmol, 1.0 eq.) in acetonitrile (10 mL) cooled to 0 °C. After stirring at 0 °C for 2 hours, the mixture was acidified to pH = 1 using 2 N HCl. The aqueous layer was washed with EtOAc (3 x 20 mL) and subsequently basified to pH = 11

using solid Na_2CO_3. The resulting emulsion was extracted with CH_2Cl_2 (3 x 50 mL), the combined organic layers washed with brine, dried over $MgSO_4$, filtered and concentrated *in vacuo* to afford **35** as a brown oil that was directly subjected to the second step.

Step 2: 2-Nitrobenzoyl chloride (0.32 mL, 2.40 mmol, 1.2 eq.) and Et_3N (0.56 mL, 4.00 mmol, 2.0 eq.) were added dropwise to a solution of the crude amine **15c** (1.0 eq.) in CH_2Cl_2 (4.0 mL) cooled to 0 °C. After stirring for 30 minutes at 0 °C, the mixture was diluted CH_2Cl_2 and sat. aq. solution of NH_4Cl (5 mL) was added. The aqueous layer was extracted with CH_2Cl_2, the combined organic layers were washed with brine, dried over $MgSO_4$, filtered and concentrated *in vacuo*. The crude product was purified by flash column chromatography (silica gel; petroleum ether/EtOAc, 3:2) and the title compound was isolated as a white solid (440 mg, 1.45 mmol, 73 % over two steps).

R*f* = 0.33 (petroleum ether/EtOAc, 1:1); **m.p.** = 115 – 116 °C; ^{1}**H** NMR (400 MHz, $CDCl_3$) *δ* 8.05 (1H, dd, *J* = 8.1, 1.2 Hz), 7.67 (1H, td, *J* = 7.4, 1.2 Hz), 7.60 – 7.56 (2H, m), 6.89 (1H, s), 3.85 (3H, s), 3.54 (1H, dd, *J* = 16.9, 2.7 Hz), 2.86 (1H, dd, *J* = 16.9, 2.6 Hz), 2.59 (1H, dq, *J* = 15.0, 7.5 Hz), 2.05 (1H, t, *J* = 2.6 Hz), 1.89 (1H, dq, *J* = 14.5, 7.4 Hz), 0.95 (3H, t, *J* = 7.4 Hz); ^{13}**C** NMR (101 MHz, $CDCl_3$) *δ* 172.8, 165.5, 146.6, 133.7, 133.1, 130.7, 129.0, 124.7, 79.5, 71.0, 65.2, 53.4, 28.1, 25.4, 8.7; **HRMS** (ESI) calcd for $C_{15}H_{17}N_2O_5$ [(M+H)$^+$]: 305.1137, found 305.1145; **IR** ν_{max} = 3305, 3259, 1731, 1638, 1547, 1531, 1444, 1347, 1314, 1283, 1246, 1180, 1134, 1092, 1014.

Synthesis of methyl 2-ethyl-2-(*N*-methyl-2-nitrobenzamido)pent-4-ynoate (54e)

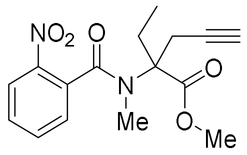

Methyl 2-ethyl-2-(*N*-methyl-2-nitrobenzamido)pent-4-ynoate was synthesized according to general procedure 4 from amide **53b** (100 mg, 0.33 mmol), purified by flash column chromatography (silica gel; petroleum ether/EtOAc, 1:1) and isolated as a white solid (89 mg, 0.28 mmol, 85 %).

R*f* = 0.24 (petroleum ether/EtOAc, 1:1); **m.p.** = 104 – 105 °C; ^{1}**H** NMR (400 MHz, $CDCl_3$) *δ* 8.18 (1H, dd, *J* = 8.3, 1.1 Hz), 7.71 (1H, td, *J* = 7.5, 1.2 Hz), 7.56 (1H, td, *J* = 8.0, 1.4 Hz), 7.40 (1H, d, *J* = 7.7 Hz), 3.76 (3H, s), 3.43 (1H, ddd, *J* = 17.7, 2.7, 1.5 Hz), 3.03 (3H, s), 2.96 (1H, br s), 2.39 (1H, br s), 2.03 (1H, t, *J* = 2.7 Hz), 2.02 – 1.95 (1H, m), 0.96 (3H, t, *J* = 7.4 Hz); ^{13}**C** NMR (101 MHz, $CDCl_3$) *δ* 171.9, 168.4, 145.1, 134.6, 133.5, 129.9, 128.1, 124.8, 80.3, 70.8,

65.5, 52.4, 34.5, 26.5, 21.2, 8.6; **HRMS** (ESI) calcd for $C_{16}H_{19}N_2O_5$ [(M+H)$^+$]: 319.1294, found 319.1292; **IR** ν_{max} = 3233, 2985, 2955, 2136, 1731, 1617, 1575, 1534, 1489, 1443, 1401, 1344, 1238, 1221, 1142, 1116, 1078, 1052, 1036, 1013.

Synthesis of 3-ethyl-4-methyl-3-propyl-3,4-dihydro-1*H*-benzo[*e*][1,4]diazepine-2,5-dione (45)

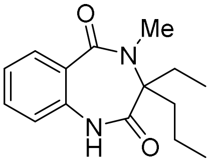

3-ethyl-4-methyl-3-propyl-3,4-dihydro-1*H*-benzo[*e*][1,4]diazepine-2,5-dione was synthesized according to general procedure 5 from amide **54e** (62 mg, 0.19 mmol), purified by flash column chromatography (silica gel; petroleum ether/EtOAc, 4:1) and isolated as a white solid (40 mg, 0.15 mmol, 79%).

R*f* = 0.48 (petroleum ether/EtOAc, 1:1); **m.p.** = 147 – 148 °C; ^{1}**H** NMR (400 MHz, $CDCl_3$) δ 8.85 (1H, s), 7.96 (1H, dd, *J* = 7.9, 1.6 Hz), 7.42 (1H, td, *J* = 7.6, 1.6 Hz), 7.19 (1H, td, *J* = 7.6, 1.1 Hz), 6.93 (1H, dd, *J* = 8.0, 1.1 Hz), 3.23 (3H, s), 2.02 – 1.76 (4H, m), 1.34 – 1.23 (2H, m), 0.88 (3H, t, *J* = 7.3 Hz), 0.83 (3H, t, *J* = 7.3 Hz); ^{13}**C** NMR (101 MHz, $CDCl_3$) δ 172.3, 168.8, 135.4, 132.4, 131.2, 128.2, 124.5, 119.1, 65.3, 33.3, 32.6, 24.7, 17.8, 14.4, 9.1; **HRMS** (ESI) calcd for $C_{15}H_{21}N_2O_2$ [(M+H)$^+$]: 261.1603, found 261.1602; **IR** ν_{max} = 3216, 3155, 2976, 2947, 2933, 2873, 1672, 1599, 1575, 1490, 1460, 1443, 1422, 1380, 1368, 1308, 1262, 1235, 1214, 1192, 1171, 1155, 1127, 1060, 1037.

Synthesis of methyl 2-(2-nitrobenzamido)-2-phenylpent-4-ynoate (53c)

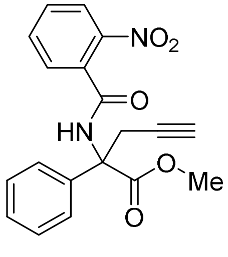

Methyl 2-(2-nitrobenzamido)-2-phenylpent-4-ynoate was synthesized according to general procedure 3 from methyl 2-amino-2-phenylpent-4-ynoate **15d** (66 mg, 0.32 mmol), purified by flash column chromatography (silica gel; petroleum ether/EtOAc, gradient 3:2 to 1:1) and isolated as a pale yellow solid (112 mg, 0.32 mmol, quant.).

R_f = 0.46 (petroleum ether/EtOAc, 1:1); **m.p.** = 123 – 124 °C; **^{1}H** NMR (400 MHz, $CDCl_3$) δ 8.06 (1H, d, J = 8.0 Hz), 7.70 – 7.66 (2H, m), 7.62 – 7.56 (3H, m), 7.45 – 7.38 (3H, m), 7.38 – 7.30 (1H, m), 3.92 (1H, dd, J = 16.6, 2.6 Hz), 3.78 (3H, s), 3.59 (1H,dd, J = 16.6, 2.6 Hz), 2.10 (1H, t, J = 2.6 Hz); **^{13}C** NMR (101 MHz, $CDCl_3$) δ 171.8, 165.0, 146.8, 137.0, 133.7, 132.7, 130.9, 129.2, 128.9, 128.7, 126.1, 124.8, 79.6, 71.7, 65.4, 53.9, 24.4; **HRMS** (ESI) calcd for $C_{19}H_{16}O_5N_2Na$ [(M+Na)$^+$]: 375.0951, found 375.0942. **IR** ν_{max} = 1743, 1670, 1575, 1529, 1475, 1448, 1437, 1348, 1313, 1229, 1084, 1045.

Synthesis of methyl 2-(*N*-methyl-2-nitrobenzamido)-2-phenylpent-4-ynoate (54f)

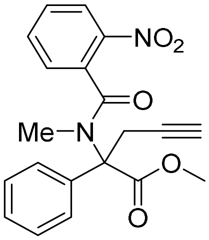

Methyl 2-(*N*-methyl-2-nitrobenzamido)-2-phenylpent-4-ynoate was synthesized according to general procedure 4 from amide **53c** (50 mg, 0.14 mmol), purified by flash column chromatography (silica gel; petroleum ether/EtOAc, 3:2) and isolated as a white solid (39 mg, 0.11 mmol, 79 %).

R_f = 0.39 (petroleum ether/EtOAc, 1:1); **m.p.** = 164 – 166 °C; **^{1}H** NMR (400 MHz, $CDCl_3$) δ 8.20 (1H, dd, J = 8.5, 1.2 Hz), 7.83 – 7.80 (2H, m), 7.75 (1H, td, J = 7.5, 1.2 Hz), 7.61 – 7.56 (2H, m), 7.46 – 7.36 (3H, m), 3.95 (1H, dd, J = 17.7, 2.7 Hz), 3.72 (3H, s), 3.57 (1H, d, J = 17.8 Hz), 2.74 (3H, s), 2.05 (1H, t, J = 2.7 Hz); **^{13}C** NMR (101 MHz, $CDCl_3$) δ 171.0, 169.5, 145.3, 134.6, 134.6, 133.4, 130.1, 129.1, 128.6 (2C), 128.2, 124.8, 80.7, 71.9, 68.8, 53.0, 37.6, 22.3; **HRMS** (ESI) calcd for $C_{20}H_{19}N_2O_5$ [(M+H)$^+$]: 367.1294, found 367.1309; **IR** ν_{max} = 1734, 1645, 1576, 1529, 1486, 1437, 1379, 1347, 1310, 1244, 1170, 1095, 1055, 1035, 1005.

Synthesis of 3-methyl-4-phenyl-3-propyl-3,4-dihydro-1H-benzo[e][1,4]diazepine-2,5-dione (46)

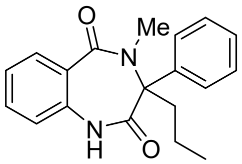

3-methyl-4-phenyl-3-propyl-3,4-dihydro-1*H*-benzo[*e*][1,4]diazepine-2,5-dione was synthesized according to general procedure 5 from amide **54f** (38 mg, 0.10 mmol), purified by flash column chromatography (silica gel; petroleum ether/EtOAc, gradient 3:2 to 1:1) and isolated as a white solid (17 mg, 0.055 mmol, 55%).

R*f* = 0.33 (petroleum ether/EtOAc, 1:1); **m.p.** = 158 – 186 °C; **^{1}H** NMR (400 MHz, $CDCl_3$) *δ* 8.51 – 8.34 (1H, m), 7.46 (1H, dd, *J* = 7.8, 1.6 Hz), 7.10 (1H, td, *J* = 7.7, 1.6 Hz), 7.01 – 6.92 (5H, m), 6.83 (1H, td, *J* = 7.6, 1.0 Hz), 6.64 (1H, dd, *J* = 8.1, 4.3 Hz), 3.45 (3H, s), 2.38 (1H, ddd, *J* = 13.3, 11.1, 4.4 Hz), 2.08 (1H, ddd, *J* = 13.1, 11.7, 3.8 Hz), 1.15 – 1.04 (1H, m), 0.82 (3H, t, *J* = 6.6 Hz), 0.78 – 0.70 (1H, m); **^{13}C** NMR (101 MHz, $CDCl_3$) *δ* 171.8, 169.8, 139.6, 134.5, 131.7, 130.8, 129.6, 127.9, 127.3, 125.4, 124.5, 119.1, 68.7, 40.6, 32.0, 18.6, 14.3; **HRMS** (ESI) *m/z* for $C_{19}H_{21}O_2N_2$ [$(M+H)^+$]: calcd. 309.1598, found 309.1589; **IR** ν_{max} = 3225, 3166, 2964, 2926, 1681, 1606, 1583, 1486, 1453, 1440, 1419, 1383, 1374, 1358, 1310, 1260, 1222, 1177, 1160, 1107, 1086, 1060, 1033.

Synthesis of methyl 2-(2-azidobenzamido)-2-methylpent-4-ynoate (55)

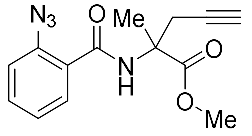

2-azidobenzoic acid (653 mg, 4.00 mmol, 1.6 eq.) was dissolved in $SOCl_2$ (2.9 mL, 40 mmol, 16 eq.) and refluxed at 85 °C for 3 hours. The mixture was concentrated *in vacuo* and the residue redissolved in CH_2Cl_2 (4 mL) and concentrated again *in vacuo* to remove residual $SOCl_2$. This procedure was repeated two additional times and the crude 2-azidobenzoylchloride **60** subsequently dissolved in CH_2Cl_2 (4 mL) and added dropwise to a solution of amine **15b** (353 mg, 2.50 mmol, 1.0 eq) and Et_3N (0.52 mL, 3.75 mmol, 1.5 eq.) in CH_2Cl_2 (6 mL) at 0 °C. The reaction was stirred at 0 °C for 45 minutes and subsequently quenched with sat. aq. solution of NH_4Cl (10 mL). The mixture was extracted with CH_2Cl_2 (3 x 20 mL), washed with brine, dried over $MgSO_4$, filtered and concentrated *in vacuo*. The crude product was purified by flash column chromatography (silica gel; petroleum ether/EtOAc, 4:1) to afford the title compound (587 mg, 3.05 mmol, 82 %) as a yellow solid.

R*f* = 0.21 (petroleum ether/EtOAc, 4:1); **m.p.** = 106 – 107 °C; **^{1}H** NMR (400 MHz, $CDCl_3$) *δ* 8.29 (1H, s), 8.13 (1H, dd, *J* = 7.9, 1.7 Hz), 7.51 (1H, td, *J* = 7.8, 1.7 Hz), 7.25 – 7.20 (2H, m), 3.79 (3H, s), 3.08 (2H, d, *J* = 2.7 Hz), 2.03 (1H, t, *J* = 2.6 Hz), 1.72 (3H, s); **^{13}C** NMR (101 MHz, $CDCl_3$) *δ* 173.4, 163.8, 137.5, 132.8, 132.4, 125.3, 124.6, 118.6, 79.7, 71.3, 59.3, 53.1, 26.8, 23.0; **HRMS** (ESI) calcd for $C_{14}H_{15}N_4O_3$ [$(M+H)^+$]: 287.1144, found 287.1138; **IR** ν_{max} =

3381, 3237, 2131, 1736, 1647, 1598, 1574, 1529, 1480, 1445, 1422, 1377, 1305, 1273, 1255, 1194, 1164, 1122.

Synthesis of 3,4-dimethyl-3-(prop-2-yn-1-yl)-3,4-dihydro-1*H*-benzo[*e*][1,4]diazepine-2,5-dione (47)

Synthesis of 3-(but-2-yn-1-yl)-3,4-dimethyl-3,4-dihydro-1*H*-benzo[e][1,4]diazepine-2,5-dione (48)

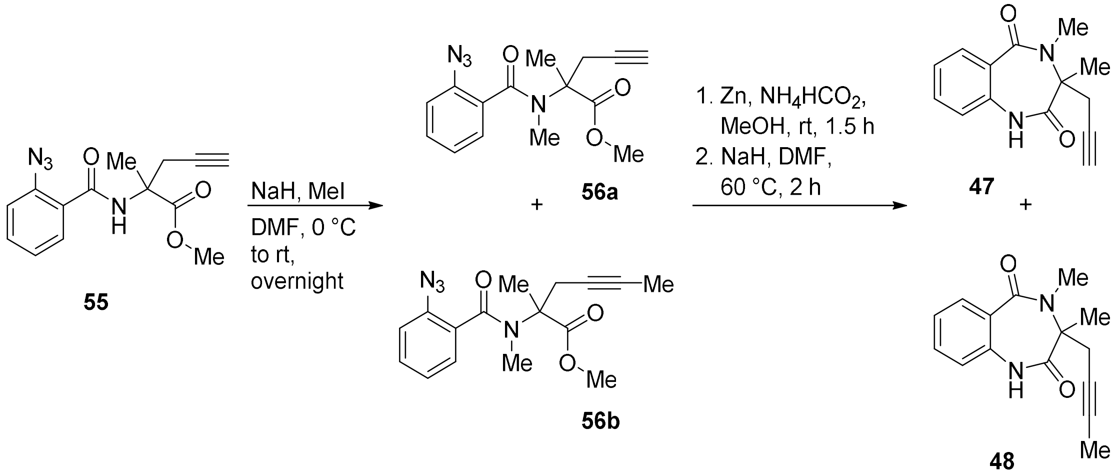

Step 1: Sodium hydride (60 % (w/w) in mineral oil, 160 mg, 4.00 mmol, 2.0 eq.) was added to a solution of amide **55** (567 mg, 2.0 mmol, 1.0 eq.) in DMF (4 mL) cooled at 0 °C. After stirring for 10 min at 0 °C, iodomethane (0.25 mL, 4.0 mmol, 2.0 eq.) was added and the mixture was stirred overnight. Subsequently, the mixture was diluted with sat. aq. solution of NH_4Cl (5 mL) and the aqueous layer was extracted with EtOAc (3 x 20 mL). The organic layer was separated, dried over $MgSO_4$ and concentrated *in vacuo*. The crude product was purified by flash column chromatography (silica gel; petroleum ether/EtOAc, gradient 7:3 to 3:2) to afford an inseparable mixture of **56a** and **56b** (257 mg, 0.86 mmol, 43%) as a white solid (**56a**:**56b** = 2.6:1:0, determined by ^{1}H NMR).

Step 2: The product mixture from step 1 was directly submitted to the reduction and cyclization reactions. 3,4-dimethyl-3-(prop-2-yn-1-yl)-3,4-dihydro-1*H*-benzo[*e*][1,4]diazepine-2,5-dione **47** and 3-(but-2-yn-1-yl)-3,4-dimethyl-3,4-dihydro-1*H*-benzo[*e*][1,4]diazepine-2,5-dione **48** were synthesized according to general procedure 6 from the mixture of **56a** and **56b** (187 mg, 0.62 mmol), purified by flash column chromatography (silica gel; EtOAc:PE 40-60, 1:1) and isolated as a white solid (88 mg, 0.37 mmol, 60 %, combined yield). 60 mg of the product mixture was subjected to reverse phase column chromatography using a *Combi Flash* system to isolate the title compounds.

47:

R$_f$ = 0.25 (petroleum ether/EtOAc, 1:1); **m.p.** = 196 – 198 °C; **^{1}H** NMR (400 MHz, DMSO-*d$_6$*) δ 10.65 (1H, s), 7.76 (1H, dd, J = 7.9, 1.6 Hz), 7.50 (1H, td, J = 7.3, 1.7 Hz), 7.20 (1H, td, J = 7.6, 1.2 Hz), 7.10 (1H, dd, J = 8.2, 1.1 Hz), 3.16 (3H, s), 2.97 (1H, t, J = 2.5 Hz), 2.55 (2H, br s), 1.65 (3H, s); **^{13}C** NMR (101 MHz, DMSO-*d$_6$*) δ 169.8, 166.9, 135.5, 132.4, 130.8, 127.3, 123.8, 119.5, 95.9, 74.5, 61.1, 31.9, 26.4, 22.7; **HRMS** (ESI) calcd for $C_{14}H_{15}N_2O_2$ [(M+H)$^+$]: 243.1128, found 243.1120; **IR** ν_{max} = 3248, 3197, 3068, 2989, 2926, 1660, 1610, 1585, 1505, 1487, 1448, 1436, 1416, 1374, 1316, 1271, 1239, 1220, 1195, 1158, 1137, 1125, 1094, 1052, 1030, 1007.

48:

R$_f$ = 0.25 (petroleum ether/EtOAc, 1:1); **m.p.** = 202 – 203 °C**;** **^{1}H** NMR (400 MHz, DMSO-*d$_6$*) δ 10.61 (1H, s), 7.75 (1H, dd, J = 7.8, 1.6 Hz), 7.49 (1H, td, J = 7.7, 1.7 Hz), 7.19 (1H, td, J = 7.5, 1.2 Hz), 7.09 (1H, dd, J = 8.1, 1.1 Hz), 3.15 (3H, s), 2.43 (2H, br s), 1.71 (3H, s), 1.64 (3H, br s); **^{13}C** NMR (101 MHz, DMSO-*d$_6$*) δ 168.3, 167.4, 135.5, 132.3, 130.7, 126.6, 123.7, 119.5, 79.3, 73.2, 61.3, 31.8, 26.0, 22.5, 3.1; **HRMS** (ESI) calcd for $C_{15}H_{17}N_2O_2$ [(M+H)$^+$]: 257.1285, found 257.1286; **IR** ν_{max} = 3199, 3067, 2926, 1663, 1620, 1588, 1488, 1439, 1415, 1375, 1273, 1242, 1223, 1199, 1135, 1123, 1095, 1059, 1029.

Synthesis of 3-butyl-3,4-dimethyl-3,4-dihydro-1*H*-benzo[*e*][1,4]diazepine-2,5-dione (49)

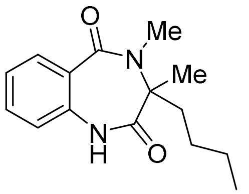

Pd/C (10 % (w/w), 4.2 mg, 0.0035 mmol, 10 mol%) was added to a degassed solution of alkyne **48** (9.0 mg, 0.035 mmol, 1.0 eq.) in MeOH:CH_2Cl_2 (1:1, 1 mL) and the reaction was stirred under H_2 atmosphere (1 atm) for 2 hours. The mixture was filtered through celite and the filtrate concentrated *in vacuo*. The crude product was purified by flash column chromatography (silica gel; hexane/EtOAc, 1:1) to afford the title compound (9.0 mg, 0.035 mmol, 99%) as a white amorphous solid.

R$_f$ = 0.24 (petroleum ether/EtOAc, 1:1); **^{1}H** NMR (400 MHz, $CDCl_3$) δ 8.21 (1H, s), 7.97 (1H, dd, J = 7.9, 1.5 Hz), 7.44 (1H, td, J = 7.7, 1.7 Hz), 7.21 (1H, t, J = 7.4 Hz), 6.90 (1H, d, J = 8.0 Hz), 3.24 (3H, s), 1.68 (3H, s), 1.63 – 1.57 (1H, m), 1.47 (1H, dt, J = 14.6, 8.0 Hz), 1.16 (2H, p, J = 7.3 Hz), 1.01 (2H, h, J = 6.9 Hz), 0.65 (3H, t, J = 7.3 Hz); **^{13}C** NMR (126 MHz, $CDCl_3$) δ

173.3, 168.3, 134.9, 132.5, 131.5, 127.8, 124.8, 119.1, 62.9, 35.9, 32.6, 26.0, 23.1, 22.5, 13.7; **HRMS** (ESI) calcd for $C_{15}H_{21}N_2O_2$ [(M+H)$^+$]: 261.1603, found 261.1608; **IR** ν_{max} = 3224, 3165, 2957, 2925, 1683, 1600, 1579, 1485, 1437, 1383, 1352, 1301, 1260, 1229, 1143, 1126, 1085, 1040.

Synthesis of methyl *N*-methyl-*N*-(2-nitrobenzoyl)glycinate (57)

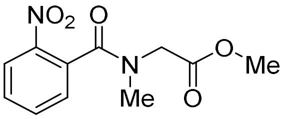

Methyl *N*-methyl-*N*-(2-nitrobenzoyl)glycinate was synthesized according to general procedure 3 from sarcosine methylester hydrochloride (419 mg, 3.00 mmol), purified by flash column chromatography (silica gel; petroleum ether/EtOAc, 2:3) and isolated as a brown oil (407 mg, 1.62 mmol, 54%).

R*f* = 0.19 (petroleum ether/EtOAc, 1:1); The product was obtained as a mixture of rotamers. High-temperature NMR was performed with measurements at room temperature, 100 °C and 120 °C to confirm. **^{1}H** NMR (500 MHz, DMSO-d$_6$, 120 °C) δ 8.16 (1H, d, *J* = 8.2 Hz), 7.84 (1H, br s), 7.71 (1H, t, *J* = 7.7 Hz), 7.48 (1H, br s), 4.13 (2H, br d, *J* = 154.5 Hz), 3.74 (3H, br s), 2.99 (3H, br d, *J* = 96.6 Hz); **^{13}C** NMR (126 MHz, DMSO-d$_6$, 120 °C) δ 168.3, 166.6, 145.0, 133.8, 131.4, 129.7, 127.4, 123.7, 51.1, 45.0, 36.6; **HRMS** (ESI) calcd for $C_{11}H_{13}N_2O_5$ [(M+H)$^+$]: 253.0819, found 253.0815; **IR** ν_{max} = 1746, 1643, 1576, 1529, 1494, 1439, 1403, 1347, 1305, 1210, 1179, 1097, 1066, 1038, 1014.

Synthesis of 4-methyl-3,4-dihydro-1*H*-benzo[*e*][1,4]diazepine-2,5-dione (50)

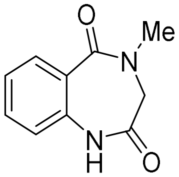

4-methyl-3,4-dihydro-1*H*-benzo[*e*][1,4]diazepine-2,5-dione was synthesized according to general procedure 5 from amide **57** (50 mg, 0.20 mmol), purified by flash column chromatography (silica gel; Et_2O) and isolated as a white solid (28 mg, 0.15 mmol, 75%).

R*f* = 0.23 (Et_2O); **m.p.** = 222 – 224 °C; **^{1}H** NMR (400 MHz, DMSO-*d$_6$*) δ 10.45 (1H, s), 7.74 (1H, dd, *J* = 7.9, 1.6 Hz), 7.49 (1H, td, *J* = 8.7, 1.6 Hz), 7.21 (1H, td, *J* = 7.6, 1.2 Hz), 7.10 (1H, dd, *J* = 8.2, 1.1 Hz), 3.84 (2H, s), 3.12 (3H, s); **^{13}C** NMR (101 MHz, DMSO-*d$_6$*) δ 169.8, 166.5,

137.0, 132.0, 130.9, 126.2, 123.9, 120.7, 52.2, 35.9; **HRMS** (ESI) calcd for $C_{10}H_{11}N_2O_2$ [(M+H)$^+$]: 191.0821, found 191.0822; **IR** ν_{max} = 3206, 3149, 3019, 1735, 1629, 1579, 1536, 1480, 1434, 1398, 1378, 1348, 1323, 1270, 1246, 1219, 1151, 1111, 1036.

Asymmetric *R*

Synthesis of (*R*)-3-methyl-5-phenyl-5,6-dihydro-2*H*-1,4-oxazin-2-one ((*R*)-61)

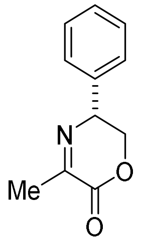

To a stirred solution of (*R*)-phenylglycinol (1.23 g, 9.00 mmol, 1.0 eq.) in trifluoroethanol (27 mL) in a microwave vial was added **12b** (0.90 mL, 9.90 mmol, 1.1 eq.) and molecular sieves (4 Å, 4.50 g). The reaction was irradiated to 120 °C for 50 minutes and subsequently combined and filtered through celite. The filtrate was concentrated *in vacuo* and the crude product purified by flash column chromatography (silica gel; EtOAc:PE 40-60, 2:8) to afford the title compound (844 mg, 4.46 mmol, 50 %) as a white solid.

R*f* = 0.44 (EtOAc:PE 40-60, 2:3); **m.p.** = 63 – 64 °C; **[α]$^{25}_{D}$** = – 227.9 ° ($CHCl_3$, 25 °C); **^{1}H** NMR (400 MHz, $CDCl_3$) δ 7.43 – 7.33 (5H, m), 4.84 (1H, ddq, *J* = 11.5, 4.9, 2.5 Hz), 4.56 (1H, dd, *J* = 11.6, 4.5 Hz), 4.26 (1H, t, *J* = 11.1 Hz), 2.41 (3H, d, *J* = 2.5 Hz); **^{13}C** NMR (101 MHz, $CDCl_3$) δ 160.4, 155.6, 136.9, 129.1, 128.4, 127.2, 71.6, 59.9, 21.9; **HRMS** (ESI) calcd for $C_{11}H_{12}NO_2$ [(M+H)$^+$]: 190.0863, found 190.0860; **IR** ν_{max} = 1721, 1641, 1494, 1468, 1454, 1404, 1373, 1316, 1272, 1145, 1087, 1067, 1022.

Synthesis of (3*R*,5*R*)-3-methyl-5-phenyl-3-(prop-2-yn-1-yl)morpholin-2-one ((*R*)-62)

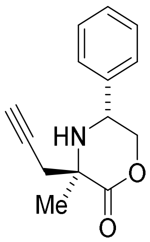

Propargyl bromide (0.70 mL, 6.47 mmol, 1.5 eq.) and activated zinc powder (423 mg, 6.47 mmol, 1.5 eq.) were added to a solution of **(*R*)-61** (815 mg, 4.31 mmol, 1.0 eq.) in DMF

(21 mL) at 0 °C. The reaction was stirred at 0 °C for 1 hour and subsequently quenched with sat. aq. solution of NH_4Cl (20 mL). After extraction with EtOAc (3 x 50 mL), the combined organic layers were washed with brine, dried over $MgSO_4$, filtered and concentrated *in vacuo*. The crude product (alkyne:allene = 3.9:1.0, determined by ^{1}H NMR) was purified by flash column chromatography (silica gel; petroleum ether/EtOAc, 9:1) to afford the title compound (857 mg, 3.74 mmol, 85 %, combined yield with inconsequential allene) as a white solid.

R$_f$ = 0.28 (petroleum ether/EtOAc, 4:1); **m.p.** = 87 – 89 °C; **$[\alpha]^{25}_D$** = + 35.1 ° ($CHCl_3$, 25 °C); **^{1}H** NMR (400 MHz, $CDCl_3$) δ 7.46 – 7.42 (2H, m), 7.42 – 7.33 (3H, m), 4.36 – 4.29 (3H, m), 3.10 (1H, dd, J = 16.9, 2.6 Hz), 2.63 (1H, dd, J = 16.9, 2.6 Hz), 2.35 (1H, s), 2.09 (1H, t, J = 2.6 Hz), 1.57 (3H, s); **^{13}C** NMR (101 MHz, $CDCl_3$) δ 172.2, 137.7, 129.0, 128.8, 127.4, 78.5, 75.4, 73.0, 59.0, 53.2, 28.7, 26.7; **HRMS** (ESI) calcd for $C_{14}H_{16}NO_2$ [(M+H)$^+$]: 230.1181, found 230.1185; **IR** ν_{max} = 3239, 1730, 1643, 1455, 1430, 1407, 1371, 1357, 1325, 1302, 1280, 1269, 1216, 1159, 1145, 1121, 1100, 1084, 1055, 1023.

Synthesis of methyl (*R*)-2-(((*R*)-2-hydroxy-1-phenylethyl)amino)-2-methylpent-4-ynoate ((*R*)-63)

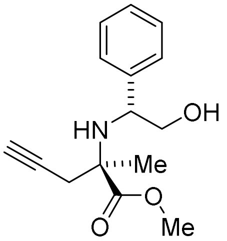

$SOCl_2$ (182 µL, 2.50 mmol, 2.0 eq.) was added to a solution of **(*R*)-62** (287 mg, 1.25 mmol, 1.0 eq.) in MeOH (12.5 mL). The reaction was stirred for 2 hours and subsequently concentrated *in vacuo*. The residue was redissolved in EtOAc (10 mL) and sat. aq. solution of $NaHCO_3$ (10 mL) and stirred for 20 minutes. After extraction with EtOAc (3 x 20 mL), the combined organic layers were washed with brine, dried over $MgSO_4$, filtered and concentrated *in vacuo*. The crude product was purified by flash column chromatography (silica gel; petroleum ether/EtOAc, 7:3) to afford the title compound (321 mg, 1.23 mmol, 98%) as a colourless oil.

R$_f$ = 0.09 (petroleum ether/EtOAc, 4:1); **$[\alpha]^{25}_D$** = – 57.1 ° ($CHCl_3$, 25 °C); **^{1}H** NMR (400 MHz, $CDCl_3$) δ 7.33 – 7.29 (2H, m), 7.26 – 7.22 (3H, m), 3.83 (1H,dd, J = 9.2, 4.7 Hz), 3.56 (1H, dd, J = 10.7, 4.7 Hz), 3.42 (1H, dd, J = 10.8, 9.3 Hz), 3.37 (3H, s), 2.99 (1H, br s), 2.55 – 2.44 (2H, m), 2.30 (1H, br s), 1.99 (1H, t, J = 2.6 Hz), 1.40 (3H, s); **^{13}C** NMR (101 MHz, $CDCl_3$) δ 174.9, 141.1, 128.6, 127.7, 127.2, 79.9, 71.4, 67.1, 60.9, 59.8, 52.1, 30.1, 22.6; **HRMS** (ESI)

calcd for $C_{15}H_{20}NO_3$ [(M+H)$^+$]: 262.1443, found 262.1446; **IR** ν_{max} = 3288, 2950, 2358, 2344, 1728, 1453, 1379, 1208, 1112, 1063, 1027.

Synthesis of methyl (*R*)-2-amino-2-methylpent-4-ynoate ((*R*)-15b)

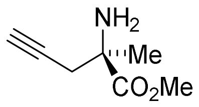

$Pb(OAc)_4$ (678 mg, 1.53 mmol, 1.4 eq.) was added to a solution of **(*R*)-63** (285 mg, 1.09 mmol, 1.0 eq.) in CH_2Cl_2:MeOH (2:1, 9 mL) at 0 °C. The reaction was stirred for 1 hour at 0 °C and subsequently 2M HCl (20 mL) was added. The mixture was warmed to room temperature and stirred for additional 2 hours. After filtering through a pad of SiO_2 and eluting with MeOH, the organic solvents were removed *in vacuo*. The aqueous residue was washed with EtOAc (3 x 20 mL) and basified to pH = 11 with solid Na_2CO_3. The resulting suspension was extracted with EtOAc (3 x 50 mL), the combined organic layers washed with brine, dried over $MgSO_4$, filtered and concentrated *in vacuo* to afford the title compound (112 mg, 0.79 mmol, 73 %) as a colourless oil. The amine was directly subjected to the next step without further purification.

HRMS (ESI) calcd for $C_7H_{12}NO_2$ [(M+H)$^+$]: 142.0863, found 142.0865; **$[\alpha]^{25}_D$** = + 12.5 ° ($CHCl_3$, 25 °C).

The spectroscopic data are in agreement with those previously reported in the literature.[12]

Synthesis of methyl (*R*)-2-methyl-2-(2-nitrobenzamido)pent-4-ynoate ((*R*)-53a)

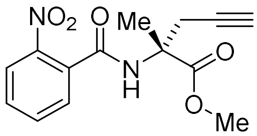

(*R*)-2-methyl-2-(2-nitrobenzamido)pent-4-ynoate was synthesized according to general procedure 3 from **(*R*)-15b** (100 mg, 0.71 mmol), purified by flash column chromatography (silica gel; petroleum ether/EtOAc, gradient 3:2 to 1:1) and isolated as a pale yellow solid (100 mg, 0.34 mmol, 48%).

R_f = 0.30 (petroleum ether/EtOAc, 1:1); **m.p.** = 146 – 147 °C; **$[\alpha]^{25}_D$** = – 43.7 ° ($CHCl_3$, 25 °C); **^{1}H** NMR (400 MHz, $CDCl_3$) δ 8.06 (1H, dd, *J* = 8.4, 1.2 Hz), 7.67 (1H, td, *J* = 7.5, 1.2 Hz), 7.60 – 7.56 (2H, m), 6.76 (1H, s), 3.84 (3H, s), 3.32 (1H, dd, *J* = 17.0, 2.7 Hz), 2.98 (1H, dd, *J* = 16.9, 2.7 Hz), 2.06 (1H, t, *J* = 2.6 Hz), 1.77 (3H, s); **^{13}C** NMR (101 MHz, $CDCl_3$) δ 173.3, 165.7, 146.5, 133.8, 132.8, 130.7, 129.0, 124.7, 79.5, 71.4, 60.0, 53.4, 26.5, 22.4; **HRMS** (ESI) calcd

for $C_{14}H_{15}N_2O_5$ [(M+H)$^+$]: 291.0981, found 291.0981; **IR** ν_{max} = 3282, 1753, 1641, 1565, 1526, 1451, 1440, 1376, 1346, 1290, 1265, 1215, 1175, 1163, 1138, 1110, 1077.

Synthesis of methyl (*R*)-2-methyl-2-(*N*-methyl-2-nitrobenzamido)pent-4-ynoate ((*R*)-54a)

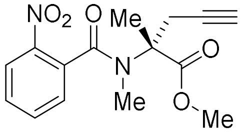

(*R*)-2-methyl-2-(*N*-methyl-2-nitrobenzamido)pent-4-ynoate was synthesized according to general procedure 4 from amide **(*R*)-53a** (50 mg, 0.170 mmol), purified by flash column chromatography (silica gel; petroleum ether/EtOAc, 1:1) and isolated as a white solid (51 mg, 0.167 mmol, 98%).

R*f* = 0.31 (petroleum ether/EtOAc, 1:1); **m.p.** = 113 – 114 °C; **$[\alpha]^{25}_D$** = + 137.5 ° ($CHCl_3$, 25 °C); **^{1}H** NMR (400 MHz, $CDCl_3$) *δ* 8.19 (1H, dd, *J* = 8.3, 1.2 Hz), 7.72 (1H, td, *J* = 7.5, 1.2 Hz), 7.57 (1H, ddd, *J* = 8.7, 7.6, 1.5 Hz), 7.43 (1H, d, *J* = 7.6 Hz), 3.78 (3H, s), 3.50 (1H, dd, *J* = 17.3, 2.7 Hz), 3.01 (3H, s), 2.83 (1H, br s), 2.07 (1H, t, *J* = 2.7 Hz), 1.73 (3H, s); **^{13}C** NMR (101 MHz, $CDCl_3$) *δ* 173.3, 168.2, 145.1, 134.6, 133.4, 130.0, 128.2, 124.8, 80.4, 71.0, 62.0, 52.7, 34.5, 25.2, 21.6; **HRMS** (ESI) calcd for $C_{15}H_{17}N_2O_5$ [(M+H)$^+$]: 305.1137, found 305.1128; **IR** ν_{max} = 3268, 1743, 1641, 1531, 1489, 1444, 1395, 1348, 1317, 1265, 1236, 1147, 1112, 1053.

Synthesis of (*R*)-3,4-dimethyl-3-propyl-3,4-dihydro-1*H*-benzo[e][1,4]diazepine-2,5-dione ((*R*)-40)

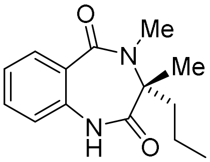

(*R*)-3,4-dimethyl-3-propyl-3,4-dihydro-1*H*-benzo[*e*][1,4]diazepine-2,5-dione was synthesized according to general procedure 5 from amide **(*R*)-54a** (40 mg, 0.13 mmol), purified by flash column chromatography (silica gel; petroleum ether/EtOAc, 1:1 to 2:3) and isolated as a white solid (23 mg, 0.093 mmol, 72%).

R*f* = 0.18 (petroleum ether/EtOAc, 1:1); **m.p.** = 140 – 141 °C; **$[\alpha]^{25}_D$** = + 182.1 ° ($CHCl_3$, 25 °C); **^{1}H** NMR (400 MHz, $CDCl_3$) *δ* 8.55 (1H, s), 7.96 (1H, dd, *J* = 7.9, 1.6 Hz), 7.44 (1H, td, *J* = 7.7, 1.6 Hz), 7.21 (1H, td, *J* = 7.6, 1.1 Hz), 6.93 (1H, dd, *J* = 8.0, 1.1 Hz), 3.23 (3H, s), 1.68

(3H, s), 1.65 – 1.57 (1H, m), 1.49 – 1.41 (1H, m), 1.26 – 1.16 (2H, m), 0.64 (3H, t, *J* = 7.3 Hz); **^{13}C** NMR (101 MHz, $CDCl_3$) *δ* 173.4, 168.3, 135.0, 132.5, 131.4, 127.8, 124.7, 119.2, 62.8, 38.4, 32.6, 23.1, 17.4, 14.1; **HRMS** (ESI) calcd for $C_{14}H_{19}N_2O_2$ [$(M+H)^+$]: 247.1441, found 247.1447; **IR** ν_{max} = 3239, 3154, 2956, 2132, 1738, 1676, 1645, 1604, 1580, 1529, 1486, 1455, 1413, 1378, 1346, 1318, 1289, 1264, 1238, 1193, 1161, 1122, 1076, 1035.

Synthesis of *(S)*-3-methyl-5-phenyl-5,6-dihydro-2*H*-1,4-oxazin-2-one ((*S)*-61)

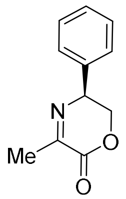

To a solution of **12b** (1.20 mL, 13.2 mmol) and molecular sieves (6.00 g) in trifluoroethanol (36 mL) was added (*S*)-phenyl glycinol (1.64 g, 12.0 mmol). The reaction was sealed in vial and irradiated to 120 °C for 50 minutes. The reaction was filtered through celite and purified by flash column chromatography (silica gel, petroleum ether/EtOAc, 4:1) to give the title compound (1.04 g, 5.47 mmol, 46%) as a white solid.

R*f* = 0.25 (petroleum ether/EtOAc, 4:1); **^{1}H** NMR (400 MHz, $CDCl_3$) δ 7.43 – 7.39 (2H, m) 7.37 – 7.33 (3H, m), 4.87 – 4.82 (1H, m), 4.56 (1H, dd, *J* = 11.6, 4.5 Hz), 4.26 (1H, m), 2.41 (3H, d, *J* = 2.4 Hz); **^{13}C** NMR (101 MHz, $CDCl_3$) δ 160.4, 155.6, 136.9, 129.0, 128.4, 127.2, 71.6, 59.8, 21.9; **HRMS** (ESI) calcd for $[C_{11}H_{12}NO_2]^+$: 190.0868, found 190.0864; **IR** ν_{max} = 3034, 3007, 1721, 1641, 1455; $[\alpha]_D^{25}$ **=** +214.0 (c = 0.1 in $CHCl_3$).

Synthesis of (3*S*,5*S*)-3-methyl-5-phenyl-3-(prop-2-yn-1-yl)morpholin-2-one ((*S*)-62)

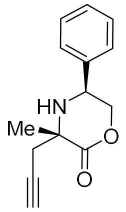

To a solution of **(*S*)-61** (960 mg, 5.08 mmol) in DMF (25 mL) cooled to -20 °C was added Zn (495 mg, 7.62 mmol) followed by propargyl bromide (810 uL, 7.62 mmol). The reaction was slowly warmed to 0 °C over 3 hours 40 minutes. Upon completion, the reaction was cooled to -15 °C and quenched with NH_4Cl saturated aqueous solution. The aqueous was extracted with

EtOAc (3 x 20 mL) and the combined organic extracts washed with brine, dried over Na_2SO_4 and concentrated *in vacuo*. The crude mixture was purified by flash column chromatography (silica gel; petroleum ether/EtOAc, 22:3) to give the title compound (606 mg, 2.64 mmol, 52%).

R_f = 0.25 (petroleum ether/EtOAc, 22:3); **^{1}H** NMR (400 MHz, $CDCl_3$) δ 7.46 – 7.44 (2H, m), 7.41 – 7.33 (3H, m), 4.36 – 4.26 (3H, m), 3.10 (1H, dd, *J* = 16.9, 2.4 Hz), 2.64 (1H, dd, *J* = 16.9, 2.6 Hz), 2.34 (1H, br s), 2.09 (1H, t, *J* = 2.6 Hz), 1.57 (3H, s); **^{13}C** NMR (101 MHz, $CDCl_3$) δ 172.2, 137.7, 129.0, 128.9, 127.4, 78.5, 75.4, 73.0, 59.0, 53.2, 28.7, 26.8; **HRMS** (ESI) calcd for $[C_{14}H_{16}NO_2]^+$: 230.1181, found 230.1171; **IR** ν_{max} = 3239, 2916, 2163, 1730, 1455, 1430, 1146**;** $[\alpha]_D^{25}$ **=** -26.0 (c = 0.1 in $CHCl_3$).

Synthesis of methyl (*S*)-2-(((*S*)-2-hydroxy-1-phenylethyl)amino)-2-methylpent-4-ynoate ((*S*)-63)

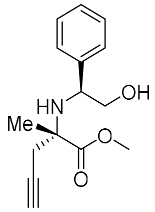

To a solution of **(*S*)-62** (430 mg, 1.88 mmol) in MeOH (18.8 mL) was added $SOCl_2$ (272 uL, 3.75 mmol) and the reaction stirred for 2 hours at room temperature. Then, the reaction was concentrated *in vacuo* and $NaHCO_3$ saturated aqueous solution and EtOAc were added and stirred for 15 minutes. The organic layer was separated and the aqueous extracted twice more. The combined organic extracts were dried over Na_2SO_4 and concentrated *in vacuo*. The crude product was purified by flash column chromatography (silica gel; petroleum ether/EtOAc, 7:3) to give the title compound (285 mg, 1.09 mmol, 58%) as a colourless oil.

R_f = 0.17 (petroleum ether/EtOAc, 7:3); **^{1}H** NMR (400 MHz, $CDCl_3$) δ 7.34 – 7.30 (2H, m), 7.27 – 7.23 (3H, m), 3.84 (1H, dd, *J* = 9.2, 4.7 Hz), 3.57 (1H, dd, *J* = 10.8, 4.4 Hz), 3.48 – 3.41 (1H, m), 3.38 (3H, s), 3.02 (1H, br s), 2.54 (1H, dd, *J* = 16.6, 2.6 Hz), 2.47 (1H, dd, *J* = 16.6, 2.6 Hz), 2.30 (1H, br s), 2.00 (1H, t, *J* = 2.6 Hz), 1.41 (3H, s); **^{13}C** NMR (101 MHz, $CDCl_3$) δ 175.0, 141.1, 128.6, 127.7, 127.2, 79.9, 71.4, 67.1, 60.9, 59.8, 52.1, 30.1, 22.6; **HRMS** (ESI) calcd for $[C_{15}H_{20}NO_3]^+$: 262.1443, found 262.1432; **IR** ν_{max} = 3284, 2948, 2342, 1728, 1454, 1113; $[\alpha]_D^{25}$ **=** +41 (c = 0.1 in $CHCl_3$).

Synthesis of methyl (*S*)-2-amino-2-methylpent-4-ynoate (*(S)*-15b)

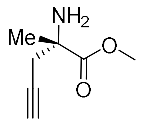

To a solution of **(*S*)-63** (250 mg, 0.957 mmol) in MeOH (3 mL) and CH_2Cl_2 (6 mL) cooled to 0 °C was added $Pb(OAc)_4$ (593 mg, 1.33 mmol) and the reaction stirred for 1 hour warming to room temperature Then, 2N HCl (aq., 10 mL) was added and stirred for 10 minutes. The resulting suspension was filtered through a silica plug, eluting with MeOH and the solvents removed *in vacuo*. The aqueous residue was extracted with EtOAc (3 x 10 mL), basified with Na_2CO_3 to pH 10-11 and extracted with EtOAc (3 x 10 mL). The combined organic extracts were dried over Na_2SO_4 and concentrated *in vacuo* to give the title compound (110 mg, 0.779 mmol, 81%) as a colourless oil.

R_f = 0.15 (petroleum ether/EtOAc, 5:3); **^{1}H** NMR (400 MHz, $CDCl_3$) δ 3.72 (3H, s), 2.64 (1H, dd, *J* = 2.6, 16.5 Hz), 2.44 (1H dd, *J* = 2.6, 16.5 Hz), 2.04 (1H, t, *J* = 2.6 Hz), 1.87 (2H, br s), 1.36 (3H, s); **^{13}C** NMR (101 MHz, $CDCl_3$) δ 176.6, 79.8, 71.5, 57.5, 52.6, 31.0, 26.0; **HRMS** (ESI) calcd for $[C_7H_{12}NO_2]^+$:142.0868, found 142.0862; **IR** ν_{max} = 3295, 2953, 2358, 1732, 1116; $[\alpha]_D^{25}$ = -13.0 (c = 0.1 in $CHCl_3$).

Synthesis of methyl (*S*)-2-methyl-2-(2-nitrobenzamido)pent-4-ynoate (*(S)*-53a)

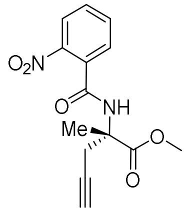

To a solution of ***(S)*-15b** (35.0 mg, 0.248 mmol) in CH_2Cl_2 (2.5 mL) cooled to 0 °C was added triethylamine (41.0 uL, 0.298 mmol) followed by 2-nitrobenzoyl chloride (39.0 uL, 0.298 mmol) and the reaction stirred for 2 hours warming to room temperature. Then, the reaction was quenched with NH_4Cl saturated aqueous solution and extracted with EtOAc (3 x 10 mL). The combined organic extracts were dried over Na_2SO_4 and concentrated *in vacuo*. The crude product was purified by flash column chromatography (silica gel; petroleum ether/EtOAc, 1:1) to give the title compound (48.0 mg, 0.166 mmol, 67%) as a colourless oil.

R_f = 0.22 (petroleum ether/EtOAc, 11:9); **^{1}H** NMR (400 MHz, $CDCl_3$) δ 8.09 – 8.06 (1H, m), 7.70 – 7.66 (1H, m), 7.61 – 7.57 (2H, m), 6.74 (1H, br s), 3.85 (3H, s), 3.34 (1H, dd, *J* = 16.9,

2.6 Hz), 2.98 (1H, dd, *J* = 16.9, 2.6 Hz), 2.06 (1H, t, *J* = 2.6 Hz), 1.78 (3H, s); **^{13}C** NMR (101 MHz, $CDCl_3$) δ 173.3, 165.7, 146.5, 133.8, 132.8, 130.7, 129.0, 124.7, 79.5, 71.4, 60.1, 53.4, 26.6, 22.4; **HRMS** (ESI) calcd for $[C_{14}H_{15}N_2O_5]^+$: 291.0981; found: 291.0993; **IR** ν_{max} = 3280, 3187, 3005, 2159, 1751, 1640, 1563, 1524; $[\alpha]_D^{25}$ **=** -35.0 (c = 0.1 in $CHCl_3$).

Synthesis of methyl (*S*)-2-methyl-2-(*N*-methyl-2-nitrobenzamido)pent-4-ynoate (*(S)*-54a)

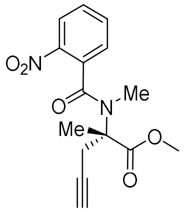

To a solution of **(*S*)-61** (37.0 mg, 0.127 mmol) in DMF (1.3 mL) at 0 °C was added NaH (60% dispersion in mineral oil, 7.60 mg, 0.190 mmol) and the reaction stirred for 15 minutes before MeI (16.0 uL, 0.254 mmol) was added. After 1 hour stirring at room temperature the reaction was quenched with NH_4Cl saturated aqueous solution and extracted with EtOAc (3 x 10 mL). The combined organic extracts were washed with brine, dried over Na_2SO_4 and concentrated *in vacuo*. The crude product was purified by flash column chromatography (silica gel; petroleum ether/EtOAc, 1:1) to give **112** (32.0 mg, 0.105 mmol, 83%) as a white solid.

R*f* = 0.27 (petroleum ether/EtOAc, 1:1); **^{1}H** NMR (400 MHz, $CDCl_3$) δ 8.19 (1H, dd, *J* = 8.3, 0.7 Hz), 7.72 (1H, td, *J* = 7.5, 1.6 Hz), 7.59 – 7.55 (1H, m), 7.43 (1H, d, *J* = 7.4 Hz), 3.78 (3H, s), 3.50 (1H, dd, *J* = 17.3, 2.2 Hz), 3.02 (3H, s), 2.86 (1H, br s), 2.07 (1H, t *J* = 2.6 Hz), 1.73 (3H, s); **^{13}C** NMR (101 MHz, $CDCl_3$) δ 173.3, 168.1, 145.0, 134.6, 133.3, 129.8, 128.0, 124.7, 80.3, 70.9, 61.9, 52.6, 34.4, 25.0, 21.4; **HRMS** (ESI) calcd for $[C_{15}H_{17}N_2O_5]^+$: 305.1137, found 305.1130**;** **IR** ν_{max} = 3283, 2923, 1740, 1640, 1530; $[\boldsymbol{\alpha}]_{\boldsymbol{D}}^{\boldsymbol{25}}$ = -121.0 (c = 0.1 in $CHCl_3$).

Synthesis of (*S*)-3,4-dimethyl-3-propyl-3,4-dihydro-1*H*-benzo[*e*][1,4]diazepine-2,5-dione (*(S)*-40)

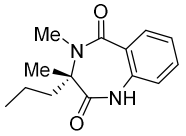

To a degassed solution of ***(S)*-54a** (23.0 mg, 0.0755 mmol) in MeOH (1 mL) was added Pd/C (8.00 mg, 800 µmol) and the reaction stirred under an atmosphere of H_2 for 2 hours 15 minutes. The reaction was filtered through celite, eluting with MeOH and the solvents removed

in vacuo. The crude intermediate was dissolved in DMF (0.6 mL) and NaH (60% dispersion in mineral oil, 6.00 mg, 0.151 mmol) was added and the reaction stirred for 1.5 hours. Then, the reaction was quenched with NH_4Cl saturated aqueous solution and extracted with EtOAc (3 x 10 mL). The combined organic extracts were washed with brine, dried over Na_2SO_4 and concentrated *in vacuo*. The crude product was purified by flash column chromatography (silica gel; petroleum ether/EtOAc, 3:2) to give the title compound (13.6 mg, 0.0552 mmol, 73%) as a white solid.

R_f = 0.14 (petroleum ether/EtOAc, 3:2); **^{1}H** NMR (400 MHz, $CDCl_3$) δ 8.25 (1H, br s), 7.97 (1H, dd, *J* = 1.4, 7.9 Hz), 7.44 (1H, td, *J* = 1.5, 11.4 Hz), 7.23 – 7.19 (1H, m), 6.90 (1H, d, *J* = 8.2 Hz), 3.23 (3H, s), 1.68 (3H, s), 1.63 – 1.57 (1H, m), 1.49 – 1.41 (1H, m), 1.26 – 1.19 (2H, m), 0.65 (3H, t, *J* = 7.3 Hz); **^{13}C** NMR (101 MHz, $CDCl_3$) δ 173.3, 168.3, 134.9, 132.5, 131.4, 127.8, 124.8, 119.1, 62.9, 38.4, 32.6, 23.1, 17.4, 14.1; **HRMS** (ESI) calcd for $[C_{14}H_{19}N_2O_2]^+$: 247.1441, found 247.1430; **IR** ν_{max} = 3151, 2958, 2873, 1676, 1603, 1487, 1432; $[\alpha]_D^{25}$ **=** +269.0 (c = 0.1 in $CHCl_3$).

5 References

1 M. R. Berthold, N. Cebron, F. Dill, T. R. Gabriel, T. Kotter, T. Meinl, P. Ohl, C. Sieb, K. Thiel and B. Wiswedel, in *Data Analysis, Machine Learning and Applications*, ed. C. Preisach, H. Burkhardt, L. Schmidt-Thieme, Springer, Berlin, Heidelberg, 2007, pp. 319–326.

2 D. Bellini, L. Koekemoer, H. Newman and C. G. Dowson, *J. Mol. Biol.*, 2019, **431**, 3501–3519.

3 X. Wang, G. Fischer and M. Hyvönen, *Nat. Commun.*, 2016, **7**, 1–11.

4 G. Winter, *J. Appl. Crystallogr.*, 2010, **43**, 186–190.

5 D. G. Waterman, G. Winter, R. J. Gildea, J. M. Parkhurst, A. S. Brewster, N. K. Sauter and G. Evans, *Acta Crystallogr. Sect. D*, 2016, **72**, 558–575.

6 W. Kabsch, *Acta Crystallogr. Sect. D*, 2010, **66**, 125–132.

7 P. Evans, *Acta Crystallogr. Sect. D*, 2006, **62**, 72–82.

8 M. D. Winn, C. C. Ballard, K. D. Cowtan, E. J. Dodson, P. Emsley, P. R. Evans, R. M. Keegan, E. B. Krissinel, A. G. W. Leslie, A. McCoy, S. J. McNicholas, G. N. Murshudov, N. S. Pannu, E. A. Potterton, H. R. Powell, R. J. Read, A. Vagin and K. S. Wilson, *Acta Crystallogr. Sect. D*, 2011, **67**, 235–242.

9 M. Wojdyr, R. Keegan, G. Winter, A. Ashton, A. Lebedev and E. Krissinel, *Acta Crystallogr. Sect. A*, 2014, **70**, C1447.

10 F. Long, R. A. Nicholls, P. Emsley, S. Gražulis, A. Merkys, A. Vaitkus and G. N. Murshudov, *Acta Crystallogr. Sect. D*, 2017, **73**, 112–122.

11 N. M. Pearce, T. Krojer, A. R. Bradley, P. Collins, R. P. Nowak, R. Talon, B. D. Marsden, S. Kelm, J. Shi, C. M. Deane and F. Von Delft, *Nat. Commun.*, 2017, **8**.

12 G. N. Murshudov, P. Skubák, A. A. Lebedev, N. S. Pannu, R. A. Steiner, R. A. Nicholls, M. D. Winn, F. Long and A. A. Vagin, *Acta Crystallogr. Sect. D*, 2011, **67**, 355–367.

13 P. Emsley, B. Lohkamp, W. G. Scott and K. Cowtan, *Acta Crystallogr. Sect. D*, 2010, **66**, 486–501.

14 N. Mateu, S. L. Kidd, L. Kalash, H. F. Sore, A. Madin, A. Bender and D. R. Spring, *Chem. – A Eur. J.*, 2018, **24**, 13681–13687.

15 G. Shang, Q. Yang and X. Zhang, *Angew. Chemie Int. Ed.*, 2006, **45**, 6360–6362.

16 C. Reddy, S. A. Babu and N. A. Aslam, *RSC Adv.*, 2014, **4**, 40199–40213.

17 K.-H. Park and M. J. Kurth, *J. Org. Chem.*, 1999, **64**, 9297–9300.

6 Supplementary Information: Spectra

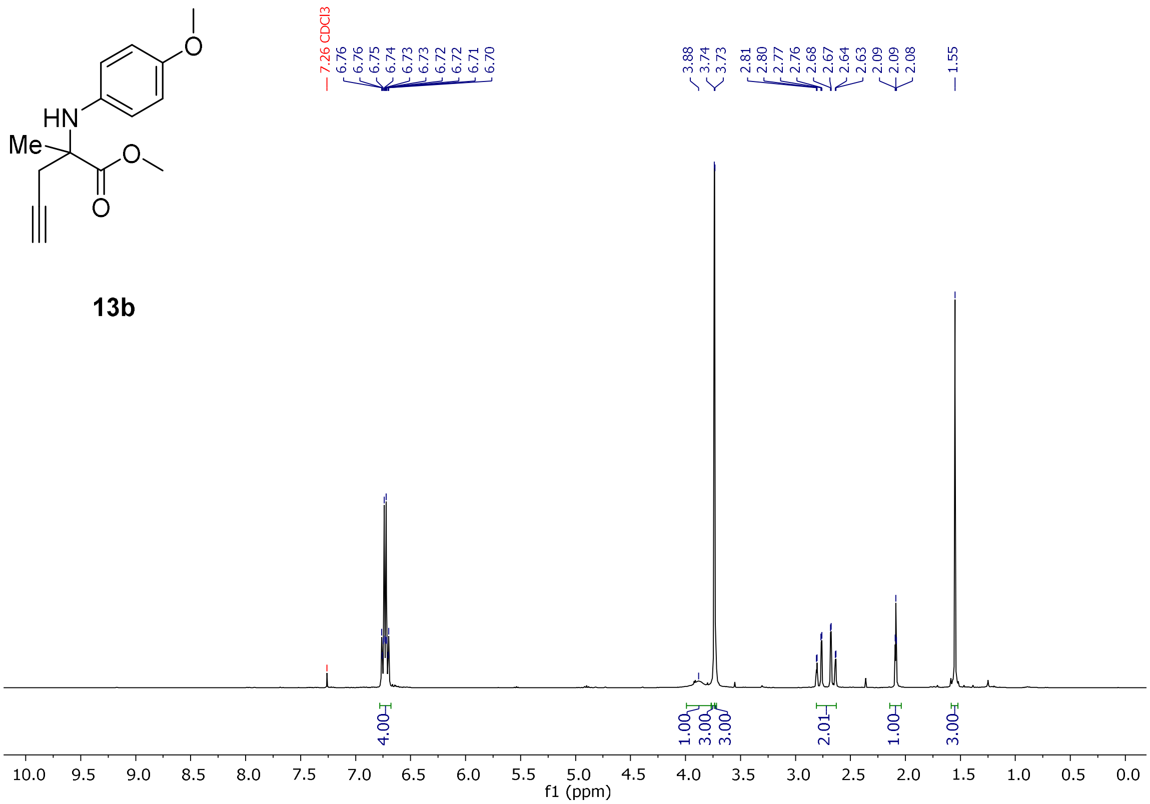

13b

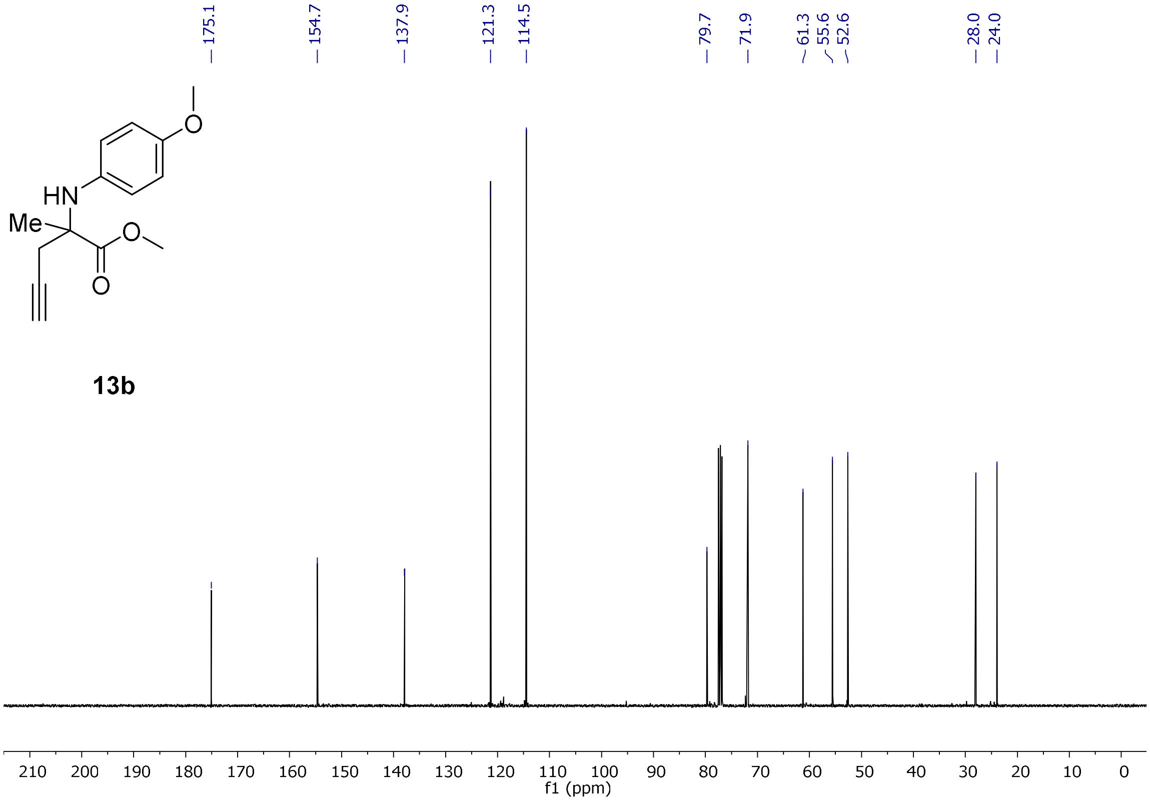

13b

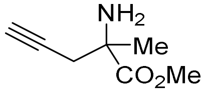
NH2
Me
CO2Me

15b

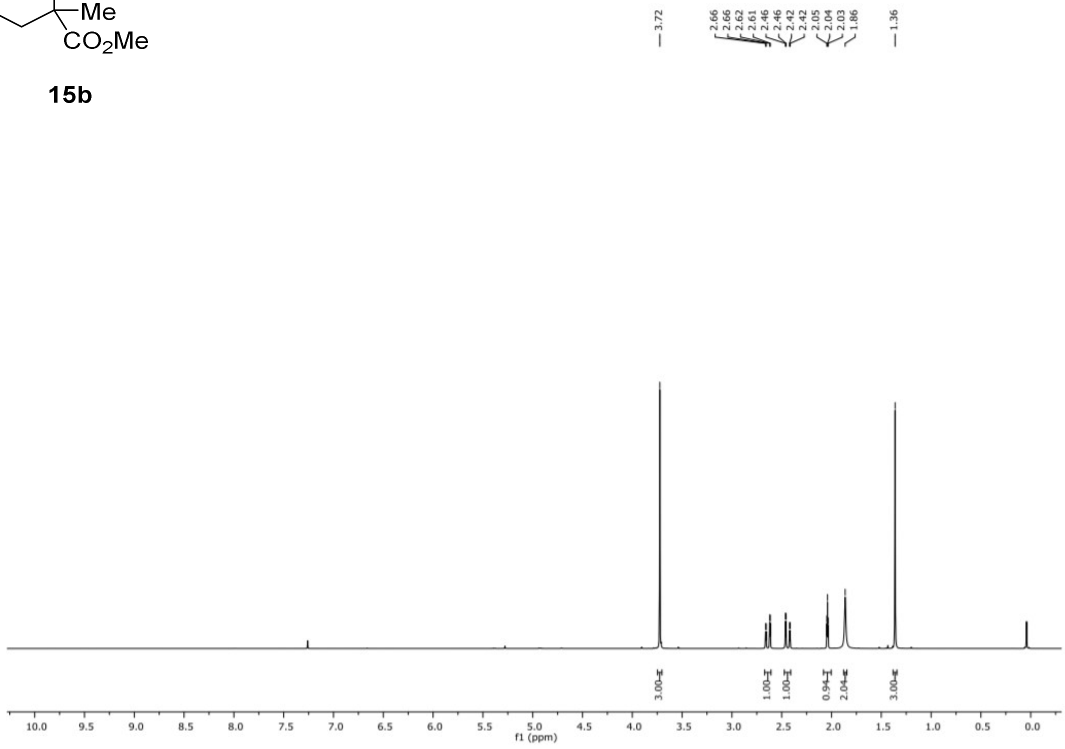
3.72
2.66
2.66
2.62
2.61
2.46
2.46
2.42
2.42
2.05
2.04
2.03
1.86
1.36
3.00
1.00
1.00
0.94
2.04
3.00
f1 (ppm)

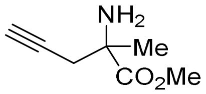
NH2
Me
CO2Me

15b

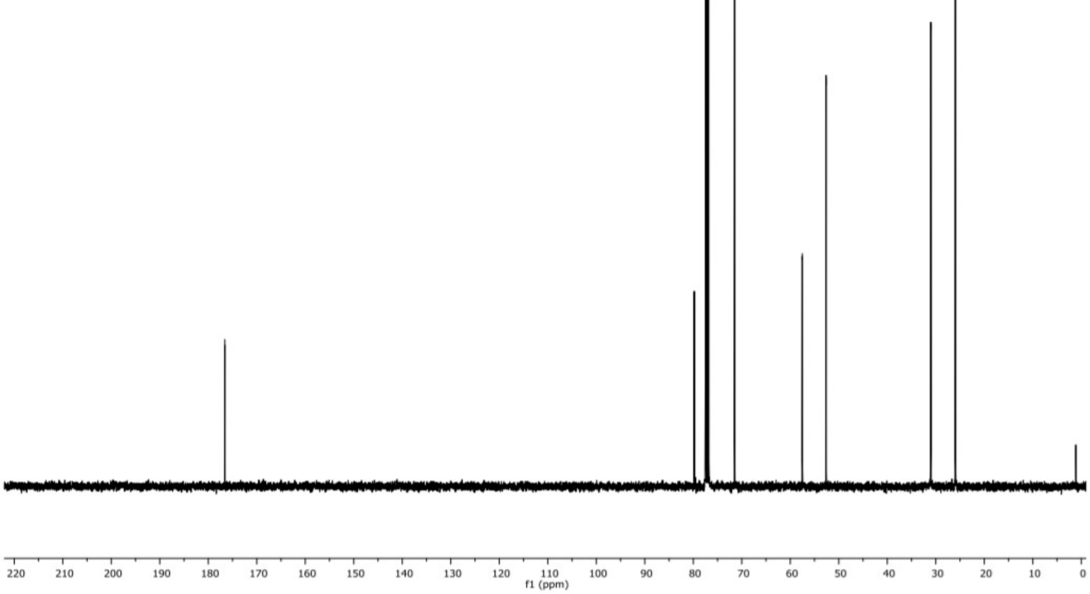
176.6
79.8
71.5
57.5
52.6
31.0
26.0
f1 (ppm)

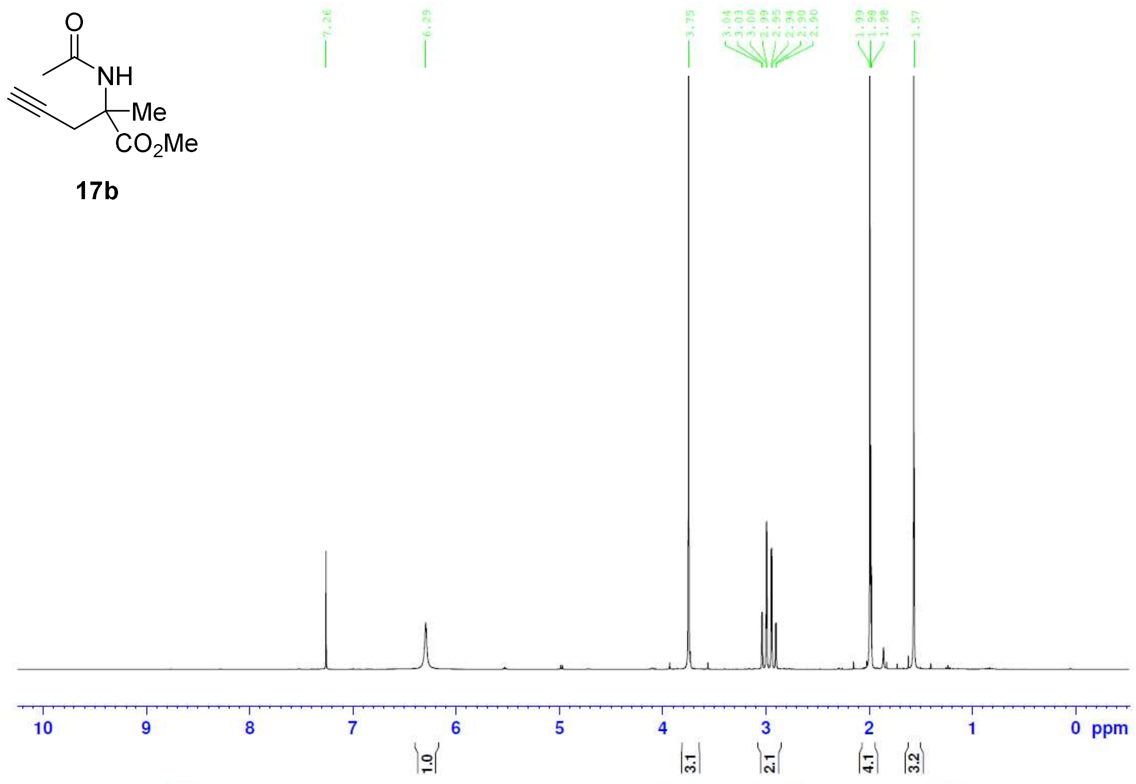

17b
ppm

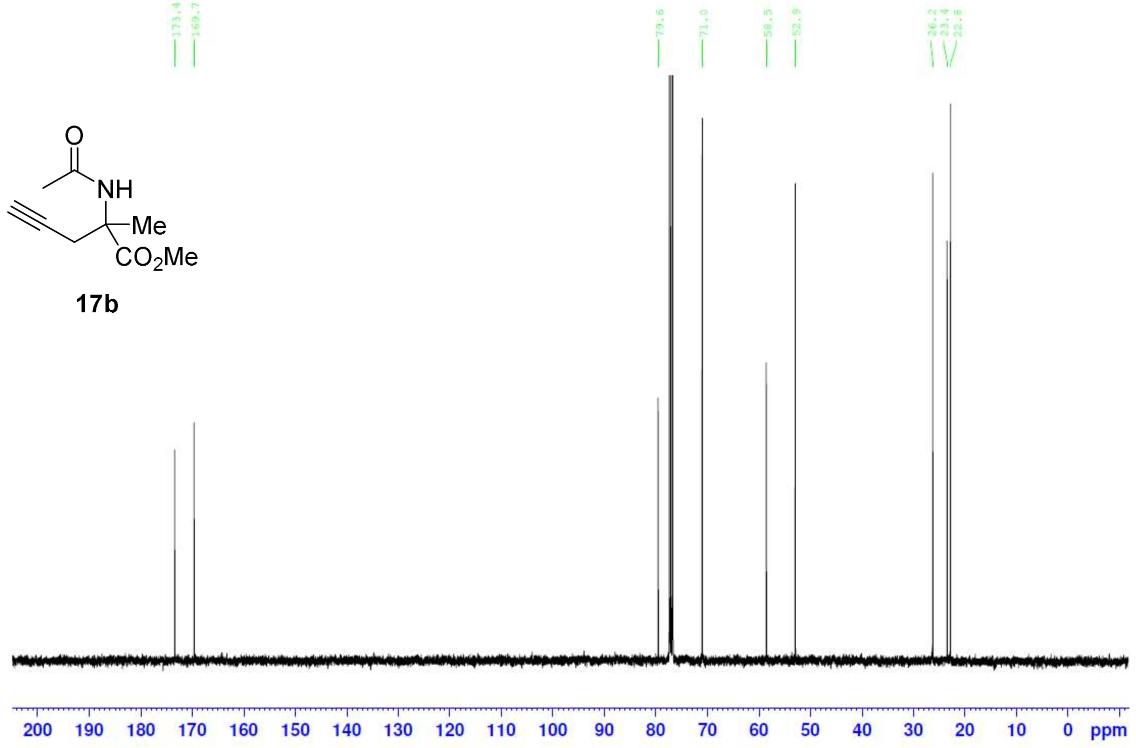

17b
ppm

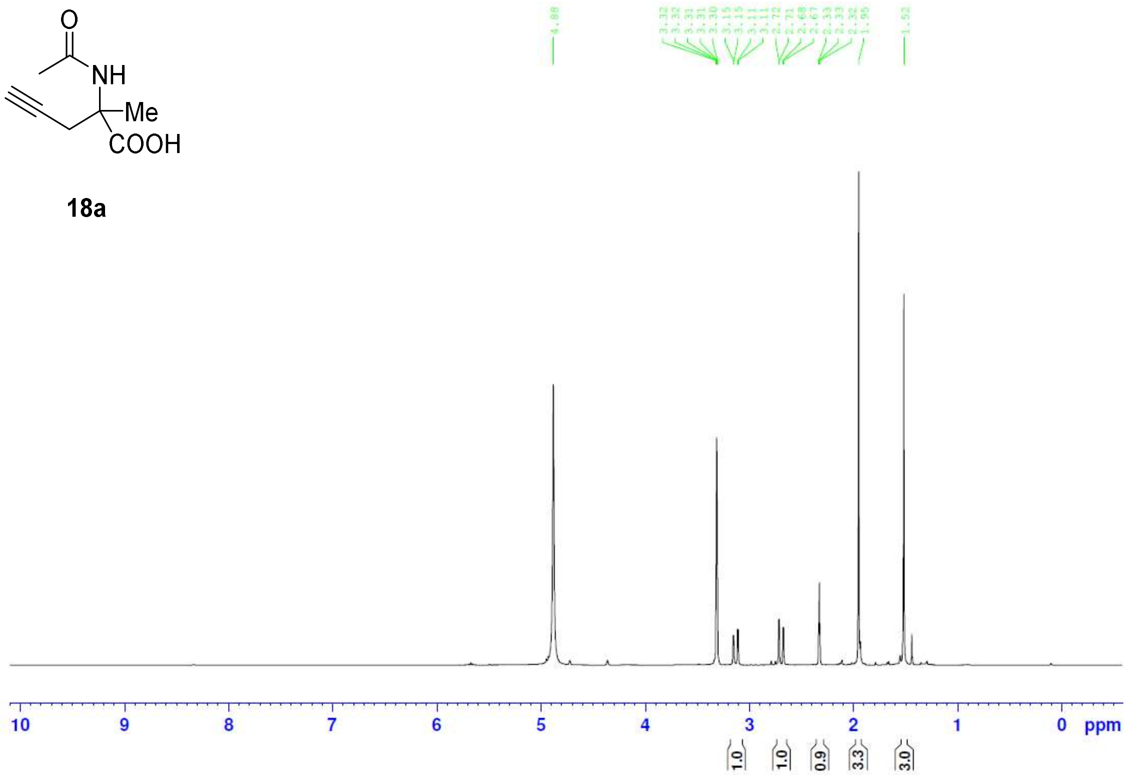
O
NH
Me
COOH
10 9 8 7 6 5 4 3 2 1 0 ppm
1.0 1.0 0.9 3.3 3.0

18a

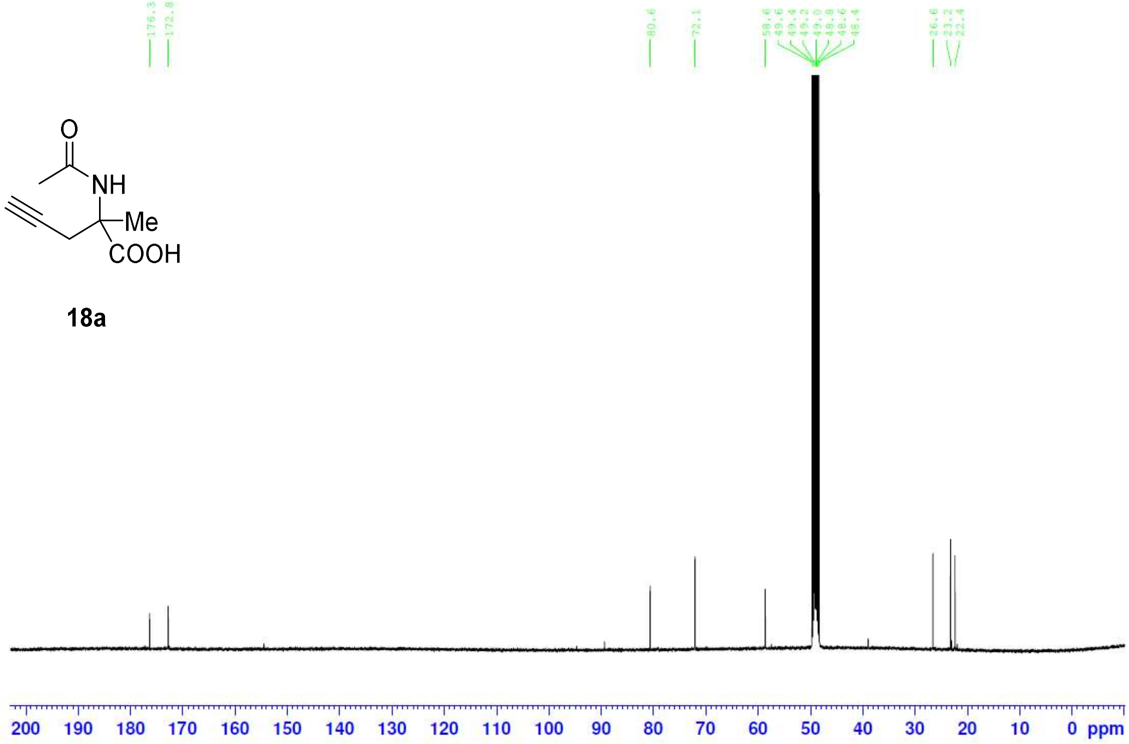
176.3
172.8
80.6
72.1
58.6
26.6
23.2
22.4
O
NH
Me
COOH
200 190 180 170 160 150 140 130 120 110 100 90 80 70 60 50 40 30 20 10 0 ppm

18a

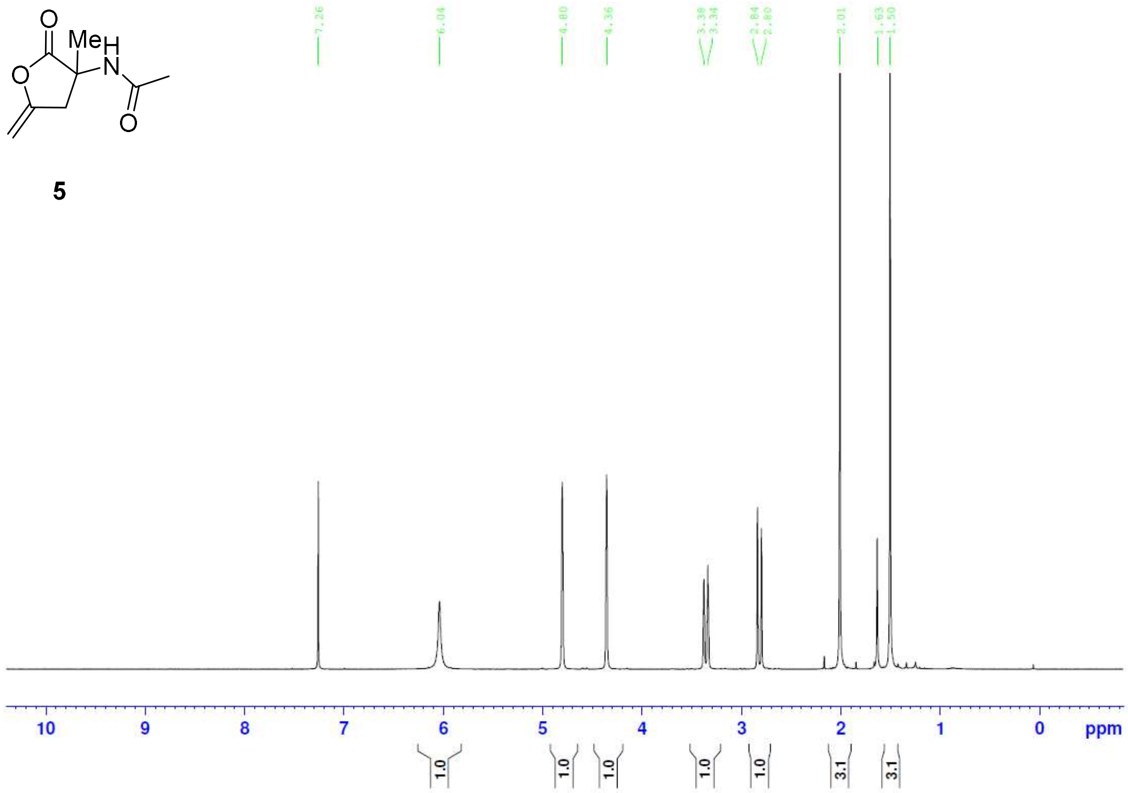
O
Me
H
N
O
O
7.26
6.04
4.80
4.36
3.38
3.34
2.84
2.80
2.01
1.63
1.50
10
9
8
7
6
5
4
3
2
1
0
ppm
1.0
1.0
1.0
1.0
1.0
3.1
3.1

5

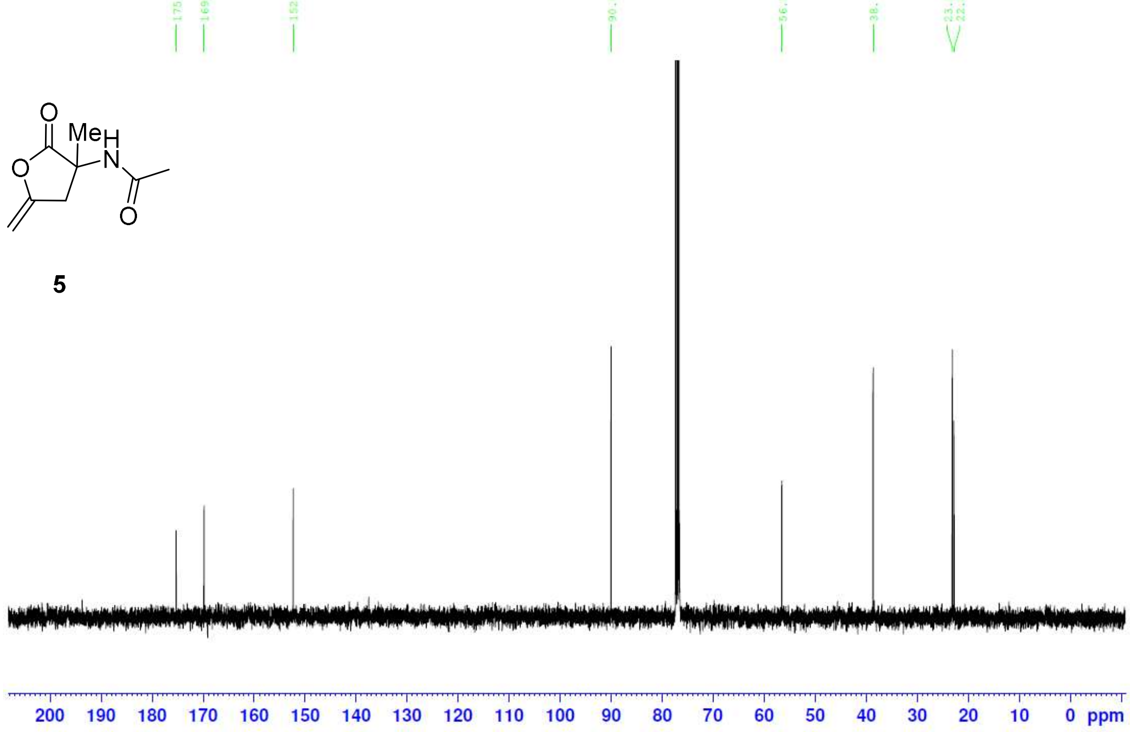
175.3
169.8
152.4
90.0
56.6
38.7
23.1
22.8
O
Me
H
N
O
O
200
190
180
170
160
150
140
130
120
110
100
90
80
70
60
50
40
30
20
10
0
ppm

5

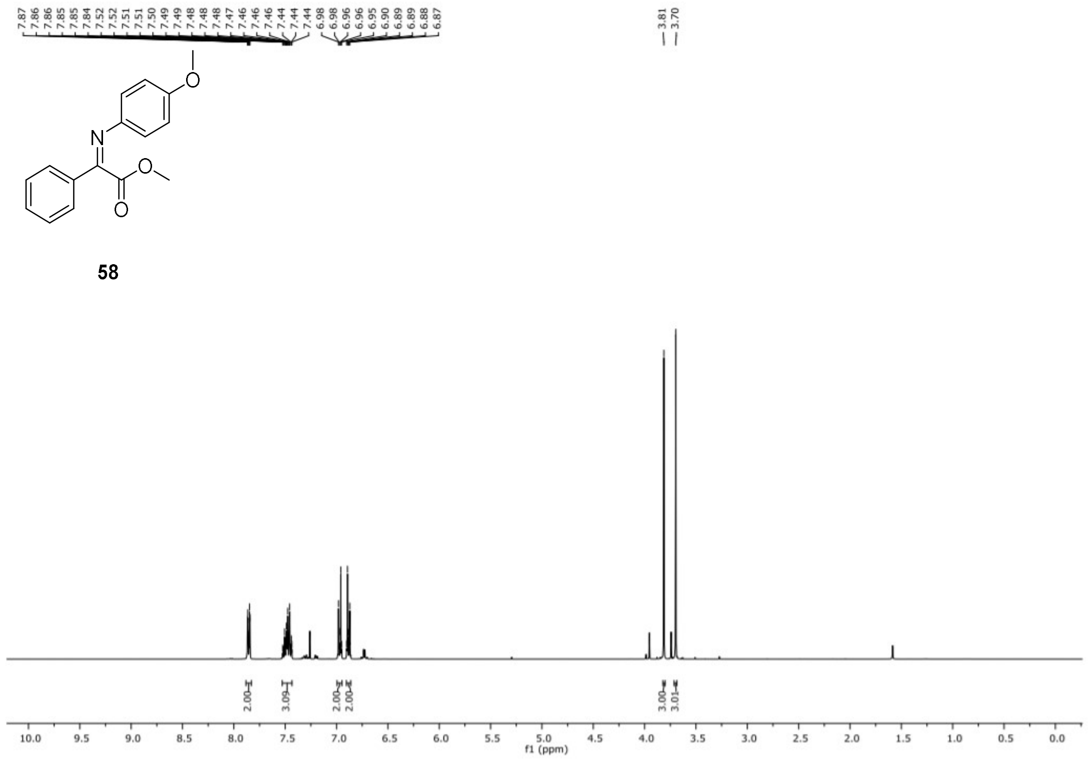
7.87 7.86 7.86 7.85 7.84 7.52 7.52 7.51 7.51 7.50 7.49 7.49 7.48 7.48 7.48 7.47 7.46 7.46 7.46 7.44 7.44 7.44 6.98 6.98 6.96 6.96 6.95 6.90 6.89 6.89 6.88 6.87
3.81
3.70
2.00
3.09
2.00
2.00
3.00
3.01
f1 (ppm)

58

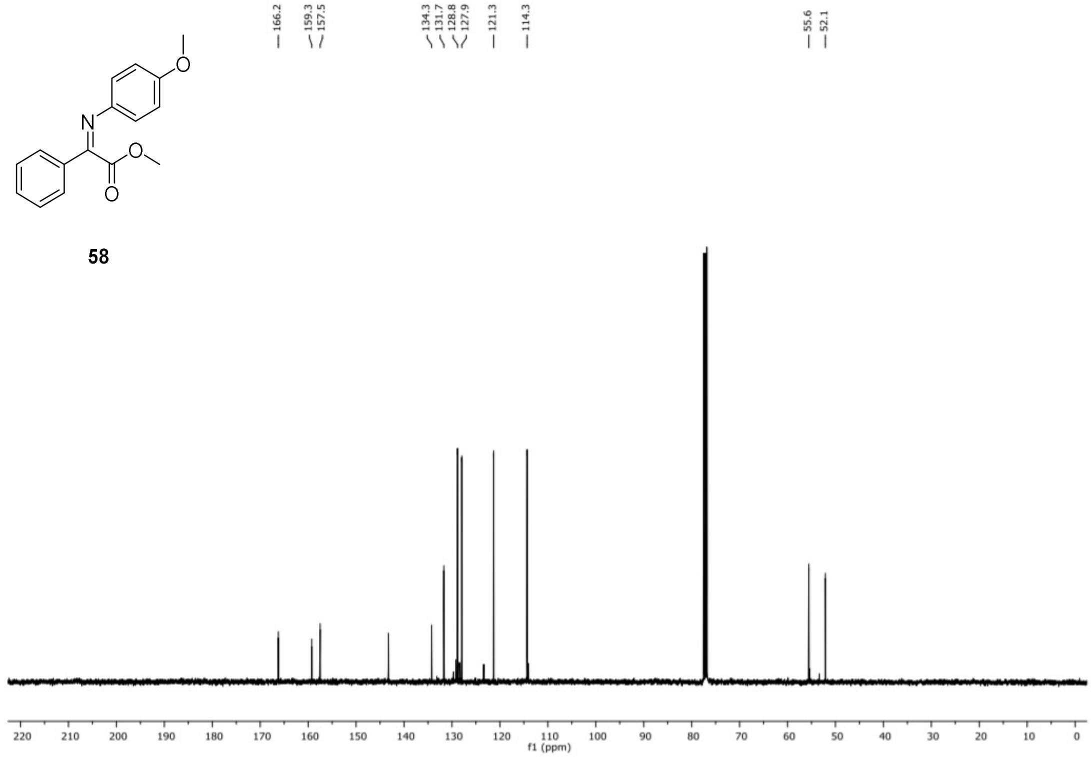
166.2
159.3
157.5
134.3
131.7
128.8
127.9
121.3
114.3
55.6
52.1
f1 (ppm)

58

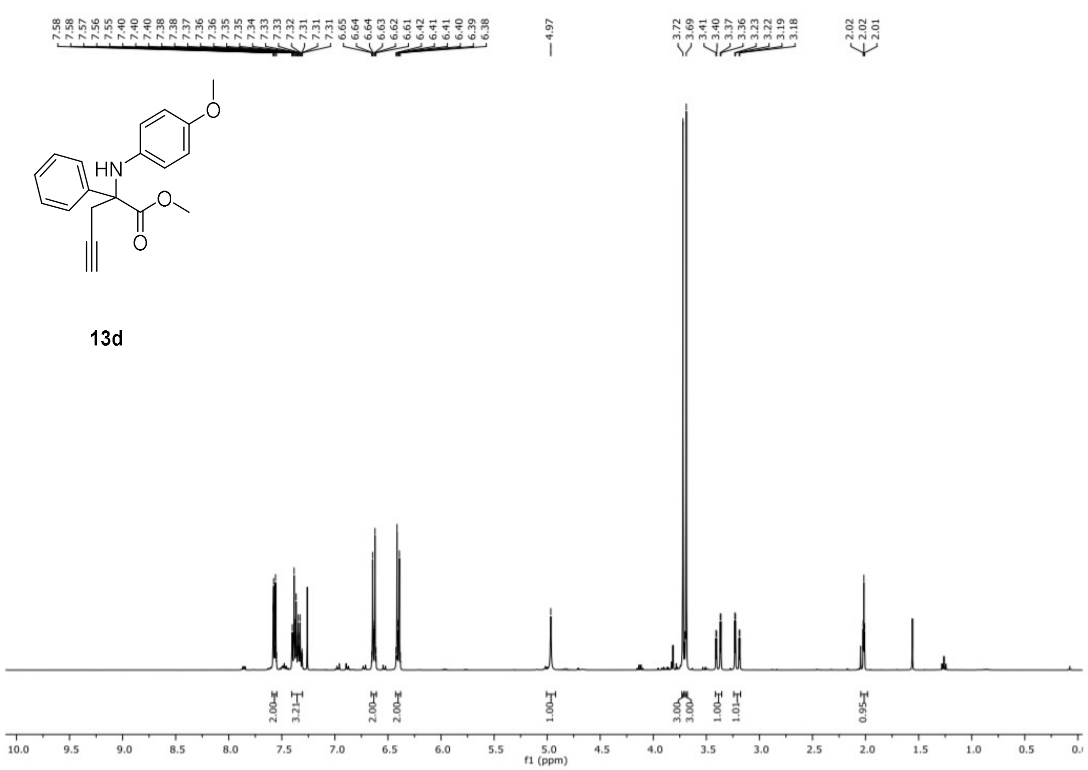

7.58
7.58
7.57
7.56
7.55
7.40
7.40
7.38
7.38
7.37
7.36
7.36
7.35
7.35
7.34
7.33
7.33
7.32
7.31
7.31
6.65
6.64
6.63
6.62
6.61
6.42
6.41
6.41
6.40
6.39
6.38
4.97
3.72
3.69
3.41
3.40
3.37
3.36
3.23
3.22
3.19
3.18
2.02
2.02
2.01
HN
O
f1 (ppm)

13d

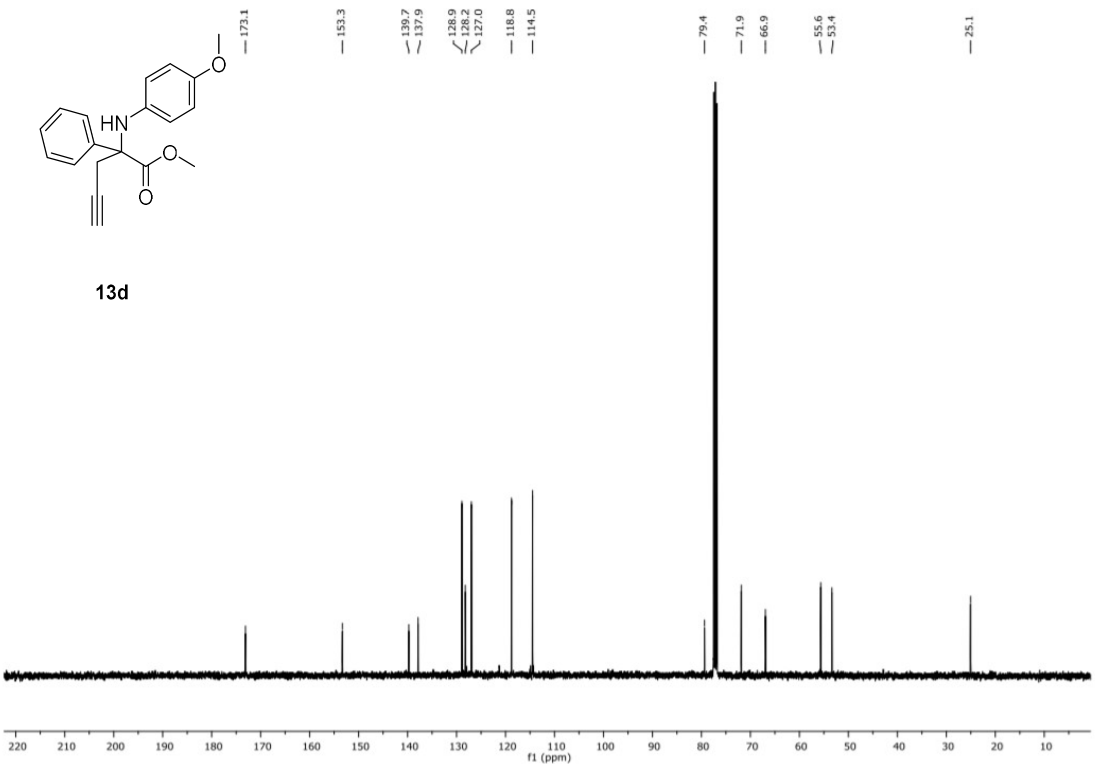

173.1
153.3
139.7
137.9
128.9
128.2
127.0
118.8
114.5
79.4
71.9
66.9
55.6
53.4
25.1
HN
O
f1 (ppm)

13d

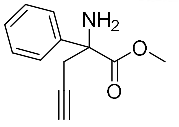

NH2
O
O

15d

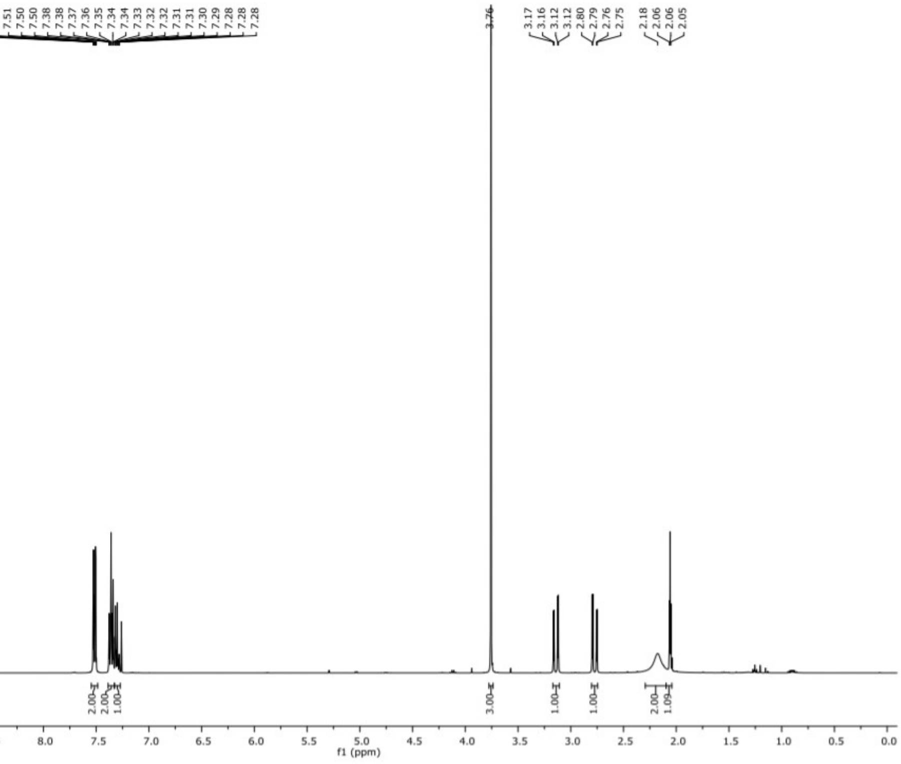

7.53
7.52
7.52
7.51
7.51
7.50
7.50
7.38
7.38
7.37
7.36
7.35
7.34
7.34
7.33
7.32
7.32
7.31
7.31
7.30
7.29
7.28
7.28
7.28
3.76
3.17
3.16
3.12
3.12
2.80
2.79
2.76
2.75
2.18
2.06
2.06
2.05
2.00
2.00
1.00
3.00
1.00
1.00
2.00
1.09
10.0 9.5 9.0 8.5 8.0 7.5 7.0 6.5 6.0 5.5 5.0 4.5 4.0 3.5 3.0 2.5 2.0 1.5 1.0 0.5 0.0
f1 (ppm)

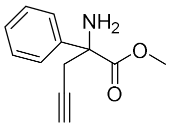

NH2
O
O

15d

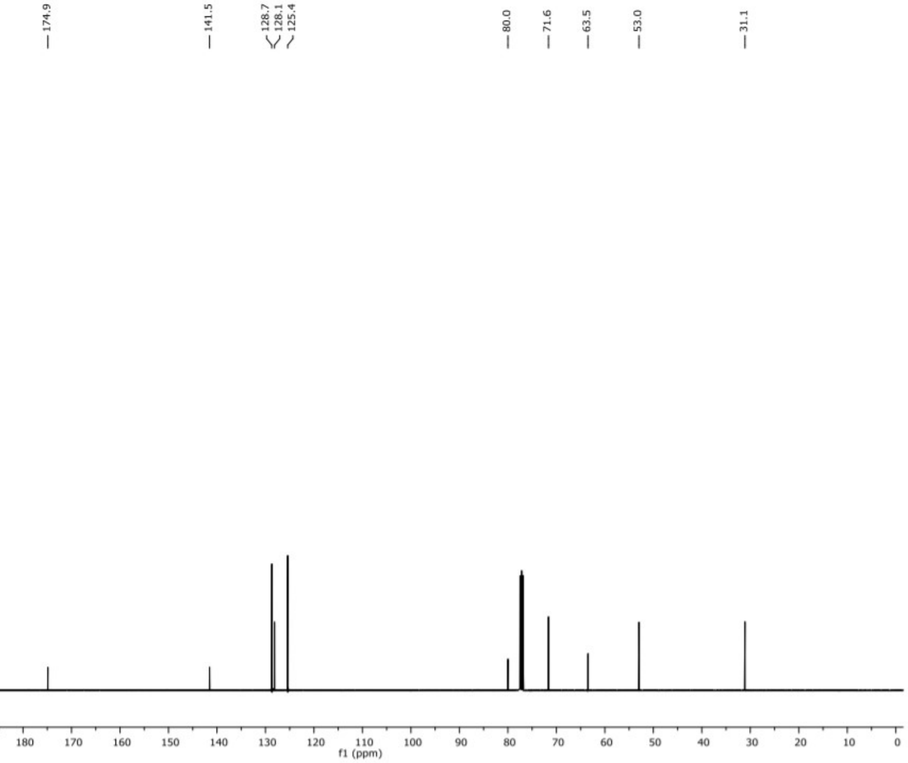

174.9
141.5
128.7
128.1
125.4
80.0
71.6
63.5
53.0
31.1
220 210 200 190 180 170 160 150 140 130 120 110 100 90 80 70 60 50 40 30 20 10 0
f1 (ppm)

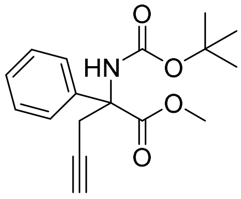

17d

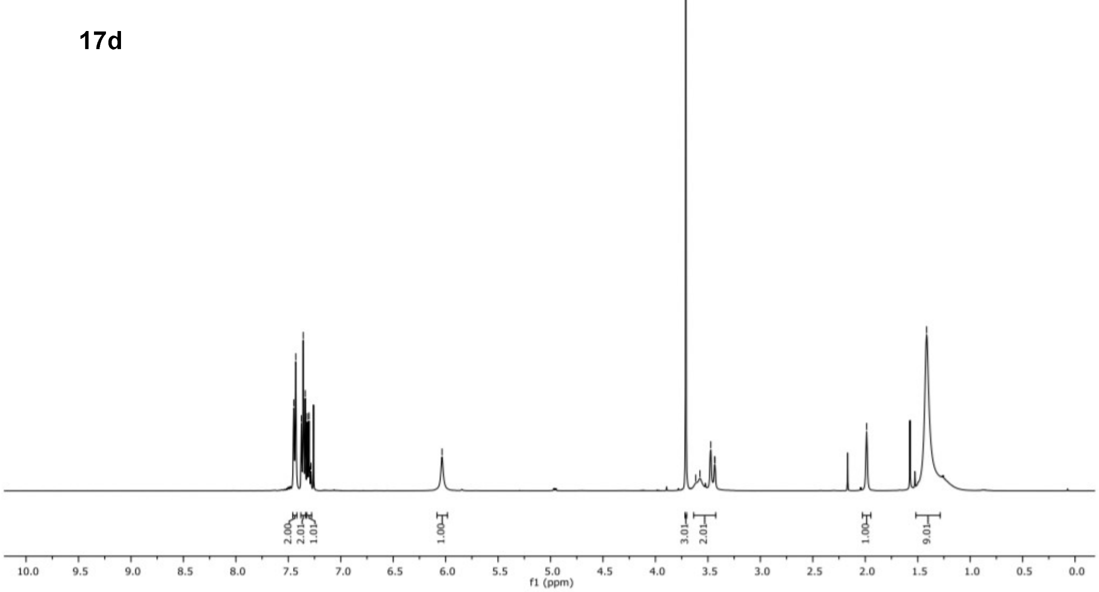

7.45 7.43 7.38 7.37 7.36 7.34 7.32 7.31 7.30 7.29 7.28
6.03
3.71 3.62 3.58 3.47 3.44 3.43
1.99
1.41
2.00 2.01 1.01 1.00 3.01 2.01 1.00 9.01
f1 (ppm)

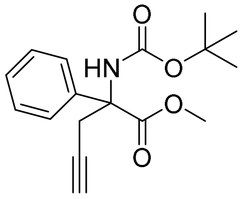

17d

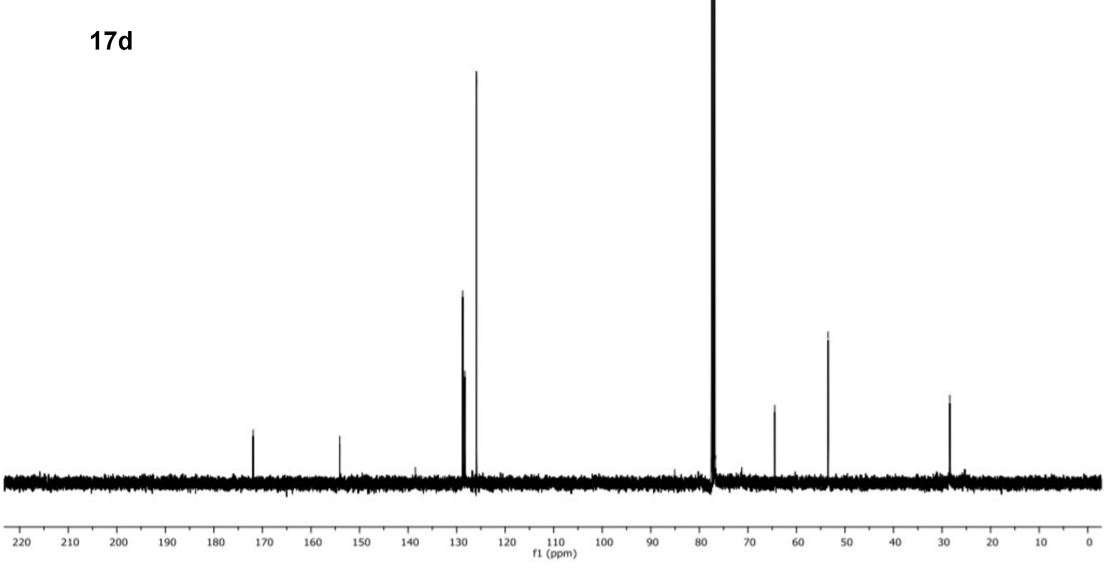

171.9
154.1
138.5
128.7 128.3 125.9
85.1
64.5
53.5
28.4
f1 (ppm)

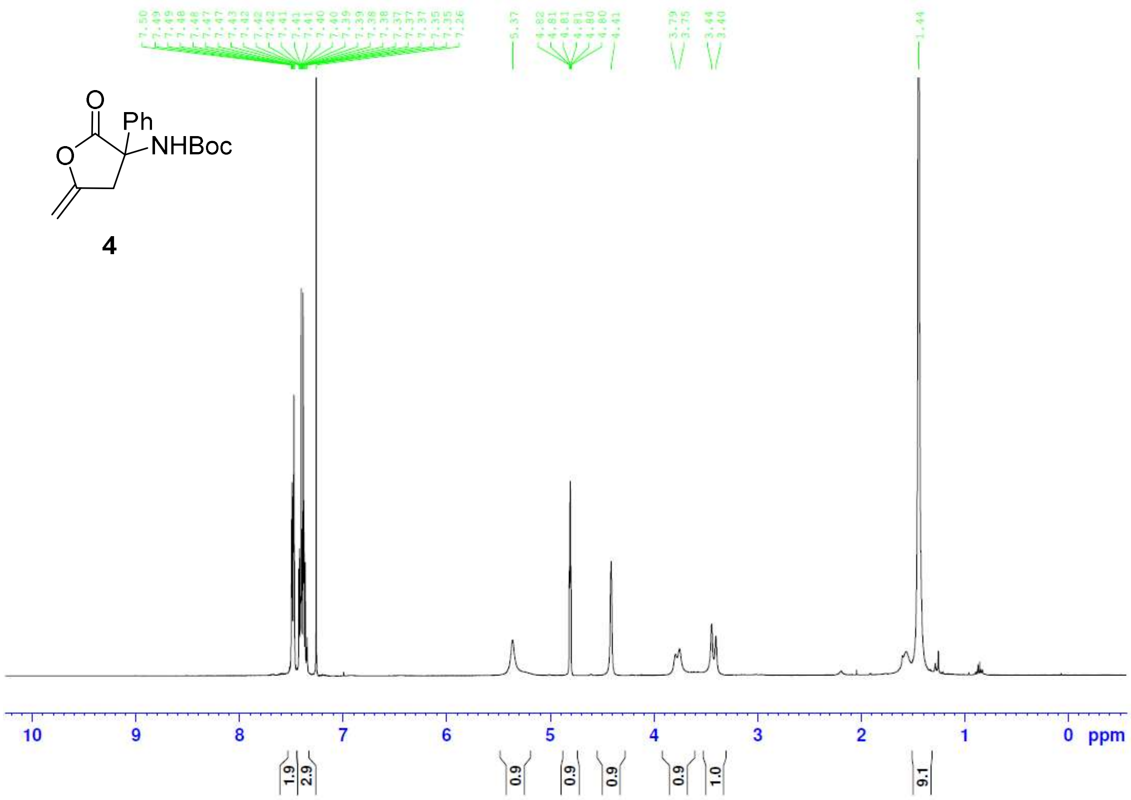
O
Ph
NHBoc
O
4
10 9 8 7 6 5 4 3 2 1 0 ppm
1.9
2.9
0.9
0.9
0.9
0.9
1.0
9.1

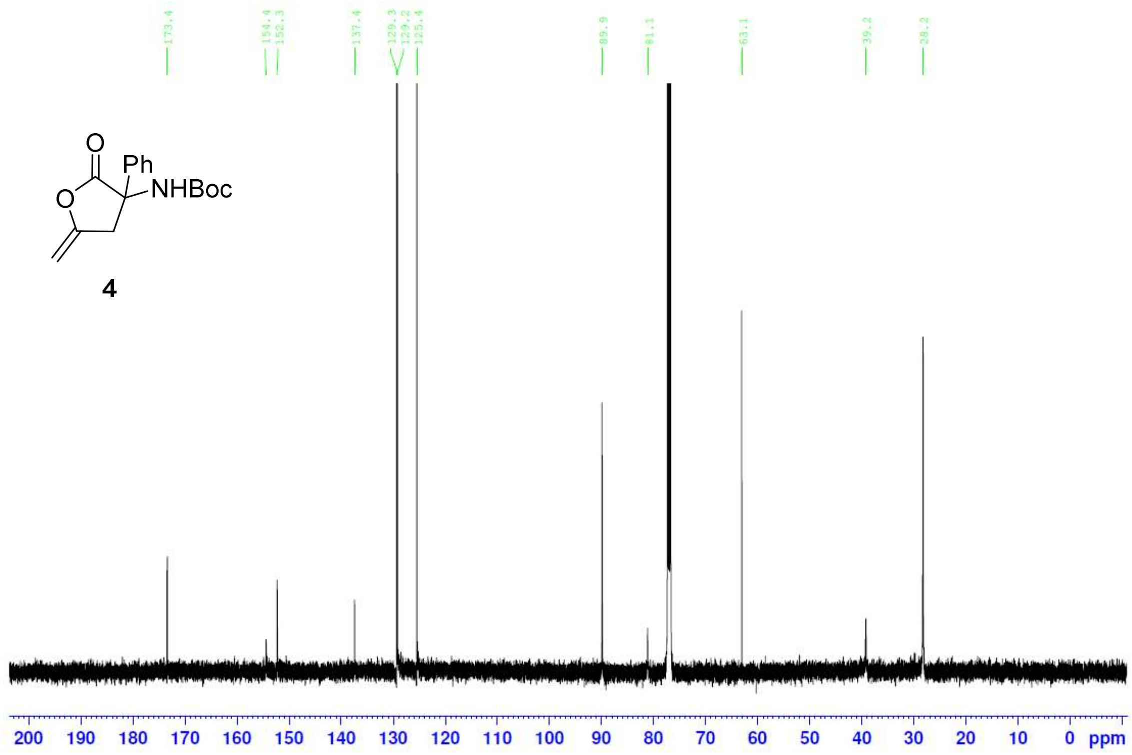
173.4
154.4
152.3
137.4
129.3
129.2
125.4
89.9
81.1
63.1
39.2
28.2
O
Ph
NHBoc
O
4
200 190 180 170 160 150 140 130 120 110 100 90 80 70 60 50 40 30 20 10 0 ppm

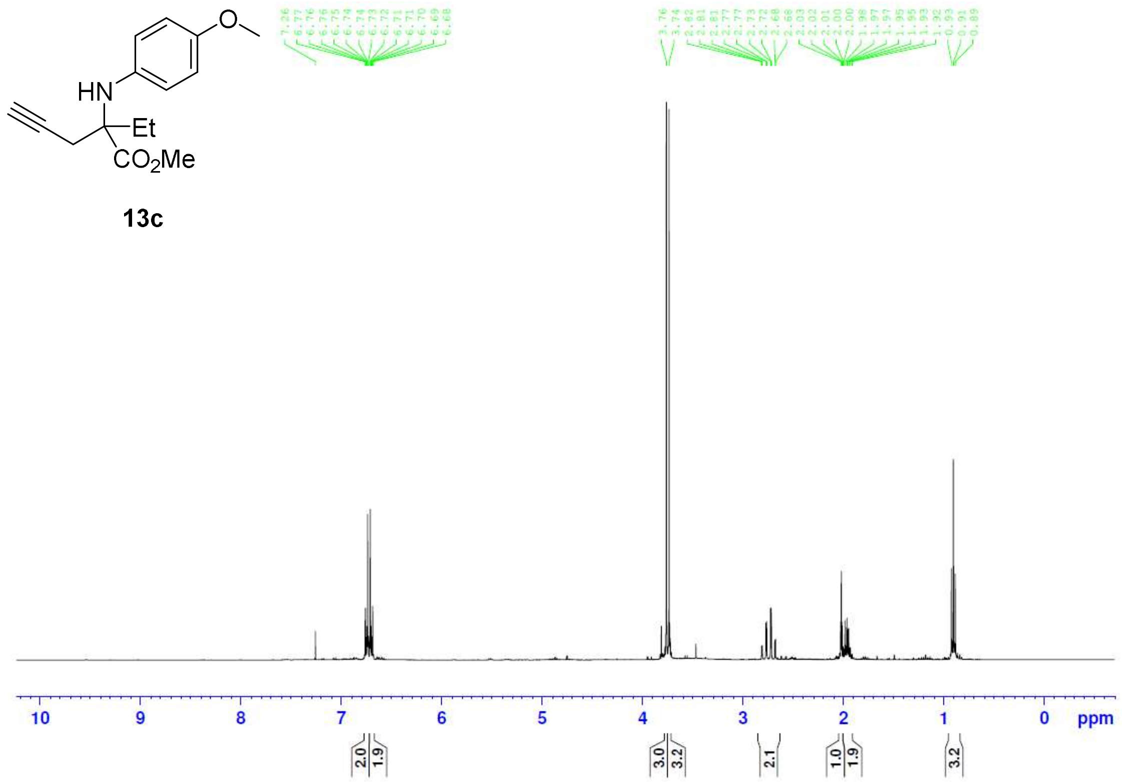
HN
Et
CO2Me
13c
2.0 1.9 3.0 3.2 2.1 1.0 1.9 3.2
10 9 8 7 6 5 4 3 2 1 0 ppm

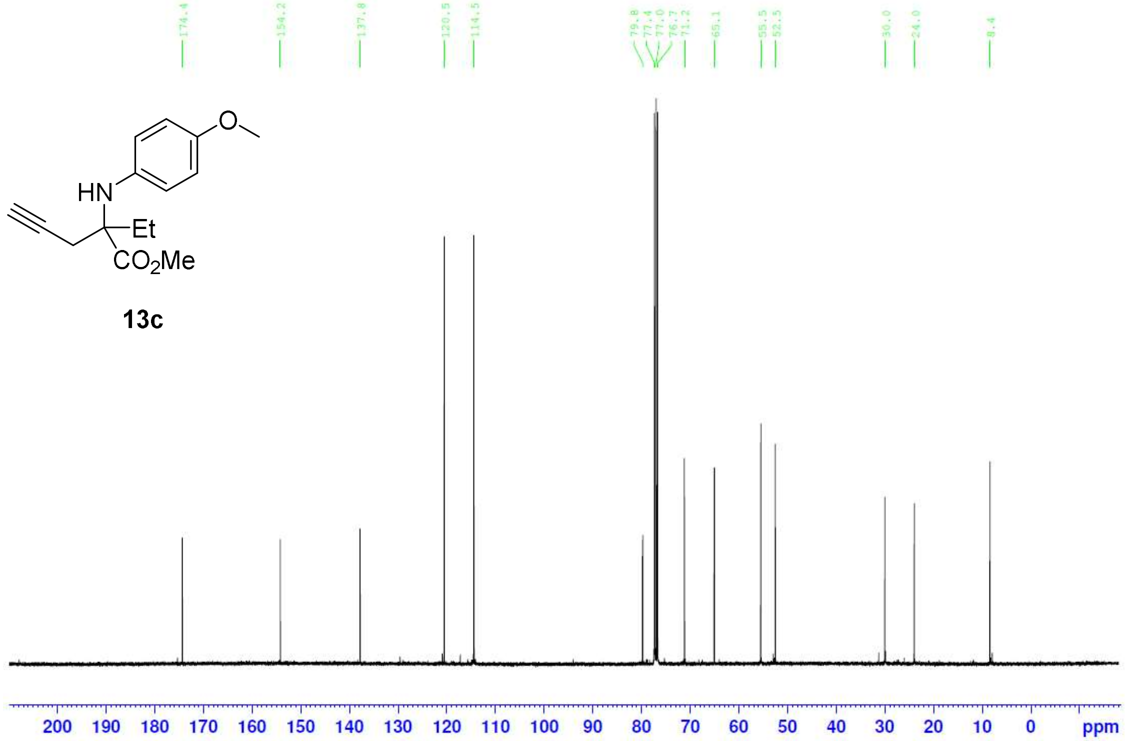
HN
Et
CO2Me
13c
200 190 180 170 160 150 140 130 120 110 100 90 80 70 60 50 40 30 20 10 0 ppm

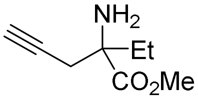

NH2
Et
CO2Me

15c

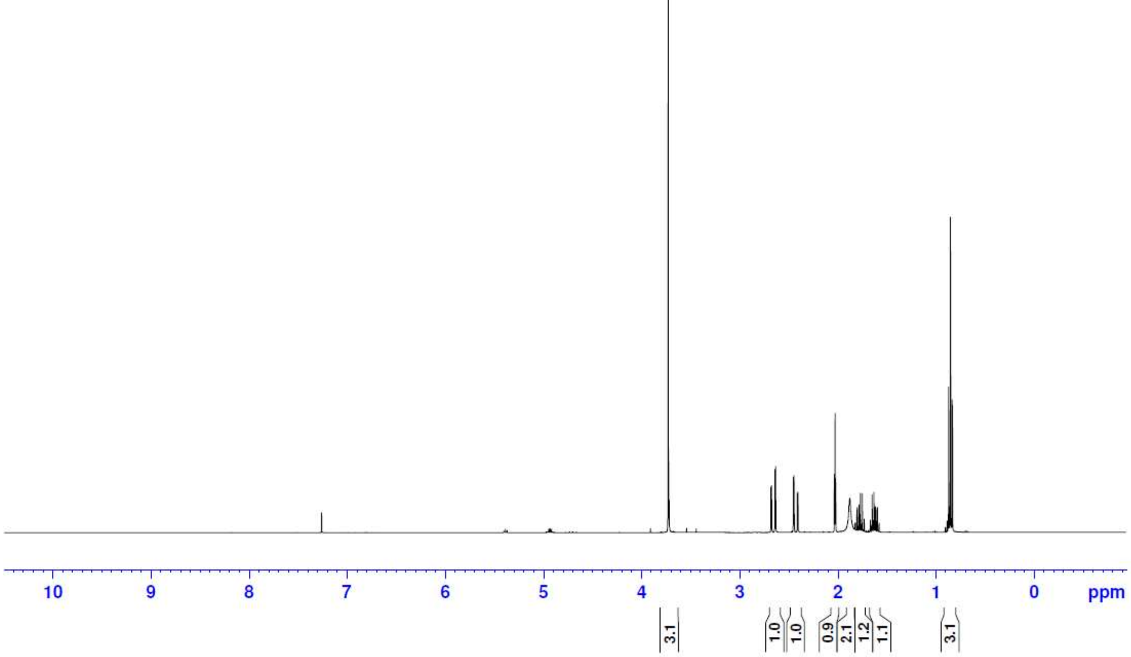

7.26
3.73
10 9 8 7 6 5 4 3 2 1 0 ppm
3.1
1.0
1.0
0.9
2.1
1.2
1.1
3.1

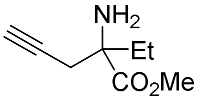

NH2
Et
CO2Me

15c

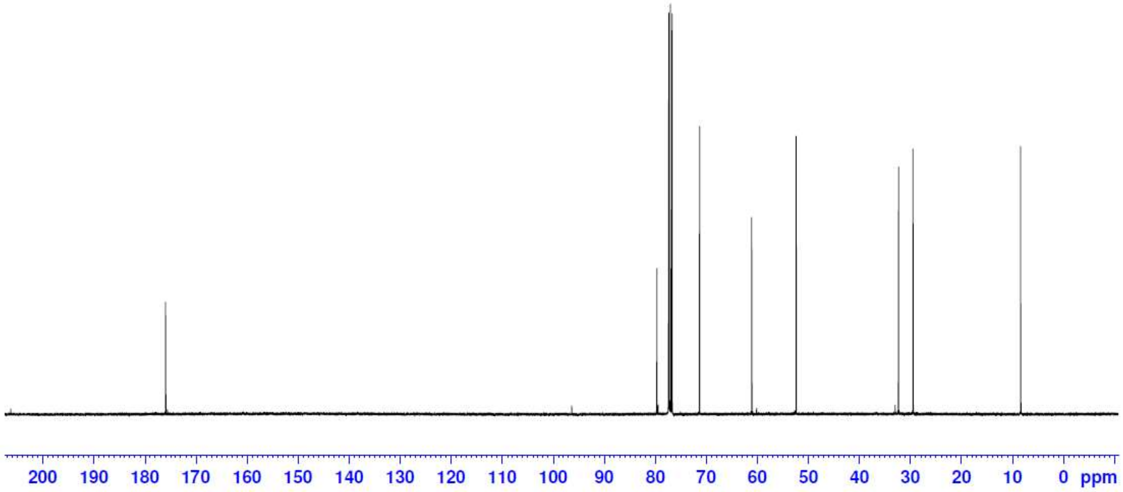

176.0
79.7
77.3
77.0
76.7
71.3
61.1
52.4
32.3
29.5
8.4
200 190 180 170 160 150 140 130 120 110 100 90 80 70 60 50 40 30 20 10 0 ppm

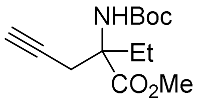

17c

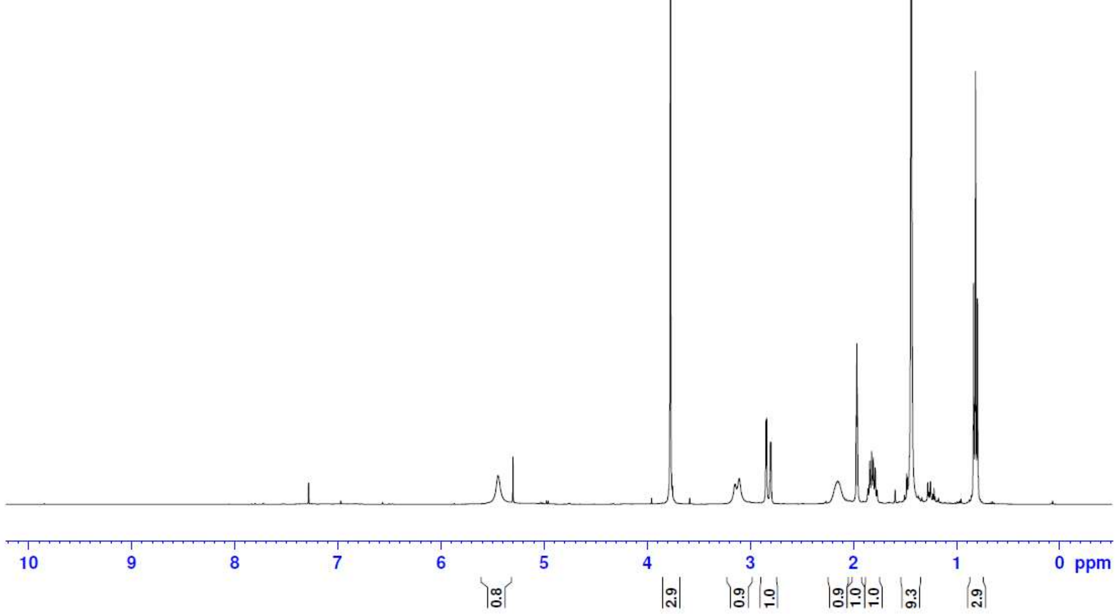

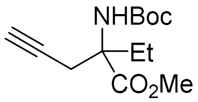

17c

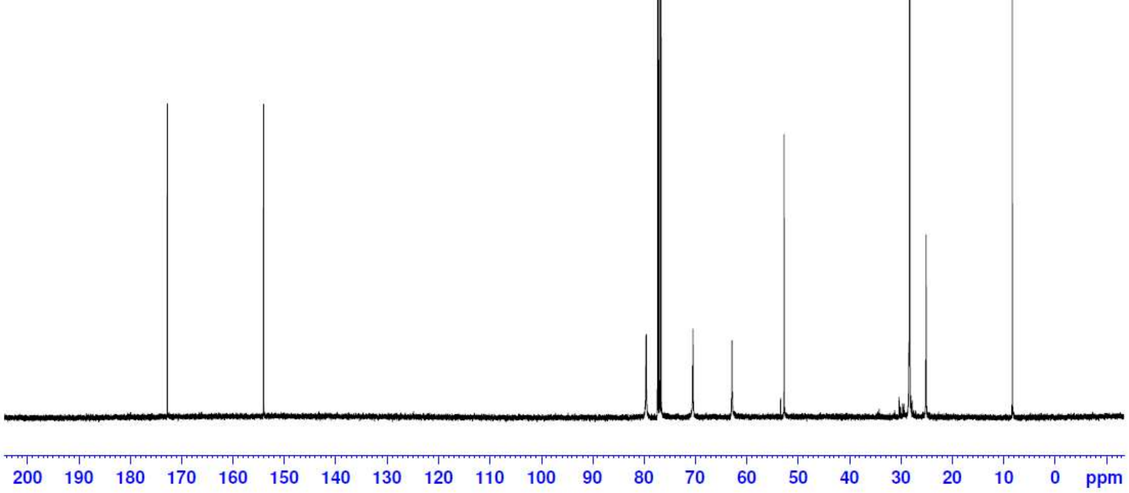

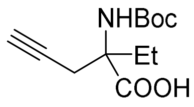

NHBoc
Et
COOH

18b

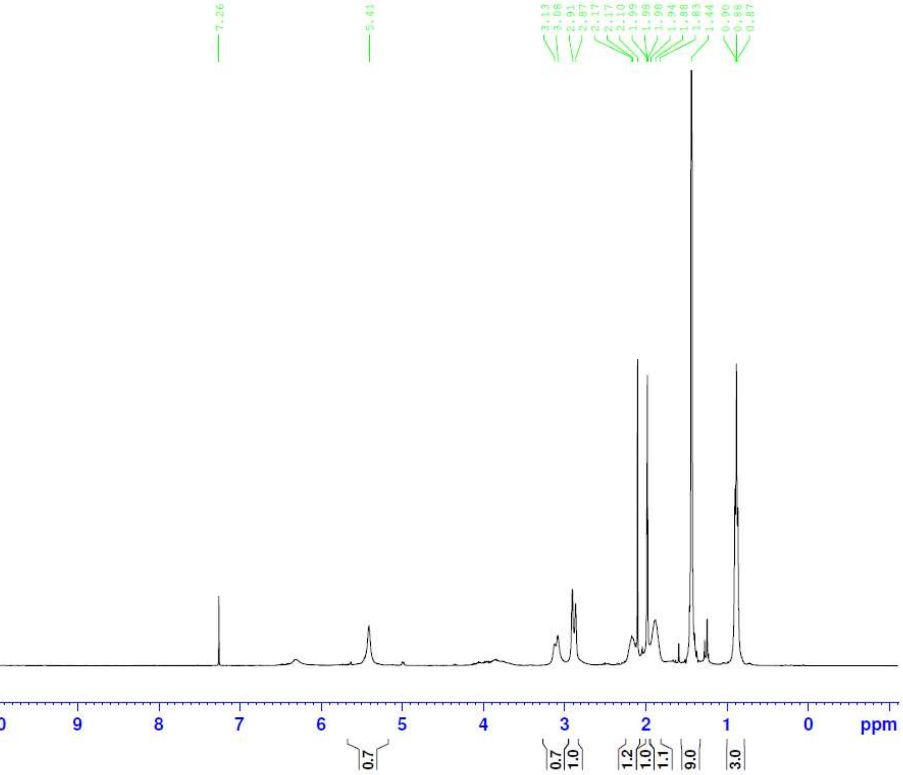

12 11 10 9 8 7 6 5 4 3 2 1 0 ppm
0.9
0.7
0.7 1.0
1.2 1.0 1.1 9.0 3.0

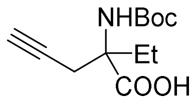

NHBoc
Et
COOH

18b

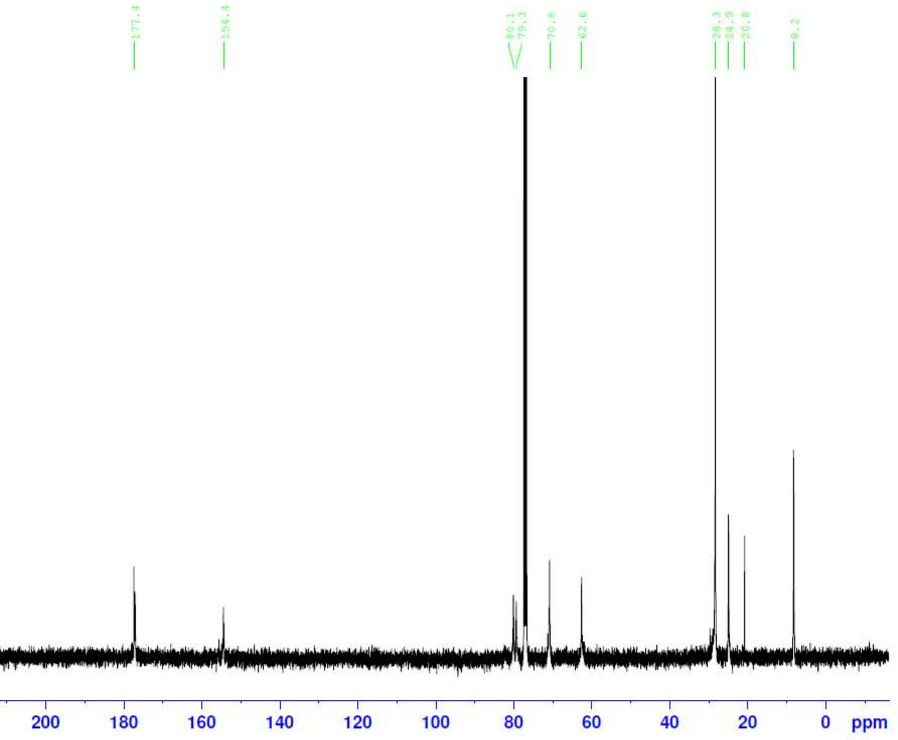

260 240 220 200 180 160 140 120 100 80 60 40 20 0 ppm

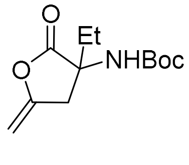
O
Et
NHBoc
O

3

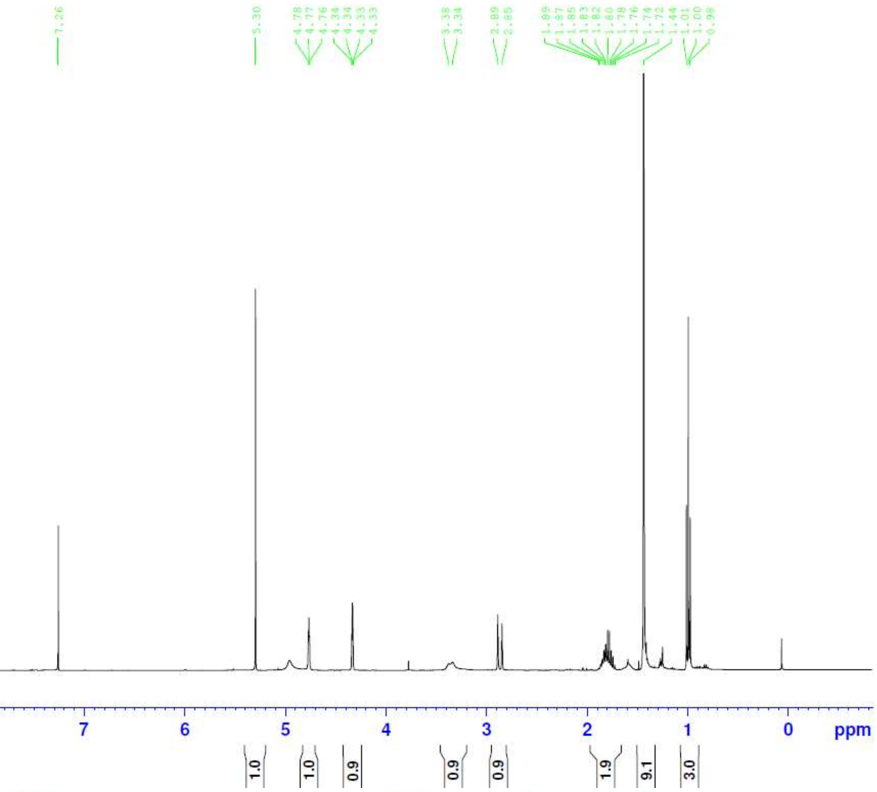
7.26
5.30
4.78
4.77
4.76
4.34
4.34
4.33
4.33
3.38
3.34
2.89
2.85
1.89
1.87
1.85
1.83
1.82
1.80
1.78
1.76
1.74
1.72
1.44
1.01
1.00
0.98
10
9
8
7
6
5
4
3
2
1
0
ppm
1.0
1.0
0.9
0.9
0.9
1.9
9.1
3.0

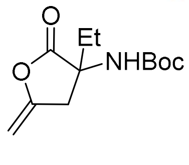
O
Et
NHBoc
O

3

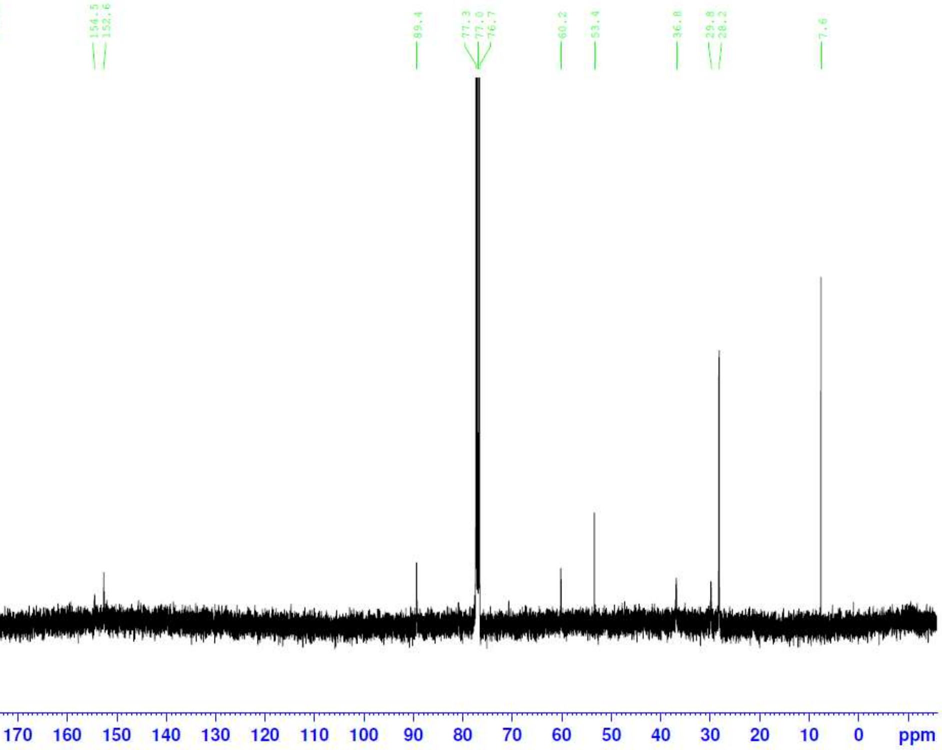
175.1
154.5
152.6
89.4
77.3
77.0
76.7
60.2
53.4
36.8
29.8
28.2
7.6
200
190
180
170
160
150
140
130
120
110
100
90
80
70
60
50
40
30
20
10
0
ppm

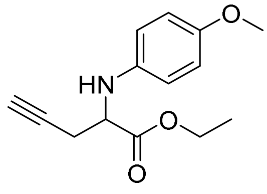
HN
O
O

13a

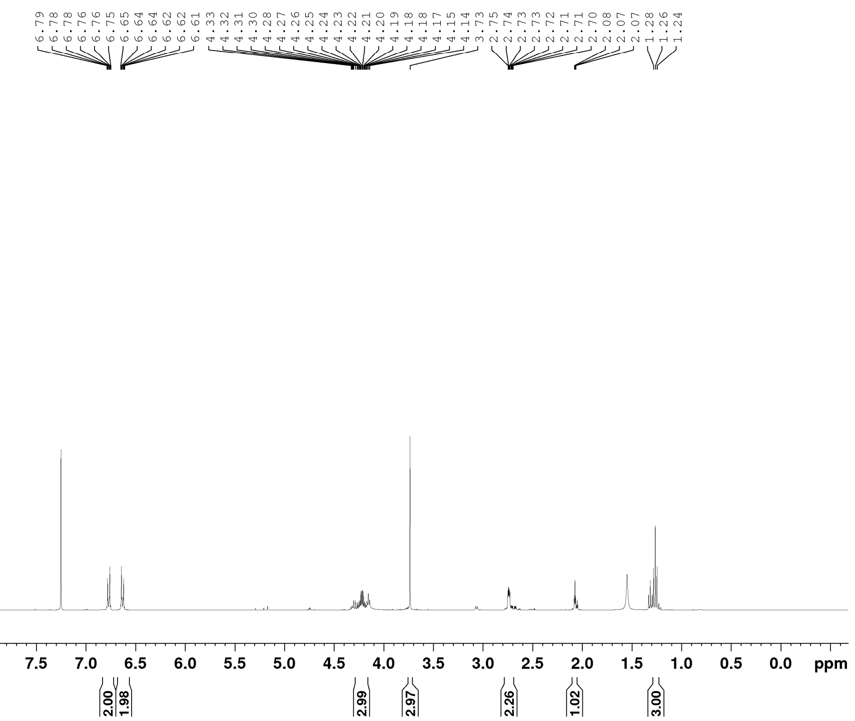
6.79 6.78 6.78 6.76 6.76 6.75 6.65 6.64 6.64 6.62 6.62 6.61 4.33 4.32 4.31 4.30 4.28 4.27 4.26 4.25 4.24 4.23 4.22 4.21 4.20 4.19 4.18 4.18 4.17 4.15 4.14 3.73 2.75 2.74 2.73 2.73 2.72 2.71 2.71 2.70 2.08 2.07 2.07 1.28 1.26 1.24
10.0 9.5 9.0 8.5 8.0 7.5 7.0 6.5 6.0 5.5 5.0 4.5 4.0 3.5 3.0 2.5 2.0 1.5 1.0 0.5 0.0 ppm
2.00 1.98 2.99 2.97 2.26 1.02 3.00

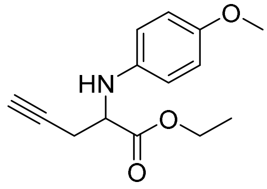
HN
O
O

13a

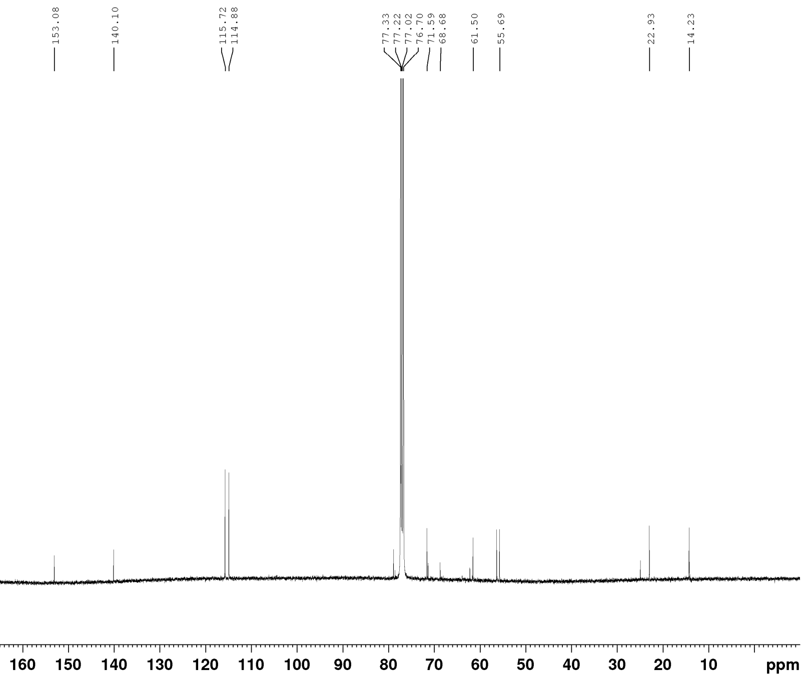
172.14 153.08 140.10 115.72 114.88 77.33 77.22 77.02 76.70 71.59 68.68 61.50 55.69 22.93 14.23
200 190 180 170 160 150 140 130 120 110 100 90 80 70 60 50 40 30 20 10 ppm

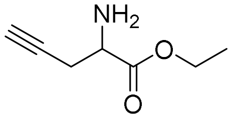
NH2
O
O

15a

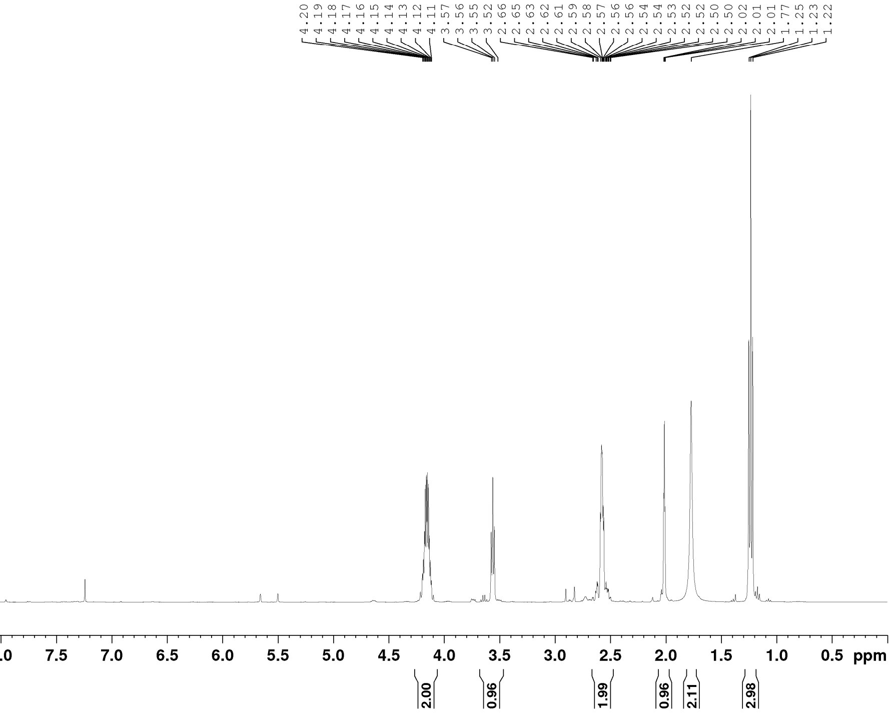
4.20 4.19 4.18 4.17 4.16 4.15 4.14 4.13 4.12 4.11 3.57 3.56 3.55 3.52 2.66 2.65 2.63 2.62 2.61 2.59 2.58 2.57 2.56 2.54 2.54 2.53 2.52 2.52 2.50 2.50 2.02 2.01 2.01 1.77 1.25 1.23 1.22
9.5 9.0 8.5 8.0 7.5 7.0 6.5 6.0 5.5 5.0 4.5 4.0 3.5 3.0 2.5 2.0 1.5 1.0 0.5 ppm
2.00 0.96 1.99 0.96 2.11 2.98

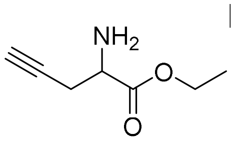
NH2
O
O

15a

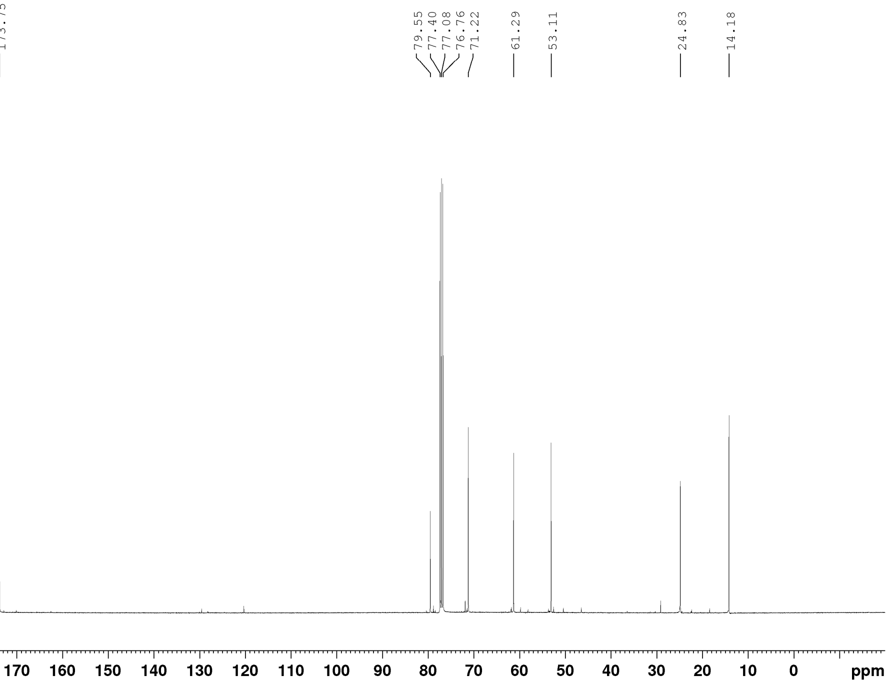
173.75
79.55 77.40 77.08 76.76 71.22
61.29
53.11
24.83
14.18
210 200 190 180 170 160 150 140 130 120 110 100 90 80 70 60 50 40 30 20 10 0 ppm

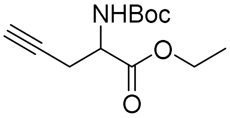
NHBoc
O
O

17e

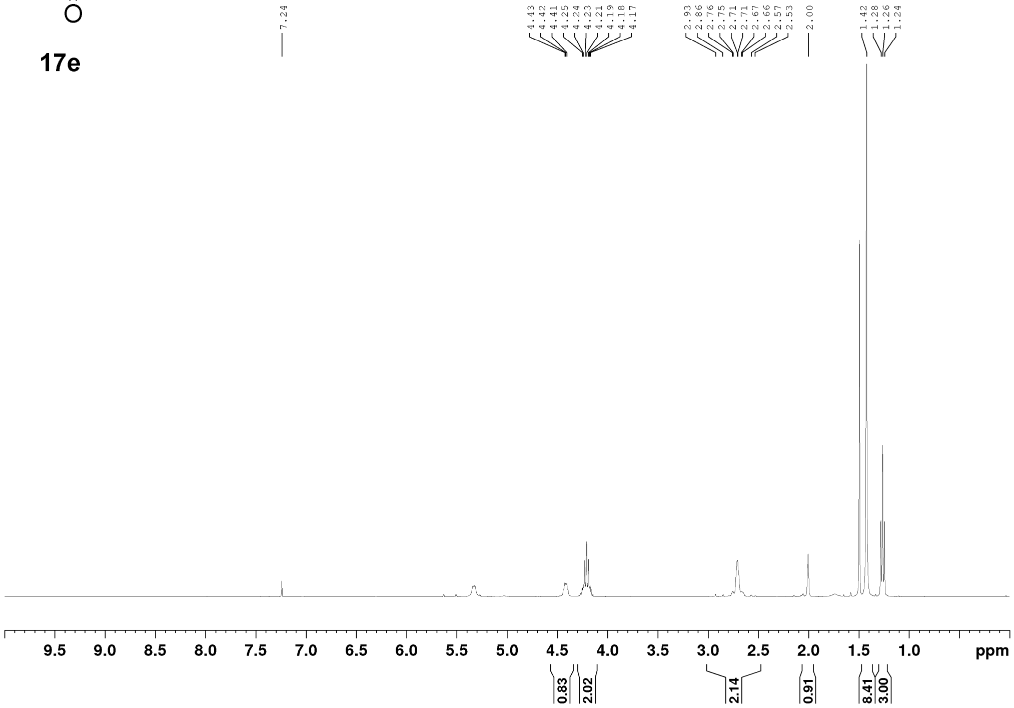
7.24
4.43 4.42 4.41 4.25 4.24 4.23 4.21 4.19 4.18 4.17
2.93 2.86 2.76 2.75 2.71 2.71 2.67 2.66 2.57 2.53
2.00
1.42 1.28 1.26 1.24
9.5 9.0 8.5 8.0 7.5 7.0 6.5 6.0 5.5 5.0 4.5 4.0 3.5 3.0 2.5 2.0 1.5 1.0 ppm
0.83 2.02 2.14 0.91 8.41 3.00

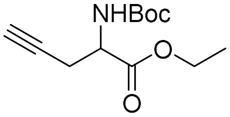
NHBoc
O
O

17e

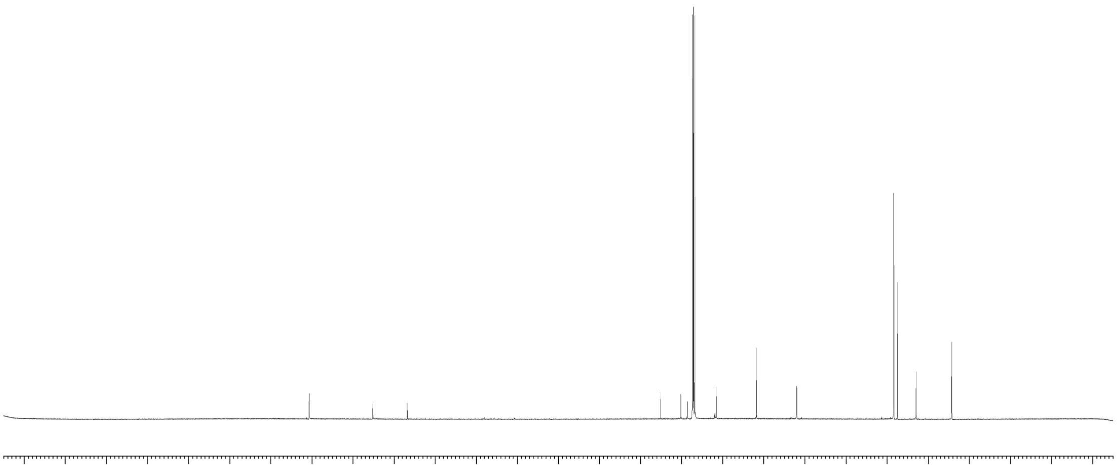
170.60
155.11
80.12 78.57
71.52
61.76
51.91
28.28
22.86
14.17
240 230 220 210 200 190 180 170 160 150 140 130 120 110 100 90 80 70 60 50 40 30 20 10 0 -10 ppm

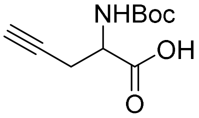
NHBoc
OH
O

18c

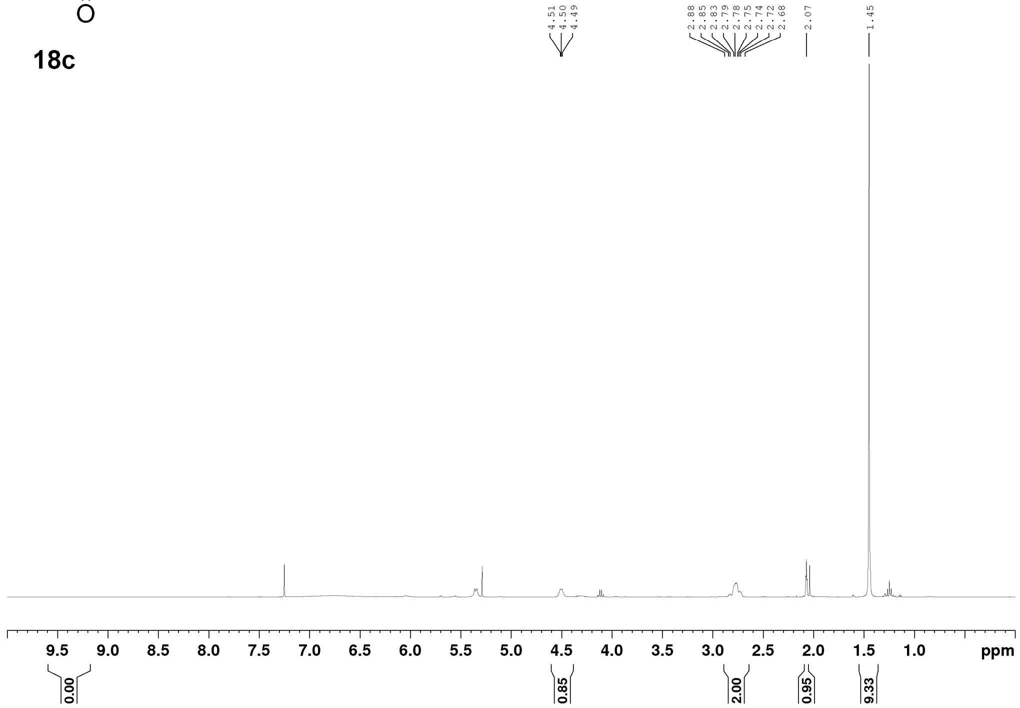
4.51
4.50
4.49
2.88
2.85
2.83
2.79
2.78
2.75
2.74
2.72
2.68
2.07
1.45
9.5 9.0 8.5 8.0 7.5 7.0 6.5 6.0 5.5 5.0 4.5 4.0 3.5 3.0 2.5 2.0 1.5 1.0 ppm
0.00
0.85
2.00
0.95
9.33

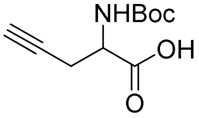
NHBoc
OH
O

18c

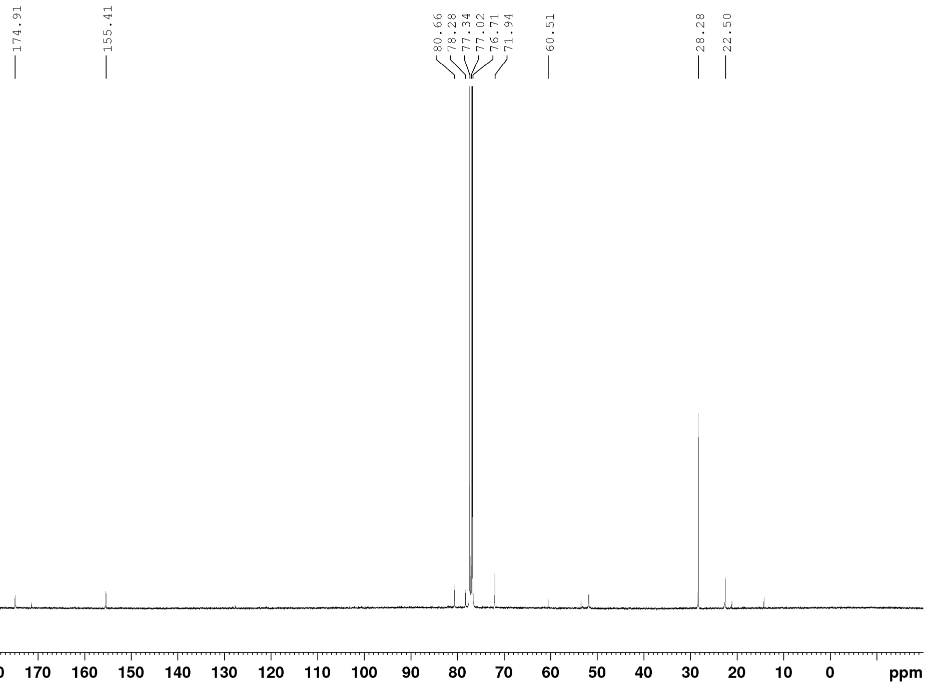
174.91
155.41
80.66
78.28
77.34
77.02
76.71
71.94
60.51
28.28
22.50
210 200 190 180 170 160 150 140 130 120 110 100 90 80 70 60 50 40 30 20 10 0 ppm

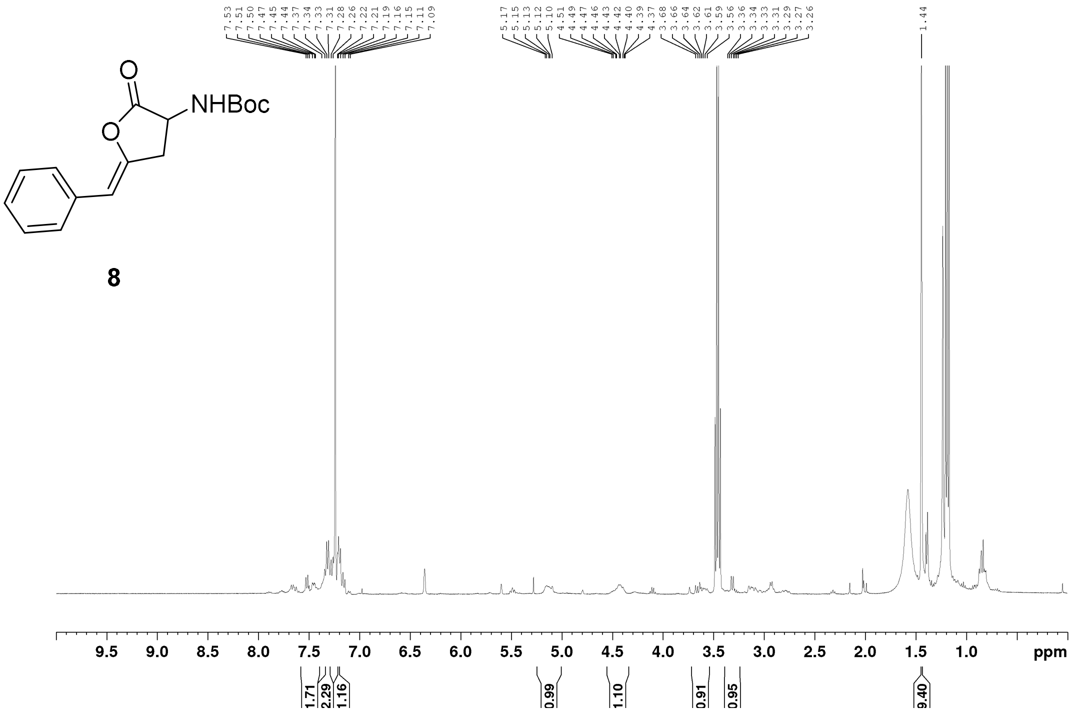
NHBoc
O
O
7.53 7.51 7.50 7.47 7.45 7.44 7.37 7.34 7.33 7.31 7.28 7.26 7.22 7.21 7.19 7.16 7.15 7.11 7.09
5.17 5.15 5.13 5.12 5.10 4.51 4.49 4.47 4.46 4.43 4.42 4.40 4.39 4.37 3.68 3.66 3.64 3.62 3.61 3.59 3.56 3.36 3.34 3.33 3.31 3.29 3.27 3.26
1.44
9.5 9.0 8.5 8.0 7.5 7.0 6.5 6.0 5.5 5.0 4.5 4.0 3.5 3.0 2.5 2.0 1.5 1.0 ppm
1.71 2.29 1.16 0.99 1.10 0.91 0.95 9.40

8

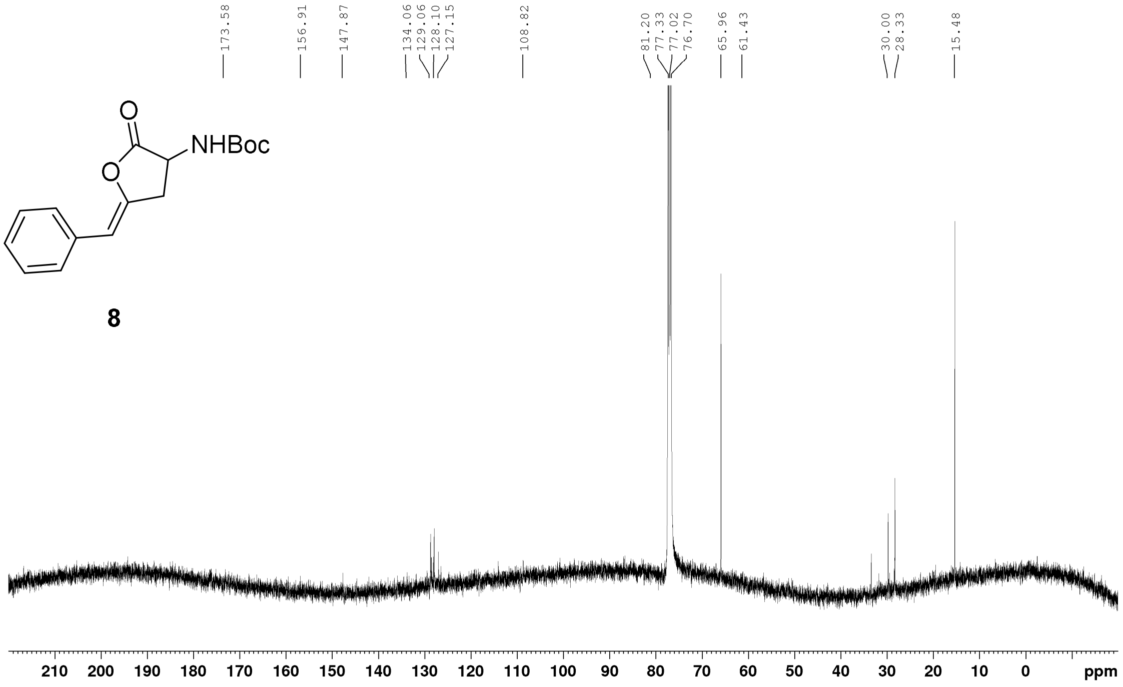
NHBoc
O
O
173.58
156.91
147.87
134.06 129.06 128.10 127.15
108.82
81.20 77.33 77.02 76.70
65.96 61.43
30.00 28.33
15.48
210 200 190 180 170 160 150 140 130 120 110 100 90 80 70 60 50 40 30 20 10 0 ppm

8

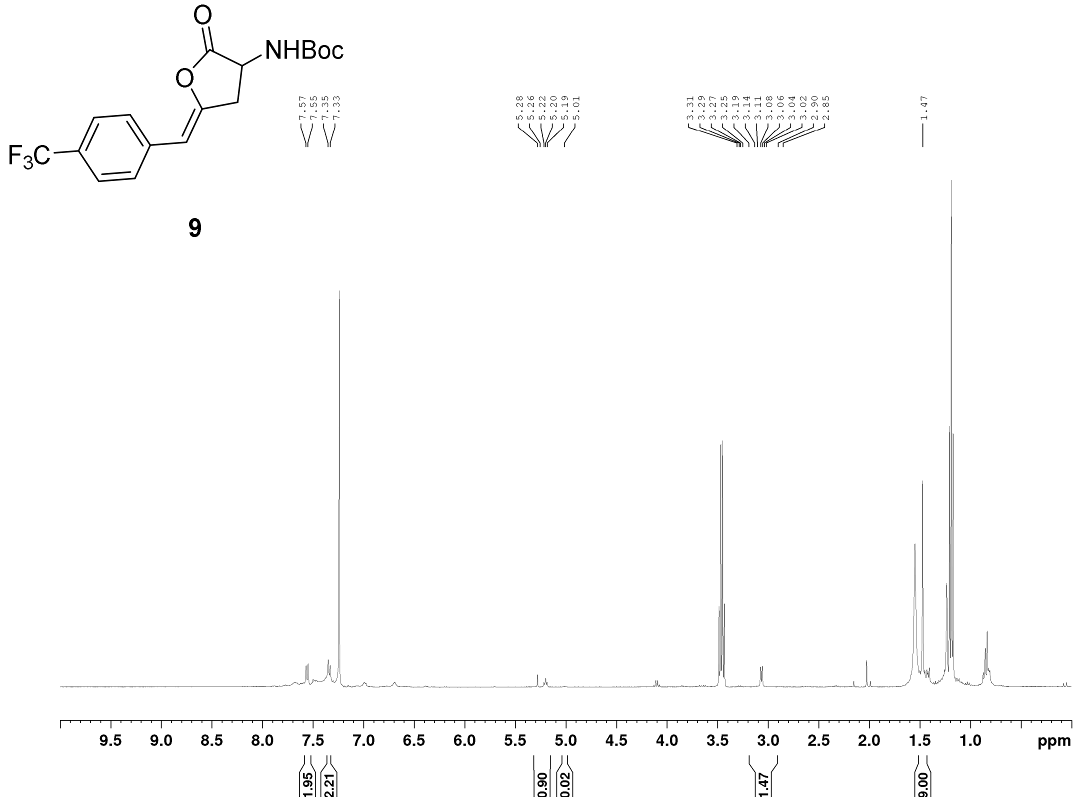
NHBoc
O
O
F3C
9
7.57
7.55
7.35
7.33
5.28
5.26
5.22
5.20
5.19
5.01
3.31
3.29
3.27
3.25
3.19
3.14
3.11
3.08
3.06
3.04
3.02
2.90
2.85
1.47
9.5 9.0 8.5 8.0 7.5 7.0 6.5 6.0 5.5 5.0 4.5 4.0 3.5 3.0 2.5 2.0 1.5 1.0 ppm
1.95
2.21
0.90
0.02
1.47
9.00

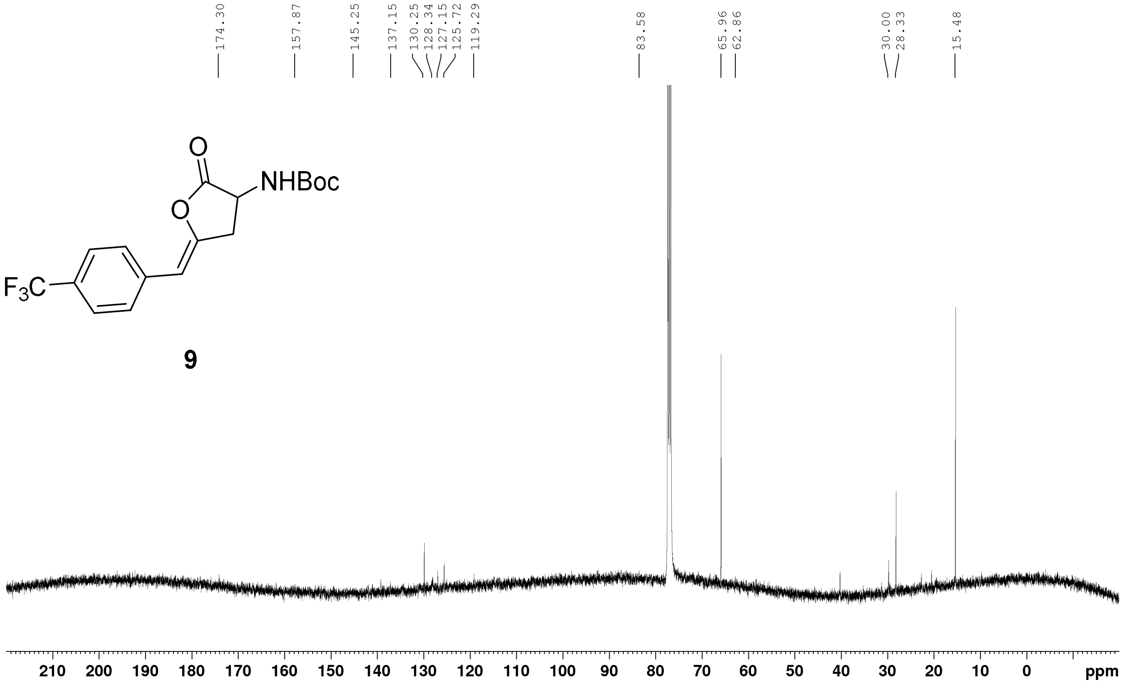
174.30
157.87
145.25
137.15
130.25
128.34
127.15
125.72
119.29
83.58
65.96
62.86
30.00
28.33
15.48
NHBoc
O
O
F3C
9
210 200 190 180 170 160 150 140 130 120 110 100 90 80 70 60 50 40 30 20 10 0 ppm

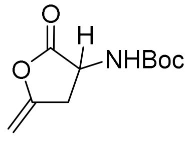

O
H
NHBoc
O

2

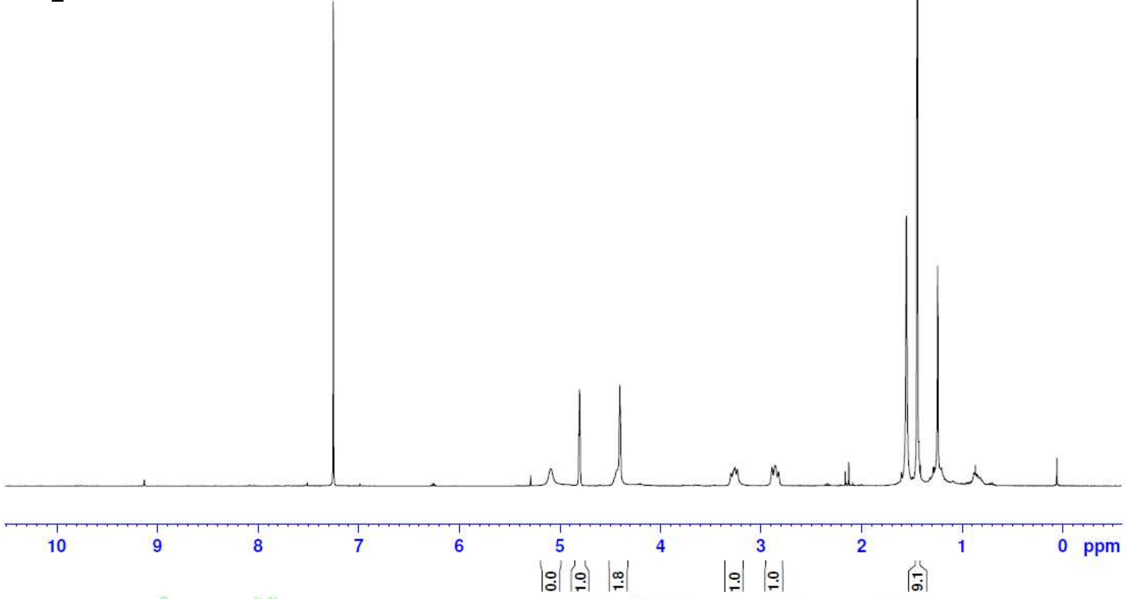

7.25
5.09
4.81
4.80
4.80
4.44
4.40
3.30
3.27
3.26
3.24
2.89
2.86
2.83
1.56
1.45
1.25
10 9 8 7 6 5 4 3 2 1 0 ppm
0.0
1.0
1.8
1.0
1.0
9.1

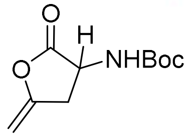

O
H
NHBoc
O

2

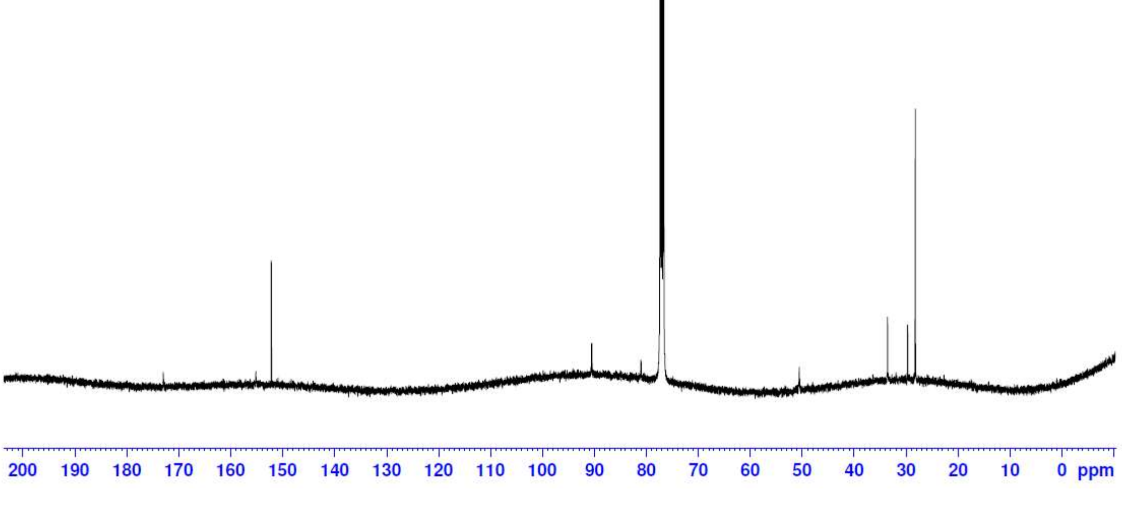

173.0
155.2
152.2
90.5
81.0
77.3
77.2
77.0
76.7
50.5
33.6
29.7
28.2
200 190 180 170 160 150 140 130 120 110 100 90 80 70 60 50 40 30 20 10 0 ppm

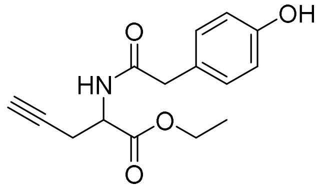
OH
O
HN
O
O

17f

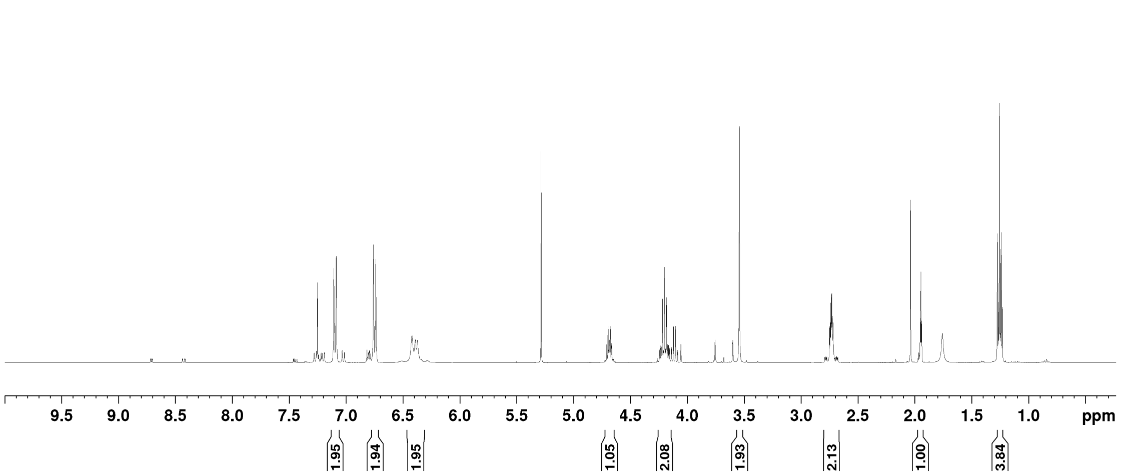
7.11
7.09
6.76
6.74
6.42
6.39
6.37
4.71
4.70
4.69
4.68
4.68
4.67
4.25
4.24
4.23
4.22
4.21
4.20
4.19
4.18
4.17
4.17
4.16
3.54
2.79
2.79
2.78
2.77
2.75
2.74
2.74
2.73
2.72
2.72
2.70
2.69
2.69
2.68
2.68
1.95
1.95
1.94
1.27
1.26
1.24
9.5
9.0
8.5
8.0
7.5
7.0
6.5
6.0
5.5
5.0
4.5
4.0
3.5
3.0
2.5
2.0
1.5
1.0
ppm
1.95
1.94
1.95
1.05
2.08
1.93
2.13
1.00
3.84

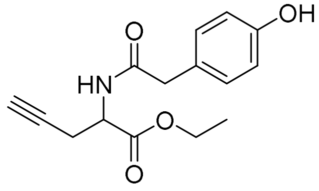
OH
O
HN
O
O

17f

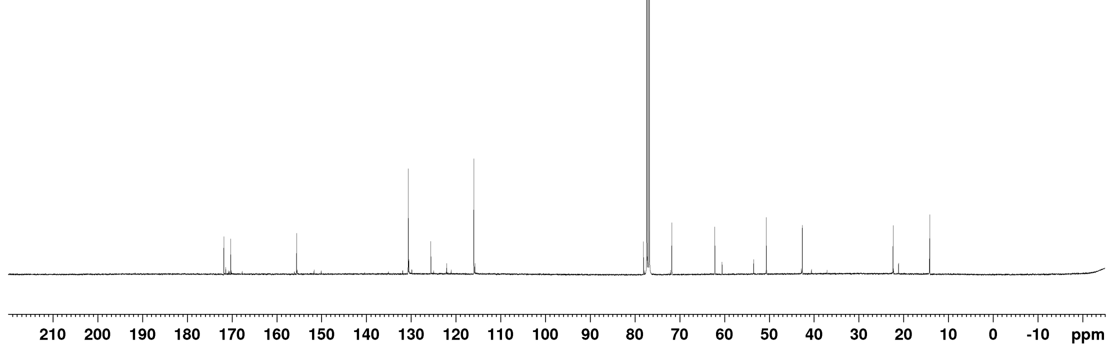
171.81
170.29
155.55
130.65
125.57
115.94
78.07
77.35
77.04
76.72
71.72
62.12
50.64
42.58
22.30
14.09
210
200
190
180
170
160
150
140
130
120
110
100
90
80
70
60
50
40
30
20
10
0
-10
ppm

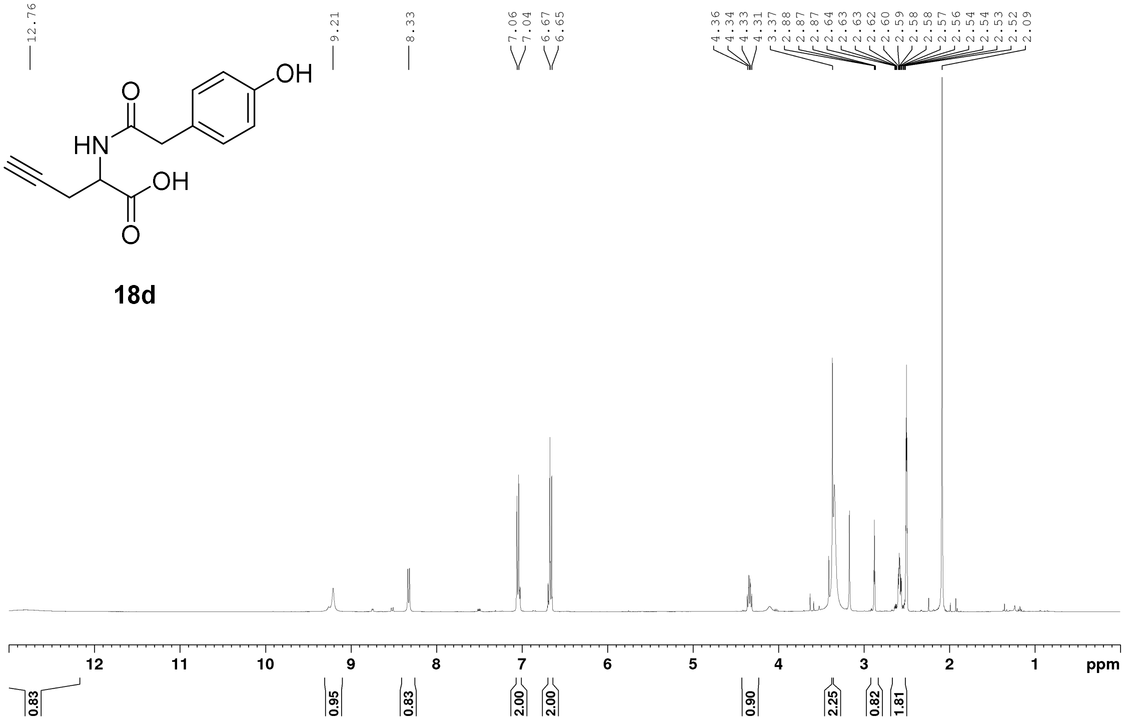
12.76
9.21
8.33
7.06
7.04
6.67
6.65
4.36
4.34
4.33
4.31
3.37
2.88
2.87
2.87
2.64
2.63
2.63
2.62
2.60
2.59
2.58
2.58
2.57
2.56
2.54
2.54
2.53
2.52
2.09
OH
O
HN
OH
O
12
11
10
9
8
7
6
5
4
3
2
1
ppm
0.83
0.95
0.83
2.00
2.00
0.90
2.25
0.82
1.81

18d

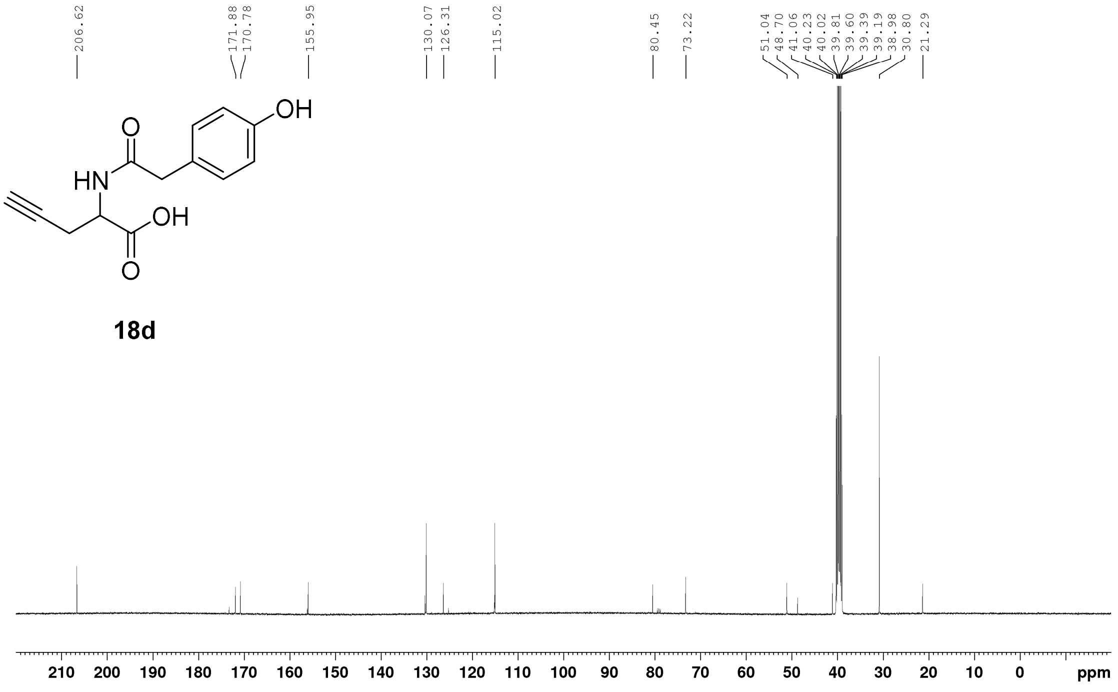
206.62
171.88
170.78
155.95
130.07
126.31
115.02
80.45
73.22
51.04
48.70
41.06
40.23
40.02
39.81
39.60
39.39
39.19
38.98
30.80
21.29
OH
O
HN
OH
O
210
200
190
180
170
160
150
140
130
120
110
100
90
80
70
60
50
40
30
20
10
0
ppm

18d

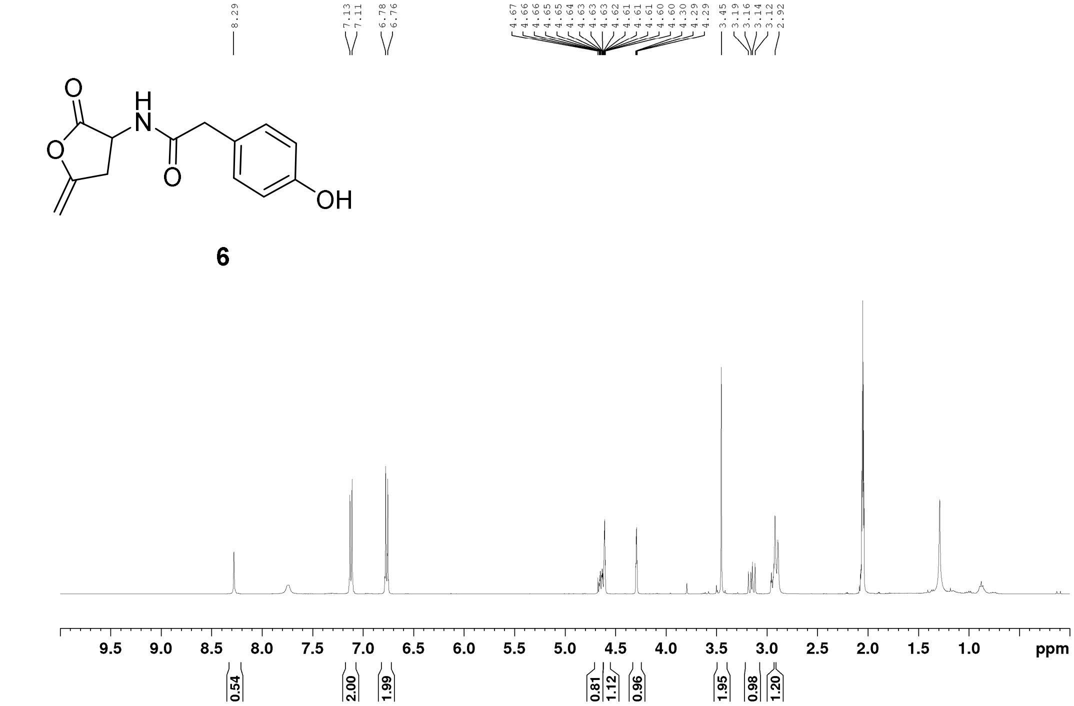
8.29
7.13
7.11
6.78
6.76
4.67
4.66
4.66
4.65
4.65
4.64
4.63
4.63
4.62
4.61
4.61
4.61
4.60
4.30
4.29
4.29
3.45
3.19
3.16
3.14
3.12
2.92
O
H
N
O
O
OH
6
ppm
0.54
2.00
1.99
0.81
1.12
0.96
1.95
0.98
1.20

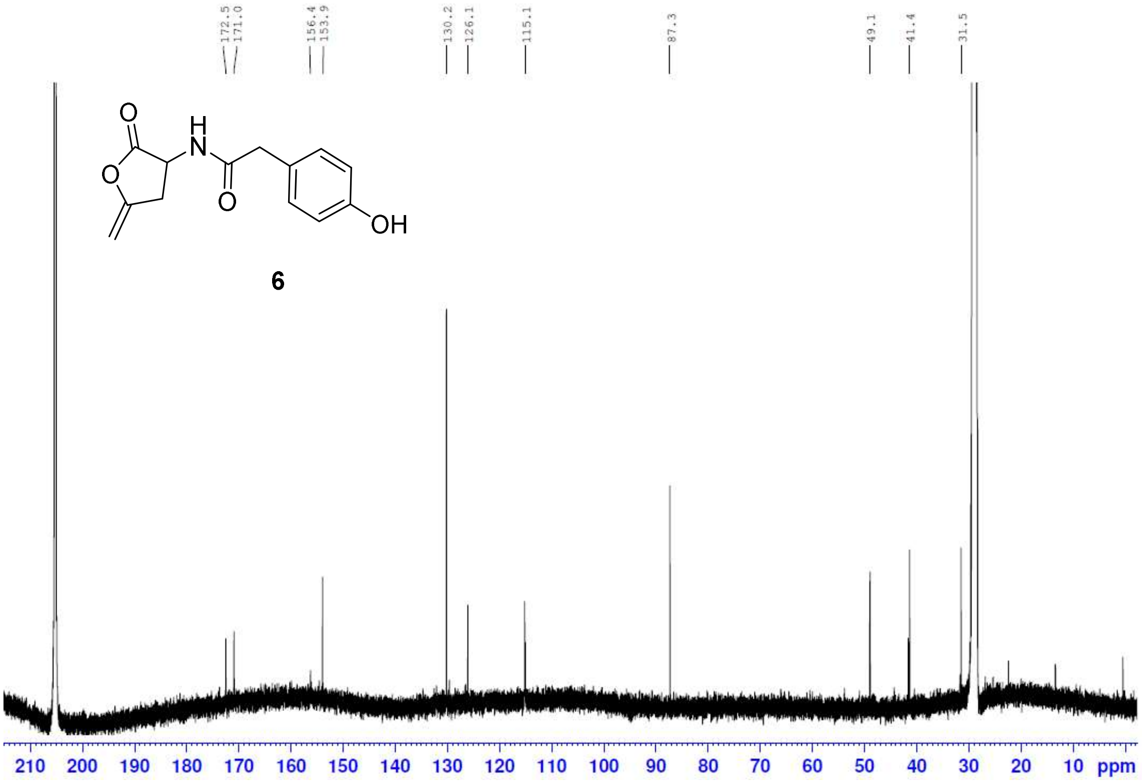
172.5
171.0
156.4
153.9
130.2
126.1
115.1
87.3
49.1
41.4
31.5
O
H
N
O
O
OH
6
ppm

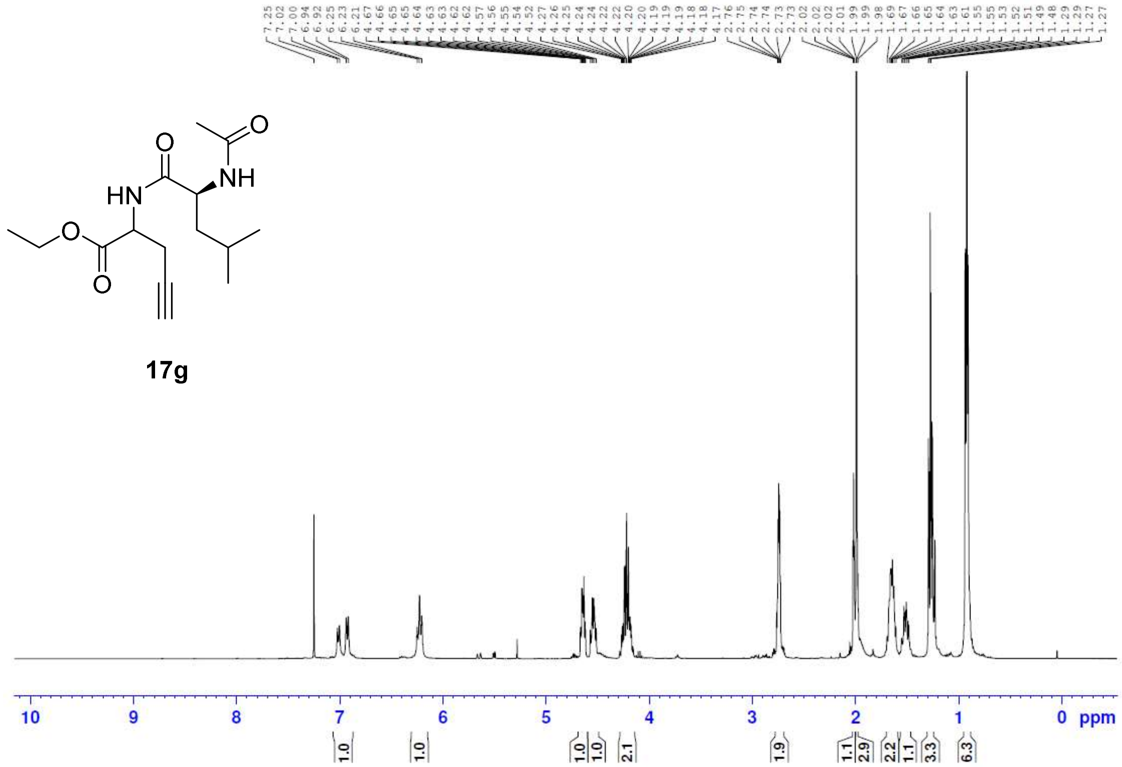
O
HN
NH
O
O
O
17g
10 9 8 7 6 5 4 3 2 1 0 ppm
1.0 1.0 1.0 1.0 2.1 1.9 1.1 2.9 2.2 1.1 3.3 6.3

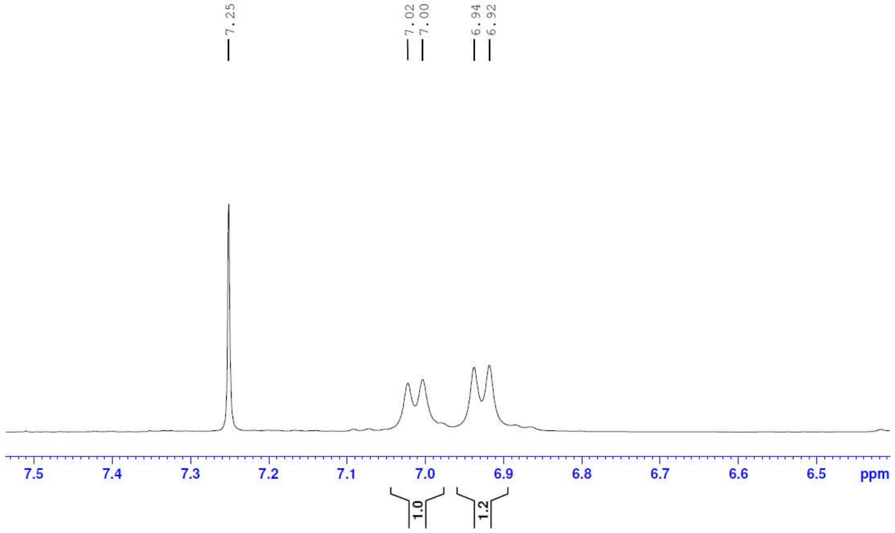
7.25
7.02 7.00
6.94 6.92
7.5 7.4 7.3 7.2 7.1 7.0 6.9 6.8 6.7 6.6 6.5 ppm
1.0 1.2

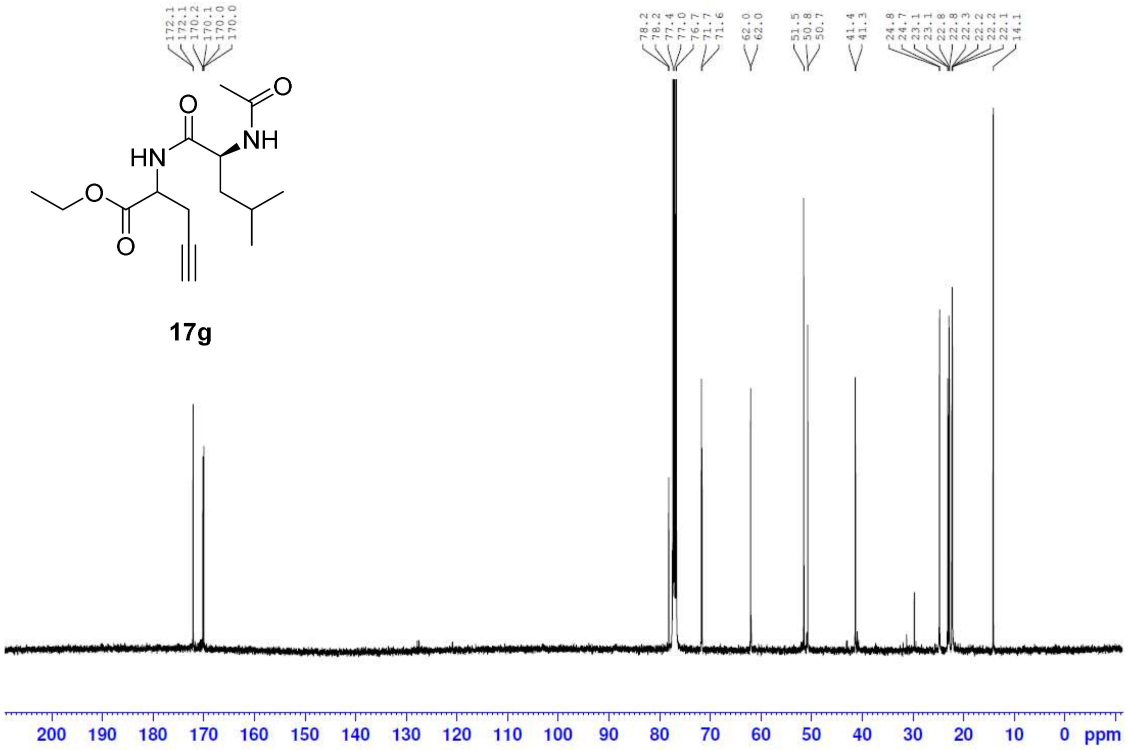
172.1
172.1
170.2
170.1
170.0
170.0
78.2
78.2
77.4
77.0
76.7
71.7
71.6
62.0
62.0
51.5
50.8
50.7
41.4
41.3
24.8
24.7
23.1
23.1
22.8
22.8
22.3
22.2
22.1
14.1
HN
NH
O
17g
200
190
180
170
160
150
140
130
120
110
100
90
80
70
60
50
40
30
20
10
0
ppm

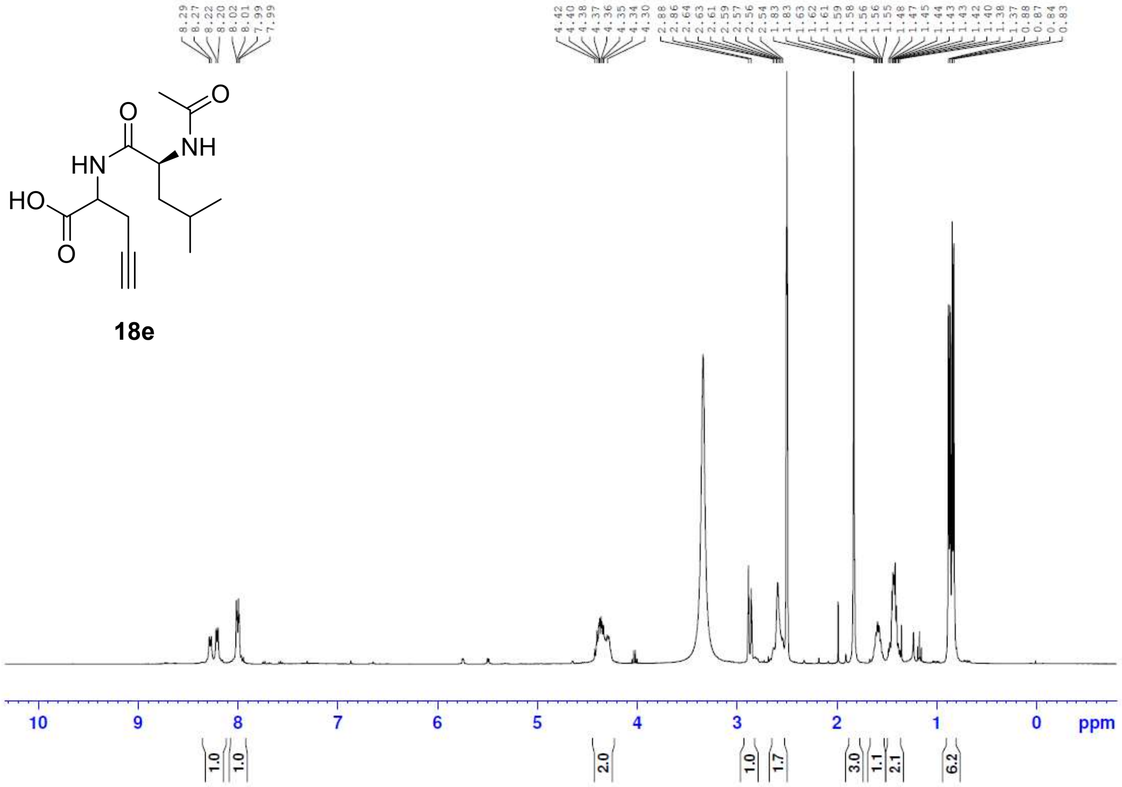

18e

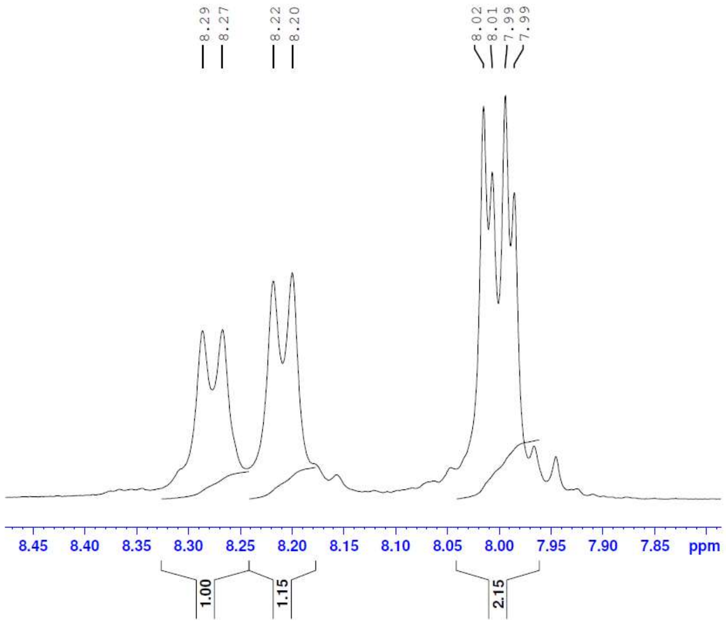

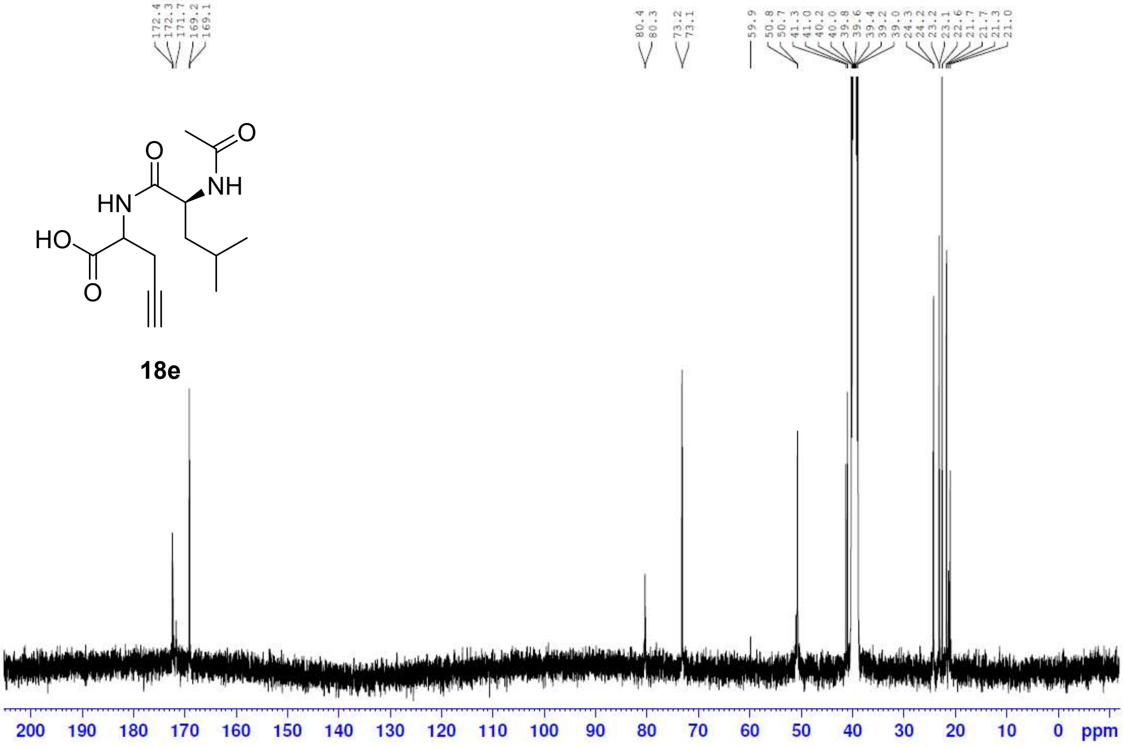

172.4
172.3
171.7
169.2
169.1
80.4
80.3
73.2
73.1
59.9
50.8
50.7
41.3
41.0
40.2
40.0
39.8
39.6
39.4
39.2
39.0
24.3
24.2
23.2
23.1
22.6
21.7
21.3
21.0
HO
HN
O
NH
18e
200 190 180 170 160 150 140 130 120 110 100 90 80 70 60 50 40 30 20 10 0 ppm

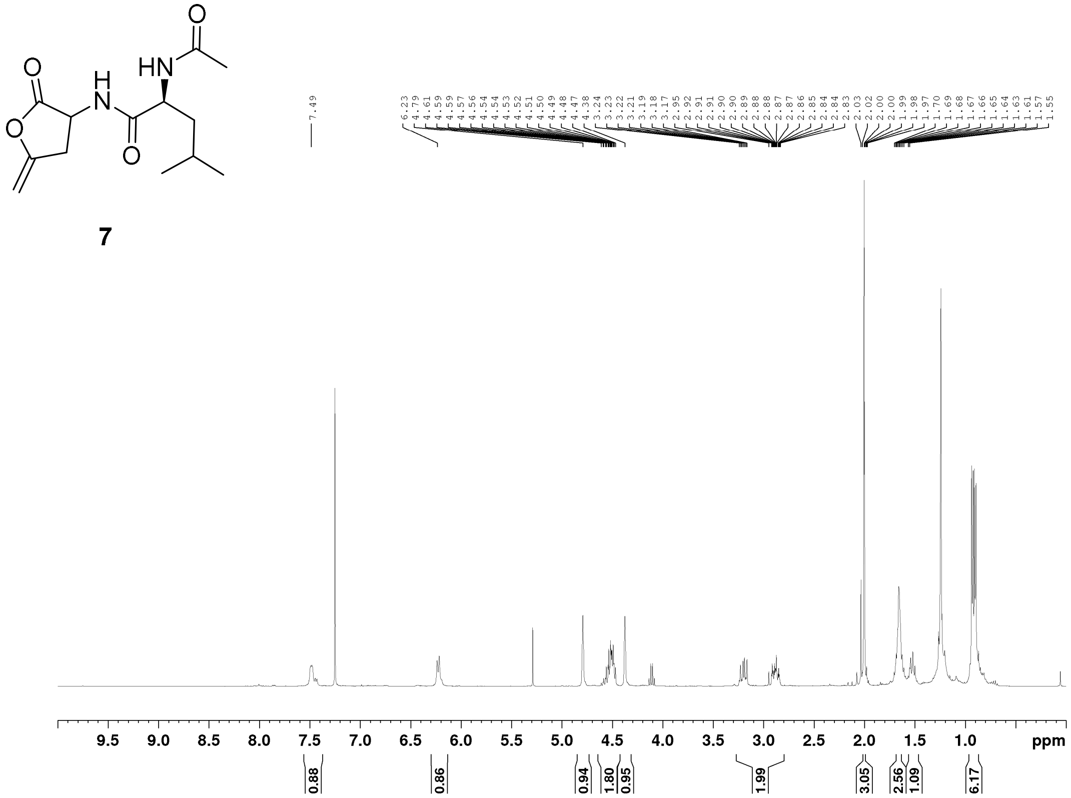

7

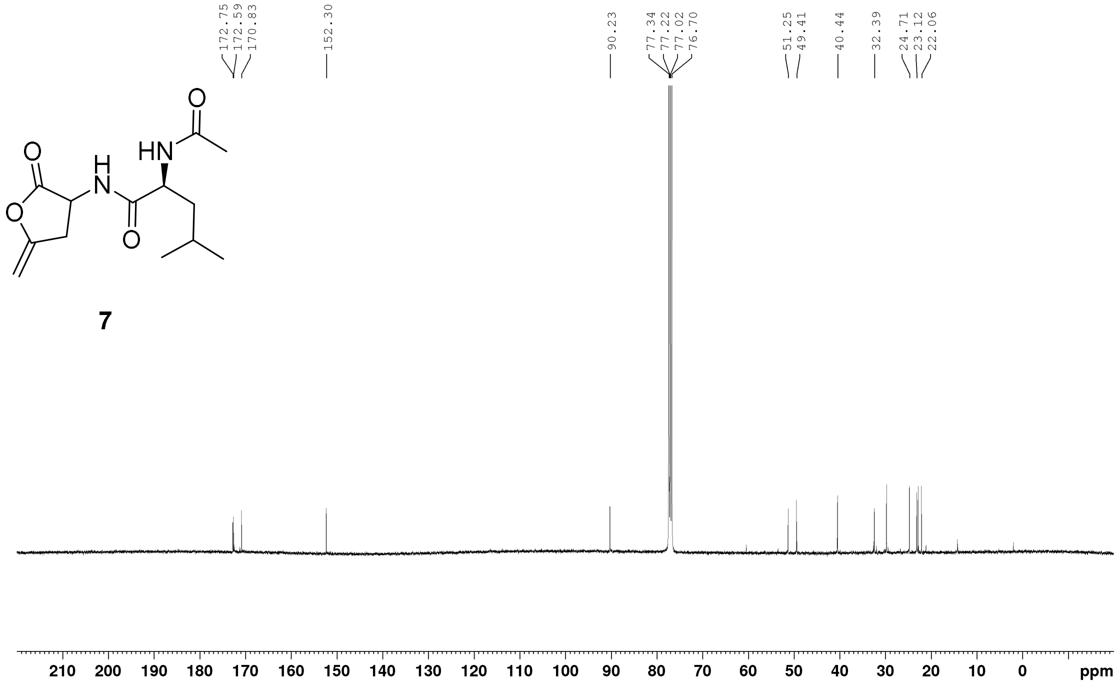

7

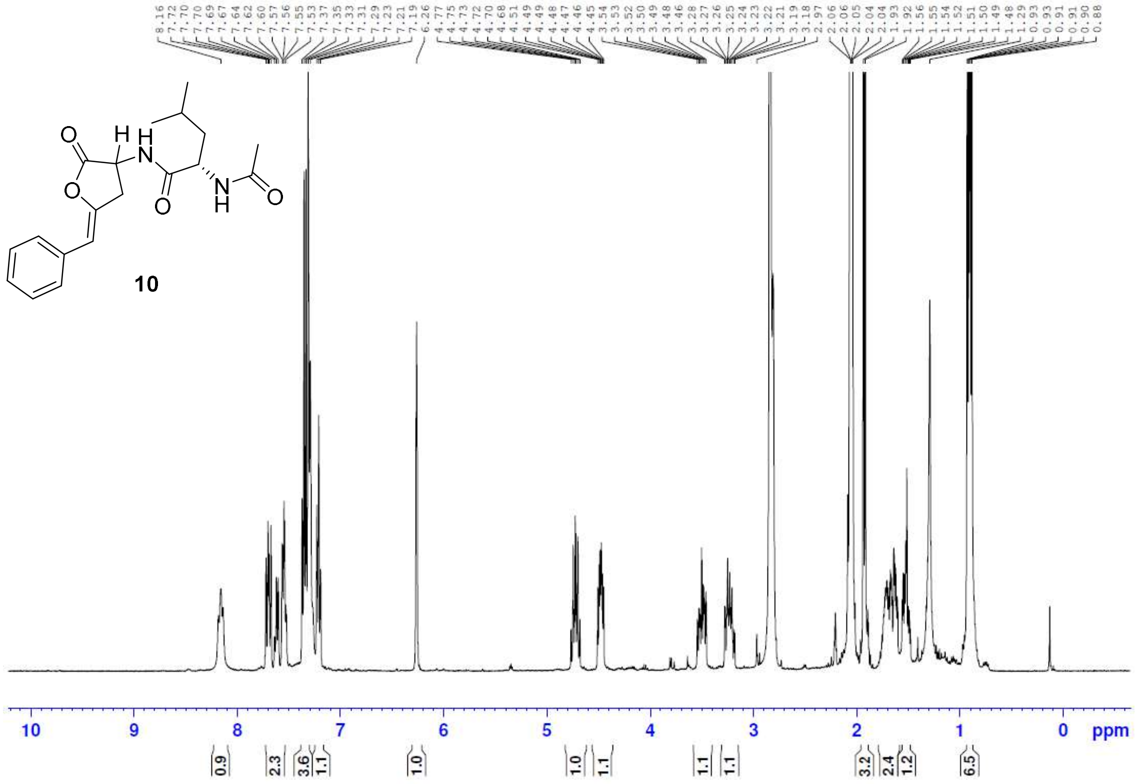
10
10 9 8 7 6 5 4 3 2 1 0 ppm
0.9 2.3 3.6 1.1 1.0 1.0 1.1 1.1 1.1 3.2 2.4 1.2 6.5

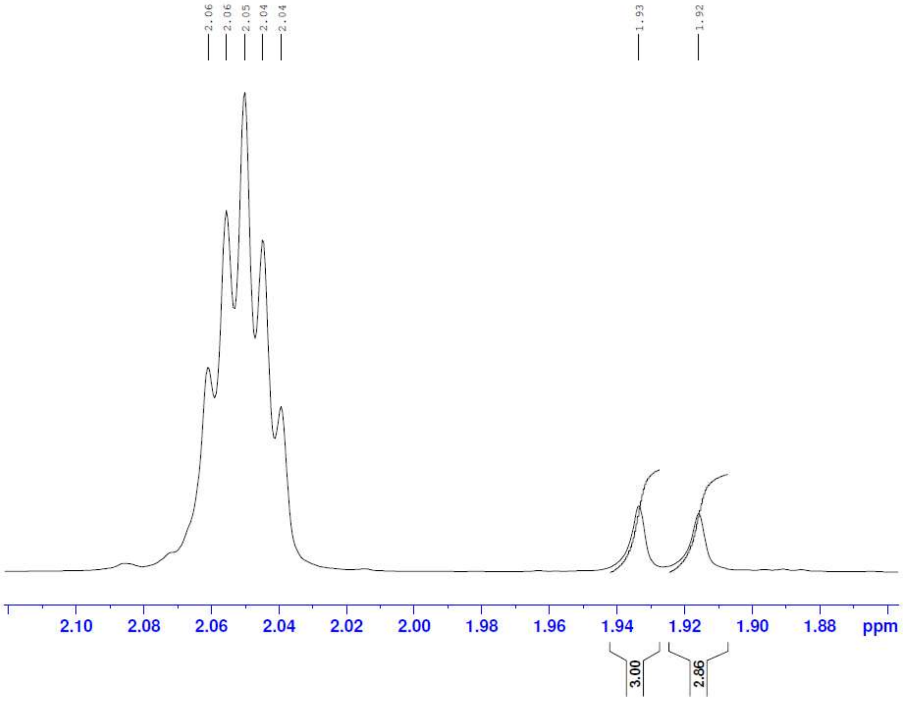
2.06 2.06 2.05 2.04 2.04
1.93
1.92
2.10 2.08 2.06 2.04 2.02 2.00 1.98 1.96 1.94 1.92 1.90 1.88 ppm
3.00
2.86

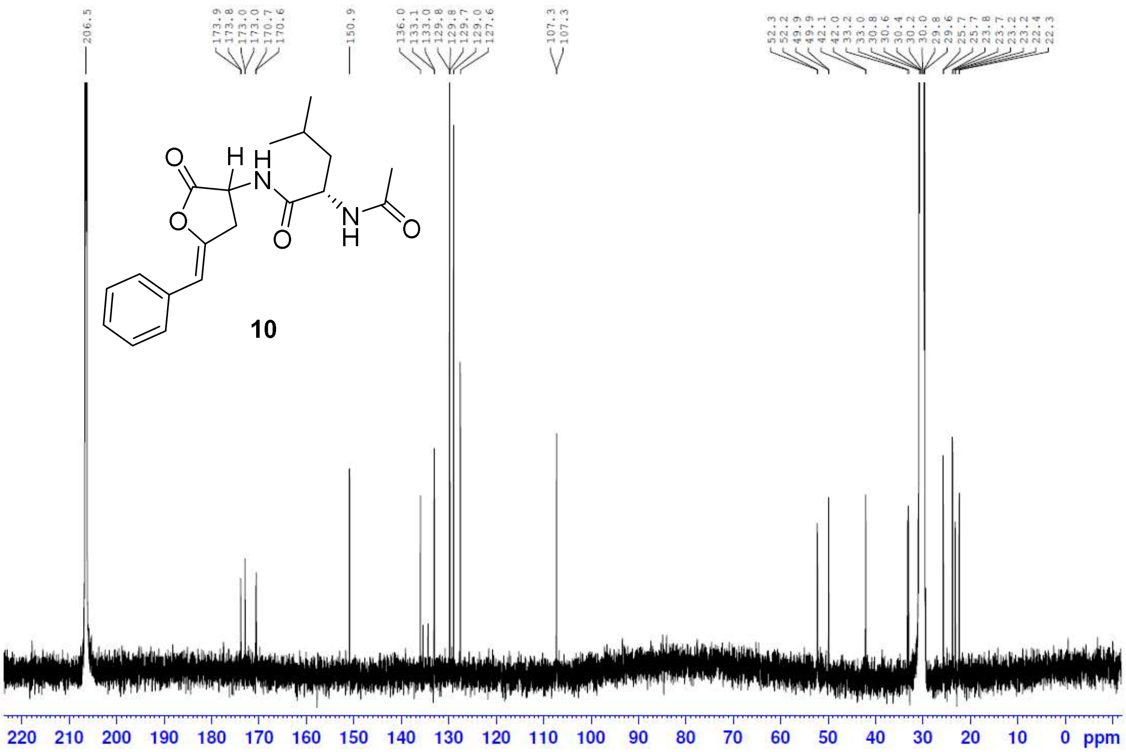
206.5
173.9
173.8
173.0
173.0
170.7
170.6
150.9
136.0
133.1
133.0
129.8
129.8
129.7
129.0
127.6
107.3
107.3
52.3
52.2
49.9
49.9
42.1
42.0
33.2
33.0
30.8
30.6
30.4
30.2
30.0
29.8
29.6
25.7
25.7
23.8
23.7
23.2
23.2
22.4
22.3
10
220 210 200 190 180 170 160 150 140 130 120 110 100 90 80 70 60 50 40 30 20 10 0 ppm

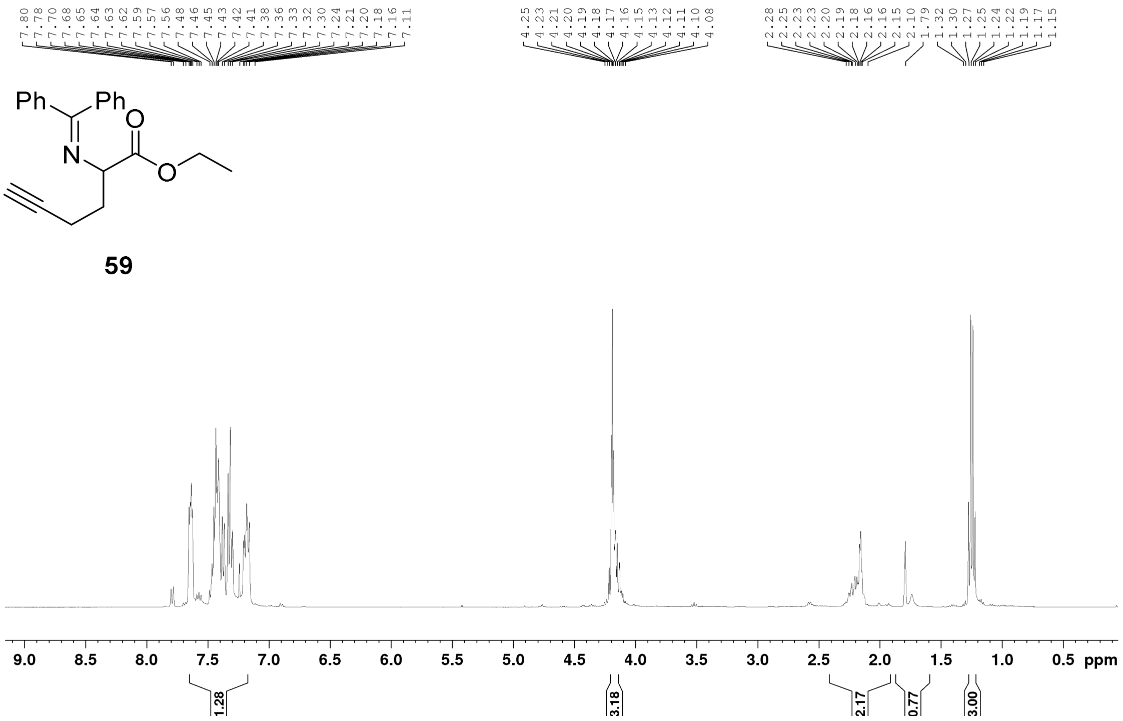

Ph
Ph
O
N
O
59
9.0
8.5
8.0
7.5
7.0
6.5
6.0
5.5
5.0
4.5
4.0
3.5
3.0
2.5
2.0
1.5
1.0
0.5 ppm
1.28
3.18
2.17
0.77
3.00

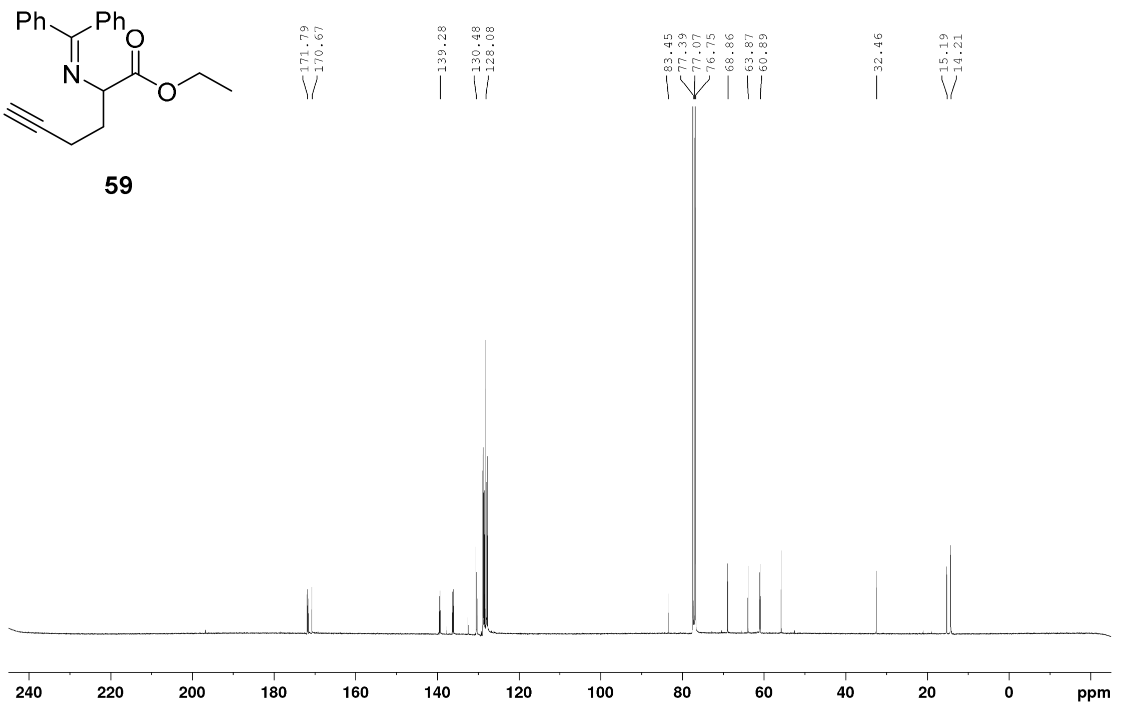

Ph
Ph
O
N
O
59
171.79
170.67
139.28
130.48
128.08
83.45
77.39
77.07
76.75
68.86
63.87
60.89
32.46
15.19
14.21
240
220
200
180
160
140
120
100
80
60
40
20
0
ppm

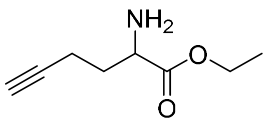
NH2
O
O

15e

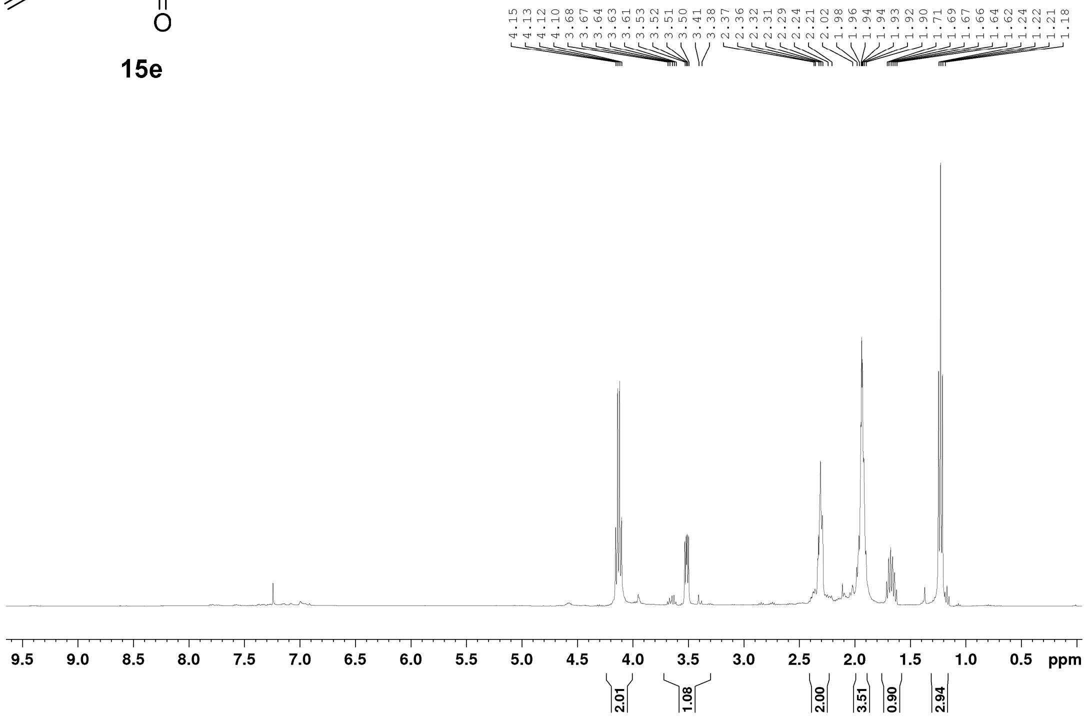
4.15
4.13
4.12
4.10
3.68
3.67
3.64
3.63
3.61
3.53
3.52
3.51
3.50
3.41
3.38
2.37
2.36
2.32
2.31
2.29
2.24
2.21
2.02
1.98
1.96
1.94
1.94
1.93
1.92
1.90
1.71
1.69
1.67
1.66
1.64
1.62
1.24
1.22
1.21
1.18
2.01
1.08
2.00
3.51
0.90
2.94
9.5 9.0 8.5 8.0 7.5 7.0 6.5 6.0 5.5 5.0 4.5 4.0 3.5 3.0 2.5 2.0 1.5 1.0 0.5 ppm

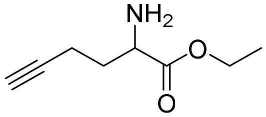
NH2
O
O

15e

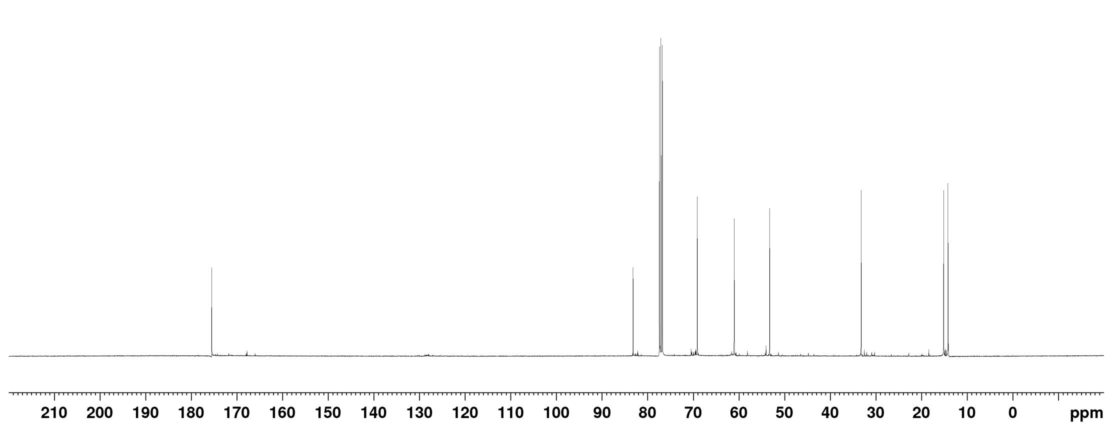
175.51
83.19
77.40
77.08
76.77
69.13
61.04
53.26
33.22
15.12
14.18
210 200 190 180 170 160 150 140 130 120 110 100 90 80 70 60 50 40 30 20 10 0 ppm

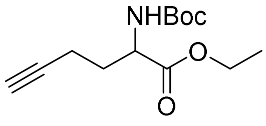
NHBoc
O
O

17h

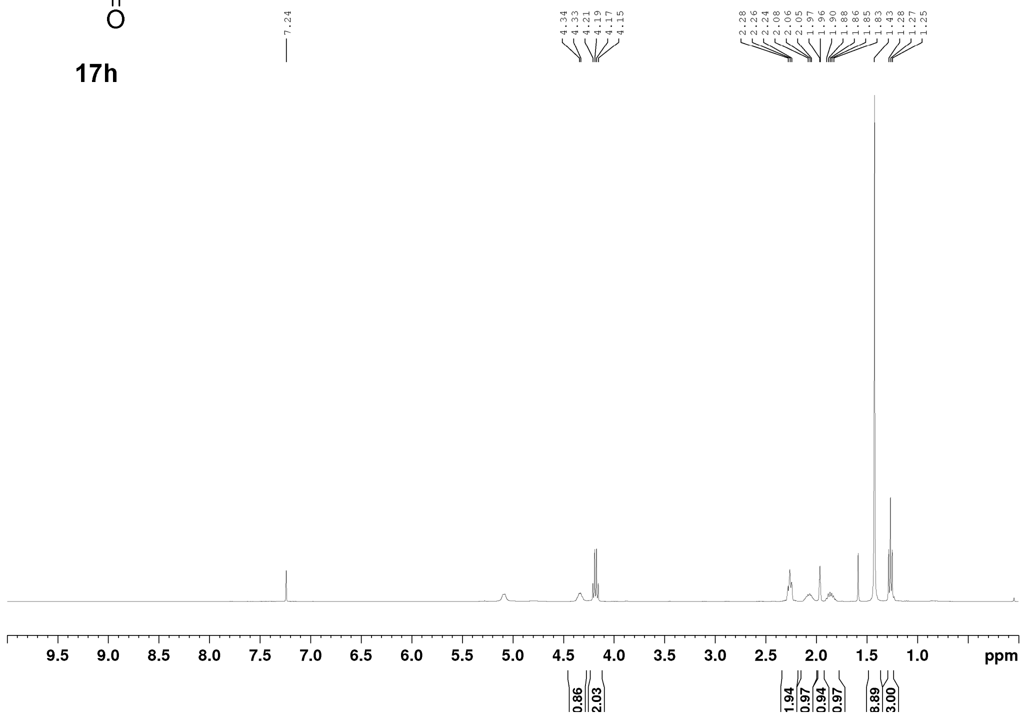
7.24
4.34
4.33
4.21
4.19
4.17
4.15
2.28
2.26
2.24
2.08
2.06
2.05
1.97
1.96
1.90
1.88
1.86
1.85
1.83
1.43
1.28
1.27
1.25
9.5 9.0 8.5 8.0 7.5 7.0 6.5 6.0 5.5 5.0 4.5 4.0 3.5 3.0 2.5 2.0 1.5 1.0 ppm
0.86
2.03
1.94
0.97
0.94
0.97
8.89
3.00

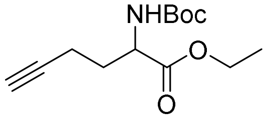
NHBoc
O
O

17h

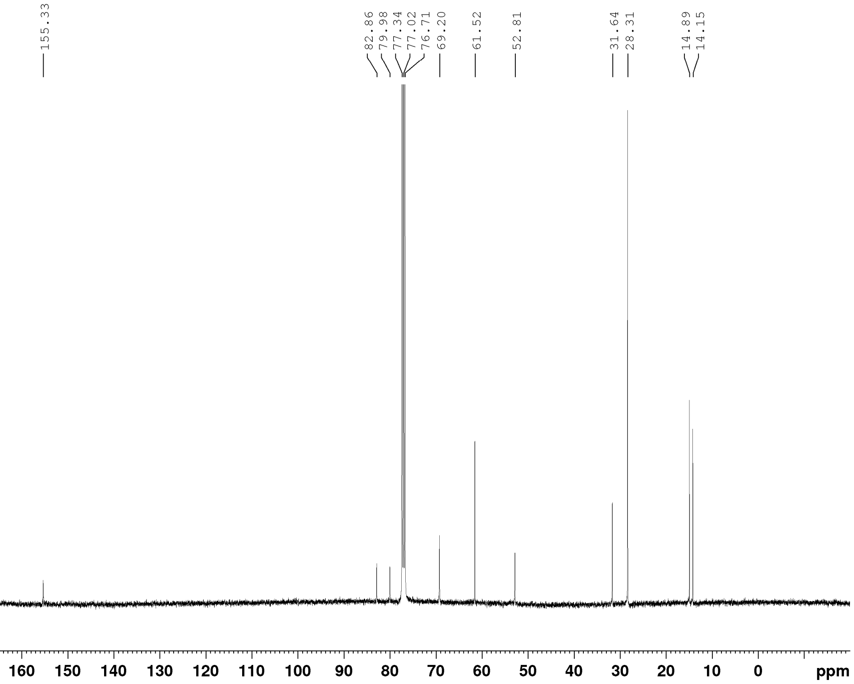
172.21
155.33
82.86
79.98
77.34
77.02
76.71
69.20
61.52
52.81
31.64
28.31
14.89
14.15
210 200 190 180 170 160 150 140 130 120 110 100 90 80 70 60 50 40 30 20 10 0 ppm

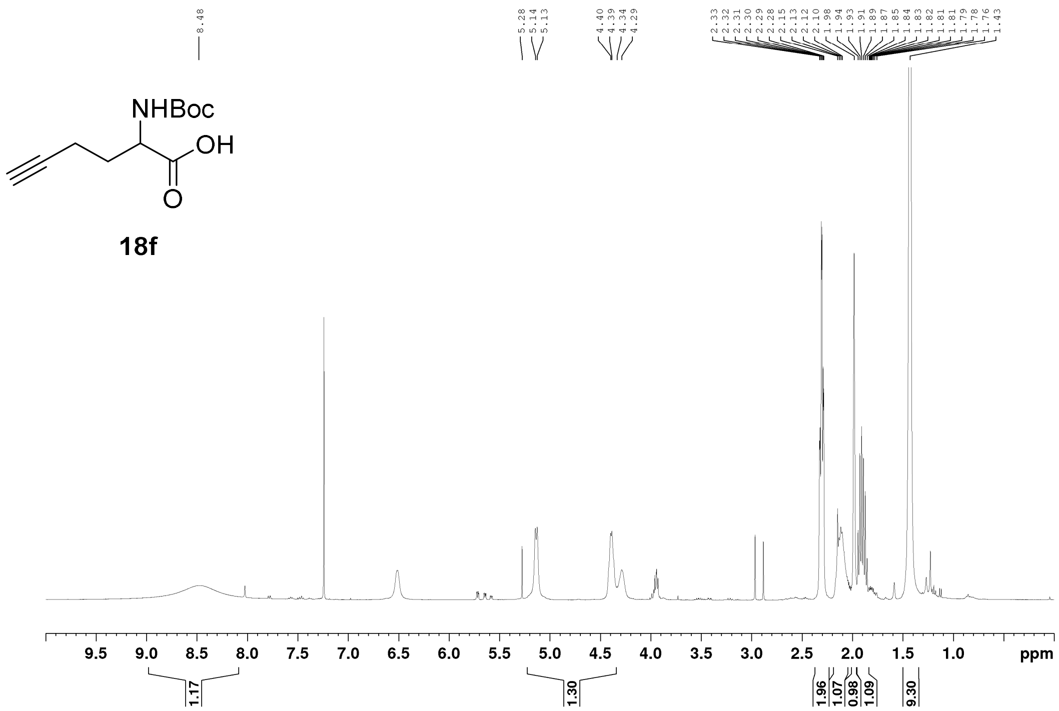
NHBoc
OH
O
18f
8.48
5.28 5.14 5.13
4.40 4.39 4.34 4.29
2.33 2.32 2.31 2.30 2.29 2.28 2.15 2.13 2.12 2.10 1.98 1.94 1.93 1.91 1.89 1.87 1.85 1.84 1.83 1.82 1.81 1.79 1.78 1.76 1.43
9.5 9.0 8.5 8.0 7.5 7.0 6.5 6.0 5.5 5.0 4.5 4.0 3.5 3.0 2.5 2.0 1.5 1.0 ppm
1.17
1.30
1.96 1.07 0.98 1.09
9.30

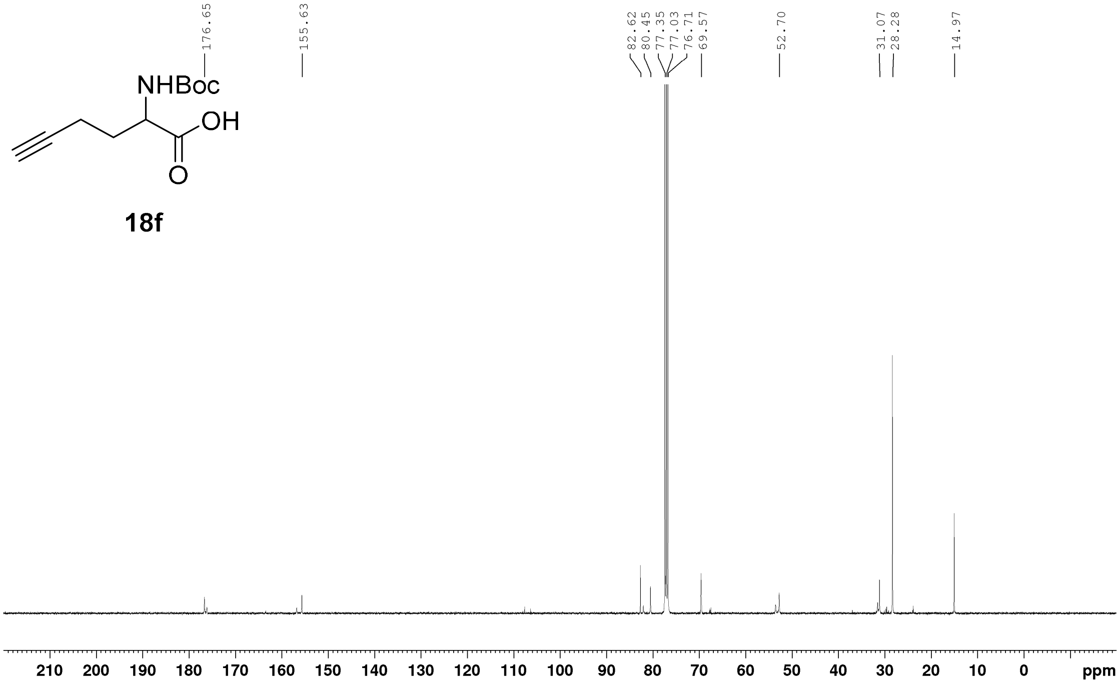
NHBoc
OH
O
18f
176.65
155.63
82.62 80.45 77.35 77.03 76.71 69.57
52.70
31.07 28.28
14.97
210 200 190 180 170 160 150 140 130 120 110 100 90 80 70 60 50 40 30 20 10 0 ppm

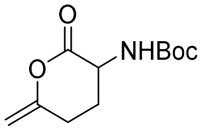
O
NHBoc
O

11

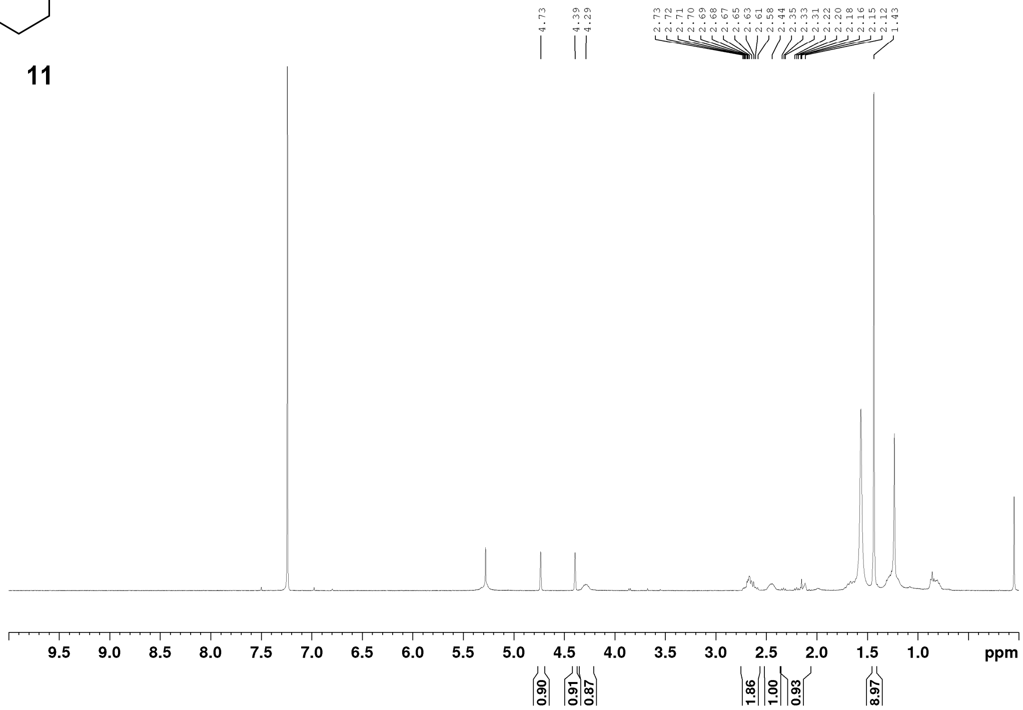
4.73
4.39
4.29
2.73 2.72 2.71 2.70 2.69 2.68 2.67 2.65 2.63 2.61 2.58 2.44 2.35 2.33 2.31 2.22 2.20 2.18 2.16 2.15 2.12 1.43
9.5 9.0 8.5 8.0 7.5 7.0 6.5 6.0 5.5 5.0 4.5 4.0 3.5 3.0 2.5 2.0 1.5 1.0 ppm
0.90
0.91
0.87
1.86
1.00
0.93
8.97

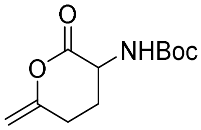
O
NHBoc
O

11

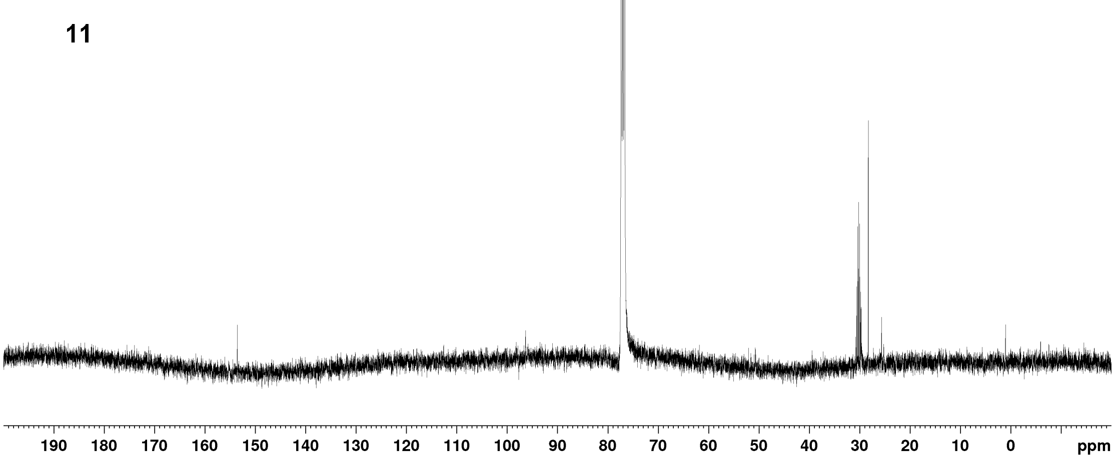
175.66
154.05
153.05
96.15
80.75
77.34
77.02
76.70
61.87
48.20
30.19
28.28
190 180 170 160 150 140 130 120 110 100 90 80 70 60 50 40 30 20 10 0 ppm

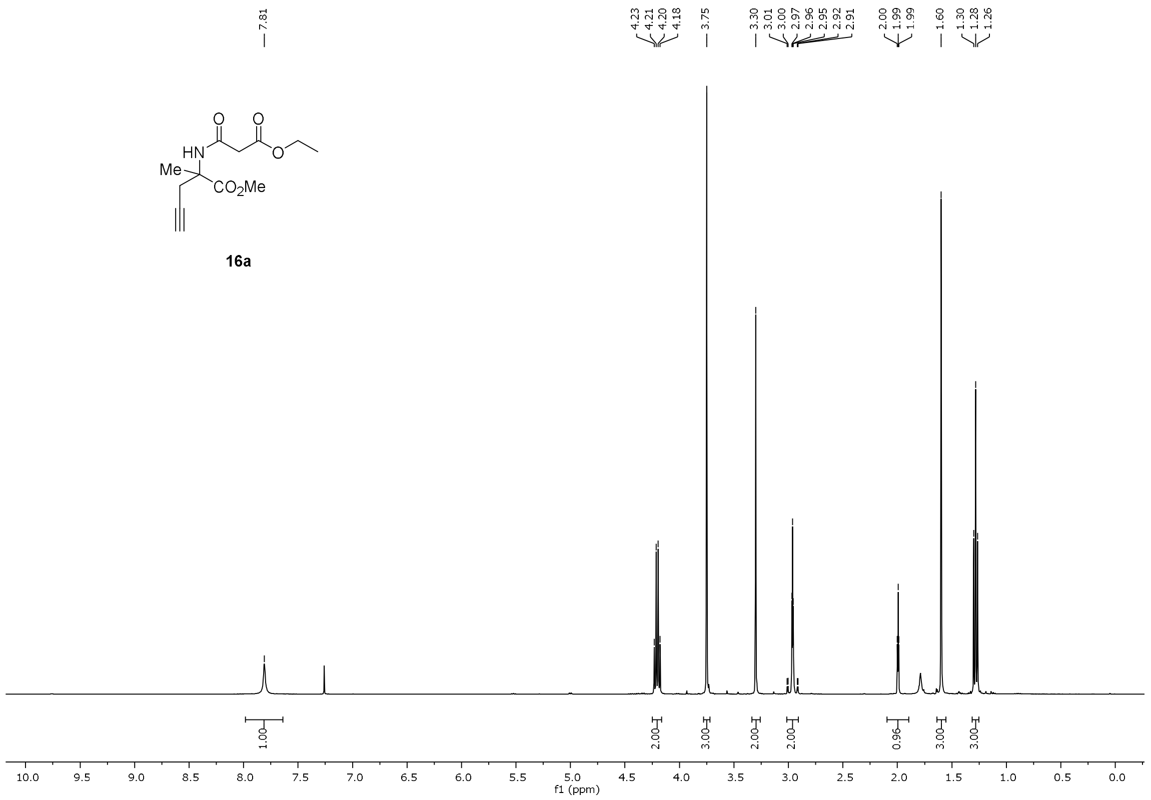
7.81
4.23
4.21
4.20
4.18
3.75
3.30
3.01
3.00
2.97
2.96
2.95
2.92
2.91
2.00
1.99
1.99
1.60
1.30
1.28
1.26
HN
Me
CO_2Me
16a
1.00
2.00
3.00
2.00
2.00
0.96
3.00
3.00
f1 (ppm)

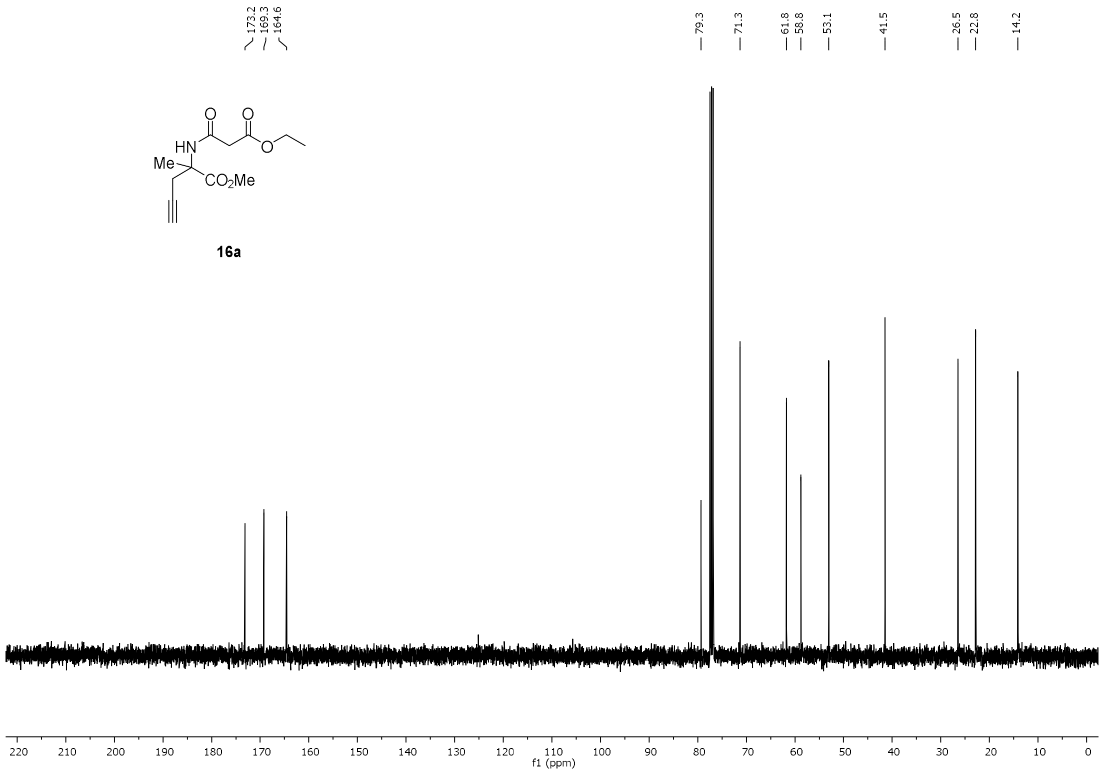
173.2
169.3
164.6
79.3
71.3
61.8
58.8
53.1
41.5
26.5
22.8
14.2
HN
Me
CO_2Me
16a
f1 (ppm)

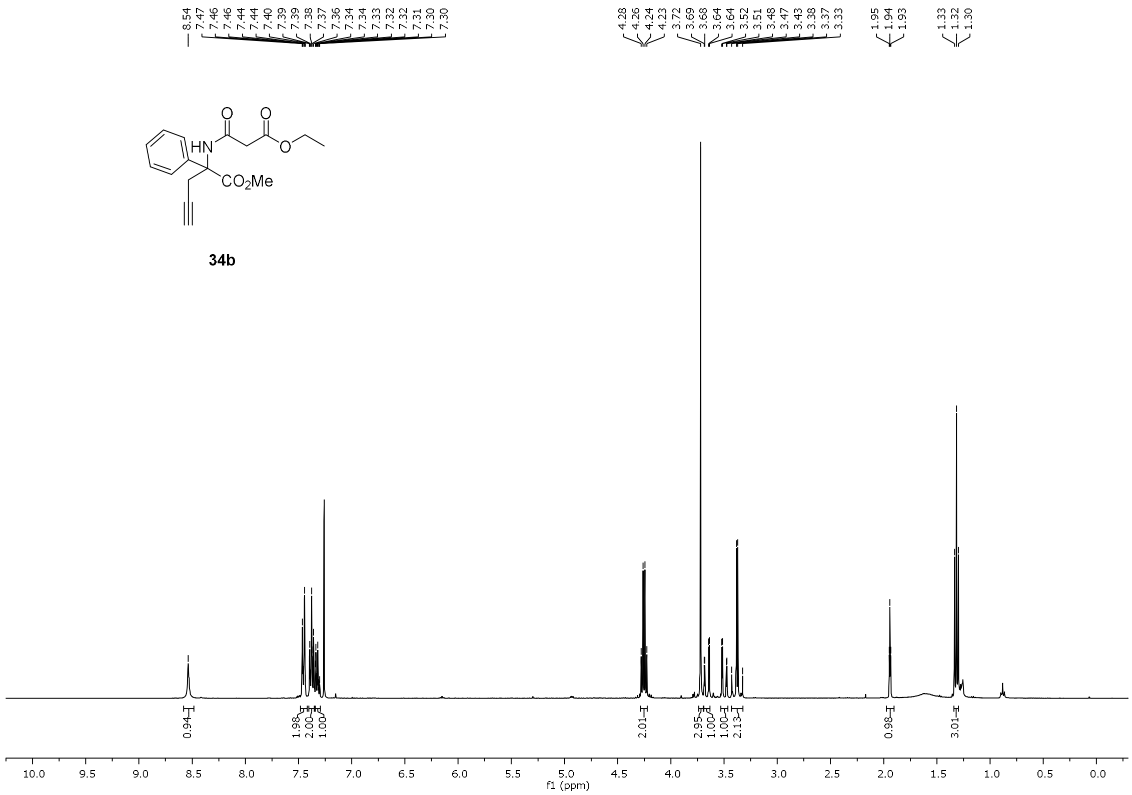
HN
CO2Me
34b
f1 (ppm)

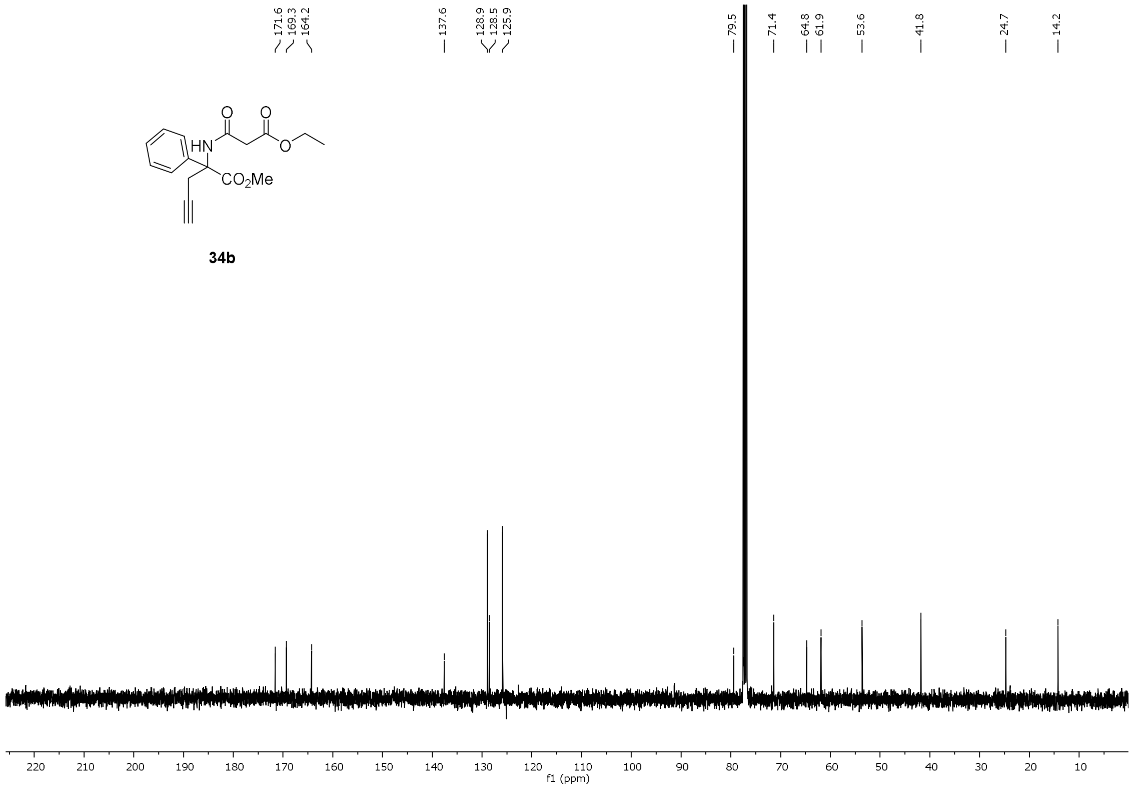
HN
CO2Me
34b
f1 (ppm)

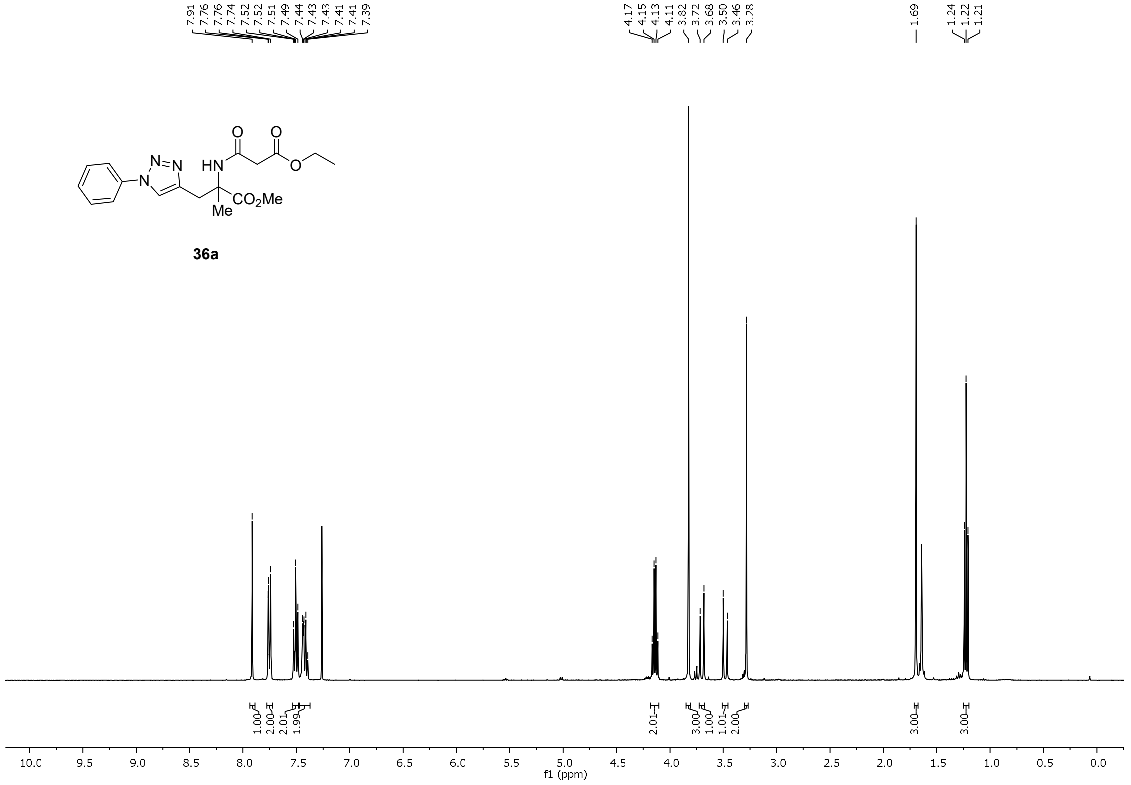

7.91 7.76 7.76 7.74 7.52 7.52 7.51 7.49 7.44 7.43 7.43 7.41 7.41 7.39
4.17 4.15 4.13 4.11 3.82 3.72 3.68 3.50 3.46 3.28
1.69
1.24 1.22 1.21
36a
1.00 2.00 2.01 1.99
2.01 3.00 1.00 1.01 2.00
3.00
3.00
f1 (ppm)

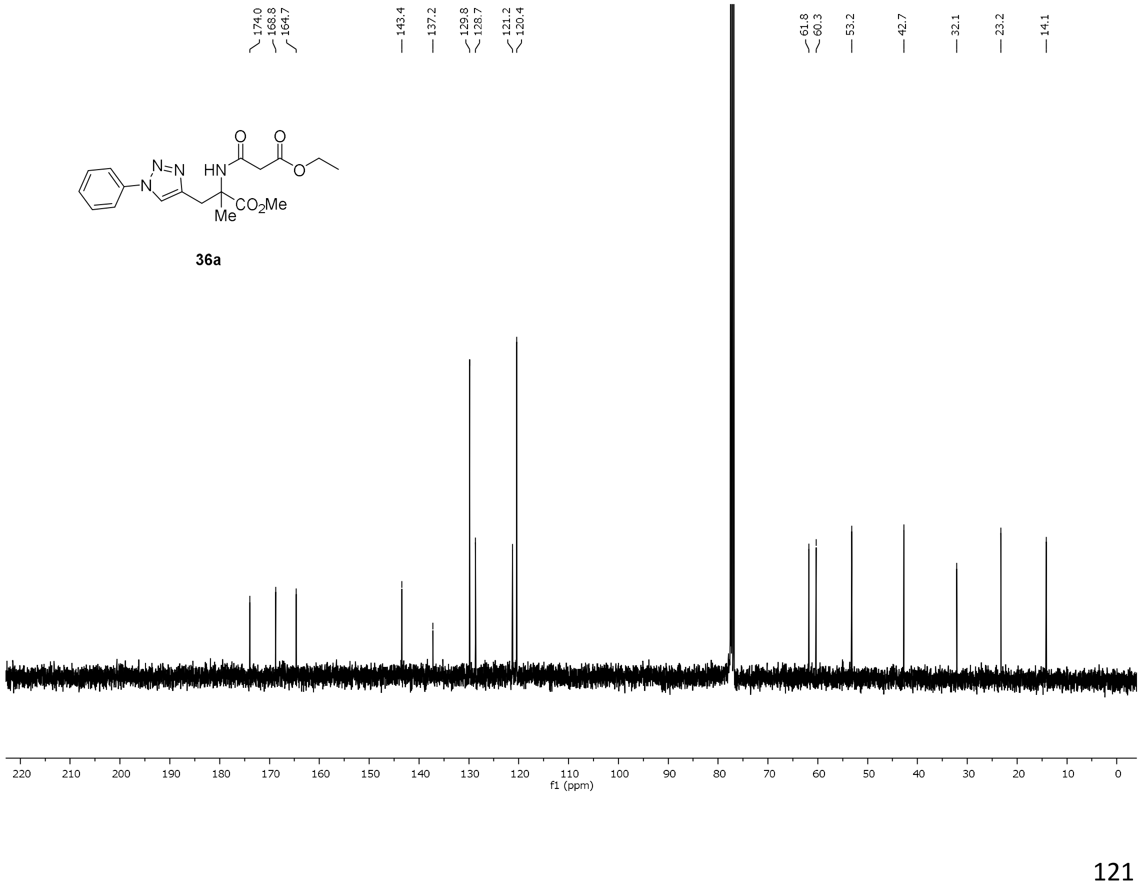

174.0 168.8 164.7
143.4
137.2
129.8 128.7
121.2 120.4
61.8 60.3
53.2
42.7
32.1
23.2
14.1
36a
f1 (ppm)

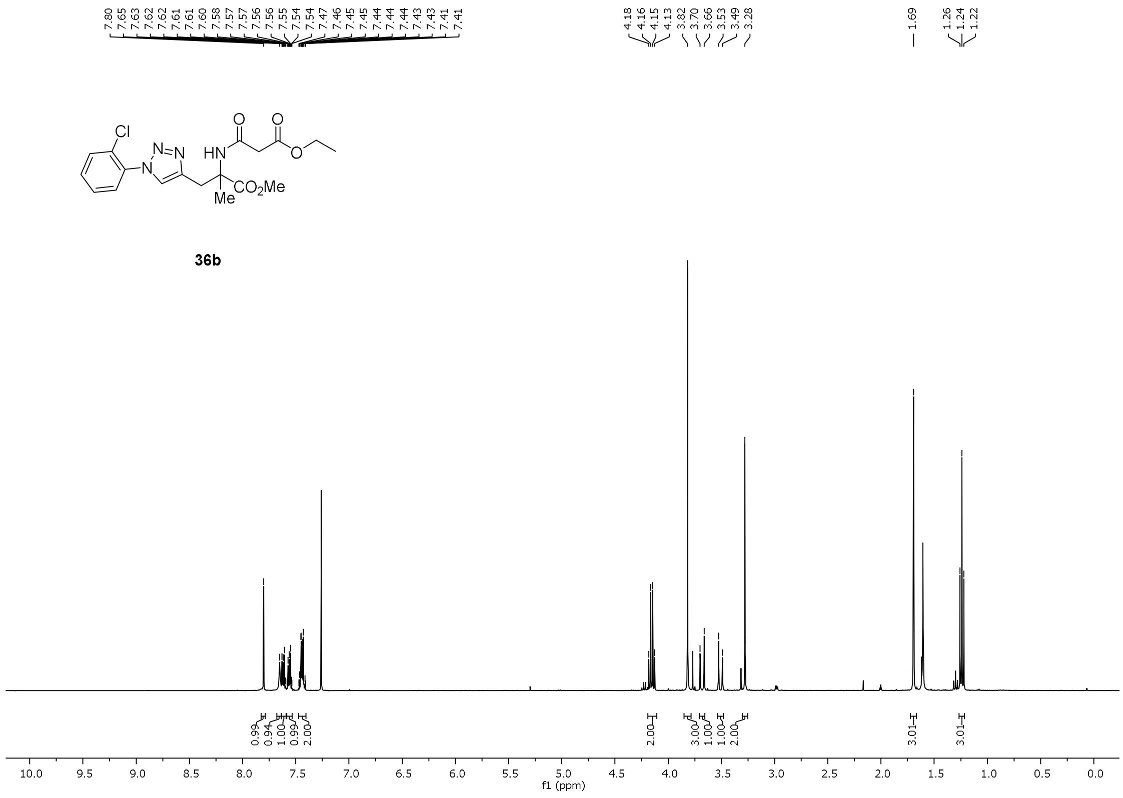
7.80 7.65 7.63 7.62 7.62 7.61 7.61 7.60 7.58 7.57 7.57 7.56 7.56 7.55 7.54 7.54 7.47 7.46 7.45 7.45 7.44 7.44 7.43 7.43 7.41 7.41
4.18 4.16 4.15 4.13 3.82 3.70 3.66 3.53 3.49 3.28
1.69 1.26 1.24 1.22
Cl
N
N=N
HN
O
O
O
CO2Me
Me
36b
0.99 0.94 1.00 0.99 2.00
2.00
3.00 1.00 1.00 2.00
3.01
3.01
10.0 9.5 9.0 8.5 8.0 7.5 7.0 6.5 6.0 5.5 5.0 4.5 4.0 3.5 3.0 2.5 2.0 1.5 1.0 0.5 0.0
f1 (ppm)

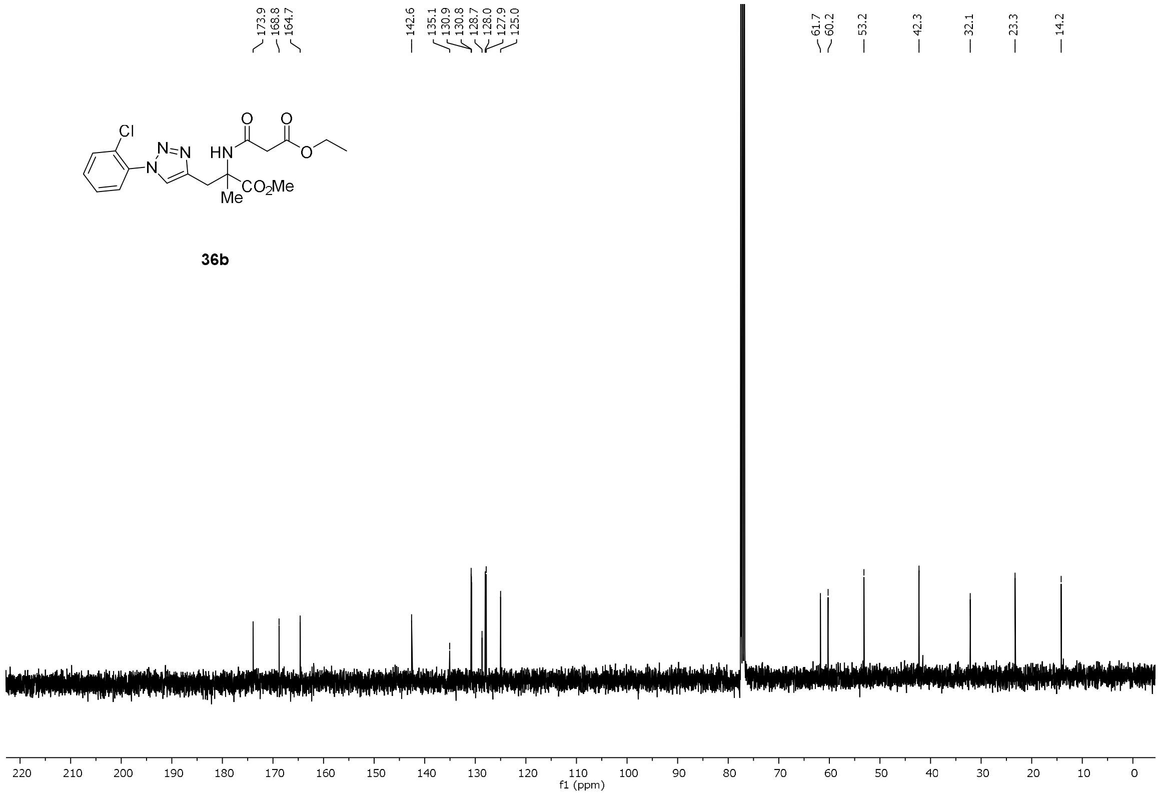
173.9 168.8 164.7
142.6 135.1 130.9 130.8 128.7 128.0 127.9 125.0
61.7 60.2 53.2 42.3 32.1 23.3 14.2
Cl
N
N=N
HN
O
O
O
CO2Me
Me
36b
220 210 200 190 180 170 160 150 140 130 120 110 100 90 80 70 60 50 40 30 20 10 0
f1 (ppm)

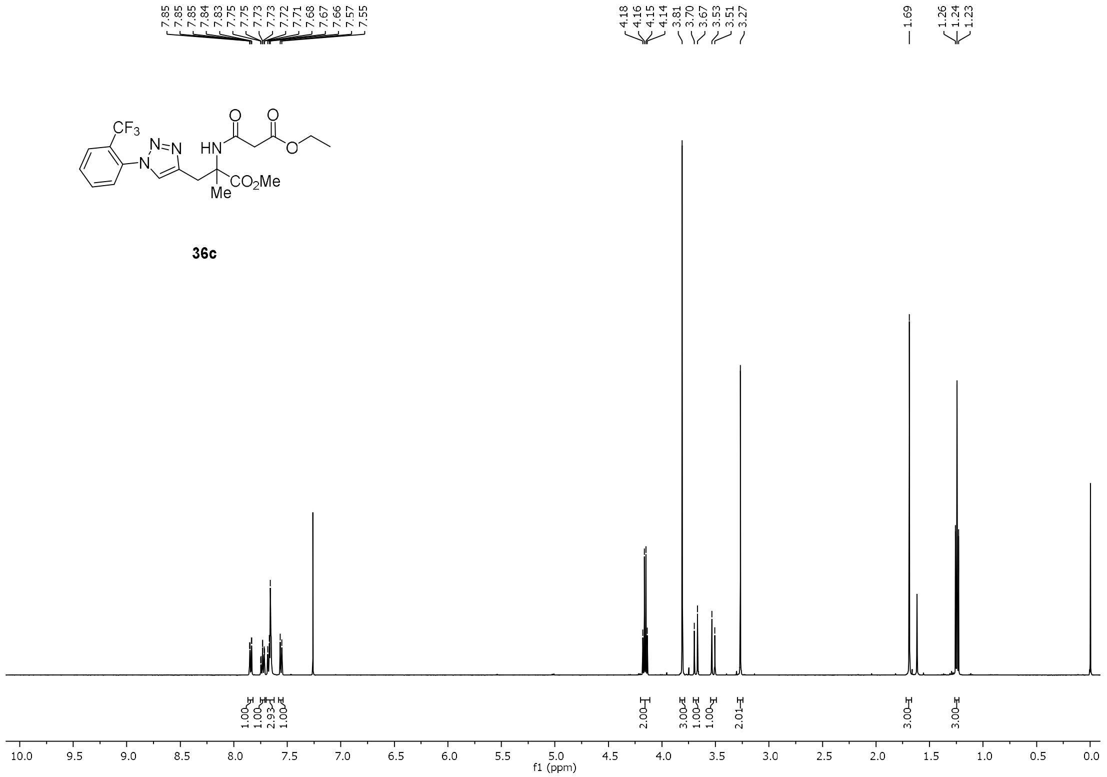
7.85 7.85 7.85 7.84 7.83 7.75 7.75 7.73 7.73 7.72 7.71 7.68 7.67 7.66 7.57 7.55
4.18 4.16 4.15 4.14 3.81 3.70 3.67 3.53 3.51 3.27
1.69
1.26 1.24 1.23
CF3
N=N
HN
CO2Me
Me
O
36c
1.00 1.00 2.93 1.00
2.00
3.00 1.00 1.00
2.01
3.00
3.00
f1 (ppm)

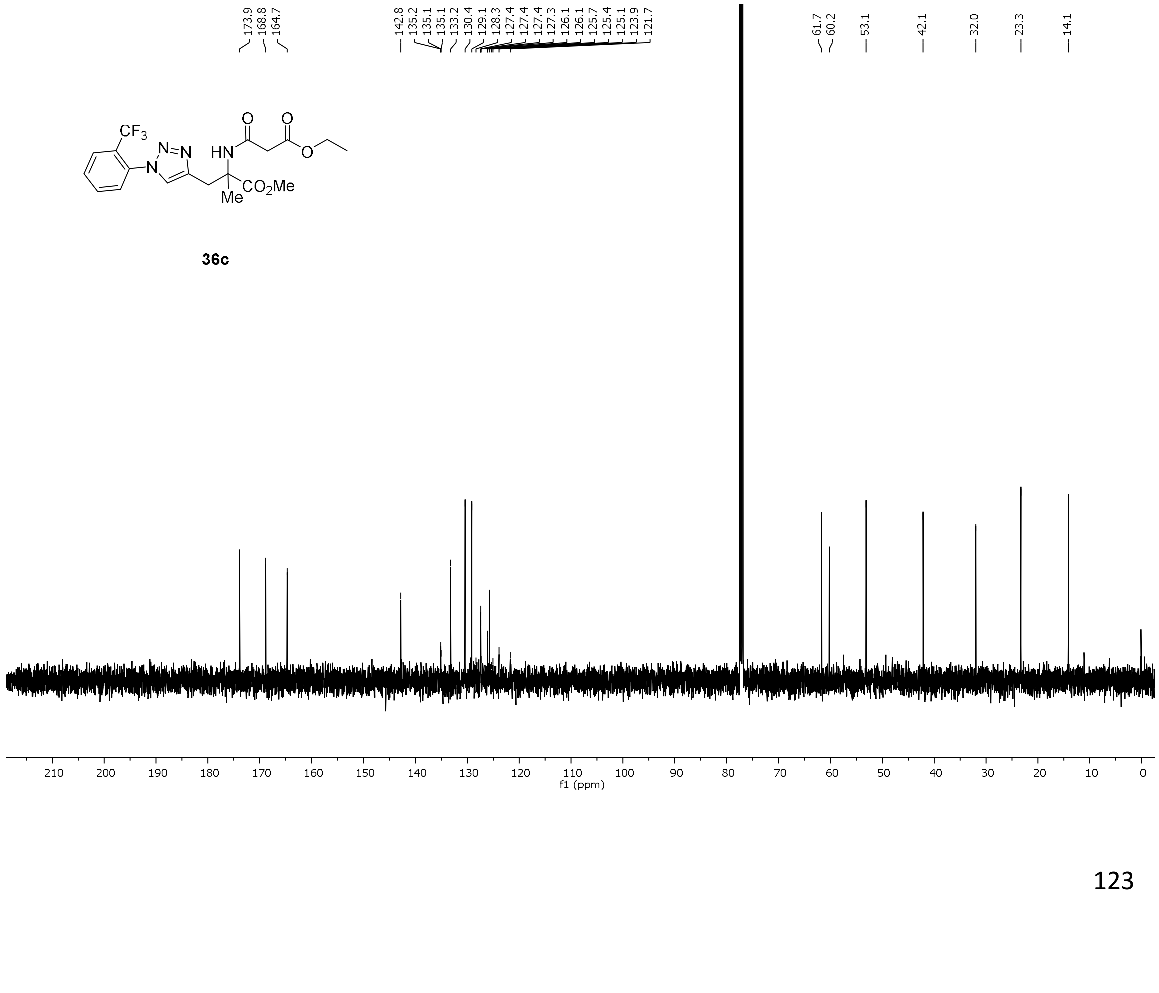
173.9 168.8 164.7
142.8 135.2 135.1 133.2 130.4 129.1 128.3 127.4 127.4 127.3 126.1 126.1 125.7 125.4 125.1 123.9 121.7
61.7 60.2
53.1
42.1
32.0
23.3
14.1
CF3
N=N
HN
CO2Me
Me
O
36c
f1 (ppm)

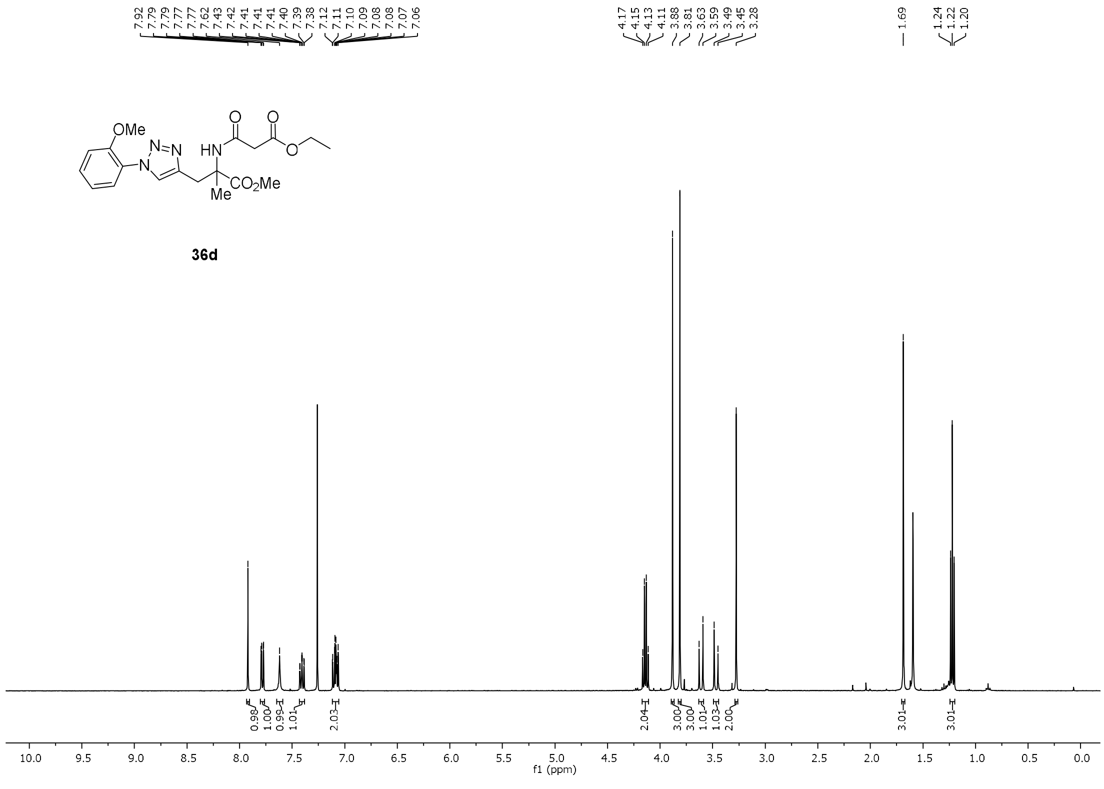
OMe
N=N
HN
CO2Me
Me
f1 (ppm)

36d

,

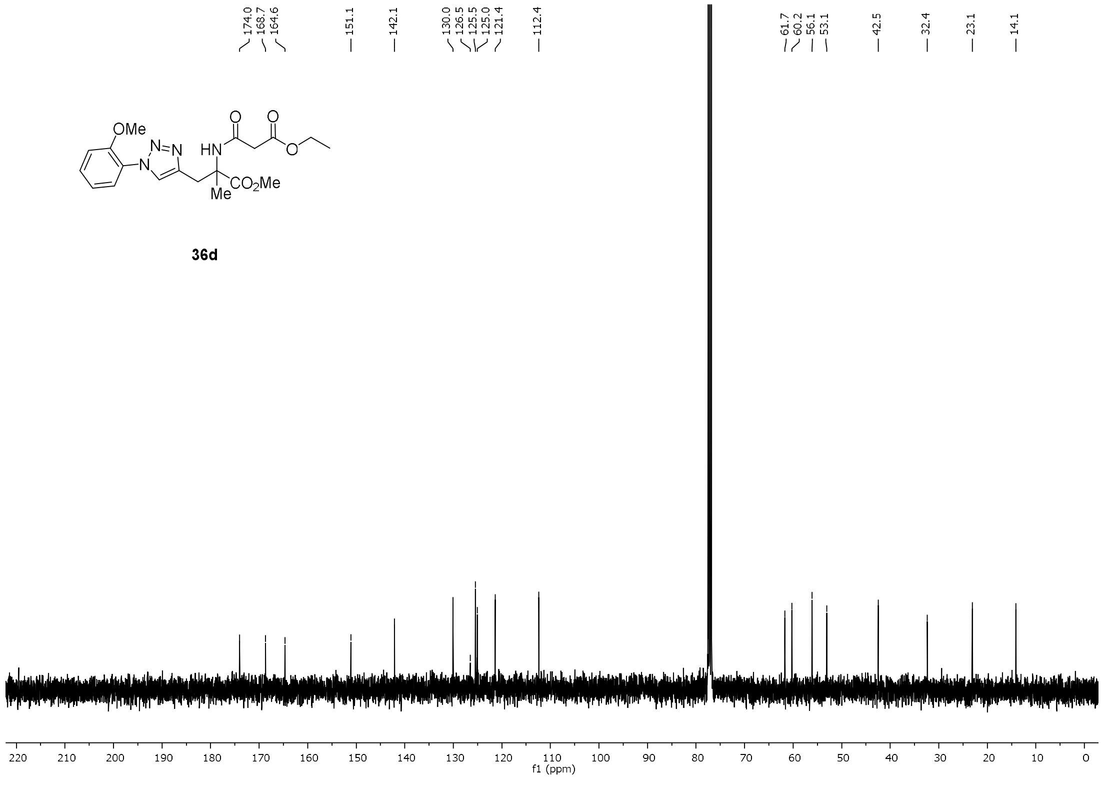
174.0
168.7
164.6
151.1
142.1
130.0
126.5
125.5
125.0
121.4
112.4
61.7
60.2
56.1
53.1
42.5
32.4
23.1
14.1
OMe
N=N
HN
CO2Me
Me
f1 (ppm)

36d

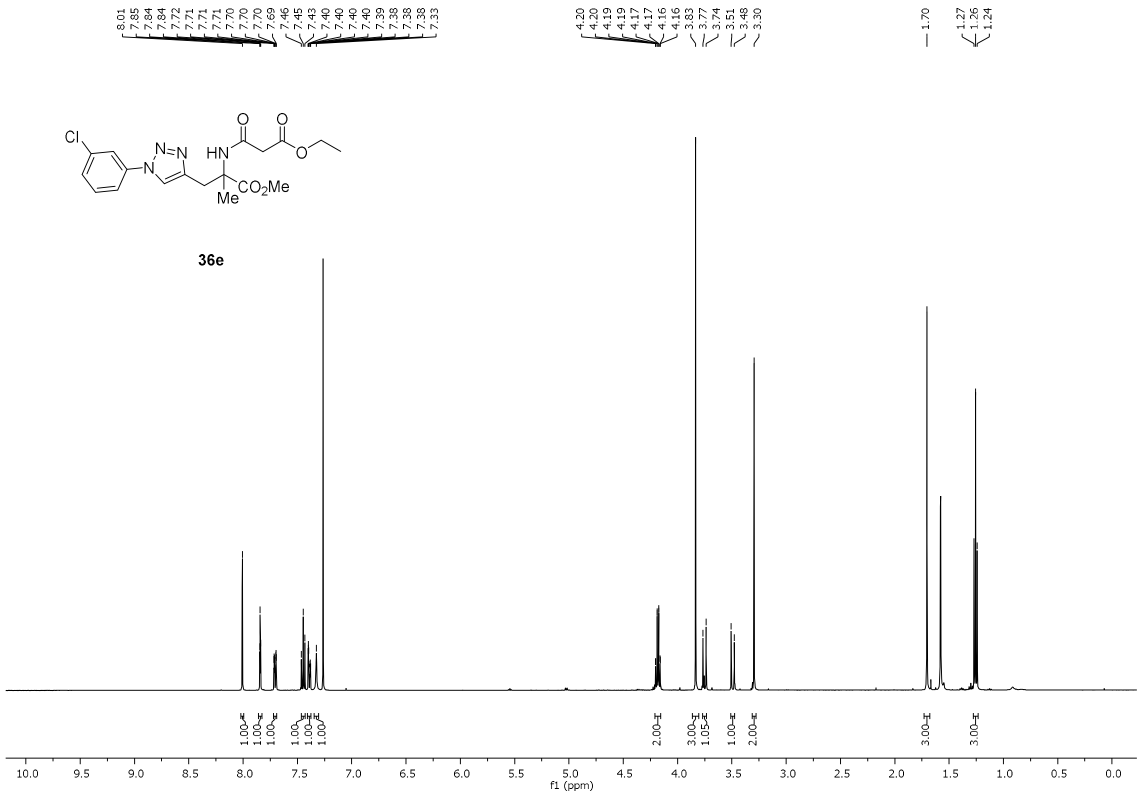

36e

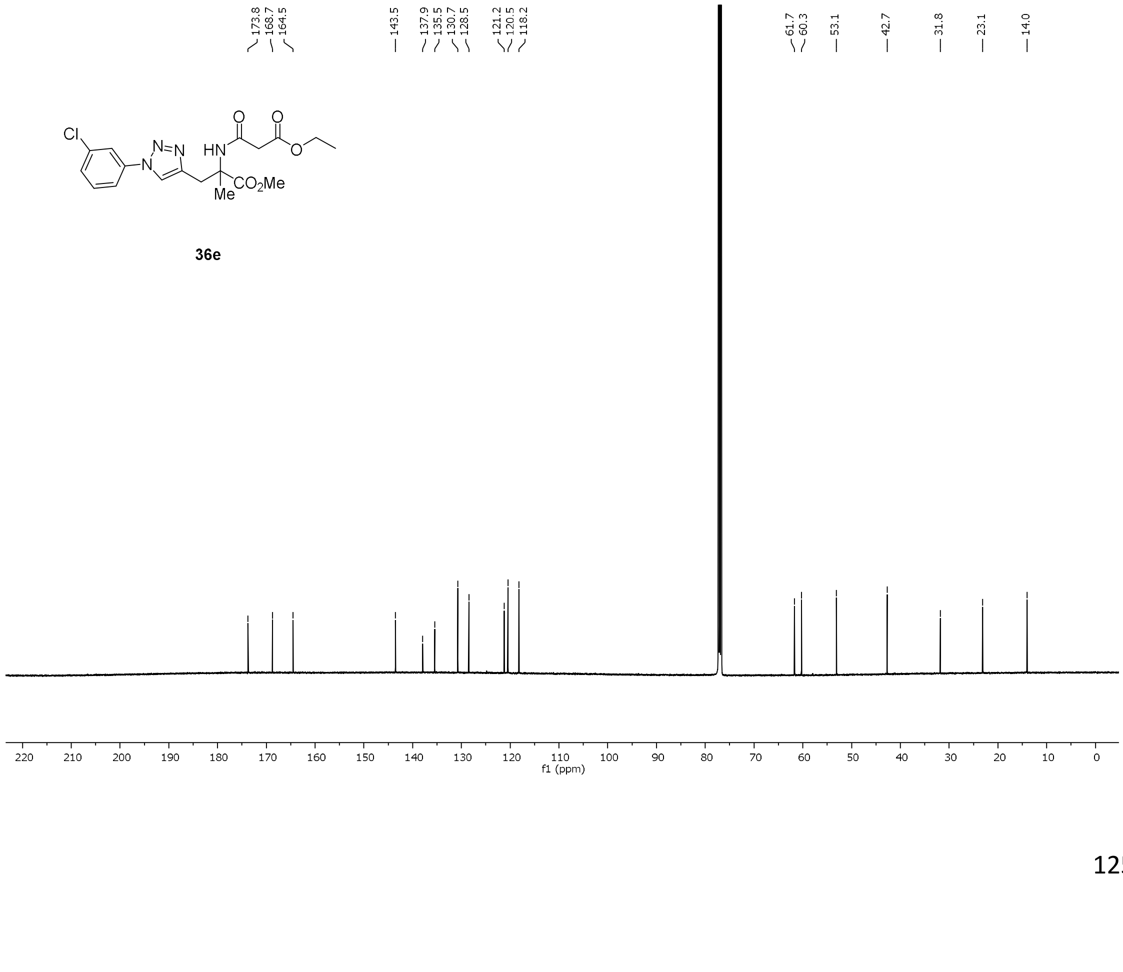

36e

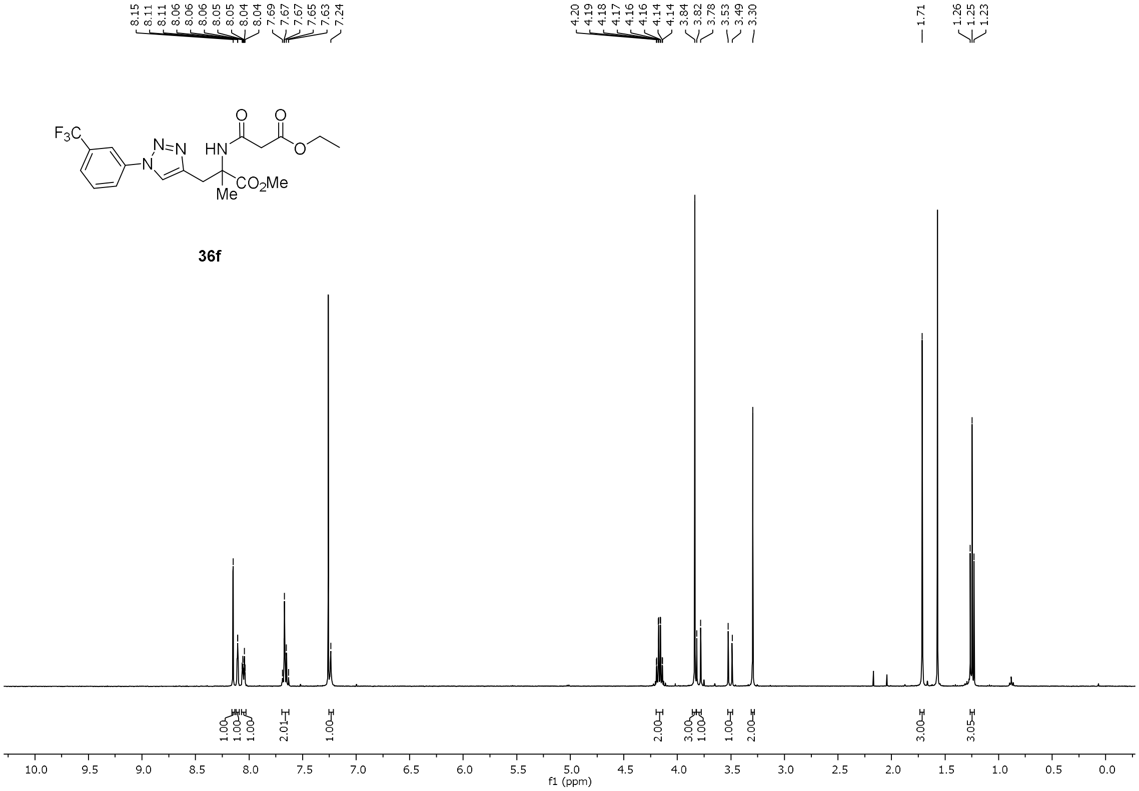
8.15 8.11 8.06 8.06 8.06 8.05 8.05 8.04 8.04 7.69 7.67 7.67 7.65 7.63 7.24
4.20 4.19 4.18 4.17 4.16 4.16 4.14 4.14 3.84 3.82 3.78 3.53 3.49 3.30
1.71
1.26 1.25 1.23
F3C
HN
CO2Me
Me
36f
1.00 1.00 1.00 2.01 1.00 2.00 3.00 1.00 1.00 2.00 3.00 3.05
f1 (ppm)

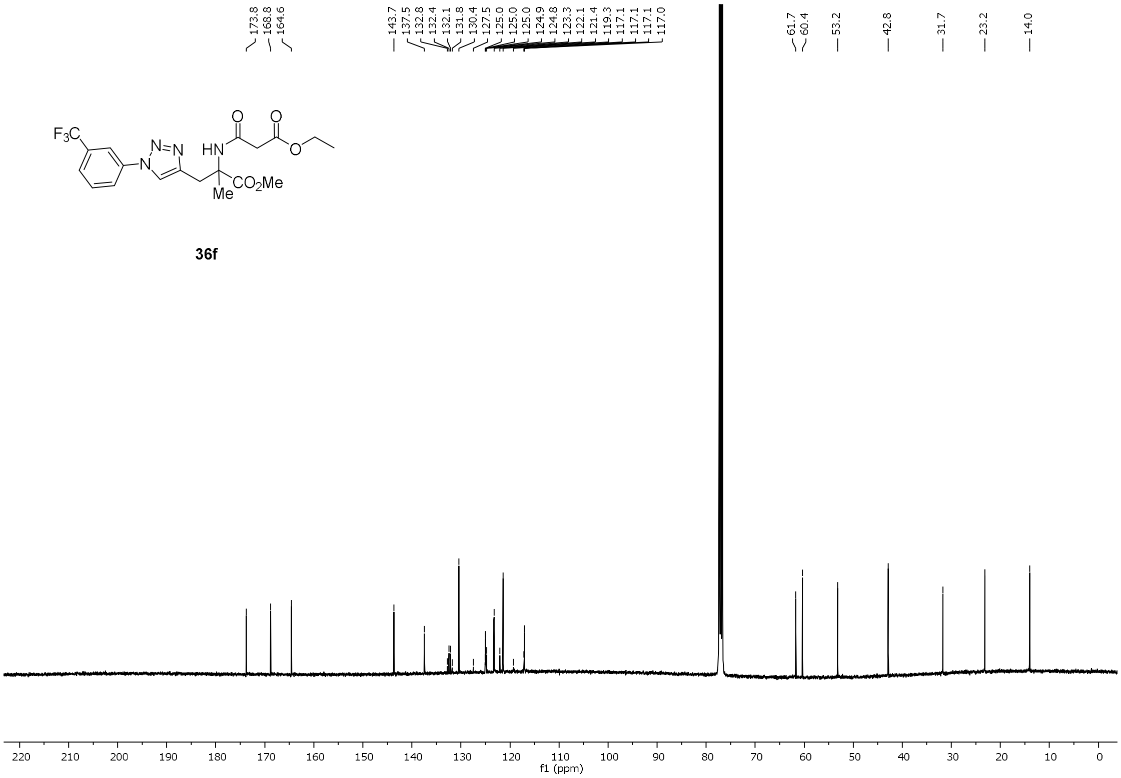
173.8 168.8 164.6
143.7 137.5 132.8 132.4 132.1 131.8 130.4 127.5 125.0 125.0 124.9 124.8 123.3 122.1 121.4 119.3 117.1 117.1 117.1 117.0
61.7 60.4
53.2
42.8
31.7
23.2
14.0
F3C
HN
CO2Me
Me
36f
f1 (ppm)

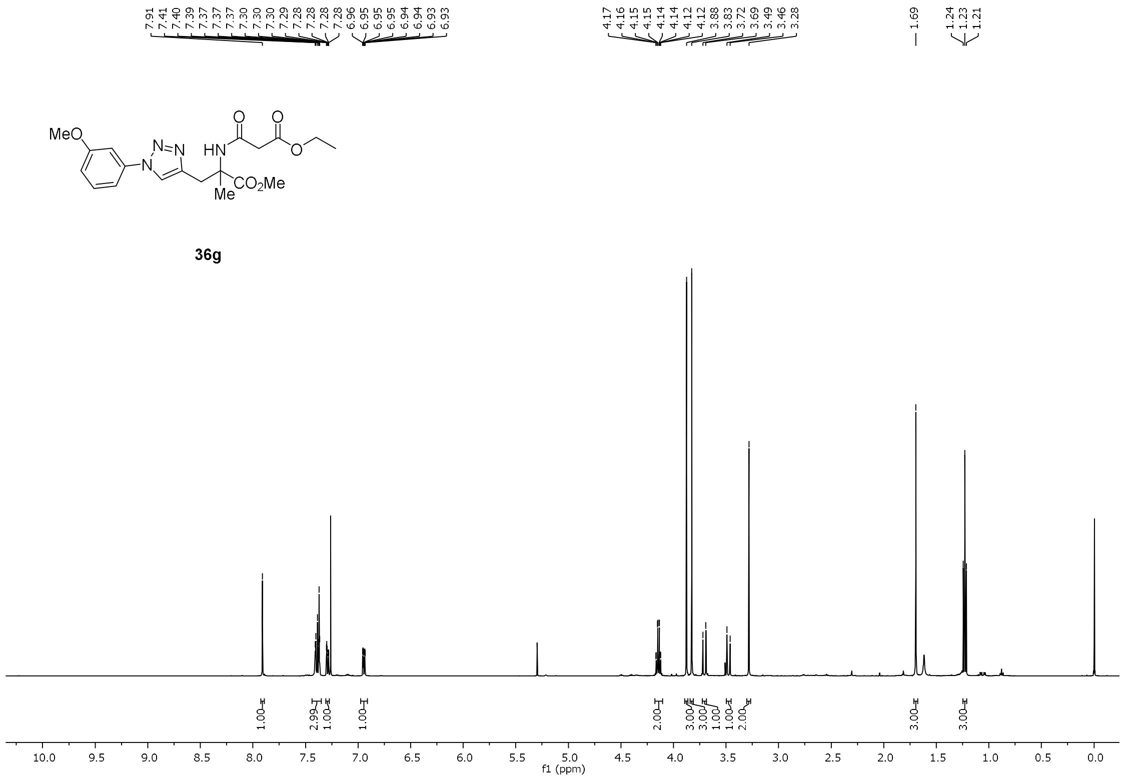
7.91 7.41 7.40 7.39 7.37 7.37 7.37 7.30 7.30 7.30 7.29 7.28 7.28 7.28 7.28 6.96 6.95 6.95 6.95 6.94 6.94 6.93 6.93
4.17 4.16 4.15 4.15 4.14 4.14 4.12 4.12 3.88 3.83 3.72 3.69 3.49 3.46 3.28
1.69
1.24 1.23 1.21
MeO
HN
CO_2Me
Me
1.00 2.99 1.00 1.00 2.00 3.00 3.00 1.00 1.00 2.00 3.00 3.00
f1 (ppm)

36g

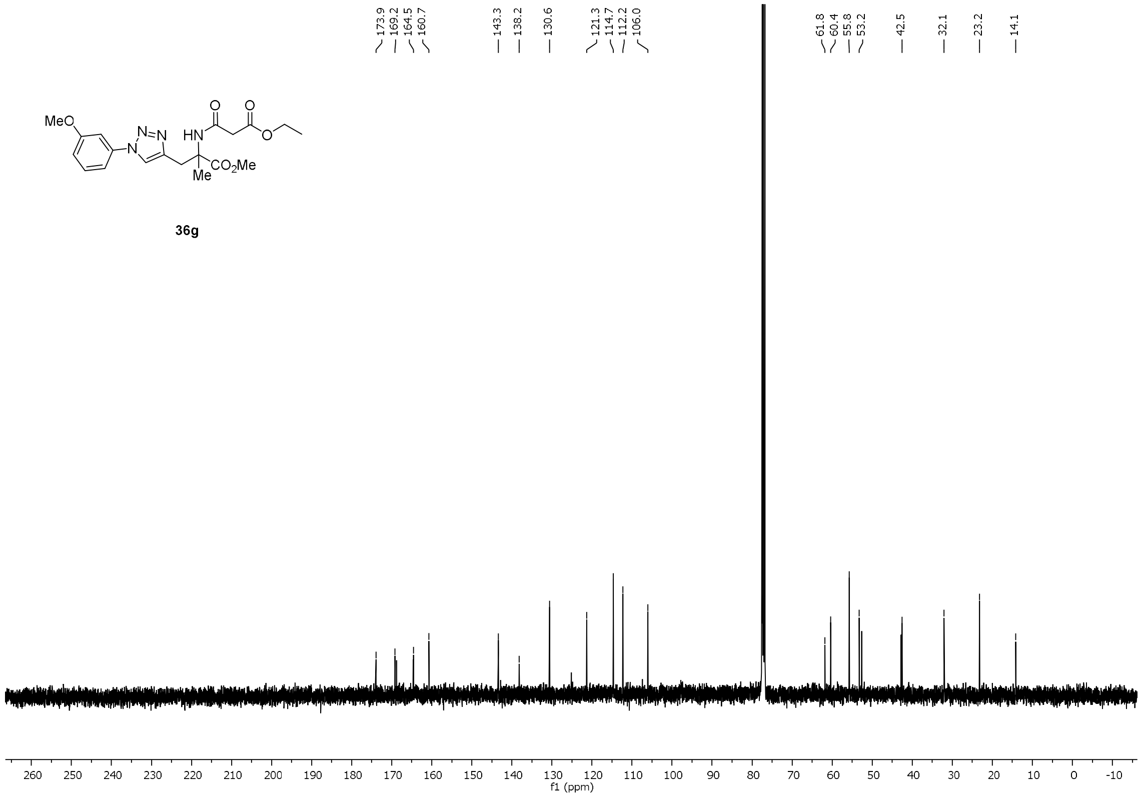
173.9 169.2 164.5 160.7
143.3 138.2 130.6
121.3 114.7 112.2 106.0
61.8 60.4 55.8 53.2
42.5 32.1 23.2 14.1
MeO
HN
CO_2Me
Me
f1 (ppm)

36g

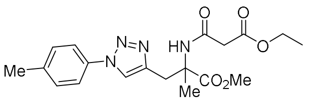

36h

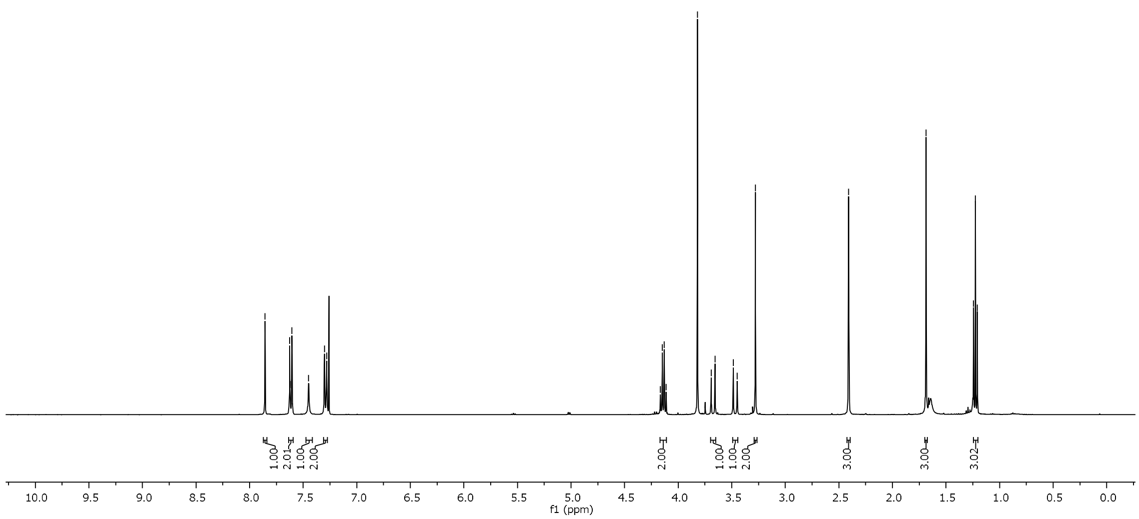

7.86
7.63
7.62
7.61
7.60
7.45
7.30
7.28
4.17
4.15
4.13
4.11
3.82
3.69
3.66
3.49
3.45
3.28
2.41
1.69
1.24
1.23
1.21
1.00
2.01
1.00
2.00
2.00
1.00
1.00
2.00
3.00
3.00
3.02
f1 (ppm)

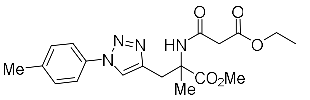

36h

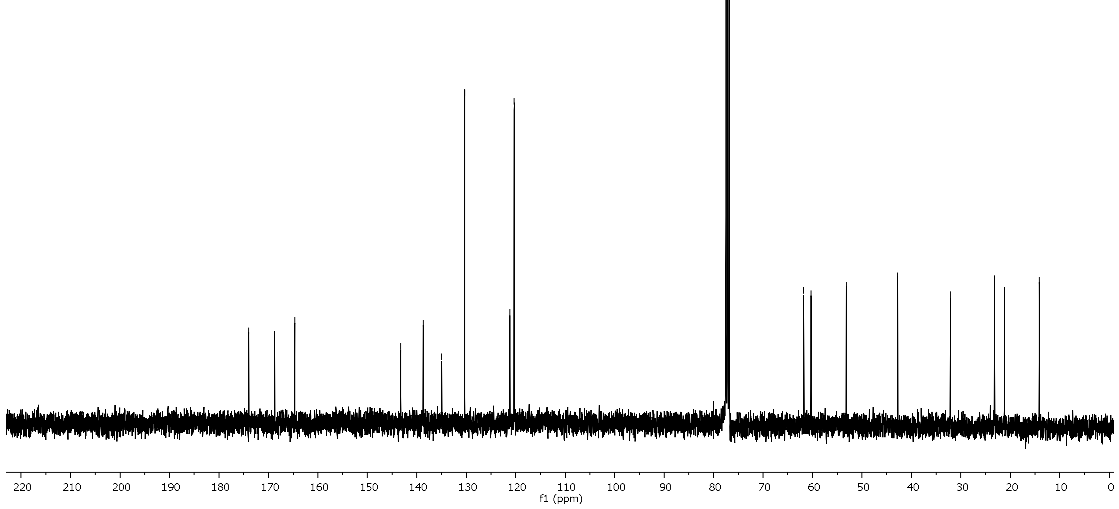

174.0
168.7
164.7
143.3
138.7
134.9
130.3
121.2
120.3
61.8
60.3
53.2
42.7
32.1
23.2
21.2
14.1
f1 (ppm)

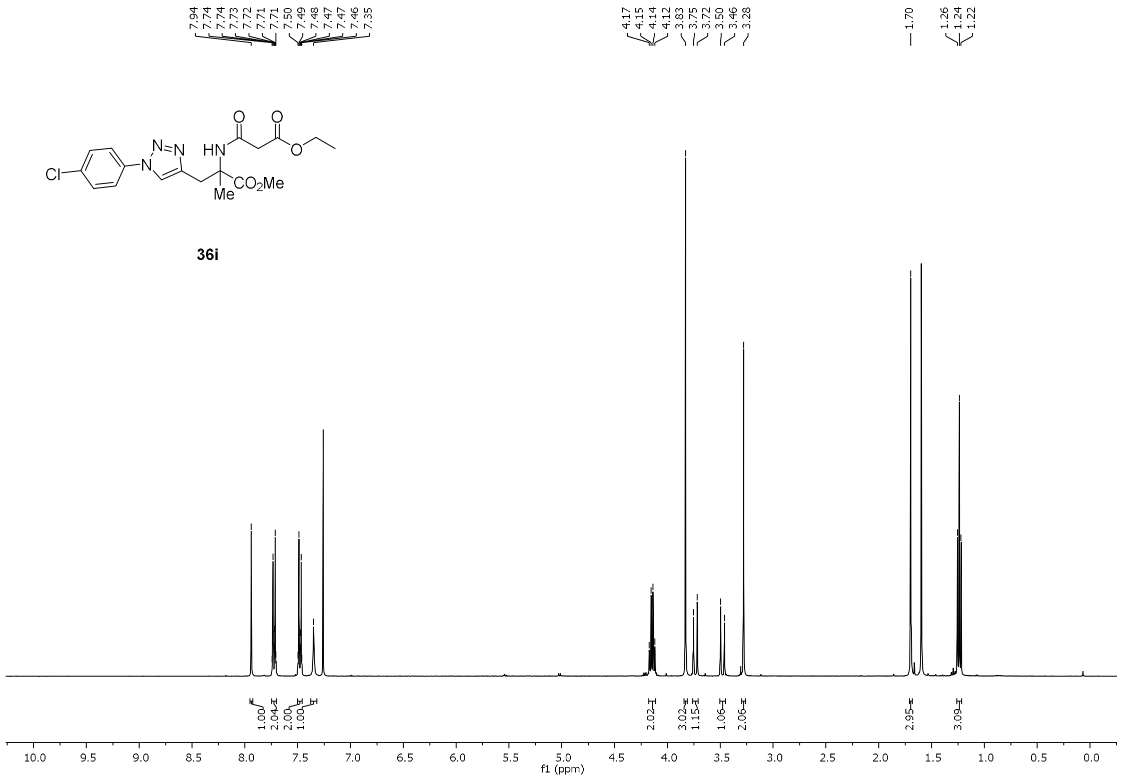
7.94
7.74
7.74
7.73
7.72
7.71
7.71
7.50
7.49
7.48
7.47
7.47
7.46
7.35
4.17
4.15
4.14
4.12
3.83
3.75
3.72
3.50
3.46
3.28
1.70
1.26
1.24
1.22
Cl
N
N=N
HN
O
O
O
CO2Me
Me
1.00
2.04
2.00
1.00
2.02
3.02
1.15
1.06
2.06
2.95
3.09
10.0
9.5
9.0
8.5
8.0
7.5
7.0
6.5
6.0
5.5
5.0
4.5
4.0
3.5
3.0
2.5
2.0
1.5
1.0
0.5
0.0
f1 (ppm)

36i

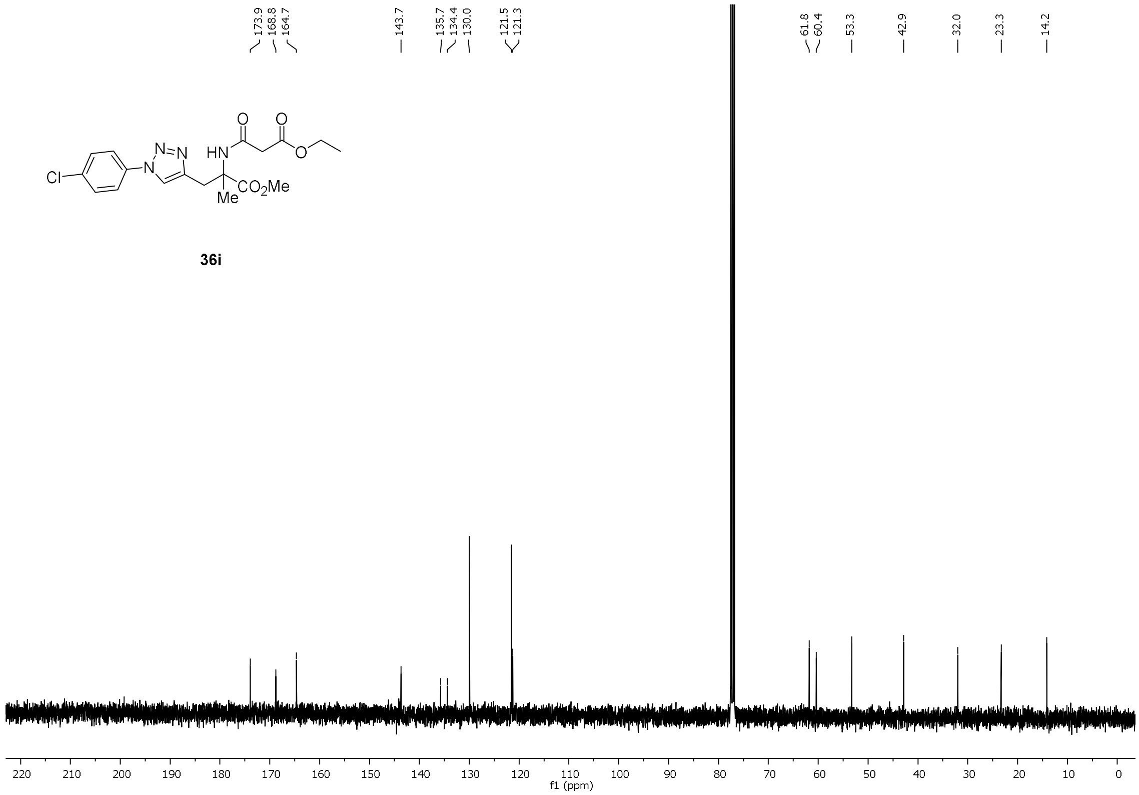
173.9
168.8
164.7
143.7
135.7
134.4
130.0
121.5
121.3
61.8
60.4
53.3
42.9
32.0
23.3
14.2
Cl
N
N=N
HN
O
O
O
CO2Me
Me
220
210
200
190
180
170
160
150
140
130
120
110
100
90
80
70
60
50
40
30
20
10
0
f1 (ppm)

36i

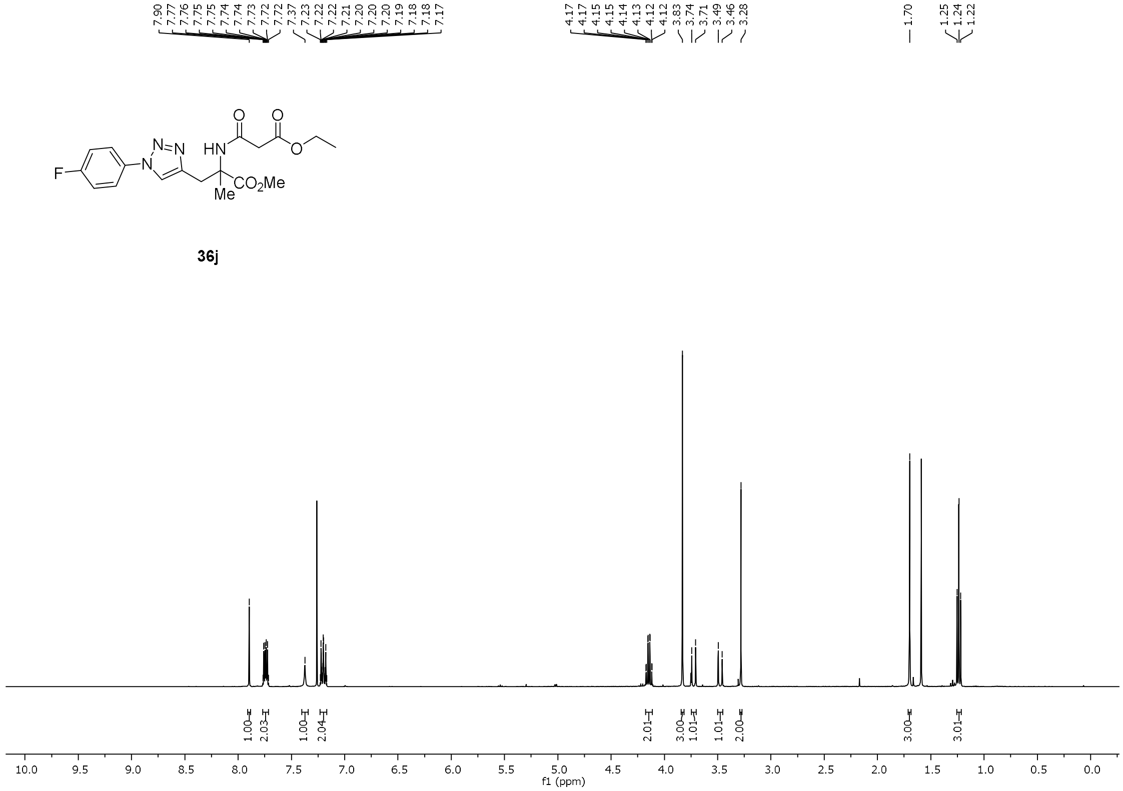
7.90 7.77 7.76 7.75 7.75 7.74 7.74 7.73 7.72 7.72 7.37 7.23 7.22 7.22 7.21 7.20 7.20 7.20 7.19 7.18 7.18 7.17
4.17 4.17 4.15 4.15 4.14 4.13 4.12 4.12 3.83 3.74 3.71 3.49 3.46 3.28
1.70
1.25 1.24 1.22
HN
O
F
N
CO2Me
Me
1.00 2.03 1.00 2.04 2.01 3.00 1.01 1.01 2.00 3.00 3.01
f1 (ppm)

36j

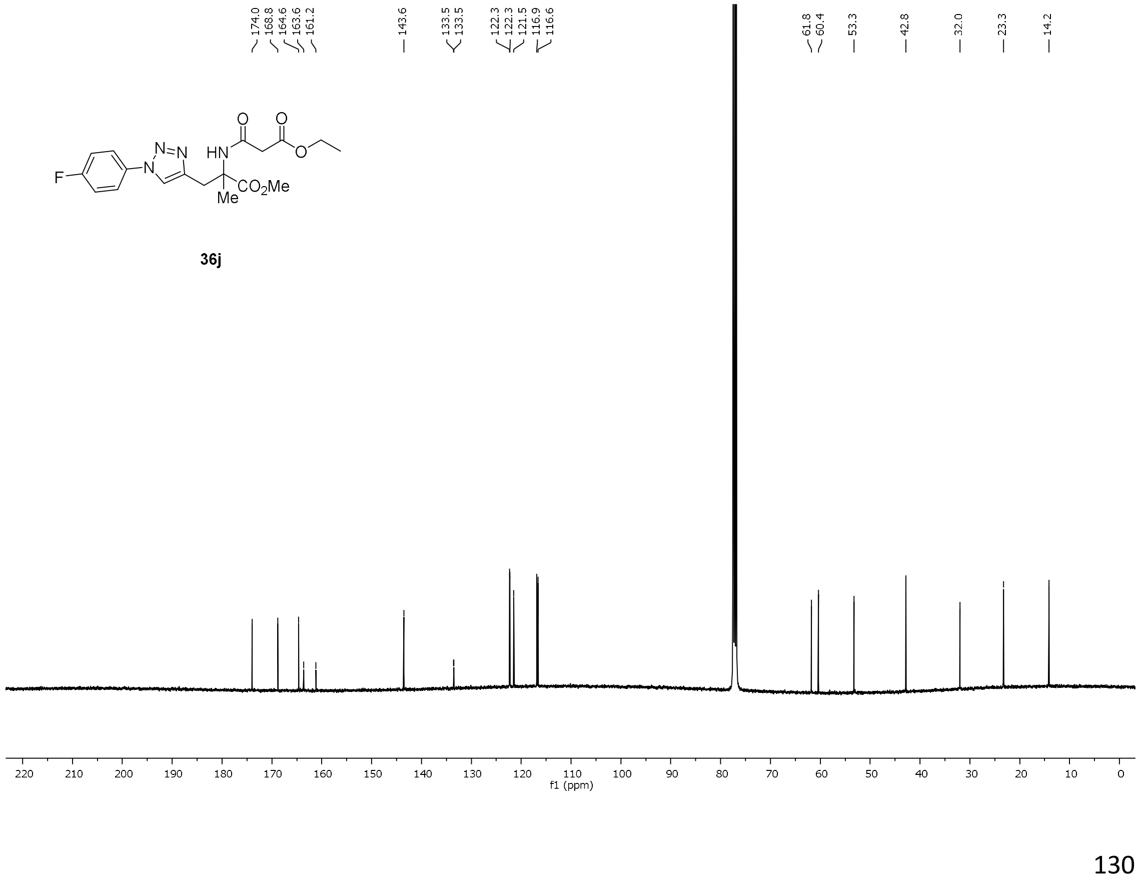
174.0 168.8 164.6 163.6 161.2
143.6
133.5 133.5
122.3 122.3 121.5 116.9 116.6
61.8 60.4
53.3
42.8
32.0
23.3
14.2
HN
O
F
N
CO2Me
Me
f1 (ppm)

36j

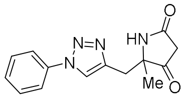

19

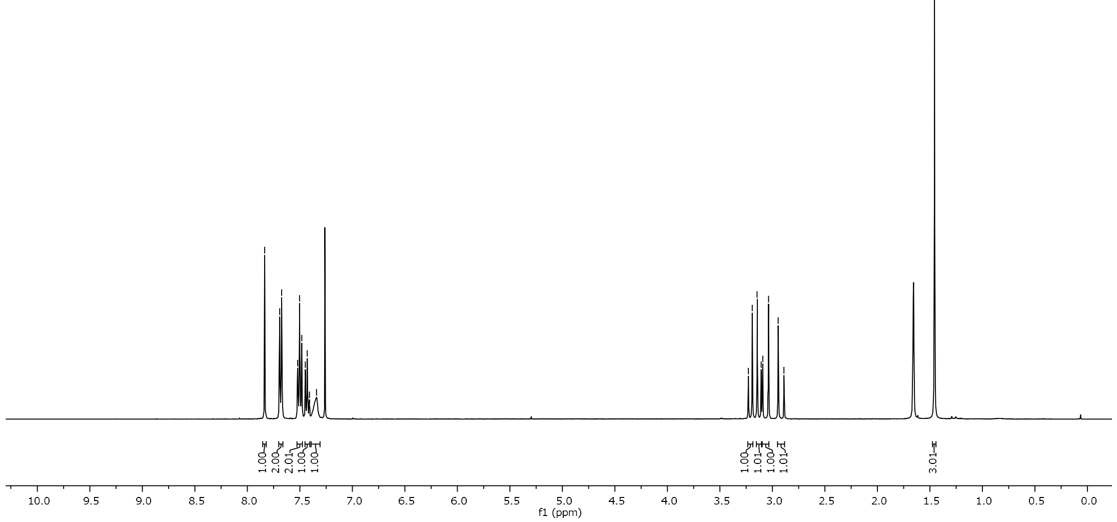
7.83
7.69
7.67
7.52
7.50
7.48
7.45
7.43
7.41
7.34
3.23
3.19
3.15
3.11
3.09
3.04
2.95
2.89
1.46
1.00
2.00
2.01
1.00
1.00
1.00
1.01
1.00
1.01
3.01
10.0 9.5 9.0 8.5 8.0 7.5 7.0 6.5 6.0 5.5 5.0 4.5 4.0 3.5 3.0 2.5 2.0 1.5 1.0 0.5 0.0
f1 (ppm)

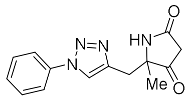

19

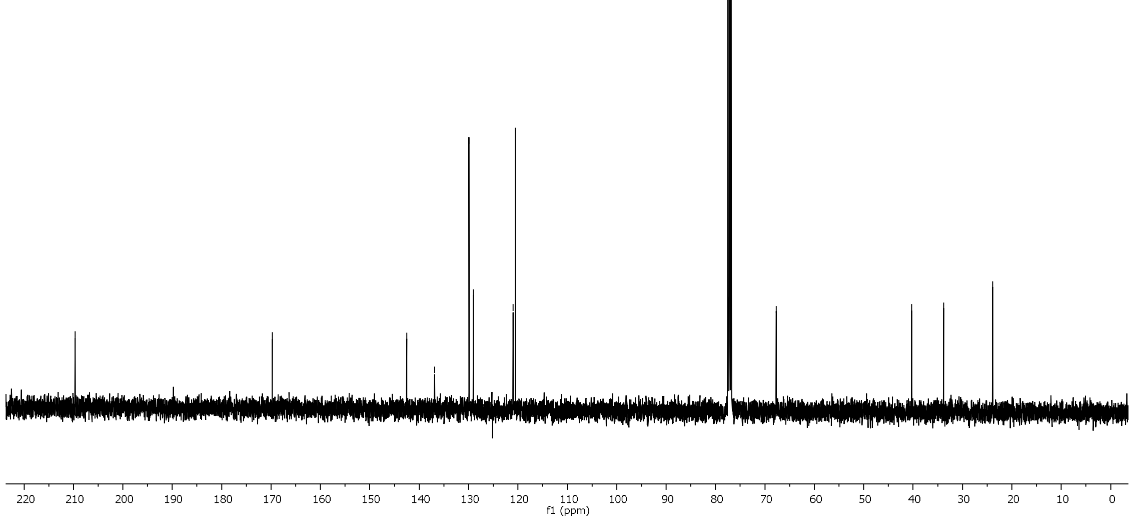
209.6
169.7
142.5
136.9
129.9
129.0
121.0
120.5
67.7
40.3
33.8
23.9
220 210 200 190 180 170 160 150 140 130 120 110 100 90 80 70 60 50 40 30 20 10 0
f1 (ppm)

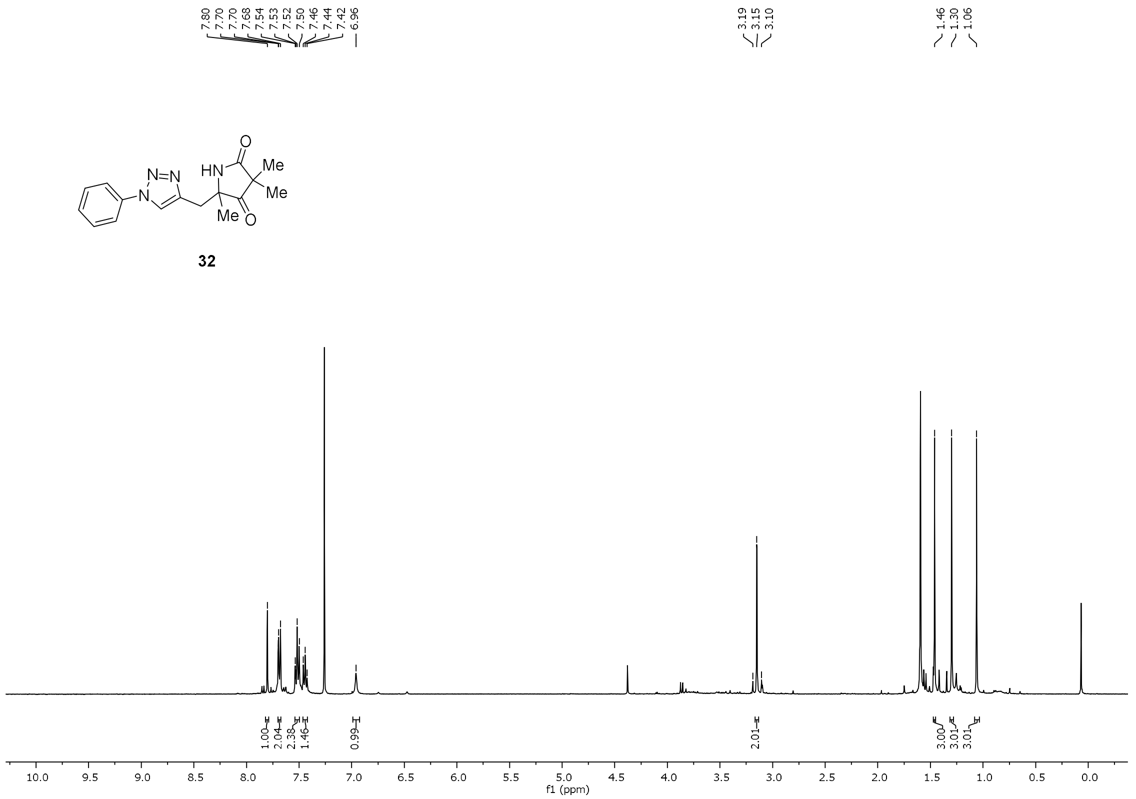
7.80
7.70
7.70
7.68
7.54
7.53
7.52
7.50
7.46
7.44
7.42
6.96
3.19
3.15
3.10
1.46
1.30
1.06
N=N
N
HN
O
Me
Me
Me
O
1.00
2.04
2.38
1.46
0.99
2.01
3.00
3.01
3.01
f1 (ppm)

32

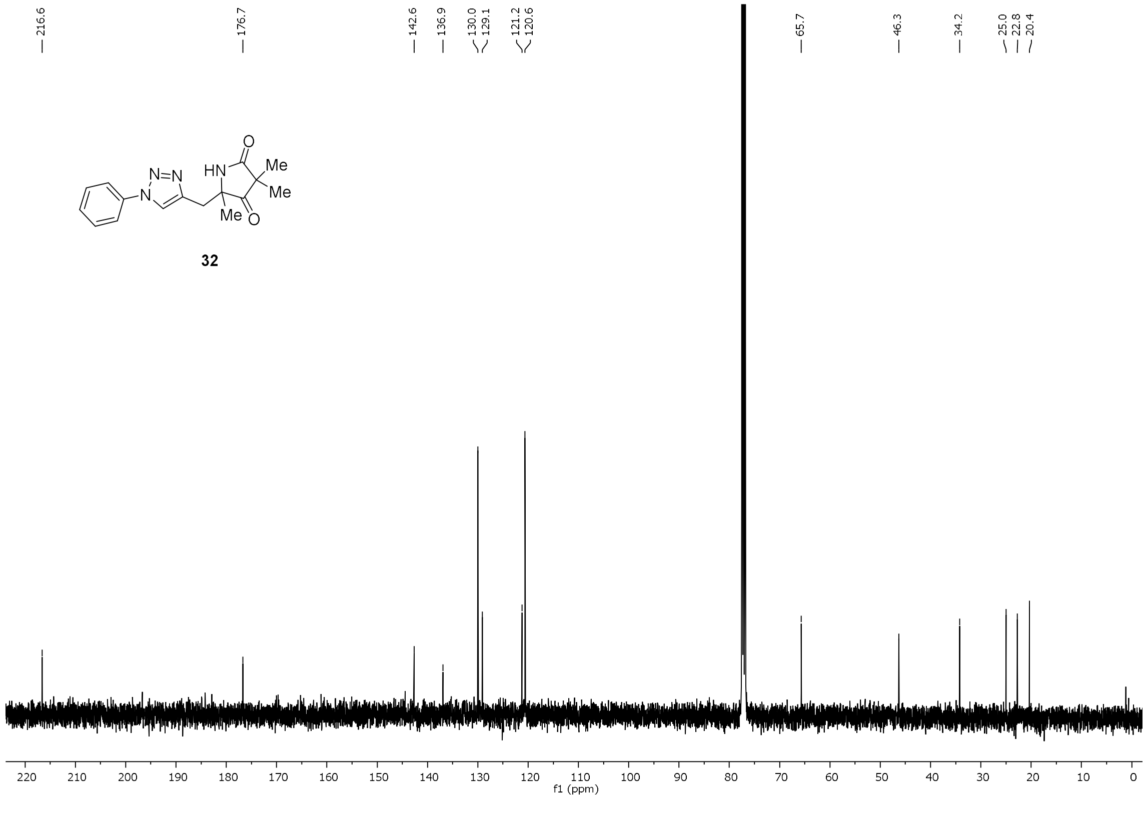
216.6
176.7
142.6
136.9
130.0
129.1
121.2
120.6
65.7
46.3
34.2
25.0
22.8
20.4
N=N
N
HN
O
Me
Me
Me
O
f1 (ppm)

32

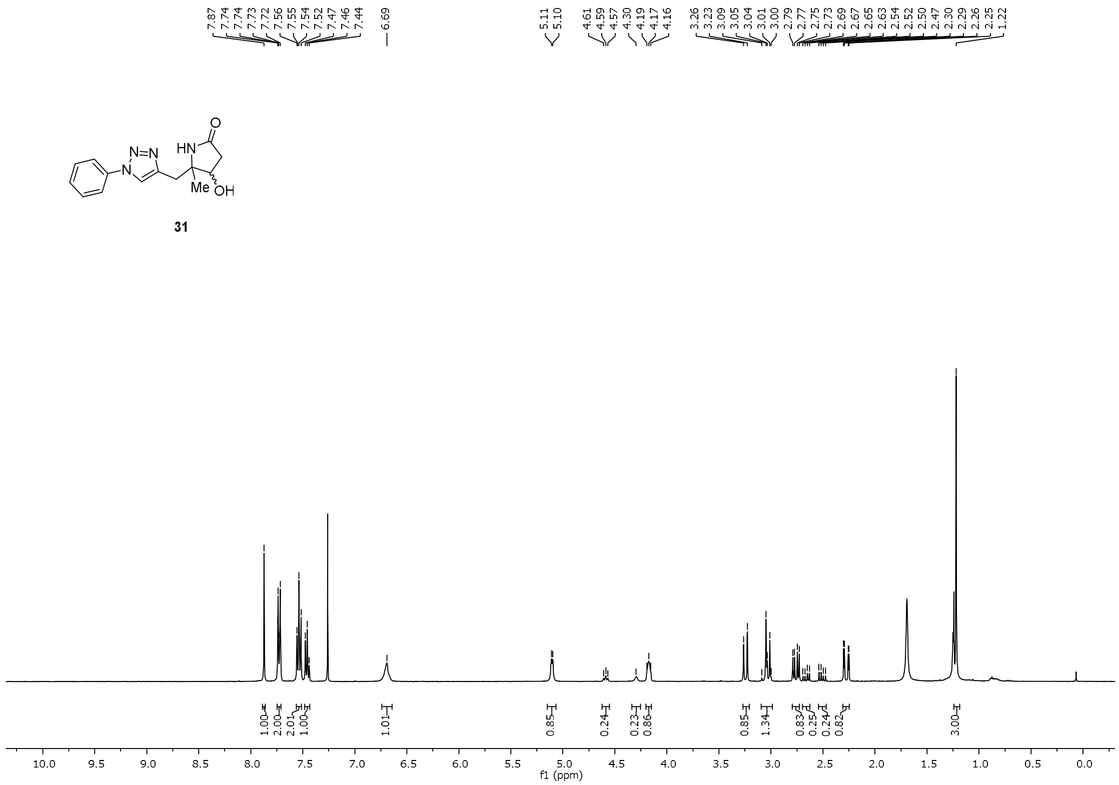
7.87 7.74 7.74 7.73 7.72 7.56 7.55 7.54 7.52 7.47 7.46 7.44
6.69
5.11 5.10
4.61 4.59 4.57 4.30 4.19 4.17 4.16
3.26 3.23 3.09 3.05 3.04 3.01 3.00 2.79 2.77 2.75 2.73 2.69 2.67 2.65 2.63 2.54 2.52 2.50 2.47 2.30 2.29 2.26 2.25 1.22
1.00 2.00 2.01 1.00 1.01 0.85 0.24 0.23 0.86 0.85 1.34 0.83 0.25 0.24 0.82 3.00
f1 (ppm)

31

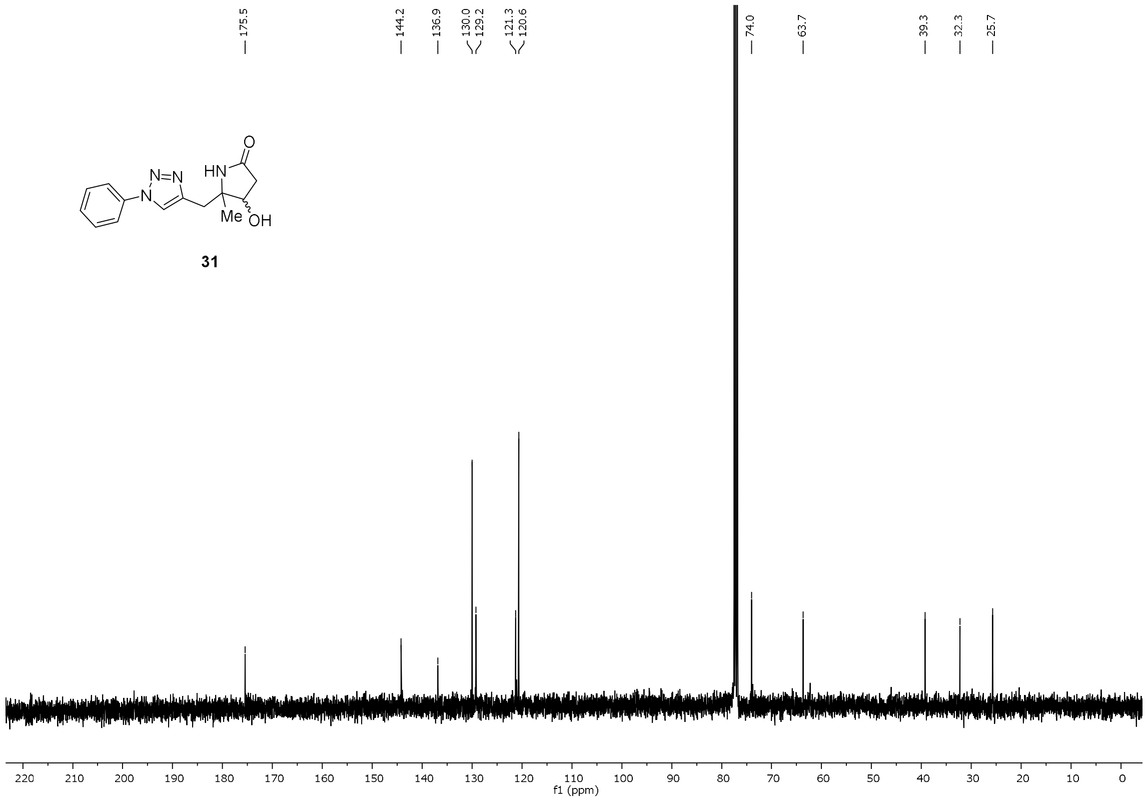
175.5
144.2
136.9
130.0 129.2
121.3 120.6
74.0
63.7
39.3
32.3
25.7
f1 (ppm)

31

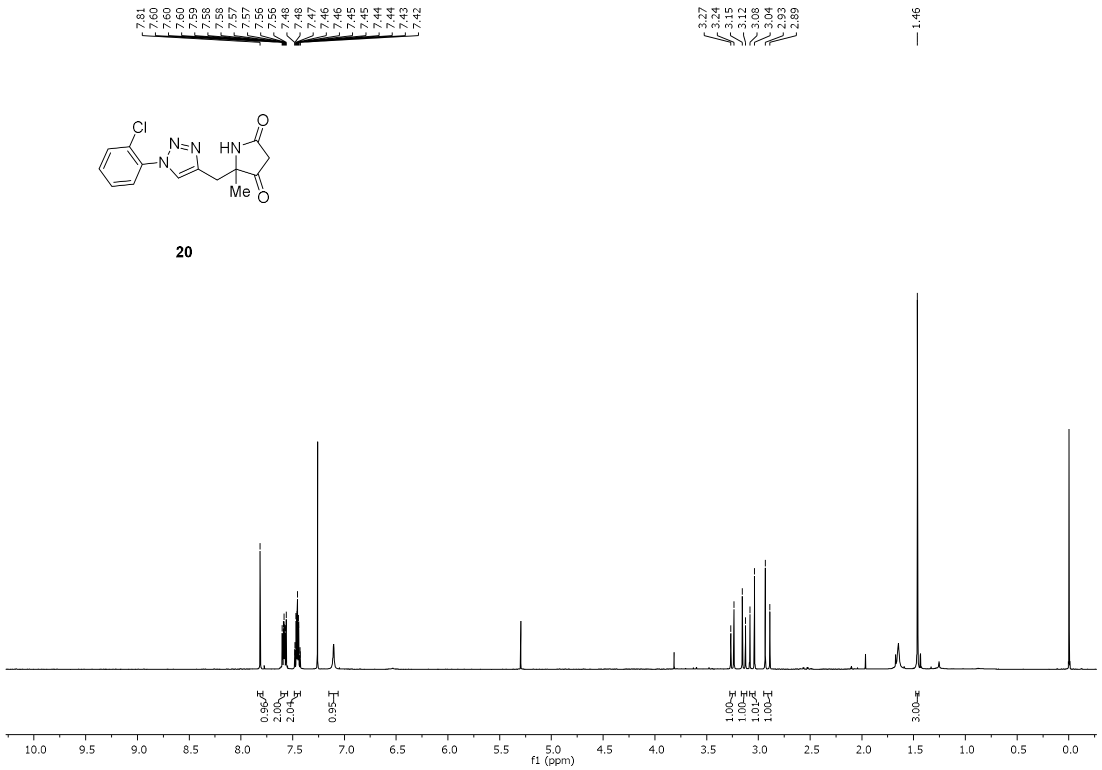
7.81 7.60 7.60 7.59 7.58 7.58 7.57 7.57 7.56 7.56 7.48 7.48 7.47 7.46 7.46 7.45 7.45 7.44 7.44 7.43 7.42
3.27 3.24 3.15 3.12 3.08 3.04 2.93 2.89
1.46
Cl
N N N
HN
O
Me
O
20
0.96 2.00 2.04 0.95
1.00 1.00 1.01 1.00
3.00
10.0 9.5 9.0 8.5 8.0 7.5 7.0 6.5 6.0 5.5 5.0 4.5 4.0 3.5 3.0 2.5 2.0 1.5 1.0 0.5 0.0
f1 (ppm)

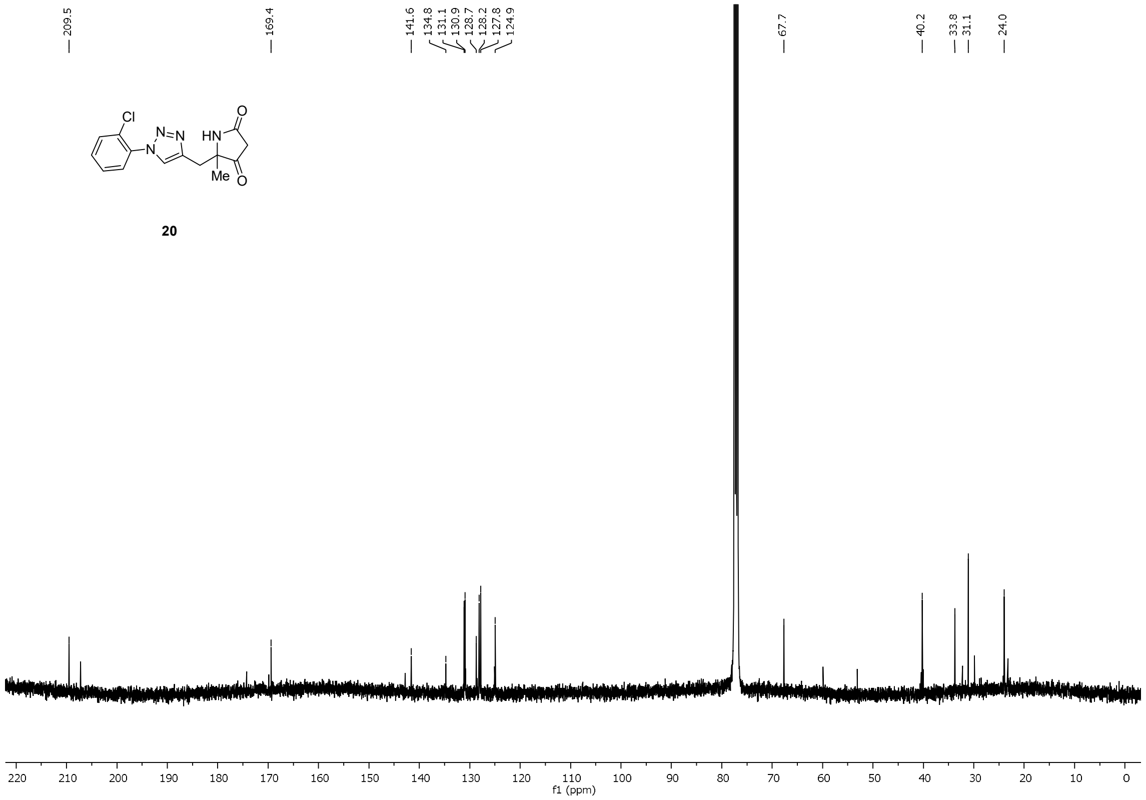
209.5
169.4
141.6 134.8 131.1 130.9 128.7 128.2 127.8 124.9
67.7
40.2 33.8 31.1 24.0
Cl
N N N
HN
O
Me
O
20
220 210 200 190 180 170 160 150 140 130 120 110 100 90 80 70 60 50 40 30 20 10 0
f1 (ppm)

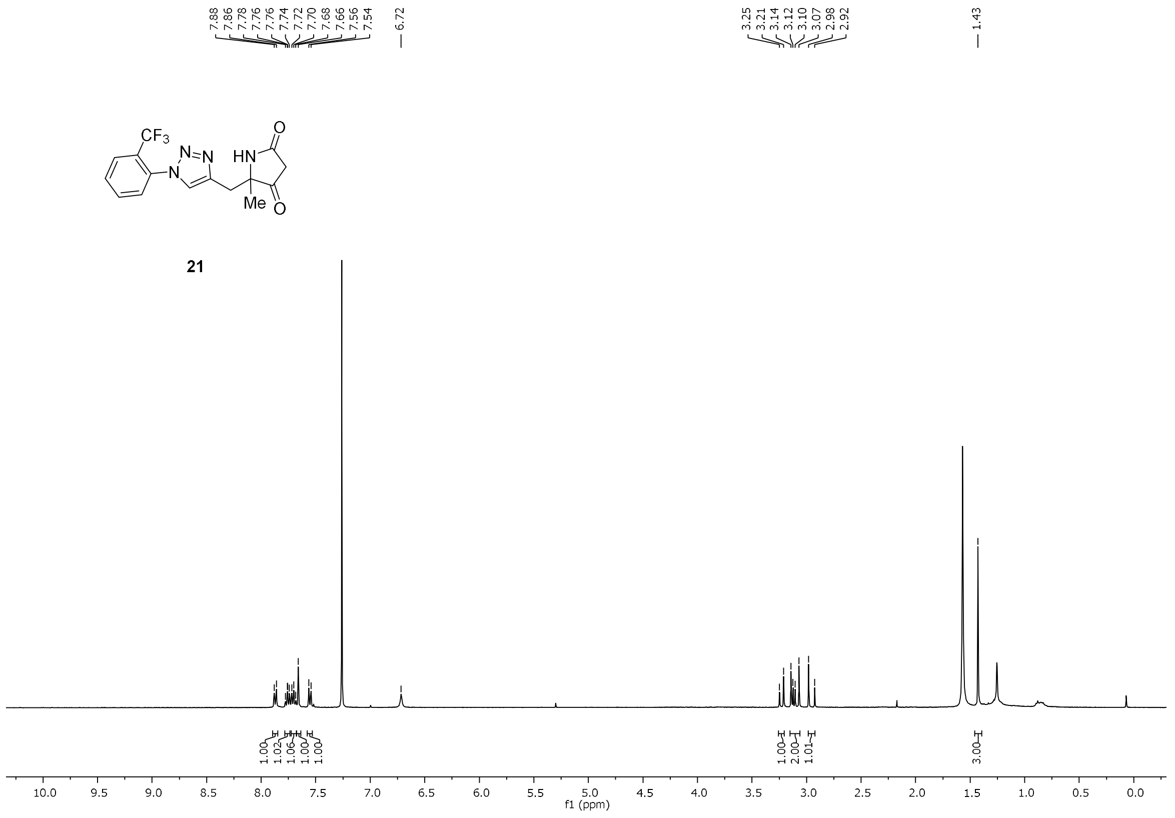

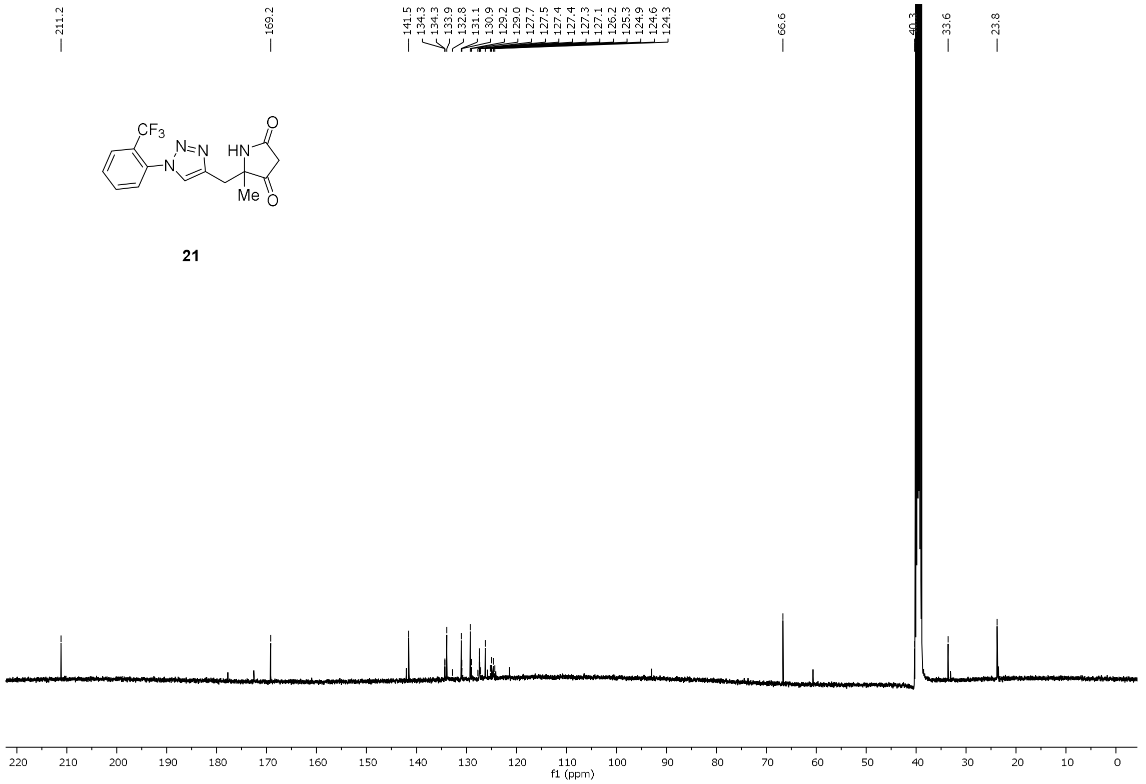

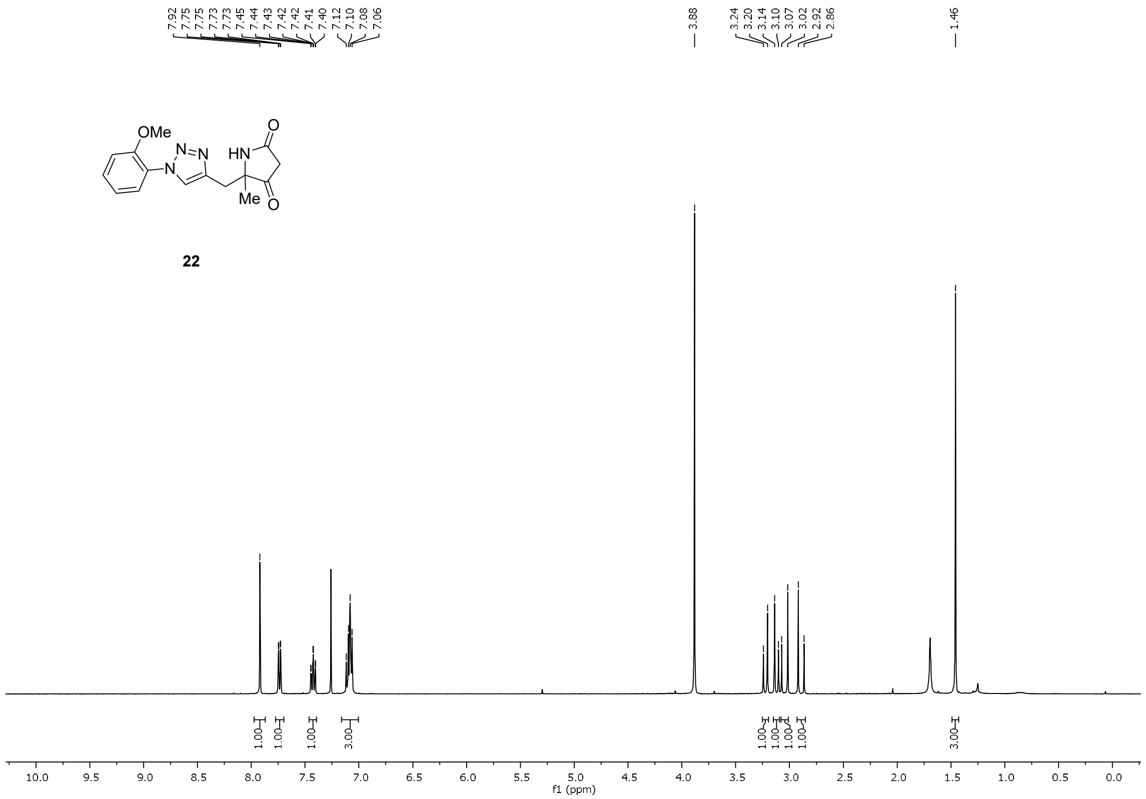

22

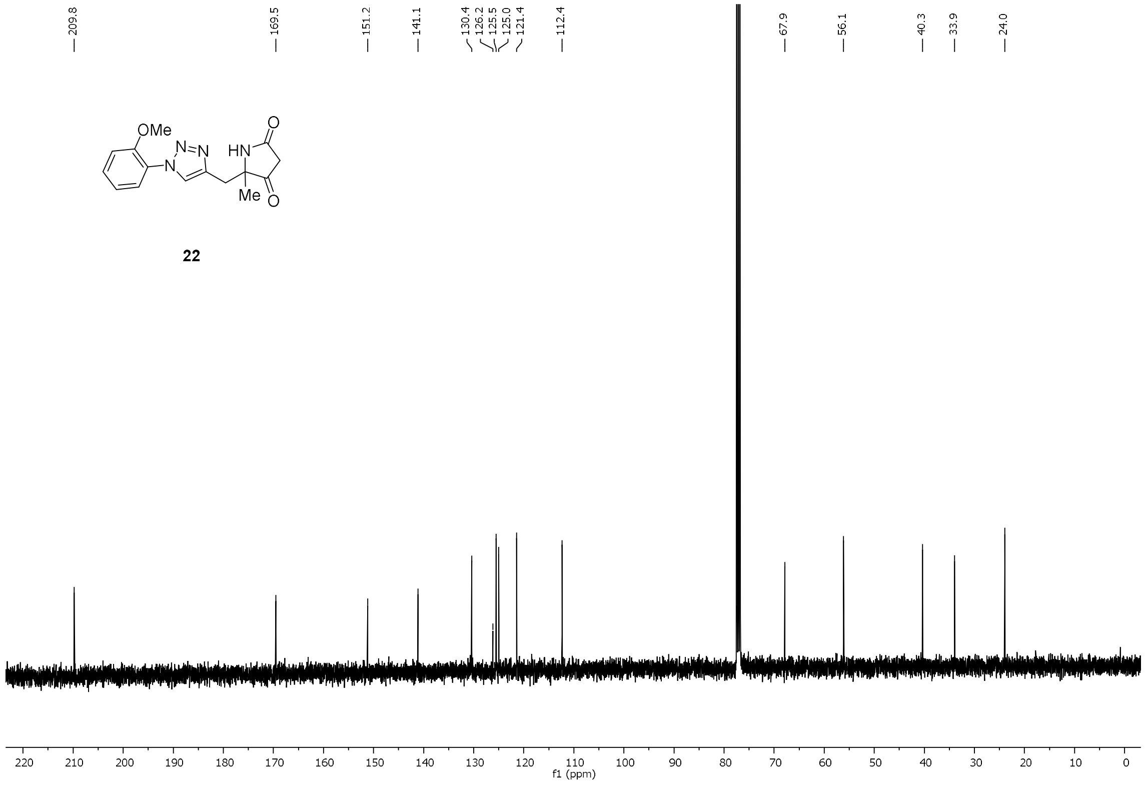

22

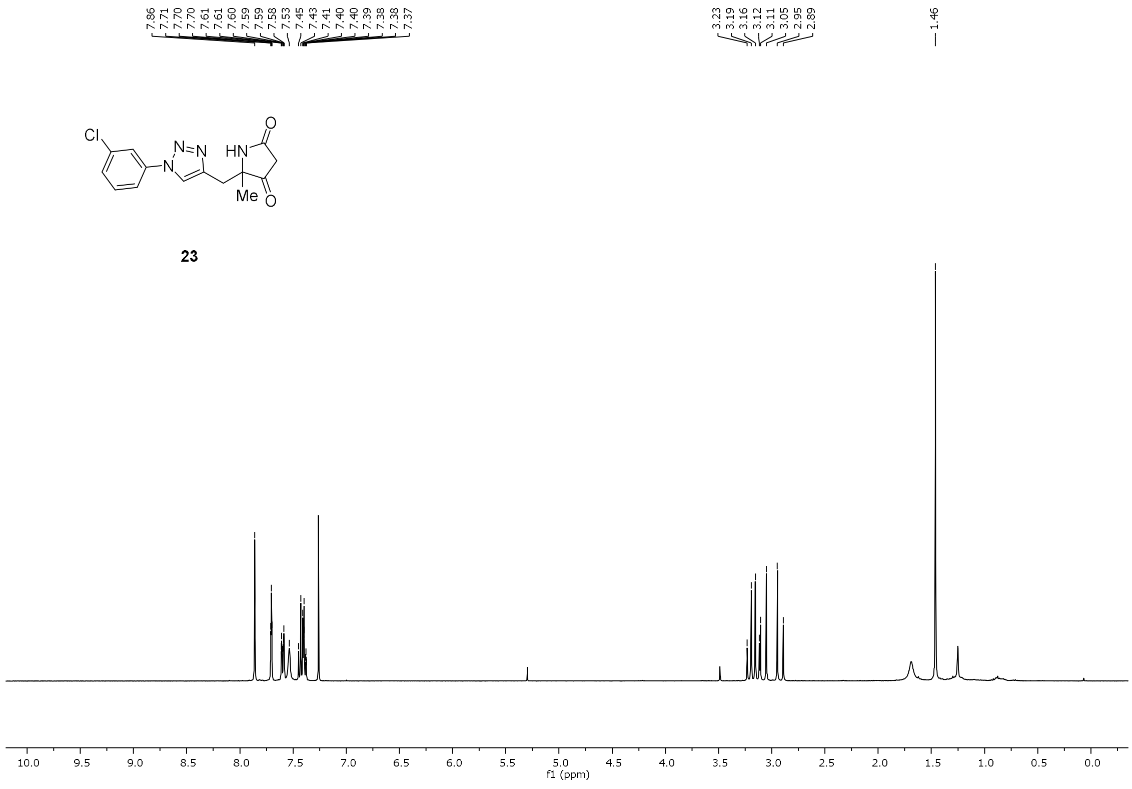
7.86 7.71 7.70 7.70 7.61 7.60 7.59 7.59 7.58 7.53 7.45 7.43 7.41 7.40 7.40 7.39 7.38 7.38 7.37
3.23 3.19 3.16 3.12 3.11 3.05 2.95 2.89
1.46
Cl
N
N=N
HN
O
Me
O
23
f1 (ppm)

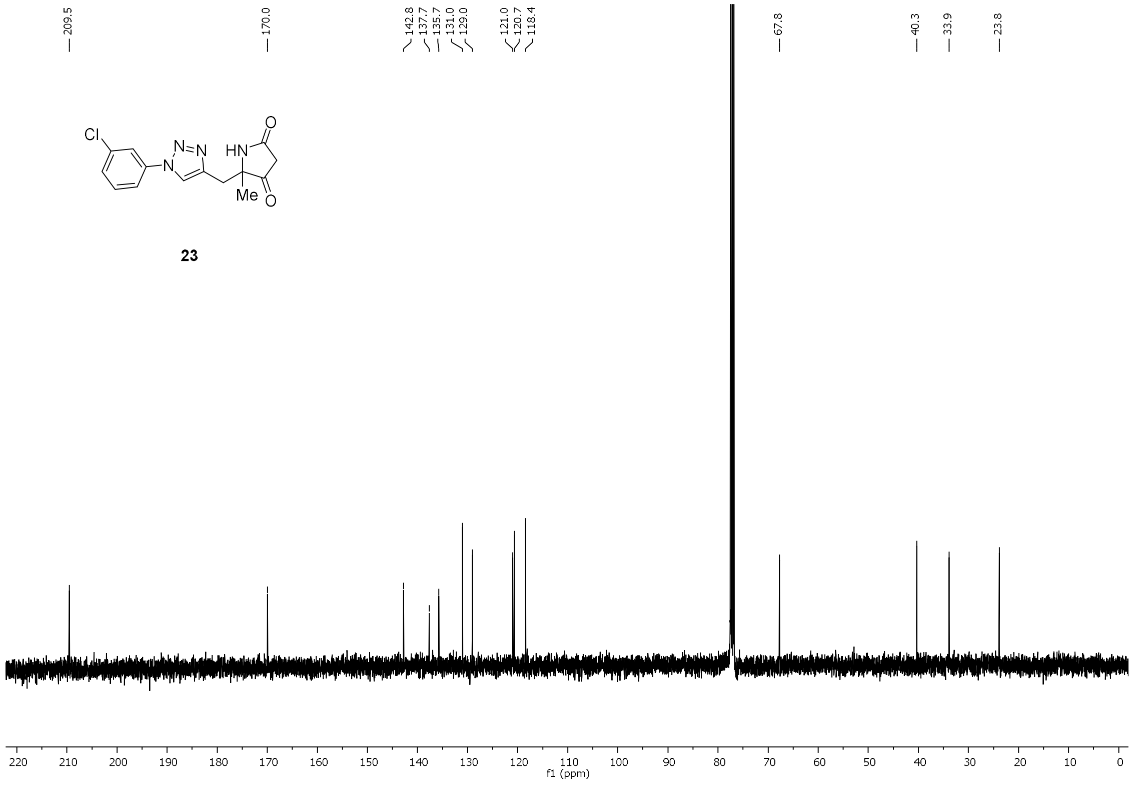
209.5
170.0
142.8 137.7 135.7 131.0 129.0
121.0 120.7 118.4
67.8
40.3
33.9
23.8
Cl
N
N=N
HN
O
Me
O
23
f1 (ppm)

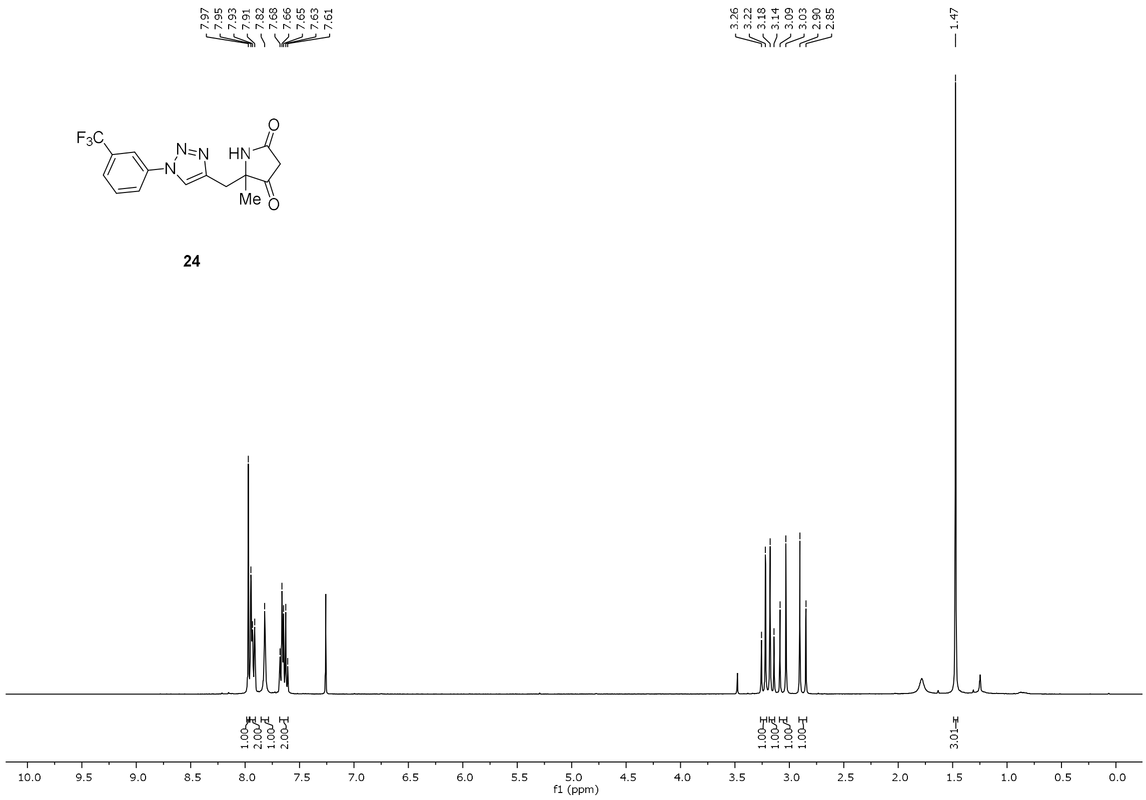

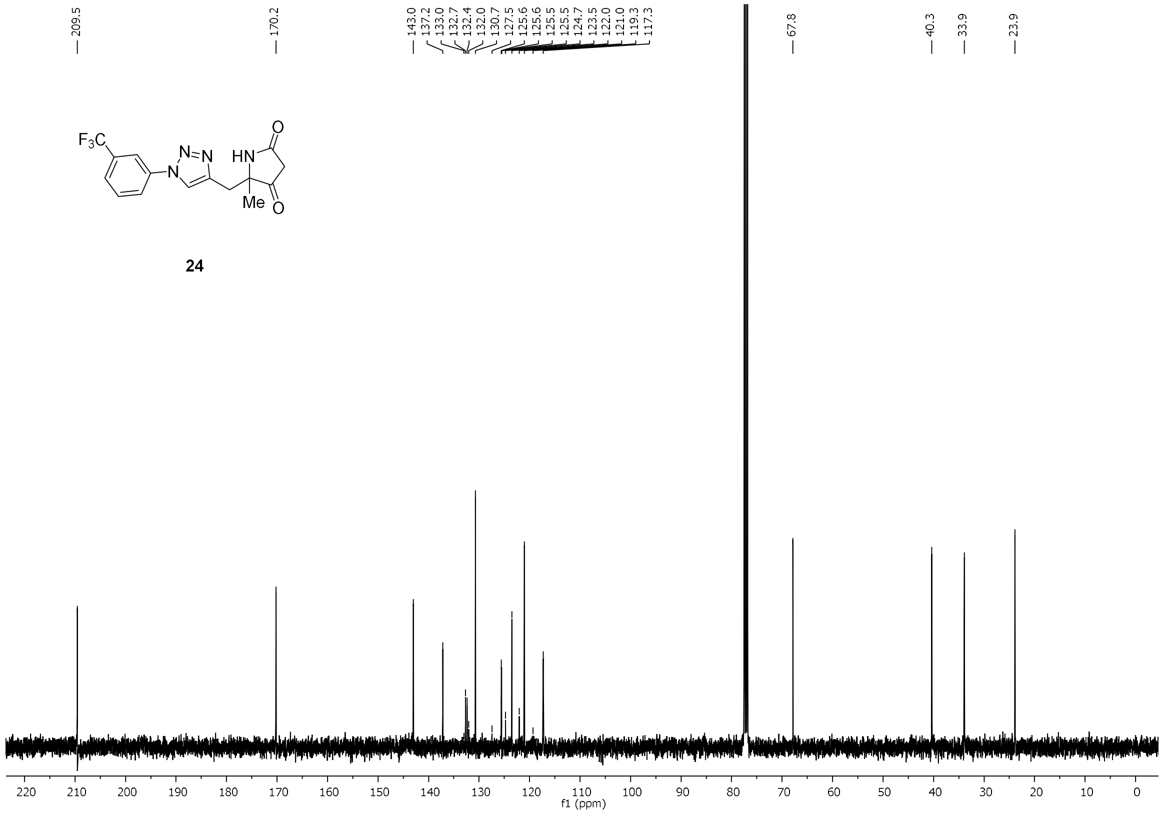

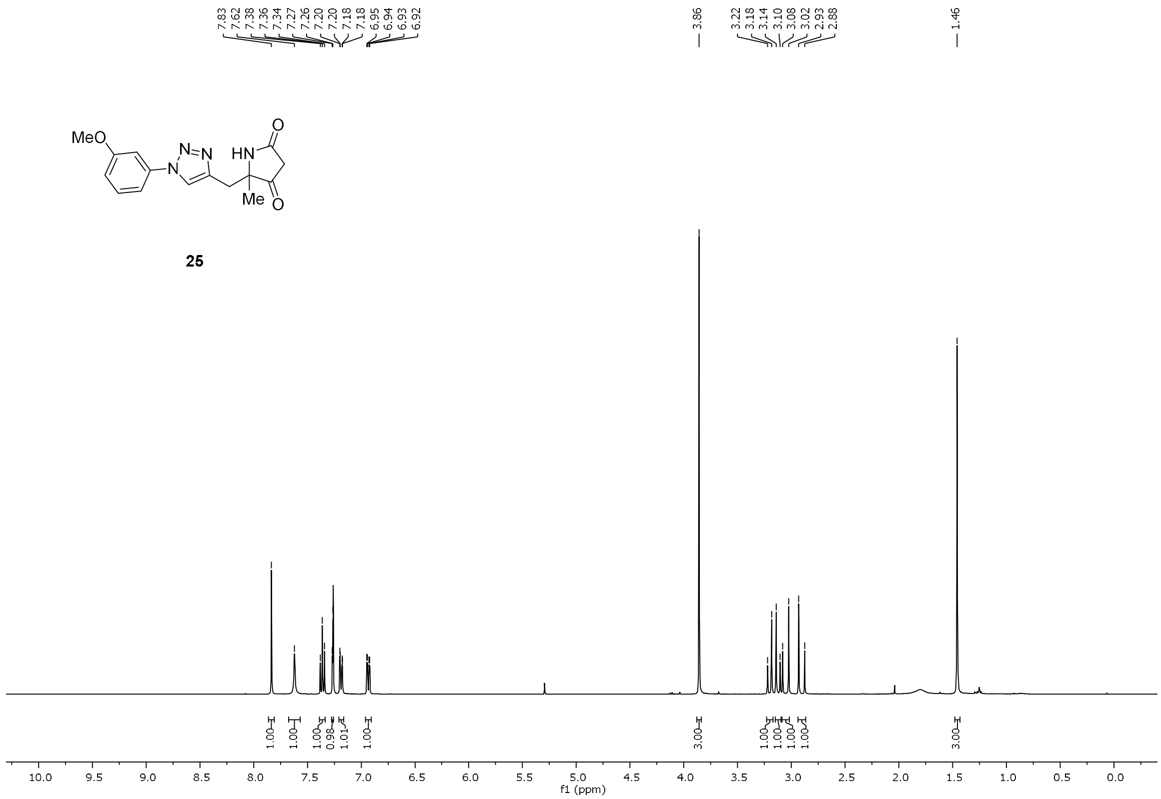
7.83
7.62
7.38
7.36
7.34
7.27
7.26
7.20
7.20
7.18
7.18
6.95
6.94
6.93
6.92
3.86
3.22
3.18
3.14
3.10
3.08
3.02
2.93
2.88
1.46
MeO
N
N
N
HN
O
Me
O
25
1.00
1.00
1.00
0.98
1.01
1.00
3.00
1.00
1.00
1.00
1.00
3.00
10.0
9.5
9.0
8.5
8.0
7.5
7.0
6.5
6.0
5.5
5.0
4.5
4.0
3.5
3.0
2.5
2.0
1.5
1.0
0.5
0.0
f1 (ppm)

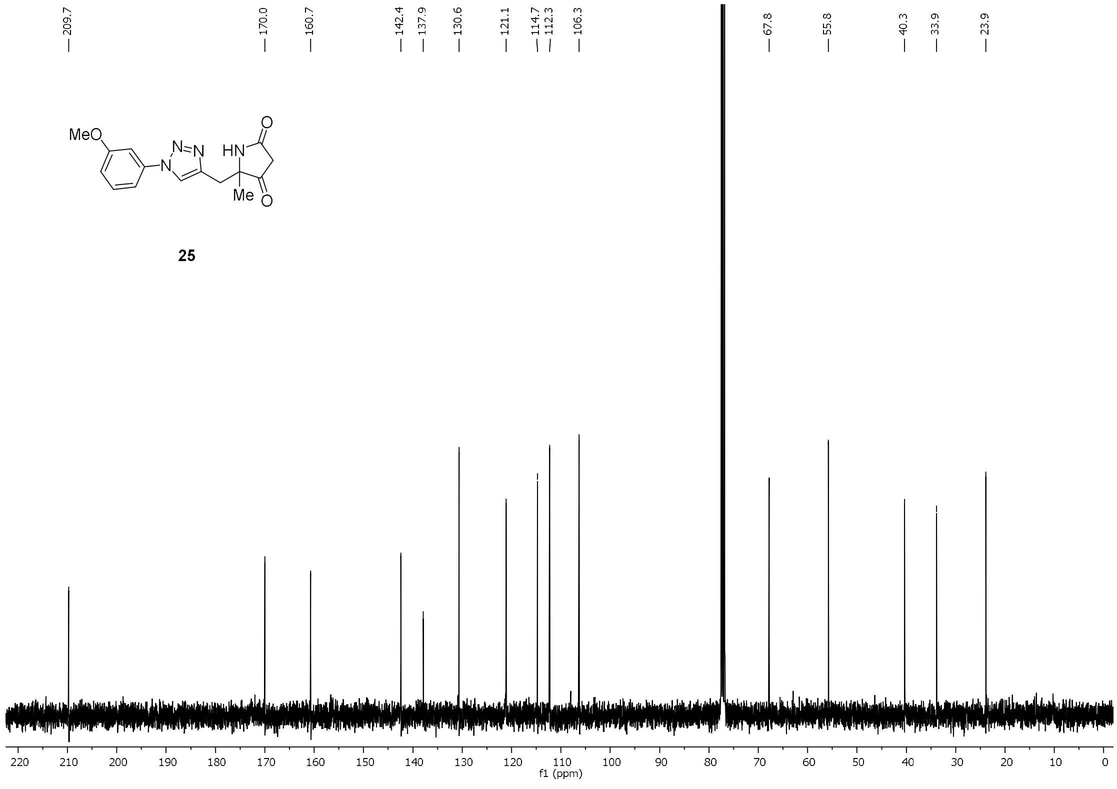
209.7
170.0
160.7
142.4
137.9
130.6
121.1
114.7
112.3
106.3
67.8
55.8
40.3
33.9
23.9
MeO
N
N
N
HN
O
Me
O
25
220
210
200
190
180
170
160
150
140
130
120
110
100
90
80
70
60
50
40
30
20
10
0
f1 (ppm)

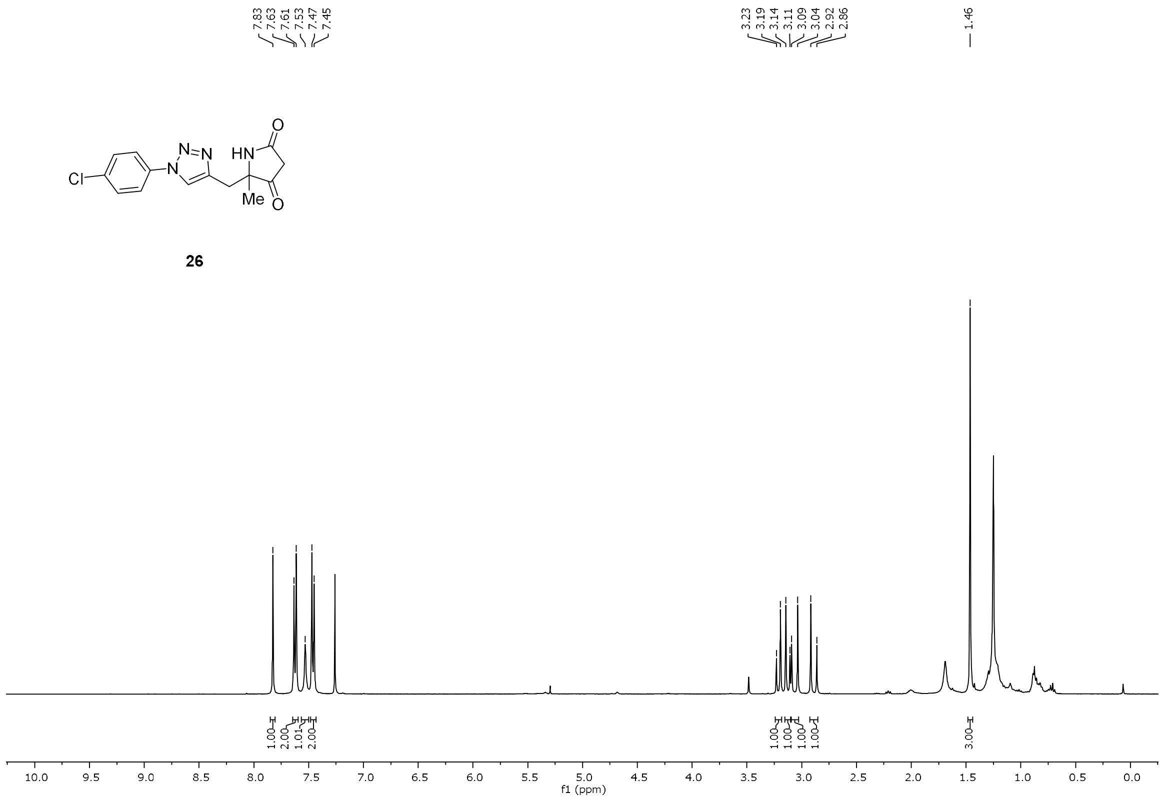
7.83
7.63
7.61
7.53
7.47
7.45
3.23
3.19
3.14
3.11
3.09
3.04
2.92
2.86
1.46
Cl
N
N=N
HN
O
Me
O
1.00
2.00
1.01
2.00
1.00
1.00
1.00
1.00
3.00
f1 (ppm)

26

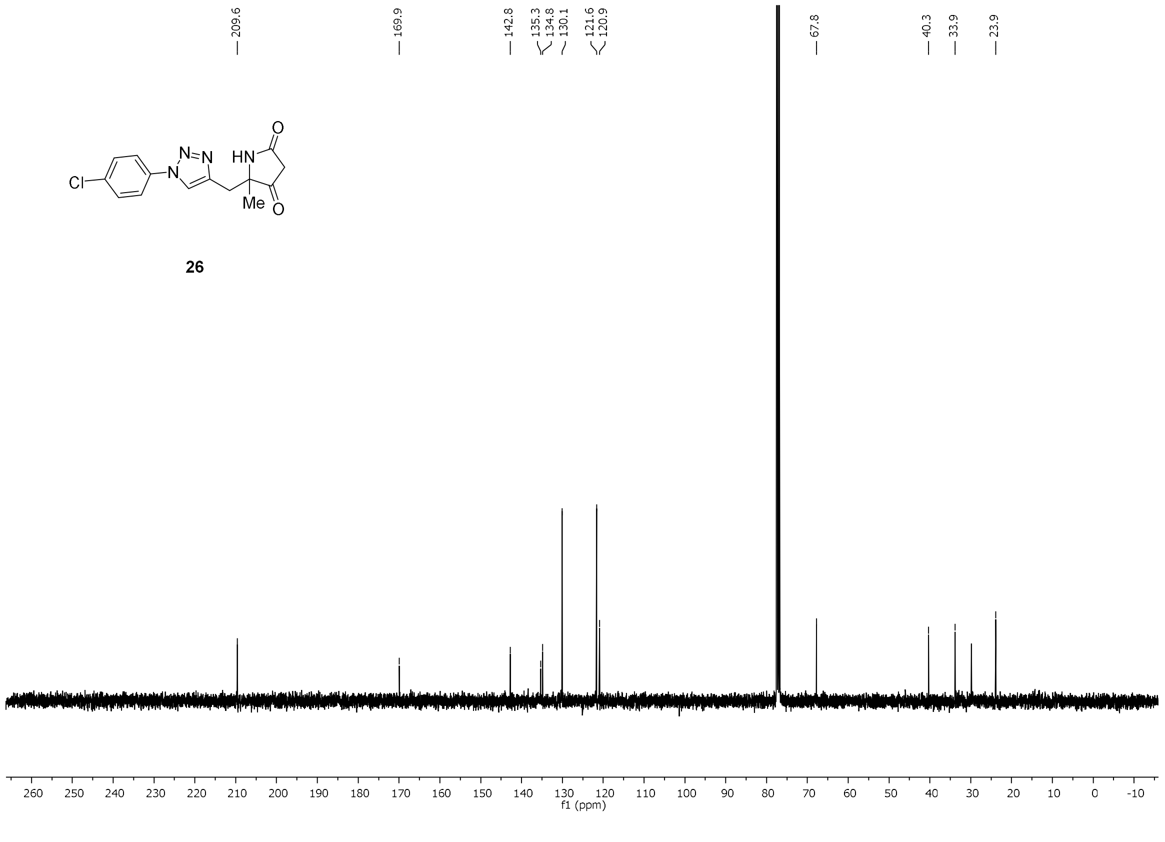
209.6
169.9
142.8
135.3
134.8
130.1
121.6
120.9
67.8
40.3
33.9
23.9
Cl
N
N=N
HN
O
Me
O
f1 (ppm)

26

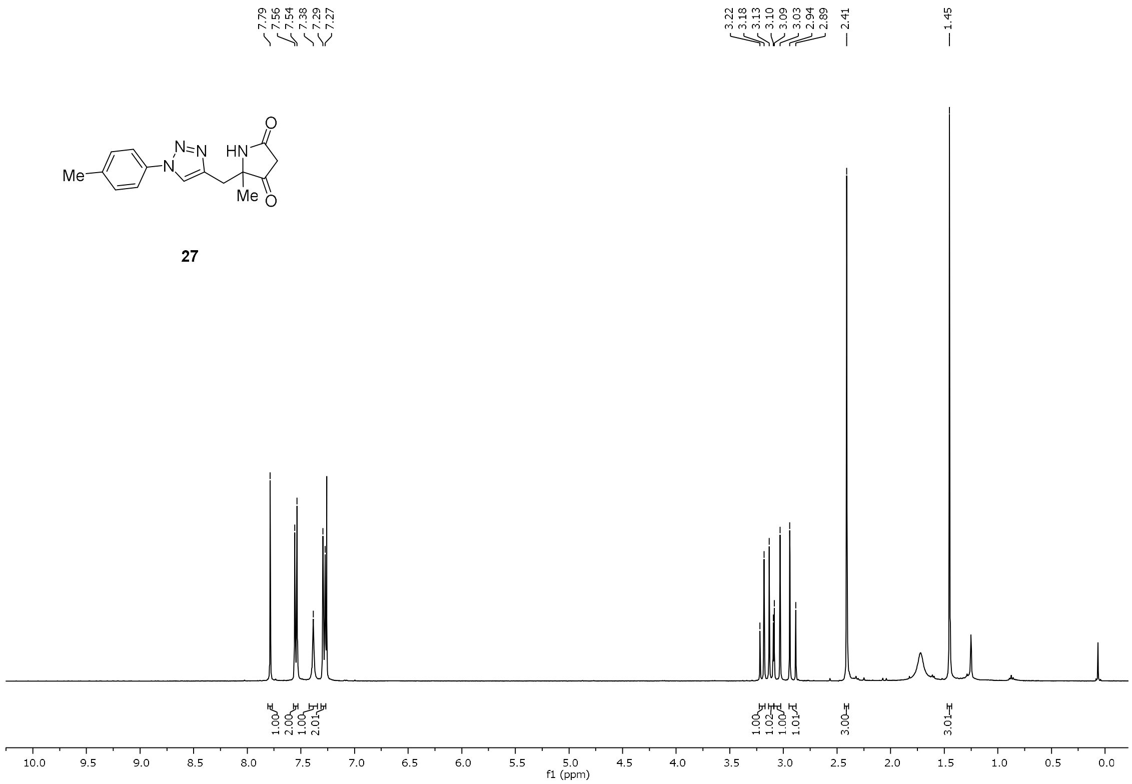
7.79
7.56
7.54
7.38
7.29
7.27
3.22
3.18
3.13
3.10
3.09
3.03
2.94
2.89
2.41
1.45
Me
N
N=N
HN
O
Me
O
1.00
2.00
1.00
2.01
1.00
1.02
1.00
1.01
3.00
3.01
f1 (ppm)

27

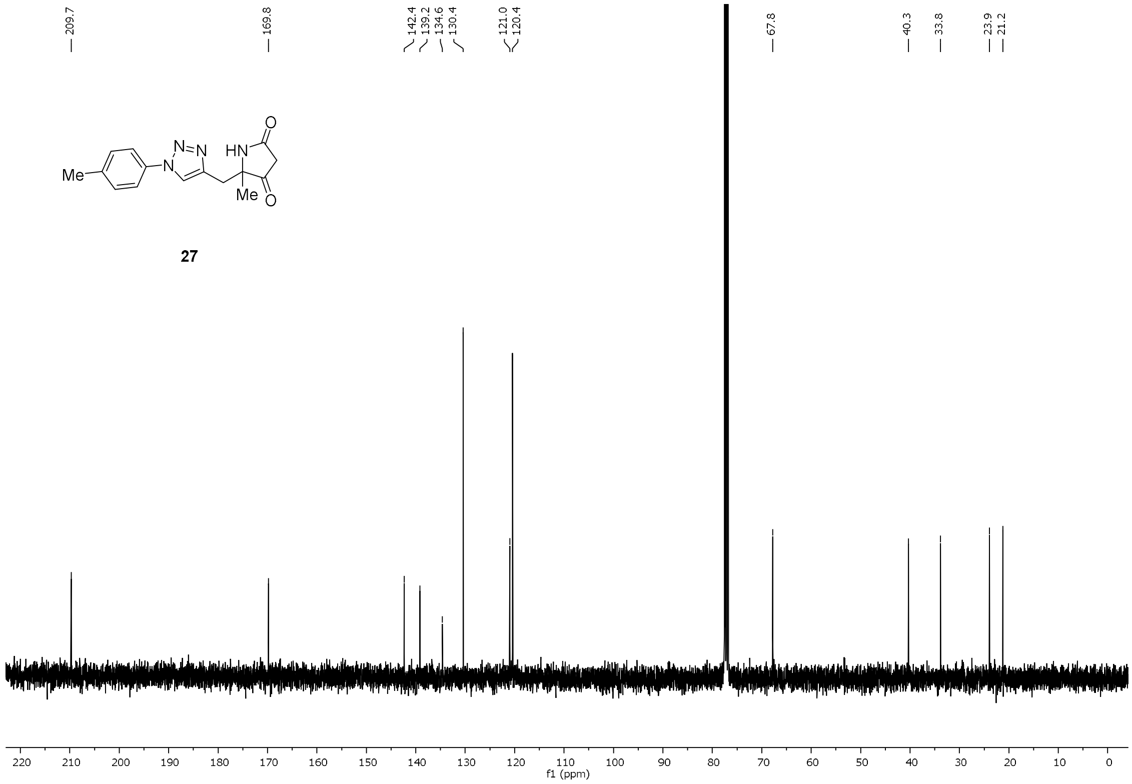
209.7
169.8
142.4
139.2
134.6
130.4
121.0
120.4
67.8
40.3
33.8
23.9
21.2
Me
N
N=N
HN
O
Me
O
f1 (ppm)

27

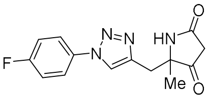
F
N
N=N
HN
O
Me
O

28

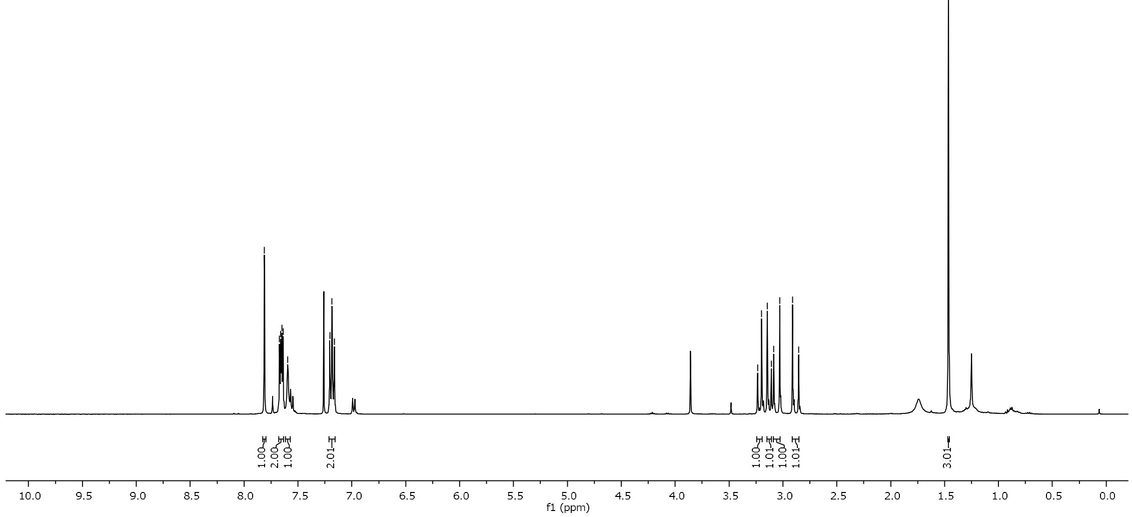
7.81
7.67
7.66
7.65
7.65
7.64
7.59
7.20
7.18
7.16
3.23
3.20
3.15
3.11
3.08
3.03
2.91
2.85
1.46
1.00
2.00
1.00
2.01
1.00
1.01
1.01
1.00
1.01
3.01
10.0
9.5
9.0
8.5
8.0
7.5
7.0
6.5
6.0
5.5
5.0
4.5
4.0
3.5
3.0
2.5
2.0
1.5
1.0
0.5
0.0
f1 (ppm)

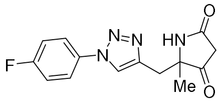
F
N
N=N
HN
O
Me
O

28

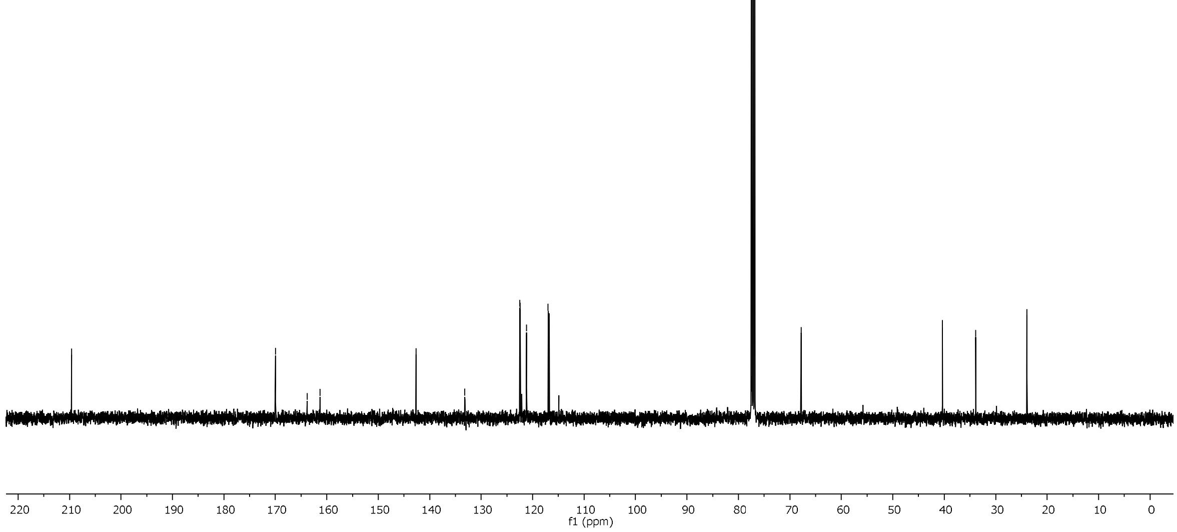
209.6
170.0
163.8
161.3
142.7
133.2
122.5
122.4
121.2
117.0
116.8
67.8
40.4
33.9
23.9
220
210
200
190
180
170
160
150
140
130
120
110
100
90
80
70
60
50
40
30
20
10
0
f1 (ppm)

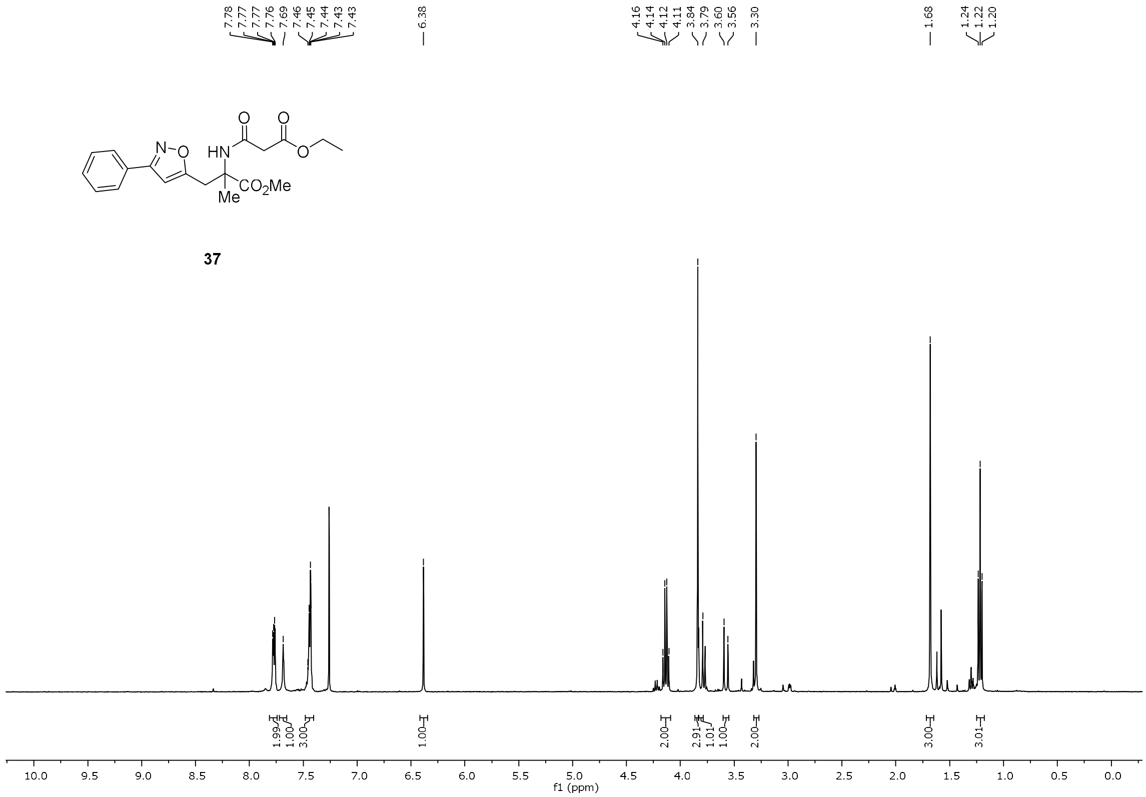
7.78 7.77 7.77 7.76 7.69 7.46 7.45 7.44 7.43 7.43
6.38
4.16 4.14 4.12 4.11 3.84 3.79 3.60 3.56 3.30
1.68
1.24 1.22 1.20
HN
N-O
CO2Me
Me
37
1.99 1.00 3.00
1.00
2.00
2.91 1.01 1.00
2.00
3.00
3.01
f1 (ppm)

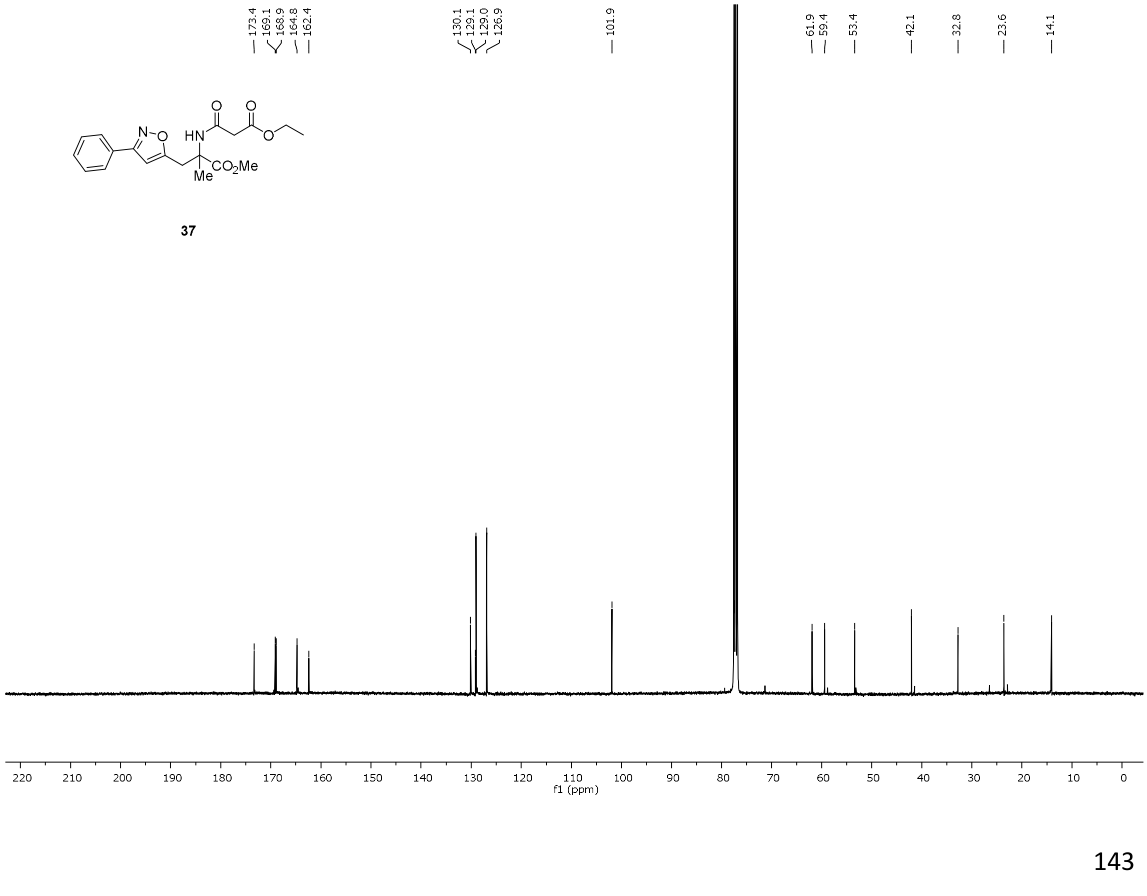
173.4 169.1 168.9 164.8 162.4
130.1 129.1 129.0 126.9
101.9
61.9 59.4
53.4
42.1
32.8
23.6
14.1
HN
N-O
CO2Me
Me
37
f1 (ppm)

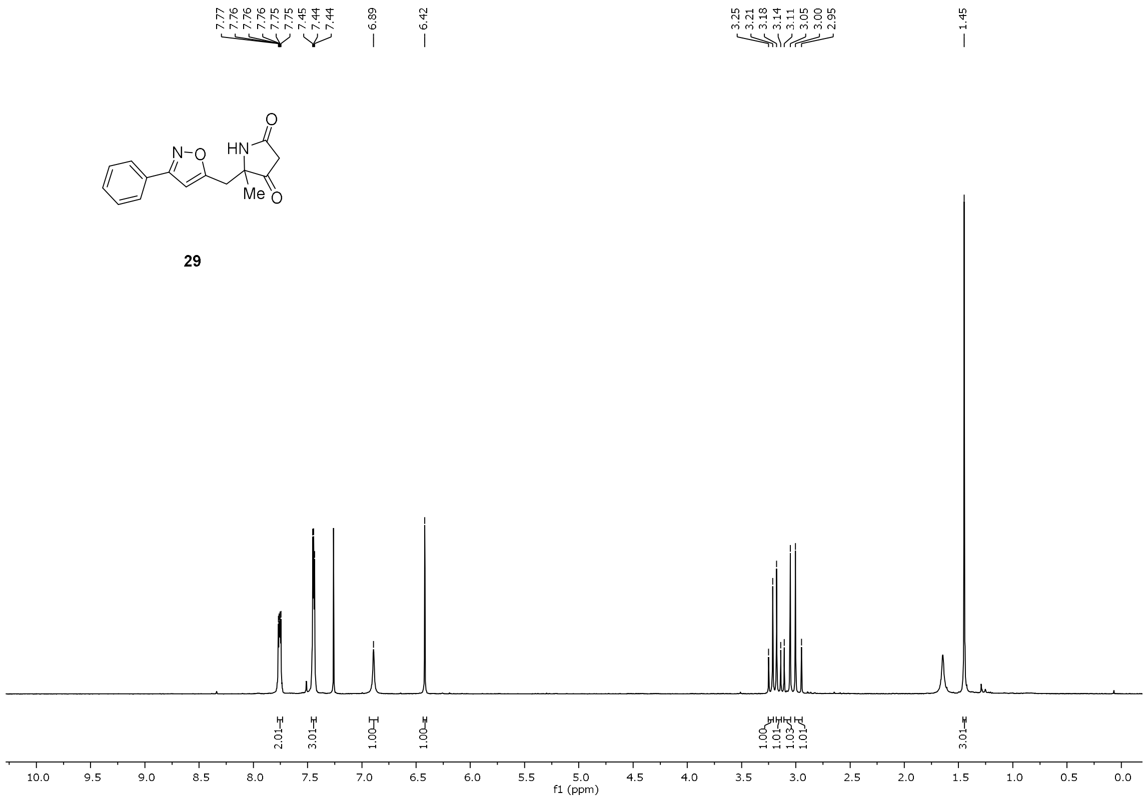
7.77 7.76 7.76 7.76 7.75 7.75 7.45 7.44 7.44
6.89
6.42
3.25 3.21 3.18 3.14 3.11 3.05 3.00 2.95
1.45
HN
N–O
O
Me
29
2.01
3.01
1.00
1.00
1.00 1.01 1.01 1.03 1.01
3.01
f1 (ppm)

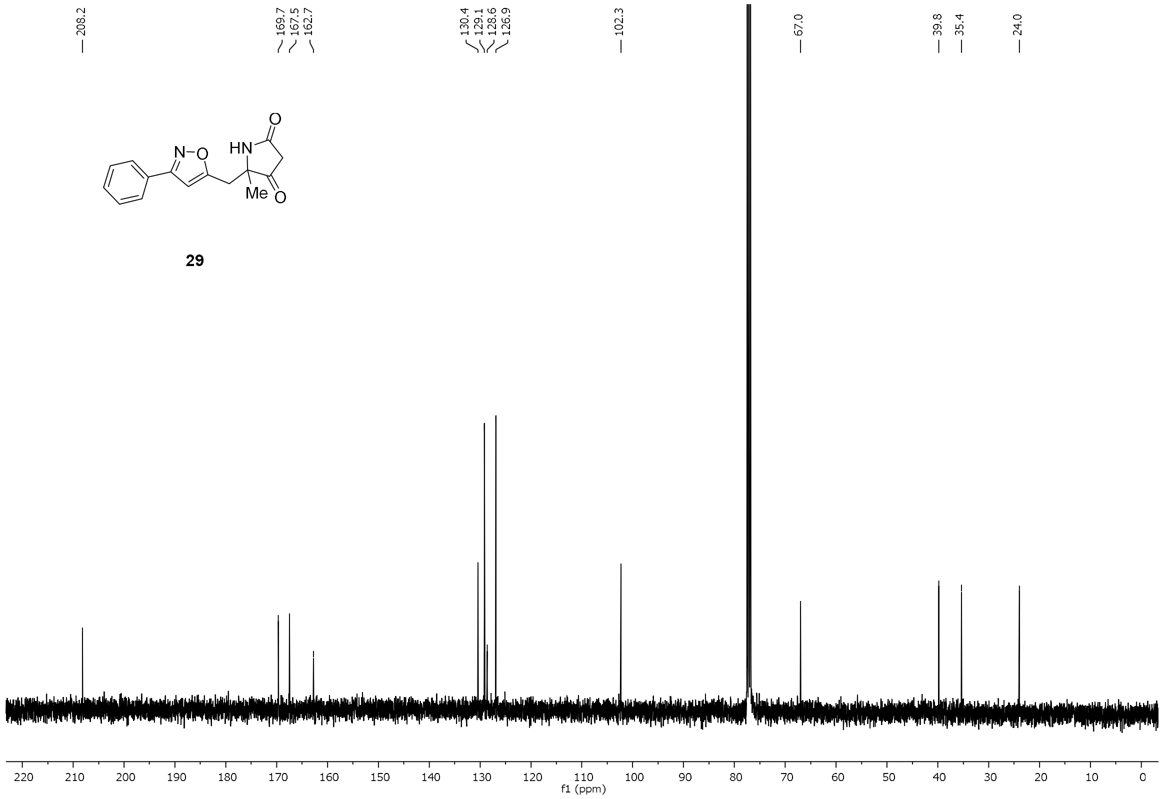
208.2
169.7 167.5 162.7
130.4 129.1 128.6 126.9
102.3
67.0
39.8
35.4
24.0
HN
N–O
O
Me
29
f1 (ppm)

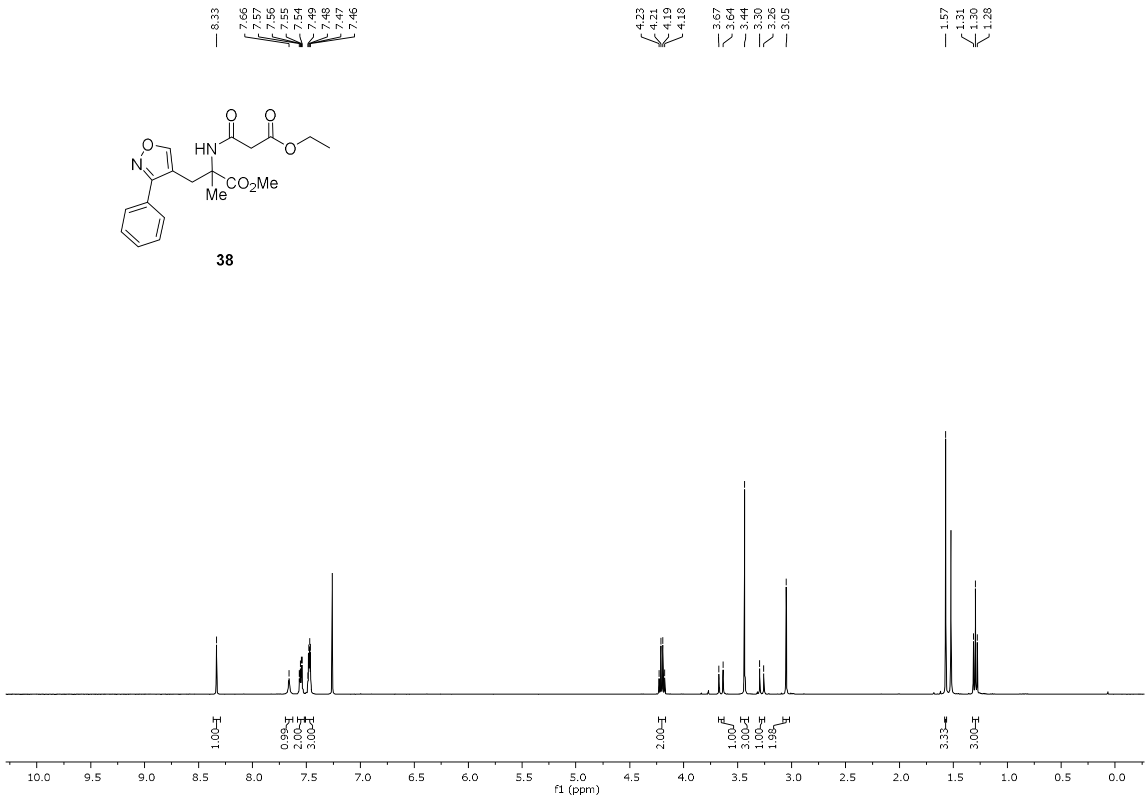
8.33
7.66 7.57 7.56 7.55 7.54 7.49 7.48 7.47 7.46
4.23 4.21 4.19 4.18
3.67 3.64 3.44 3.30 3.26 3.05
1.57 1.31 1.30 1.28
HN
CO2Me
Me
1.00
0.99 2.00 3.00
2.00
1.00 3.00 1.00 1.98
3.33
3.00
f1 (ppm)

38

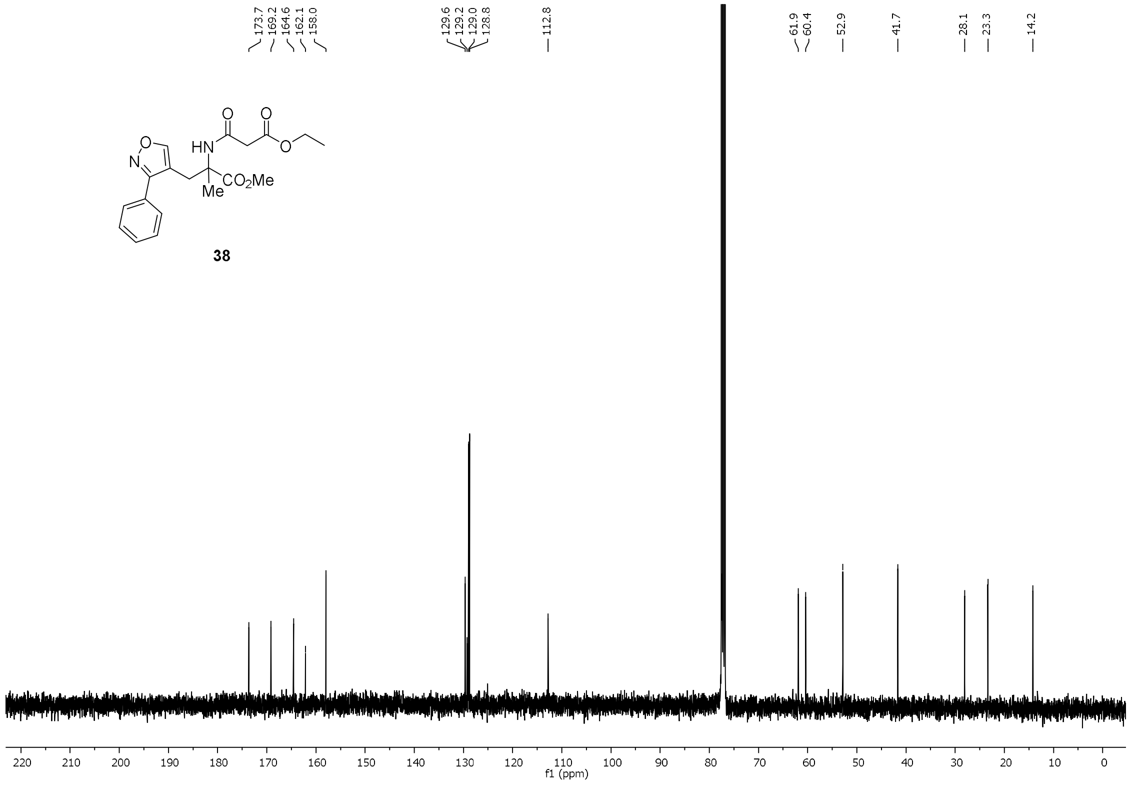
173.7 169.2 164.6 162.1 158.0
129.6 129.2 129.0 128.8
112.8
61.9 60.4
52.9
41.7
28.1
23.3
14.2
HN
CO2Me
Me
f1 (ppm)

38

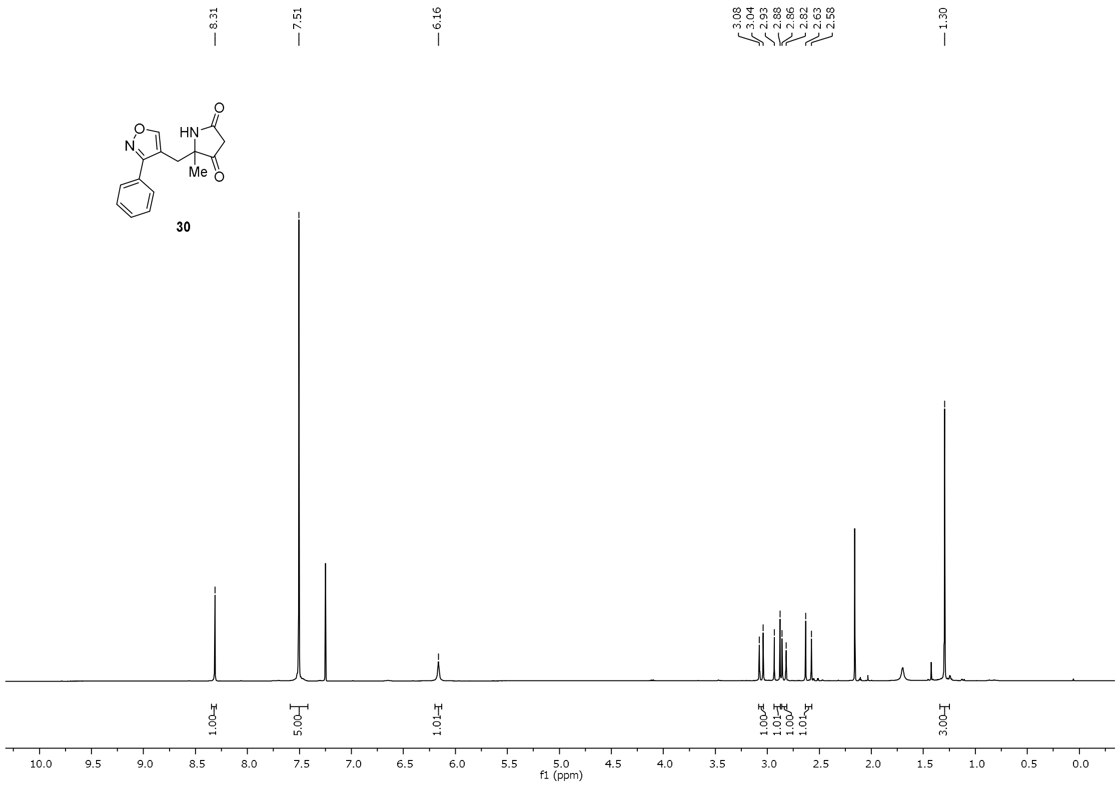
8.31
7.51
6.16
3.08
3.04
2.93
2.88
2.86
2.82
2.63
2.58
1.30
HN
O
N
Me
30
1.00
5.00
1.01
1.00
1.01
1.00
1.01
3.00
f1 (ppm)

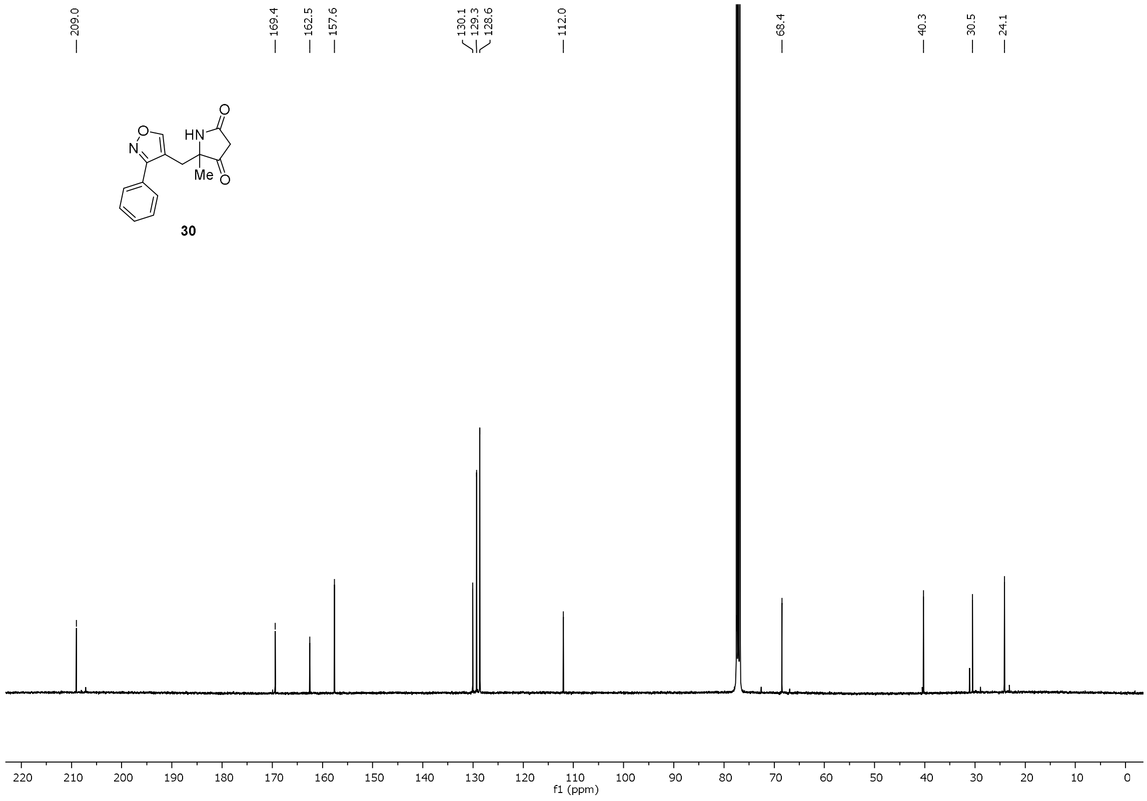
209.0
169.4
162.5
157.6
130.1
129.3
128.6
112.0
68.4
40.3
30.5
24.1
HN
O
N
Me
30
f1 (ppm)

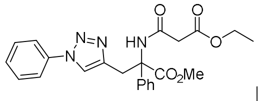

39

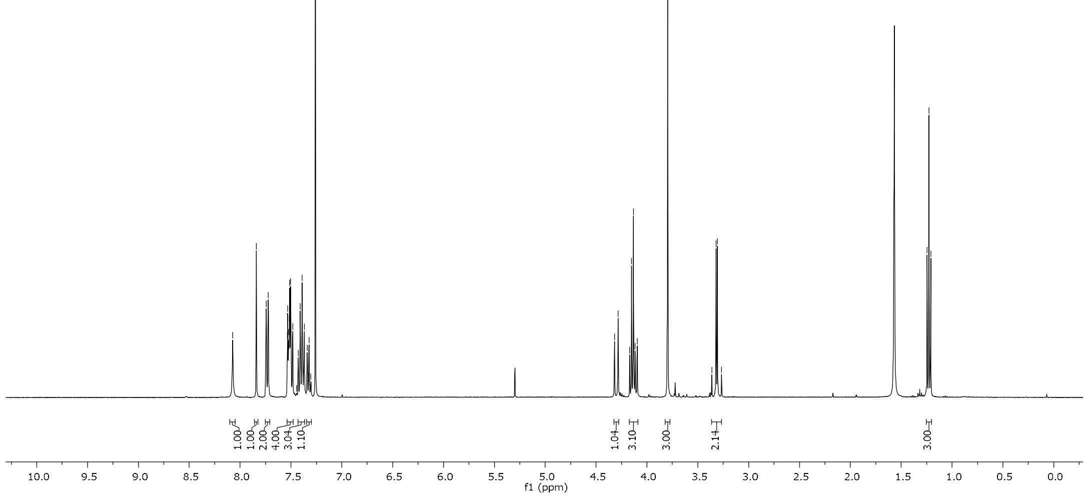
8.07 7.84 7.75 7.74 7.72 7.53 7.53 7.52 7.52 7.51 7.50 7.48 7.44 7.43 7.41 7.39 7.37 7.34 7.33 7.32 7.30
4.32 4.28 4.17 4.15 4.13 4.12 4.10 3.80
3.36 3.32 3.31 3.27
1.25 1.23 1.21
1.00 1.00 2.00 4.00 3.04 1.10
1.04 3.10 3.00 2.14 3.00
f1 (ppm)

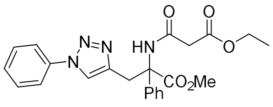

39

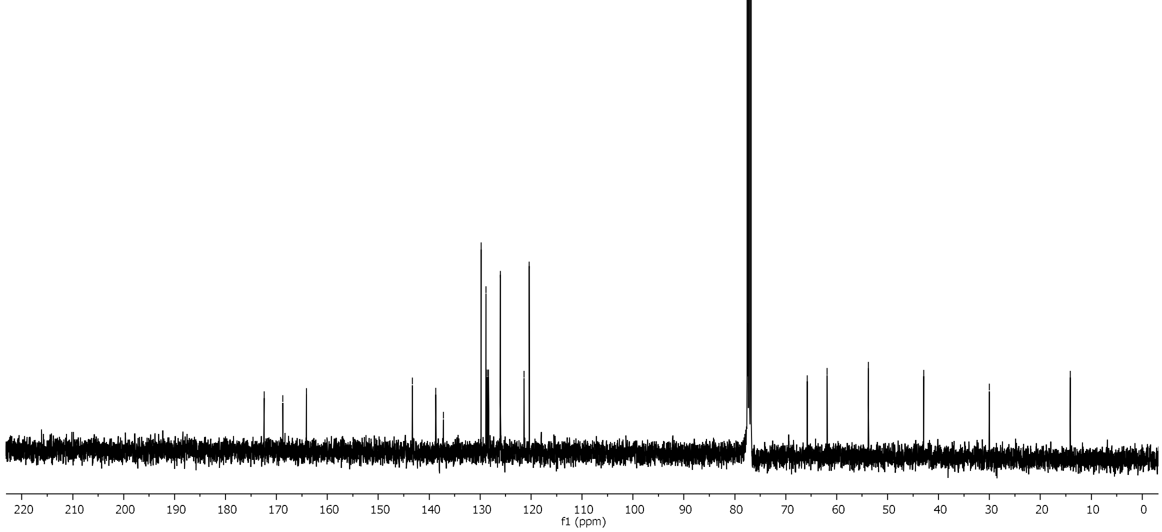
172.4 168.7 164.1
143.3 138.7 137.2
129.8 128.9 128.6 128.3 126.0 121.4 120.3
65.7 61.9 53.8 42.9 30.0 14.1
f1 (ppm)

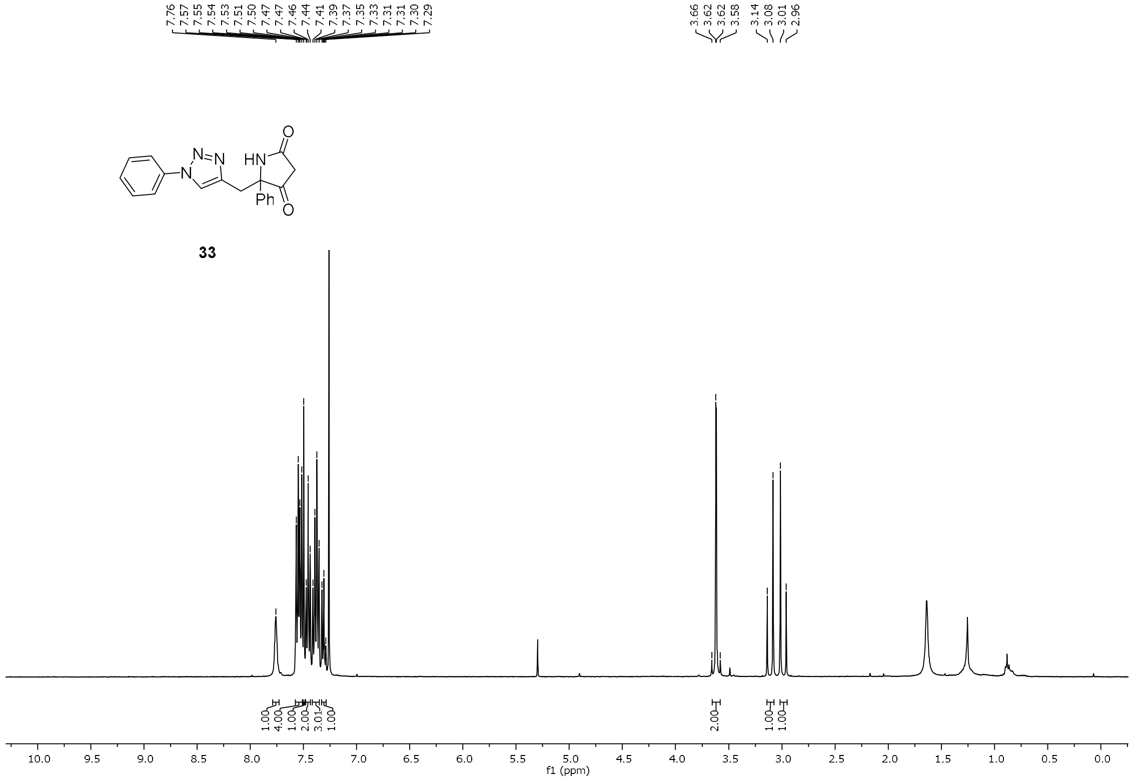
7.76 7.57 7.55 7.54 7.53 7.51 7.50 7.47 7.47 7.46 7.44 7.41 7.39 7.37 7.35 7.33 7.31 7.31 7.30 7.29
3.66 3.62 3.62 3.58 3.14 3.08 3.01 2.96
HN
O
N
N=N
Ph
33
1.00 4.00 1.00 2.00 3.01 1.00
2.00
1.00 1.00
10.0 9.5 9.0 8.5 8.0 7.5 7.0 6.5 6.0 5.5 5.0 4.5 4.0 3.5 3.0 2.5 2.0 1.5 1.0 0.5 0.0
f1 (ppm)

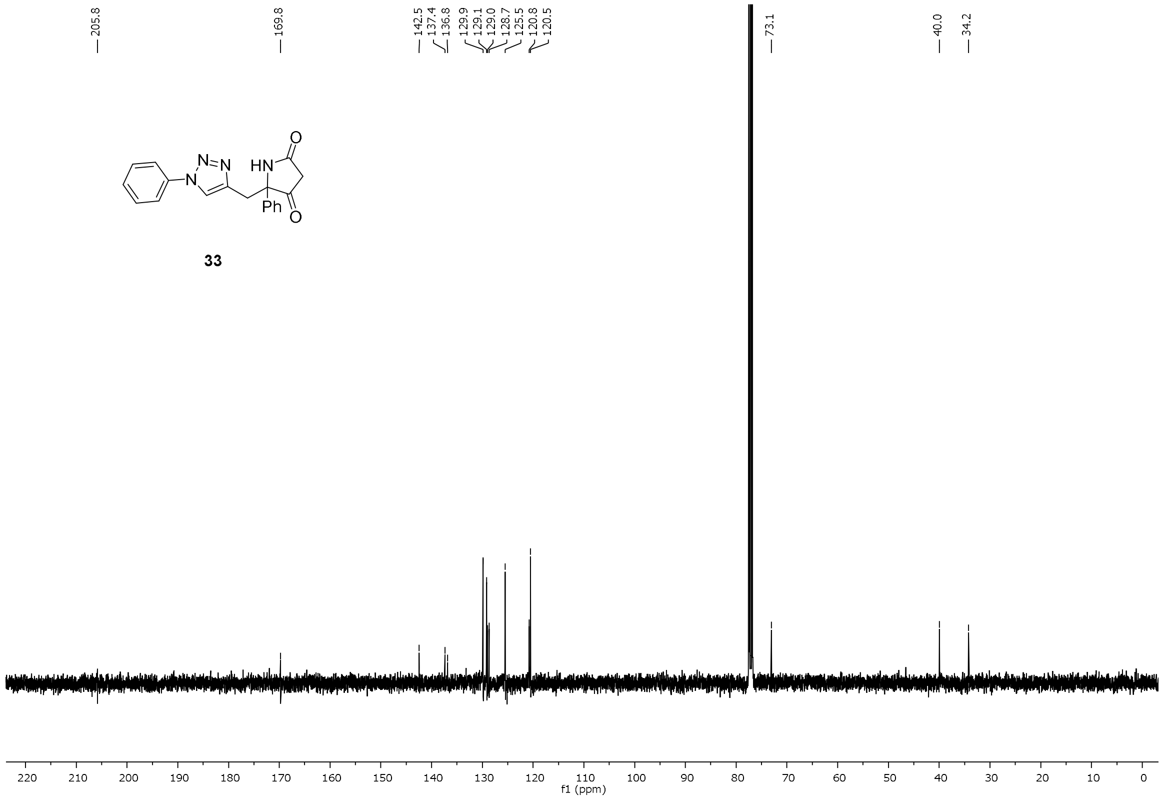
205.8
169.8
142.5 137.4 136.8 129.9 129.1 129.0 128.7 125.5 120.8 120.5
73.1
40.0 34.2
HN
O
N
N=N
Ph
33
220 210 200 190 180 170 160 150 140 130 120 110 100 90 80 70 60 50 40 30 20 10 0
f1 (ppm)

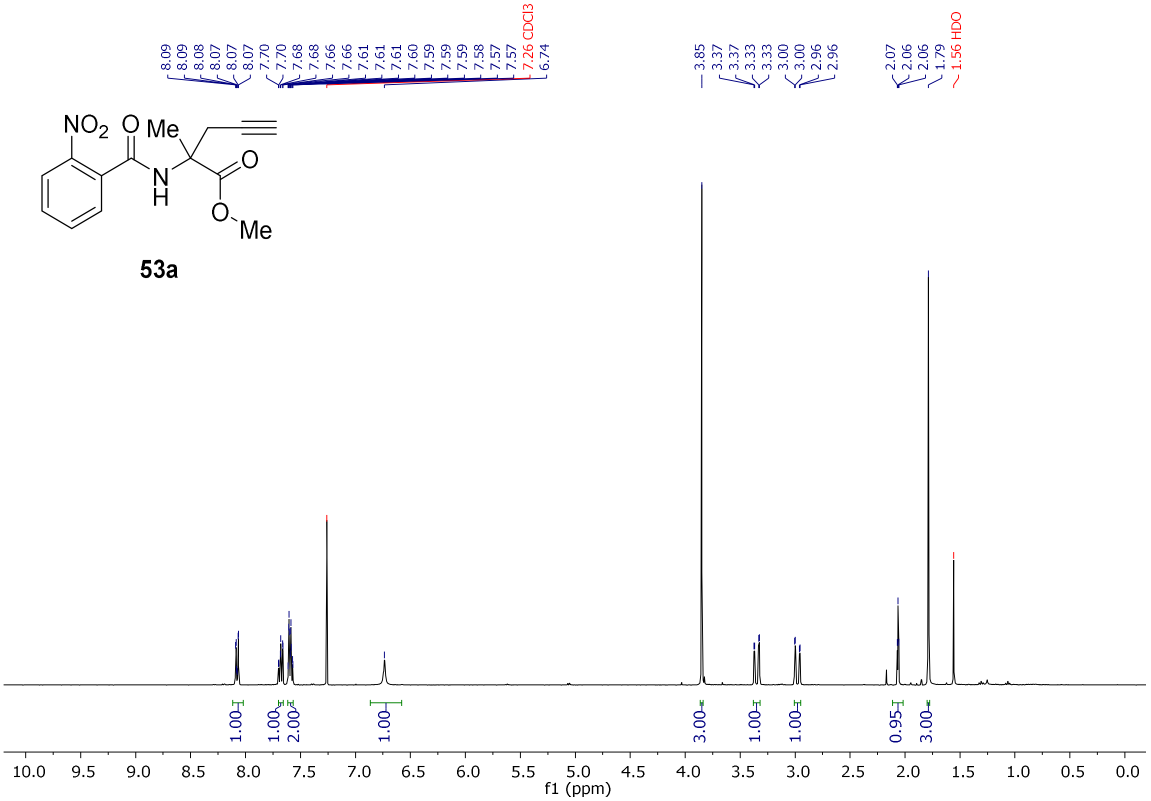
8.09
8.09
8.08
8.07
8.07
8.07
7.70
7.70
7.68
7.68
7.66
7.66
7.61
7.61
7.61
7.60
7.59
7.59
7.59
7.58
7.57
7.57
7.26 CDCl3
6.74
3.85
3.37
3.37
3.33
3.33
3.00
3.00
2.96
2.96
2.07
2.06
2.06
1.79
1.56 HDO
NO2
O
Me
N
H
O
O
Me
53a
1.00
1.00
2.00
1.00
3.00
1.00
1.00
0.95
3.00
10.0
9.5
9.0
8.5
8.0
7.5
7.0
6.5
6.0
5.5
5.0
4.5
4.0
3.5
3.0
2.5
2.0
1.5
1.0
0.5
0.0
f1 (ppm)

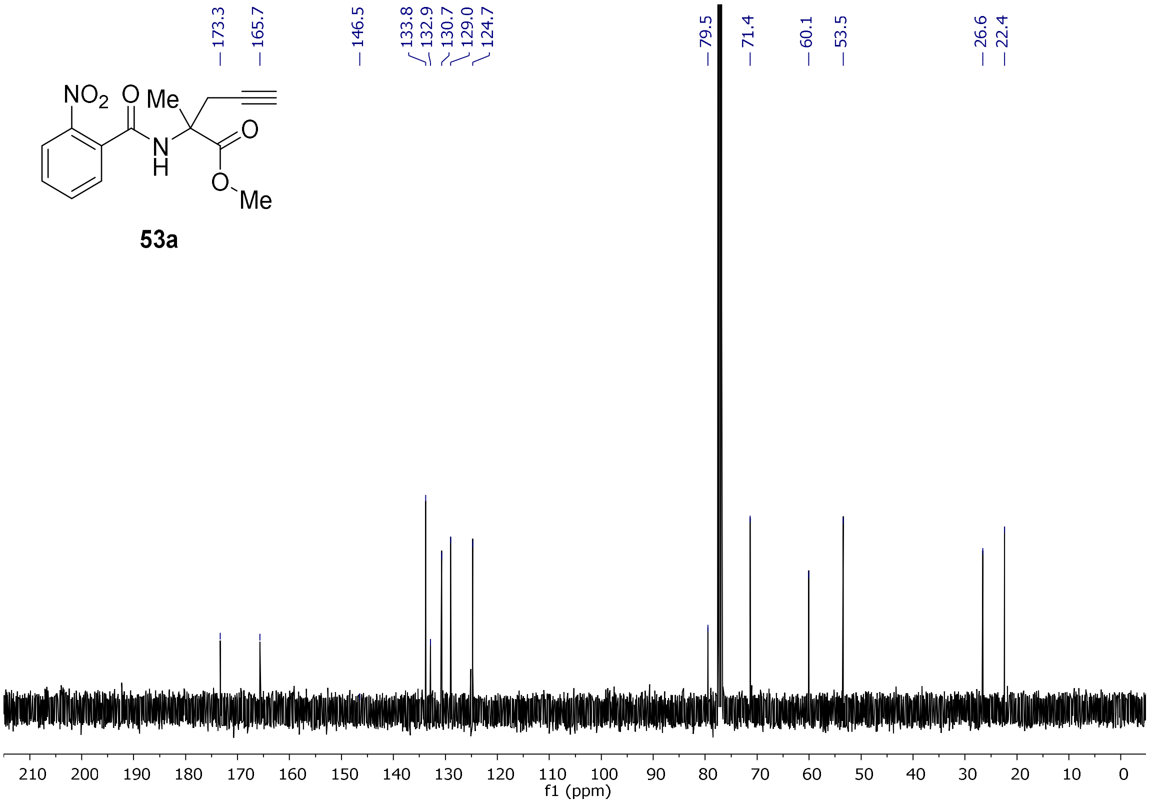
173.3
165.7
146.5
133.8
132.9
130.7
129.0
124.7
79.5
71.4
60.1
53.5
26.6
22.4
NO2
O
Me
N
H
O
O
Me
53a
210
200
190
180
170
160
150
140
130
120
110
100
90
80
70
60
50
40
30
20
10
0
f1 (ppm)

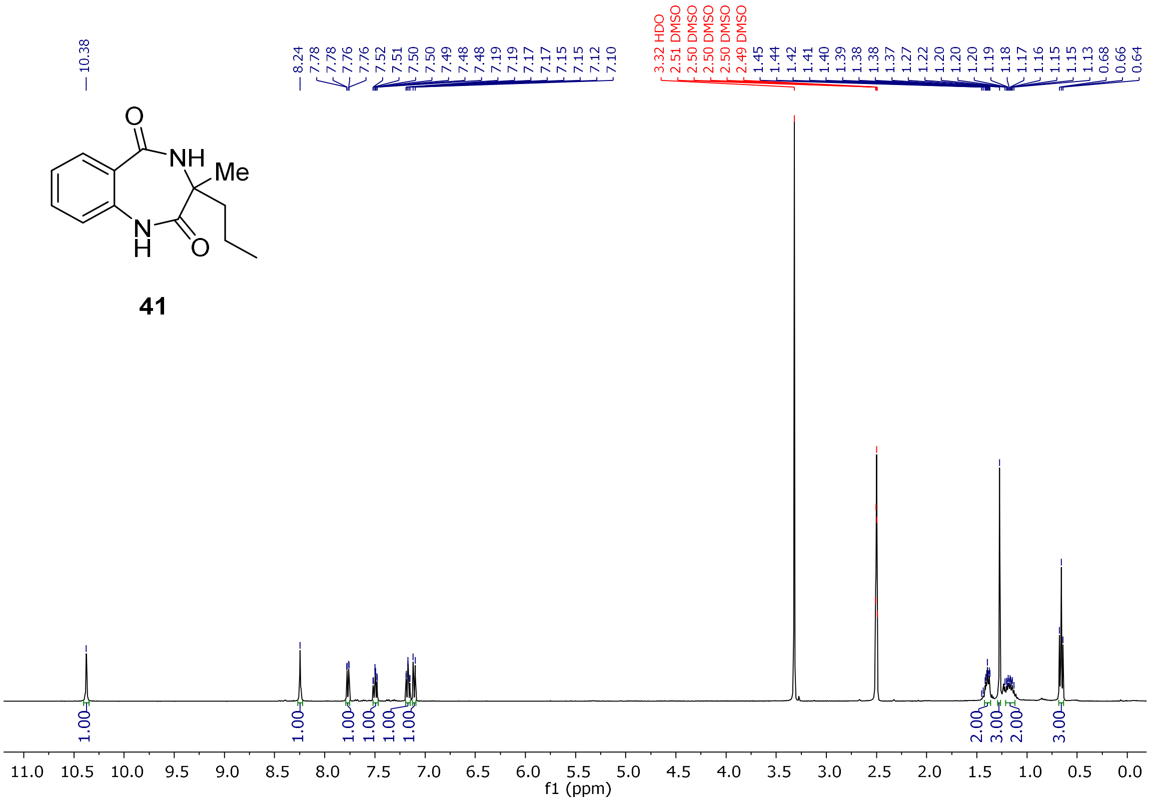
41
f1 (ppm)
1.00
1.00
1.00
1.00
1.00
1.00
2.00
3.00
2.00
3.00

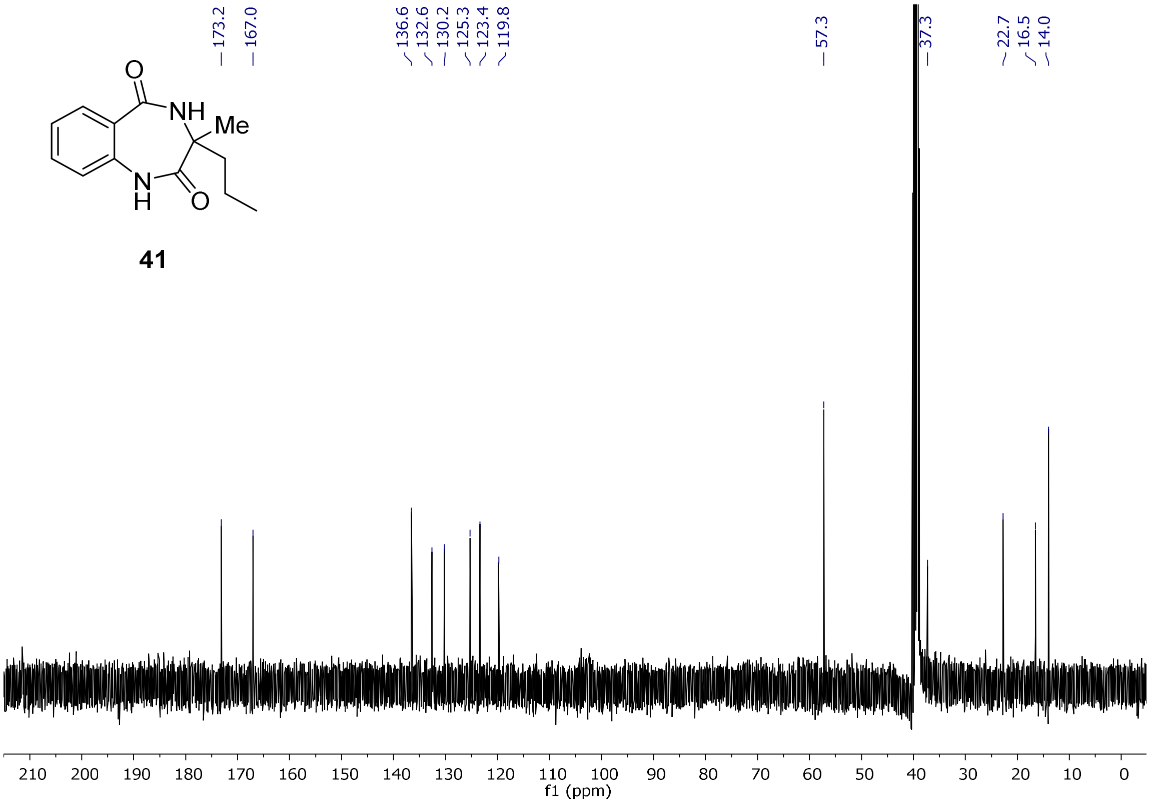
41
f1 (ppm)

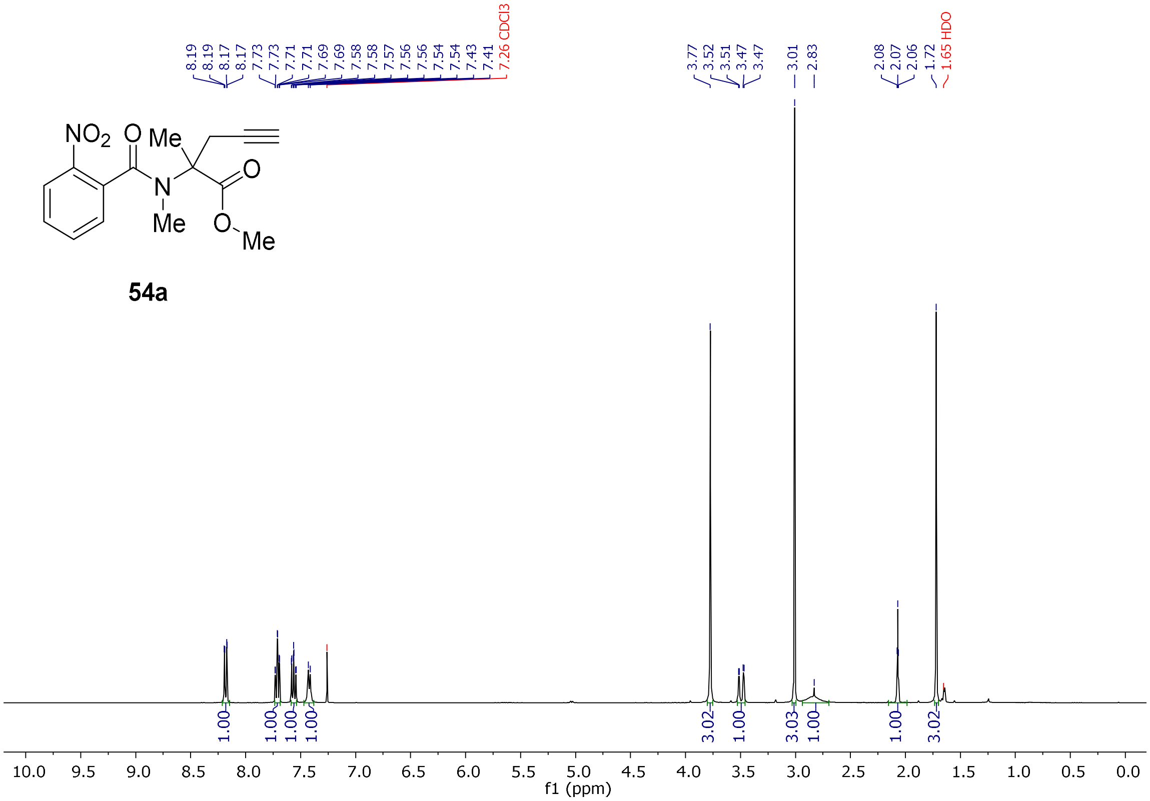

8.19
8.19
8.17
8.17
7.73
7.73
7.71
7.71
7.69
7.69
7.58
7.58
7.57
7.56
7.56
7.54
7.54
7.43
7.41
7.26 CDCl3
3.77
3.52
3.51
3.47
3.47
3.01
2.83
2.08
2.07
2.06
1.72
1.65 HDO
1.00
1.00
1.00
1.00
3.02
1.00
3.03
1.00
1.00
3.02
10.0 9.5 9.0 8.5 8.0 7.5 7.0 6.5 6.0 5.5 5.0 4.5 4.0 3.5 3.0 2.5 2.0 1.5 1.0 0.5 0.0
f1 (ppm)

54a

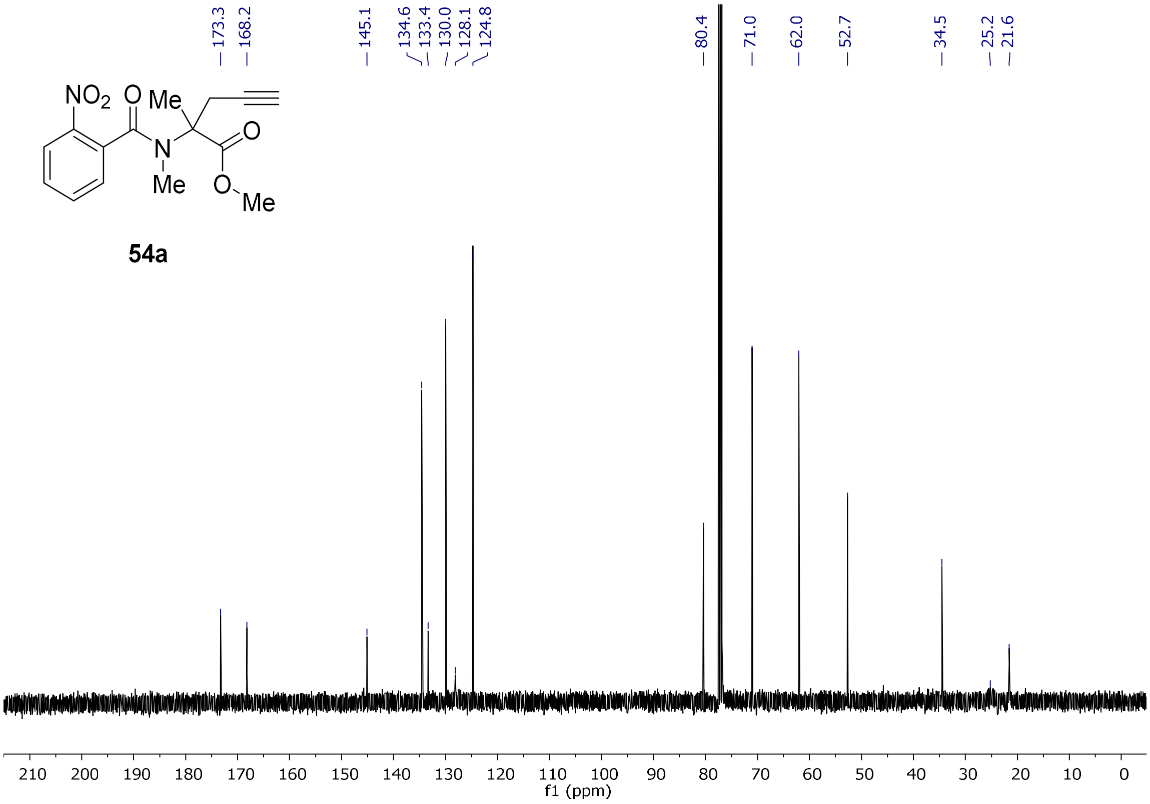

173.3
168.2
145.1
134.6
133.4
130.0
128.1
124.8
80.4
71.0
62.0
52.7
34.5
25.2
21.6
210 200 190 180 170 160 150 140 130 120 110 100 90 80 70 60 50 40 30 20 10 0
f1 (ppm)

54a

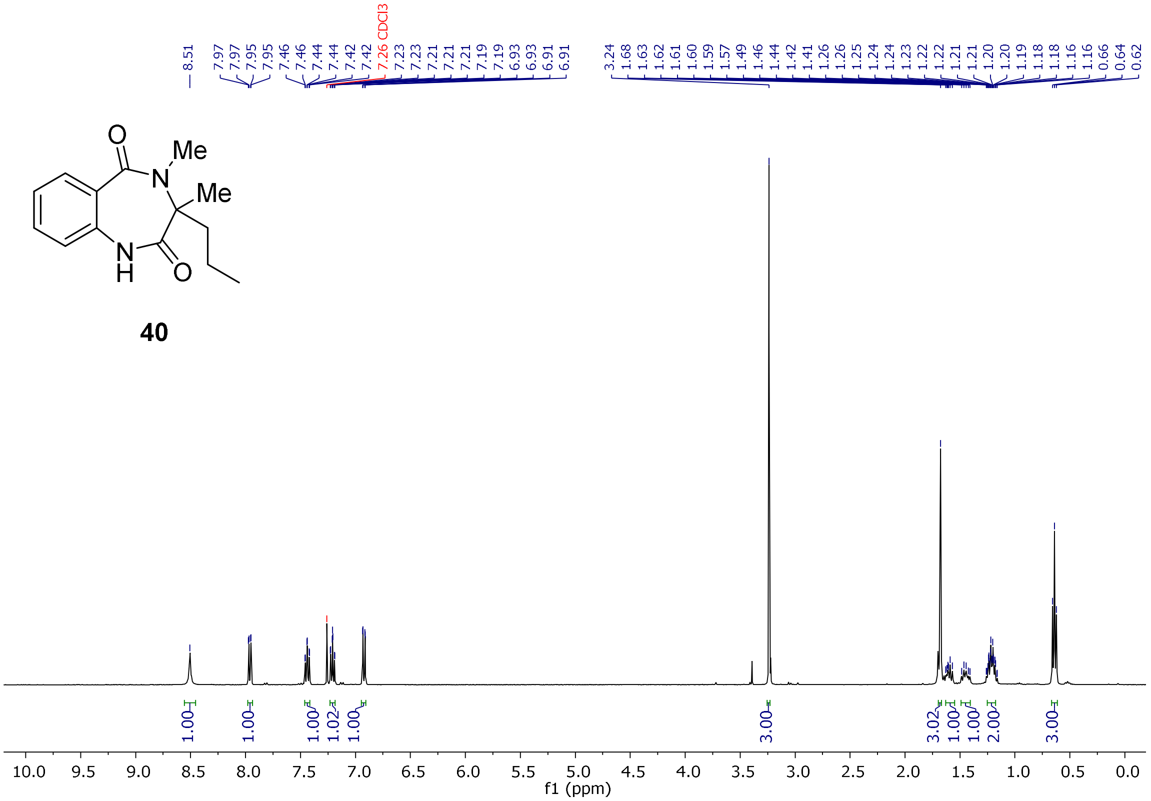
8.51
7.97 7.97 7.95 7.95 7.46 7.46 7.44 7.44 7.42 7.42
7.26 CDCl3
7.23 7.23 7.21 7.21 7.21 7.19 6.93 6.93 6.91 6.91
3.24 1.68 1.63 1.62 1.61 1.60 1.59 1.57 1.49 1.46 1.44 1.42 1.41 1.26 1.26 1.25 1.24 1.24 1.23 1.22 1.22 1.21 1.21 1.20 1.20 1.19 1.18 1.18 1.16 1.16 0.66 0.64 0.62
O
Me
N
Me
N
H
O
1.00 1.00 1.00 1.02 1.00 3.00 3.02 1.00 1.00 2.00 3.00
10.0 9.5 9.0 8.5 8.0 7.5 7.0 6.5 6.0 5.5 5.0 4.5 4.0 3.5 3.0 2.5 2.0 1.5 1.0 0.5 0.0
f1 (ppm)

40

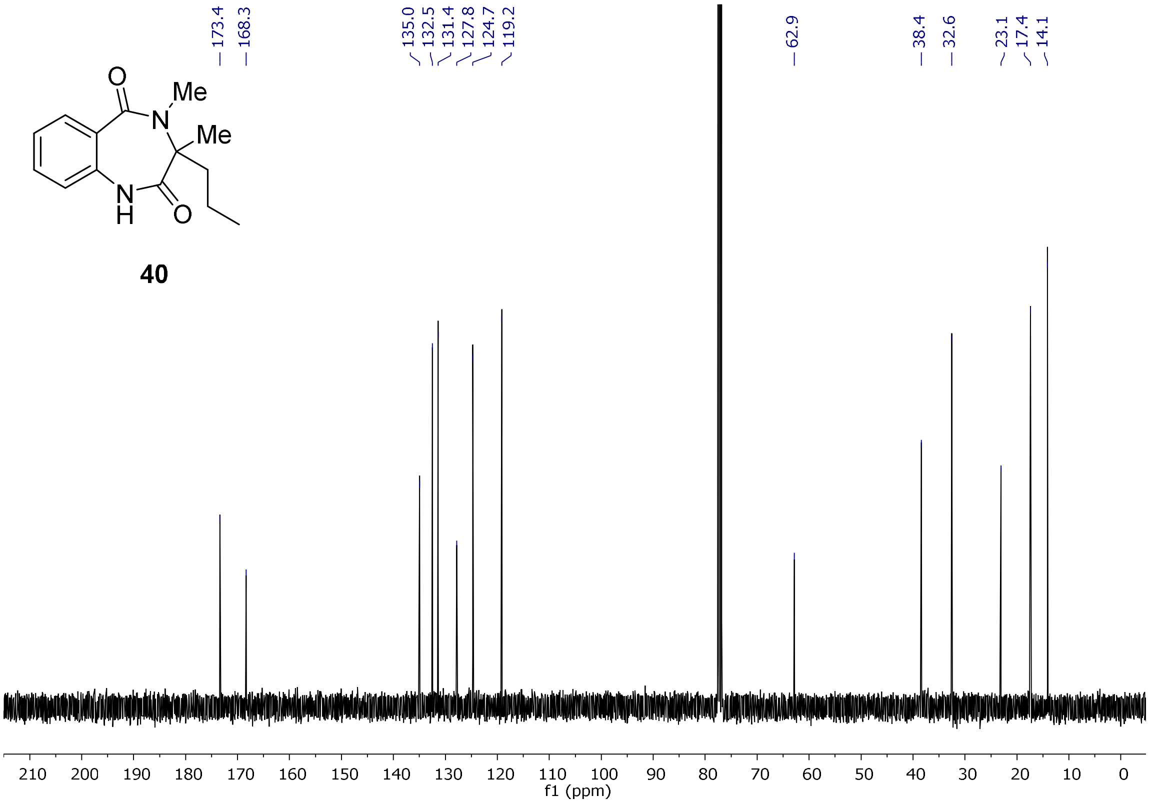
173.4 168.3
135.0 132.5 131.4 127.8 124.7 119.2
62.9
38.4 32.6
23.1 17.4 14.1
O
Me
N
Me
N
H
O
210 200 190 180 170 160 150 140 130 120 110 100 90 80 70 60 50 40 30 20 10 0
f1 (ppm)

40

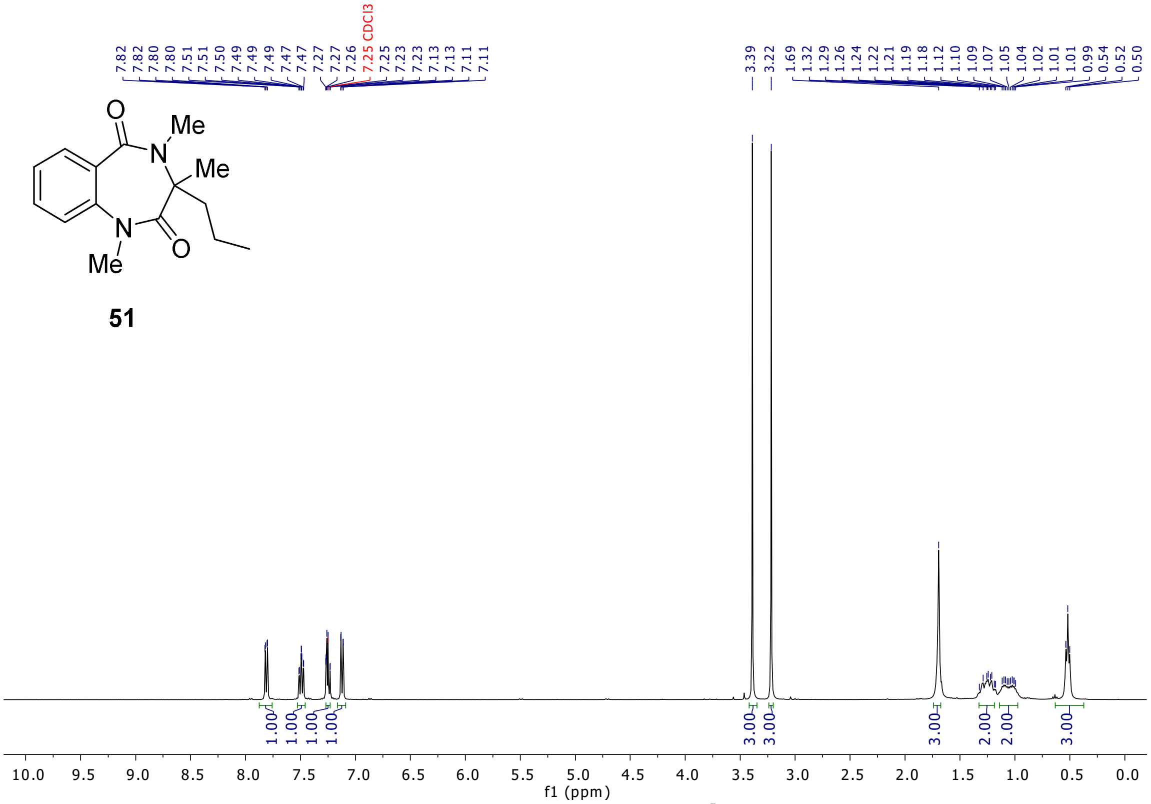
7.82
7.82
7.80
7.80
7.51
7.51
7.50
7.49
7.49
7.49
7.47
7.47
7.27
7.27
7.26
7.25 CDCl3
7.25
7.23
7.23
7.13
7.13
7.11
7.11
3.39
3.22
1.69
1.32
1.29
1.26
1.24
1.22
1.21
1.19
1.18
1.12
1.10
1.09
1.07
1.05
1.04
1.02
1.01
1.01
0.99
0.54
0.52
0.50
O
Me
N
Me
N
O
Me
51
1.00
1.00
1.00
1.00
3.00
3.00
3.00
2.00
2.00
3.00
10.0
9.5
9.0
8.5
8.0
7.5
7.0
6.5
6.0
5.5
5.0
4.5
4.0
3.5
3.0
2.5
2.0
1.5
1.0
0.5
0.0
f1 (ppm)

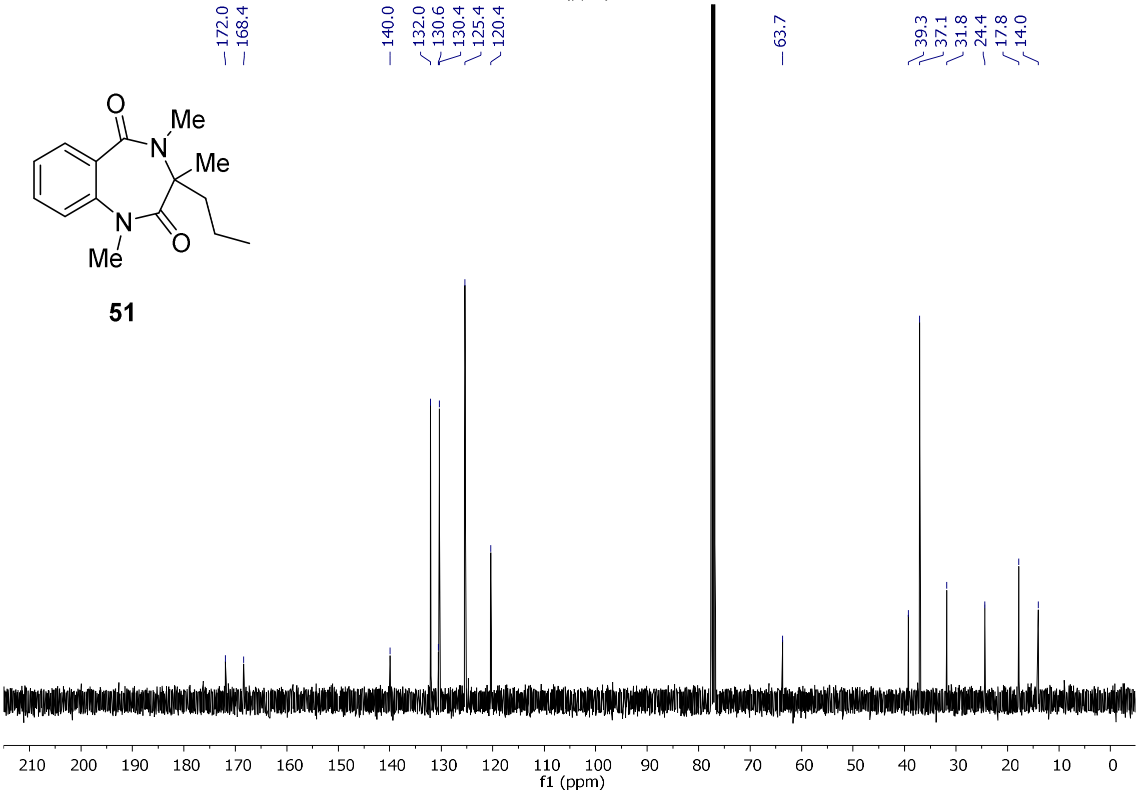
172.0
168.4
140.0
132.0
130.6
130.4
125.4
120.4
63.7
39.3
37.1
31.8
24.4
17.8
14.0
O
Me
N
Me
N
O
Me
51
210
200
190
180
170
160
150
140
130
120
110
100
90
80
70
60
50
40
30
20
10
0
f1 (ppm)

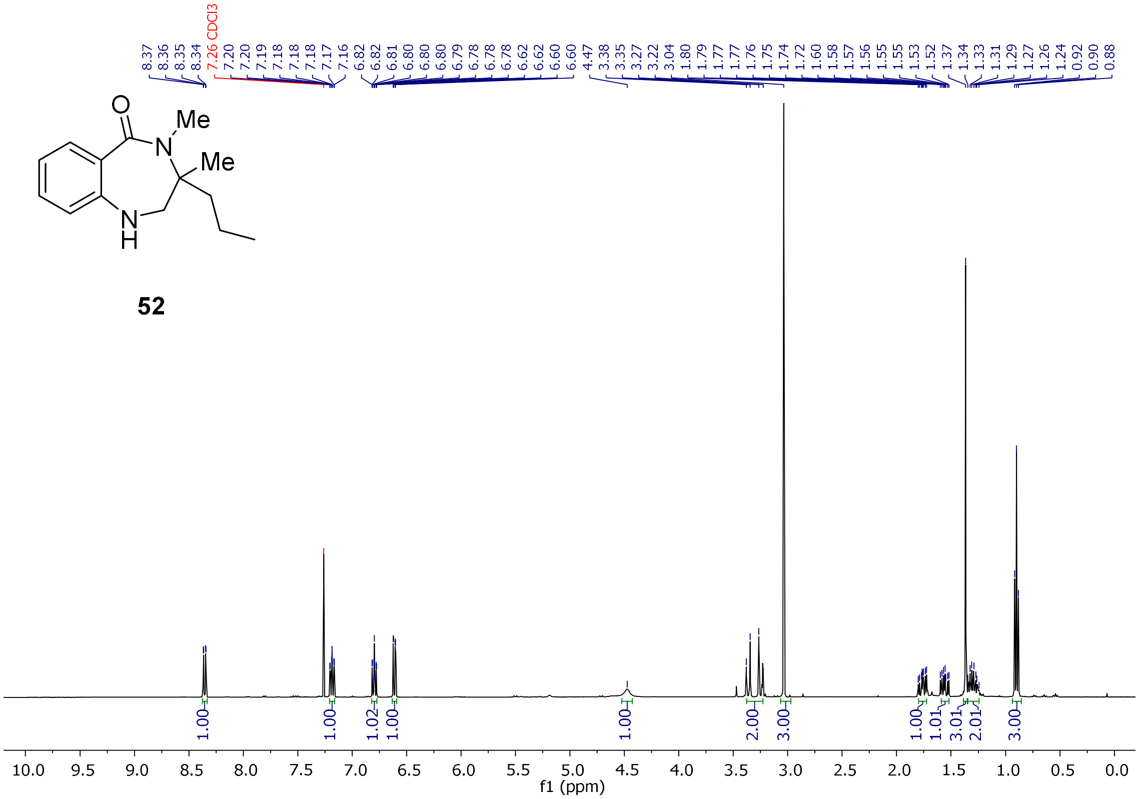

Me
Me
N
N
H
O
8.37 8.36 8.35 8.34 7.26 CDCl3 7.20 7.20 7.19 7.18 7.18 7.18 7.17 7.16 6.82 6.82 6.81 6.80 6.80 6.80 6.79 6.78 6.78 6.62 6.62 6.60 6.60 4.47 3.38 3.35 3.27 3.22 3.04 1.80 1.79 1.77 1.77 1.76 1.75 1.74 1.72 1.60 1.58 1.57 1.56 1.55 1.55 1.53 1.52 1.37 1.34 1.33 1.31 1.29 1.27 1.26 1.24 0.92 0.90 0.88
1.00 1.00 1.02 1.00 1.00 2.00 3.00 1.00 1.01 3.01 2.01 3.00
10.0 9.5 9.0 8.5 8.0 7.5 7.0 6.5 6.0 5.5 5.0 4.5 4.0 3.5 3.0 2.5 2.0 1.5 1.0 0.5 0.0
f1 (ppm)

52

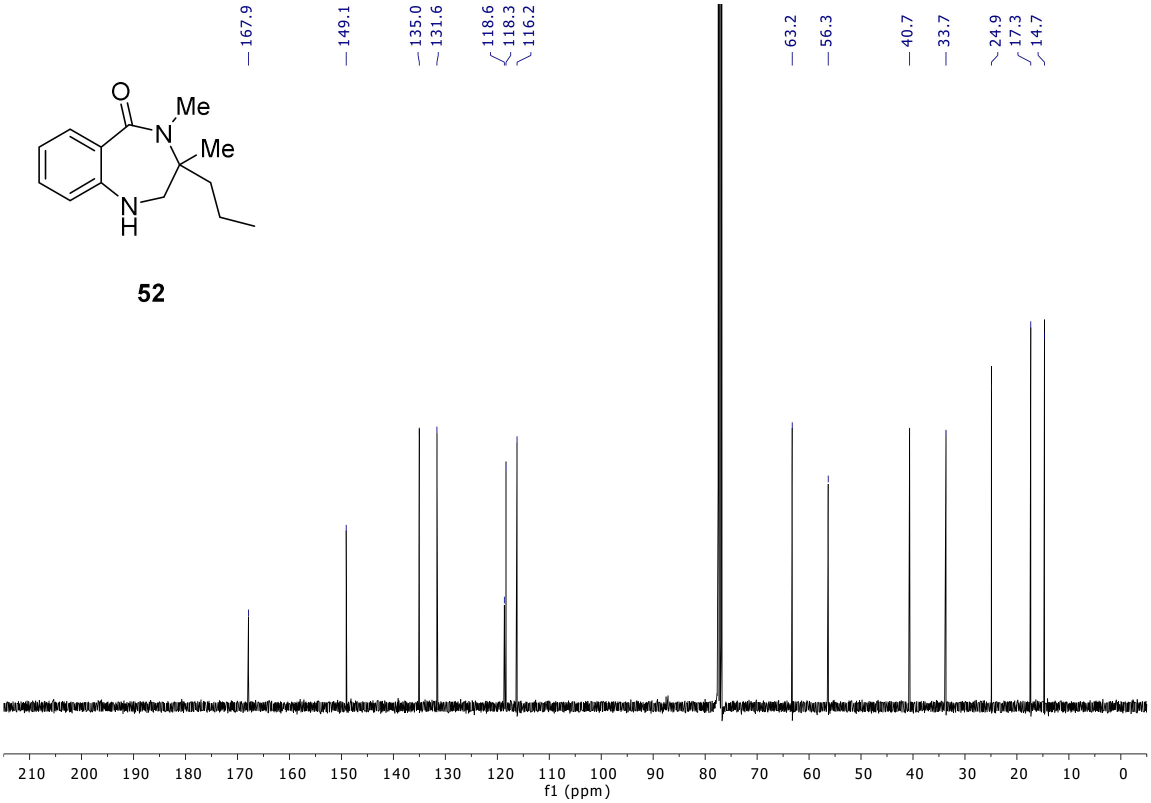

Me
Me
N
N
H
O
167.9 149.1 135.0 131.6 118.6 118.3 116.2 63.2 56.3 40.7 33.7 24.9 17.3 14.7
210 200 190 180 170 160 150 140 130 120 110 100 90 80 70 60 50 40 30 20 10 0
f1 (ppm)

52

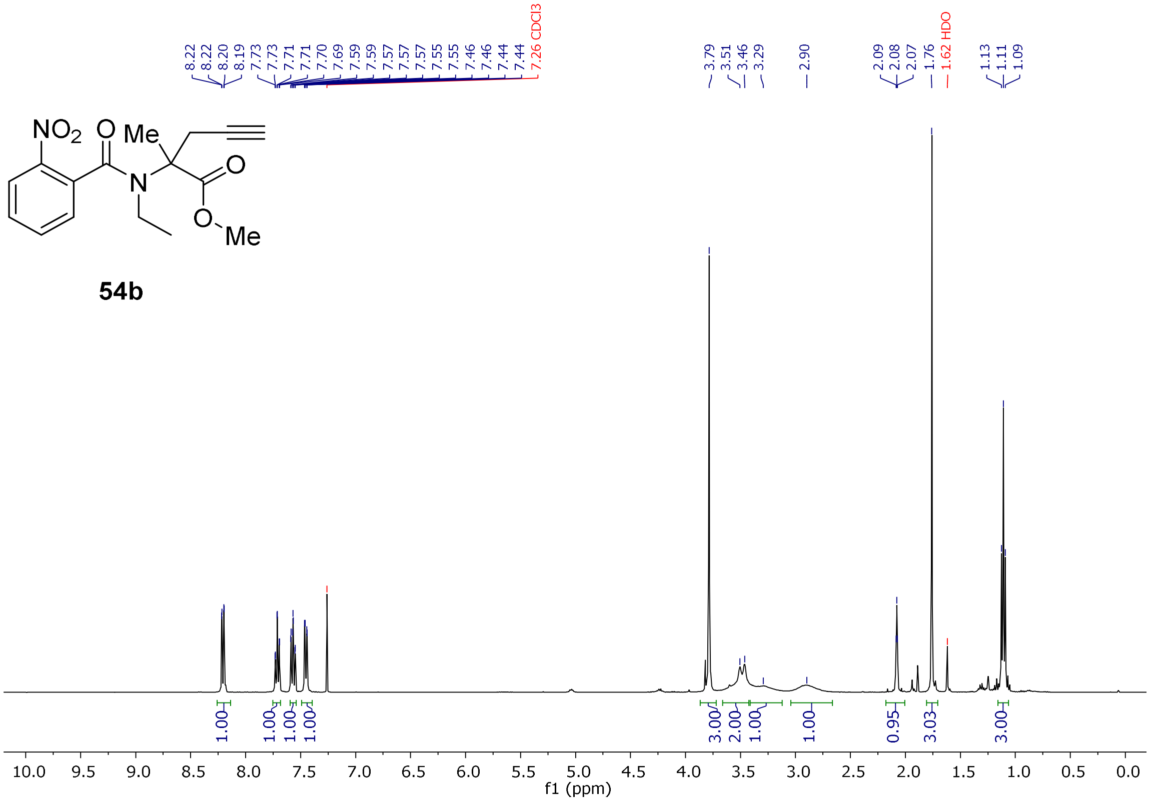
NO2
Me
N
O
Me
8.22
8.22
8.20
8.19
7.73
7.73
7.71
7.71
7.70
7.69
7.59
7.59
7.57
7.57
7.57
7.55
7.55
7.46
7.46
7.44
7.44
7.26 CDCl3
3.79
3.51
3.46
3.29
2.90
2.09
2.08
2.07
1.76
1.62 HDO
1.13
1.11
1.09
1.00
1.00
1.00
1.00
3.00
2.00
1.00
1.00
0.95
3.03
3.00
f1 (ppm)

54b

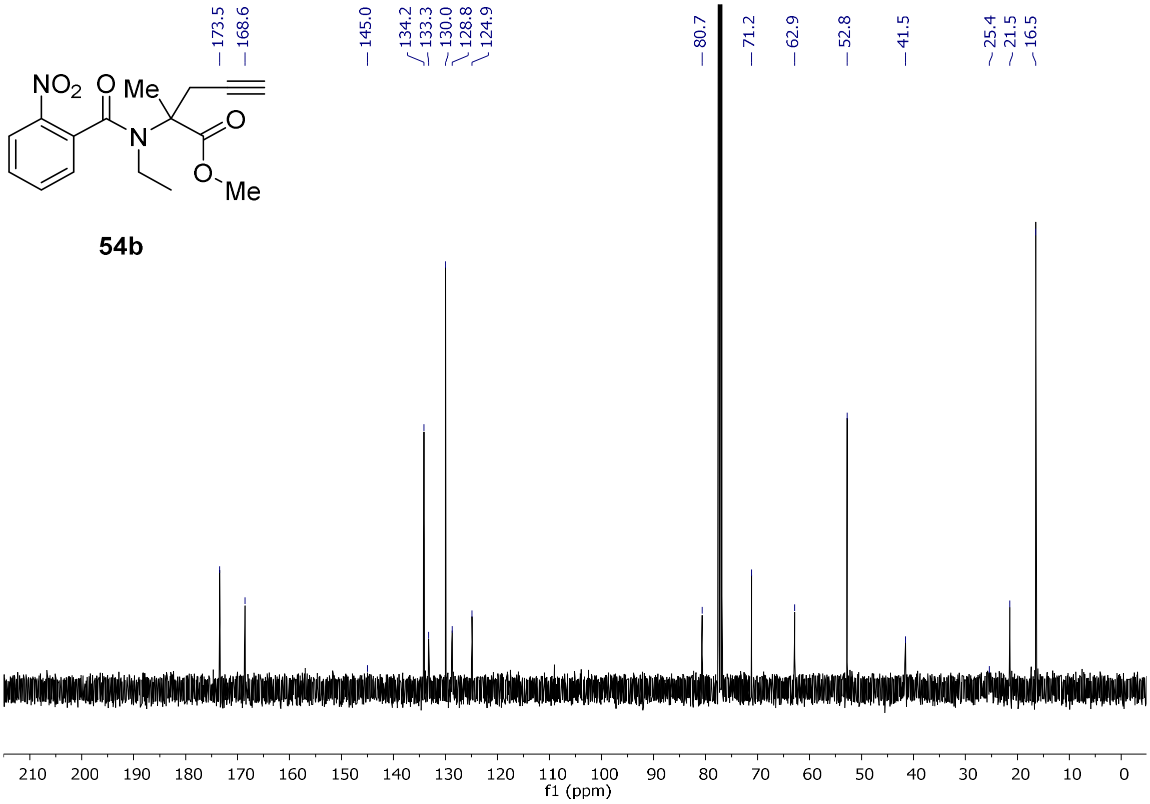
NO2
Me
N
O
Me
173.5
168.6
145.0
134.2
133.3
130.0
128.8
124.9
80.7
71.2
62.9
52.8
41.5
25.4
21.5
16.5
f1 (ppm)

54b

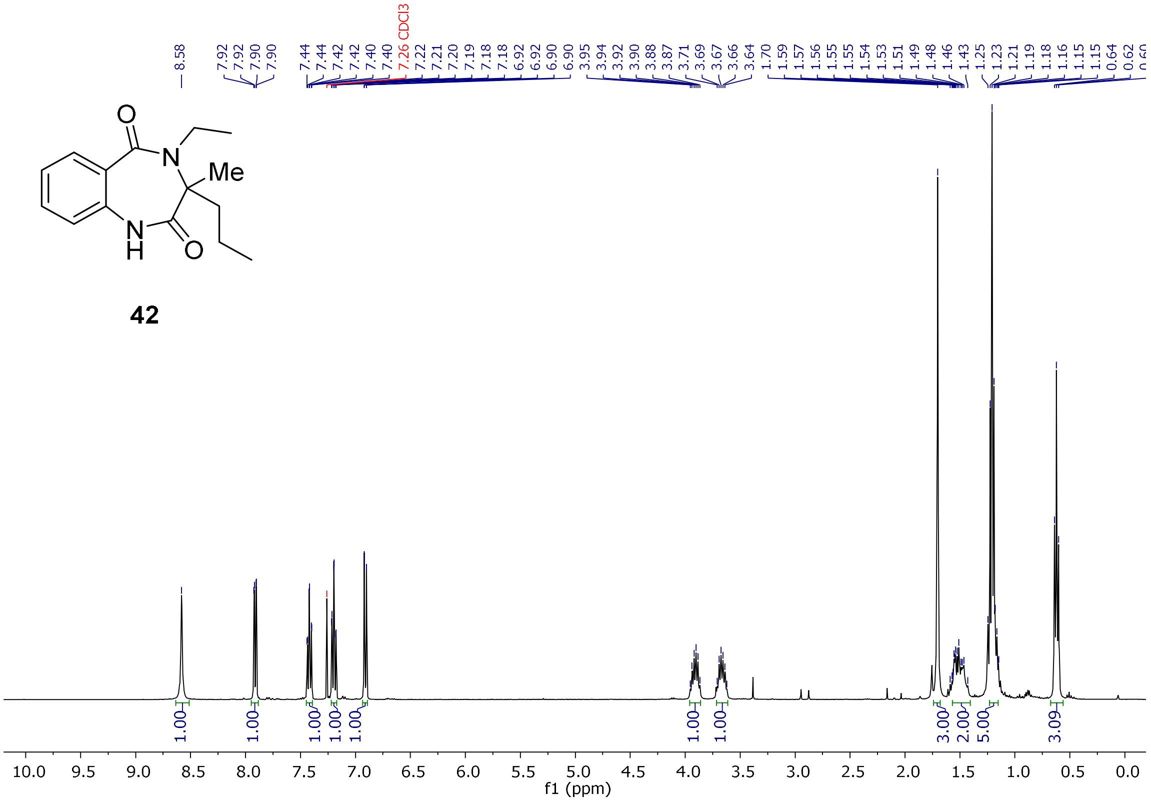
8.58
7.92 7.92 7.90 7.90
7.44 7.44 7.42 7.42 7.40 7.40
7.26 CDCl3
7.22 7.21 7.20 7.19 7.18 7.18 6.92 6.92 6.90 6.90
3.95 3.94 3.92 3.90 3.88 3.87 3.71 3.69 3.67 3.66 3.64
1.70 1.59 1.57 1.56 1.55 1.55 1.54 1.53 1.51 1.49 1.48 1.46 1.43
1.25 1.23 1.21 1.19 1.18 1.16 1.15 1.15 0.64 0.62
1.00 1.00 1.00 1.00 1.00 1.00 1.00 3.00 2.00 5.00 3.09
Me
f1 (ppm)

42

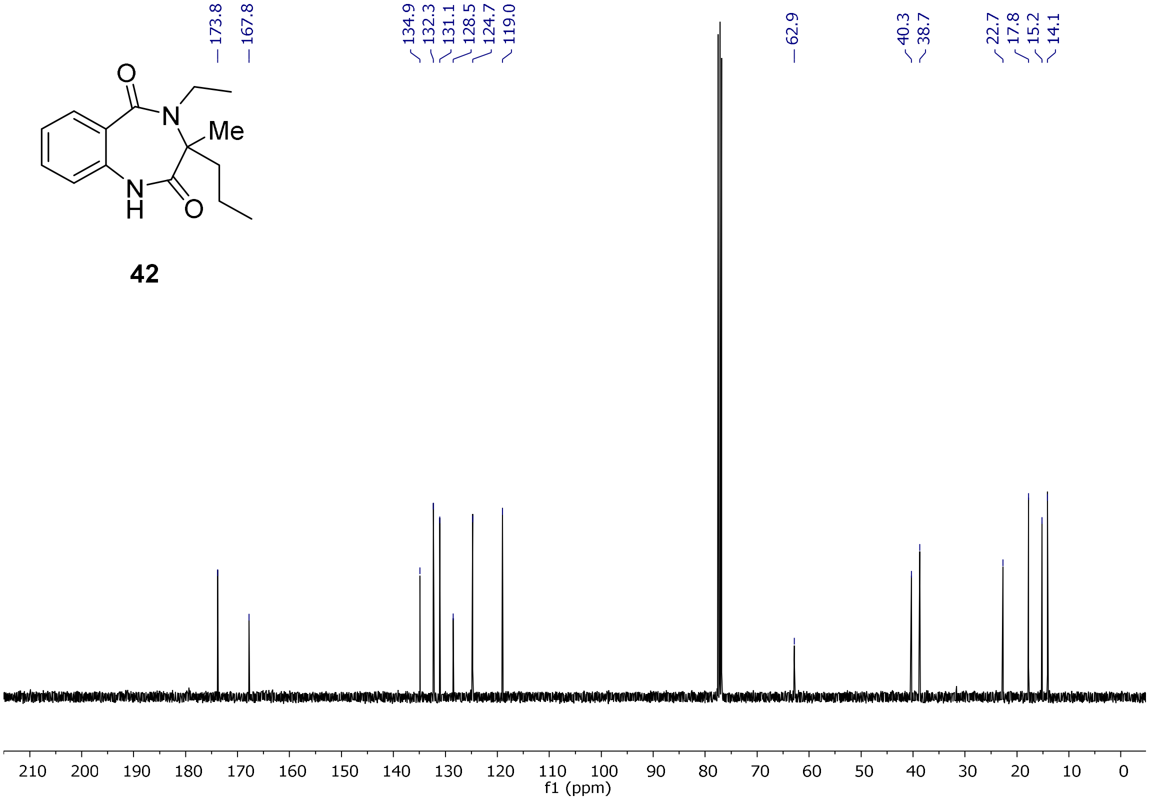
173.8
167.8
134.9 132.3 131.1 128.5 124.7 119.0
62.9
40.3 38.7
22.7 17.8 15.2 14.1
Me
f1 (ppm)

42

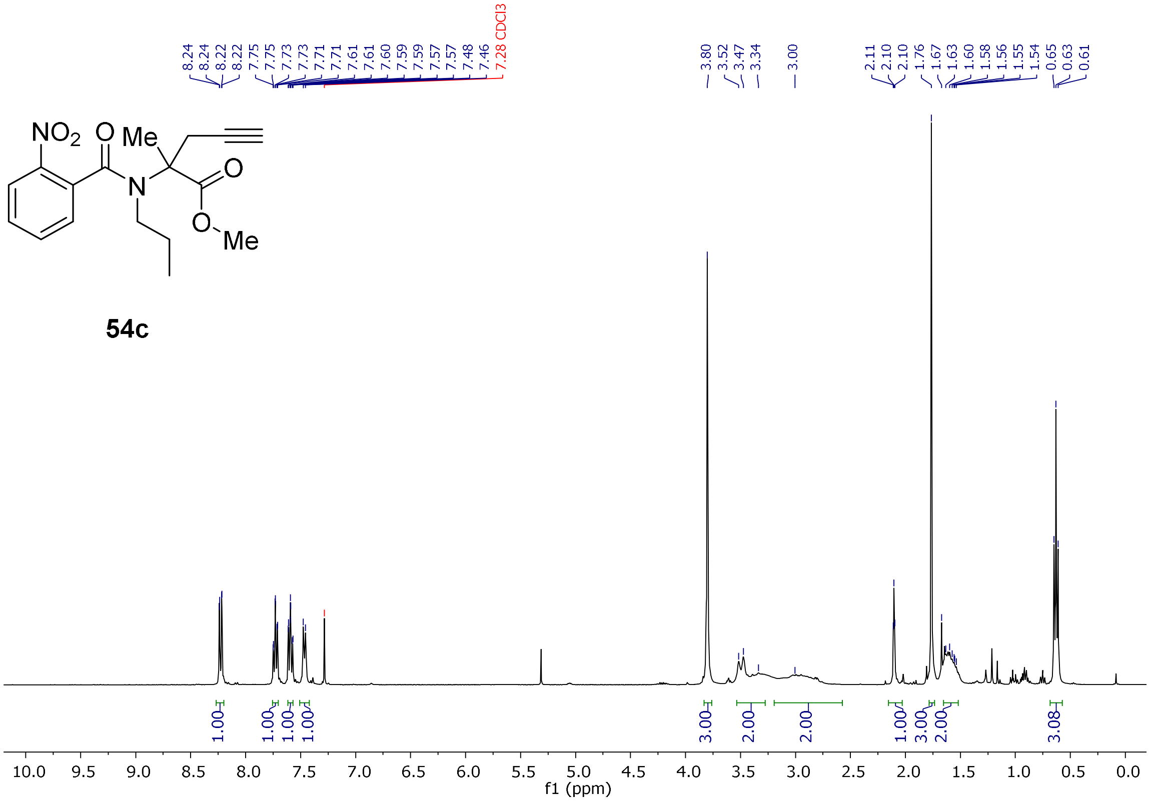
NO2 O Me
N
O
O
Me
54c
8.24 8.24 8.22 8.22 7.75 7.75 7.73 7.73 7.71 7.71 7.61 7.61 7.60 7.59 7.59 7.57 7.57 7.48 7.46 7.28 CDCl3
3.80 3.52 3.47 3.34 3.00
2.11 2.10 2.10 1.76 1.67 1.63 1.60 1.58 1.56 1.55 1.54 0.65 0.63 0.61
1.00 1.00 1.00 1.00 3.00 2.00 2.00 1.00 3.00 2.00 3.08
10.0 9.5 9.0 8.5 8.0 7.5 7.0 6.5 6.0 5.5 5.0 4.5 4.0 3.5 3.0 2.5 2.0 1.5 1.0 0.5 0.0
f1 (ppm)

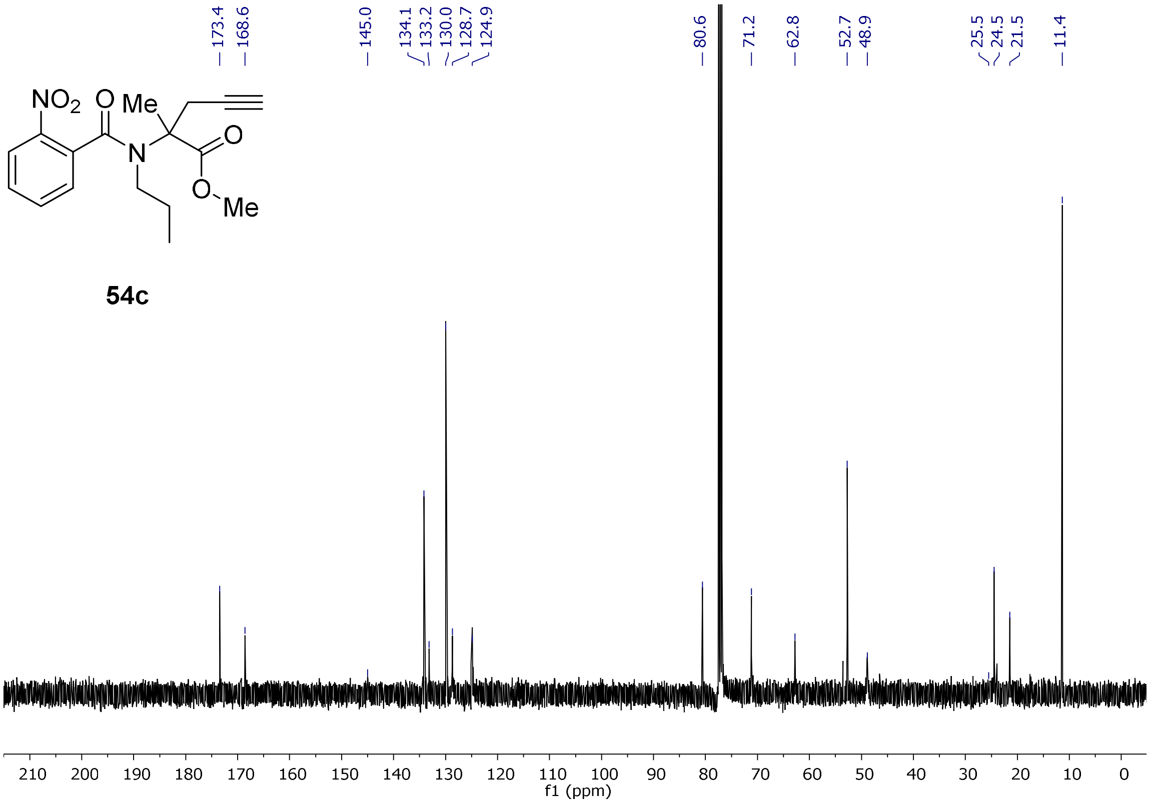
NO2 O Me
N
O
O
Me
54c
173.4 168.6 145.0 134.1 133.2 130.0 128.7 124.9 80.6 71.2 62.8 52.7 48.9 25.5 24.5 21.5 11.4
210 200 190 180 170 160 150 140 130 120 110 100 90 80 70 60 50 40 30 20 10 0
f1 (ppm)

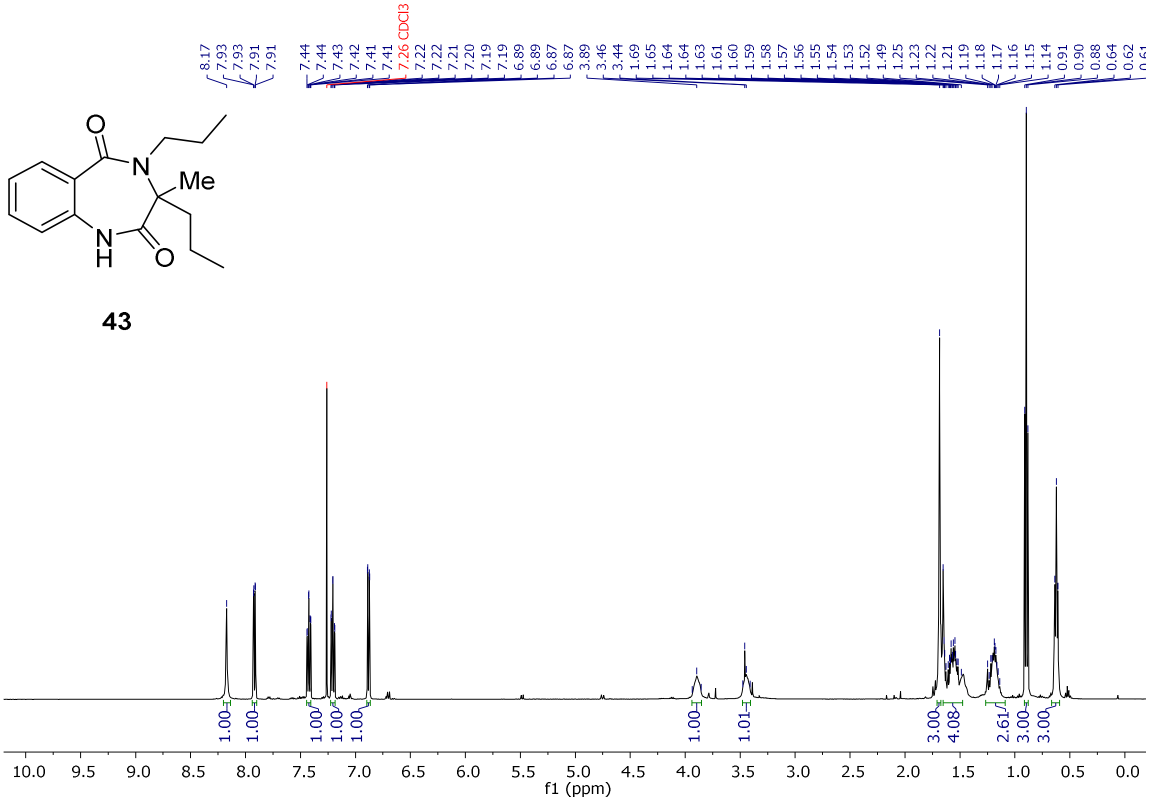
8.17 7.93 7.93 7.91 7.91 7.44 7.44 7.43 7.42 7.41 7.41 7.26 CDCl3 7.22 7.22 7.21 7.20 7.19 7.19 6.89 6.89 6.87 6.87 3.89 3.46 3.44 1.69 1.65 1.64 1.64 1.63 1.61 1.60 1.59 1.58 1.57 1.56 1.55 1.54 1.53 1.52 1.49 1.25 1.23 1.22 1.21 1.19 1.18 1.17 1.16 1.15 1.14 0.91 0.90 0.88 0.64 0.62
O
N
Me
N
H
O
1.00 1.00 1.00 1.00 1.00 1.00 1.01 3.00 4.08 2.61 3.00 3.00
10.0 9.5 9.0 8.5 8.0 7.5 7.0 6.5 6.0 5.5 5.0 4.5 4.0 3.5 3.0 2.5 2.0 1.5 1.0 0.5 0.0
f1 (ppm)

43

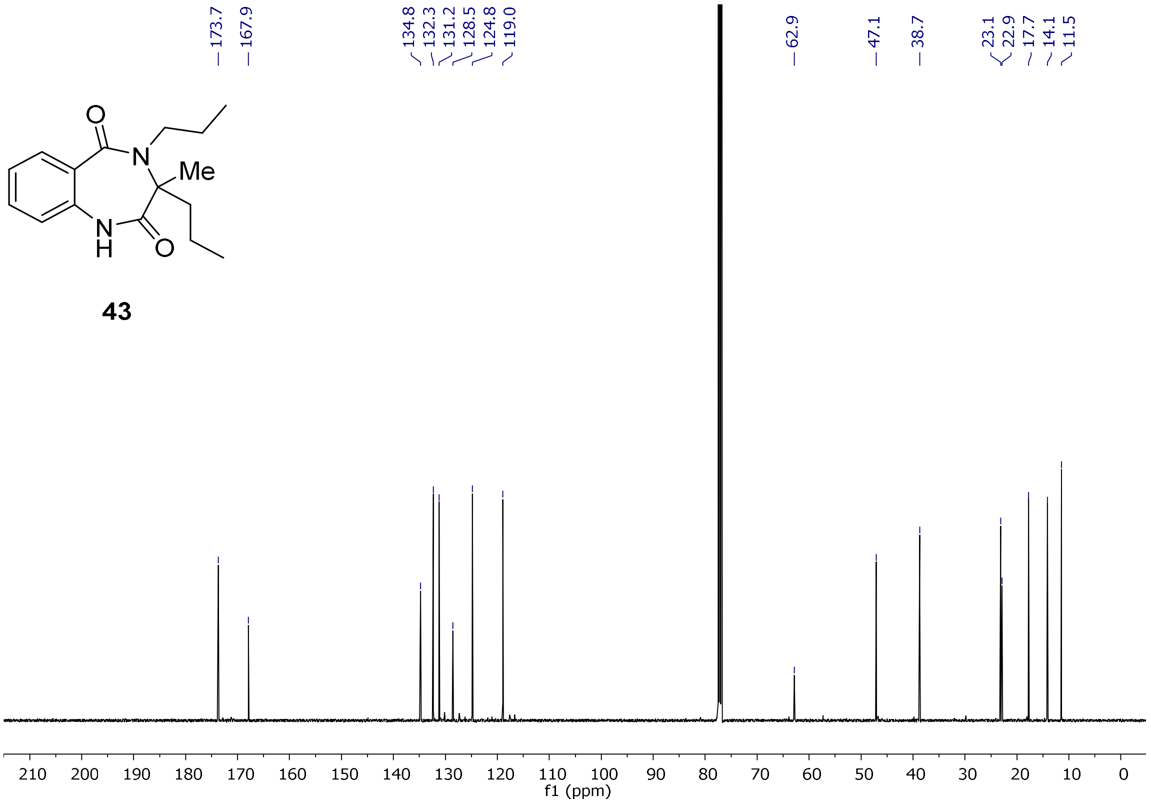
173.7 167.9 134.8 132.3 131.2 128.5 124.8 119.0 62.9 47.1 38.7 23.1 22.9 17.7 14.1 11.5
O
N
Me
N
H
O
210 200 190 180 170 160 150 140 130 120 110 100 90 80 70 60 50 40 30 20 10 0
f1 (ppm)

43

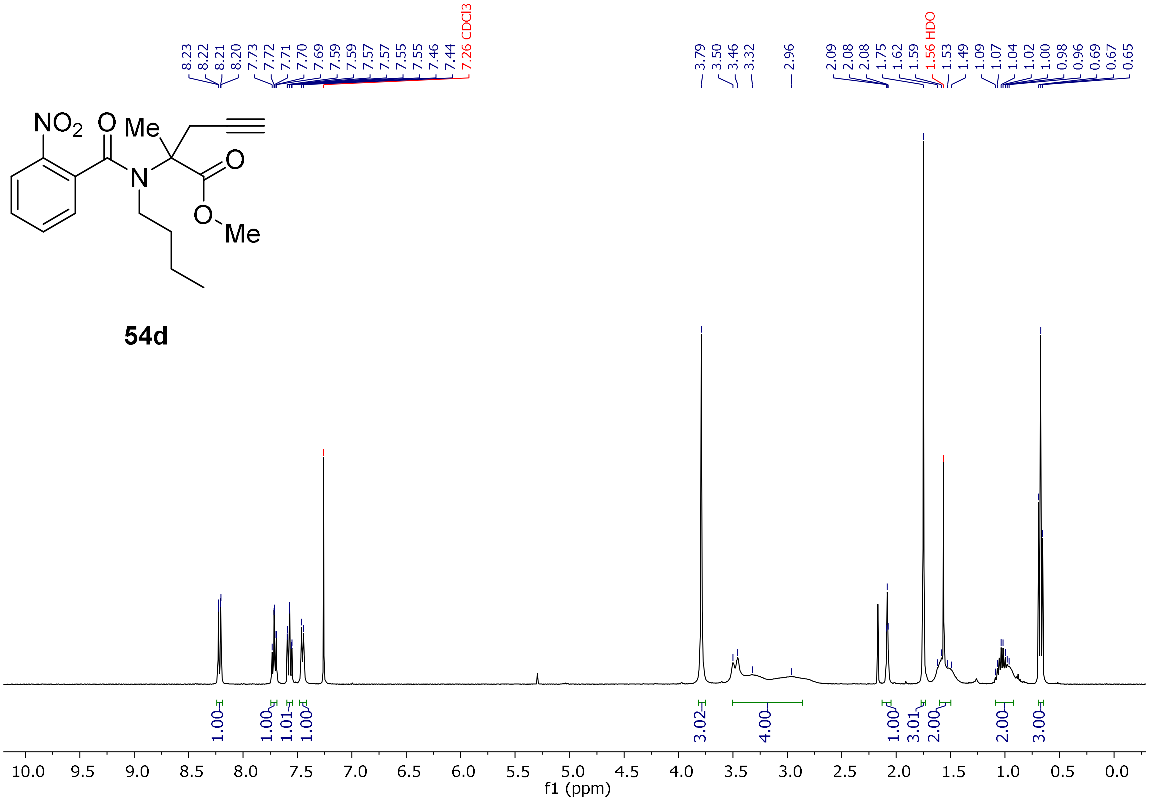

8.23 8.22 8.21 8.20 7.73 7.72 7.71 7.70 7.69 7.59 7.59 7.57 7.57 7.55 7.46 7.44
7.26 CDCl3
3.79 3.50 3.46 3.32 2.96 2.09 2.08 2.08 1.75 1.62 1.59
1.56 HDO
1.53 1.49 1.09 1.07 1.04 1.02 1.00 0.98 0.96 0.69 0.67 0.65
NO_2
O
Me
N
O
O
Me
54d
1.00 1.00 1.01 1.00 3.02 4.00 1.00 3.01 2.00 2.00 3.00
10.0 9.5 9.0 8.5 8.0 7.5 7.0 6.5 6.0 5.5 5.0 4.5 4.0 3.5 3.0 2.5 2.0 1.5 1.0 0.5 0.0
f1 (ppm)

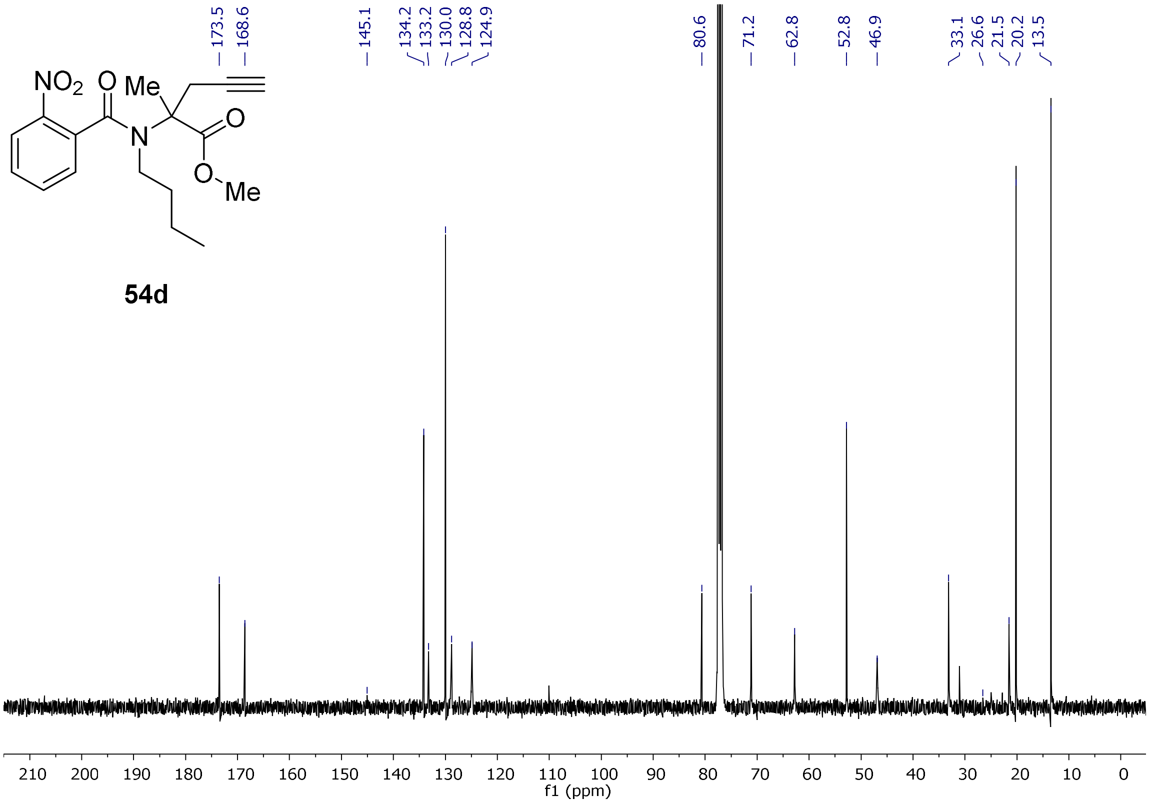

173.5 168.6 145.1 134.2 133.2 130.0 128.8 124.9 80.6 71.2 62.8 52.8 46.9 33.1 26.6 21.5 20.2 13.5
NO_2
O
Me
N
O
O
Me
54d
210 200 190 180 170 160 150 140 130 120 110 100 90 80 70 60 50 40 30 20 10 0
f1 (ppm)

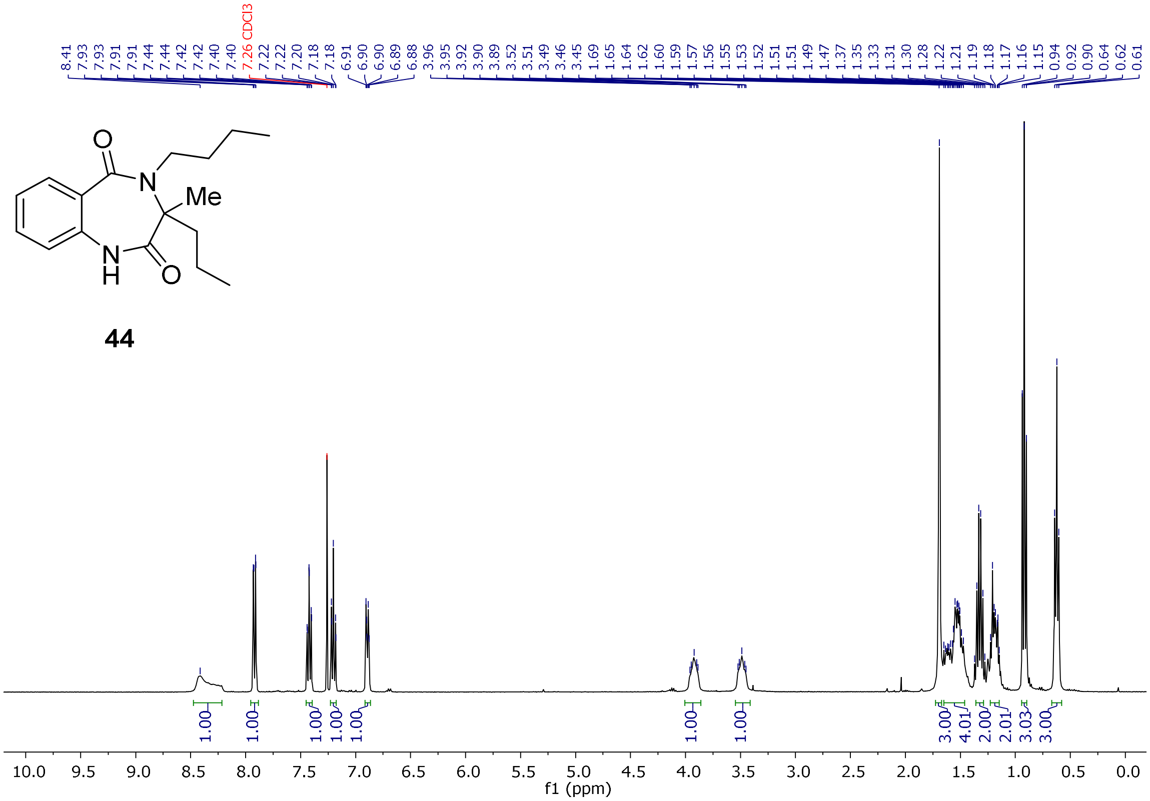
8.41 7.93 7.93 7.91 7.91 7.44 7.44 7.42 7.42 7.40 7.40 7.26 CDCl3 7.22 7.22 7.20 7.20 7.18 7.18 6.91 6.90 6.90 6.89 6.88 3.96 3.95 3.92 3.90 3.89 3.52 3.51 3.49 3.46 3.45 1.69 1.65 1.64 1.62 1.60 1.59 1.57 1.56 1.55 1.53 1.52 1.51 1.51 1.49 1.47 1.37 1.35 1.33 1.31 1.30 1.28 1.22 1.21 1.19 1.18 1.17 1.16 1.15 0.94 0.92 0.90 0.64 0.62 0.61
O
N
Me
N
H
O
44
1.00
1.00
1.00
1.00
1.00
1.00
1.00
3.00
4.01
2.00
2.01
3.03
3.00
f1 (ppm)

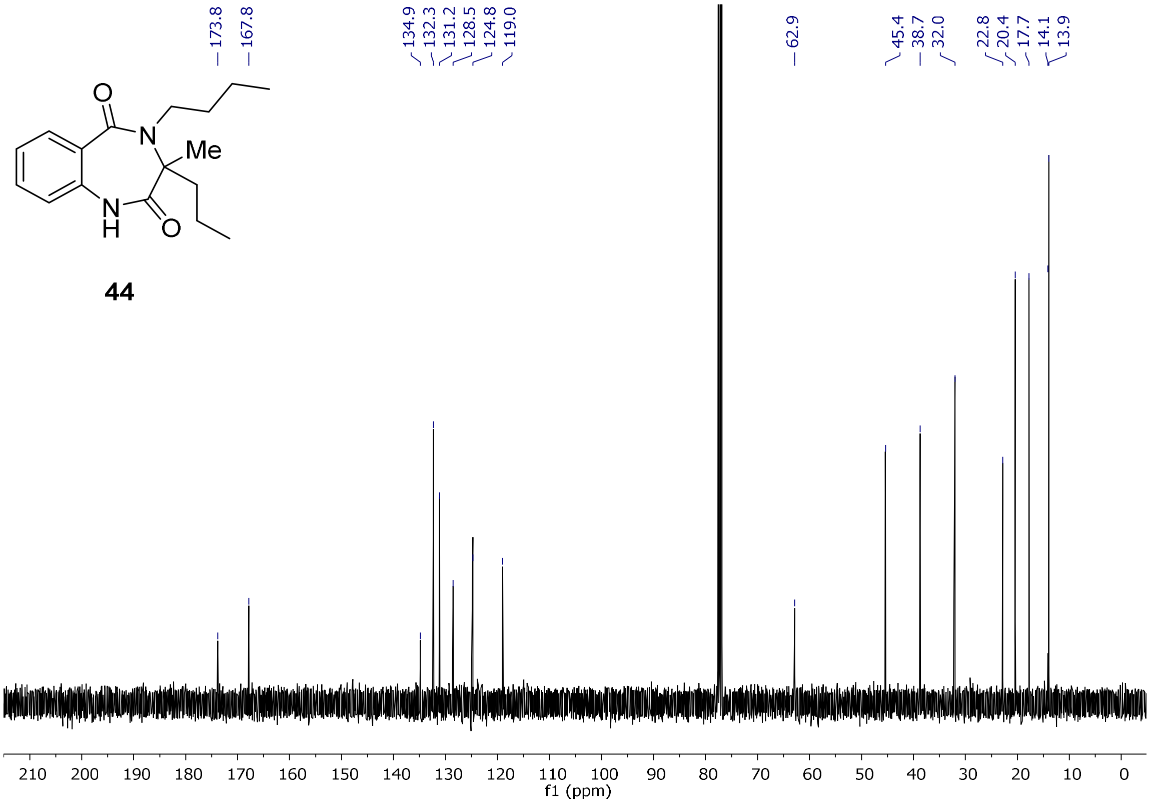
173.8
167.8
134.9
132.3
131.2
128.5
124.8
119.0
62.9
45.4
38.7
32.0
22.8
20.4
17.7
14.1
13.9
O
N
Me
N
H
O
44
f1 (ppm)

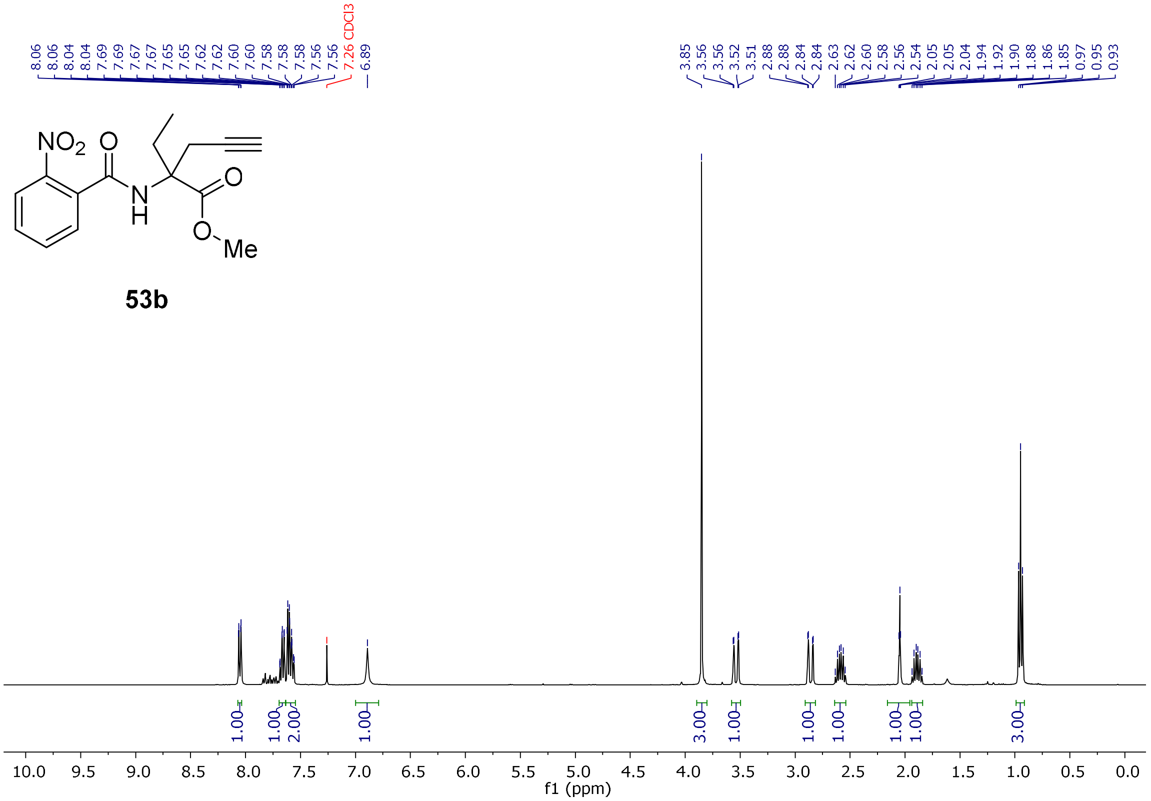

53b

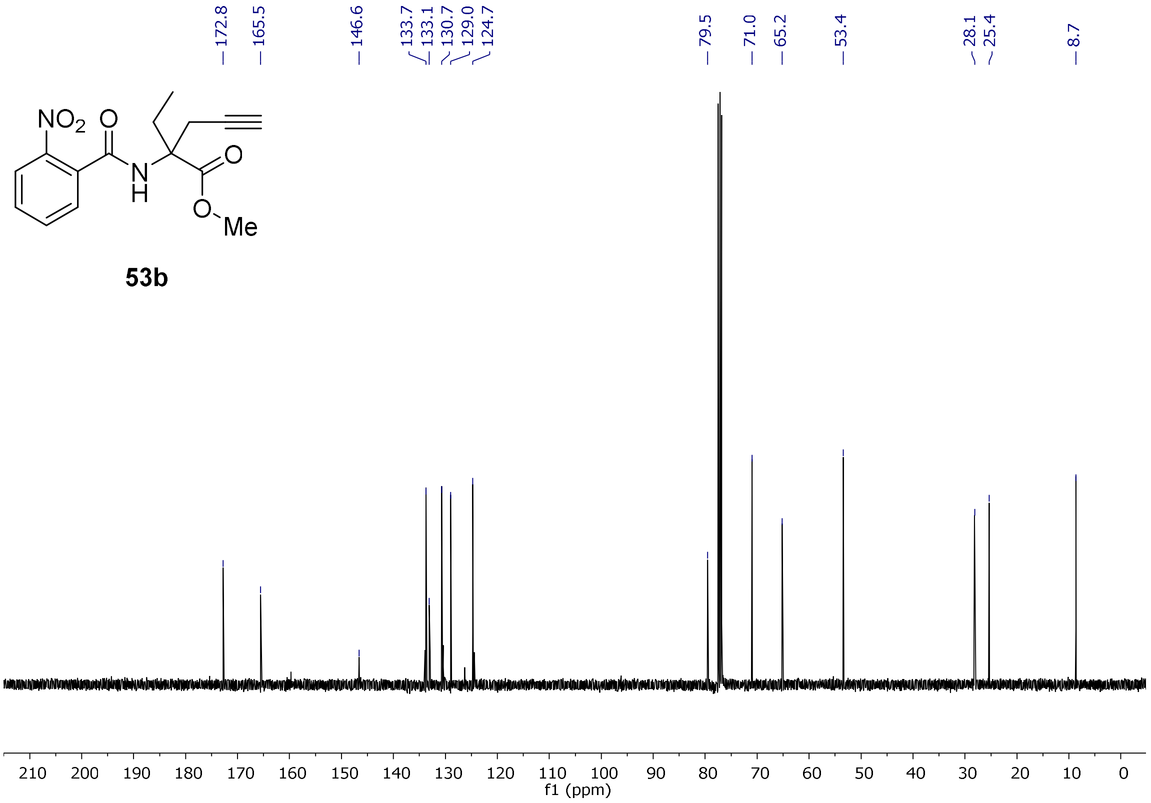

53b

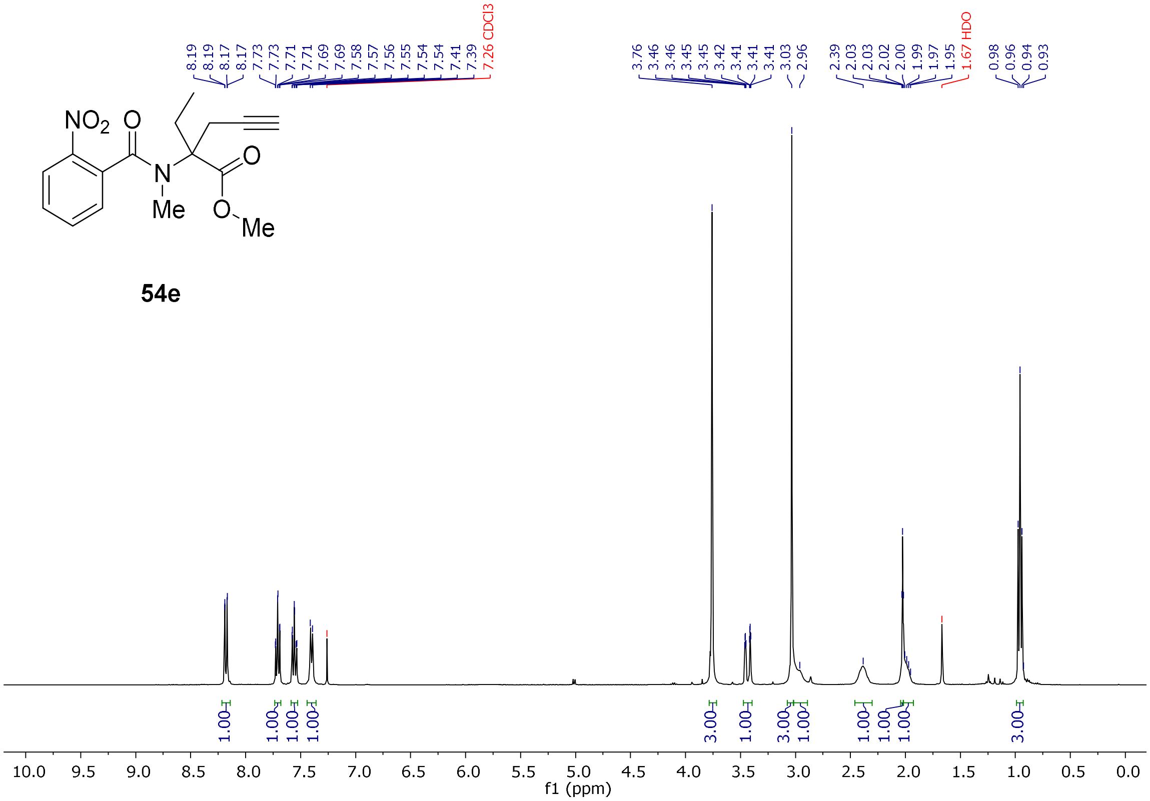
8.19
8.19
8.17
8.17
7.73
7.73
7.71
7.71
7.69
7.69
7.58
7.57
7.56
7.55
7.54
7.54
7.41
7.39
7.26 CDCl3
3.76
3.46
3.46
3.45
3.45
3.42
3.41
3.41
3.03
2.96
2.39
2.03
2.03
2.02
2.00
1.99
1.97
1.95
1.67 HDO
0.98
0.96
0.94
0.93
NO_2
O
N
Me
O
O
Me
1.00
1.00
1.00
1.00
3.00
1.00
3.00
1.00
1.00
1.00
1.00
3.00
10.0 9.5 9.0 8.5 8.0 7.5 7.0 6.5 6.0 5.5 5.0 4.5 4.0 3.5 3.0 2.5 2.0 1.5 1.0 0.5 0.0
f1 (ppm)

54e

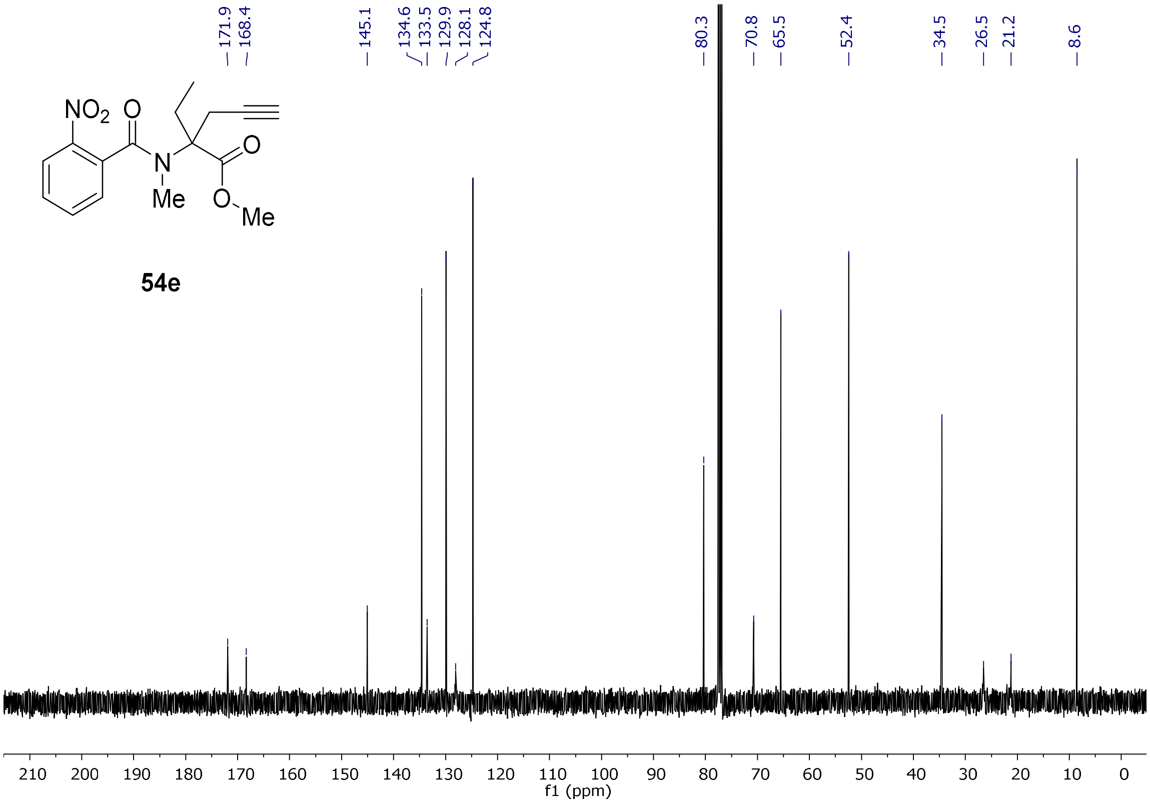
171.9
168.4
145.1
134.6
133.5
129.9
128.1
124.8
80.3
70.8
65.5
52.4
34.5
26.5
21.2
8.6
NO_2
O
N
Me
O
O
Me
210 200 190 180 170 160 150 140 130 120 110 100 90 80 70 60 50 40 30 20 10 0
f1 (ppm)

54e

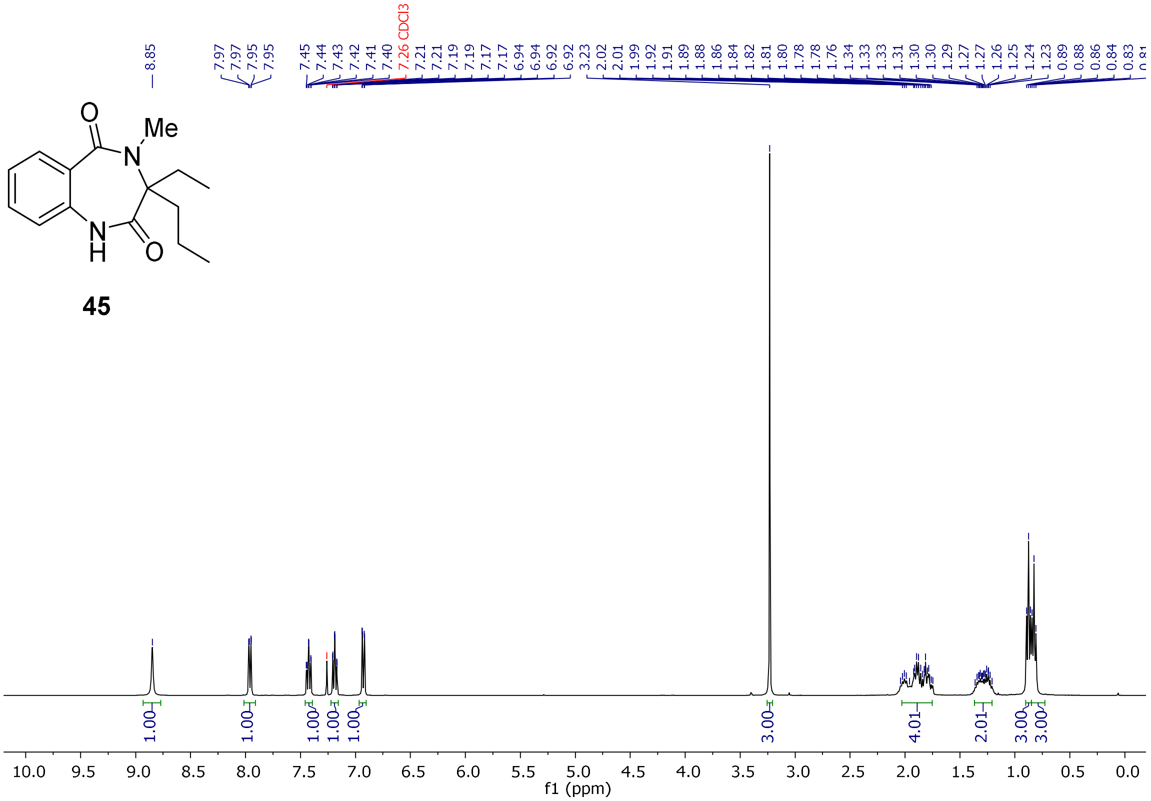

8.85
7.97
7.97
7.95
7.95
7.45
7.44
7.43
7.42
7.41
7.40
7.26 CDCl3
7.21
7.21
7.19
7.19
7.17
7.17
6.94
6.94
6.92
6.92
3.23
2.02
2.01
1.99
1.92
1.91
1.89
1.88
1.86
1.84
1.82
1.81
1.80
1.78
1.78
1.76
1.34
1.33
1.33
1.31
1.30
1.30
1.29
1.27
1.27
1.26
1.25
1.24
1.23
0.89
0.88
0.86
0.84
0.83
O
Me
N
N
H
O
45
1.00
1.00
1.00
1.00
1.00
3.00
4.01
2.01
3.00
3.00
10.0 9.5 9.0 8.5 8.0 7.5 7.0 6.5 6.0 5.5 5.0 4.5 4.0 3.5 3.0 2.5 2.0 1.5 1.0 0.5 0.0
f1 (ppm)

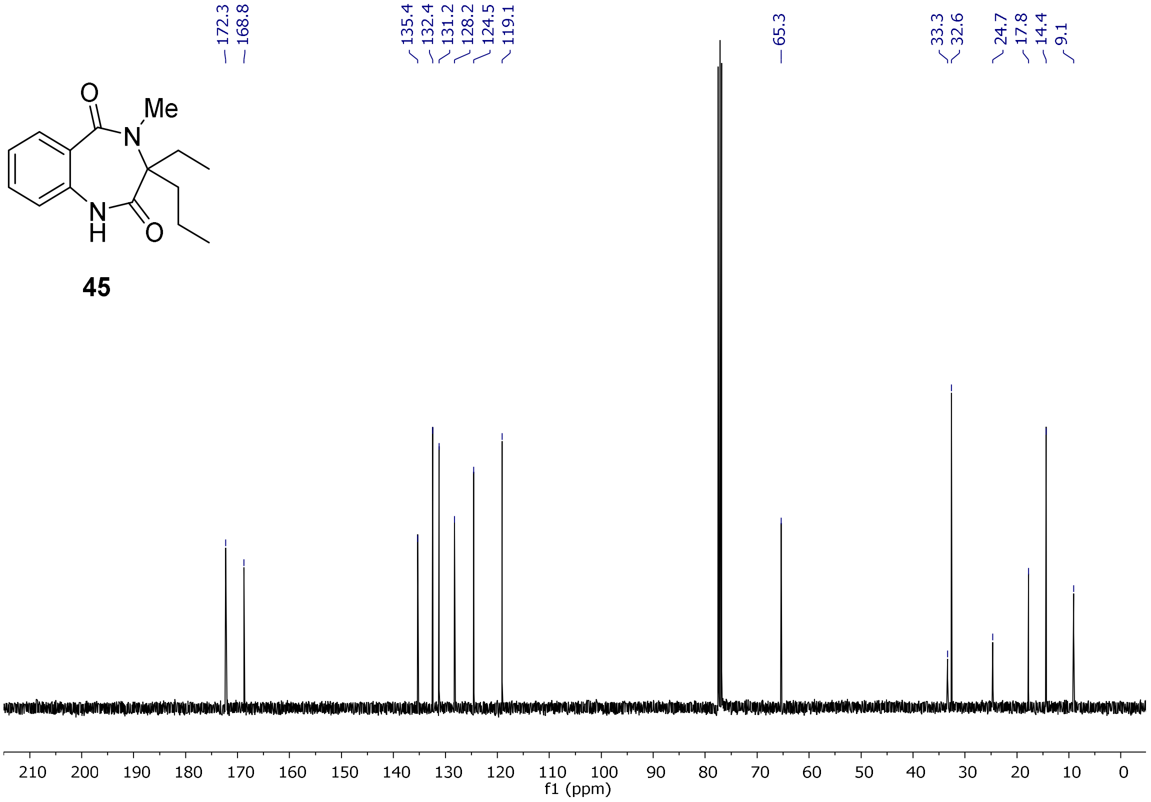

172.3
168.8
135.4
132.4
131.2
128.2
124.5
119.1
65.3
33.3
32.6
24.7
17.8
14.4
9.1
O
Me
N
N
H
O
45
210 200 190 180 170 160 150 140 130 120 110 100 90 80 70 60 50 40 30 20 10 0
f1 (ppm)

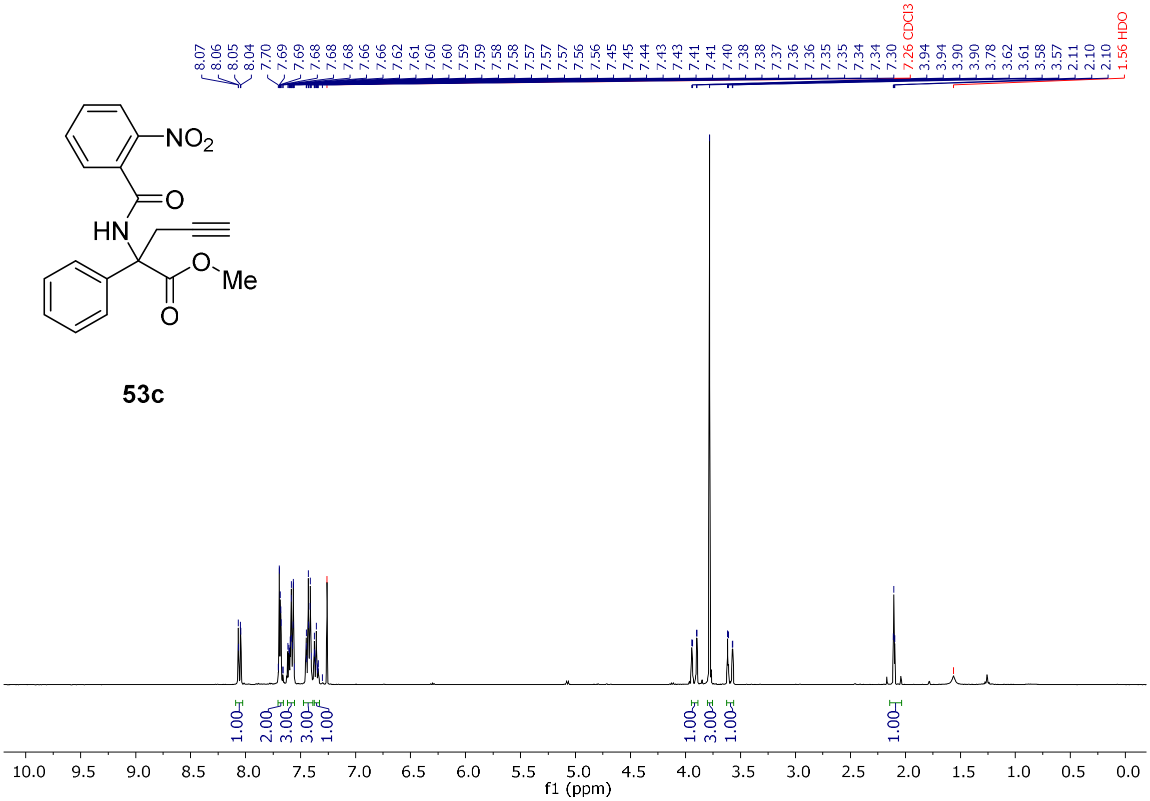

53c

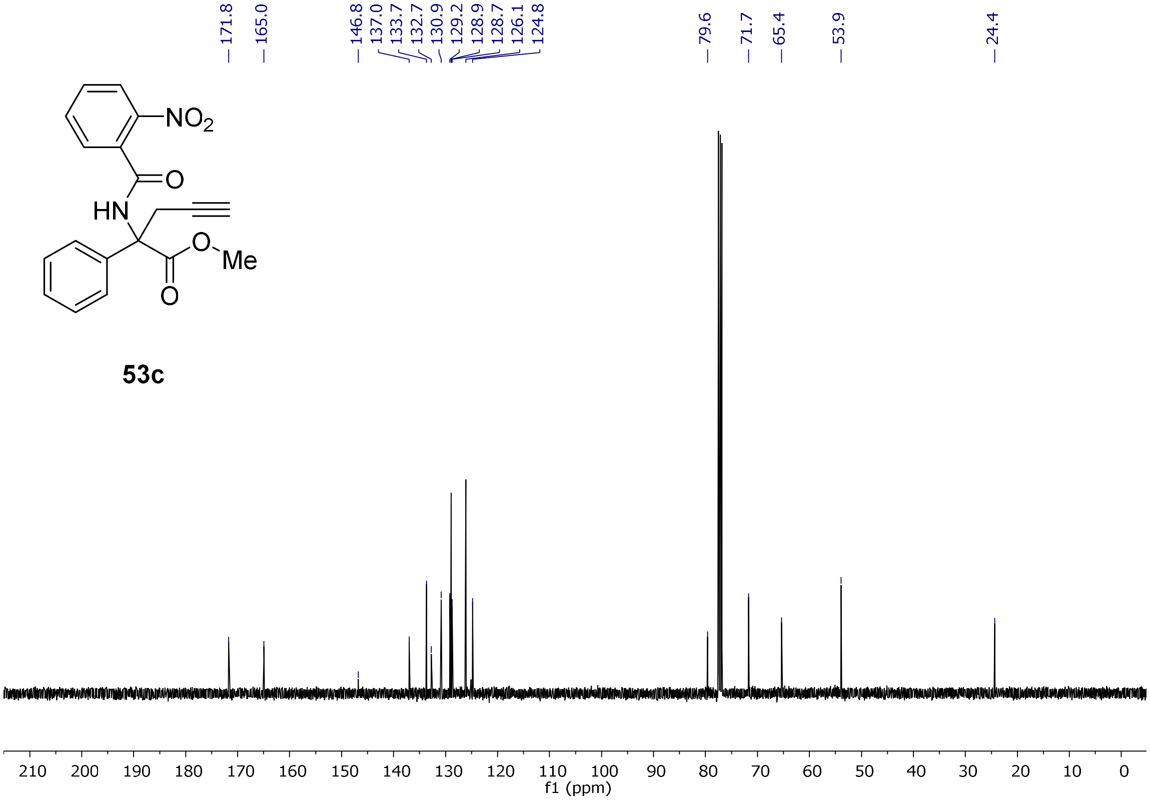

53c

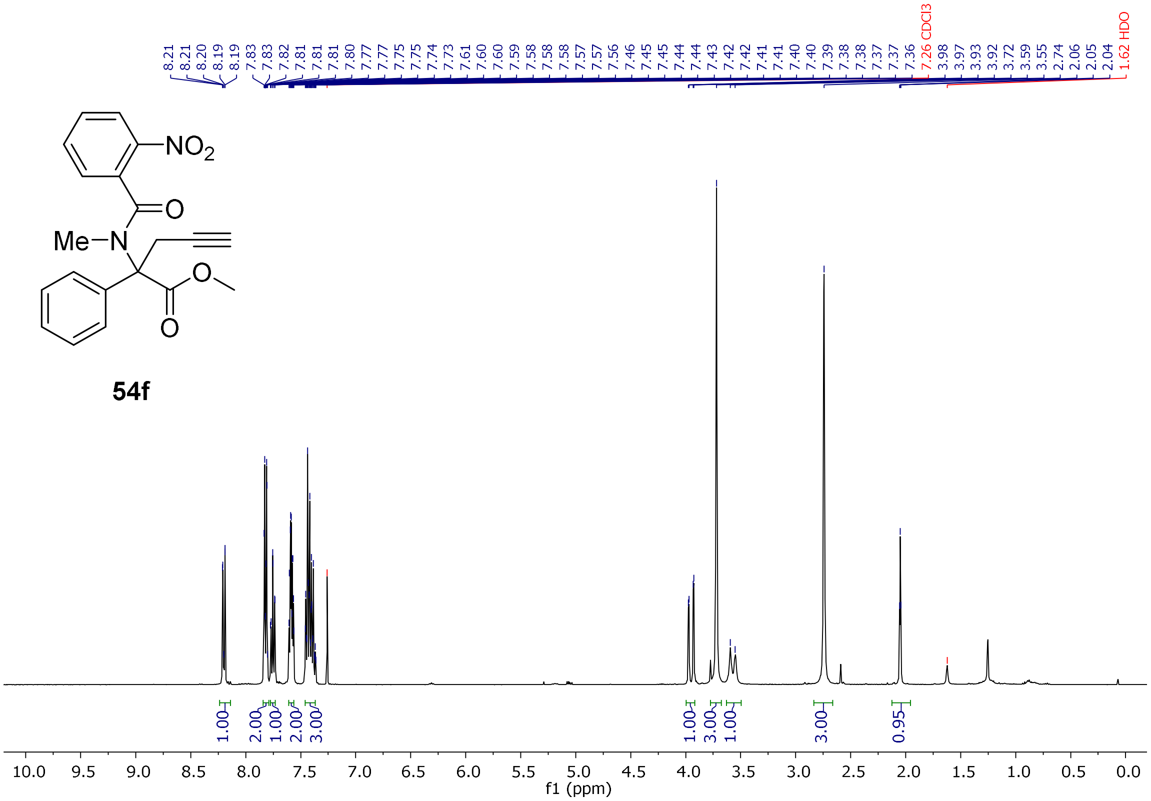
NO2
Me
N
O
54f
f1 (ppm)

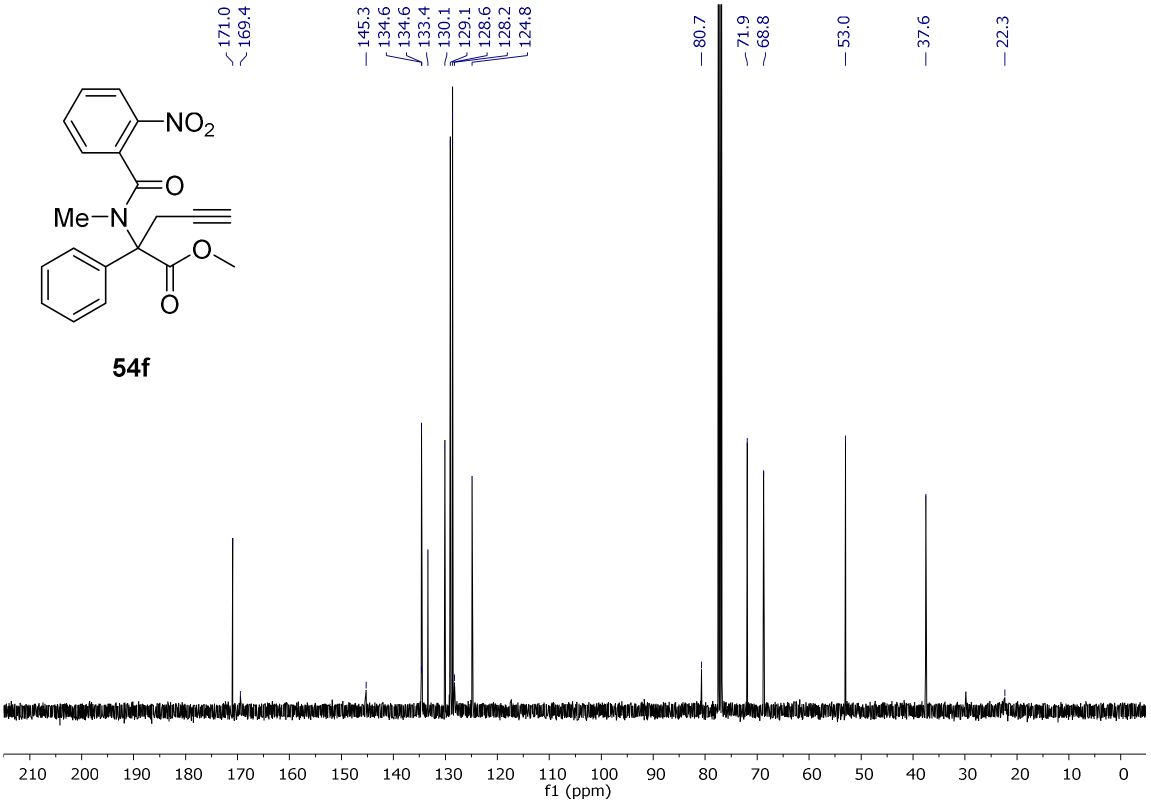
171.0
169.4
145.3
134.6
134.6
133.4
130.1
129.1
128.6
128.2
124.8
80.7
71.9
68.8
53.0
37.6
22.3
NO2
Me
N
O
54f
f1 (ppm)

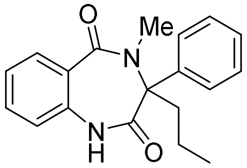

46

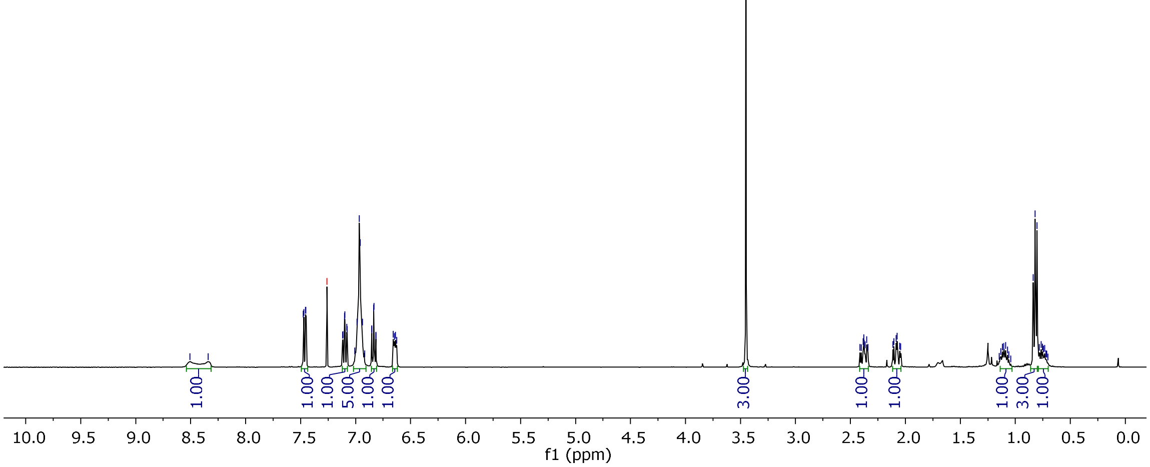

8.51 8.34 7.48 7.47 7.46 7.45 7.26 CDCl3 7.12 7.12 7.10 7.10 7.08 7.01 6.99 6.97 6.96 6.94 6.92 6.85 6.84 6.83 6.82 6.81 6.66 6.65 6.64 6.63 3.45 2.41 2.40 2.38 2.38 2.37 2.37 2.35 2.34 2.11 2.10 2.09 2.08 2.05 2.04 1.15 1.13 1.12 1.11 1.09 1.07 1.06 1.04 0.84 0.82 0.80 0.78 0.76 0.75 0.73 0.72 0.72 0.71 0.70
1.00
1.00 1.00 5.00 1.00 1.00
3.00
1.00 1.00
1.00 3.00 1.00
10.0 9.5 9.0 8.5 8.0 7.5 7.0 6.5 6.0 5.5 5.0 4.5 4.0 3.5 3.0 2.5 2.0 1.5 1.0 0.5 0.0
f1 (ppm)

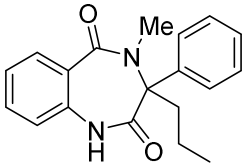

46

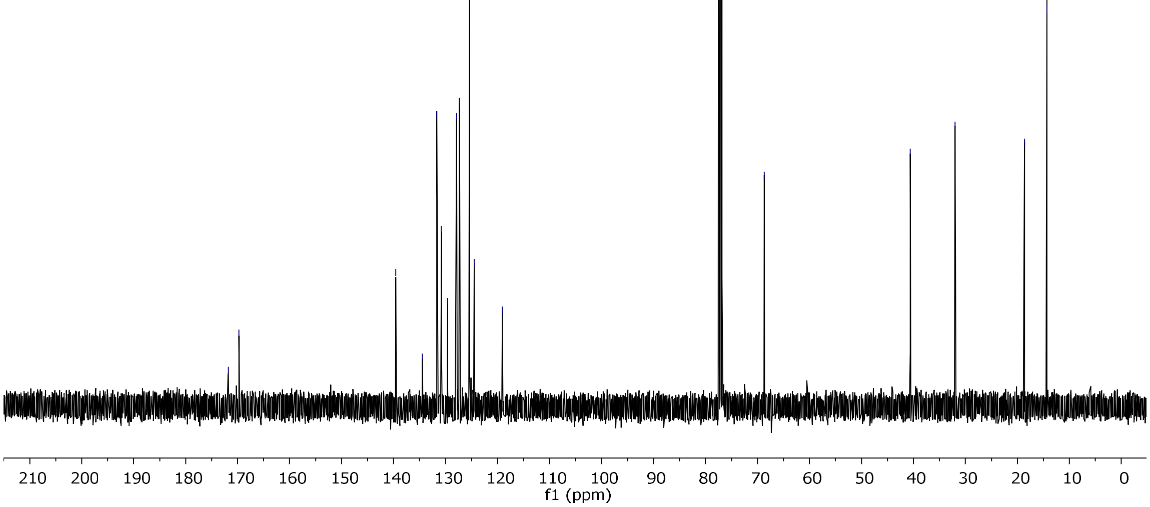

171.8 169.8
139.6 134.5 131.7 130.8 129.6 127.9 127.3 125.4 124.5 119.1
68.7
40.6
32.0
18.6
14.3
210 200 190 180 170 160 150 140 130 120 110 100 90 80 70 60 50 40 30 20 10 0
f1 (ppm)

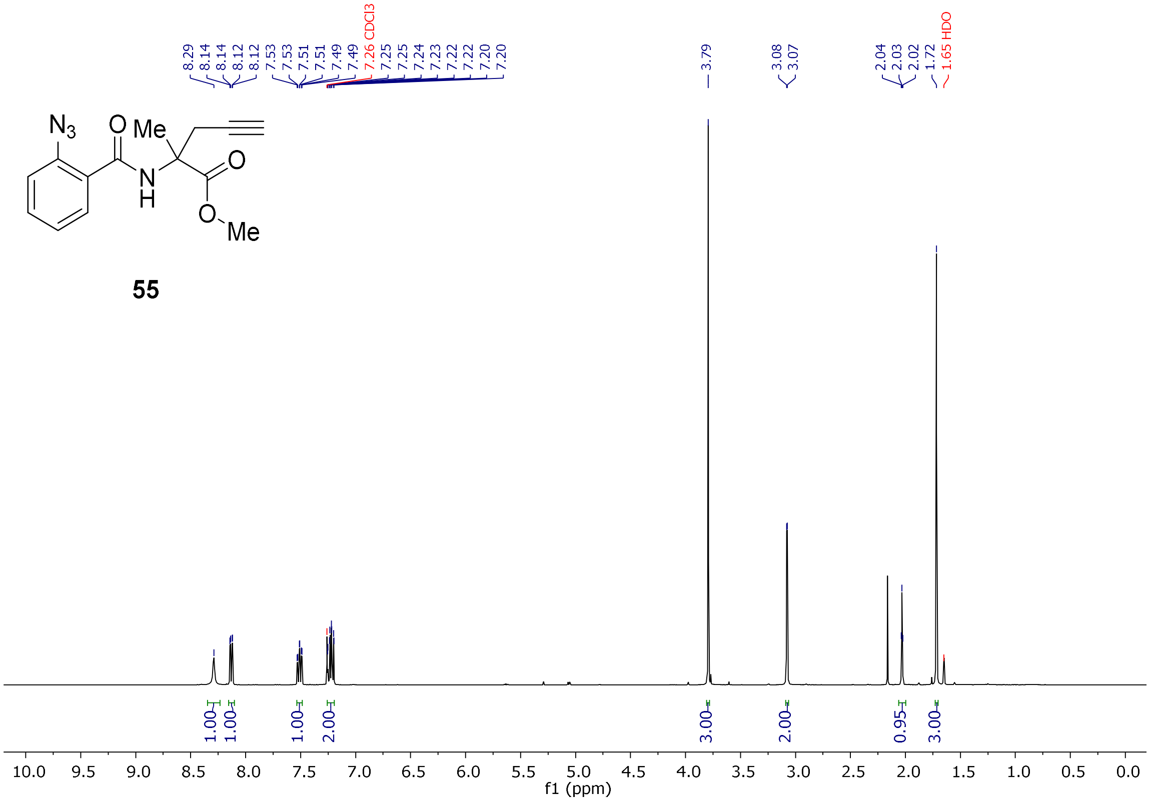
8.29
8.14
8.14
8.12
8.12
7.53
7.53
7.51
7.51
7.49
7.49
7.26 CDCl3
7.25
7.25
7.24
7.23
7.22
7.22
7.20
7.20
3.79
3.08
3.07
2.04
2.03
2.02
1.72
1.65 HDO
N3
O
Me
N
H
O
O
Me
1.00
1.00
1.00
2.00
3.00
2.00
0.95
3.00
10.0 9.5 9.0 8.5 8.0 7.5 7.0 6.5 6.0 5.5 5.0 4.5 4.0 3.5 3.0 2.5 2.0 1.5 1.0 0.5 0.0
f1 (ppm)

55

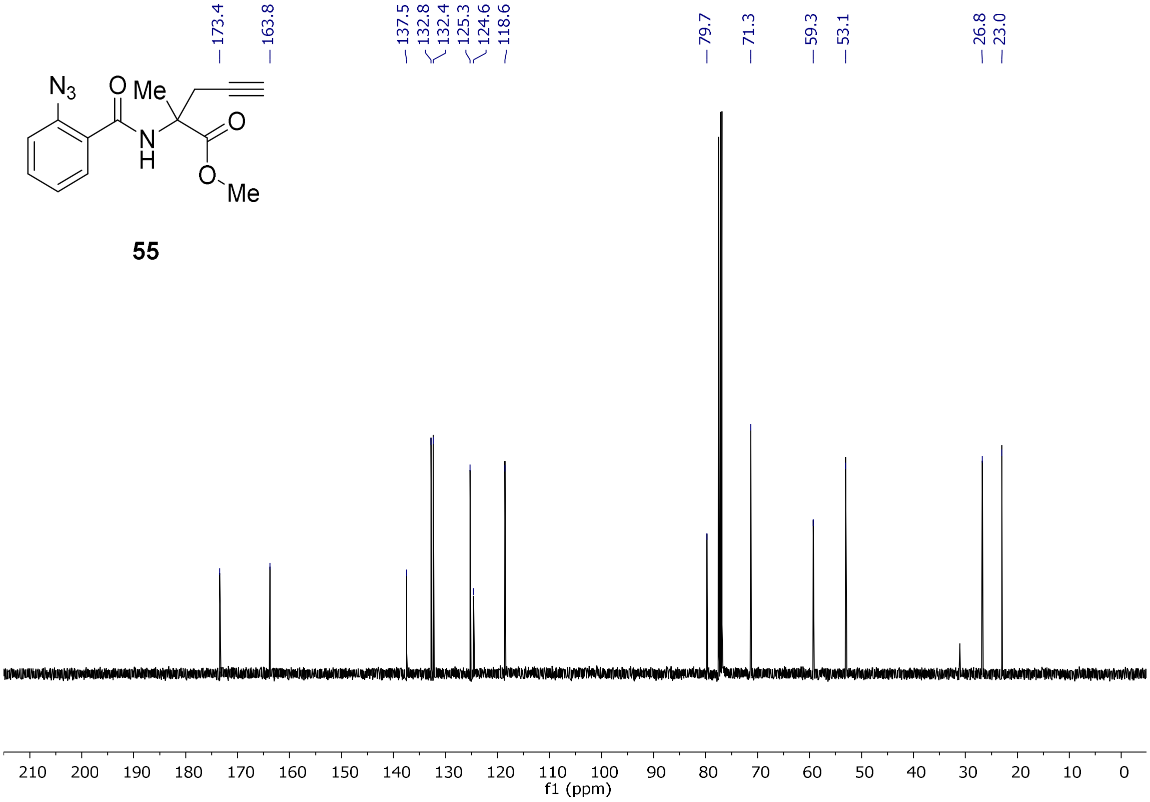
173.4
163.8
137.5
132.8
132.4
125.3
124.6
118.6
79.7
71.3
59.3
53.1
26.8
23.0
N3
O
Me
N
H
O
O
Me
210 200 190 180 170 160 150 140 130 120 110 100 90 80 70 60 50 40 30 20 10 0
f1 (ppm)

55

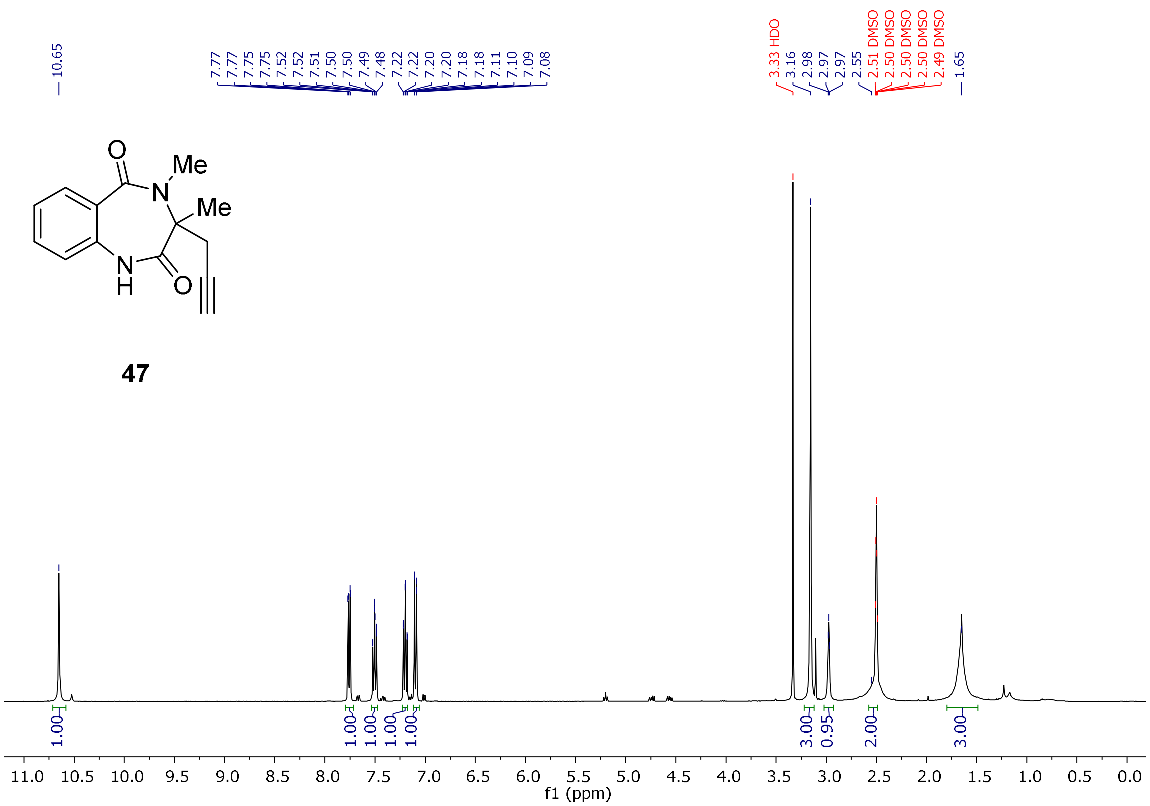

47

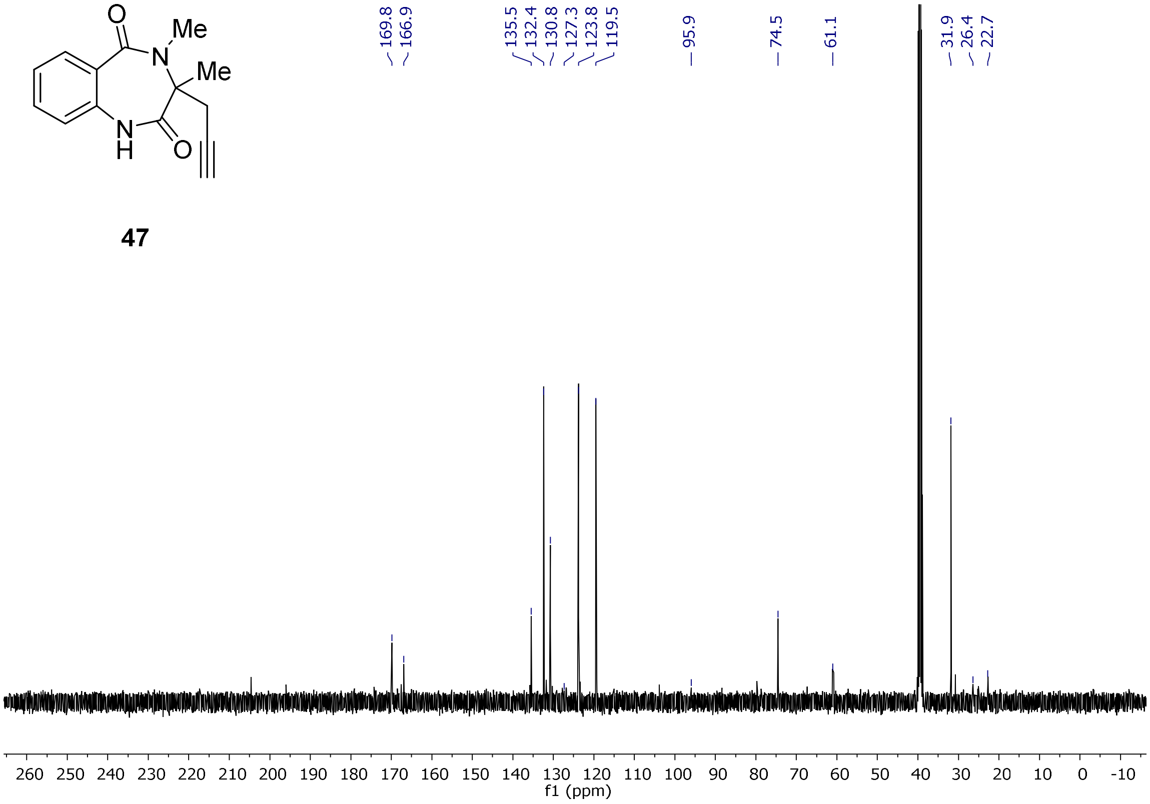

47

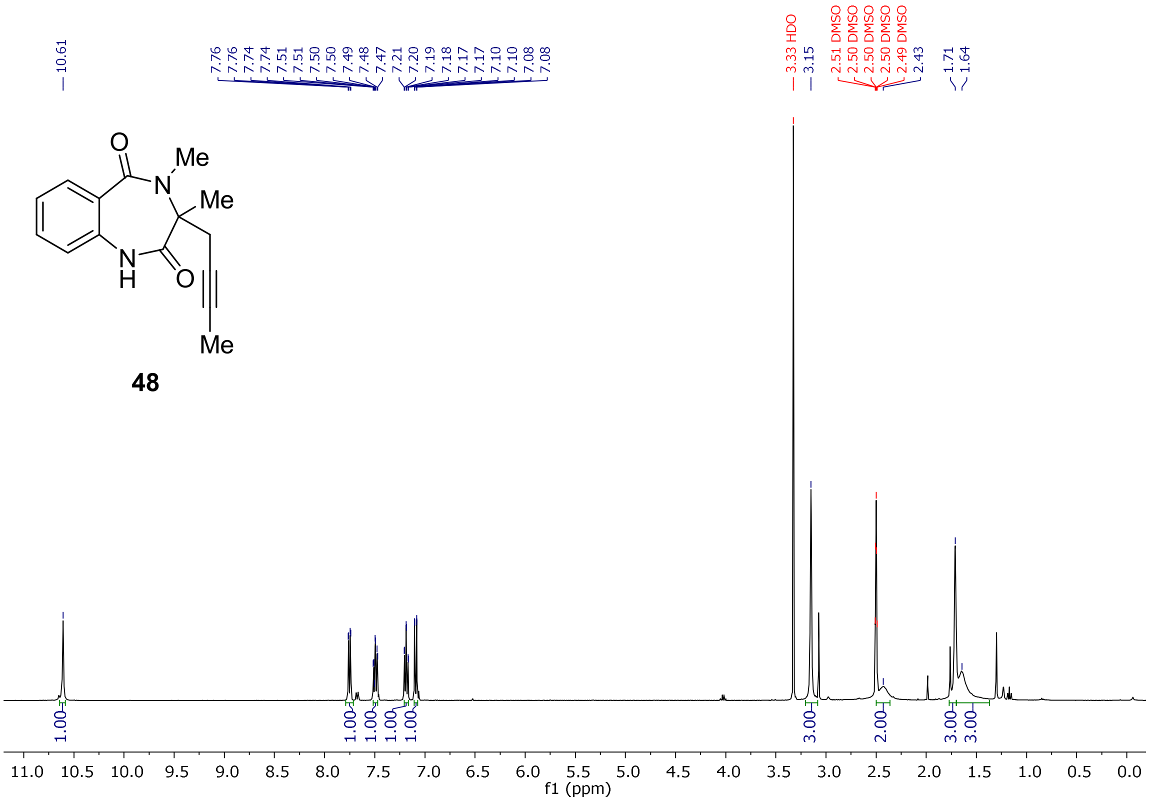
10.61
7.76
7.76
7.74
7.74
7.51
7.51
7.50
7.50
7.49
7.48
7.47
7.21
7.20
7.19
7.18
7.17
7.17
7.10
7.10
7.08
7.08
3.33 HDO
3.15
2.51 DMSO
2.50 DMSO
2.50 DMSO
2.50 DMSO
2.49 DMSO
2.43
1.71
1.64
O
Me
N
Me
N
H
O
Me
48
1.00
1.00
1.00
1.00
1.00
3.00
2.00
3.00
3.00
11.0 10.5 10.0 9.5 9.0 8.5 8.0 7.5 7.0 6.5 6.0 5.5 5.0 4.5 4.0 3.5 3.0 2.5 2.0 1.5 1.0 0.5 0.0
f1 (ppm)

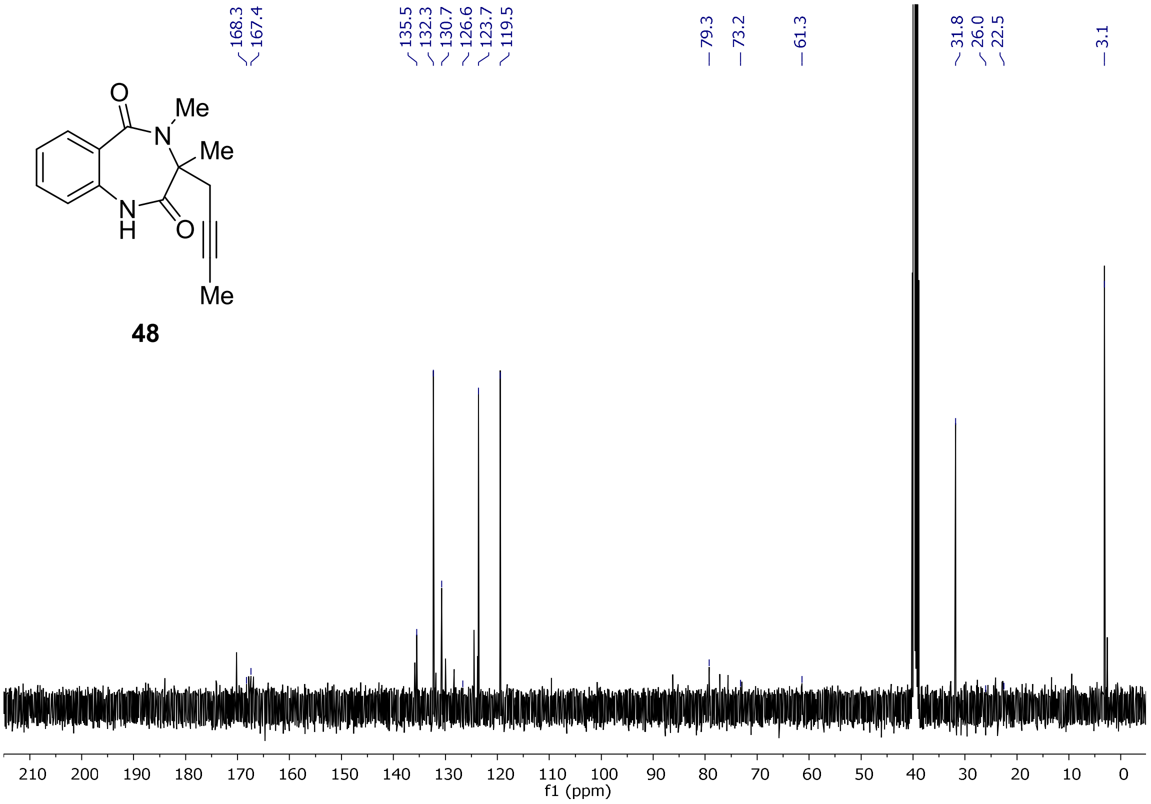
168.3
167.4
135.5
132.3
130.7
126.6
123.7
119.5
79.3
73.2
61.3
31.8
26.0
22.5
3.1
O
Me
N
Me
N
H
O
Me
48
210 200 190 180 170 160 150 140 130 120 110 100 90 80 70 60 50 40 30 20 10 0
f1 (ppm)

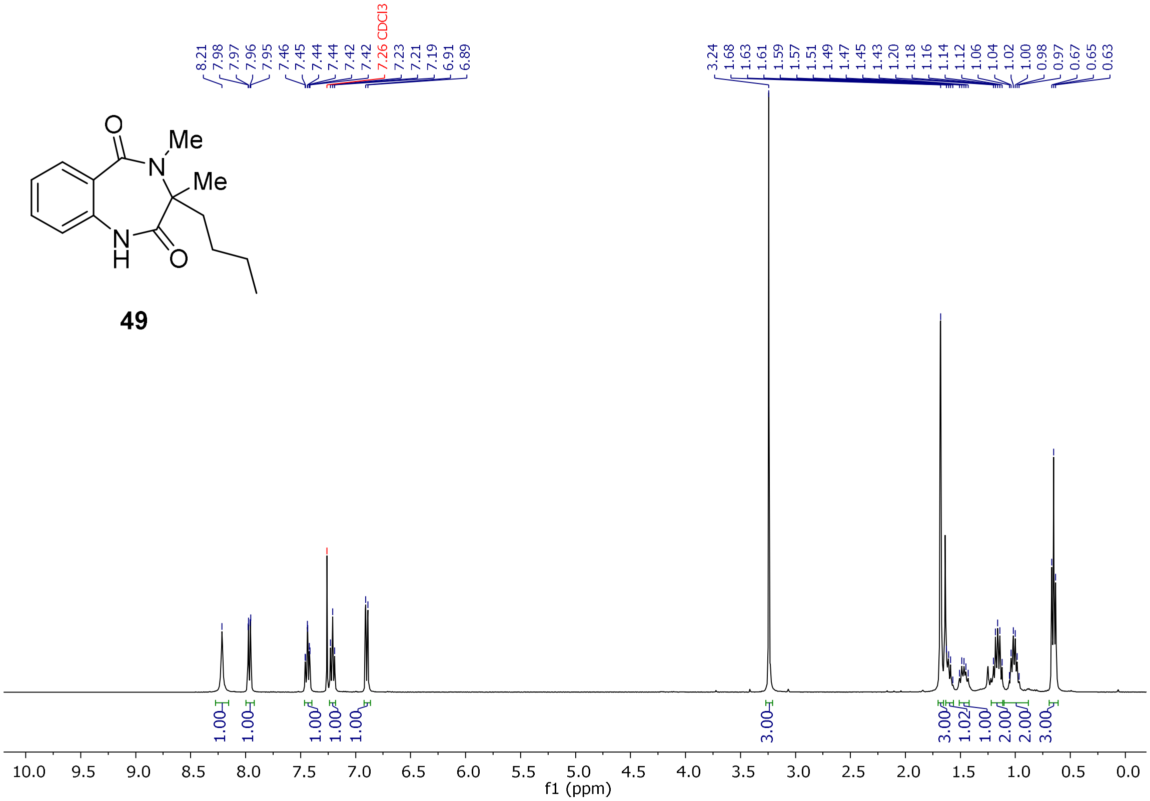

49

8.21
7.98
7.97
7.96
7.95
7.46
7.45
7.44
7.44
7.42
7.42
7.26 CDCl3
7.23
7.21
7.19
6.91
6.89
3.24
1.68
1.63
1.61
1.59
1.57
1.51
1.49
1.47
1.45
1.43
1.20
1.18
1.16
1.14
1.12
1.06
1.04
1.02
1.00
0.98
0.97
0.67
0.65
0.63
1.00
1.00
1.00
1.00
1.00
3.00
3.00
1.02
1.00
2.00
2.00
3.00
f1 (ppm)

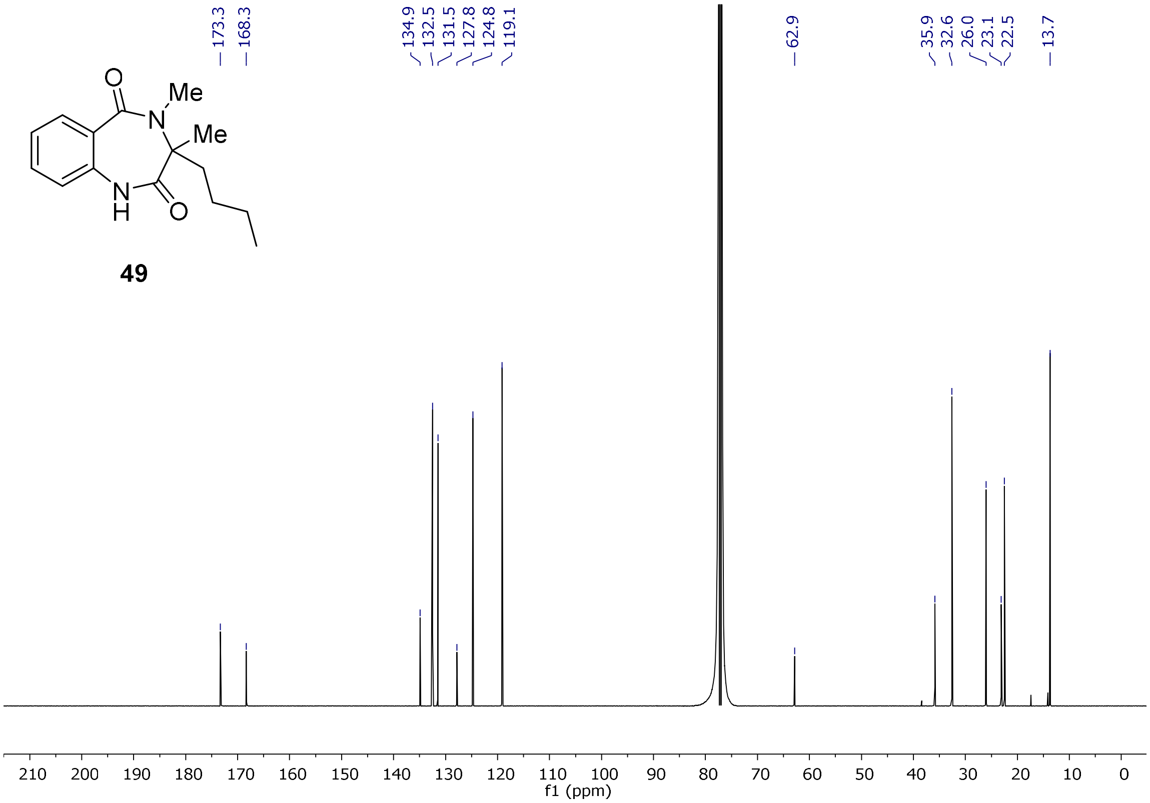

49

173.3
168.3
134.9
132.5
131.5
127.8
124.8
119.1
62.9
35.9
32.6
26.0
23.1
22.5
13.7
f1 (ppm)

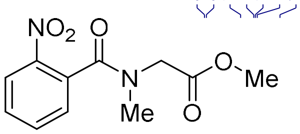

57

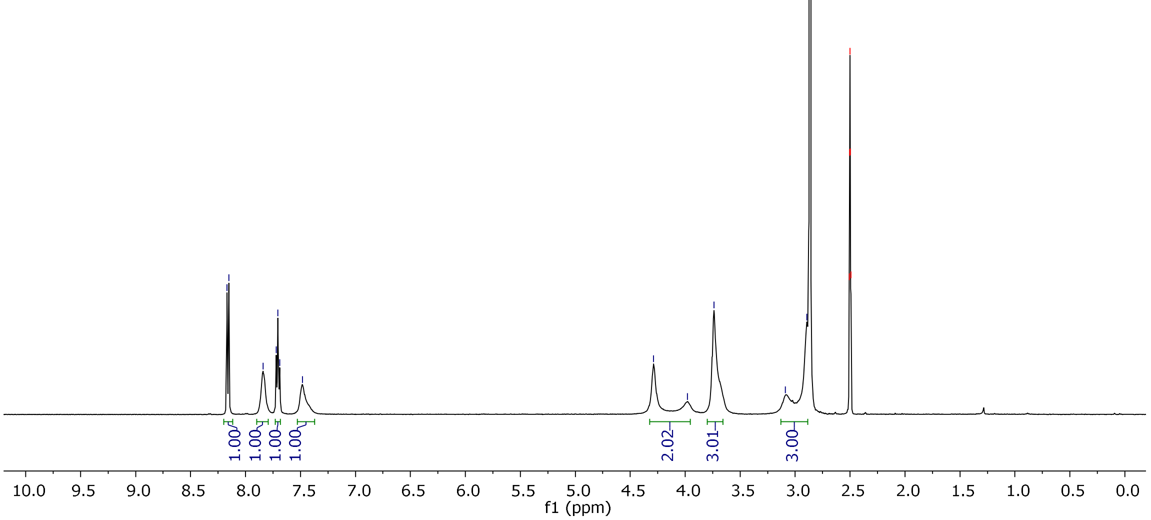
8.17
8.15
7.84
7.72
7.71
7.69
7.48
4.29
3.98
3.74
3.09
2.89
2.87 HDO
2.51 hot-dmso
2.50 hot-dmso
2.50 hot-dmso
2.50 hot-dmso
2.49 hot-dmso
1.00
1.00
1.00
1.00
2.02
3.01
3.00
f1 (ppm)

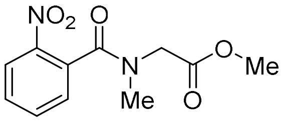

57

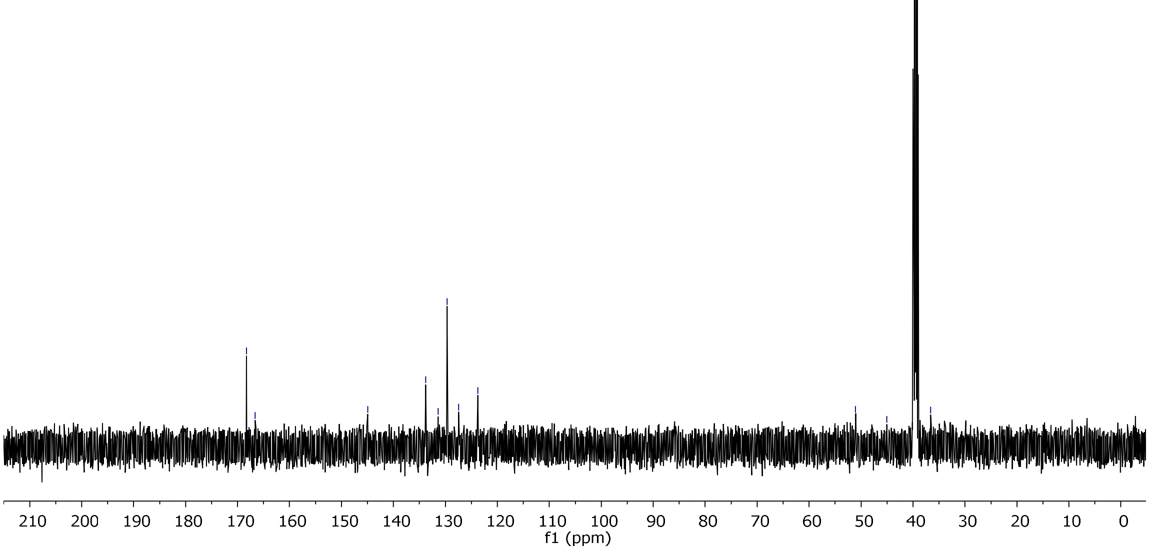
168.3
166.6
145.0
133.8
131.4
129.7
127.4
123.7
51.1
45.0
36.6
f1 (ppm)

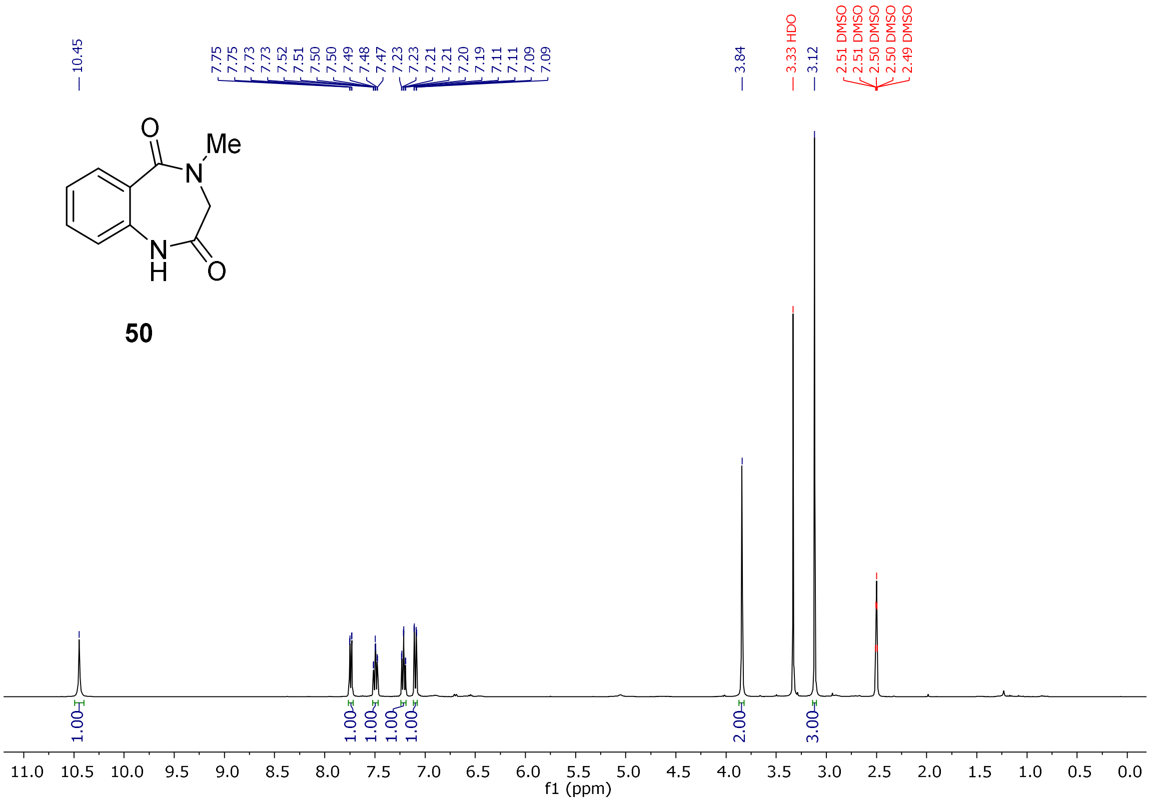
10.45
7.75 7.75 7.73 7.73 7.52 7.51 7.50 7.50 7.49 7.48 7.47 7.23 7.23 7.21 7.21 7.20 7.19 7.11 7.11 7.09 7.09
3.84
3.33 HDO
3.12
2.51 DMSO 2.51 DMSO 2.50 DMSO 2.50 DMSO 2.49 DMSO
O
Me
N
N
H
O
50
1.00 1.00 1.00 1.00 1.00 2.00 3.00
11.0 10.5 10.0 9.5 9.0 8.5 8.0 7.5 7.0 6.5 6.0 5.5 5.0 4.5 4.0 3.5 3.0 2.5 2.0 1.5 1.0 0.5 0.0
f1 (ppm)

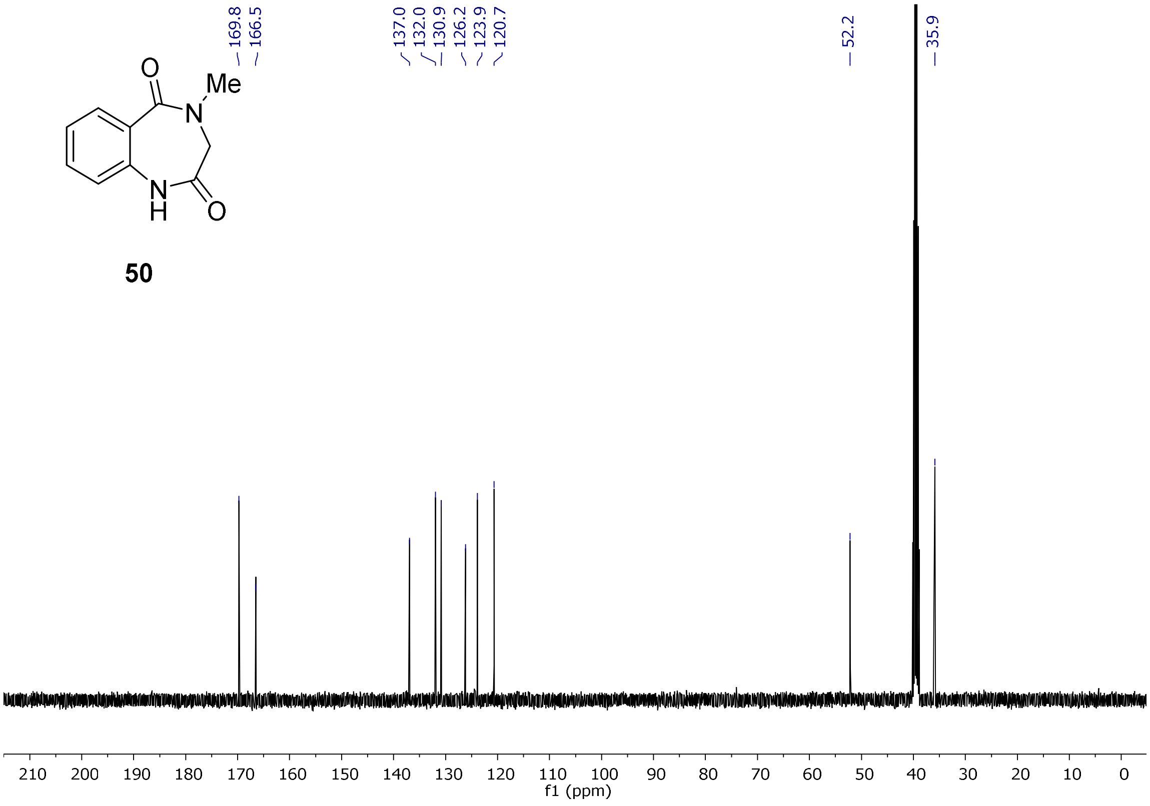
169.8 166.5
137.0 132.0 130.9 126.2 123.9 120.7
52.2
35.9
O
Me
N
N
H
O
50
210 200 190 180 170 160 150 140 130 120 110 100 90 80 70 60 50 40 30 20 10 0
f1 (ppm)

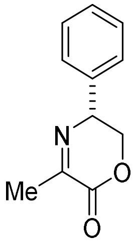
N
O
Me
O

(*R*)-61

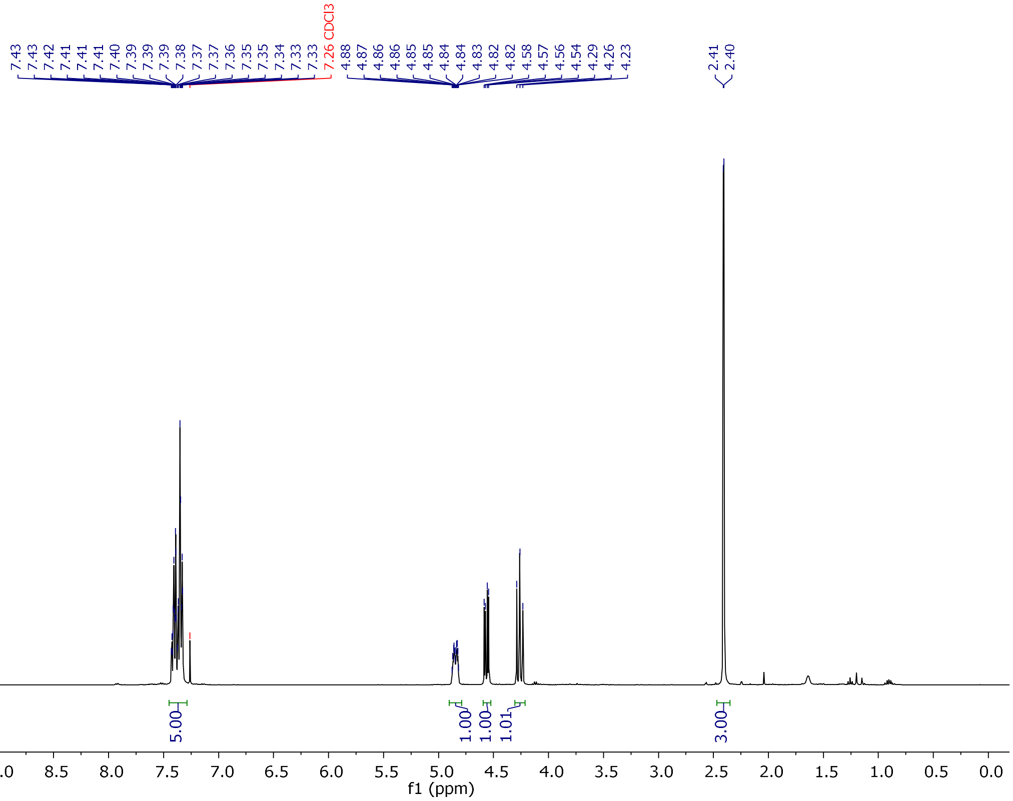
7.43
7.43
7.42
7.41
7.41
7.41
7.40
7.39
7.39
7.39
7.38
7.37
7.37
7.36
7.35
7.35
7.34
7.33
7.33
7.26 CDCl3
4.88
4.87
4.86
4.86
4.85
4.85
4.84
4.84
4.83
4.82
4.82
4.58
4.57
4.56
4.54
4.29
4.26
4.23
2.41
2.40
5.00
1.00
1.00
1.01
3.00
10.0 9.5 9.0 8.5 8.0 7.5 7.0 6.5 6.0 5.5 5.0 4.5 4.0 3.5 3.0 2.5 2.0 1.5 1.0 0.5 0.0
f1 (ppm)

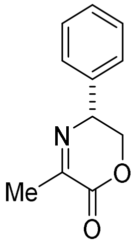
N
O
Me
O

(*R*)-61

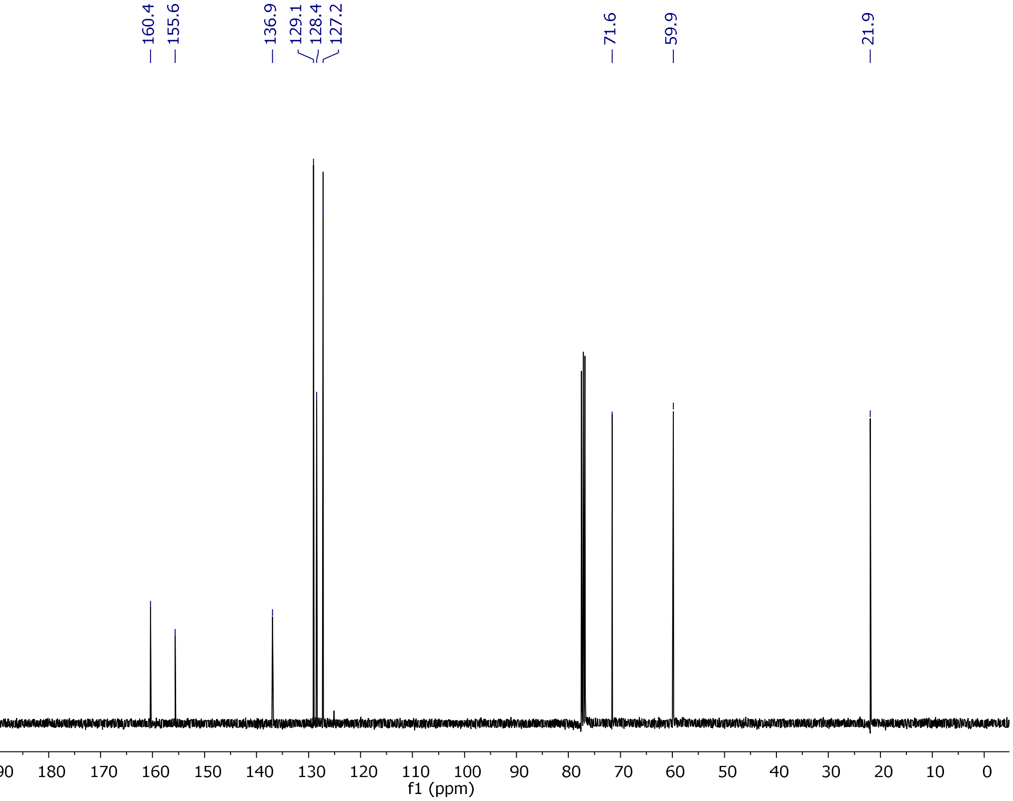
160.4
155.6
136.9
129.1
128.4
127.2
71.6
59.9
21.9
210 200 190 180 170 160 150 140 130 120 110 100 90 80 70 60 50 40 30 20 10 0
f1 (ppm)

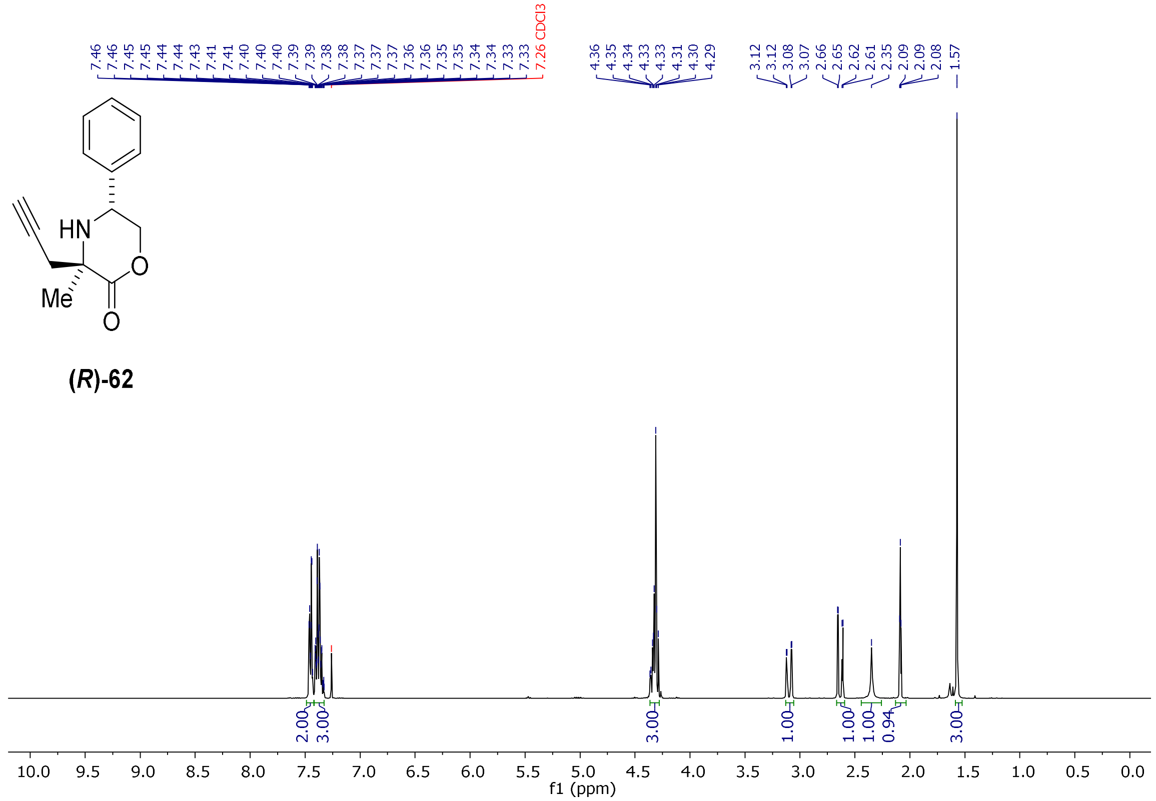
7.46 7.46 7.45 7.45 7.44 7.44 7.43 7.41 7.41 7.40 7.40 7.40 7.39 7.39 7.38 7.38 7.37 7.37 7.37 7.36 7.36 7.35 7.35 7.34 7.34 7.33 7.33
7.26 CDCl3
4.36 4.35 4.34 4.33 4.33 4.31 4.30 4.29
3.12 3.12 3.08 3.07 2.66 2.65 2.62 2.61 2.35 2.09 2.09 2.08
1.57
HN
O
Me
O
(R)-62
2.00
3.00
3.00
1.00
1.00
1.00
0.94
3.00
10.0 9.5 9.0 8.5 8.0 7.5 7.0 6.5 6.0 5.5 5.0 4.5 4.0 3.5 3.0 2.5 2.0 1.5 1.0 0.5 0.0
f1 (ppm)

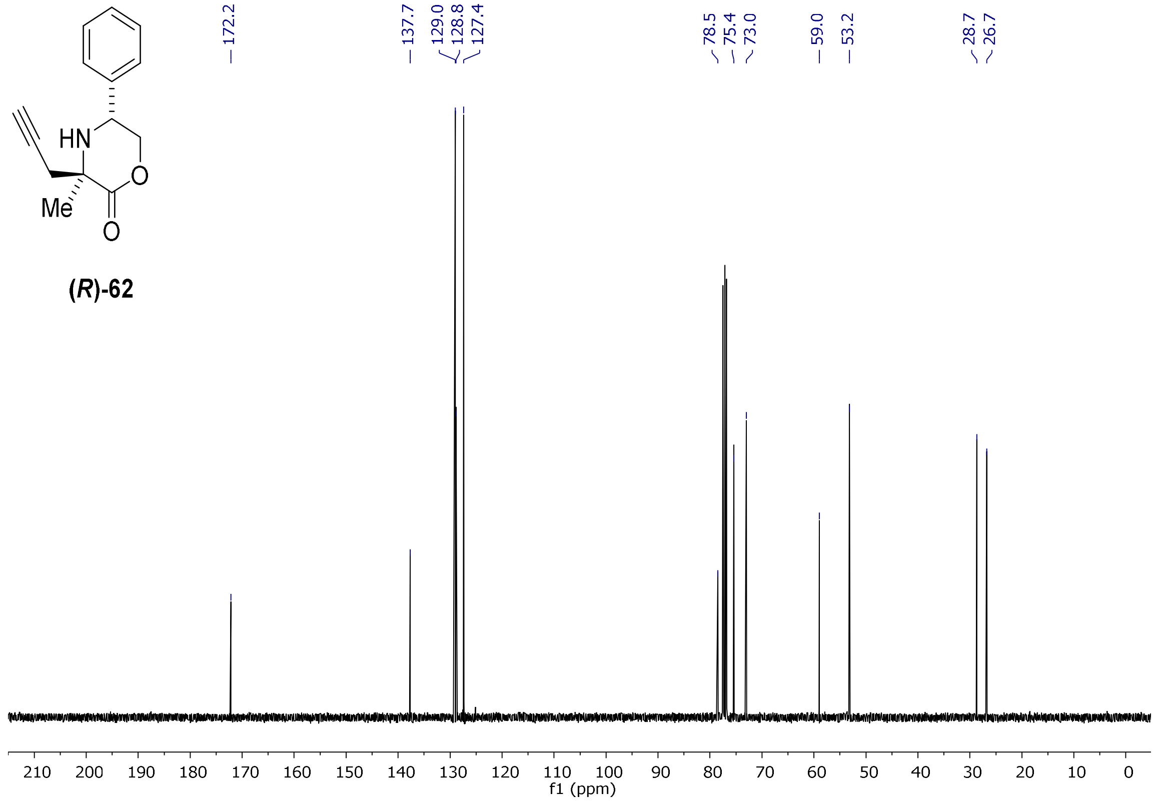
172.2
137.7
129.0
128.8
127.4
78.5
75.4
73.0
59.0
53.2
28.7
26.7
HN
O
Me
O
(R)-62
210 200 190 180 170 160 150 140 130 120 110 100 90 80 70 60 50 40 30 20 10 0
f1 (ppm)

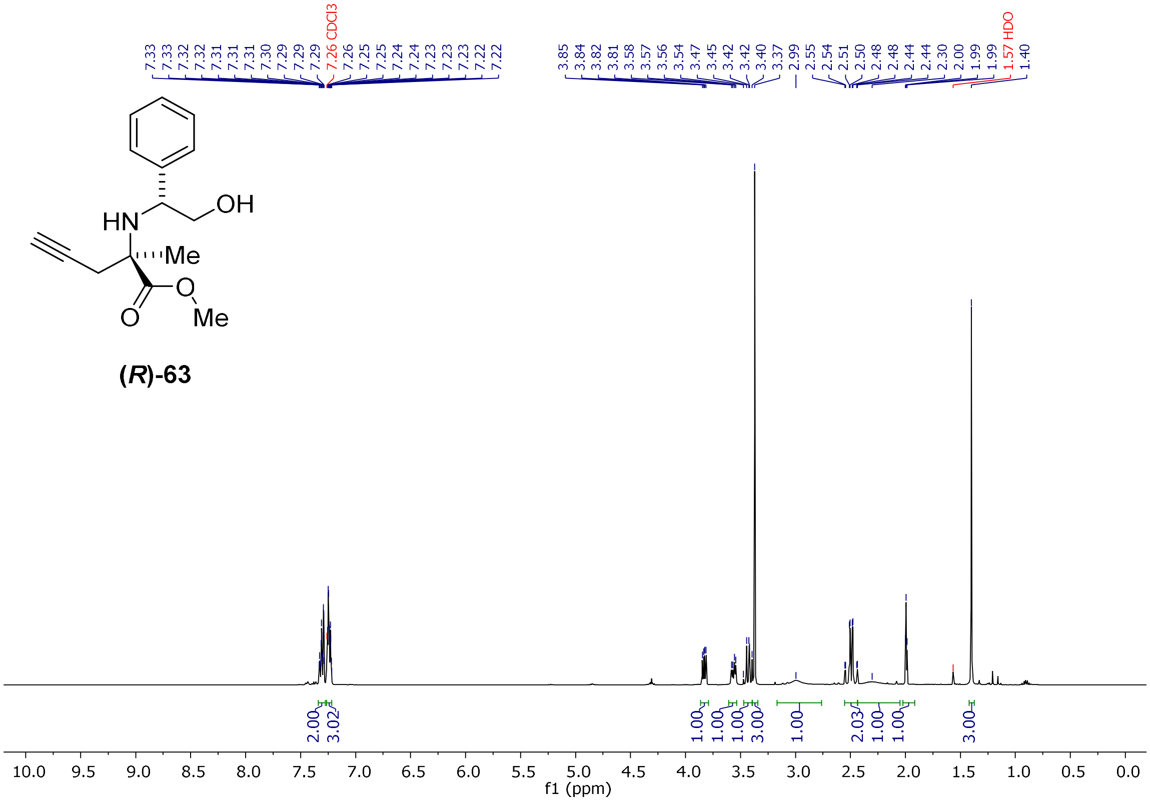
7.33 7.33 7.32 7.32 7.31 7.31 7.31 7.30 7.29 7.29 7.29 7.26 CDCl3 7.26 7.25 7.25 7.24 7.24 7.23 7.23 7.23 7.22 7.22
3.85 3.84 3.82 3.81 3.58 3.57 3.56 3.54 3.47 3.45 3.42 3.40 3.37 2.99 2.55 2.54 2.51 2.50 2.48 2.48 2.44 2.44 2.30 2.00 1.99 1.99 1.57 HDO 1.40
HN
OH
Me
O
O
Me
(R)-63
2.00 3.02 1.00 1.00 1.00 3.00 1.00 2.03 1.00 1.00 3.00
f1 (ppm)

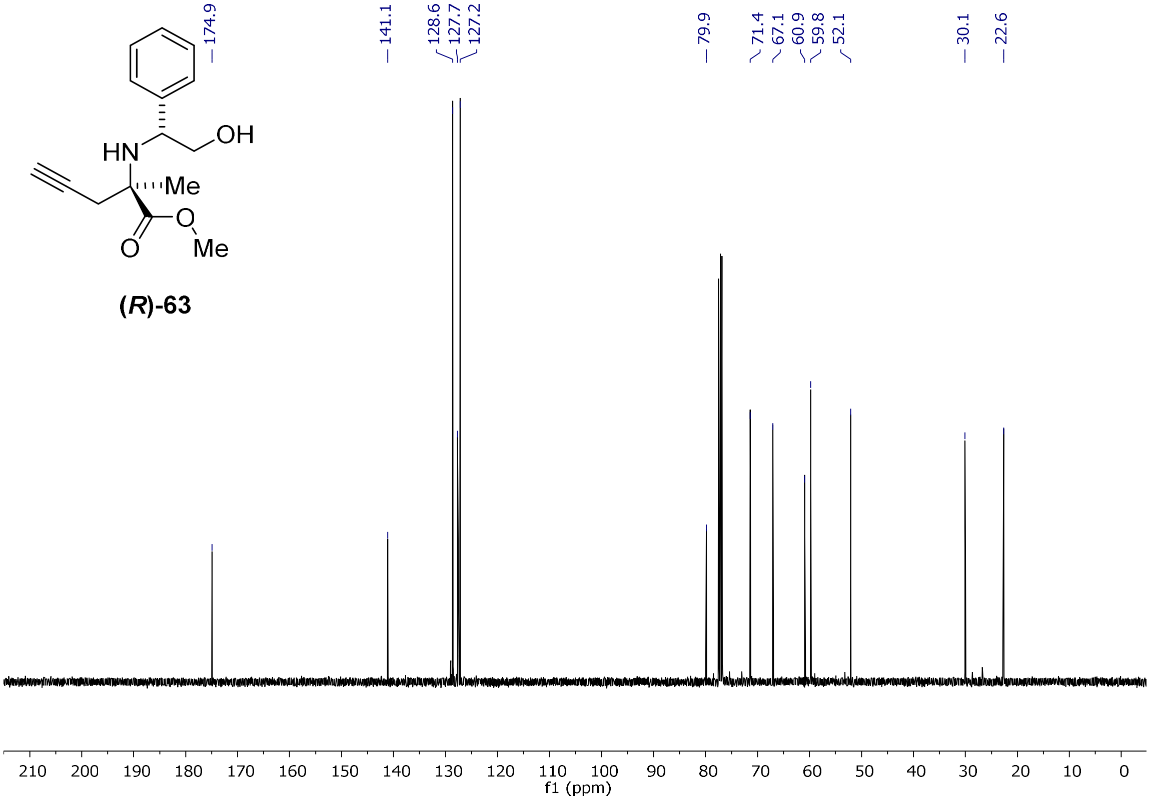
174.9 141.1 128.6 127.7 127.2 79.9 71.4 67.1 60.9 59.8 52.1 30.1 22.6
HN
OH
Me
O
O
Me
(R)-63
f1 (ppm)

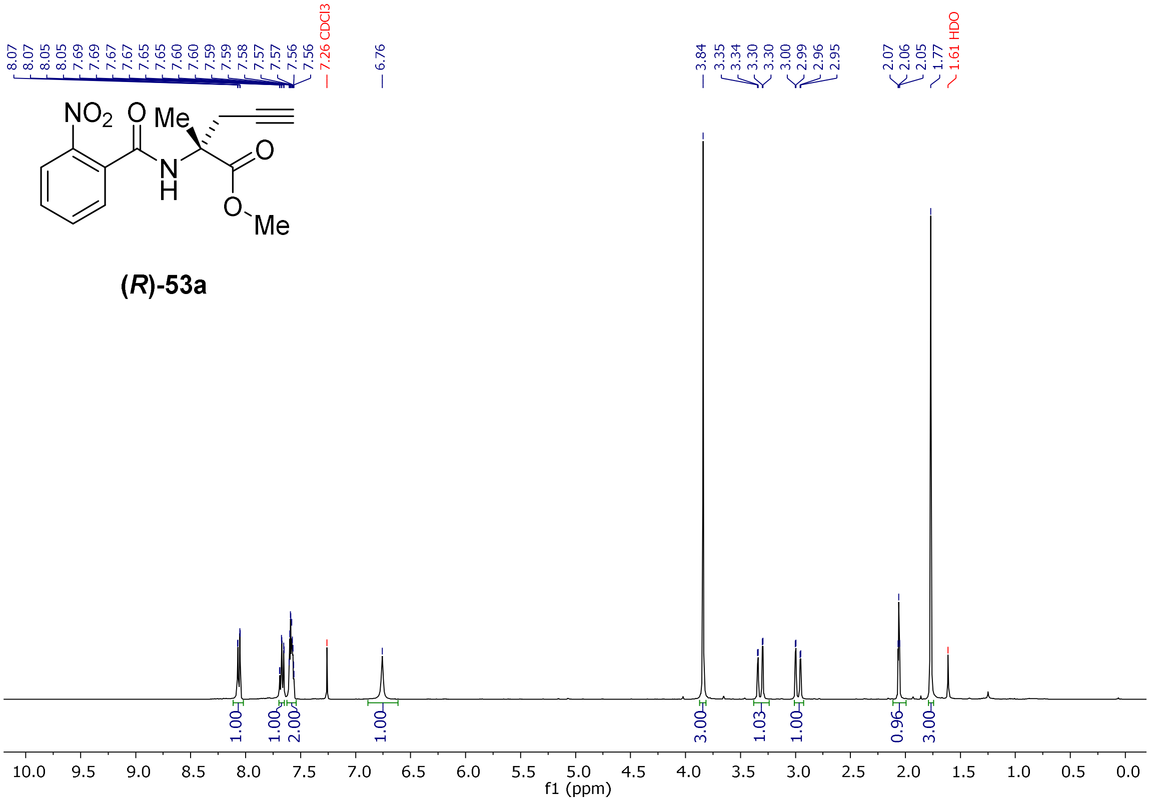
8.07 8.07 8.05 8.05 7.69 7.69 7.67 7.67 7.65 7.65 7.60 7.60 7.59 7.59 7.58 7.57 7.57 7.56 7.56
7.26 CDCl3
6.76
3.84 3.35 3.34 3.30 3.30 3.00 2.99 2.96 2.95
2.07 2.06 2.05 1.77
1.61 HDO
NO_2 O Me
N H
O
O Me
(*R*)-53a
1.00 1.00 2.00 1.00 3.00 1.03 1.00 0.96 3.00
10.0 9.5 9.0 8.5 8.0 7.5 7.0 6.5 6.0 5.5 5.0 4.5 4.0 3.5 3.0 2.5 2.0 1.5 1.0 0.5 0.0
f1 (ppm)

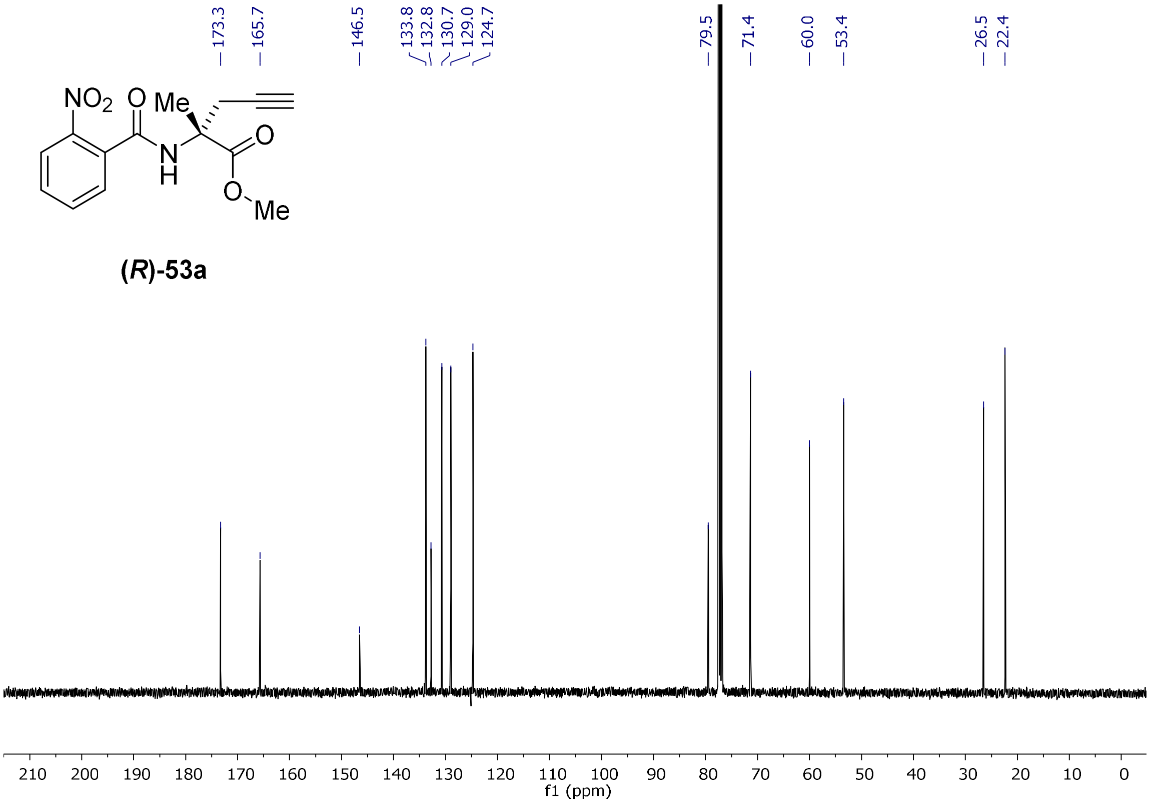
173.3
165.7
146.5
133.8 132.8 130.7 129.0 124.7
79.5
71.4
60.0
53.4
26.5 22.4
NO_2 O Me
N H
O
O Me
(*R*)-53a
210 200 190 180 170 160 150 140 130 120 110 100 90 80 70 60 50 40 30 20 10 0
f1 (ppm)

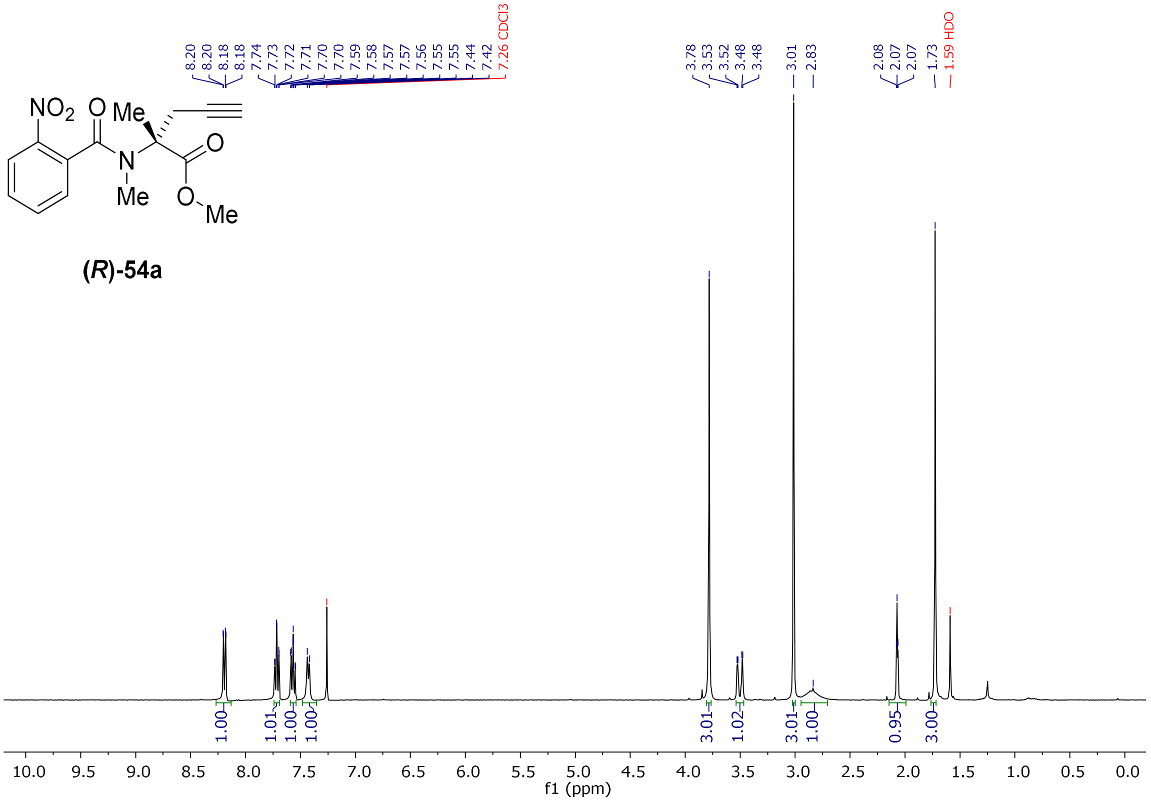

8.20 8.20 8.18 8.18 7.74 7.73 7.72 7.71 7.70 7.70 7.59 7.58 7.57 7.57 7.56 7.55 7.55 7.44 7.42
7.26 CDCl3
3.78 3.53 3.52 3.48 3.48
3.01
2.83
2.08 2.07 2.07
1.73
1.59 HDO
NO2 O Me N Me O O Me
(R)-54a
1.00 1.01 1.00 1.00 3.01 1.02 3.01 1.00 0.95 3.00
10.0 9.5 9.0 8.5 8.0 7.5 7.0 6.5 6.0 5.5 5.0 4.5 4.0 3.5 3.0 2.5 2.0 1.5 1.0 0.5 0.0
f1 (ppm)

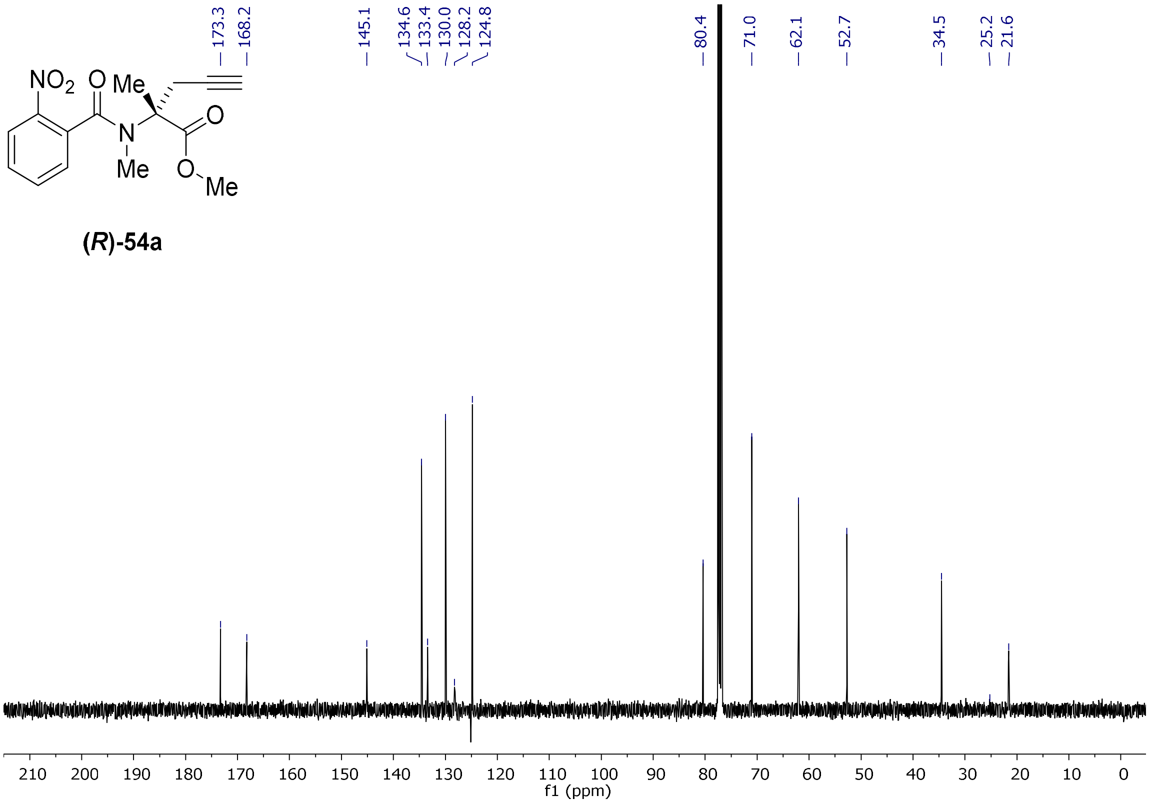

173.3
168.2
145.1
134.6 133.4 130.0 128.2 124.8
80.4
71.0
62.1
52.7
34.5
25.2 21.6
NO2 O Me N Me O O Me
(R)-54a
210 200 190 180 170 160 150 140 130 120 110 100 90 80 70 60 50 40 30 20 10 0
f1 (ppm)

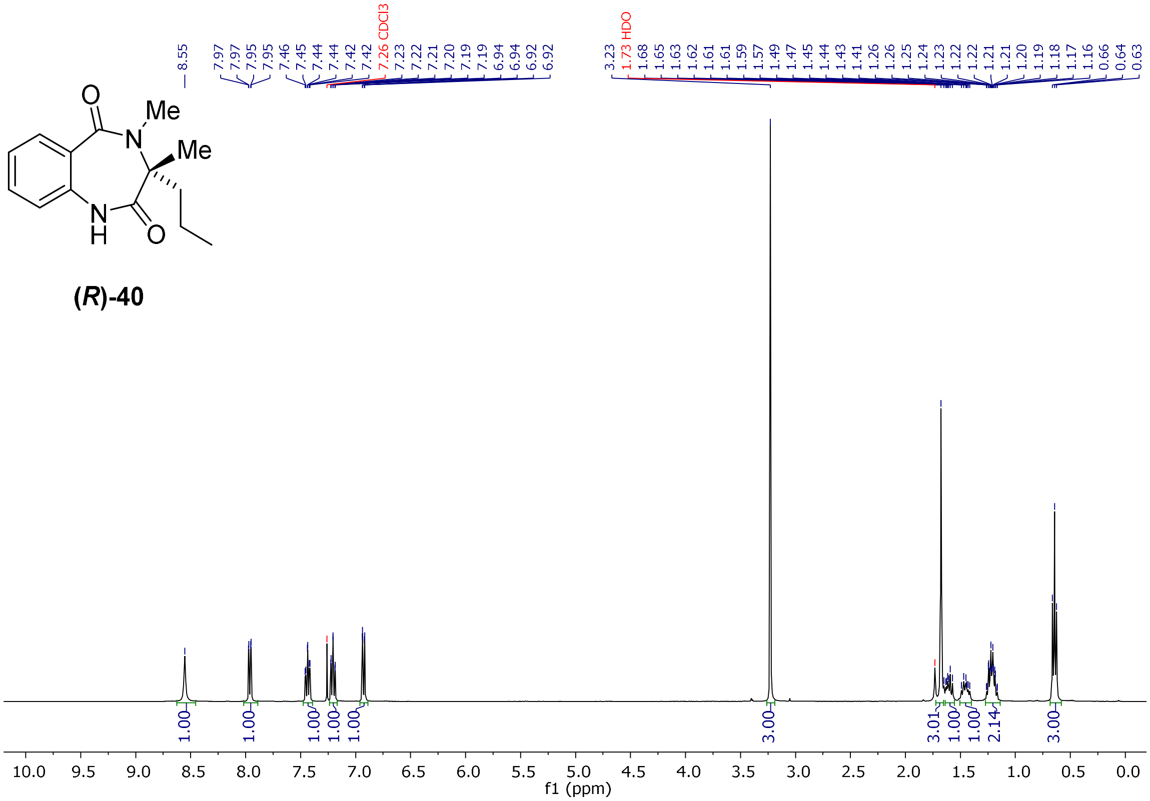
(R)-40
f1 (ppm)

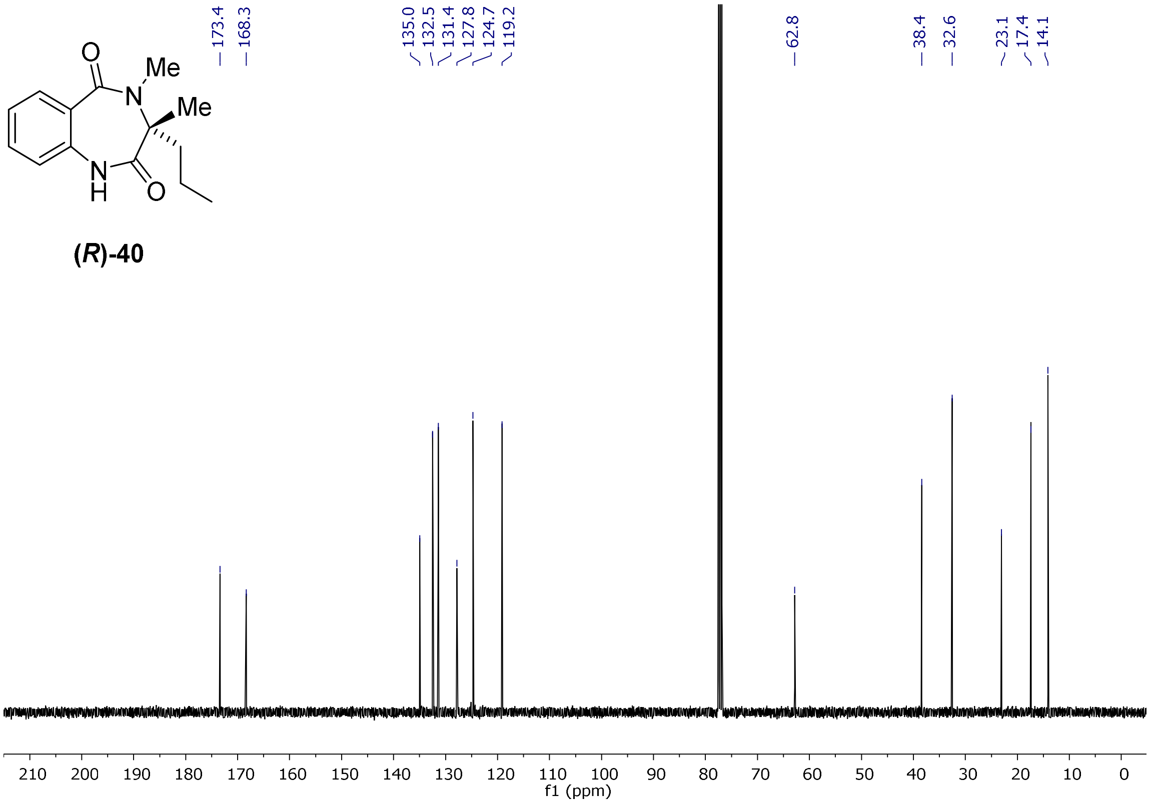
(R)-40
f1 (ppm)

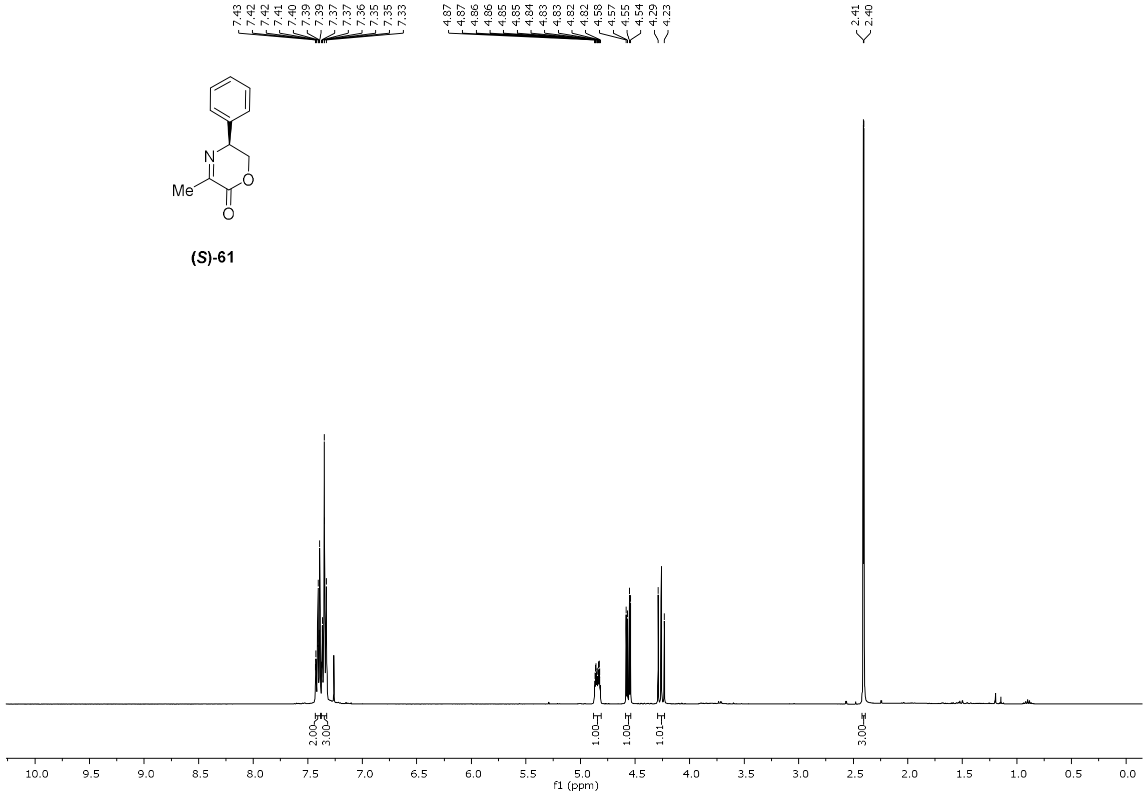
7.43 7.42 7.42 7.41 7.40 7.39 7.39 7.37 7.37 7.36 7.35 7.35 7.33
4.87 4.87 4.86 4.86 4.85 4.85 4.84 4.83 4.83 4.82 4.82 4.58 4.57 4.55 4.54 4.29 4.23
2.41 2.40
N
O
Me
O
2.00 3.00 1.00 1.00 1.01 3.00
10.0 9.5 9.0 8.5 8.0 7.5 7.0 6.5 6.0 5.5 5.0 4.5 4.0 3.5 3.0 2.5 2.0 1.5 1.0 0.5 0.0
f1 (ppm)

(S)-61

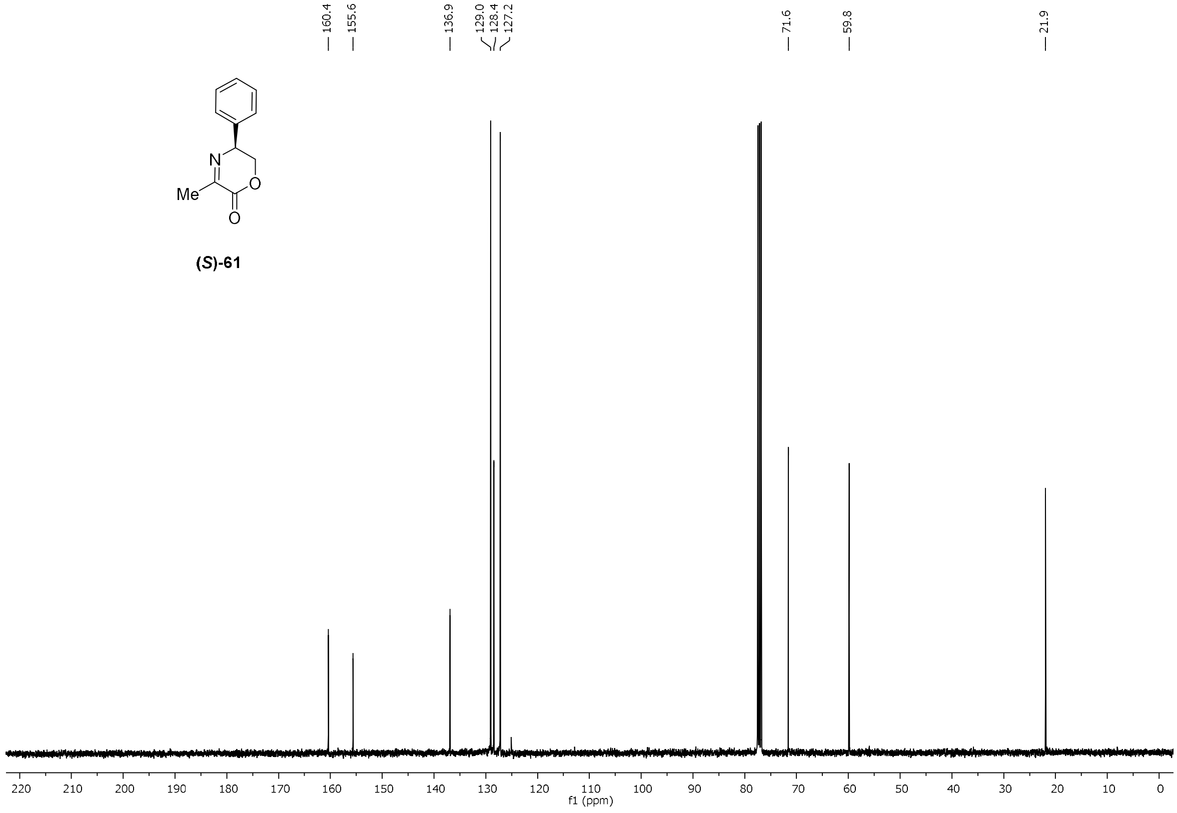
160.4 155.6 136.9 129.0 128.4 127.2 71.6 59.8 21.9
N
O
Me
O
220 210 200 190 180 170 160 150 140 130 120 110 100 90 80 70 60 50 40 30 20 10 0
f1 (ppm)

(S)-61

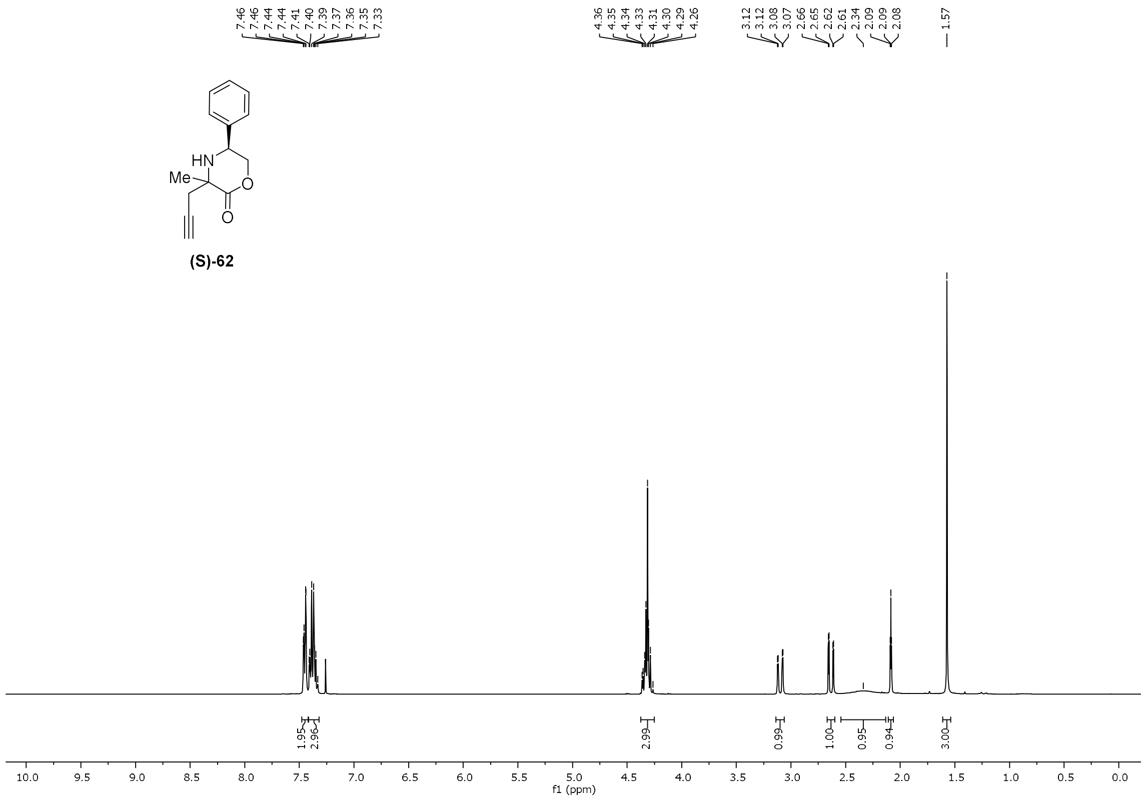
7.46 7.46 7.44 7.44 7.41 7.40 7.39 7.37 7.36 7.35 7.33
4.36 4.35 4.34 4.33 4.31 4.30 4.29 4.26
3.12 3.12 3.08 3.07 2.66 2.65 2.62 2.61 2.34 2.09 2.09 2.08
1.57
HN
Me
O
O
(S)-62
1.95 2.96 2.99 0.99 1.00 0.95 0.94 3.00
10.0 9.5 9.0 8.5 8.0 7.5 7.0 6.5 6.0 5.5 5.0 4.5 4.0 3.5 3.0 2.5 2.0 1.5 1.0 0.5 0.0
f1 (ppm)

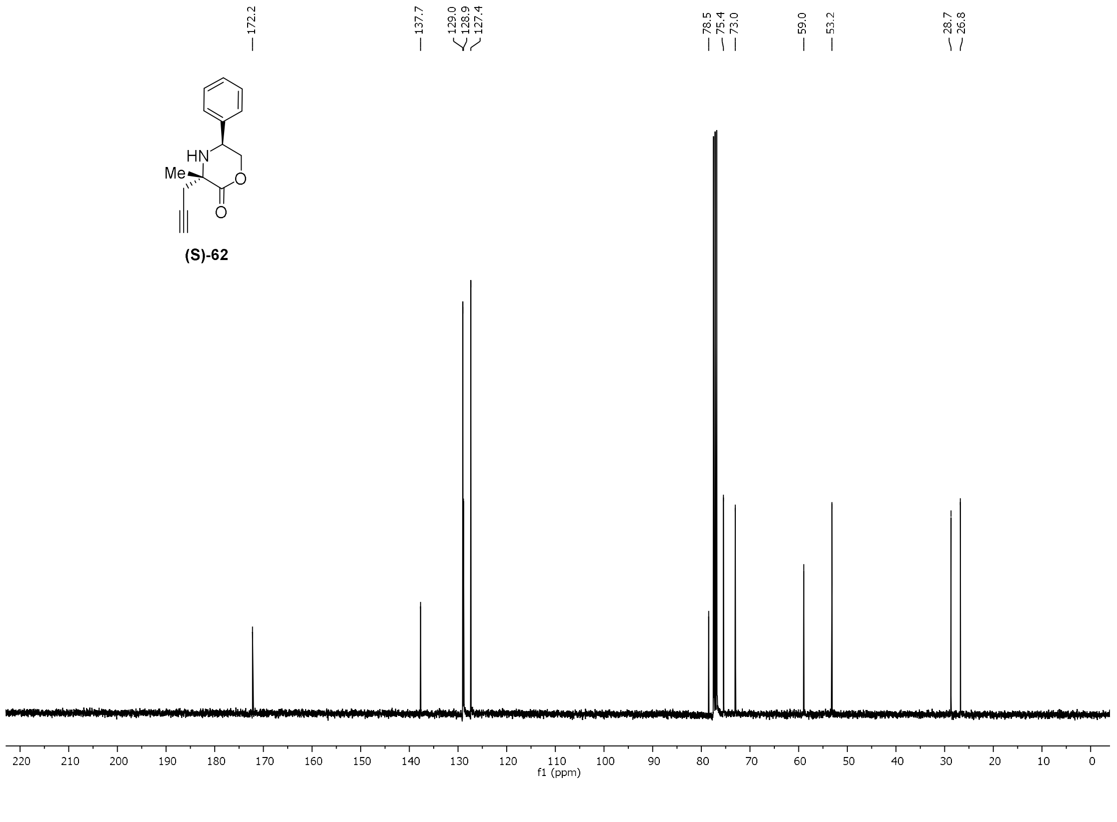
172.2
137.7
129.0 128.9 127.4
78.5 75.4 73.0
59.0
53.2
28.7 26.8
HN
Me
O
O
(S)-62
220 210 200 190 180 170 160 150 140 130 120 110 100 90 80 70 60 50 40 30 20 10 0
f1 (ppm)

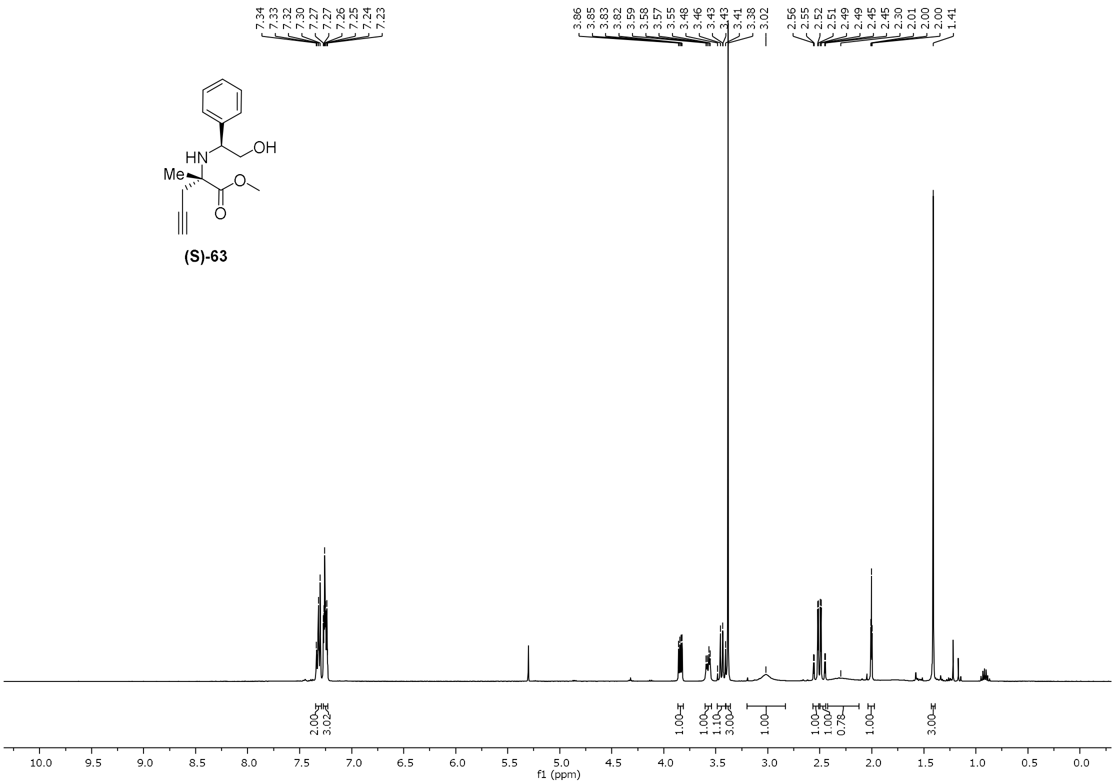
7.34 7.33 7.32 7.30 7.27 7.27 7.26 7.25 7.24 7.23
3.86 3.85 3.83 3.82 3.59 3.58 3.57 3.55 3.48 3.46 3.43 3.43 3.41 3.38 3.02
2.56 2.55 2.52 2.51 2.49 2.49 2.45 2.45 2.30 2.01 2.00 2.00 1.41
HN
OH
Me
O
O
(S)-63
2.00 3.02
1.00 1.00 1.10 3.00 1.00 1.00 1.00 0.78 1.00 3.00
f1 (ppm)

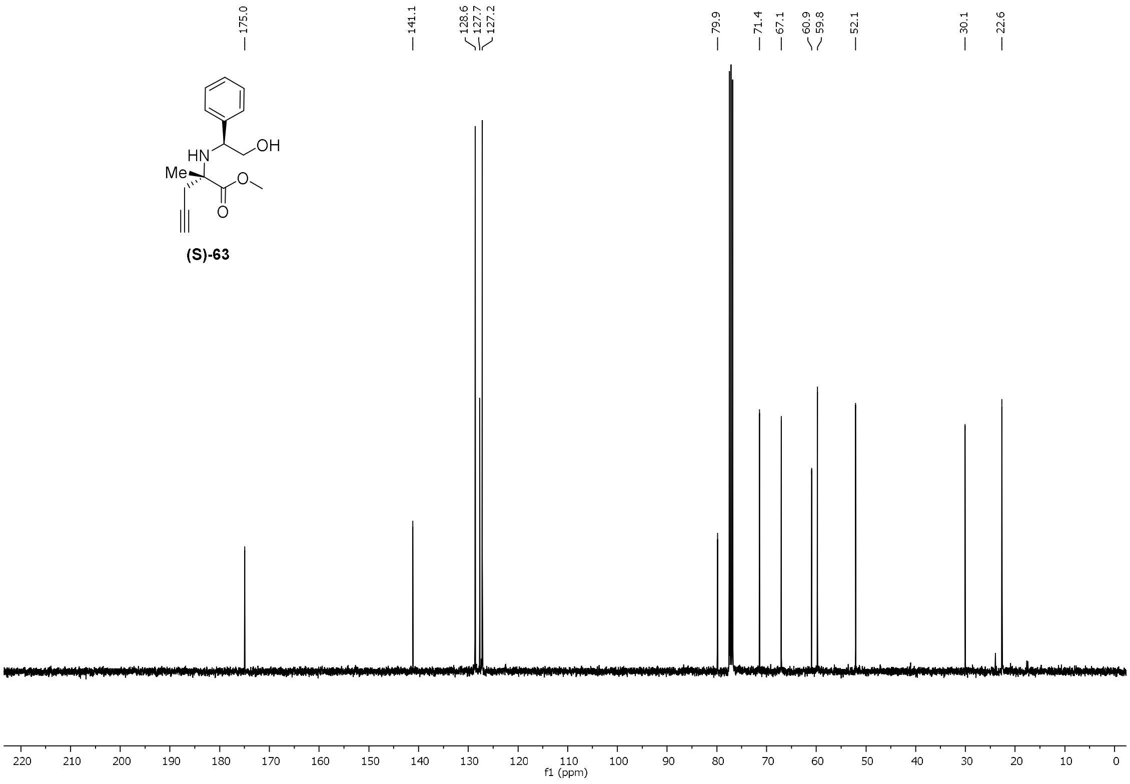
175.0
141.1
128.6 127.7 127.2
79.9
71.4
67.1
60.9 59.8
52.1
30.1
22.6
HN
OH
Me
O
O
(S)-63
f1 (ppm)

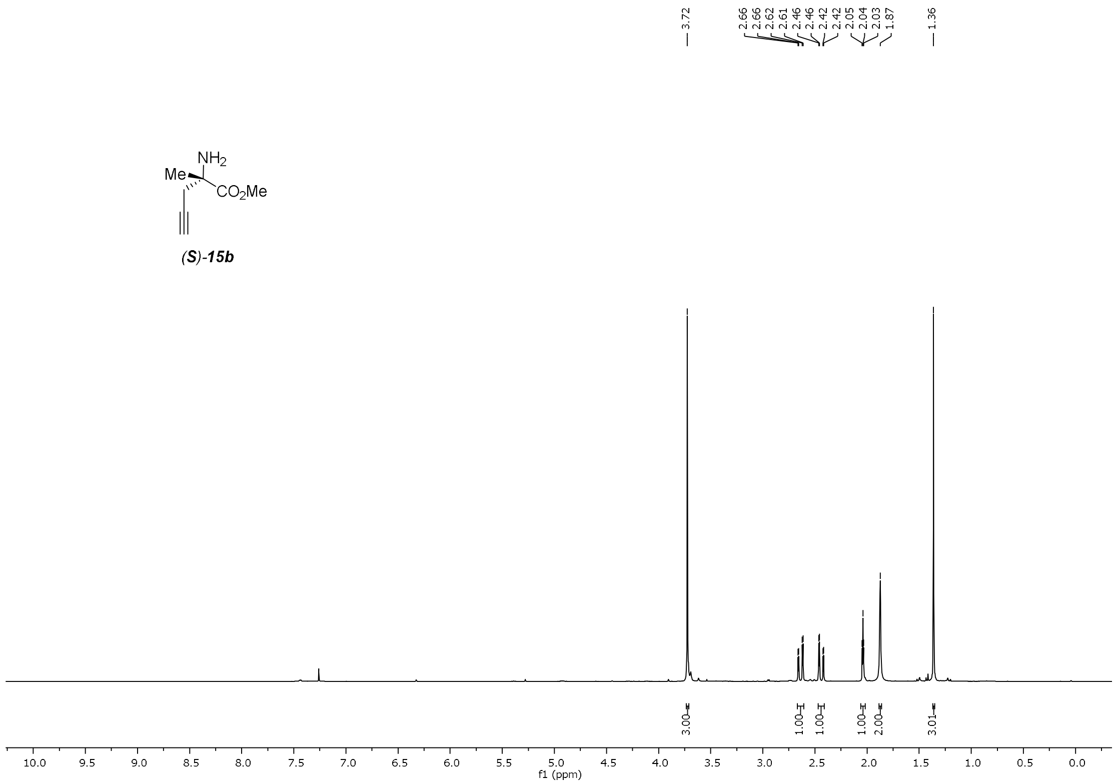
3.72
2.66
2.66
2.62
2.61
2.46
2.46
2.42
2.42
2.05
2.04
2.03
1.87
1.36
NH2
Me
CO2Me
(S)-15b
3.00
1.00
1.00
1.00
2.00
3.01
10.0 9.5 9.0 8.5 8.0 7.5 7.0 6.5 6.0 5.5 5.0 4.5 4.0 3.5 3.0 2.5 2.0 1.5 1.0 0.5 0.0
f1 (ppm)

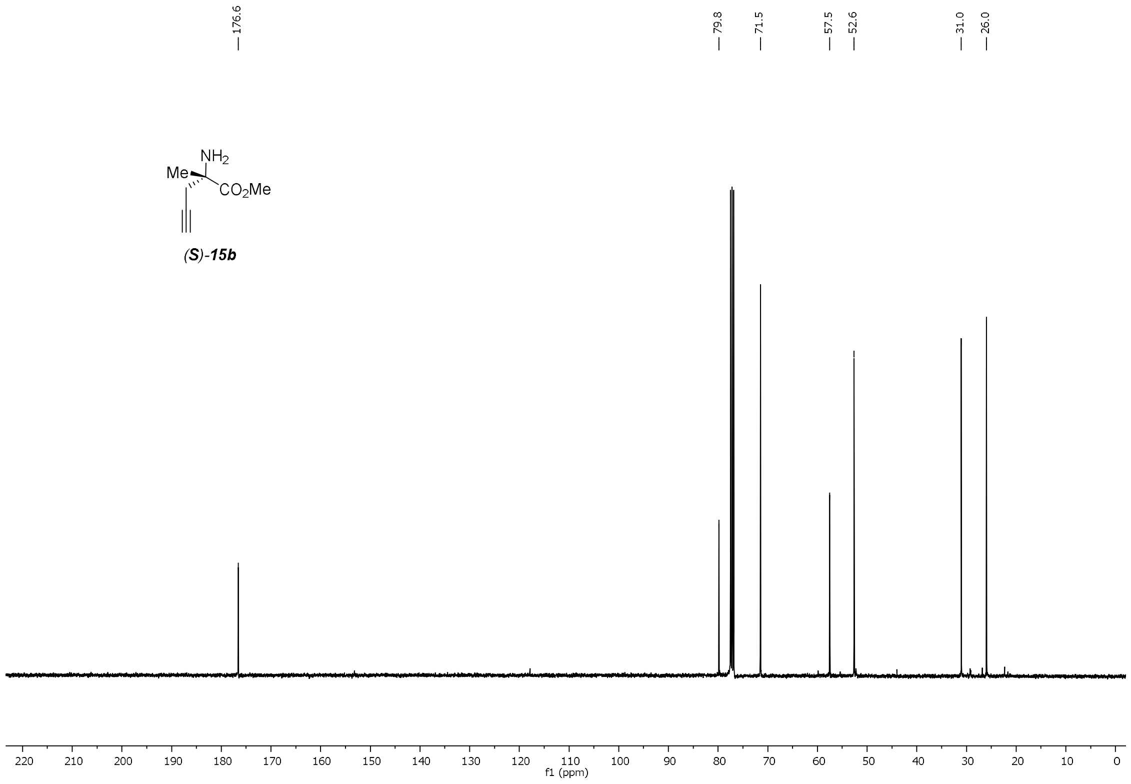
176.6
79.8
71.5
57.5
52.6
31.0
26.0
NH2
Me
CO2Me
(S)-15b
220 210 200 190 180 170 160 150 140 130 120 110 100 90 80 70 60 50 40 30 20 10 0
f1 (ppm)

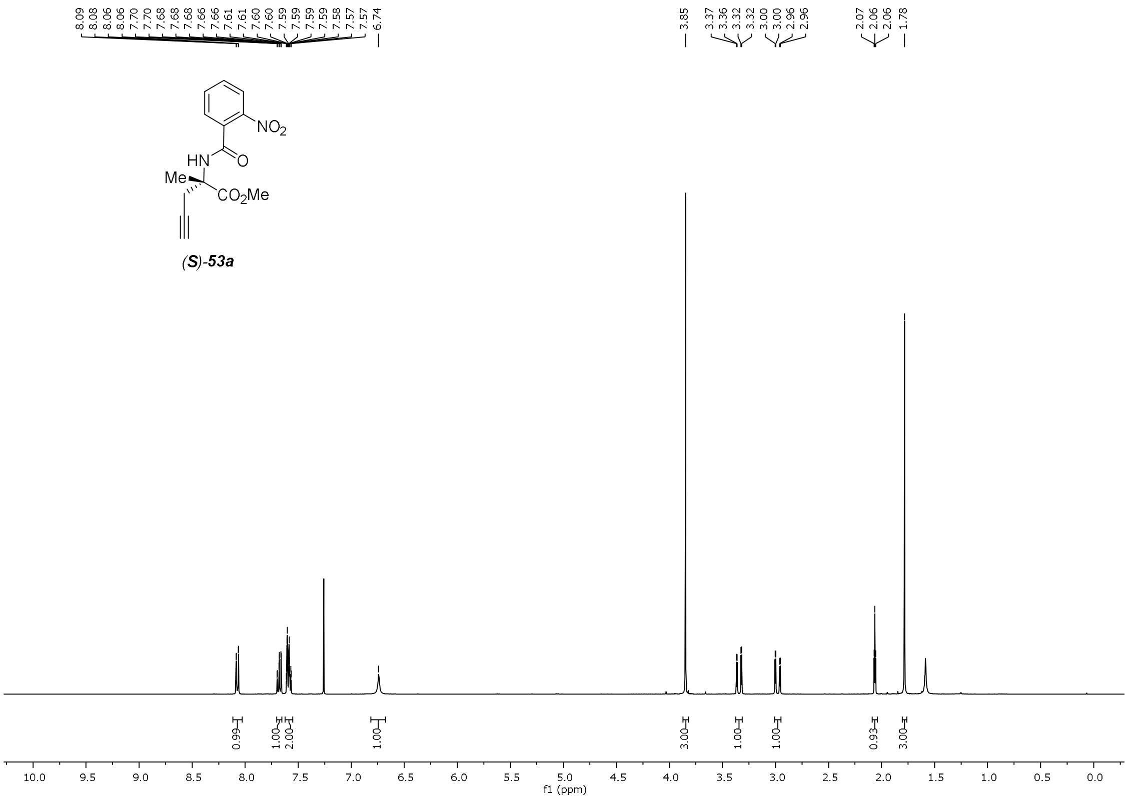
8.09 8.08 8.06 8.06 7.70 7.70 7.68 7.68 7.68 7.66 7.66 7.61 7.61 7.60 7.60 7.59 7.59 7.59 7.59 7.58 7.57 7.57 6.74
3.85
3.37 3.36 3.32 3.32 3.00 3.00 2.96 2.96
2.07 2.06 2.06 1.78
NO2
HN
O
Me
CO2Me
0.99
1.00
2.00
1.00
3.00
1.00
1.00
0.93
3.00
f1 (ppm)

(**S**)-**53a**

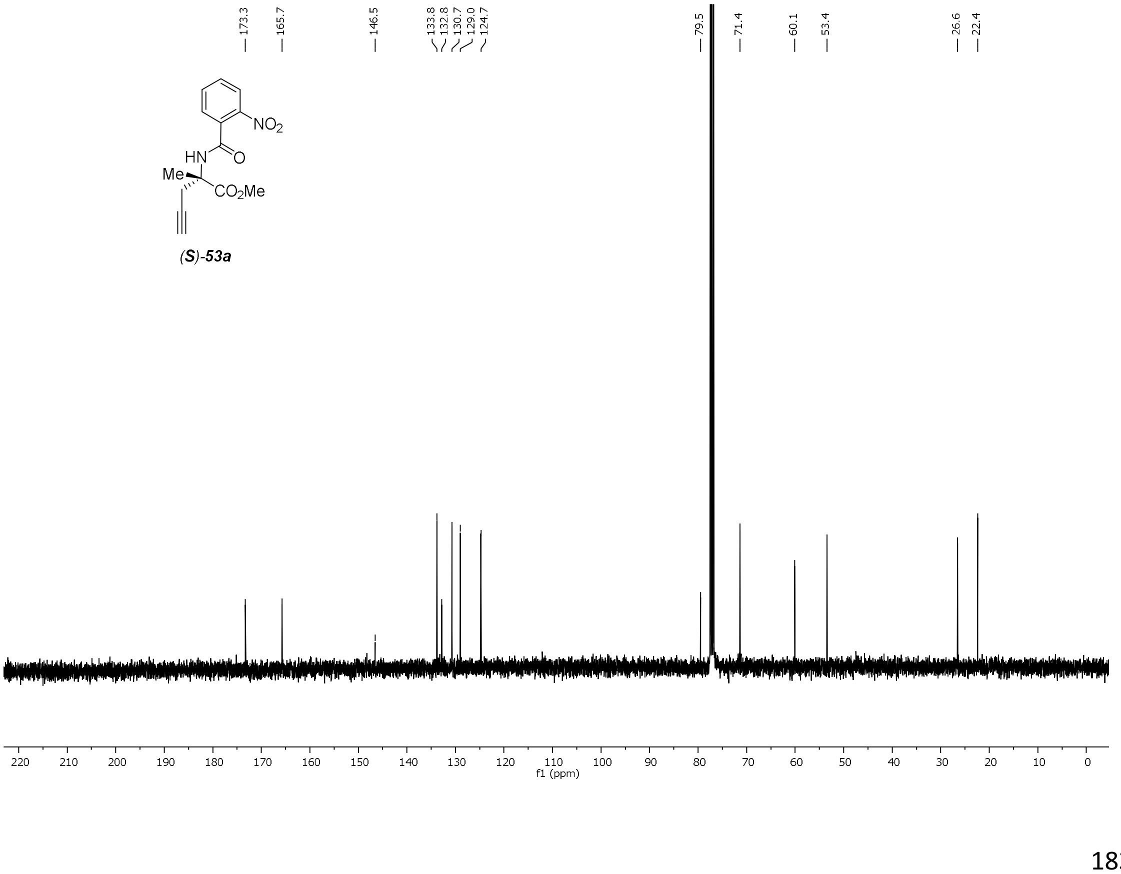
173.3
165.7
146.5
133.8 132.8 130.7 129.0 124.7
79.5
71.4
60.1
53.4
26.6
22.4
NO2
HN
O
Me
CO2Me
f1 (ppm)

(**S**)-**53a**

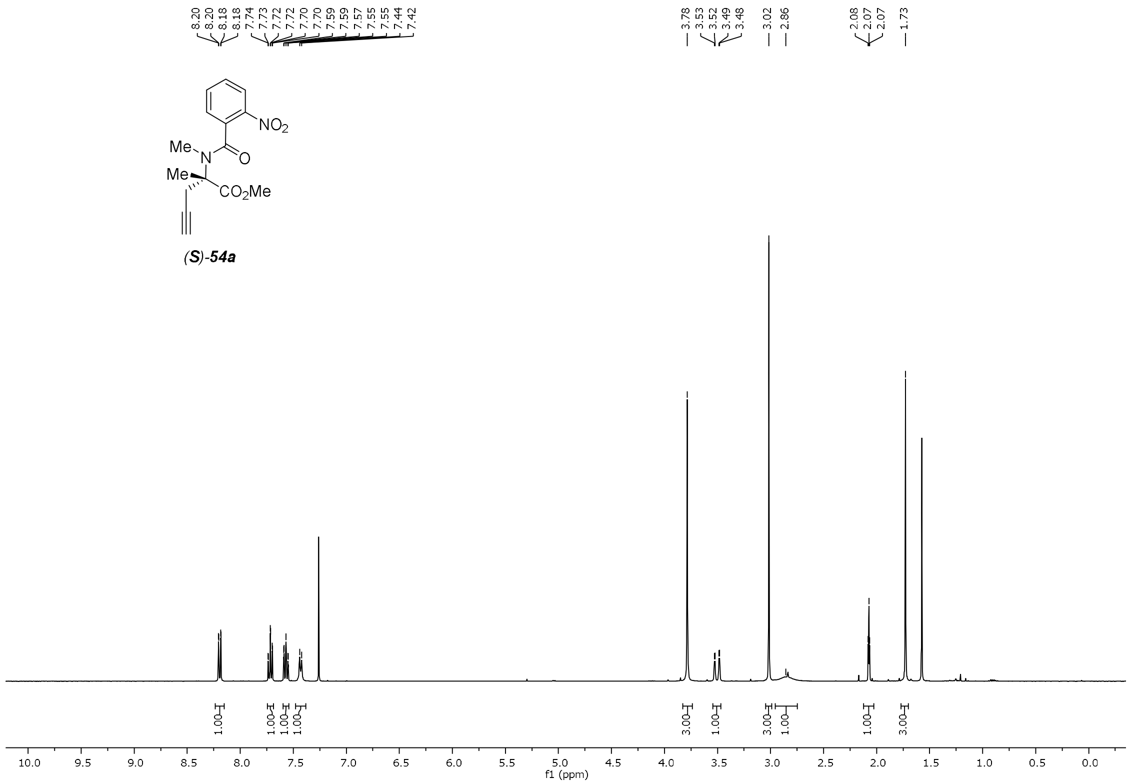
8.20
8.20
8.18
8.18
7.74
7.73
7.72
7.72
7.70
7.70
7.59
7.59
7.57
7.55
7.55
7.44
7.42
3.78
3.53
3.52
3.49
3.48
3.02
2.86
2.08
2.07
2.07
1.73
NO2
Me
N
O
Me
CO2Me
(S)-54a
1.00
1.00
1.00
1.00
3.00
1.00
3.00
1.00
1.00
3.00
f1 (ppm)

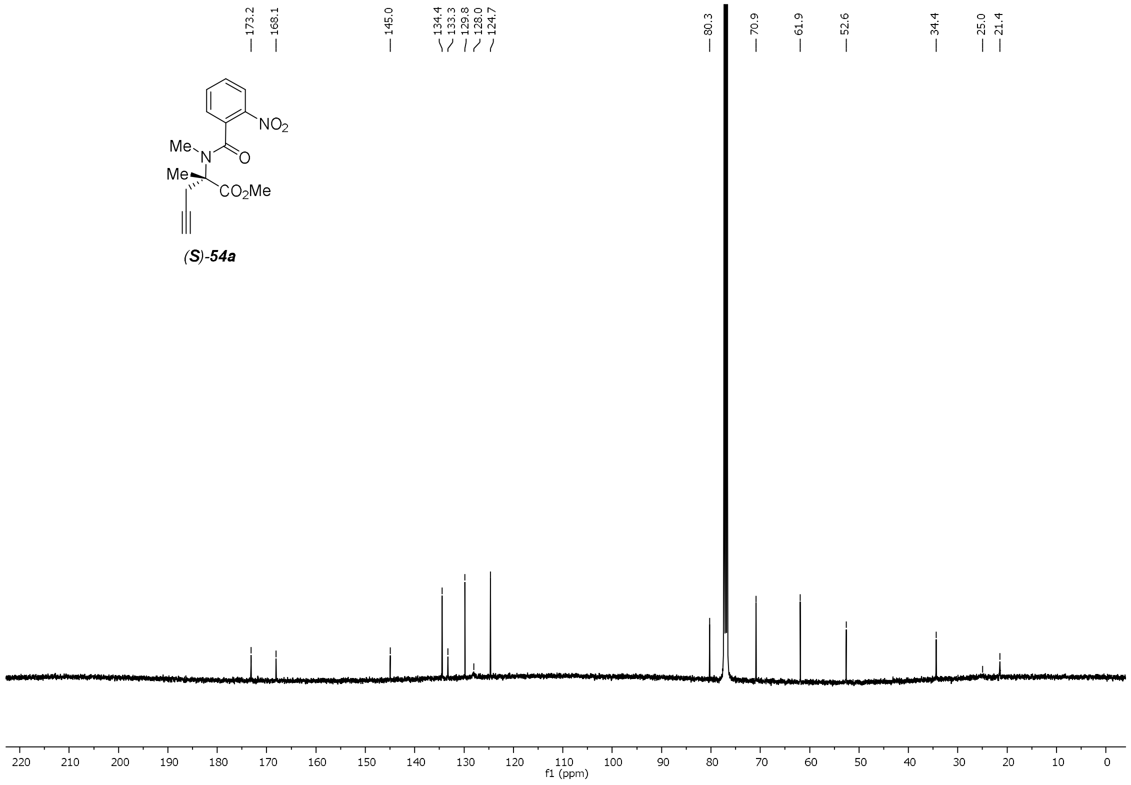
173.2
168.1
145.0
134.4
133.3
129.8
128.0
124.7
80.3
70.9
61.9
52.6
34.4
25.0
21.4
NO2
Me
N
O
Me
CO2Me
(S)-54a
f1 (ppm)

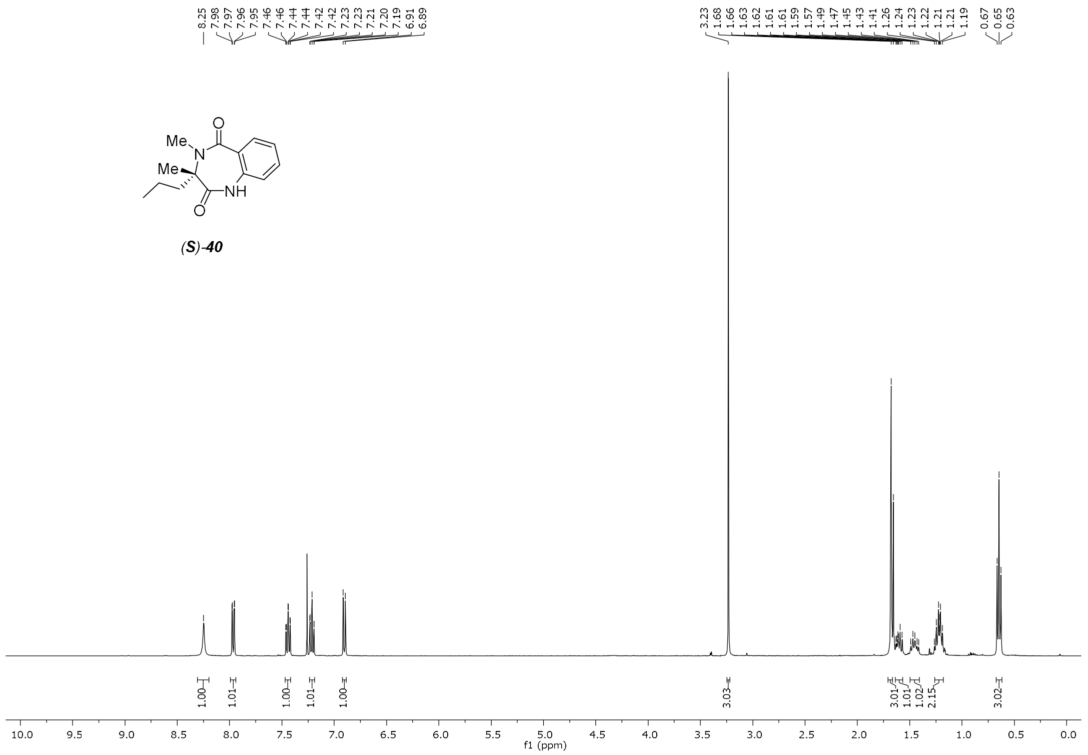
(S)-40
f1 (ppm)

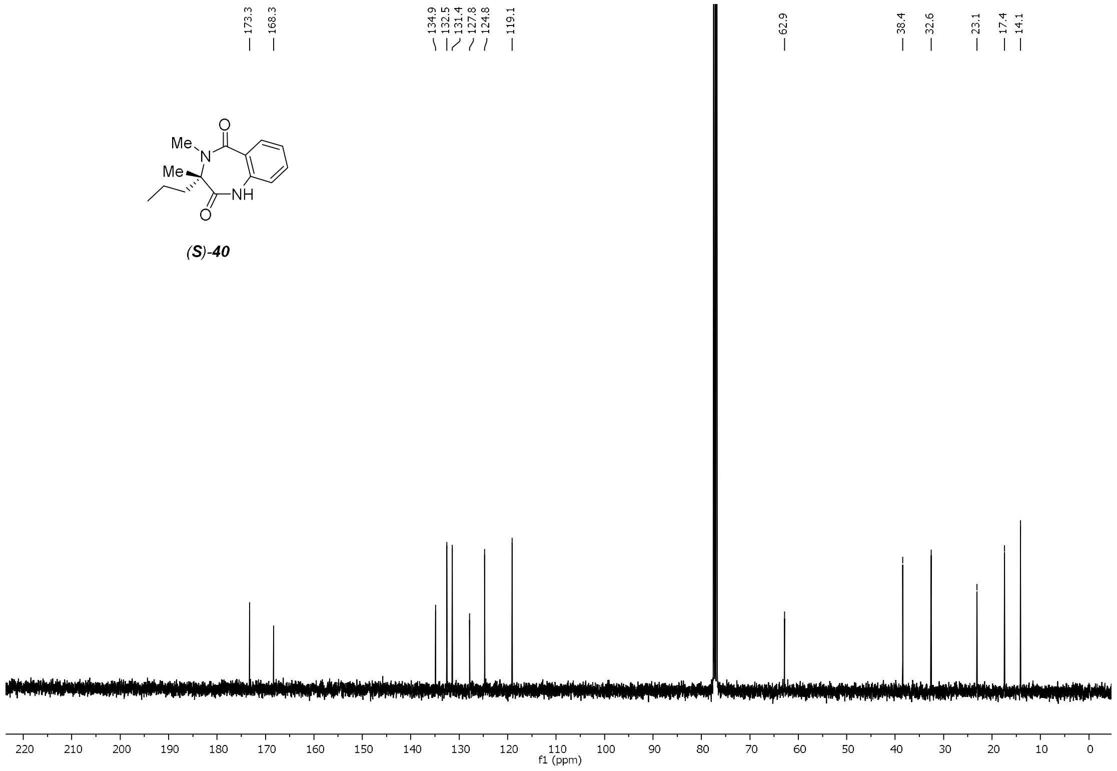
(S)-40
f1 (ppm)
